# Effect Of XBP1 Deficiency In Cartilage On The Regulatory Network Of LncRNA/circRNA-miRNA-mRNA

**DOI:** 10.7150/ijbs.64054

**Published:** 2022-01-01

**Authors:** Xiaoli Li, Yuyou Yang, Li Liang, Mengtian Fan, Xingyue Li, Naibo Feng, Yiming Pan, Qiaoyan Tan, Qingbo Xu, Yangli Xie, Fengjin Guo

**Affiliations:** 1 Department of Cell Biology and Genetics, Core Facility of Development Biology, Chongqing Medical University, Chongqing 400016, China.; 2 Department of Wound Repair and Rehabilitation Medicine, State Key Laboratory of Trauma, Burns and Combined Injury, Trauma Center, Research Institute of Surgery, Daping Hospital, Army Medical University, Chongqing 400042, China.; 3 Cardiovascular Division, King's College London BHF Centre, London, United Kingdom.

**Keywords:** XBP1, whole transcriptome analysis, cartilage, circRNA-miRNA-mRNA, lncRNA-miRNA-mRNA

## Abstract

X-box binding protein 1(XBP1) is a critical component for unfolded protein response (UPR) in ER stress. According to previous studies performed with different XBP1-deficient mice, the *XBP1* gene affects mouse cartilage development and causes other related diseases. However, how the complete transcriptome, including mRNA and ncRNAs, affects the function of cartilage and other tissues when *XBP1* is deficient in chondrocytes is unclear. In this study, we aimed to screen the differentially expressed (DE) mRNAs, circRNAs, lncRNAs and miRNAs in XBP1 cartilage-specific knockout (CKO) mice using high throughput sequencing and construct the circRNA-miRNA-mRNA and lncRNA-miRNA-mRNA regulatory networks. DE LncRNAs (DE-LncRNAs), circRNAs (DE-circRNAs), miRNAs (DE-miRNAs), and mRNAs [differentially expressed genes (DEGs)] between the cartilage tissue of XBP1 CKO mice and controls were identified, including 441 DE-LncRNAs, 15 DE-circRNAs, 6 DE-miRNAs, and 477 DEGs. Further, 253,235 lncRNA-miRNA-mRNA networks and 1,822 circRNA-miRNA-mRNA networks were constructed based on the correlation between lncRNAs/circRNAs, miRNAs, mRNAs. The whole transcriptome analysis revealed that XBP1 deficiency in cartilage affects the function of cartilage and other different tissues, as well as associated diseases. Overall, our findings may provide potential biomarkers and mechanisms for the diagnosis and treatment of cartilage and other related diseases.

## Introduction

Abnormal chondrocyte differentiation is an important cause of common orthopedic diseases, such as rickets, dwarfism, and osteoarthritis 1, 2. During endoplasmic reticulum stress, IRE1α undergoes autophosphorylation and activation, and the XBP1u mRNA is spliced in the cytoplasm to produce the transcription factor, XBP1s, with multiple regulatory functions. This transcription factor enters the nucleus to regulate the transcription and expression of its associated gene, and participates in several pathophysiological processes by regulating cell growth and apoptosis, differentiation and development, immune response, among others 3-6. The unfolded protein response (UPR) signaling pathway, inositol-requiring enzyme 1 (IRE1)-XBP1, which is mediated by XBP1s, is an important pathway in the determination of cell fate and a key pathway for plasma cell differentiation 3-5. According to our previous studies, XBP1s is expressed in the proliferation and hypertrophy stages of chondrocyte differentiation and is involved in the regulation of chondrocyte differentiation and cartilage development in chondrocytes. As a BMP2-inducible transcription factor, XBP1s positively regulates endochondral bone formation by activating GEP chondrogenic growth factor 6. Cameron et al. reported that the cartilage-specific deficiency of XBP1 in mouse leads to a chondrodysplasia characteristically defined by reduced chondrocyte proliferation and delayed cartilage maturation and mineralization. XBP1 was inferred to control chondrocyte proliferation and the timing of cartilage maturation and matrix mineralization during endochondral ossification 7, 8. As reported in many literatures, in eukaryotic genomes that transcribe up to 90% of genomic DNA, only 1-2% of the transcripts encode proteins, with most being transcribed as non-coding RNAs (ncRNAs). The number of ncRNAs increases significantly with the complexity of the organism. Although the expression level of most predicted ncRNAs is markedly lower than that of mRNAs, they do not affect their regulatory functions. Based on increasing evidence, ncRNA has a regulatory role in the development process and in the response to stress and environmental stimuli 9-14.

Non-coding RNAs play an important regulatory role in cells. In fact, these RNAs, can regulate several biological processes, including epigenetic modification, variable RNA splicing, and protein stability 15-17. For instance, rRNAs, tRNAs, small nuclear RNA (snRNAs), small nucleolar RNAs (snoRNAs) are involved in mRNA translation, splicing, modification of rRNAs, respectively 10. High-throughput sequencing (NGS) technology and bioinformatics analysis have led to the discovery of a remarkable number of circRNAs, lncRNAs, miRNAs, mRNAs and their networks 18. Long-noncoding RNAs (LncRNAs) are ncRNAs longer than 200 nt^19^. Previous studies have shown that lncRNAs play a crucial role in the regulation of disease processes and gene expression 20, 21. Further, lncRNAs are reported to participate in cell differentiation, migration, proliferation and apoptosis 22. For example, a novel osteogenesis-associated lncRNA (lncRNA-OG) significantly promotes BM-MSC osteogenesis. LncRNA-OG interacts with heterogeneous nuclear ribonucleoprotein K (hnRNPK) protein to regulate the activation of the bone morphogenetic protein signaling pathway. hnRNPK positively regulates the transcriptional activity of lncRNA-OG by promoting H3K27 acetylation of the lncRNA-OG promoter 23. CircRNAs is a class of ncRNAs widely expressed in eukaryotic cells. CircRNAs have a covalent closed circular configuration without 5' and 3' polarities and poly A tail structure 24. CircRNA can act as an miRNA sponge to affect the functions of miRNAs 25, and has a regulatory effect on several diseases, such as cancer, diabetes, cardiovascular diseases and certain immune diseases 26. MiR-124-3p is an essential microRNA (miRNA), and its expression changes are related to the proliferation ability of bone mesenchymal stem cells (BMSCs) 27. MiR-124-3p is also reported to be associated with osteoporosis (OP) and fragility fractures 28. RNAs have become a novel hotspot in research in cartilage development. Although some ncRNAs, including lncRNAs, circRNAs and miRNAs, have been identified in bone development and associated diseases, the ncRNAs related to the regulation of XBP1 have not been revealed.

The coordinated regulation between different tissues and organs is performed by the full spectrum of transcriptome RNAs, including mRNAs and ncRNAs. In the current study, we attempted to elucidate whether and how XBP1 deficiency affects the full spectrum of ncRNA functional characterization and the normal physiological functions of ncRNA and their role in human diseases. First, we conducted whole transcriptome sequencing of cartilage tissue obtained from XBP1 conditional knockout mice, and carried out a differential expression profile assay of circRNAs, lncRNAs, miRNAs and mRNAs. Thereafter, based on bioinformatics analysis, two competitive endogenous RNA (ceRNA) regulatory networks (the circRNA-miRNA-mRNA and lncRNA-miRNA-mRNA) were established, and GO and KEGG enrichment analyses were performed.

XBP1 deficiency in cartilage leads to differences in the complete transcriptome RNA of cartilage and other different tissues, including various ncRNAs with differential expression and regulatory profiles, as well as differences in protein expression and abnormalities of various different secreted factors. Our results support the identification of both the full spectrum of the function and characterization of ncRNAs induced by XBP1 deficiency in cartilage and the role of these XBP1-related ncRNAs in normal physiological functions and human diseases.

## Materials and Methods

### Animals and Cartilage Tissue Collection

The XBP1^flox/flox^ mice was a gift from Prof. Qingbo Xu (King's College London BHF Centre, London, United Kingdom), the Col2 Cre mice was a gift from Prof. Lin Chen (Third Military Medical University; Chongqing, China) 29, 30. The primer is the following: XBP1^flox/flox^-F: 5'-TGG CAA GGC TGA GCC TGA TCG-3'; XBP1^flox/flox^-R: 5'-GGA ACTAGAGATACC ACTGAG-3' (XBP1^flox/flox^ 315 bp; XBP1^flox/+^ 265bp and 315 bp; XBP1^+/+^ 265 bp); col2Cre-F: 5'- GAGGGTCCAGCCCGAGCTACTT-3', col2CRE-R: 5'-GCATCGACCGGTAATGCA GGC-3'. All the animal studies were performed in accordance with Chongqing Medical University Institutional Animal Care and Use Committee. Mice were group housed (25 °C) on a 12-hour light-dark cycle. All mice were maintained on a C57BL/6J background; genotyping of XBP1^flox/flox^ col2CRE^+^ (CKO) mice and control mice (XBP1^flox/flox^ col2CRE^-^) littermates was performed as described previously 29-31. Three pairs of littermates (XBP1^flox/flox^ col2CRE^+^ and XBP1^flox/flox^ col2CRE^-^) were sacrificed at 4 weeks and hyaline cartilage tissues were processed for full transcriptome RNA-seq.

### Total RNA Extraction and Quality Control

Total RNA from 6 samples was extracted using the TRIzol reagent (Invitrogen, Carlsbad, CA, USA). The concentration of total RNA was checked by using Qubit® RNA Assay Kit in Qubit® 2.0 Fluorometer, its purity was checked by using a NanoPhotometer spectrophotometer (IMPLEN, CA, USA), and its integrity was checked by Bioanalyzer 2100 system (Agilent Technologies, CA, USA).

### RNA-seq Library Preparation and Sequencing

For constructing the library of mRNA or circRNA, 5 μg of RNA for per sample was used as input. For mRNA library construction, Epicentre Ribo-Zero™ rRNA Removal Kit (Illumina, USA) was used to remove rRNA from the total RNA. For circRNA library construction, 3 U of RNase R (Epicentre, USA) per microgram was added to the rRNA removal system and incubated to remove linear RNA. Subsequently, the sequencing libraries were prepared in accordance with by using NEBNext® Ultra™ Directional RNA Library Prep Kit for Illumina® (NEB, USA). The Agilent Bioanalyzer 2100 system was used to detect the quality of the RNA library. After the library was qualified, the Illumina PE150 sequencing was performed. Considering that the Ribo-Zero library contains both mRNA and lncRNA, we will select lncRNA by establishing a series of strict screening conditions in the data analysis part. For miRNA library construction, 5 μg of total RNA for per sample was used as input using the NEBNext® Multiplex Small RNA Library Prep Set for Illumina® (NEB, USA) according to the manufacturer's protocol. Similarly, library quality was detected on the Agilent Bioanalyzer 2100 system. the Illumina PE150 sequencing was performed.

### Data Analysis

The libraries were sequenced using an Illumina sequencing platform. Then adapter sequences and reads with poor quality were filtered using FASTX toolkit pipeline (version 0.0.13). Clean reads were aligned to the UCSC (mus_musculus_Ensembl_97) using Hisat2 (http://ccb.jhu.edu/software/hisat2) 10. For transcriptome assembly, the mapped reads of each sample were assembled by StringTie (v1.3.1) 32, which has specific parameters for different libraries, and can accurately splice transcripts then achieve transcript quantification 33. Then, GC contents, Q20 and Q30 of the clean data were calculated.

We use soft of find_circ 34 and CIRI2 35 to detect and identify circRNA.The workflow of find_circ is as follows: 1) The reads that are continuously aligned with the genome are filtered out, and the spliced reads are retain; 2) the end of each candidate read are mapped to the genome to find unique anchor position; 3)candidate circRNAs are confirmed when the 3'end of the anchor sequence is aligned to the upstream of the 5'end of the anchor sequence, and the presumed breakpoint is flanked by GU/AG splicing signals. CIRI was first analyzed the CIGAR value in the sam file, scans the PCC signal (paired chiastic clipping signals) from the sam file, then filters the Junction reads based on the PEM and GT-AG signals, and finally detects unbalanced junction reads based on the DM algorithm. Filter and prevent false positives caused by homologous gene similarity and repeated sequences. We will choose two software for joint analysis to improve the accuracy of circRNA identification.

Small RNA tags were mapped onto the assembled genome of mus_musculus_Ensembl_97 using Bowtie 36, 37. The mapped small RNA tags were screened in the miRBase 20.0 database to search for known miRNA. In addition, modified software mirdeep2 and srna-tools-cli were used to obtain the potential miRNA and draw the secondary structures 38. The hairpin structure of miRNA precursors can be used to predict novel miRNA. We integrate miREvo 39 and mirdeep2 38 to predict the novel miRNAs. Meanwhile, custom scripts were used to obtain the identified miRNA counts and base bias on the first position with certain length and on each position of all identified miRNA, respectively 40. miRNA expression was estimated using TPM (transcript per million) Density distribution^41^. Differentially expressed miRNAs were identified using the threshold: P-value < 0.05.

### Differential expression gene analysis (DEG analysis) and Enrichment Analysis

The gene expression level of RNA-seq is usually measured with FPKM (Fragments Per Kilobase of transcript sequence per Millions base pairs sequenced) 42. We used a cutoff value of FPKM > 1 to define the gene expression. Differential expression gene analysis was performed using edgeR 43. We use p-value or corrected p value (padj) to determine the significance level. The significant difference standard is padj less than 0.05. Differentially expressed genes are screened and subjected to hierarchical cluster analysis. GO (Gene Ontology) is a comprehensive database describing gene functions, which can be divided into three parts: molecular function, biological process, and cellular component. GO analysis was performed by GOseq 44 (Bioconductor, USA). The p-value of GO term and the false discovery rate (FDR) of p-value (q-value) were calculated to find out the most relevant GO term for the genes with differential expression. GO enrichment takes padj less than 0.05 (default) or pvalue less than 0.05 as significant enrichment. Pathway enrichment analysis was performed using KEGG database 45 (Kanehisa Laboratories, Japan). Hypergeometric test was applied to identify the most significant enriched pathways among the candidate differently expressed genes. Similarly, for KEGG pathway enrichment, pvalue or padj is less than 0.05 as significant enrichment.

### Construction of the ceRNA regulatory network

The lncRNA-miRNA, miRNA-mRNA, lncRNA-miRNA-mRNA, circRNA-miRNA, miRNA-mRNA, and circRNA-miRNA-mRNA networks were developed based on possible functional relationships between DE-lncRNAs, DE-miRNAs, DE-circRNAs, and DE-mRNAs. The lncRNA-miRNA-mRNA ceRNA regulation network was based on the theory that lncRNAs can directly interact by invoking miRNA sponges to regulate mRNA activity 46. “GDCRNA Tools” (http://bioconductor.org/packages/devel/bioc/html/GDCRNATools.html) package in R software were used 47. We use Cytoscape 3.6.1 software to plot lncRNA-miRNA-mRNA network (Biological network exploration with Cytoscape 3). The candidate miRNA-circRNA regulation network was predicted using miRanda (MicroRNA targets in Drosophila). Similarly, the target mRNAs of DE-miRNAs were predicted by scanning for conserved miRNA target sites with MiRanda (MicroRNA targets in Drosophila), and a miRNA-mRNA regulation network was constructed. We use Cytoscape 3.6.1 software to combine circRNA-miRNA network and miRNA-mRNA network to generate circRNA-miRNA-mRNA network (Biological network exploration with Cytoscape 3).

### Validation of the Differential Expression of lncRNA and circRNA

To order to verify the results of transcriptome sequencing, we performed real-time PCR to determine the RNA levels of five lncRNAs and three circRNAs in chondrocyte and cartilage tissue. Quantitative real-time PCR amplification was performed by the ABI 7500 Real-Time PCR Systems (Applied Biosystems, USA). The first-strand cDNA was synthesized with PrimeScript™ RT Master Mix (Takara, Japan) and 2 μg total RNA. The amplification procedure was one cycle of 30s at 95 °C for pre-degeneration followed by 40 cycles of 30s at 95 °C and 34 s at 60 °C. The Ct values of the reference gene and the target genes were obtained according to the amplification curve. The relative quantification of gene expression was obtained by using the 2^-ΔΔCt^ method. GAPDH was used to normalize mRNA levels. The primer sequences used for real-time PCR are shown in Table 1.

## Results

### Identification of DE mRNAs between XBP1 CKO mice and control mice

**Figure 1:**
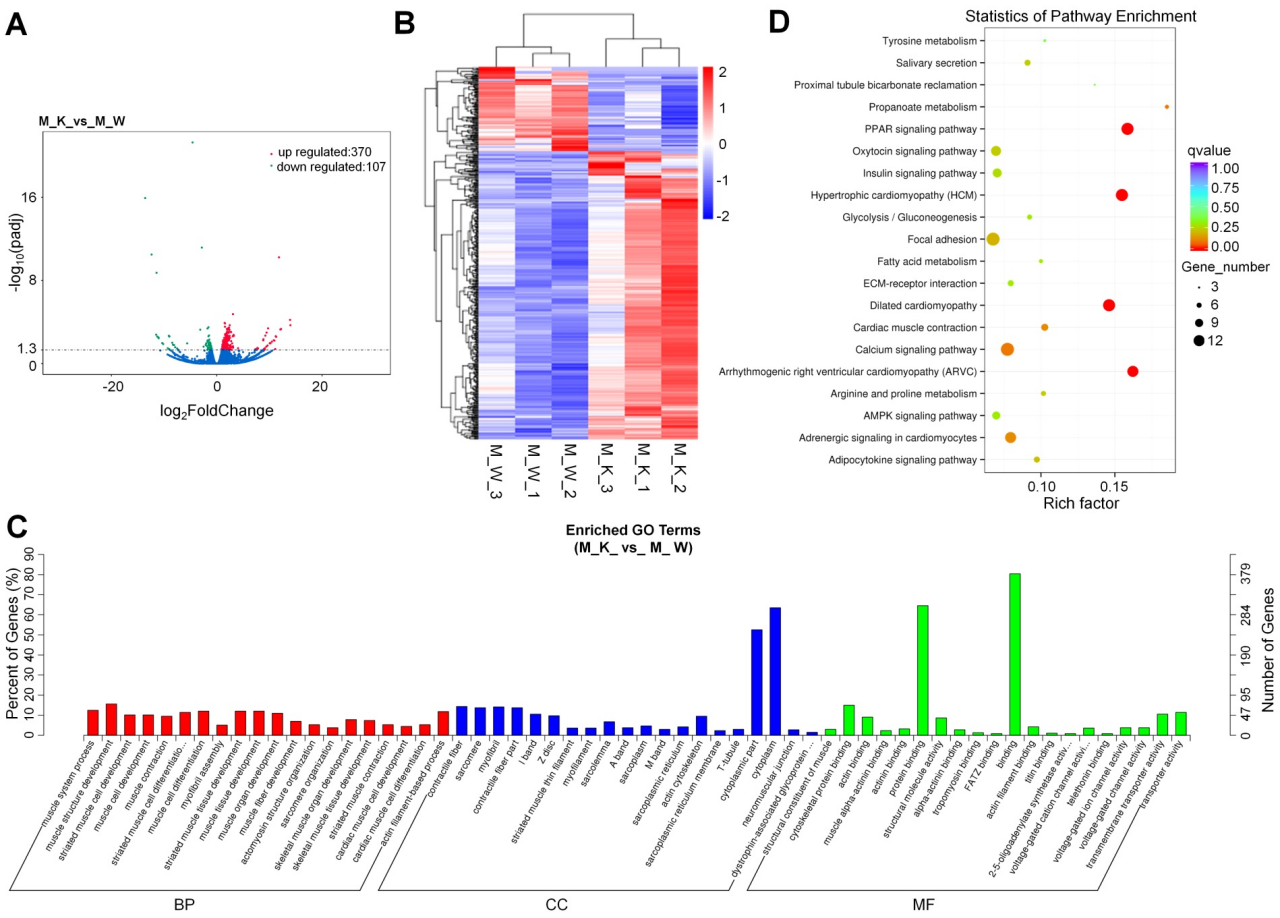
Overview of mRNA expression. (A) Volcano plots for mRNA. (B) Heatmap is used to assess the expression of mRNA. Red and blue denote high and low expression, respectively. Each DE-miRNA is represented by a single row of colored boxes, and each sample is represented by a single column. (C) Go term analysis of mRNA. (D) KEGG analysis of DE mRNA.

To identify the expression levels of mRNAs in XBP1 CKO mice and control mice, 6 mRNA libraries were constructed. Compared with the wildtype control, 477 genes, including 307 (74.2%) upregulated genes and 107 (25.8%) downregulated genes, were differentially expressed in XBP1 CKO mice, with a q value < 0.05 (Fig. 1A and Table S1). Based on hierarchical cluster analysis, some of the 477 DEGs may participate in the regulation of the same pathway or possess similar functions (Fig. 1B and Table S2). Among these genes, genes related to cartilage development (derived from the GO terms GO:1990079, GO:0061037, GO:0060351, GO:0003417, GO:0061035, and GO:0051216) were significantly enriched after XBP1 knockdown. There were 5 out of 138 such genes, compared with 477 out of 55573 genes (p=0.001, hypergeometric test). To further explore the functions of these DEGs, GO and KEGG functional analyses were performed. GO is an international standard classification system for gene function. GO can be divided into three parts: biological process (BP), cellular component (CC), and molecular function (MF). A total of 274 significantly enriched GO terms were identified (Fig. 1C and Table S3), with 74.5%, 15.3%, and 10.2% mRNAs assigned to BP, CC and MF, respectively. The MF category is associated with binding, protein binding and cytoskeletal protein binding; the BP category is associated with muscle structure development, muscle cell differentiation, muscle tissue development, and striated muscle cell differentiation; and the CC category is associated with contractile cytoplasm, cytoplasmic part, myofibril, sarcomere, contractile fiber and contractile fiber part. The DEGs were also aligned in the KEGG pathway database. As shown in Fig. 1D and Table S4, the pathways of PPAR signaling, hypertrophic cardiomyopathy (HCM), dilated cardiomyopathy (DCM), arrhymogenic right ventricular cardiomyopathy (ARVC) and calcium signaling were mainly activated.

### Prediction and function analysis of the lncRNA target genes

**Figure 2:**
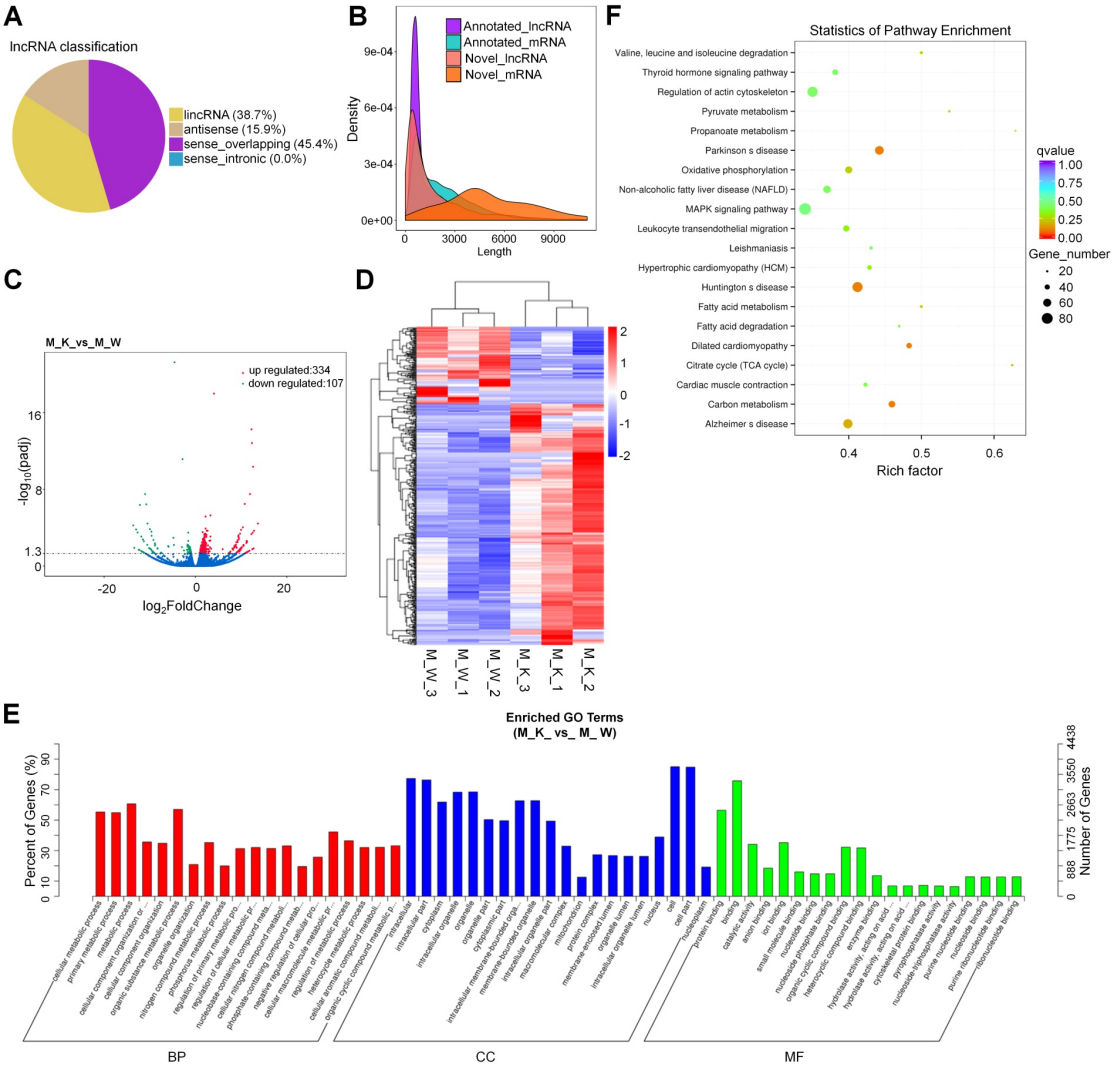
Prediction and function analysis of lncRNA target genes. (A) LncRNA type distribution. (B) Comparison of the length of lncRNA and mRNA density distribution. (C) Volcano plots for lncRNA. (D) Heatmap is used to assess the expression of lncRNA. (E) GO analysis of target genes of co-expression of differential lncRNA. (F) KEGG analysis of target genes of co-expression of differential lncRNA.

With reference to HUGO Gene Nomenclature Committee (HGNC), the newly screened lncRNAs were divided into four types according to their positional relationship with known mRNAs (Fig. 2A). To verify whether the new lncRNA meets the general characteristics, we compared the length of the transcript, the number of exons, and the length of the ORF between the lncRNA and the mRNA (Fig. 2B and Fig. S1A and S1B). Based on the figure, the new lncRNA conforms with the general characteristics of the transcript (transcripts with a transcript length longer than 200 nt and number of exons greater than or equal to 2 (Fig. 2B and Fig. S1A and S1B)). The DEGs in the lncRNA expression patterns between XBP1 knockout mice and controls were analyzed, including 334(75.7%) up-regulated genes and 107(24.3%) down-regulated genes (Fig. 2C and Table S5). To explore the expression patterns of these lncRNAs, a hierarchical cluster analysis of 441 differentially expressed lncRNAs was performed between XBP1 knockout mice and controls, which classified the expression patterns of the XBP1 knockout mice and wildtype mice into different clusters (Fig. 2D and Table S6). We carried out target gene prediction for all lncRNAs, (i.e., predict the target genes of lncRNA through the co-location and co-expression of lncRNA and protein-coding genes). Thereafter, function enrichment analysis (GO/KEGG) of the target genes of the differential lncRNA was carried out to predict the main function of lncRNA. The prediction results of co-expression and co-location are shown in Fig. 2E, Fig. S2 and Tables S7 and S8). The 841 core GO terms and 45(co-location) were extracted (Fig. 2E, Fig S2, Tables S7 and S8). In the BP category, GO terms, such as cellular metabolic process, primary metabolic process, metabolic process, organic substance metabolic process, cellular macromolecule metabolic and nitrogen compound metabolic process, were functionally enriched. The CC category was found to be associated with cell, cell part, intracellular part, intracellular, organelle, intracellular organelle and cytoplasm which the MF category was found to be associated with binding, protein binding, ion binding, catalytic activity, organic cyclic compound binding and heterocyclic compound binding. To further focus on the function of these lncRNAs, the KEGG pathways were analyzed. Based on the results, the differentially expressed lncRNA is involved in 4(co-expression) (Fig. 2F and Table S9) and 4(co-location) (Fig. S3 and Table S10). KEGG metabolic pathways, such as Parkinson's disease (PD), Huntington's disease (HD), carbon metabolism and dilated cardiomyopathy (DCM), were identified (Fig. 2F). The prediction result of co-location is shown in Fig. S4 and Table S10.

### Overview of the circRNA sequencing data

**Figure 3:**
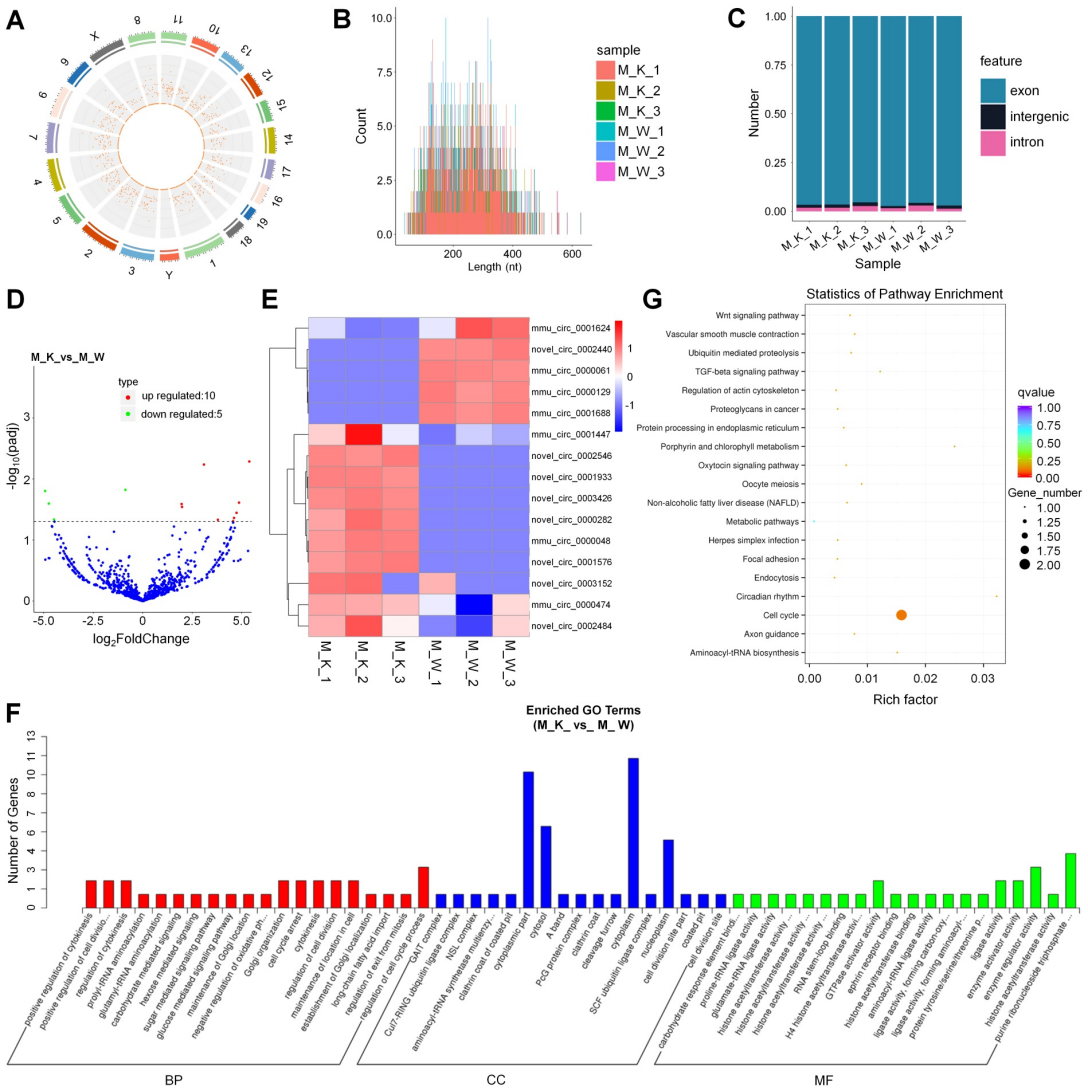
Identification and function analysis of circRNA. (A) Genome location of circRNAs from M_K_1 library. (B) The length distribution of circRNAs. (C) The source of circRNA. (D) Volcano plots for circRNA. (E) Heatmap is used to assess the expression of circRNA. (F) Go term analysis of circRNA**.** (G) KEGG analysis of DE circRNA.

To understand the roles of XBP1-associated circRNAs in cartilage development, we performed circRNA sequencing using the rRNA-depleted chondrocyte samples of wildtype mice (CON) and XBP1 CKO mice. Clean reads from the 6 libraries were used for circRNA identification. After a series of selection, 1651 novel circRNAs, which were widely distributed on 21 chromosomes were obtained and termed as mmu_circ_0000004 to novel_circ_0003429 (Fig. 3A, Fig. S4 and Table S11). A size distribution analysis revealed that the length of most circRNAs ranged from 200 to 400bp (Fig. 3B). By counting the sources of circRNA in all samples, we found that 95.08% of circRNAs were composed of exons, while 3.34% and 1.58% were located in the intronic and intergenic regions, respectively (Fig. 3C). To identify circRNAs that potentially participated in cartilage development, the differences in circRNA expression patterns between XBP1 CKO mice and wildtype mice were analyzed. As shown in Table S12, a total of 15 differentially expressed circRNAs were observed relative to the control, including 10 upregulated genes and 5 downregulated genes (Fig. 3D). To explore the expression patterns of these circRNAs, a hierarchical cluster analysis of 15 differentially expressed circRNAs was performed between the XBP1 CKO mice and wildtype mice, which classified the expression patterns of samples from XBP1 CKO mice and wildtype mice into different clusters (Fig. 3E). GO and KEGG functional analyses of these circRNAs were also conducted to explore the biological function of circRNAs. GO analysis, revealed that the regulation of cell cycle process, positive regulation of cytokinesis and cell division, regulation of cytokinesis, cell cycle arrest, cytokinesis and maintenance of location in cell were enriched in the BP category. The dominant functions in each of the remaining two categories included cytoplasm and cytoplasmic part in the CC category and purine ribonucleoside triphosphate and enzyme regulator activity in the MF category (Fig. 3F and Table S13). KEGG pathways analysis, which revealed the function of these circRNAs, showed that the pathways of cell cycle, and metabolic pathway were activated (Fig. 3G and Table S14).

### Overview of the miRNA sequencing data

**Figure 4:**
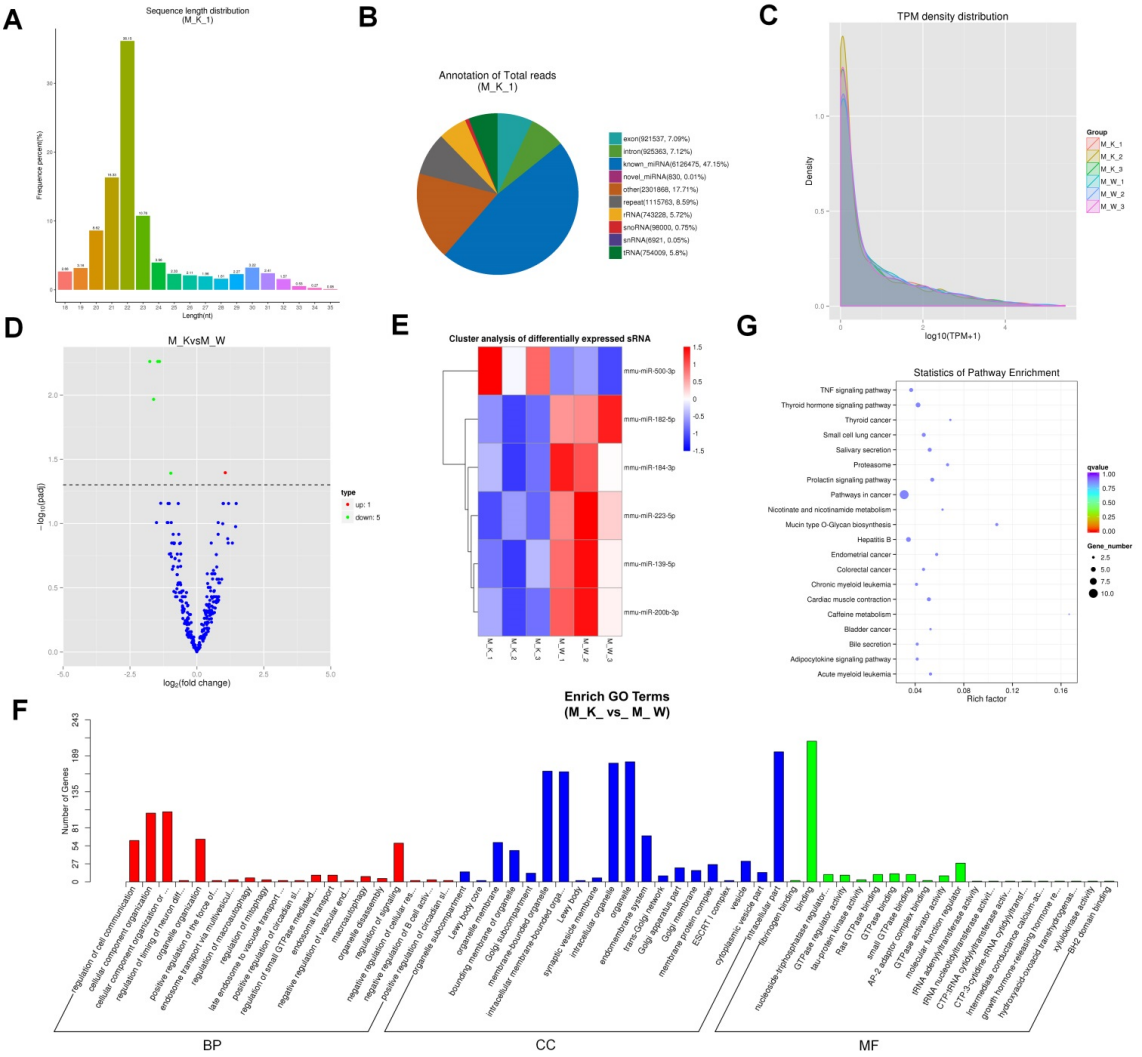
Analysis of miRNA sequencing data. (A) Size distribution of sRNAs from M_K_1 library. (B) Classification statistics of sRNA from M_K_1 library. (C) TPM (number of transcripts per million clean tags) density distribution of sRNAs from 6 samples. (D) Volcano plots for DE-miRNAs. (E) Heatmap is used to assess the expression of DE-miRNAs. (F) Go term analysis of DE miRNAs. (G) KEGG analysis of DE miRNAs.

We identified miRNAs in XBP1 CKO mice and explored their expression patterns. We found that 90.01% to 91.27% of small RNA reads were mapped onto the mouse (mus_musculus_Ensembl_97) genome (Table S15). The length distribution of sRNA was counted, and was found to range from 18 to 35 nt, with a peak of 22 nt, followed by 21 nt and 23 nt, respectively (Fig. 4A and Fig. S5). The statistics and annotations of all small RNAs and various types of RNAs are summarized in Fig. 4B and S6 Fig. We proceeded to predict the new miRNA. M_K_1 as an example, a total of 12,993,994 miRNAs were identified, including 6,126,475 known miRNAs and 830 novel miRNAs (Fig. 4B). The predicted new miRNA and the comparison of each sample sRNA are presented in Table S16. The expression levels of new and known miRNAs in each sample were determined, and the TPM (number of transcripts per million clean tags) density distribution was used to verify the gene expression pattern of the sample as a whole (Fig. 4C). The expression of miRNAs in different groups was compared, and the overall expression patterns of miRNAs in these groups were found to be highly consistent (Fig. 4C). Compared with the miRNA expression levels of the control group, 6 miRNAs showed significantly differential expression (p < 0.05), including 1 significantly up-regulated miRNA and 5 significantly down-regulated miRNA (Fig. 4D). To explore the expression patterns of these miRNAs, a hierarchical clustering analysis of 6 differentially expressed miRNAs was performed. Based on the results of this analysis, the cartilage samples of XBP1 CKO mice and control mice were divided into different clusters (Fig. 4E). After further evaluation, we found that these miRNAs target 43,617 transcripts. According to the correspondence between miRNAs and their targets, we performed GO and KEGG enrichment analysis on each set of differentially expressed miRNA target genes. GO analysis showed that these target genes were mainly enriched in 468 GO term processes (p < 0.05) (Fig. 4F). Analysis of the KEGG pathways revealed that these miRNA target genes were involved in the cancer pathway, Hepatitis B and caffeine metabolism, thyroid hormone signaling pathway, and mucin type O-glycan biosynthesis (Fig. 4G).

### Construction of the potential circRNA-miRNA and lncRNA-miRNA network

Competitive endogenous RNA (ceRNA) analysis is directly based on the ceRNA hypothesis, centered on miRNA, and analyzed based on the correlation between the expression levels of different molecules in multiple samples. Further, this assessment does not rely on difference analysis 48. To clarify the functional relationship between lncRNA and its target miRNA, and circRNA and its target miRNA, we conducted a comprehensive analysis of the interaction between lncRNA and its target miRNA, and circRNA and its target miRNA. According to the expression values of miRNA and circRNA, 5,882 miRNA-circRNA interactions were identified (absolute correlation coefficient > 0.7 and p-value <0.05), with 974 miRNAs and 1,627 circRNAs, respectively (Table S17). Among these interactions, 5,055 were determined to be positively correlated, while the remaining 827 were negative (Table S17). Similarly, 272,329 miRNA-lncRNA interactions were identified (absolute correlation coefficient > 0.7 and p-value <0.05), including 1,076 miRNA and 37,320 lncRNAs, respectively (Table S18). Among these interactions, 178473 were determined to be positively correlated, while the remaining 93,856 were negative (Table S18). Further, we found that 14 circRNAs (mmu_circ_0001376, mmu_circ_0001632, mmu_circ_0000713, mmu_circ_0000714, novel_circ_0002622, novel_circ_0002301, novel_circ_0002887, novel_circ_0002720, novel_circ_0000181, novel_circ_0000182, novel_circ_0000199, novel_circ_0000280, novel_circ_0000108 and novel_circ_0000140) had binding sites for mmu-miR-99b-5p, which targets ENSMUSG00000031849 (log2 fold change=4.59) (Table S19). LncRNAs (ENSMUST00000206990 (log2 fold change=0.98)) had binding sites for mmu-miR-23a-5p, which targets ENSMUSG00000000126 (log2 fold change=3.19) (Table S20). In summary, a deficiency in XBP1 in chondrocyte leads to changes in the expression of some miRNAs, lncRNAs and circRNAs, which are positively or negatively correlated with target marker genes that regulate cartilage growth. XBP1 may thus participate in the process of bone and cartilage growth through the network of miRNAs-lncRNA or miRNAs-circRNAs, which play an important role in cartilage growth and bone associated diseases.

### Construction of the potential miRNA-mRNA network

We used the miRNA software to construct a regulatory network of miRNA and its corresponding target mRNA. Collectively, we identified 14141 miRNA-mRNA interactions, involving 907 and 8758 miRNA and mRNAs, respectively (Table S21). Most of these miRNAs were found to target multiple mRNAs. For example, mmu-miR-709, mmu-miR-149-3p, mmu-miR-1249-5p, mmu-miR-7648-3p and mmu-miR-328-5p were found to have 177, 195, 166, 174, and 68 target mRNAs, respectively. Further, 136 types (14.99%) of miRNAs were found to only target one mRNA. Many mRNAs were related to more than one miRNA. For example, ENSMUST00000108883 was targeted by 2 miRNAs, including mmu-miR-3154 and mmu-miR-370-5p. Such findings indicate a complex miRNA-mRNA regulatory network in the process of cartilage development, the involvement of some miRNAs in cartilage development through the directly targeting of the mRNA of some cartilage growth related marker genes. Other miRNAs were found to participate in cartilage development through the network of miRNA-lncRNA or miRNA-circRNA.

### Comprehensive analysis of the circRNA--miRNA-mRNA and lncRNA-miRNA-mRNA networks

**Figure 5:**
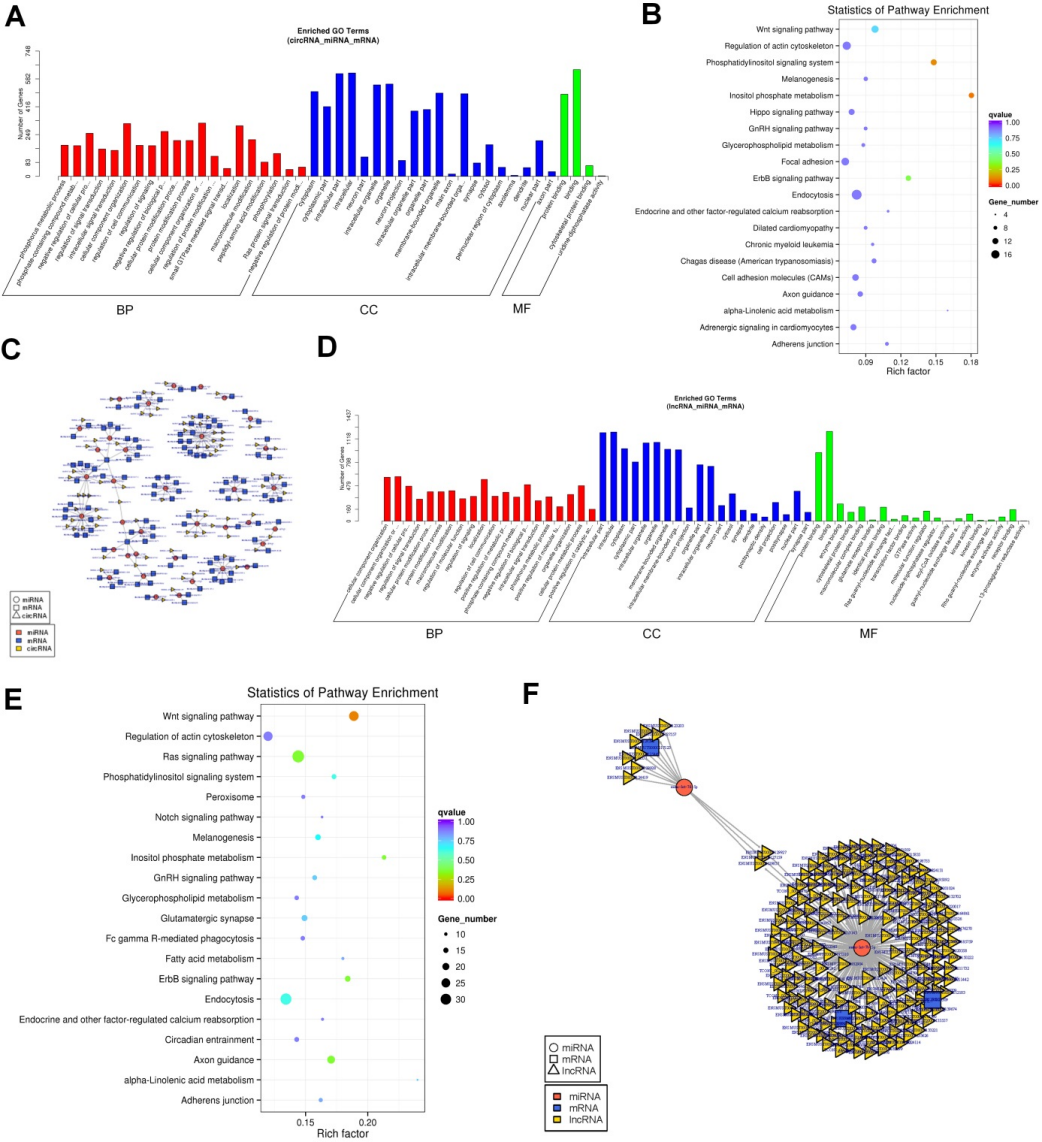
Established circRNA-miRNA-mRNA and lncRNA-miRNA-mRNA network.(A)GO analysis of the DEGs in the circRNA-miRNA-mRNA network. (B)KEGG analysis of the DEGs in the circRNA-miRNA-mRNA network. (C)The circRNA-miRNA-mRNA Competing endogenous RNA network. The squares indicate mRNAs in blue, triangles represent lncRNAs in light yellow and round shapes represent miRNAs in orange. (D)GO analysis of the DEGs in the lncRNA-miRNA-mRNA network. (E) KEGG analysis of the DEGs in the lncRNA-miRNA-mRNA network. (F)The lncRNA-miRNA-mRNA Competing endogenous RNA network. The squares indicate mRNAs in blue, triangles represent lncRNAs in light yellow and round shapes represent miRNAs in orange.

To further explore the potential networks among circRNA, miRNA, and mRNA, we combined the circRNA-miRNA interaction and miRNA-mRNA interaction to construct 1,822 circRNA-miRNA-mRNA interactions (satisfying |R|>0.7 and p< 0.05 for both circ-mRNA and miRNA-mRNA interactions) (Fig. 5C), that involve 203 circRNAs, 186 miRNAs, and 893 mRNAs (Table S22). The circRNA-miRNA-mRNA networks suggested that 11 upregulated circRNAs were bound to 7 miRNAs and 34 miRNA-targeted mRNAs. Three down-regulated circRNAs (novel_circ_0001966, novel_circ_0002499, and mmu_circ_0000906) harbored the miRNA mmu-miR-3102-3p which targeted the *BIG-2A* gene and may be related to the down regulation of that gene. Meanwhile, the upregulated circRNAs mmu_circRNA, mmu_circ_0000905, and mmu_circ_0000950, harboring Anx6 target the miRNA mmu-miR-3153 and mmu-miR-370-5p, which may be related to its up regulation.

Based on our prior results, 204 differentially expressed lncRNAs, 530 differentially expressed mRNAs, and 1076 differential expressed miRNAs that might play a role in cartilage development were revealed. We constructed 253235 lncRNA-miRNA-mRNA co-expression networks based on the lncRNA-miRNA and miRNA-mRNA results (satisfying |R|>0.7 and p< 0.05 for both circ-mRNA and miRNA-mRNA interactions) (Fig. 5F and Table S23). A remarkable number of lncRNA was determined to be candidate inhibitors for the repressive effect of miRNAs to their targets. For example, there were 311 lncRNAs including highly upregulated HO2, harboring the targeting miRNA of the Vgrl gene and may be related to its induction of expression after XBP1 knockout. Similarly, another 31 downregulated lncRNAs harboring the targeting miRNAs of the Prhr1 and may be related to its increased expression level.

The networks between circRNA, lncRNA, miRNA and mRNA are complicated. In fact, the same miRNA can target different cirRNAs, lncRNAs and mRNAs, and different miRNAs can target different cirRNAs, lncRNAs and same mRNAs, Different circRNA-lncRNA -miRNA-mRNA network perform different biological functions according to the non-coding RNAs involved, which can be used as candidates for subsequent functional analysis.

### GO and KEGG analysis of lncRNA and circRNA regulatory networks

We performed GO and KEGG analyses to evaluate the function of DEGs in the network. In the circRNA regulatory networks, GO analysis revealed that there were 119, 24, and 4 enriched GO terms with statistical significance (p < 0.05) in the BP, CC, and MF, respectively (Fig. 5A and Table S24). For BPs, the DEGs were enriched in cellular component organization, negative regulation of biological process, location and macromolecule modification. For CC, the top enriched items were intracellular, intracellular organelle, cytoplasm and membrane-bounded organelle.

Finally, for MF, binding and protein binding were enriched. KEGG pathway enrichment analysis was also conducted to characterize the target genes. Accordingly, the top significantly enriched pathways were identified to be related to inositol phosphate metabolism and phosphatidylinositol signaling system (Fig. 5B and Table S25). In the lncRNA co-expression networks, GO analysis revealed 298, 67, and 24 enriched GO terms with statistical significance (p < 0.05) in the BP, CC, and MF, respectively (Fig. 5D and Table S26). The MF category had enriched terms in protein binding and binding. The BP category had enriched terms in intracellular component organization, localization, negative regulation of biological process and cellular protein metabolic process. The CC category had enriched terms in intracellular, intracellular organelle, membrane-bounded organelle and organelle part. KEGG pathway enrichment analysis indicated that the top significantly enriched pathway was Wnt signaling pathway (Fig. 5E and Table S27).

### Validation of the differentially expressed ncRNAs in mice

**Figure 6:**
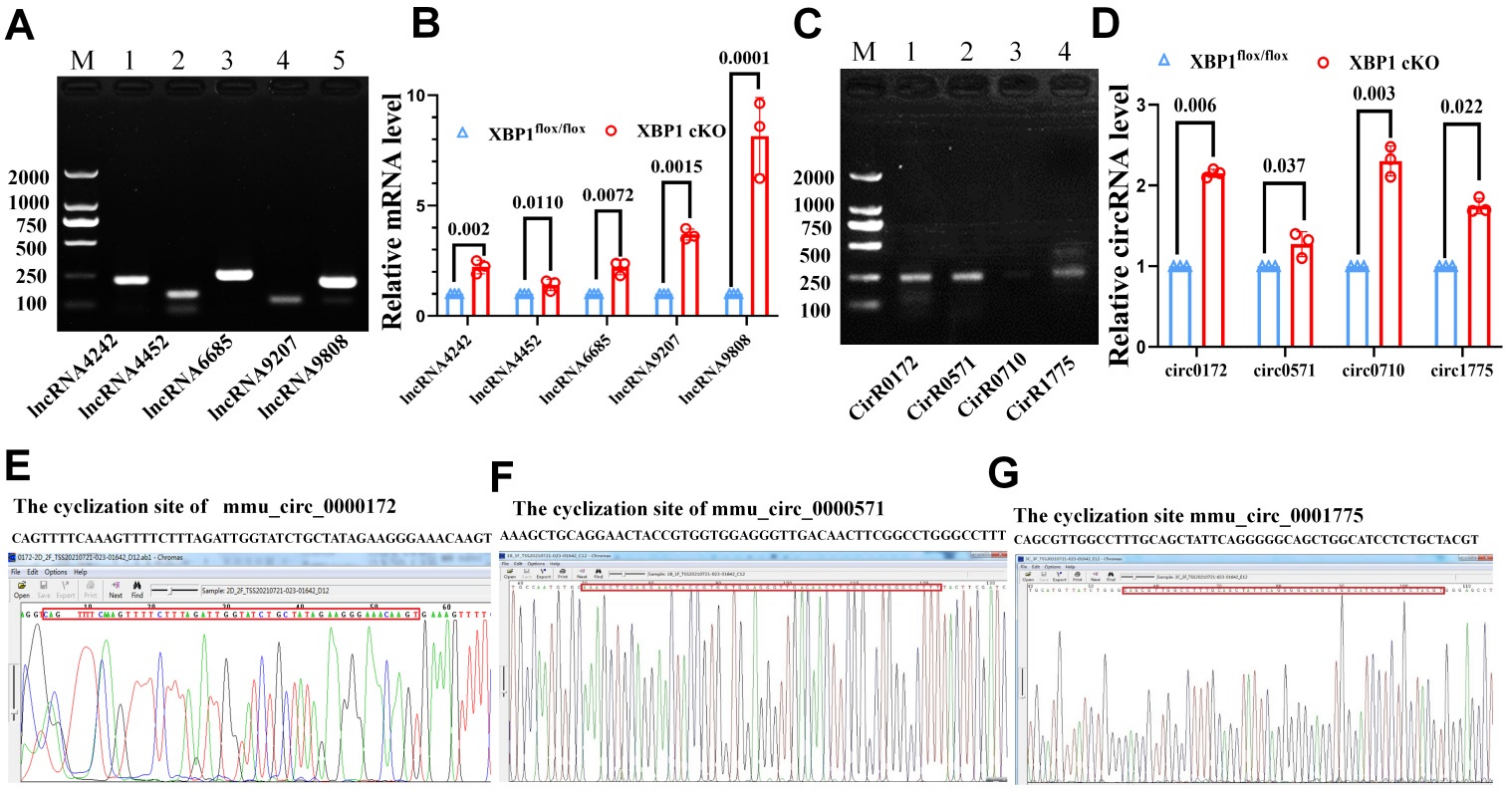
Confirmation the differential expression of ncRNAs in XBP1CKO and control mice. (A) The PCR detection of five lncRNAs. M. Marker, 1. ENSMUST00000124242, 2. ENSMUST00000224452, 3. ENSMUST00000126685, 4. ENSMUST00000169207, 5. ENSMUST00000219808. (B) mRNA level of five lncRNAs in the cartilage of XBP1 CKO mice and control mice. (C) The PCR detection of four circRNAs. M. Marker, 1. mmu_circ_0000172, 2.mmu_circ_0000571, 3. novel_circ_0000710, 4.mmu_circ_0001775. (D) mRNA level of four circRNAs in the cartilage of XBP1 CKO mice and control mice by QPCR. CircRNA sequencing of mmu_circ_0000172. (E) mmu_circ_0000571. (F) mmu_circ_0001775 (G).

To confirm the expression levels of RNA obtained by Illumina sequencing, we first selected five lncRNAs, including ENSMUST00000124242 (log2 fold change=3.06), ENSMUST00000224452 (log2 fold change=2.18), ENSMUST00000126685 (log2 fold change=4.98), ENSMUST00000169207 (log2 fold change=2.13), and ENSMUST00000219808 (log2 fold change=5.78), and measured their expression levels using RT-PCR (Fig. 6A). Thereafter, we isolated the cartilage tissue from XBP1 CKO (XBP1flox/flox col2CRE+) and control (XBP1flox/flox col2CRE-) mice, and detected the mRNA level of five lncRNAs in different cartilage tissues. QPCR revealed that the expression of five lncRNAs were upregulated in the cartilage of XBP1 CKO mice compared with that of controls (Fig. 6B).

We selected four circ-RNAs, including mmu_circ_0000172 (log2 fold change=2.40), mmu_circ_0001775 (log2 fold change=1.72), novel_circ_0000710 (log2 fold change=0.64), and mmu_circ_0000571 (log2 fold change=0.70), and detected their expression as well as their cyclization sites. The results of RT-PCR, qPCR and circRNA sequencing were consistent with those of Illumina sequencing. As depicted in Fig. 6 C, D, E, F, G, the cyclization sites were determined by circRNA sequencing, and the expression of three circRNAs was found to be upregulated in the cartilage of XBP1 CKO mice compared with that of controls. The primers sequences are listed in Table 1. All of the randomly selected lncRNA and circRNA showed a similar expression pattern between qPCR and Illumina sequencing. Further, the both RT-PCR, qPCR and circRNA sequencing results confirmed the accuracy and reliability of the sequencing data.

## Discussion

Abnormal differentiation of chondrocytes will lead to several cartilage or bone developmental diseases, which are closely related to the molecular mechanism of their differentiation and development 49. Previous studies have shown that the IRE1/XBP1s signaling pathway is involved in cartilage formation and bone growth 50; however, the regulation mechanism of XBP1 and its associated non-coding RNA involved in cartilage development remain unclear. In the present study, we established XBP1^-/-^ cartilage-specific knockout mice (XBP1 CKO) and conducted whole transcriptome sequencing using cartilage tissue obtained from XBP1 CKO mice and controls. Whole transcriptome sequencing can reveal the target regulate relationship between various non-coding RNAs and mRNAs and the interaction network between ncRNAs. First, we analyzed differentially expressed circRNAs, lncRNAs, miRNAs, and mRNAs, which revealed 15 DE circRNAs, 441 DE lncRNAs, 6 miRNAs and 477 mRNAs. Among them, genes related to cartilage development (derived from the GO terms GO: 1990079, GO: 0061037, GO: 0060351, GO: 0003417, GO: 0061035, and GO: 0051216) were significantly enriched after XBP1 knockdown. There were 5 out of 138 such genes, compared with 477 out of 55573 genes (p=0.001, hypergeometric test). The biological functions of the above RNAs were analyzed by GO and KEGG enrichment analysis.

With the development of molecular biotechnology, non-coding RNA, a type of RNA that cannot encode proteins, was considered to be noise in the genome in the past, and it plays a vital role in various biological processes 51. Some studies found that the expression disorder of lncRNA or circRNA was related to the occurrence of bone development disease, such as lncRNA‐OG 23 and circRNA_0001052/miR-124-3p 27. However, whether and how XBP1 splicing regulates the formation and function of ncRNAs remain unknown. Further, whether and how XBP1 modulates cartilage development through these ncRNAs-mediated regulatory networks need to be clarified. Herein, some lncRNA-miRNA-mRNA and circRNA-miRNA-mRNA networks related to XBP1 deficiency were constructed. These networks indicated that circRNA and lncRNA may play a nodal regulatory role. A single circRNA and lncRNA can be associated with multiple mRNAs and multiple identical miRNAs. For example, 14 circRNAs (mmu_circ_0001376, mmu_circ_0001632, mmu_circ_0000713, mmu_circ_0000714, novel_circ_0002622, novel_circ_0002301, novel_circ_0002887, novel_circ_0002720, novel_circ_0000181, novel_circ_0000182, novel_circ_0000199, novel_circ_0000280, novel_circ_0000108 and novel_circ_0000140) were found to have binding sites for mmu-miR-99b-5p, which targets ENSMUSG00000031849 (log2 fold change=4.59) (Table S19). Hsa circ_0000729 was reported as a potential prognostic biomarker in lung adenocarcinoma, and hsa-miR-3154 serves as a potential prognostic biomarker for cervical cancer^52^, ^53^. MicroRNA is a type of small RNA molecule, that is usually combined with a short complementary sequence located in the 3'_UTR region of mRNA to regulate the expression of target mRNA. MiRNA can directly regulate gene expression by interacting with not only lncRNA, but also circRNA and mRNA. Furthermore, both lncRNAs and circRNAs can regulate microRNAs through the “sponge” effect. LncRNAs, miRNAs, and circRNAs can also modulate gene expression by binding to the mRNA or mRNA splicing 12, 54-56.

LncRNAs are a highly heterogeneous class of RNAs that can be transcribed from any part of protein-coding genes and intergenic regions in sense or antisense orientation. The functions of lncRNA in eukaryotic cells are very diverse, including roles in high-order chromosome dynamics, telomere biology, and subcellular structure organization 10, 11, 57. We found that lncRNAs (ENSMUST00000206990 (log2 fold change=0.98)) possessed binding sites for mmu-miR-23a-5p, which targets ENSMUSG00000000126 (log2 fold change=3.19) (Table S20). MiRNAs are thought to modulate the translation process of more than 60% of protein-coding genes in eukaryotic cells, which are involved in the regulation of many biological processes, including proliferation, apoptosis, differentiation, and development. Different miRNAs have different regulatory processes; some miRNAs control specific individual targets, whereas others serve as the main regulators of one process. Therefore, pivotal miRNAs can simultaneously regulate the expression levels of multiple genes, and different types of miRNAs coordinate their targets 10, 11, 57. In XBP1 KO chondrocytes, mmu-miR-709, mmu-miR-149-3p, mmu-miR-1249-5p, mmu-miR-7648-3p and mmu-miR-328-5p had 177, 195, 166, 174 and 68 target mRNAs, respectively. In summary, these RNAs play an important role in cartilage development by modulating XBP1 expression. In addition, the biological function of target genes was identified based on GO annotation and KEGG pathway enrichment analyses.

**Figure 7:**
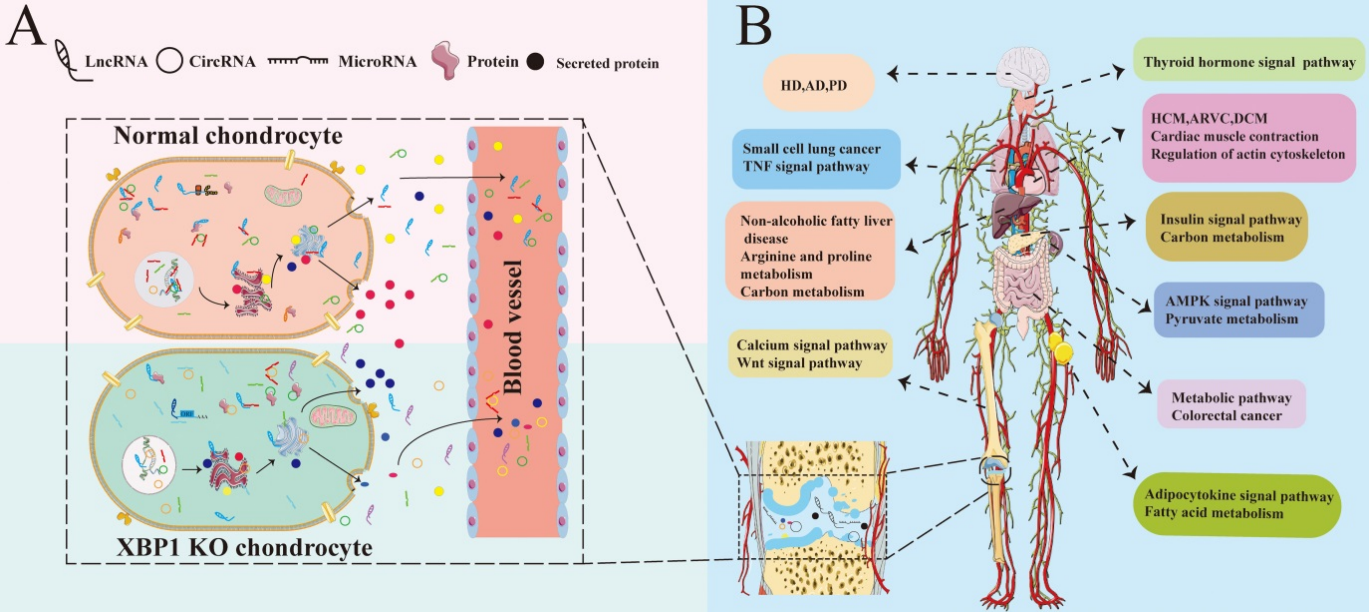
The model of XBP1 deficiency in cartilage is associated with various tissue diseases. (A)XBP1 deficiency in cartilage leads to differences in various of secreted factors and complete transcriptome RNA of Chondrocytes, including all kinds of ncRNAs with differential expression and regulatory profiles 63. (B) The coordinated regulation between different tissues and organs is performed by various of secreted factors and full spectrum of transcriptome RNA, including mRNA and ncRNAs. The differential ncRNAs and secreted factors caused by XBP1 deficiency in chondrocyte may cause abnormalities in different signaling pathways of different organs and tissues through blood and body fluid circulation 64, 65, including Wnt, insulin, AMPK, and adipocytokine signal pathway. Then these differential signal pathways lead to abnormalities in the functions of other different tissues and organs, including the nervous system, heart, bones, thyroid, and intestines. Such abnormalities, in turn, result in many kinds of different diseases. The black marks in the joint cavity represent various different ncRNAs and secreted proteins from chondrocytes.

All is known that the coordinated regulation between different tissues and organs is performed by blood and body fluid circulation. The chondrocyte secretome is made up of many proteins participated in cellular regulatory pathways, cell-cell or cell-ECM interactions, including chaperons, apolipoproteins and chondrocalcin, etc. Chondrocyte metabolism is affected by its micro-environment, including various different secreted factors and ncRNAs. Furthermore, the chondrocyte micro-environment is also influenced by many kinds of secret proteins released from osteoblasts, osteocytes, osteoclasts 58-60. XBP1 deficiency in chondrocyte causes differences in various of secreted factors and full transcriptome RNA, including all kinds of ncRNAs with differential expression and regulatory profiles as well as differences in protein expression and abnormalities of various different secreted factors. We analyzed the full profile of transcriptome RNAs, and found that XBP1 deficiency cartilage not only affects cartilage and related diseases influenced by the transcriptome, but also the functions of other tissues influenced by the transcriptome. Compared with those of normal mice, the expression and regulation profiles of not only multiple genes associated with many aspects of cartilage development and other cartilage-related diseases but also corresponding genes in other tissues and organs were altered in XBP1 CKO mice. Such changes result in different diseases in different tissues. As summarized in Fig. 7, differential ncRNAs and secreted factors caused by the lack of XBP1 in chondrocytes may cause abnormal signaling pathways in different organs and tissues through blood and body fluid circulation 60-62. These differential signal pathways cause abnormalities in the functions of other tissues and organs, including the nervous system, heart, bone, thyroid, and intestine. Such abnormalities result in many types of diseases, including HCM, DCM, ARVC, PD, HD, and thyroid associated disease.

In summary, XBP1s is produced under UPR in ER stress. We analyzed the expression of ncRNAs and the network between circRNA, lncRNA, miRNA, and mRNA under XBP1 deficiency in cartilage. NcRNA plays a crucial role in the occurrence and development of diseases. To date, most of the functions of lncRNA and circRNA have not been well understood. The present study revealed that there is a variety of ncRNAs in XBP1 knockout mice, including lncRNA, circRNA, miRNA and mRNA, that are significantly differentially expressed compared with those in wild type controls. Although the results of this study need further experimental validation, the profile of these differentially expressed circRNA and lncRNA may help elucidate the occurrence and development of bone development-associated diseases and help identify prospective clinical markers.

## Supplementary Material

Supplementary figures and tables.

## Figures and Tables

**Table 1:** The lncRNA and circRNA number and their amplification primer.

order name	Sequence	Length (nt)	Target- ncRNAs
qmlncR4242F	TTCTCGGTGTCCAGGTTGC	19	>ENSMUST00000124242
qmlncR4242R	GCGGTAGTGAGTCGGTTTG	19	
qmlncR4452F	TGCGTCCCCACTGACTAGC	19	>ENSMUST00000224452
qmlncR4452R	GTTGGCATCTGGACCATCTTC	21	
qmlncR6685F	AATAAGGCAGCGGAAAGAAG	20	>ENSMUST00000126685
qmlncR6685R	AAGGCTCCCAATGACACG	18	
qmlncR9207F	ACAGTGAGTCCTCCTTACAAAC	22	>ENSMUST00000169207
qmlncR9207R	ATGGCCTGGAACTTCTGTG	19	
qmlncR9808F	CACCCTCAATACCCTCACCTC	21	> ENSMUST00000219808
qmlncR9808R	AGCGGCAGTCTGTCTCATAC	20	
qmcirR0172F	AGTCCGTCGTGTCGTTCCA	19	>mmu_circ_0000172
qmcirR0172R	CTGCTGGGTATAAATGGTAGGAG	23	
qmcirR0571F	CCATCACGGGAGATTTTGC	19	>mmu_circ_0000571
qmcirR0571R	CACCAGAAAGGAACTGTCGC	20	
qmcirR1775F	CAACCCCTCTGACCTCTACG	20	>mmu_circ_0001775
qmcirR1775R	TCAGGCCAATGATGAAAAGC	20	
qmcirR0710F	ACTGGCAGCACCACTAACAC	20	>mmu_circ_0000710
qmcirR0710R	CGAACTGCTCACTAAGGACG	20	

## Abbreviations

XBP1: X-box binding protein 1
UPR: unfolded protein response
ER stress: endoplasmic reticulum stress
XBP1 CKO: XBP1conditional knockout
DE: Differentially expressed
DEGs: differential expression genes
GO: gene ontology
KEGG: Kyoto Encyclopedia of Genes and Genomes
BP: biological process
CC: cellular component
MF: molecular function
HUGO: Human Genome Organization
HCM: hypertrophic cardiomyopathy
DCM: dilated cardiomyopathy
ARVC: arrhymogenic right ventricular cardiomyopathy
PD: Parkinson's disease (PD)
HD: Huntington's disease
