# Supplementary material for: Effect Of XBP1 Deficiency In Cartilage On The Regulatory Network Of LncRNA/circRNA-miRNA-mRNA
Source: Int J Biol Sci. 2022 Jan 1;18(1):315–30. doi: 10.7150/ijbs.64054 (PMC8692151; doi:10.7150/ijbs.64054)
Supplement: Supplementary file 1 — Supplementary figures and tables. [file ijbsv18p0315s1.pdf]

Supplementary Figure

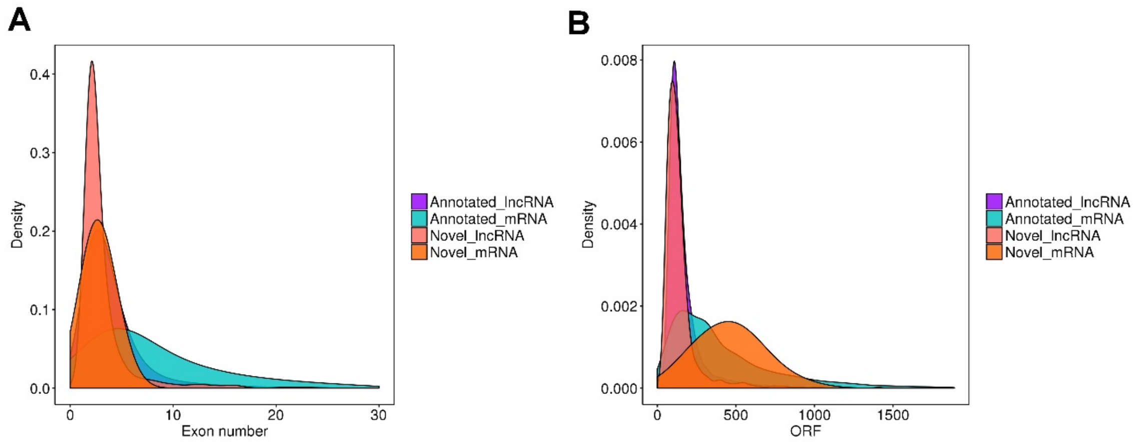

Figure S1. Comparison of lncRNA and mRNA characteristics. **(A)**The exon number density distribution of lncRNA and mRNA.(B)ORF length density distribution of lncRNA and mRNA.

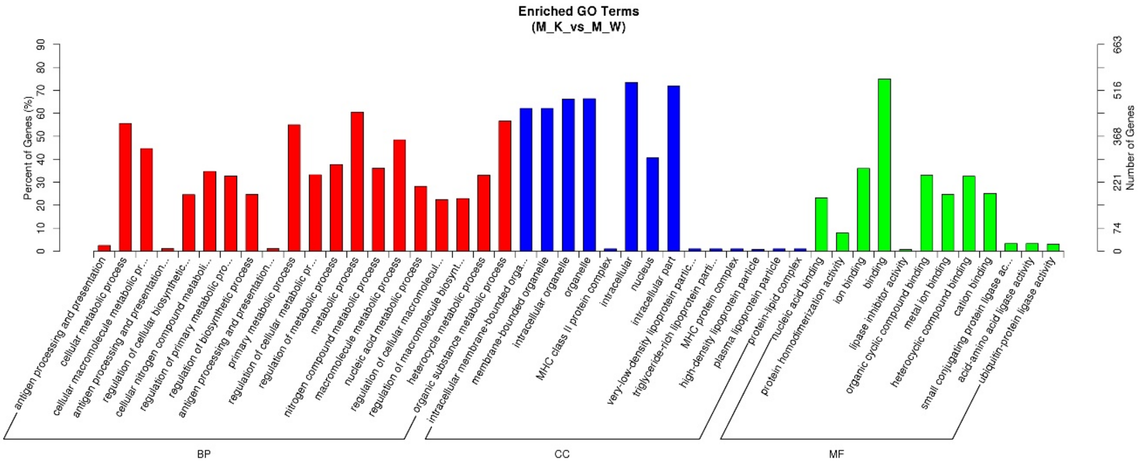

Figure S2. GO analysis of target genes of co-location of differential lncRNA.

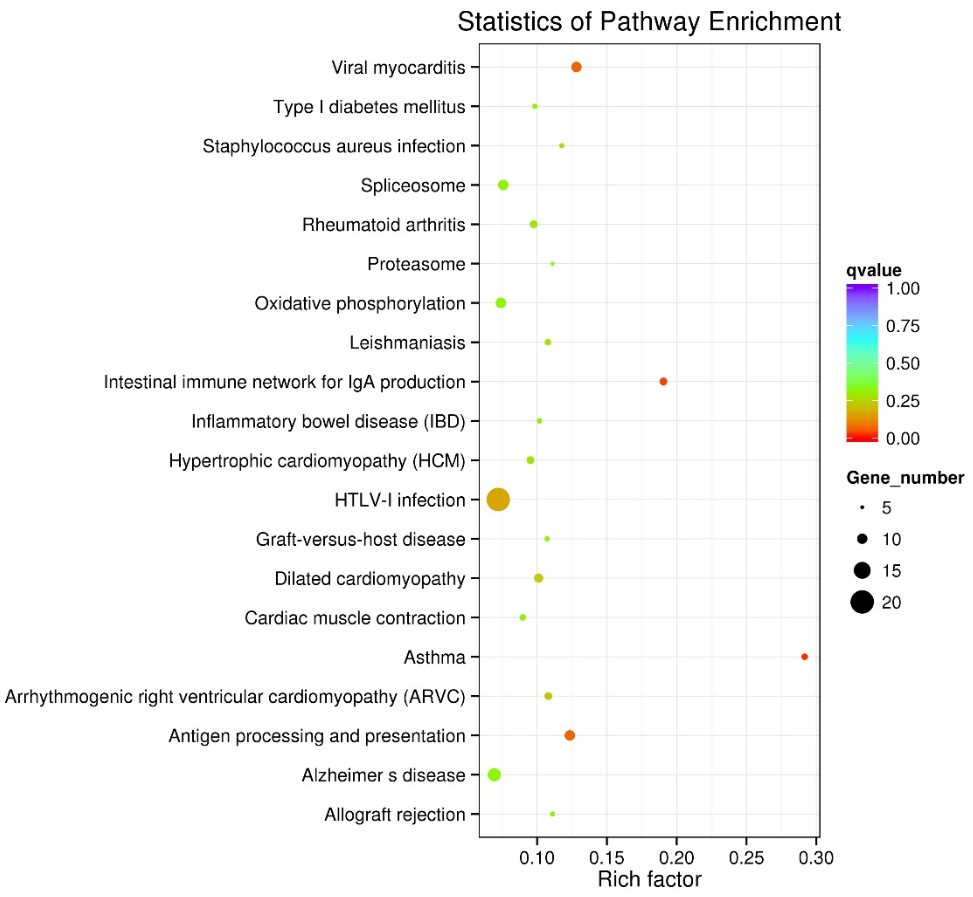

Figure S3. KEGG analysis of target genes of co-location of differential lncRNA.

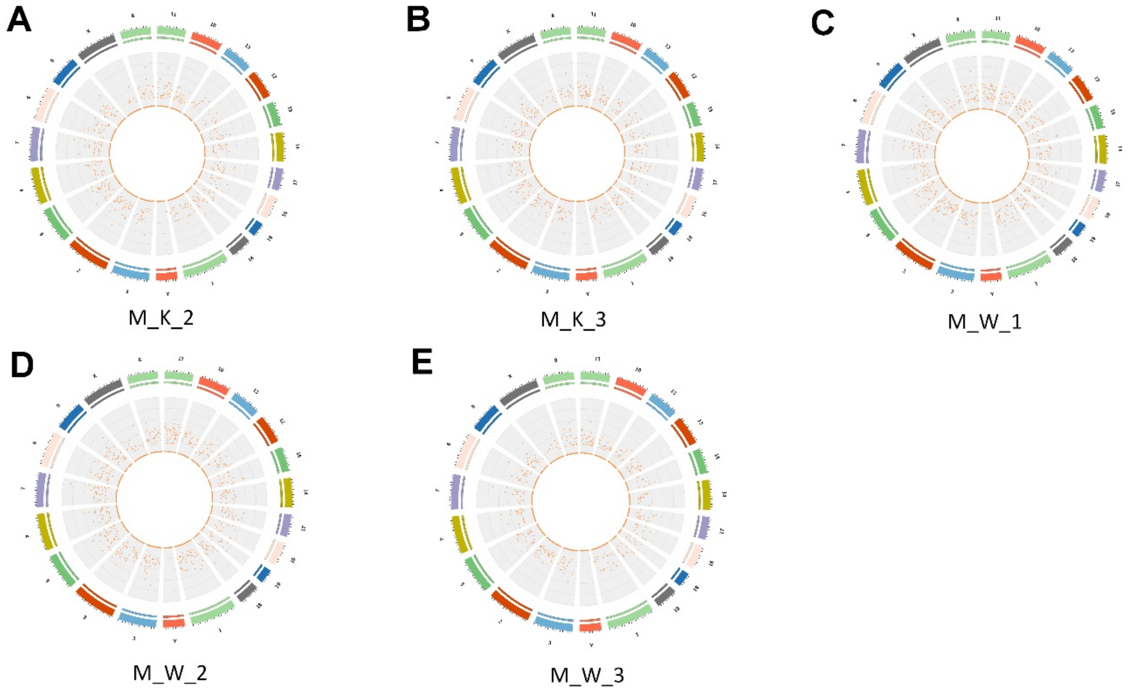

Figure S4. Genome location of circRNAs from 5 libraries.

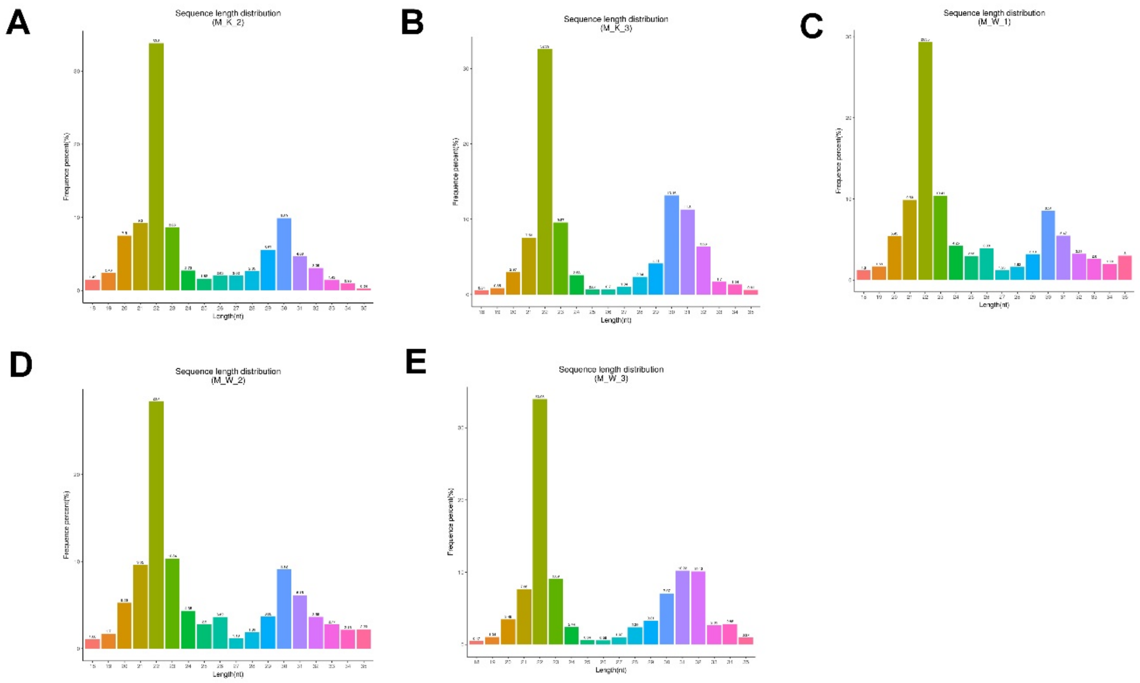

Figure S5. Size distribution of sRNAs from 5 libraries.

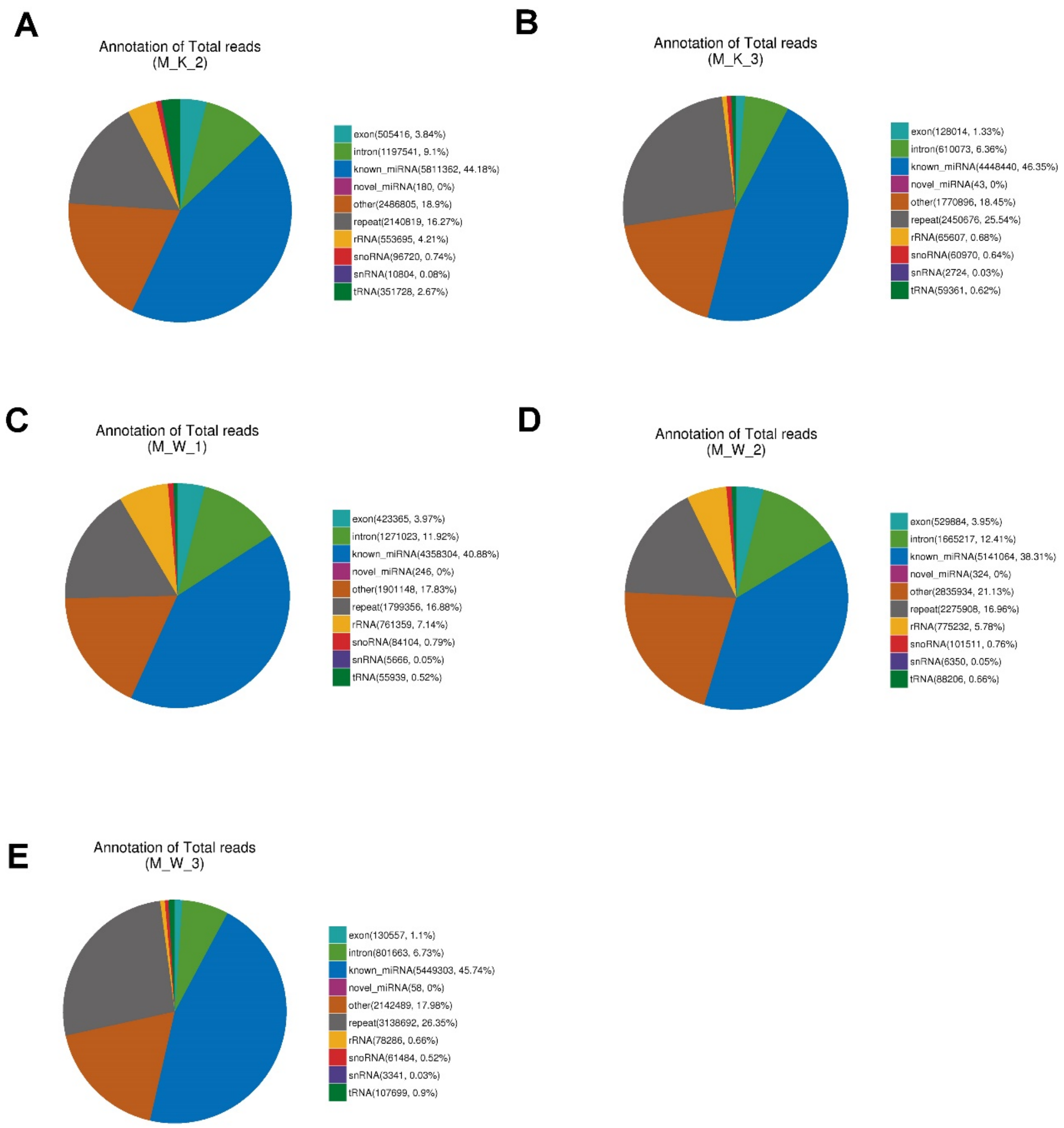

Figure S6. Classification statistics of sRNA from 5 libraries.

Table S1_M_K_vs_M_W.mRNA.different gene

gene_id	gene_name	gene_locus	M_K_FPKM	M_W_FPKM	log2FoldChange	pvalue	padj
ENSMUSG00000031849	Comp	8:70373558-70382066	3.441248	85.927	-4.585136053	3.05E-26	5.41E-22
ENSMUSG00000031620	Iqcm	8:75448694-75984503	0	0.057975	-13.58937362	1.70E-20	1.21E-16
ENSMUSG00000051510	Mafg	11:120625117-120633600	13.7015	29.56415	-2.829998116	1.57E-15	6.99E-12
ENSMUSG00000117098	Gm49909	17:70974119-71002208	0	4.408517	-12.37335359	8.29E-15	3.27E-11
ENSMUSG00000106379	Lhfpl3	5:22746059-23275597	0.023938	0	11.82747015	1.88E-14	6.06E-11
ENSMUSG00000092544	Gm20422	8:69742862-69774886	0	0.366563	-11.42406119	6.31E-13	1.87E-09
ENSMUSG00000022358	Fbxo32	15:58175879-58214932	42.73755	5.462675	3.085542086	1.16E-08	1.79E-05
ENSMUSG00000107011	Gm42906	5:151063546-151428163	0.105461	0	13.92990845	5.00E-08	6.34E-05
ENSMUSG00000026725	Tnn	1:160085029-160153580	12.21925	4.447636	1.5024048	1.15E-07	0.0001242
ENSMUSG00000021388	Aspn	13:49544443-49567565	35.88648	12.68657	1.57772048	1.32E-07	0.0001377
ENSMUSG00000089675	Ugt1a8	1:88087813-88219004	0.313779	0	13.98710982	2.40E-07	0.0002062
ENSMUSG00000028834	Trim63	4:134315120-134329629	46.58094	7.102054	2.468943247	2.45E-07	0.0002062
ENSMUSG00000040118	Cacna2d1	5:15934691-16374511	19.71149	8.193246	1.569704683	3.66E-07	0.0002885
ENSMUSG00000003038	Hmgn2	4:133964738-133968650	39.40259	117.1841	-1.56144892	4.30E-07	0.0003247
ENSMUSG00000051985	Igfn1	1:135953578-136006342	6.153291	1.298315	2.340052654	4.78E-07	0.0003466
ENSMUSG00000030562	Nox4	7:87246096-87398710	3.153967	0.765463	2.173383642	6.17E-07	0.0004135
ENSMUSG00000052613	Pcdh15	10:73099342-74649737	0.062184	0	9.756033009	6.31E-07	0.0004149
ENSMUSG00000026073	Il1r2	1:40074079-40125231	15.95305	52.10709	-1.756652616	6.58E-07	0.0004248
ENSMUSG00000018893	Mb	15:77014056-77057070	305.4656	86.2569	1.935241256	7.29E-07	0.0004626
ENSMUSG00000059203	Il1rapl2	X:137570608-138846946	0.011286	0	12.23005039	7.65E-07	0.0004765
ENSMUSG00000114923	Gm49345	13:67592159-67637769	0.347182	0	12.08013437	8.80E-07	0.0005318
ENSMUSG00000027470	Mylk2	2:152911352-152923068	53.7736	12.14693	2.2790385	8.83E-07	0.0005318
ENSMUSG00000028773	Fabp3	4:130308595-130315463	83.86241	22.19656	2.038040095	9.80E-07	0.0005595
ENSMUSG00000019577	Pdk4	6:5483351-5496309	121.036	24.41155	2.429790527	9.86E-07	0.0005595
ENSMUSG00000000182	Fgf23	6:127072902-127081408	0.448465	4.06671	-3.122546287	9.92E-07	0.0005595
ENSMUSG00000029361	Nos1	5:117781032-117958840	6.248129	2.358071	2.098066432	1.02E-06	0.0005672
ENSMUSG00000021622	Ckmt2	13:91853387-91876885	195.3319	53.15125	1.999396659	1.05E-06	0.0005731
ENSMUSG00000030470	Csrp3	7:48830398-48848033	77.23468	15.70018	2.384607893	1.83E-06	0.0008888
ENSMUSG00000060923	Acyp2	11:30505991-30649587	9.07397	2.531856	1.956316448	1.93E-06	0.0009133
ENSMUSG00000032495	Lrrc2	9:110951545-110984066	9.310924	1.683001	2.621474811	1.97E-06	0.0009186
ENSMUSG00000047746	Fbxo40	16:36963460-36990467	23.22435	5.335227	2.278451949	2.06E-06	0.0009417
ENSMUSG00000022357	Klhl38	15:58314573-58324169	9.609774	1.588254	2.739146898	2.07E-06	0.0009417
ENSMUSG00000027077	Smtnl1	2:84811176-84822652	14.66208	3.618414	2.144749726	2.35E-06	0.0010554
ENSMUSG00000019892	Lrriq1	10:103046031-103236322	0.043448	0	9.470971063	2.38E-06	0.0010566

ENSMUSG00000034656	Cacna1a	8:84338639-84640427	3.273888	0.827123	1.926059644	2.61E-06	0.0011329
ENSMUSG00000079243	Xirp1	9:120013755-120023598	23.19124	5.321845	2.243905788	2.68E-06	0.0011329
ENSMUSG00000019899	Lama2	10:26980036-27619758	9.935432	3.433511	1.724581757	3.30E-06	0.0013794
ENSMUSG00000026527	Rgs7	1:175059087-175492500	0.864852	0.314131	1.585616317	3.87E-06	0.0015483
ENSMUSG00000032060	Cryab	9:50751325-50756636	74.09354	22.84448	1.671735224	3.99E-06	0.0015762
ENSMUSG00000045875	Adra1a	14:66635251-66771168	0.918553	0.267962	1.913730246	4.22E-06	0.0016468
ENSMUSG00000041565	L3mbtl4	17:68273797-68780086	0	0.034211	-11.50191925	4.33E-06	0.0016704
ENSMUSG00000031710	Ucp1	8:83290352-83298452	35.88223	4.302124	3.20600623	4.65E-06	0.0017761
ENSMUSG00000022270	Retreg1	15:25843180-25973687	38.47421	12.68735	1.831712468	4.75E-06	0.0017938
ENSMUSG00000007097	Atp1a2	1:172271709-172298064	103.9755	29.49184	1.962001481	4.83E-06	0.0018064
ENSMUSG00000015568	Lpl	8:68880491-68907448	138.1473	48.10354	1.640439377	5.38E-06	0.0019714
ENSMUSG00000027068	Dhrs9	2:69380445-69404533	7.491172	20.89101	-1.450018815	5.55E-06	0.002013
ENSMUSG00000101337	Dnah7c	1:46425592-46807476	0.005324	0	9.222345751	5.83E-06	0.0020693
ENSMUSG00000006221	Hspb7	4:141420779-141425748	63.35815	12.69079	2.443454406	6.26E-06	0.0021695
ENSMUSG00000001334	Fndc5	4:129136999-129144593	7.492347	1.877238	2.120806885	6.26E-06	0.0021695
ENSMUSG00000051596	Otop1	5:38275972-38304217	1.144786	0.130732	3.265764881	6.33E-06	0.0021695
ENSMUSG00000021680	Crhbp	13:95431371-95444924	0	0.096153	-9.098146807	6.35E-06	0.0021695
ENSMUSG00000030727	Rabep2	7:126428759-126463103	121.6098	42.51706	1.711247608	6.65E-06	0.0022269
ENSMUSG00000028841	Cnksr1	4:134228041-134238399	6.342169	0.966175	3.041948296	7.11E-06	0.0023611
ENSMUSG00000020738	Sumo2	11:115523102-115536276	2.522937	9.395768	-1.565319893	7.22E-06	0.0023738
ENSMUSG00000062275	Fbxw24	9:109601116-109626057	0	0.051238	-8.983654014	7.34E-06	0.0023772
ENSMUSG00000063558	Aox1	1:58029931-58106413	4.736599	1.350809	1.957604778	7.38E-06	0.0023772
ENSMUSG00000048834	Vstm2a	11:16257724-16427310	0.049492	0	11.19968887	7.43E-06	0.0023772
ENSMUSG00000041014	Nrg3	14:38368952-39473088	0	0.010043	-11.29385143	8.06E-06	0.0025553
ENSMUSG00000031204	Asb12	X:95470201-95557987	6.290251	1.59718	2.110510825	8.15E-06	0.0025575
ENSMUSG00000058952	Cfi	3:129835884-129875332	0	0.069611	-8.990721951	8.21E-06	0.0025575
ENSMUSG00000042529	Kcnj12	11:61022564-61071131	4.634794	1.363054	1.878789119	8.38E-06	0.002586
ENSMUSG00000057604	Lmcd1	6:112273758-112330425	30.13441	8.12511	2.019124355	8.45E-06	0.002586
ENSMUSG00000033510	Otud7a	7:63444751-63759028	0.016982	0	11.2852151	9.03E-06	0.0027184
ENSMUSG00000024526	Cidea	18:67343604-67367785	9.09012	2.462615	1.95509039	9.12E-06	0.002723
ENSMUSG00000057280	Musk	4:58285960-58374303	4.279629	1.307324	2.040270835	9.34E-06	0.0027657
ENSMUSG00000009670	Tex11	X:100838648-101059667	0	0.038448	-11.10264553	1.05E-05	0.0030477
ENSMUSG00000026834	Acvr1c	2:58267453-58357895	2.598557	0.763395	1.754464913	1.08E-05	0.003106
ENSMUSG00000095474	Cldn34c2	X:123576286-123602172	0	0.212383	-11.03625846	1.16E-05	0.0032118
ENSMUSG00000041548	Hspb8	5:116408491-116422864	35.04796	10.53957	1.856879412	1.16E-05	0.0032118

ENSMUSG00000068699	Flnc	6:29433256-29461883	72.14875	17.45787	2.183286383	1.18E-05	0.0032118
ENSMUSG00000039891	Txlnb	10:17796226-17845665	22.43778	5.680056	2.103224787	1.21E-05	0.0032548
ENSMUSG00000074218	Cox7a1	7:30184144-30186078	81.63426	25.91701	1.765268789	1.28E-05	0.0033712
ENSMUSG00000030739	Myh14	7:44605803-44670843	3.304664	1.071492	1.963661942	1.28E-05	0.0033712
ENSMUSG00000025172	Ankrd2	19:42036000-42045110	25.828	4.906259	2.519635388	1.28E-05	0.0033712
ENSMUSG00000022032	Scara5	14:65666403-65764826	5.479423	2.146999	1.415676727	1.29E-05	0.0033712
ENSMUSG00000050315	Synpo2	3:123076519-123236149	7.363445	2.372589	1.82580689	1.39E-05	0.0036061
ENSMUSG00000049134	Nrap	19:56320035-56390037	64.39814	17.78121	1.992079363	1.43E-05	0.0036827
ENSMUSG00000103800	Pcdha8	18:36992371-37187657	0	0.025527	-11.0164291	1.44E-05	0.0036827
ENSMUSG00000034648	Lrrn1	6:107529768-107570214	2.6194	0.842433	1.738422954	1.46E-05	0.0037166
ENSMUSG00000068697	Myoz1	14:20649107-20656540	136.6506	41.37457	1.850548667	1.55E-05	0.0038641
ENSMUSG00000030996	Art1	7:102101743-102113933	28.44281	8.56278	1.943347855	1.56E-05	0.0038641
ENSMUSG00000075307	Klhl41	2:69670120-69684230	64.0874	18.76291	1.903851136	1.71E-05	0.0041473
ENSMUSG00000031382	Asb11	X:164436994-164459170	21.10466	4.537274	2.350509584	1.72E-05	0.0041473
ENSMUSG00000026141	Col19a1	1:24261890-24587472	0.23031	0.059193	2.024913524	1.80E-05	0.0043024
ENSMUSG00000021798	Ldb3	14:34526603-34588682	70.62265	27.97575	1.803730304	1.99E-05	0.0046643
ENSMUSG00000109392	Gm5737	7:120812365-120831193	0.052702	0	8.854646677	2.00E-05	0.0046643
ENSMUSG00000030852	Tacc2	7:130577438-130764785	69.09865	19.7437	2.02833124	2.02E-05	0.0046947
ENSMUSG00000062077	Trim54	5:31116712-31137630	22.79109	6.016348	2.058337012	2.13E-05	0.0049051
ENSMUSG00000046818	Ddit4l	3:137621612-137628333	16.68814	5.041586	1.84466083	2.19E-05	0.0049764
ENSMUSG00000042265	Trem1	17:48232768-48246924	6.268077	17.54244	-1.404262135	2.19E-05	0.0049764
ENSMUSG00000017300	Tnnc2	2:164777161-164779967	268.96	85.56823	1.74695724	2.25E-05	0.0050948
ENSMUSG00000034055	Phka1	X:102513975-102644246	22.9078	7.885369	1.67872897	2.29E-05	0.0051567
ENSMUSG00000027210	Meis2	2:115863064-116065839	3.453525	1.474447	1.431308196	2.44E-05	0.0054111
ENSMUSG00000042010	Acacb	5:114146535-114250761	22.7879	8.282713	1.648198933	2.45E-05	0.0054111
ENSMUSG00000028396	2310002L09	4:73939371-73950846	13.35394	2.645058	2.3119404	2.51E-05	0.005462
ENSMUSG00000078937	Cpt1b	15:89416405-89425863	33.88259	11.00356	1.770066268	2.54E-05	0.0054732
ENSMUSG00000071317	Bves	10:45335772-45372479	4.387417	1.290134	1.804230676	2.62E-05	0.0055445
ENSMUSG00000038763	Alpk3	7:81057600-81105612	18.17029	5.556834	1.854571086	2.62E-05	0.0055445
ENSMUSG00000004558	Ndrg2	14:51905271-51914158	152.191	46.55601	1.753979321	2.64E-05	0.0055577
ENSMUSG00000040147	Maob	X:16709282-16817366	2.877549	1.287172	1.240333502	2.67E-05	0.0055768
ENSMUSG00000055632	Hmcn2	2:31314415-31460738	11.92269	4.237292	1.542168163	2.68E-05	0.0055768
ENSMUSG00000049107	Ntf3	6:126101412-126166910	0.667568	0.187973	1.878120835	2.71E-05	0.0055972
ENSMUSG00000103793	Pcdhga6	18:37707039-37841873	1.691316	0.588827	1.547852622	2.74E-05	0.0055972
ENSMUSG00000022519	Srl	16:4480216-4541816	62.61684	21.91941	1.652980705	2.74E-05	0.0055972

ENSMUSG00000029392	Rilpl1	5:124493080-124531391	19.35411	5.831982	1.754921671	2.78E-05	0.0056108
ENSMUSG00000074001	Klhl40	9:121777607-121783818	18.46147	5.061706	2.063525328	2.78E-05	0.0056108
ENSMUSG00000036745	Ttll7	3:146852367-146984009	7.56145	2.544268	1.903301677	2.82E-05	0.0056619
ENSMUSG00000031636	Pdlim3	8:45885461-45919548	47.47885	13.05726	2.04565702	2.90E-05	0.0057865
ENSMUSG00000104318	Pcdha7	18:36973802-37187657	0.017338	0	10.8272041	3.00E-05	0.0058934
ENSMUSG00000028116	Myoz2	3:123006206-123035015	43.08568	12.12739	1.941596308	3.02E-05	0.0058934
ENSMUSG00000074852	Hpse2	19:42786539-43388355	0.055655	0	8.836975347	3.07E-05	0.0059525
ENSMUSG00000026308	Klhl30	1:91351016-91362416	9.207159	2.525334	1.980905994	3.12E-05	0.0060115
ENSMUSG00000096719	Mrgpra2b	7:47463806-47528862	9.603512	29.17187	-1.543245384	3.13E-05	0.0060115
ENSMUSG00000027022	Xirp2	2:67446002-67526614	61.49236	17.49349	1.944535178	3.18E-05	0.0060415
ENSMUSG00000030111	A2m	6:121635376-121679227	1.341637	4.025118	-1.749820936	3.18E-05	0.0060415
ENSMUSG00000034457	Eda2r	X:97333840-97377216	2.813782	0.703843	2.297834465	3.24E-05	0.0061186
ENSMUSG00000023092	Fhl1	X:56731787-56793346	158.0328	49.16987	1.83372931	3.30E-05	0.0061941
ENSMUSG00000025433	Crisp3	17:40221777-40242288	0.24438	0	10.95724065	3.35E-05	0.0062245
ENSMUSG00000042895	Abra	15:41864076-41869720	12.98857	3.362206	2.060840143	3.58E-05	0.00652
ENSMUSG00000046275	Trarg1	11:76679808-76698664	5.130669	1.784514	1.644994194	3.64E-05	0.0065949
ENSMUSG00000029561	Oasl2	5:114896936-114912234	13.67238	43.22139	-1.596465181	3.68E-05	0.0066025
ENSMUSG00000025488	Cox8b	7:140898945-140900446	100.1704	29.57857	1.86996414	3.74E-05	0.0066745
ENSMUSG00000035296	Sgcg	14:61219115-61258490	9.708931	3.079083	1.736038271	3.84E-05	0.0067915
ENSMUSG00000024471	Myot	18:44334074-44355724	85.22032	24.77791	1.900401868	3.94E-05	0.0069014
ENSMUSG00000001508	Sgca	11:94962791-94976327	13.23749	4.210891	1.728452604	3.94E-05	0.0069014
ENSMUSG00000037139	Myom3	4:135759715-135815564	14.438	4.942859	1.734226515	3.98E-05	0.0069233
ENSMUSG00000025432	Avil	10:127000709-127020994	2.432288	0.373829	2.747887977	4.08E-05	0.0070233
ENSMUSG00000027978	Prss12	3:123446913-123506597	0.784003	0.233683	1.847733072	4.19E-05	0.0071525
ENSMUSG00000020836	Coro6	11:77462411-77470484	20.59466	5.944329	1.944662587	4.34E-05	0.0073377
ENSMUSG00000021702	Thbs4	13:92751590-92794818	29.42743	12.52435	1.336927016	4.41E-05	0.0073834
ENSMUSG00000029096	Htra3	5:35652023-35679782	20.58148	9.214664	1.192944143	4.43E-05	0.0073948
ENSMUSG00000026208	Des	1:75360329-75368579	186.8652	59.84784	1.826142056	4.46E-05	0.0073956
ENSMUSG00000027488	Snta1	2:154376313-154408099	23.78244	9.079703	1.499800523	4.60E-05	0.0075585
ENSMUSG00000029683	Lmod2	6:24597762-24605414	27.67699	8.23553	1.868055836	4.66E-05	0.0076249
ENSMUSG00000027737	Slc7a11	3:49892526-50443614	0.40427	1.207677	-1.750908197	4.81E-05	0.0078438
ENSMUSG00000022935	Grik1	16:87895900-88290265	0.019609	0	10.46804212	4.93E-05	0.0079806
ENSMUSG00000030246	Ldhb	6:142490249-142507957	71.43585	26.29662	1.638632449	4.94E-05	0.0079806
ENSMUSG00000034810	Scn7a	2:66673425-66784914	2.778335	1.051302	1.513257947	5.22E-05	0.0083297
ENSMUSG00000041476	Smpx	X:157698910-157752591	28.7147	8.754654	1.832991606	5.23E-05	0.0083297

ENSMUSG00000032238	Rora	9:68621970-69388246	10.6029	4.665072	1.305129777	5.38E-05	0.0084886
ENSMUSG00000026489	Coq8a	1:180165238-180199602	60.63687	22.96987	1.675603528	5.53E-05	0.0085753
ENSMUSG00000095217	Hist1h2bn	13:21754123-21754503	267.1905	916.006	-1.731034875	5.61E-05	0.0086292
ENSMUSG00000042717	Ppp1r3a	6:14713977-14755274	23.9938	7.164656	1.873947562	5.68E-05	0.0086958
ENSMUSG00000022040	Ephx2	14:66084374-66124500	13.38973	4.526182	1.680529329	5.78E-05	0.0088181
ENSMUSG00000024924	Vldlr	19:27216484-27254231	13.98675	5.337362	1.531469337	5.82E-05	0.0088365
ENSMUSG00000032661	Oas3	5:120753098-120777661	16.02621	40.54997	-1.302275764	6.03E-05	0.0091106
ENSMUSG00000079588	Tmem182	1:40805601-40856887	24.16788	8.208846	1.681585724	6.51E-05	0.009628
ENSMUSG00000023019	Gpd1	15:99717515-99725005	43.0978	19.46275	1.223835413	6.53E-05	0.009628
ENSMUSG00000027530	Fabp12	3:10244209-10301183	0	0.01601	-8.457369262	6.81E-05	0.0099538
ENSMUSG00000021200	Asb2	12:103321142-103356001	41.37096	12.62212	1.842551988	6.86E-05	0.009992
ENSMUSG00000072720	Myo18b	5:112688876-112896362	16.20673	5.403289	1.681270986	6.89E-05	0.009992
ENSMUSG00000004864	Mapk13	17:28769297-28780233	9.435846	22.39678	-1.417506433	7.39E-05	0.0106225
ENSMUSG00000044117	290001100€	16:13986604-14101500	0	0.026982	-10.35106223	7.55E-05	0.0108158
ENSMUSG00000032285	Dnaja4	9:54698873-54716315	17.15816	7.151215	1.371225398	7.67E-05	0.0109431
ENSMUSG00000032643	Fhl3	4:124700701-124708611	37.96975	13.55984	1.611469457	7.77E-05	0.0109431
ENSMUSG00000032561	Acpp	9:104288251-104337748	7.020166	16.65452	-1.254019397	7.81E-05	0.0109431
ENSMUSG00000032357	Tinag	9:76951693-77045794	0.028524	0	10.10084845	7.83E-05	0.0109431
ENSMUSG00000030786	Itgam	7:128062640-128118491	27.32042	70.73823	-1.30807917	7.91E-05	0.0110112
ENSMUSG00000021898	Asb14	14:26894557-26915258	9.752808	3.101149	1.837292873	8.04E-05	0.0110888
ENSMUSG00000029158	Yipf7	5:69516671-69542648	9.048084	2.98193	1.708258242	8.05E-05	0.0110888
ENSMUSG00000031097	Tnni2	7:142441808-142444410	306.1263	98.05576	1.679908955	8.27E-05	0.0112965
ENSMUSG00000033389	Arhgap44	11:65002039-65162961	1.481528	0.620127	1.352075524	8.36E-05	0.0113065
ENSMUSG00000034361	Cpne2	8:94532990-94570531	16.39199	38.43125	-1.181204142	8.42E-05	0.0113065
ENSMUSG00000042254	Cilp	9:65265180-65280605	23.1856	10.6146	1.220216339	8.43E-05	0.0113065
ENSMUSG00000001333	Sync	4:129287617-129308559	5.59245	1.743071	1.785371176	8.44E-05	0.0113065
ENSMUSG00000006435	Neurl1a	19:47178820-47259441	11.05928	4.220681	1.507487341	8.49E-05	0.011331
ENSMUSG00000042828	Trim72	7:128003949-128011033	21.90899	7.753534	1.65970589	8.55E-05	0.0113759
ENSMUSG00000043639	Rbm20	19:53677306-53867080	4.727131	1.570391	1.907032235	8.63E-05	0.0114162
ENSMUSG00000028931	Kcnab2	4:152390742-152477910	8.384676	21.82405	-1.41948083	8.73E-05	0.0114162
ENSMUSG00000037942	Crp	1:172698055-172833031	0.034391	0	10.07227648	8.74E-05	0.0114162
ENSMUSG00000038170	Pde4dip	3:97689263-97888707	153.0351	51.80844	1.563159719	8.77E-05	0.0114162
ENSMUSG00000056973	Ces1d	8:93166068-93197838	19.7741	8.702083	1.407616531	8.77E-05	0.0114162
ENSMUSG00000079386	Gm3173	14:4430992-4519452	0.104747	0	10.20300814	8.87E-05	0.0115044
ENSMUSG00000040740	Slc25a34	4:141618824-141623821	5.088086	0.738406	2.926627879	8.92E-05	0.0115188

ENSMUSG00000021373	Cap2	13:46501848-46650281	13.86539	4.702942	1.646862646	9.00E-05	0.0115683
ENSMUSG00000030399	Ckm	7:19404776-19422841	549.1017	189.8165	1.641998841	9.02E-05	0.0115683
ENSMUSG00000048486	Fitm2	2:163466379-163472629	15.86072	5.835977	1.560291585	9.13E-05	0.0115989
ENSMUSG00000074264	Amy1	3:113555710-113606699	8.841471	2.944392	1.623560759	9.14E-05	0.0115989
ENSMUSG00000098078	Gm26992	X:103422010-103481705	0.025799	1.23778	-5.615768892	9.14E-05	0.0115989
ENSMUSG00000034459	Ifit1	19:34640871-34650009	3.081464	13.20255	-2.001886983	9.38E-05	0.0117761
ENSMUSG00000049641	Vgll2	10:52022502-52028471	14.68443	4.008057	2.023605222	9.52E-05	0.0118688
ENSMUSG00000027999	Pla2g12a	3:129878606-129895825	14.46946	4.810812	1.758985443	9.83E-05	0.0122134
ENSMUSG00000018566	Slc2a4	11:69942539-69948188	48.37256	17.25407	1.581594082	0.0001006	0.0123996
ENSMUSG00000062694	Cav3	6:112459505-112472872	5.457631	1.880166	1.637911594	0.0001032	0.0125485
ENSMUSG00000049265	Kcnk3	5:30588170-30625271	2.851215	1.003923	1.464464951	0.0001055	0.0126962
ENSMUSG00000020032	Nuak1	10:84370905-84440597	9.164796	4.347997	1.186799743	0.000106	0.0126962
ENSMUSG00000052374	Actn2	13:12269426-12340760	100.0815	33.44589	1.69645987	0.0001062	0.0126962
ENSMUSG00000030730	Atp2a1	7:126445858-126463108	1096.527	392.9351	1.601608921	0.0001093	0.0130253
ENSMUSG00000035948	Acss3	10:106933517-107123668	3.437982	1.284594	1.673627764	0.000111	0.0131892
ENSMUSG00000041710	Trpc5	X:144381671-144688180	0	0.010734	-10.15399799	0.0001137	0.0134414
ENSMUSG00000093973	Mrgpra2a	7:47426328-47452139	5.414467	16.42429	-1.538606539	0.0001139	0.0134414
ENSMUSG00000032496	Ltf	9:111019271-111042767	562.7672	1336.084	-1.230858878	0.0001143	0.0134414
ENSMUSG00000030546	Plin1	7:79720218-79732903	23.64677	9.727685	1.291453302	0.0001149	0.0134714
ENSMUSG00000056328	Myh1	11:67200052-67224575	378.699	137.3612	1.560630863	0.0001169	0.0136599
ENSMUSG00000028278	Rragd	4:32983037-33022180	10.63803	4.24705	1.470822086	0.000124	0.0142534
ENSMUSG00000019787	Trdn	10:33080554-33476709	30.29266	13.3421	1.627714024	0.0001252	0.0143467
ENSMUSG00000026697	Myoc	1:162639155-162649693	9.825179	4.057799	1.354144744	0.0001257	0.0143594
ENSMUSG00000029685	Asb15	6:24528144-24573164	3.13861	1.055938	1.690074076	0.0001293	0.0147126
ENSMUSG00000033065	Pfkm	15:98041299-98132451	159.8958	59.46506	1.558632343	0.0001297	0.0147126
ENSMUSG00000027750	Postn	3:54361109-54391037	56.14353	27.91271	1.084766986	0.0001349	0.0151123
ENSMUSG00000032648	Pygm	19:6384399-6398459	239.4248	84.82192	1.625865502	0.0001376	0.015312
ENSMUSG00000031633	Slc25a4	8:46206797-46211284	216.1602	87.32876	1.437497172	0.000139	0.015312
ENSMUSG00000041801	Phlda3	1:135766119-135769136	32.92867	9.681618	1.928298464	0.0001393	0.015312
ENSMUSG00000033880	Lgals3bp	11:118392751-118402092	20.5248	32.26237	-1.328503489	0.0001399	0.015312
ENSMUSG00000076441	Ass1	2:31470207-31520672	5.410749	12.09944	-1.171591615	0.00014	0.015312
ENSMUSG00000027438	Napb	2:148693864-148732467	2.095579	0.483194	2.070094712	0.00014	0.015312
ENSMUSG00000078486	Perm1	4:156202750-156221307	14.89444	5.378176	1.606427931	0.0001417	0.015312
ENSMUSG00000020620	Abca8b	11:109932190-109995845	3.595869	1.439341	1.472046628	0.0001418	0.015312
ENSMUSG00000020216	Jsrp1	10:80808496-80813498	27.06406	9.176079	1.664768708	0.0001423	0.0153149

ENSMUSG00000001027	Scn4a	11:106318592-106353288	17.96371	5.52024	1.647943064	0.0001432	0.0153383
ENSMUSG00000016349	Eef1a2	2:181147653-181157014	98.50509	35.90785	1.574160224	0.0001434	0.0153383
ENSMUSG00000005373	Mlxipl	5:135089890-135138382	9.625982	3.305979	1.745445989	0.0001455	0.0155242
ENSMUSG00000049422	Chchd10	10:75933130-75937747	79.63616	37.43741	1.265167335	0.0001465	0.0155329
ENSMUSG00000018411	Mapt	11:104231390-104332090	5.770558	1.612134	1.793082786	0.0001465	0.0155329
ENSMUSG00000025317	Car5a	8:121916126-121944904	0	0.027538	-8.308648961	0.0001488	0.0155936
ENSMUSG00000044716	Dok7	5:35056766-35087839	2.612186	0.87084	1.669021997	0.0001526	0.0159149
ENSMUSG00000031596	Slc7a2	8:40862396-40922308	8.218212	3.183924	1.702379764	0.0001528	0.0159149
ENSMUSG00000055546	Timd4	11:46810800-46844332	0.29941	1.268117	-2.096880039	0.0001556	0.0161578
ENSMUSG00000060961	Slc4a4	5:88886818-89239653	5.153161	1.883975	1.777719937	0.0001567	0.0162254
ENSMUSG00000061462	Obscn	11:58994256-59139170	101.851	31.07689	1.706036077	0.0001585	0.0163131
ENSMUSG00000051747	Ttn	2:76703980-76982547	343.6501	118.7688	1.700818511	0.0001609	0.0165145
ENSMUSG00000025900	Rp1	1:3999557-4409241	0.304899	0.094084	1.828009221	0.0001631	0.0166985
ENSMUSG00000028631	Kcnq4	4:120696138-120748612	1.766877	0.572317	1.752028452	0.0001637	0.0167044
ENSMUSG00000056900	Usp13	3:32817546-32938071	17.06776	3.908683	2.213226026	0.0001647	0.0167587
ENSMUSG00000001095	Slc13a2	11:78397087-78422217	0	0.025483	-8.095577319	0.0001662	0.0168054
ENSMUSG00000057715	A830018L16	1:11414105-11975901	0.088356	0.010706	2.619995095	0.0001663	0.0168054
ENSMUSG00000036278	Macrod1	19:7056810-7198057	18.84488	8.012123	1.344781206	0.0001665	0.0168054
ENSMUSG00000052212	Cd177	7:24743983-24760311	130.4787	326.942	-1.273610453	0.0001689	0.0168712
ENSMUSG00000002944	Cd36	5:17781690-17888801	110.3746	47.45897	1.391263638	0.0001696	0.0168712
ENSMUSG00000021573	Tppp	13:74009407-74035753	3.967301	1.525943	1.453837753	0.0001697	0.0168712
ENSMUSG00000040265	Dnm3	1:161982453-162478034	2.09025	0.679061	1.919164874	0.0001699	0.0168712
ENSMUSG00000041828	Abca8a	11:110025634-110095978	16.12977	6.279005	1.284260126	0.00017	0.0168712
ENSMUSG00000055489	Ano5	7:51511029-51598709	8.244463	2.982888	1.590442265	0.00017	0.0168712
ENSMUSG00000026387	Sctr	1:120006894-120063536	0.687747	0.170665	2.135212618	0.000171	0.0169209
ENSMUSG00000020407	Upp1	11:9118103-9136170	4.841629	14.22975	-1.535705492	0.0001773	0.0174249
ENSMUSG00000040694	Apobec2	17:48419231-48432930	76.70952	26.89035	1.557676718	0.0001776	0.0174249
ENSMUSG00000039474	Wfs1	5:36966104-36989205	8.511716	3.506189	1.395740865	0.0001791	0.0175264
ENSMUSG00000022548	Apod	16:31296192-31314808	8.450609	3.279588	1.382515419	0.0001804	0.0176027
ENSMUSG00000071714	Csf2rb2	15:78282507-78305721	5.497619	12.51097	-1.161018493	0.0001823	0.0176401
ENSMUSG00000052776	Oas1a	5:120896256-120907521	8.963704	25.42193	-1.417890578	0.0001829	0.0176522
ENSMUSG00000031461	Myom2	8:15057653-15133541	58.70268	19.16568	1.75044397	0.0001844	0.0176588
ENSMUSG00000038670	Mybpc2	7:44501699-44524656	104.6407	38.95401	1.539771591	0.0001869	0.0178441
ENSMUSG00000031791	Tmem38a	8:72572055-72587282	83.47631	31.06907	1.533528798	0.0001885	0.0179285
ENSMUSG00000021838	Samd4	14:46882854-47105815	10.04127	4.186451	1.249010717	0.0001888	0.0179285

ENSMUSG00000034898	Filip1	9:79815051-80012851	6.628143	2.23492	1.570140513	0.0001897	0.0179673
ENSMUSG00000036854	Hspb6	7:30552178-30555443	73.75804	20.46632	1.98765052	0.0001954	0.0184135
ENSMUSG00000027574	Nkain4	2:180934772-180954699	0.155827	0	9.644625981	0.0002002	0.0187664
ENSMUSG00000041624	Gucy1a2	9:3532778-3894736	1.813562	0.817479	1.239185995	0.0002002	0.0187664
ENSMUSG00000019194	Scn1b	7:31116524-31127003	40.56738	17.01505	1.401362576	0.0002025	0.0188743
ENSMUSG00000031274	Col4a5	X:141475385-141689234	0.933474	0.429327	1.119799151	0.0002053	0.0189367
ENSMUSG00000018845	Unc45b	11:82910550-82943403	12.56864	5.00904	1.546268004	0.0002061	0.0189367
ENSMUSG00000025348	Itga7	10:128933818-128958282	12.46675	4.702972	1.491513362	0.0002099	0.0191616
ENSMUSG00000024211	Grm8	6:27275119-28135178	0.296056	0.055428	2.380618266	0.0002127	0.0192722
ENSMUSG00000044951	Mylk4	13:32700834-32784017	24.09707	8.397081	1.635696557	0.0002143	0.0193677
ENSMUSG00000033196	Myh2	11:67171027-67197517	198.5896	72.48721	1.561660689	0.0002155	0.0194267
ENSMUSG00000002688	Prkd1	12:50341231-50649098	2.25146	1.138143	1.163233864	0.0002189	0.0196796
ENSMUSG00000069601	Ank3	10:69398773-70027438	17.33158	7.136751	1.278413603	0.0002197	0.0196848
ENSMUSG00000038967	Pdk2	11:95026258-95041354	33.51602	13.23582	1.428819254	0.00022	0.0196848
ENSMUSG00000026430	Rassf5	1:131176410-131245258	13.07895	37.21209	-1.113885063	0.0002236	0.0199559
ENSMUSG00000038201	Kcna7	7:45405653-45409763	10.17068	2.944042	1.922446986	0.0002251	0.0200406
ENSMUSG00000057101	Zfp180	7:24081924-24107713	2.849505	5.522325	-1.88927264	0.0002292	0.0203499
ENSMUSG00000032369	Plscr1	9:92249750-92272278	10.31531	22.97745	-1.265466757	0.0002307	0.0204305
ENSMUSG00000002831	Plin4	17:56100591-56109803	29.71225	11.99534	1.423697903	0.0002318	0.0204316
ENSMUSG00000040314	Ctsg	14:56099881-56102574	87.33242	223.753	-1.294978081	0.0002366	0.0207473
ENSMUSG00000026950	Neb	2:52136647-52378474	120.4892	43.3016	1.626966541	0.0002372	0.0207473
ENSMUSG00000020061	Mybpc1	10:88518279-88605152	102.1645	39.10801	1.539104021	0.0002391	0.0208674
ENSMUSG00000057897	Camk2b	11:5969644-6066362	7.703621	3.658906	1.401704947	0.0002414	0.0210146
ENSMUSG00000023232	Serinc2	4:130253495-130279205	2.41121	0.698752	1.896752898	0.000243	0.0210571
ENSMUSG00000030785	Cox6a2	7:128205435-128206387	86.06834	25.8316	1.858704419	0.0002436	0.0210571
ENSMUSG00000028427	Aqp7	4:41033074-41048139	8.392672	3.309085	1.43368036	0.0002437	0.0210571
ENSMUSG00000042724	Map3k9	12:81721010-81781175	7.292881	15.49401	-1.050730714	0.000245	0.0211199
ENSMUSG00000040666	Sh3bgr	16:96200450-96228935	23.05876	8.377741	1.614183472	0.0002464	0.0211911
ENSMUSG00000006369	Fbln1	15:85205949-85286535	5.028614	2.41095	1.074457924	0.0002471	0.0212002
ENSMUSG00000021768	Dusp13	14:21733394-21797832	10.98738	3.229444	1.516992539	0.0002493	0.0212868
ENSMUSG00000079105	C7	15:4988762-5063740	0.982036	0.118749	3.087115638	0.0002589	0.0220508
ENSMUSG00000024059	Clip4	17:71768473-71864273	8.778207	3.153622	1.775221394	0.0002604	0.0221239
ENSMUSG00000035963	Odf3l2	10:79639526-79645738	2.873268	0.475757	2.654084323	0.0002741	0.02304
ENSMUSG00000043542	Zc2hc1a	3:7503483-7553836	2.945135	1.375292	1.164466815	0.0002747	0.02304
ENSMUSG00000044086	Lmod3	6:97238534-97252759	10.26372	3.838981	1.538877618	0.000275	0.02304

ENSMUSG00000045667	Smtnl2	11:72389164-72411713	23.29342	7.530087	1.574104406	0.0002758	0.0230497
ENSMUSG00000031400	G6pdx	X:74409483-74429194	50.30819	119.966	-1.232252915	0.0002822	0.0233447
ENSMUSG00000025938	Slco5a1	1:12866549-12992650	3.520324	1.34312	1.503044016	0.0002825	0.0233447
ENSMUSG00000073375	Lrrc30	17:67630964-67632723	14.40246	1.890157	3.044650899	0.0002826	0.0233447
ENSMUSG00000021536	Adcy2	13:68620043-68999541	5.322921	2.569238	1.235093667	0.000284	0.0234018
ENSMUSG00000018865	Sult4a1	15:84076097-84105754	0.055199	0	9.432684858	0.0002882	0.0236958
ENSMUSG00000027513	Pck1	2:173153048-173159273	50.01888	21.05929	1.382226087	0.00029	0.0237871
ENSMUSG00000047246	Hist1h2be	13:23551258-23698454	48.89843	205.4672	-2.503947655	0.0002918	0.0238808
ENSMUSG00000056880	Gadl1	9:115909455-116076176	0.310115	0.10385	1.690010552	0.0002932	0.0239385
ENSMUSG00000066842	Hmcn1	1:150562524-150993435	1.224789	0.588697	1.040055997	0.0002952	0.0240526
ENSMUSG00000041798	Gck	11:5900820-5950081	0.639059	0.099686	2.646070334	0.0003007	0.024442
ENSMUSG00000021238	Aldh6a1	12:84430717-84451004	21.78528	8.310752	1.509590606	0.0003039	0.0246465
ENSMUSG00000028348	Cavin4	4:48663514-48673502	9.410517	3.007317	1.764479627	0.0003064	0.0247643
ENSMUSG00000017412	Cacnb4	2:52428320-52676831	0.116787	0.025157	1.9900196	0.0003072	0.0247643
ENSMUSG00000001227	Sema6b	17:56123085-56140343	4.079338	10.06585	-1.219148841	0.0003075	0.0247643
ENSMUSG00000005716	Pvalb	15:78191114-78206400	465.2111	183.77	1.443855004	0.0003134	0.0251259
ENSMUSG00000045975	C2cd2	16:97855209-97962621	12.17309	6.883272	1.251088509	0.0003169	0.0252472
ENSMUSG00000078566	Bnip3	7:138890836-138909519	55.29247	22.0517	1.471723767	0.000317	0.0252472
ENSMUSG00000071713	Csf2rb	15:78325752-78353847	15.82764	36.73141	-1.166021087	0.000318	0.0252692
ENSMUSG00000018428	Akap1	11:88830792-88864586	14.49713	6.297957	1.297161797	0.0003288	0.026009
ENSMUSG00000028464	Tpm2	4:43514711-43523765	167.1576	67.44248	1.425485944	0.0003325	0.0262435
ENSMUSG00000026109	Tmeff2	1:50900647-51187270	0.409384	0.143916	1.594165813	0.0003339	0.0262989
ENSMUSG00000032845	Alpk2	18:65265529-65394066	6.670462	2.658362	1.428275525	0.0003363	0.0264252
ENSMUSG00000064357	mt-Atp6	MT:7927-8607	1087.69	186.6355	2.734599159	0.0003412	0.0267508
ENSMUSG00000071604	Fam189a2	19:23972750-24031019	4.144912	1.853345	1.280839063	0.0003425	0.0267975
ENSMUSG00000033182	Kbtbd12	6:88545114-88637950	2.827035	1.223472	1.727429114	0.0003449	0.026924
ENSMUSG00000026407	Cacna1s	1:136052750-136119822	36.74998	14.14679	1.5023334	0.0003475	0.0270662
ENSMUSG00000041731	Pgm5	19:24683016-24861855	5.092443	2.253198	1.36123594	0.0003512	0.0272993
ENSMUSG00000093985	Gm10406	14:7006115-7027449	0	0.026719	-7.884636049	0.0003541	0.0274314
ENSMUSG00000026131	Dst	1:33908225-34308661	57.5793	23.24246	1.303941089	0.0003545	0.0274314
ENSMUSG00000057729	Prtn3	10:79874471-79883174	145.401	343.5801	-1.193062486	0.0003553	0.0274316
ENSMUSG00000022053	Ebf2	14:67233291-67430918	2.147978	1.032202	1.25611302	0.0003565	0.0274674
ENSMUSG00000031972	Acta1	8:123891769-123894751	1995.173	800.5092	1.423226418	0.0003582	0.0274765
ENSMUSG00000071540	3425401B19	14:32659119-32685293	17.16802	6.404158	1.560106726	0.0003589	0.0274765
ENSMUSG00000019848	Popdc3	10:45178098-45318452	4.506322	1.23742	2.029492854	0.0003639	0.0277988

ENSMUSG00000033355	Rtp4	16:23520291-23614222	3.996206	11.84926	-1.591485757	0.0003677	0.0280227
ENSMUSG00000020354	Sgcd	11:46896253-47989377	11.41305	5.104836	1.363620094	0.0003699	0.0280711
ENSMUSG00000035522	Tsga8	X:82948902-83955069	0.783297	0.235793	1.860147611	0.0003699	0.0280711
ENSMUSG00000020067	Mypn	10:63115795-63203952	12.86633	4.945509	1.516527503	0.0003774	0.0285428
ENSMUSG00000025473	Adam8	7:139978932-139992562	22.52209	47.72245	-1.034653982	0.0003846	0.0289418
ENSMUSG00000005628	Tmod4	3:95124476-95129209	31.68739	12.41738	1.505500573	0.0003863	0.0289651
ENSMUSG00000042686	Jph1	1:16964560-17097889	14.62263	5.867949	1.446520514	0.0003873	0.0289651
ENSMUSG00000028259	Fhl5	4:25199908-25242876	2.341777	0.710412	1.797579612	0.000388	0.0289651
ENSMUSG00000037709	Fam13a	6:58932090-59024549	5.399931	2.036291	1.459695724	0.0003909	0.0291085
ENSMUSG00000030220	Arhgdib	6:136923655-136941899	228.0787	557.1156	-1.165349792	0.0003936	0.029184
ENSMUSG00000027961	Lrrc39	3:116562973-116583134	4.784914	1.782126	1.534127857	0.0003984	0.0294791
ENSMUSG00000045103	Dmd	X:82948870-85206141	20.30047	7.071698	1.612634582	0.0003999	0.0295304
ENSMUSG00000062515	Fabp4	3:10204088-10208576	233.547	108.6795	1.144797942	0.0004018	0.029608
ENSMUSG00000030672	Mylpf	7:127208890-127214298	513.0169	207.4863	1.398820012	0.0004085	0.0300175
ENSMUSG00000003477	Inmt	6:55170626-55175043	4.333841	0.550827	3.103861814	0.0004099	0.0300191
ENSMUSG00000022091	Sorbs3	14:70180468-70211989	8.760634	4.15461	1.15590658	0.0004109	0.0300275
ENSMUSG00000041827	Oasl1	5:114923240-114937915	1.911931	7.181055	-1.650906507	0.0004141	0.0301367
ENSMUSG00000066705	Fxyd6	9:45370185-45396159	16.31168	7.646415	1.205788339	0.0004187	0.0302524
ENSMUSG00000062908	Acadm	3:153922357-153944632	38.77472	19.04353	1.150789328	0.000419	0.0302524
ENSMUSG00000046480	Scn4b	9:45138395-45154152	21.75343	8.651344	1.46019338	0.0004221	0.030337
ENSMUSG00000031610	Scrg1	8:57436358-57479597	3.324381	8.981932	-1.693228969	0.0004263	0.0304935
ENSMUSG00000017697	Ada	2:163726584-163750239	4.407146	10.04614	-1.195033906	0.0004273	0.0304935
ENSMUSG00000021903	Galnt15	14:32028989-32062197	9.532159	3.295247	1.620916934	0.0004312	0.030589
ENSMUSG00000021123	Rdh12	12:79208914-79222665	8.079787	16.6035	-1.052551179	0.0004315	0.030589
ENSMUSG00000030921	Trim30a	7:104409025-104465193	14.81857	33.32679	-1.137100924	0.0004363	0.0307606
ENSMUSG00000038034	Igsf8	1:172261641-172319841	16.14295	9.157732	1.440194592	0.0004415	0.0309715
ENSMUSG00000002500	Rpl3l	17:24727820-24736143	35.27475	12.91538	1.579428521	0.0004425	0.0309715
ENSMUSG00000025129	Ppp1r27	11:120549979-120551132	35.17649	8.974157	2.107684035	0.0004447	0.0309715
ENSMUSG00000058297	Spock2	10:60106219-60135198	2.733132	0.933798	1.589851684	0.0004489	0.0312017
ENSMUSG00000048416	Mlf1	3:67374097-67400003	25.02549	9.78983	1.460516796	0.0004519	0.03135
ENSMUSG00000027716	Trpc3	3:36620482-36690167	2.05851	0.721619	1.467510353	0.0004538	0.0314224
ENSMUSG00000025006	Sorbs1	19:40294753-40513779	30.53897	14.88342	1.124999146	0.0004553	0.0314658
ENSMUSG00000070424	Art5	7:102096879-102111145	7.979681	1.740775	2.376239679	0.0004592	0.0316738
ENSMUSG00000026207	Speg	1:75375297-75432320	15.30555	6.931964	1.458542917	0.0004646	0.031983
ENSMUSG00000090053	Palm2	4:57434247-57712016	0.714177	0.203218	2.404900301	0.0004678	0.0320774

ENSMUSG00000005994	Tyrp1	4:80834123-80851719	0.038448	0	7.704649021	0.0004692	0.0320842
ENSMUSG00000026817	Ak1	2:32621758-32635058	86.996	37.7089	1.315541992	0.0004698	0.0320842
ENSMUSG00000027792	Bche	3:73635808-73708415	2.376175	1.042098	1.430188961	0.0004727	0.0320958
ENSMUSG00000001025	S100a6	3:90612882-90624181	108.0871	244.2054	-1.103938514	0.0004729	0.0320958
ENSMUSG00000038086	Hspb2	9:50751078-50756635	15.33196	5.426811	1.682779902	0.0004735	0.0320958
ENSMUSG00000047419	Cmya5	13:93040713-93144724	50.18784	18.25869	1.580498629	0.000477	0.0321444
ENSMUSG00000009350	Mpo	11:87793581-87804413	258.037	598.6785	-1.131711281	0.0004778	0.0321444
ENSMUSG00000030592	Ryr1	7:29003344-29125179	79.72203	28.28175	1.442501797	0.0004778	0.0321444
ENSMUSG00000068794	Col28a1	6:7997808-8192617	0.847944	0.379661	1.176807371	0.0004813	0.0323137
ENSMUSG00000001604	Tcea3	4:136247729-136274898	14.61494	5.463129	1.527178617	0.000483	0.0323677
ENSMUSG00000030278	Cidec	6:113424634-113435760	25.83148	10.30424	1.431397085	0.0004897	0.0326964
ENSMUSG00000018574	Acadvl	11:70010183-70015411	57.39599	30.03398	1.267213896	0.0004897	0.0326964
ENSMUSG00000030554	Synm	7:67730160-67759742	18.10001	7.475387	1.381938944	0.0004912	0.0327338
ENSMUSG00000019913	Sim1	10:50894754-50989152	0.323896	0.080885	2.367928686	0.0004924	0.032752
ENSMUSG00000031709	Tbc1d9	8:83165352-83272934	1.0703	2.214696	-0.983215415	0.0005028	0.0333171
ENSMUSG00000040485	Lrrc52	1:167445669-167466780	0.698138	0.045076	4.049432143	0.0005096	0.0336337
ENSMUSG00000042082	Arsb	13:93771630-93943016	17.59782	36.81313	-1.039871916	0.0005106	0.0336337
ENSMUSG00000024049	Myom1	17:70994291-71126856	63.33889	27.95504	1.358832727	0.0005113	0.0336337
ENSMUSG00000033377	Palmd	3:116918258-116968987	4.792548	1.805654	1.572728161	0.000513	0.0336783
ENSMUSG00000091722	Siah3	14:75455982-75529708	0.01837	0	9.139240619	0.0005141	0.0336925
ENSMUSG00000060600	Eno3	11:70657202-70662513	327.9492	136.2376	1.380079418	0.000516	0.0337
ENSMUSG00000055493	Epm2a	10:11343404-11459644	6.341632	3.028804	1.174426575	0.0005161	0.0337
ENSMUSG00000036030	Prtg	9:72806874-72917291	0.355638	0.112173	1.723529991	0.0005179	0.0337539
ENSMUSG00000017002	Slpi	2:164354070-164389155	42.75036	98.34836	-1.159010911	0.0005229	0.0340154
ENSMUSG00000028172	Tacr3	3:134829007-134934579	0	0.014044	-9.296359415	0.0005238	0.0340157
ENSMUSG00000027360	Hdc	2:126593667-126619299	31.49973	67.77786	-1.061107111	0.0005331	0.0344288
ENSMUSG00000055737	Ghr	15:3317760-3583492	26.93319	13.67765	1.115736581	0.0005417	0.0348566
ENSMUSG00000029219	Slc10a4	5:73006883-73012955	0.084209	0	7.848185012	0.0005509	0.0352551
ENSMUSG00000070471	Erich6	3:58616300-58637207	0	0.020071	-7.51845169	0.0005631	0.0357168
ENSMUSG00000006498	Ptbp1	10:79854427-79864771	42.59978	80.52774	-0.950953966	0.0005743	0.0362779
ENSMUSG00000025330	Padi4	4:140745865-140774236	38.2876	72.73609	-0.996120393	0.0005767	0.0362779
ENSMUSG00000038357	Camp	9:109847379-109849617	799.8277	1729.122	-1.060609848	0.0005784	0.0362779
ENSMUSG00000062329	Cytl1	5:37735519-37739820	127.1016	62.30598	1.072870576	0.0005787	0.0362779
ENSMUSG00000028150	Rorc	3:94372794-94398276	9.009652	2.683952	1.866106399	0.000579	0.0362779
ENSMUSG00000037940	Inpp4b	8:81342556-82127914	6.782241	3.650341	1.009522441	0.0005791	0.0362779

ENSMUSG00000024597	Slc12a2	18:57878678-57946821	11.08163	5.318614	1.157732506	0.0005862	0.0366543
ENSMUSG00000017491	Rarb	14:16430839-16819156	1.622786	0.857485	1.231367603	0.0005962	0.0372193
ENSMUSG00000037071	Scd1	19:44394455-44407879	252.8883	120.9531	1.109439381	0.0006012	0.0373946
ENSMUSG00000037095	Lrg1	17:56119678-56122001	68.73282	175.3197	-1.286260472	0.0006025	0.0374145
ENSMUSG00000059108	Ifitm6	7:141015699-141017924	179.9178	500.2526	-1.411957885	0.0006098	0.0377523
ENSMUSG00000022584	Ly6c2	15:75045013-75111970	141.9829	342.4725	-1.193462344	0.000612	0.0377523
ENSMUSG00000053338	Tarm1	7:3486500-3502624	5.860881	13.81496	-1.206632675	0.0006124	0.0377523
ENSMUSG00000096146	Kcnj11	7:46093953-46100764	6.964752	2.262684	1.822203395	0.0006129	0.0377523
ENSMUSG00000039914	Coq10a	10:128337754-128368997	26.95833	10.69604	1.272413879	0.0006133	0.0377523
ENSMUSG00000069793	Slfn9	11:82978390-82991830	4.30694	7.779148	-1.230042558	0.0006155	0.0378221
ENSMUSG00000058975	Kcnc1	7:46396497-46438704	5.046232	1.925001	1.531023079	0.0006232	0.0382331
ENSMUSG00000035357	Pdzrn3	6:101149609-101377897	5.38385	2.454673	1.232723448	0.000631	0.0385742
ENSMUSG00000028518	Prkaa2	4:105029874-105109890	12.03549	4.824817	1.441167515	0.0006383	0.0388463
ENSMUSG00000046598	Bdh1	16:31422280-31458901	5.083396	2.074348	1.628858799	0.0006387	0.0388463
ENSMUSG00000061780	Cfd	10:79890853-79892655	237.3345	113.3119	1.144591981	0.0006421	0.0389335
ENSMUSG00000000126	Wnt9a	11:59306928-59333552	1.433823	0.457899	1.756492496	0.0006453	0.0389779
ENSMUSG00000007877	Tcap	11:98383811-98384953	107.6186	45.95685	1.342059928	0.0006529	0.0393734
ENSMUSG00000038522	Mfsd4b1	10:40001575-40025268	1.167587	0.281468	2.181269099	0.0006557	0.0394713
ENSMUSG00000078234	Klhdc7a	4:139960220-139968026	0.565772	0	11.11003319	0.0006734	0.0401963
ENSMUSG00000041324	Inhba	13:16011851-16031621	4.842655	10.50623	-1.033729865	0.0006785	0.0404337
ENSMUSG00000019929	Dcn	10:97479609-97518143	176.1185	97.0832	0.914582358	0.000688	0.0407264
ENSMUSG00000062980	Cped1	6:21985916-22256404	10.3363	5.221708	1.097778698	0.0006891	0.0407264
ENSMUSG00000079808	AC168977.1	GL456212.1:128555-15045	0	0.057939	-9.053431981	0.0006899	0.0407264
ENSMUSG00000067279	Ppp1r3c	19:36731731-36736653	36.14538	14.63435	1.374539927	0.0006903	0.0407264
ENSMUSG00000022860	Chodl	16:78930948-78951733	3.827541	1.357737	1.654517895	0.0006994	0.0411182
ENSMUSG00000026180	Cxcr2	1:74153989-74161246	30.62557	71.76025	-1.178317014	0.0007022	0.0411182
ENSMUSG00000043286	Pnpla1	17:28858411-28890309	0.437105	1.318057	-1.512839324	0.0007026	0.0411182
ENSMUSG00000024726	Carnmt1	19:18670764-18707200	8.138224	4.025699	1.053360112	0.0007049	0.0411827
ENSMUSG00000040489	Sox30	11:45980310-46017994	0.031501	0	8.949372753	0.0007106	0.041338
ENSMUSG00000038403	Hjv	3:96525172-96529210	32.17383	12.92116	1.433857696	0.0007114	0.041338
ENSMUSG00000043705	Capn13	17:73306464-73400866	0	0.024848	-9.058010037	0.0007119	0.041338
ENSMUSG00000045761	Togaram2	17:71673261-71729669	1.445458	0.353994	2.040948552	0.0007123	0.041338
ENSMUSG00000006764	Tph2	10:115078641-115185022	0	0.012753	-9.009695923	0.0007174	0.0415136
ENSMUSG00000052396	Mogat2	7:99219084-99238619	3.745837	8.21243	-1.093543002	0.0007221	0.0416342
ENSMUSG00000102692	Dchs2	3:83127948-83357209	1.145511	0.483548	1.344217368	0.0007251	0.0417431

ENSMUSG00000030862	Cpxm2	7:132032687-132154739	3.324056	1.657566	1.038200629	0.0007286	0.0418753
ENSMUSG00000069306	Hist1h4m	13:21811746-21812147	270.1208	921.1078	-1.710837941	0.0007301	0.0418936
ENSMUSG00000038257	Glra3	8:55940460-56130070	0	0.009331	-9.034016457	0.0007317	0.041905
ENSMUSG00000003410	Elavl3	9:22015005-22052023	0.016723	0	7.453392424	0.0007336	0.041905
ENSMUSG00000095687	Rnaset2a	17:8115445-8148097	6.256528	11.94618	-1.071867345	0.0007363	0.041905
ENSMUSG00000068129	Cst7	2:150570415-150578944	8.004554	19.06755	-1.219950846	0.0007382	0.041905
ENSMUSG00000003283	Hck	2:153108468-153151441	28.60422	62.38205	-1.080115058	0.0007387	0.041905
ENSMUSG00000103677	Pcdhga4	18:37685210-37841873	1.927209	0.398177	2.206830296	0.0007391	0.041905
ENSMUSG00000046080	Clec9a	6:129408862-129424763	2.105712	0.835528	1.791823891	0.0007402	0.041905
ENSMUSG00000043460	Elfn2	15:78667331-78718771	0	0.015726	-7.408663309	0.000744	0.0420146
ENSMUSG00000038239	Hrc	7:45335290-45338974	27.80247	10.44296	1.514691791	0.0007473	0.0420711
ENSMUSG00000090066	1110002E22	3:138065052-138081506	9.775877	3.765058	1.462820405	0.0007486	0.0420711
ENSMUSG00000022562	Oplah	15:76296601-76331104	7.874615	3.65141	1.304198996	0.0007486	0.0420711
ENSMUSG00000025920	Stau2	1:16228674-16520112	5.840255	2.831826	1.089382423	0.0007522	0.0421736
ENSMUSG00000003644	Rps6ka1	4:133847290-133887797	44.68436	89.88707	-1.025134419	0.0007528	0.0421736
ENSMUSG00000061723	Tnnt3	7:142498836-142516009	435.1589	168.8822	1.501864358	0.0007546	0.0422064
ENSMUSG00000009210	Prr29	11:106317555-106388515	1.98037	0.831912	1.592950044	0.000756	0.0422191
ENSMUSG00000109564	Muc16	9:18495455-18674530	0.160597	0.014963	2.616117421	0.0007585	0.0422925
ENSMUSG00000028223	Decr1	4:15917240-15945507	16.83153	8.456658	1.169617844	0.0007615	0.0423652
ENSMUSG00000028441	1110017D15	4:41505009-41517333	0.036376	0	7.333719925	0.000764	0.0424009
ENSMUSG00000022383	Ppara	15:85734983-85802819	5.372448	1.821335	1.538709947	0.0007693	0.042603
ENSMUSG00000038045	Sult6b1	17:78883938-78906992	0	0.053588	-9.00933516	0.0007712	0.042603
ENSMUSG00000059089	Fcgr4	1:171018920-171029761	8.649214	21.04021	-1.251458849	0.0007817	0.0430489
ENSMUSG00000042734	Ttc9	12:81631249-81667557	2.885893	1.046898	1.505540219	0.0007912	0.0434352
ENSMUSG00000031782	Coq9	8:94838321-94854895	38.02781	18.04017	1.205086716	0.0007938	0.0435089
ENSMUSG00000025479	Cyp2e1	7:140763739-140774987	45.66081	12.48919	2.004675728	0.0008036	0.043915
ENSMUSG00000040505	Abcg5	17:84658234-84683011	0.090172	0	7.533129704	0.0008064	0.0439498
ENSMUSG00000027805	Pfn2	3:57841895-57848079	17.49059	6.335105	1.556620979	0.0008068	0.0439498
ENSMUSG00000027499	Pkia	3:7366669-7445366	32.15092	13.62333	1.363386968	0.0008159	0.0443135
ENSMUSG00000027199	Gatm	2:122594467-122611303	14.46358	29.96143	-0.981937791	0.0008193	0.0444169
ENSMUSG00000027514	Zbp1	2:173206612-173218923	3.612648	12.33683	-1.526393032	0.0008203	0.0444169
ENSMUSG00000015889	Lta4h	10:93453411-93484875	89.46437	179.4685	-0.974248513	0.0008218	0.0444256
ENSMUSG00000030401	Rtn2	7:19282624-19296160	47.83997	19.37319	1.416876302	0.000823	0.0444256
ENSMUSG00000038663	Fsd2	7:81533308-81566944	9.645516	4.142168	1.329604945	0.0008251	0.044472
ENSMUSG00000021930	Spryd7	14:61531993-61556886	2.986287	1.448822	1.243242051	0.0008366	0.045015

ENSMUSG00000021957	Tkt	14:30548359-30574720	120.5113	231.6222	-1.019366207	0.0008377	0.045015
ENSMUSG00000005360	Slc1a3	15:8634124-8710764	5.795611	2.582436	1.181019051	0.0008566	0.0459575
ENSMUSG00000006457	Actn3	19:4861216-4877909	142.1935	57.80533	1.412479982	0.0008661	0.046401
ENSMUSG00000073600	Prob1	18:35650351-35655238	7.975732	2.45523	1.85483156	0.0008695	0.0464058
ENSMUSG00000031519	Asb5	8:54520454-54587842	24.45012	6.823951	1.463434304	0.0008699	0.0464058
ENSMUSG00000022876	Samsn1	16:75858793-76022281	14.38061	32.63545	-1.211259362	0.0008707	0.0464058
ENSMUSG00000041660	Bbox1	2:110262697-110314560	0.027106	0	9.123593905	0.0008714	0.0464058
ENSMUSG00000055027	Smyd1	6:71213940-71322233	21.61866	8.247411	1.519107176	0.0008762	0.046484
ENSMUSG00000078763	Slfn1	11:83116849-83122670	25.79166	58.05199	-1.109944496	0.0008768	0.046484
ENSMUSG00000015340	Cybb	X:9435252-9487771	110.5372	233.3955	-1.046050904	0.0008856	0.0468195
ENSMUSG00000002588	Pon1	6:5168090-5193946	0.735494	0.11359	2.816781658	0.0008858	0.0468195
ENSMUSG00000025808	Ccdc7a	8:128734235-129065517	0	0.021422	-8.933066755	0.0008917	0.0470622
ENSMUSG00000074207	Adh1	3:138260991-138290698	5.01235	2.05163	1.362847201	0.0009	0.04733
ENSMUSG00000057003	Myh4	11:67238029-67260446	406.4791	153.4356	1.517793992	0.0009008	0.04733
ENSMUSG00000030353	Tead4	6:128224288-128300823	1.709133	0.805304	1.314791115	0.0009084	0.047657
ENSMUSG00000004383	Large1	8:72814599-73353540	6.779166	3.24062	1.222132314	0.0009114	0.0477071
ENSMUSG00000079110	Capn3	2:120456019-120504913	8.303796	3.722422	1.581335353	0.000912	0.0477071
ENSMUSG00000066861	Oas1g	5:120871632-120887613	1.688981	5.917646	-1.798334685	0.0009284	0.0483533
ENSMUSG00000036144	Meox2	12:37108540-37179534	4.374673	1.878678	1.336448532	0.0009324	0.0484863
ENSMUSG00000072620	Slfn2	11:83065112-83070678	50.84579	103.9642	-0.987036896	0.0009409	0.0488608
ENSMUSG00000055320	Tead1	7:112679318-112906807	13.08888	6.621105	1.191580843	0.0009477	0.049132
ENSMUSG00000021579	Lrrc14b	13:74359578-74364005	8.668826	2.689998	1.763523302	0.0009489	0.049132
ENSMUSG00000042567	Nek10	14:14803415-15012059	0.058872	0.009501	2.854798769	0.0009576	0.0494386

Table S4_KEGG analysis of differentially expressed mRNA_top20

pathway_term	rich_factor	qvalue	gene_number
PPAR signaling pathway	0.158536585	0.0006894	13
Hypertrophic cardiomyopathy (HCM)	0.154761905	0.0006894	13
Arrhythmogenic right ventricular cardiomyopathy (ARVC)	0.162162162	0.0006894	12
Dilated cardiomyopathy	0.146067416	0.0006894	13
Propanoate metabolism	0.185185185	0.0919799	5
Calcium signaling pathway	0.077348066	0.0919799	14
Adrenergic signaling in cardiomyocytes	0.079470199	0.1197424	12
Cardiac muscle contraction	0.102564103	0.1197424	8
Focal adhesion	0.06763285	0.1832602	14
Adipocytokine signaling pathway	0.097222222	0.210457	7
Arginine and proline metabolism	0.101694915	0.2310365	6
Salivary secretion	0.090909091	0.2310365	7
Oxytocin signaling pathway	0.069620253	0.2310365	11
Insulin signaling pathway	0.070422535	0.2657503	10
Glycolysis / Gluconeogenesis	0.092307692	0.2865822	6
AMPK signaling pathway	0.069767442	0.2954286	9
Fatty acid metabolism	0.1	0.2954286	5
ECM-receptor interaction	0.079545455	0.2954286	7
Proximal tubule bicarbonate reclamation	0.136363636	0.3944773	3
Tyrosine metabolism	0.102564103	0.3944773	4

Table S5_M_K_vs_M_W.lncRNA.different gene

gene_id	gene_name	gene_locus	M_K_FPKM	M_W_FPKM	log2FoldChange	pvalue	padj
ENSMUSG00000031849	Comp	8:70373558-70382066	3.441247667	85.92699833	-4.585136053	3.05E-26	5.41E-22
XLOC_139731	XLOC_139731	8:86798464-86809300	2.710059667	0	13.30118531	1.22E-23	1.45E-19
ENSMUSG00000115410	2810457G06Rik	14:46882842-47053397	1.369764333	0.121854667	4.05047467	1.12E-22	9.96E-19
ENSMUSG00000100120	Gm553	1:52522902-52582679	0.185377	0	12.25649471	9.14E-19	5.41E-15
XLOC_153739	XLOC_153739	X:9747754-9786815	0.290731333	0	12.3519825	2.88E-17	1.46E-13
ENSMUSG00000051510	Mafg	11:120625117-120633600	13.70150067	29.56415233	-2.829998116	1.57E-15	6.99E-12
XLOC_051387	XLOC_051387	15:33843805-33884624	0.373486667	0	12.60024891	1.23E-14	4.38E-11
ENSMUSG00000107985	Gm35037	7:3451357-3475020	0	0.208081333	-11.01856398	1.15E-11	3.11E-08
ENSMUSG00000114372	2310067P03Rik	13:83065457-83194347	0.064209333	0	11.93291445	1.23E-11	3.11E-08
ENSMUSG00000109814	Gm45847	7:123031415-123068305	0.135538667	0	10.78545829	1.41E-10	3.34E-07
ENSMUSG00000101734	4933400C23Rik	9:92717456-92795711	0	0.13662	-10.76860289	1.64E-10	3.65E-07
ENSMUSG00000086503	Xist	X:103460366-103483254	0.009730333	51.54281267	-12.16045965	2.00E-10	4.18E-07
ENSMUSG00000086801	Gm15943	15:58176161-58324111	2.377397	0.212147333	3.285871835	2.92E-09	5.19E-06
ENSMUSG00000082488	1700119I11Rik	13:91853157-91876869	12.89909767	3.114599333	2.200284115	4.03E-09	6.82E-06
ENSMUSG00000074388	Gm5544	3:97930173-97967018	0	0.091534	-10.49170496	2.64E-08	3.69E-05
ENSMUSG00000102548	Gm16701	1:166974023-167079312	0.275227667	0	13.66260252	2.70E-08	3.69E-05
ENSMUSG00000097582	Gm26527	13:93770950-93943089	0	0.169617667	-13.6081311	4.38E-08	5.76E-05
XLOC_068279	XLOC_068279	17:64217189-64234819	0.178851	0	10.37655049	7.56E-08	8.95E-05
ENSMUSG00000087467	Gm13601	2:67446003-67526614	3.357779	0.778429333	2.223122349	1.11E-07	0.000122933
ENSMUSG00000087518	Gm13561	2:62357071-62470913	0	0.020983333	-9.970934253	1.42E-07	0.0001444
XLOC_012472	XLOC_012472	10:37541332-37615839	0	0.25555	-12.97411157	1.54E-07	0.000152092
ENSMUSG00000087514	Gm16076	1:69091913-69106740	0.147819	0	9.976471226	1.73E-07	0.000166405
XLOC_077515	XLOC_077515	19:36852209-36915052	0.238979	0	12.81799404	2.33E-07	0.00020616
ENSMUSG00000028834	Trim63	4:134315120-134329629	46.580937	7.102054333	2.468943247	2.45E-07	0.00020616
ENSMUSG00000098072	Gm26995	5:117781069-117955421	0.580305333	0.097631	2.650542284	2.50E-07	0.00020616
ENSMUSG00000054510	Gm14461	2:78237547-78302230	0	0.079439333	-9.637644153	3.21E-07	0.000259325
ENSMUSG00000040118	Cacna2d1	5:15934691-16374511	19.71149133	8.193246333	1.569704683	3.66E-07	0.000288522
ENSMUSG00000117979	AC150898.1	19:24295079-24558401	0	0.059951	-12.67636786	4.19E-07	0.000323791
ENSMUSG00000003038	Hmgn2	4:133964738-133968650	39.40259167	117.1840847	-1.56144892	4.30E-07	0.000324658
XLOC_013803	XLOC_013803	10:77359458-77418254	0.207834333	0	12.51682497	4.47E-07	0.000330531
ENSMUSG00000030562	Nox4	7:87246096-87398710	3.153966667	0.765463333	2.173383642	6.17E-07	0.000413465
ENSMUSG00000052613	Pcdh15	10:73099342-74649737	0.062184333	0	9.756033009	6.31E-07	0.000414896
ENSMUSG00000018893	Mb	15:77014056-77057070	305.4655867	86.25690233	1.935241256	7.29E-07	0.000462572
ENSMUSG00000059203	Il1rapl2	X:137570608-138846946	0.011286333	0	12.23005039	7.65E-07	0.000476513

ENSMUSG00000098230	1700095B10Rik	5:112688876-112896403	2.461242667	0.706829667	1.945248886	9.25E-07	0.000547429
ENSMUSG00000019577	Pdk4	6:5483351-5496309	121.0359957	24.41154533	2.429790527	9.86E-07	0.000559478
ENSMUSG00000029361	Nos1	5:117781032-117958840	6.248129	2.358070667	2.098066432	1.02E-06	0.000567205
ENSMUSG00000116989	Gm49709	16:90349417-90362580	0.115928333	0	9.413546213	1.17E-06	0.000627001
XLOC_068078	XLOC_068078	17:55905746-55910656	0	0.373102	-9.599054531	1.30E-06	0.000687033
ENSMUSG00000112707	D830005E20Rik	10:33083526-33475472	2.409885333	0.662726	1.957285827	1.61E-06	0.000835807
XLOC_142230	XLOC_142230	8:55100395-55401752	0.78752	0.079712667	3.276902271	1.62E-06	0.000835807
XLOC_058158	XLOC_058158	16:25145203-25178075	0	0.263799333	-11.85002946	1.65E-06	0.00083955
XLOC_139675	XLOC_139675	8:85461249-85471904	0.149056667	0	9.396331671	1.79E-06	0.000884203
ENSMUSG00000030470	Csrp3	7:48830398-48848033	77.23467633	15.70018333	2.384607893	1.83E-06	0.000888825
ENSMUSG00000047746	Fbxo40	16:36963460-36990467	23.224345	5.335227	2.278451949	2.06E-06	0.000941657
ENSMUSG00000019892	Lrriq1	10:103046031-103236322	0.043448333	0	9.470971063	2.38E-06	0.001056559
ENSMUSG00000034656	Cacna1a	8:84338639-84640427	3.273888	0.827122667	1.926059644	2.61E-06	0.001132853
ENSMUSG00000085715	Tsix	X:103431517-103484977	0.059331333	1.143857667	-4.833504845	2.66E-06	0.001132853
ENSMUSG00000019899	Lama2	10:26980036-27619758	9.935432	3.433511	1.724581757	3.30E-06	0.00137938
ENSMUSG00000105245	Gm31305	3:97689725-97868423	9.929486333	3.239844667	1.835156708	3.44E-06	0.001420659
ENSMUSG00000085645	Hoxb5os	11:96291024-96306910	0.228742333	0	9.510920778	3.80E-06	0.001548301
ENSMUSG00000026527	Rgs7	1:175059087-175492500	0.864852333	0.314130667	1.585616317	3.87E-06	0.001548301
ENSMUSG00000032060	Cryab	9:50751325-50756636	74.09353633	22.844477	1.671735224	3.99E-06	0.001576218
ENSMUSG00000045875	Adra1a	14:66635251-66771168	0.918553	0.267962333	1.913730246	4.22E-06	0.001646762
ENSMUSG00000041565	L3mbtl4	17:68273797-68780086	0	0.034210667	-11.50191925	4.33E-06	0.001670443
ENSMUSG00000007097	Atp1a2	1:172271709-172298064	103.975456	29.49183967	1.962001481	4.83E-06	0.001806411
ENSMUSG00000087689	Gm15845	16:36359497-36387406	0	0.067626	-9.200343513	5.23E-06	0.001933341
ENSMUSG00000015568	Lpl	8:68880491-68907448	138.147275	48.10353967	1.640439377	5.38E-06	0.001971446
ENSMUSG00000085653	Gm15179	1:75360322-75432318	17.51681933	4.351186667	2.038154149	5.67E-06	0.002035897
ENSMUSG00000101337	Dnah7c	1:46425592-46807476	0.005323667	0	9.222345751	5.83E-06	0.002069273
ENSMUSG00000051596	Otop1	5:38275972-38304217	1.144786333	0.130731667	3.265764881	6.33E-06	0.002169479
ENSMUSG00000040705	A930016O22Rik	7:19411019-19421633	161.8966853	50.83355933	1.796979792	6.50E-06	0.002199619
ENSMUSG00000030727	Rabep2	7:126428759-126463103	121.609764	42.51705567	1.711247608	6.65E-06	0.002226924
ENSMUSG00000028841	Cnksr1	4:134228041-134238399	6.342168667	0.966174667	3.041948296	7.11E-06	0.002361062
ENSMUSG00000020738	Sumo2	11:115523102-115536276	2.522936667	9.395767667	-1.565319893	7.22E-06	0.002373795
ENSMUSG00000063558	Aox1	1:58029931-58106413	4.736598667	1.350809	1.957604778	7.38E-06	0.002377186
ENSMUSG00000048834	Vstm2a	11:16257724-16427310	0.049492333	0	11.19968887	7.43E-06	0.002377186
ENSMUSG00000041014	Nrg3	14:38368952-39473088	0	0.010042667	-11.29385143	8.06E-06	0.002555319
ENSMUSG00000101880	Gm29282	1:155011332-155057179	0.142832	0	11.22770148	8.99E-06	0.002718439

ENSMUSG00000033510	Otud7a	7:63444751-63759028	0.016982333	0	11.2852151	9.03E-06	0.002718439
ENSMUSG00000057280	Musk	4:58285960-58374303	4.279628667	1.307323667	2.040270835	9.34E-06	0.002765726
ENSMUSG00000085348	Myhas	11:67171012-67305975	89.75831767	29.344829	1.682782991	9.65E-06	0.002832189
ENSMUSG00000086354	Gm13938	2:76703984-76982555	15.66602133	4.613550667	1.964894033	1.13E-05	0.003211824
ENSMUSG00000118020	AC118724.1	19:27145316-27192009	0.026913	0	9.218990378	1.14E-05	0.003211824
ENSMUSG00000087268	Gm14486	2:30658258-30678011	0.064440667	0	8.932650045	1.14E-05	0.003211824
ENSMUSG00000041548	Hspb8	5:116408491-116422864	35.04796433	10.53957067	1.856879412	1.16E-05	0.003211824
XLOC_001142	XLOC_001142	1:56368465-56399157	0.200784667	0	11.2642422	1.18E-05	0.003211824
ENSMUSG00000068699	Flnc	6:29433256-29461883	72.148751	17.45787	2.183286383	1.18E-05	0.003211824
ENSMUSG00000039891	Txlnb	10:17796226-17845665	22.43778133	5.680055667	2.103224787	1.21E-05	0.003254772
ENSMUSG00000030739	Myh14	7:44605803-44670843	3.304664333	1.071492	1.963661942	1.28E-05	0.003371226
ENSMUSG00000022032	Scara5	14:65666403-65764826	5.479422667	2.146998667	1.415676727	1.29E-05	0.003371226
ENSMUSG00000050315	Synpo2	3:123076519-123236149	7.363445	2.372589333	1.82580689	1.39E-05	0.003606135
ENSMUSG00000068697	Myoz1	14:20649107-20656540	136.6506147	41.37457433	1.850548667	1.55E-05	0.003864133
XLOC_067665	XLOC_067665	17:45313717-45338018	0.226833667	0	11.12805271	1.58E-05	0.003907873
ENSMUSG00000116272	Gm49540	15:77014093-77015898	22.46242133	5.446345	2.165937843	1.66E-05	0.004064882
ENSMUSG00000031382	Asb11	X:164436994-164459170	21.10465967	4.537274333	2.350509584	1.72E-05	0.004147262
ENSMUSG00000026141	Col19a1	1:24261890-24587472	0.230309667	0.059192667	2.024913524	1.80E-05	0.004302391
ENSMUSG00000104982	Gm32554	5:11165537-11181825	0	0.075046667	-8.861365407	1.82E-05	0.004302391
ENSMUSG00000021798	Ldb3	14:34526603-34588682	70.62265133	27.975754	1.803730304	1.99E-05	0.004664341
ENSMUSG00000030852	Tacc2	7:130577438-130764785	69.098654	19.74370233	2.02833124	2.02E-05	0.004694696
ENSMUSG00000046818	Ddit4l	3:137621612-137628333	16.68814367	5.041585667	1.84466083	2.19E-05	0.004976371
ENSMUSG00000017300	Tnnc2	2:164777161-164779967	268.9599507	85.568226	1.74695724	2.25E-05	0.005094808
ENSMUSG00000034055	Phka1	X:102513975-102644246	22.90780133	7.885369333	1.67872897	2.29E-05	0.00515671
ENSMUSG00000108607	Gm44646	7:44501689-44524670	13.17377367	4.488055	1.669719282	2.35E-05	0.005242953
ENSMUSG00000027210	Meis2	2:115863064-116065839	3.453525333	1.474447	1.431308196	2.44E-05	0.005411098
ENSMUSG00000042010	Acacb	5:114146535-114250761	22.78790133	8.282713	1.648198933	2.45E-05	0.005411098
ENSMUSG00000028396	2310002L09Rik	4:73939371-73950846	13.35393633	2.645057667	2.3119404	2.51E-05	0.005462019
ENSMUSG00000078937	Cpt1b	15:89416405-89425863	33.88259133	11.00355533	1.770066268	2.54E-05	0.005473184
ENSMUSG00000114968	A630019I02Rik	13:93040547-93144604	3.699649333	1.170155667	1.814625363	2.56E-05	0.005473184
ENSMUSG00000071317	Bves	10:45335772-45372479	4.387417333	1.290134	1.804230676	2.62E-05	0.005544545
ENSMUSG00000038763	Alpk3	7:81057600-81105612	18.17028567	5.556834333	1.854571086	2.62E-05	0.005544545
ENSMUSG00000004558	Ndrg2	14:51905271-51914158	152.1909893	46.55600633	1.753979321	2.64E-05	0.005557684
ENSMUSG00000055632	Hmcn2	2:31314415-31460738	11.922692	4.237291667	1.542168163	2.68E-05	0.005576787
ENSMUSG00000022519	Srl	16:4480216-4541816	62.61684167	21.91941067	1.652980705	2.74E-05	0.005597223

ENSMUSG00000074001	Klhl40	9:121777607-121783818	18.46146967	5.061706	2.063525328	2.78E-05	0.005610822
ENSMUSG00000036745	Ttll7	3:146852367-146984009	7.561450333	2.544268333	1.903301677	2.82E-05	0.005661899
ENSMUSG00000031636	Pdlim3	8:45885461-45919548	47.47884733	13.057259	2.04565702	2.90E-05	0.005786495
ENSMUSG00000102717	Gm37759	1:136052534-136119924	3.341764333	1.164523667	1.651451235	2.99E-05	0.005893436
ENSMUSG00000116961	Gm49662	16:36569145-36598497	0	0.098297333	-8.818140454	3.00E-05	0.005893436
ENSMUSG00000028116	Myoz2	3:123006206-123035015	43.08567767	12.127394	1.941596308	3.02E-05	0.005893442
ENSMUSG00000074852	Hpse2	19:42786539-43388355	0.055655333	0	8.836975347	3.07E-05	0.005952474
ENSMUSG00000030111	A2m	6:121635376-121679227	1.341637	4.025117667	-1.749820936	3.18E-05	0.006041539
ENSMUSG00000034457	Eda2r	X:97333840-97377216	2.813781667	0.703842667	2.297834465	3.24E-05	0.006118562
ENSMUSG00000023092	Fhl1	X:56731787-56793346	158.0328063	49.169866	1.83372931	3.30E-05	0.006194082
ENSMUSG00000100197	Gm28638	2:66125317-66175250	0	0.069659	-10.53378712	3.32E-05	0.006214217
XLOC_049898	XLOC_049898	14:70443533-70462067	0.165876333	0	10.48707448	3.40E-05	0.006293255
XLOC_139182	XLOC_139182	8:72231215-72238975	0.05962	2.228833667	-4.727147032	3.45E-05	0.006353583
ENSMUSG00000090185	Gm15523	12:103321142-103352460	4.829644333	1.494902667	2.023387183	3.67E-05	0.006602488
ENSMUSG00000029561	Oasl2	5:114896936-114912234	13.67238467	43.22139233	-1.596465181	3.68E-05	0.006602488
XLOC_067356	XLOC_067356	17:35714814-35731414	0.204160667	0	10.57339575	3.82E-05	0.006780653
ENSMUSG00000035296	Sgcg	14:61219115-61258490	9.708930667	3.079083333	1.736038271	3.84E-05	0.006791535
ENSMUSG00000037139	Myom3	4:135759715-135815564	14.43799533	4.942859	1.734226515	3.98E-05	0.006923274
ENSMUSG00000025432	Avil	10:127000709-127020994	2.432287667	0.373829333	2.747887977	4.08E-05	0.007023323
XLOC_141650	XLOC_141650	8:33141724-33145306	0	0.310241667	-8.871557812	4.34E-05	0.00733766
ENSMUSG00000097003	D930007P13Rik	15:103123070-103146828	0.034154333	0	8.562972073	4.39E-05	0.00738343
ENSMUSG00000021702	Thbs4	13:92751590-92794818	29.42742633	12.52435033	1.336927016	4.41E-05	0.00738343
ENSMUSG00000029096	Htra3	5:35652023-35679782	20.58148	9.214663667	1.192944143	4.43E-05	0.007394847
ENSMUSG00000026208	Des	1:75360329-75368579	186.8652267	59.847842	1.826142056	4.46E-05	0.00739558
ENSMUSG00000085945	2310014F06Rik	7:112612560-112899129	3.347731	0.970667667	1.911597551	4.48E-05	0.00739558
ENSMUSG00000027488	Snta1	2:154376313-154408099	23.78244467	9.079703333	1.499800523	4.60E-05	0.00755846
ENSMUSG00000027737	Slc7a11	3:49892526-50443614	0.40427	1.207677	-1.750908197	4.81E-05	0.007843805
ENSMUSG00000022935	Grik1	16:87895900-88290265	0.019609	0	10.46804212	4.93E-05	0.007980562
ENSMUSG00000041476	Smpx	X:157698910-157752591	28.71469733	8.754653667	1.832991606	5.23E-05	0.008329707
ENSMUSG00000086501	4930597A21Rik	11:44575191-44591343	0.011383667	0.935433333	-5.974543982	5.26E-05	0.00834317
ENSMUSG00000032238	Rora	9:68621970-69388246	10.60289667	4.665072	1.305129777	5.38E-05	0.008488622
ENSMUSG00000107265	Gm15469	5:31116556-31139243	1.947265	0.442206333	2.277967987	5.50E-05	0.008575345
ENSMUSG00000096992	Gm26788	1:34011495-34308577	2.367599667	0.925264667	1.446417959	5.50E-05	0.008575345
ENSMUSG00000087652	Gm15918	14:63602524-63607372	0.160259333	0	8.505780452	5.51E-05	0.008575345
ENSMUSG00000026489	Coq8a	1:180165238-180199602	60.63687367	22.96986533	1.675603528	5.53E-05	0.008575345

ENSMUSG00000024924	Vldlr	19:27216484-27254231	13.98675333	5.337361667	1.531469337	5.82E-05	0.008836533
ENSMUSG00000032661	Oas3	5:120753098-120777661	16.02621333	40.54997367	-1.302275764	6.03E-05	0.009110635
ENSMUSG00000117231	Gm41609	17:70765839-70806059	0.034840667	0	8.877389546	6.25E-05	0.009405158
ENSMUSG00000111610	Gm34829	9:77992599-78002261	0.099988	0	8.68693206	6.29E-05	0.00942107
ENSMUSG00000109224	Tmem147os	7:30734739-30756126	0.002021	0.139611667	-5.872907315	6.43E-05	0.009591264
ENSMUSG00000023019	Gpd1	15:99717515-99725005	43.09779533	19.462747	1.223835413	6.53E-05	0.009627957
ENSMUSG00000093553	Gm20633	3:96238108-96247329	21.29102767	51.08013533	-1.742992902	6.53E-05	0.009627957
ENSMUSG00000021200	Asb2	12:103321142-103356001	41.370956	12.62211933	1.842551988	6.86E-05	0.009991959
ENSMUSG00000072720	Myo18b	5:112688876-112896362	16.206731	5.403289	1.681270986	6.89E-05	0.009991959
ENSMUSG00000114150	Gm46367	12:83792603-83921910	0.315889	1.083573	-2.961067825	7.34E-05	0.010603511
ENSMUSG00000004864	Mapk13	17:28769297-28780233	9.435845667	22.39678233	-1.417506433	7.39E-05	0.010622468
ENSMUSG00000086312	Gm15336	18:38993110-39364031	0.535725667	0.088151667	3.1742238	7.78E-05	0.010943133
ENSMUSG00000032561	Acpp	9:104288251-104337748	7.020166333	16.65451633	-1.254019397	7.81E-05	0.010943133
ENSMUSG00000030786	Itgam	7:128062640-128118491	27.320418	70.73822767	-1.30807917	7.91E-05	0.011011215
ENSMUSG00000036162	Fam219aos	4:41517437-41569535	0.181670667	0	10.14886277	7.98E-05	0.011075233
ENSMUSG00000021898	Asb14	14:26894557-26915258	9.752807667	3.101149	1.837292873	8.04E-05	0.011088776
ENSMUSG00000072902	Gm10435	11:58993838-59139257	8.358708667	2.915023667	1.759308534	8.25E-05	0.011296532
ENSMUSG00000031097	Tnni2	7:142441808-142444410	306.1263427	98.055757	1.679908955	8.27E-05	0.011296532
ENSMUSG00000034361	Cpne2	8:94532990-94570531	16.39199233	38.43125467	-1.181204142	8.42E-05	0.011306523
ENSMUSG00000001333	Sync	4:129287617-129308559	5.592449667	1.743071	1.785371176	8.44E-05	0.011306523
ENSMUSG00000043639	Rbm20	19:53677306-53867080	4.727131	1.570390667	1.907032235	8.63E-05	0.011416169
ENSMUSG00000028931	Kcnab2	4:152390742-152477910	8.384676	21.82404633	-1.41948083	8.73E-05	0.011416169
ENSMUSG00000037942	Crp	1:172698055-172833031	0.034391	0	10.07227648	8.74E-05	0.011416169
ENSMUSG00000038170	Pde4dip	3:97689263-97888707	153.0351207	51.808444	1.563159719	8.77E-05	0.011416169
ENSMUSG00000056973	Ces1d	8:93166068-93197838	19.7741	8.702083333	1.407616531	8.77E-05	0.011416169
ENSMUSG00000021373	Cap2	13:46501848-46650281	13.86539433	4.702941667	1.646862646	9.00E-05	0.01156826
ENSMUSG00000030399	Ckm	7:19404776-19422841	549.1016643	189.8165307	1.641998841	9.02E-05	0.01156826
ENSMUSG00000074264	Amy1	3:113555710-113606699	8.841471333	2.944392	1.623560759	9.14E-05	0.011598922
ENSMUSG00000107296	Gm43500	5:135090004-135138493	0.923059	0.225668	2.136984791	9.42E-05	0.011780071
ENSMUSG00000027999	Pla2g12a	3:129878606-129895825	14.469456	4.810811667	1.758985443	9.83E-05	0.012213408
XLOC_141003	XLOC_141003	8:8959708-8969766	0.261471667	0	10.09306458	9.97E-05	0.012344068
ENSMUSG00000018566	Slc2a4	11:69942539-69948188	48.372563	17.25406733	1.581594082	0.000100645	0.012399566
ENSMUSG00000118370	AC124430.1	18:62142618-62146508	0.188981	0	8.448087962	0.000100889	0.012399566
ENSMUSG00000110534	Gm45708	8:93565977-93628624	0.036065667	0	10.00778418	0.000101306	0.012407834
XLOC_054886	XLOC_054886	15:48842102-48887572	0	0.561188667	-13.37853311	0.000103137	0.012548513

XLOC_135561	XLOC_135561	7:100432711-100436150	0.36094	0	9.021381265	0.000104133	0.012601053
ENSMUSG00000114796	A930028N01Rik	19:40292041-40513752	1.372514333	0.771467667	1.32901361	0.000104302	0.012601053
ENSMUSG00000049265	Kcnk3	5:30588170-30625271	2.851214667	1.003923	1.464464951	0.00010546	0.012696173
ENSMUSG00000052374	Actn2	13:12269426-12340760	100.081543	33.445886	1.69645987	0.000106162	0.012696173
ENSMUSG00000035948	Acss3	10:106933517-107123668	3.437982	1.284594333	1.673627764	0.000111027	0.013189217
ENSMUSG00000041710	Trpc5	X:144381671-144688180	0	0.010734333	-10.15399799	0.000113685	0.013441439
ENSMUSG00000032496	Ltf	9:111019271-111042767	562.767202	1336.0837	-1.230858878	0.000114286	0.013441439
XLOC_025776	XLOC_025776	11:29500337-29502486	0	0.346599667	-8.304314574	0.000117694	0.013706101
ENSMUSG00000084876	Gm14965	19:6384283-6418606	15.34091033	7.058050333	1.3795469	0.000121748	0.014082514
ENSMUSG00000028278	Rragd	4:32983037-33022180	10.63802667	4.24705	1.470822086	0.000123999	0.014253433
ENSMUSG00000019787	Trdn	10:33080554-33476709	30.292664	13.34210133	1.627714024	0.000125214	0.014346738
XLOC_159384	XLOC_159384	X:97827562-97923336	0.179034333	0	12.71873905	0.000130328	0.014742433
ENSMUSG00000110301	Gm35363	7:101525376-101538330	0.050324	0	8.14466408	0.00013273	0.014966441
ENSMUSG00000113935	Gm35732	13:31971086-31989599	0.126709667	0	9.82719218	0.000133177	0.014969323
ENSMUSG00000027750	Postn	3:54361109-54391037	56.14352533	27.91271433	1.084766986	0.000134874	0.015112322
ENSMUSG00000032648	Pygm	19:6384399-6398459	239.42483	84.82191733	1.625865502	0.000137572	0.015311957
ENSMUSG00000031633	Slc25a4	8:46206797-46211284	216.160151	87.32876067	1.437497172	0.000139038	0.015311957
ENSMUSG00000041801	Phlda3	1:135766119-135769136	32.92867033	9.681618	1.928298464	0.000139266	0.015311957
ENSMUSG00000033880	Lgals3bp	11:118392751-118402092	20.524798	32.26237167	-1.328503489	0.000139905	0.015311957
ENSMUSG00000076441	Ass1	2:31470207-31520672	5.410749333	12.09943767	-1.171591615	0.000139973	0.015311957
ENSMUSG00000027438	Napb	2:148693864-148732467	2.095578667	0.483194	2.070094712	0.000139981	0.015311957
ENSMUSG00000109773	Gm34474	8:35218638-35222071	0.181302333	0	8.084232642	0.000140452	0.015311957
ENSMUSG00000085899	Gm15338	7:124186718-124290270	0	0.022849667	-9.946384042	0.000141092	0.015311957
ENSMUSG00000087621	1700003G18Rik	7:116081759-116093159	0.093636667	0	8.131661517	0.000141331	0.015311957
ENSMUSG00000001027	Scn4a	11:106318592-106353288	17.96371433	5.520239667	1.647943064	0.000143206	0.015338296
ENSMUSG00000005373	Mlxipl	5:135089890-135138382	9.625981667	3.305978667	1.745445989	0.000145544	0.015524244
ENSMUSG00000049422	Chchd10	10:75933130-75937747	79.63616467	37.43741233	1.265167335	0.00014648	0.015532935
ENSMUSG00000018411	Mapt	11:104231390-104332090	5.770558333	1.612134333	1.793082786	0.0001465	0.015532935
ENSMUSG00000101636	4930579H20Rik	10:27264942-27616869	0.776480667	0.163727667	2.337680928	0.000147368	0.015578452
ENSMUSG00000099906	Gm28653	1:20669882-20684298	3.127373667	0.303368667	3.443606302	0.000147913	0.015589673
ENSMUSG00000104886	Gm43000	5:15934412-16371865	1.073635	0.414648667	1.494376531	0.000148397	0.01559364
ENSMUSG00000025317	Car5a	8:121916126-121944904	0	0.027537667	-8.308648961	0.000148829	0.01559364
ENSMUSG00000044716	Dok7	5:35056766-35087839	2.612186333	0.87084	1.669021997	0.000152616	0.015914885
ENSMUSG00000060961	Slc4a4	5:88886818-89239653	5.153161333	1.883975333	1.777719937	0.000156685	0.016225371
ENSMUSG00000085479	9430073C21Rik	11:67171012-67199234	24.593325	9.063670667	1.551025455	0.000157641	0.016276926

ENSMUSG00000061462	Obscn	11:58994256-59139170	101.8510477	31.07688833	1.706036077	0.000158451	0.016313141
ENSMUSG00000051747	Ttn	2:76703980-76982547	343.650126	118.7687533	1.700818511	0.000160873	0.016514539
ENSMUSG00000025900	Rp1	1:3999557-4409241	0.304899	0.094083667	1.828009221	0.000163135	0.016698512
ENSMUSG00000028631	Kcnq4	4:120696138-120748612	1.766877333	0.572317333	1.752028452	0.000163662	0.016704367
ENSMUSG00000057715	A830018L16Rik	1:11414105-11975901	0.088356	0.010705667	2.619995095	0.000166277	0.016805404
ENSMUSG00000052212	Cd177	7:24743983-24760311	130.4787243	326.9420317	-1.273610453	0.000168911	0.016871168
ENSMUSG00000021573	Tppp	13:74009407-74035753	3.967301	1.525942667	1.453837753	0.000169689	0.016871168
ENSMUSG00000040265	Dnm3	1:161982453-162478034	2.090249667	0.679061333	1.919164874	0.000169876	0.016871168
ENSMUSG00000041828	Abca8a	11:110025634-110095978	16.12976967	6.279005	1.284260126	0.000169965	0.016871168
ENSMUSG00000097193	Gm26664	8:84665128-84669406	0	0.481096333	-9.783844057	0.000172239	0.016993781
ENSMUSG00000020407	Upp1	11:9118103-9136170	4.841629	14.229752	-1.535705492	0.000177294	0.017424853
ENSMUSG00000040694	Apobec2	17:48419231-48432930	76.709516	26.890349	1.557676718	0.000177589	0.017424853
ENSMUSG00000022548	Apod	16:31296192-31314808	8.450609333	3.279588	1.382515419	0.000180393	0.017602662
XLOC_088082	XLOC_088082	2:136535563-136624094	0.124265	0	12.42697068	0.000181051	0.017618494
XLOC_142238	XLOC_142238	8:55353318-55378548	5.074737333	0.707924	2.93130953	0.000181728	0.017636092
ENSMUSG00000071714	Csf2rb2	15:78282507-78305721	5.497619333	12.510972	-1.161018493	0.000182266	0.017640099
ENSMUSG00000106245	Gm43824	3:128610987-128887161	0.423075	0.004967667	5.957836798	0.000183385	0.017652203
ENSMUSG00000054391	4930517O19Rik	14:100213142-100245339	0.073113667	0	9.864844187	0.000183988	0.017658768
ENSMUSG00000031461	Myom2	8:15057653-15133541	58.70268133	19.16567567	1.75044397	0.000184448	0.017658768
ENSMUSG00000021838	Samd4	14:46882854-47105815	10.04126833	4.186450667	1.249010717	0.000188779	0.017928491
ENSMUSG00000034898	Filip1	9:79815051-80012851	6.628142667	2.23492	1.570140513	0.000189694	0.017967272
ENSMUSG00000090427	Gm17225	3:126432883-126439269	0.107954	0	8.208542202	0.000195214	0.01841347
ENSMUSG00000036854	Hspb6	7:30552178-30555443	73.758043	20.46632333	1.98765052	0.000195441	0.01841347
ENSMUSG00000027574	Nkain4	2:180934772-180954699	0.155827333	0	9.644625981	0.000200174	0.018766373
ENSMUSG00000041624	Gucy1a2	9:3532778-3894736	1.813562333	0.817479	1.239185995	0.000200244	0.018766373
ENSMUSG00000019194	Scn1b	7:31116524-31127003	40.567379	17.01504567	1.401362576	0.000202458	0.018874279
ENSMUSG00000117727	AC131675.1	18:35069325-35087623	0.092192333	0	9.639157396	0.000205271	0.018936714
ENSMUSG00000031274	Col4a5	X:141475385-141689234	0.933474	0.429327	1.119799151	0.000205334	0.018936714
ENSMUSG00000018845	Unc45b	11:82910550-82943403	12.56863533	5.00904	1.546268004	0.000206082	0.018936714
XLOC_074422	XLOC_074422	19:6922224-6925167	0.216305667	0	8.22885537	0.000206417	0.018936714
ENSMUSG00000106515	Gm30382	3:149284418-149445301	0.022853667	0	9.653201175	0.000206596	0.018936714
ENSMUSG00000101168	Gm28892	1:134754744-134955938	0.984474	0.432661333	1.302909151	0.000206793	0.018936714
XLOC_075511	XLOC_075511	19:38420369-38430883	0.186107	0	9.913936141	0.00020686	0.018936714
ENSMUSG00000025348	Itga7	10:128933818-128958282	12.466748	4.702972333	1.491513362	0.000209855	0.019161587
ENSMUSG00000086249	Gm12724	4:106241216-106290026	0.038995333	0	9.873855037	0.000210492	0.019170467

ENSMUSG00000024211	Grm8	6:27275119-28135178	0.296056	0.055428333	2.380618266	0.000212695	0.019272189
ENSMUSG00000044951	Mylk4	13:32700834-32784017	24.09706933	8.397081333	1.635696557	0.000214294	0.019367731
ENSMUSG00000033196	Myh2	11:67171027-67197517	198.5896427	72.487207	1.561660689	0.000215494	0.019426722
ENSMUSG00000069601	Ank3	10:69398773-70027438	17.331575	7.136751	1.278413603	0.000219731	0.019684836
ENSMUSG00000038967	Pdk2	11:95026258-95041354	33.51601867	13.23581967	1.428819254	0.00022002	0.019684836
ENSMUSG00000026430	Rassf5	1:131176410-131245258	13.07894567	37.21209067	-1.113885063	0.000223611	0.019955858
ENSMUSG00000057101	Zfp180	7:24081924-24107713	2.849505	5.522324667	-1.88927264	0.000229172	0.020349902
ENSMUSG00000032369	Plscr1	9:92249750-92272278	10.31531367	22.977451	-1.265466757	0.000230655	0.020430522
ENSMUSG00000028177	1810013D15Rik	3:157925220-157938355	0.103312	0	8.224988374	0.000231657	0.020431625
ENSMUSG00000085931	Gm12648	4:94089576-94425588	0	0.038838333	-12.41103808	0.000234269	0.020596555
ENSMUSG00000040314	Ctsg	14:56099881-56102574	87.332424	223.75297	-1.294978081	0.000236614	0.02074732
ENSMUSG00000026950	Neb	2:52136647-52378474	120.4892297	43.30160133	1.626966541	0.000237152	0.02074732
ENSMUSG00000020061	Mybpc1	10:88518279-88605152	102.164487	39.10800867	1.539104021	0.000239112	0.020867412
ENSMUSG00000057897	Camk2b	11:5969644-6066362	7.703621	3.658906333	1.401704947	0.000241391	0.021014602
ENSMUSG00000028427	Aqp7	4:41033074-41048139	8.392672	3.309085333	1.43368036	0.000243658	0.02105712
ENSMUSG00000042724	Map3k9	12:81721010-81781175	7.292881	15.494005	-1.050730714	0.000244978	0.021119864
ENSMUSG00000006369	Fbln1	15:85205949-85286535	5.028614333	2.410949667	1.074457924	0.000247103	0.02120016
XLOC_100810	XLOC_100810	4:64572023-64693682	0	0.096096	-12.23974913	0.000247973	0.021223465
ENSMUSG00000021768	Dusp13	14:21733394-21797832	10.987377	3.229443667	1.516992539	0.000249312	0.021286833
ENSMUSG00000024059	Clip4	17:71768473-71864273	8.778207333	3.153622	1.775221394	0.000260362	0.022123935
XLOC_078968	XLOC_078968	2:19415396-19423529	0	0.224647333	-9.59666637	0.00026124	0.022145566
ENSMUSG00000102590	Mannr	3:29891014-29924191	0.053859	0	9.755858479	0.00026197	0.022154588
XLOC_012407	XLOC_012407	10:35313722-35323974	0.164947333	0	9.696211964	0.000271174	0.022878495
ENSMUSG00000043542	Zc2hc1a	3:7503483-7553836	2.945135	1.375292333	1.164466815	0.000274669	0.023040015
ENSMUSG00000045667	Smtnl2	11:72389164-72411713	23.29341733	7.530087	1.574104406	0.0002758	0.023049706
XLOC_122930	XLOC_122930	6:26752346-26766756	0.138603	0	9.752464533	0.000279414	0.02329697
ENSMUSG00000031400	G6pdx	X:74409483-74429194	50.308188	119.9660367	-1.232252915	0.000282246	0.023344715
ENSMUSG00000025938	Slco5a1	1:12866549-12992650	3.520324333	1.34312	1.503044016	0.000282534	0.023344715
ENSMUSG00000021536	Adcy2	13:68620043-68999541	5.322920667	2.569238	1.235093667	0.000283965	0.023401771
ENSMUSG00000027513	Pck1	2:173153048-173159273	50.01887933	21.05929	1.382226087	0.000289981	0.023787115
ENSMUSG00000047246	Hist1h2be	13:23551258-23698454	48.89843367	205.4671783	-2.503947655	0.000291795	0.023880817
ENSMUSG00000066842	Hmcn1	1:150562524-150993435	1.224789333	0.588696667	1.040055997	0.000295249	0.024052596
ENSMUSG00000021238	Aldh6a1	12:84430717-84451004	21.78528433	8.310752	1.509590606	0.000303927	0.024646503
ENSMUSG00000017412	Cacnb4	2:52428320-52676831	0.116787	0.025157333	1.9900196	0.000307205	0.024764264
ENSMUSG00000105891	A230001M10Rik	3:102262405-102445132	0.012319	0	9.477106964	0.000311126	0.025001985

ENSMUSG00000086053	Gm15178	1:75375259-75432314	0.296873667	0	11.81143573	0.000314678	0.025173558
ENSMUSG00000045975	C2cd2	16:97855209-97962621	12.17309367	6.883272	1.251088509	0.000316943	0.025247247
ENSMUSG00000078566	Bnip3	7:138890836-138909519	55.29247233	22.05170067	1.471723767	0.000317021	0.025247247
ENSMUSG00000071713	Csf2rb	15:78325752-78353847	15.827642	36.73140733	-1.166021087	0.000318008	0.025269175
XLOC_025570	XLOC_025570	11:22534887-22536774	0.759288	0	9.402826986	0.000320053	0.025374906
ENSMUSG00000018428	Akap1	11:88830792-88864586	14.49712633	6.297957	1.297161797	0.000328783	0.026009011
ENSMUSG00000028464	Tpm2	4:43514711-43523765	167.1576463	67.44248467	1.425485944	0.000332486	0.026243516
ENSMUSG00000026109	Tmeff2	1:50900647-51187270	0.409384	0.143916	1.594165813	0.000333929	0.026298922
ENSMUSG00000032845	Alpk2	18:65265529-65394066	6.670462	2.658362	1.428275525	0.000336276	0.026425223
ENSMUSG00000071604	Fam189a2	19:23972750-24031019	4.144912333	1.853345	1.280839063	0.000342523	0.026797544
ENSMUSG00000033182	Kbtbd12	6:88545114-88637950	2.827034667	1.223472	1.727429114	0.000344898	0.026924022
ENSMUSG00000026407	Cacna1s	1:136052750-136119822	36.74997733	14.14678767	1.5023334	0.000347481	0.027066156
ENSMUSG00000041731	Pgm5	19:24683016-24861855	5.092443	2.253198333	1.36123594	0.000351243	0.027299344
ENSMUSG00000026131	Dst	1:33908225-34308661	57.579304	23.24246067	1.303941089	0.000354487	0.027431402
ENSMUSG00000057729	Prtn3	10:79874471-79883174	145.4010493	343.580073	-1.193062486	0.000355261	0.02743159
ENSMUSG00000022053	Ebf2	14:67233291-67430918	2.147977667	1.032202	1.25611302	0.000356499	0.027467444
XLOC_094921	XLOC_094921	3:21437743-21489557	0	0.165181	-11.85642217	0.000358232	0.027476489
ENSMUSG00000071540	3425401B19Rik	14:32659119-32685293	17.168022	6.404158333	1.560106726	0.000358937	0.027476489
ENSMUSG00000020354	Sgcd	11:46896253-47989377	11.41305467	5.104836333	1.363620094	0.000369861	0.028071126
ENSMUSG00000035522	Tsga8	X:82948902-83955069	0.783297333	0.235793333	1.860147611	0.000369866	0.028071126
ENSMUSG00000097286	Gm26684	15:54887569-54964642	0.828667667	0.227216667	2.222639502	0.000377689	0.028542809
ENSMUSG00000025473	Adam8	7:139978932-139992562	22.52208933	47.72244667	-1.034653982	0.000384597	0.028941773
ENSMUSG00000005628	Tmod4	3:95124476-95129209	31.68738667	12.417381	1.505500573	0.000386292	0.028965094
ENSMUSG00000042686	Jph1	1:16964560-17097889	14.62262667	5.867948667	1.446520514	0.000387305	0.028965094
ENSMUSG00000087203	Gm13986	2:117857219-118111202	0.46207	0.125030333	2.958221155	0.000388169	0.028965094
ENSMUSG00000037709	Fam13a	6:58932090-59024549	5.399931	2.036291333	1.459695724	0.000390911	0.029108499
ENSMUSG00000027961	Lrrc39	3:116562973-116583134	4.784914333	1.782126333	1.534127857	0.000398378	0.029479128
ENSMUSG00000045103	Dmd	X:82948870-85206141	20.30047	7.071697667	1.612634582	0.000399903	0.029530434
ENSMUSG00000062515	Fabp4	3:10204088-10208576	233.547007	108.6795017	1.144797942	0.000401786	0.029607993
ENSMUSG00000030672	Mylpf	7:127208890-127214298	513.0169477	207.4863053	1.398820012	0.000408511	0.03001753
ENSMUSG00000112810	Gm35101	10:99669514-99675623	0.259365333	0	9.617378928	0.000409034	0.03001753
ENSMUSG00000003477	Inmt	6:55170626-55175043	4.333841333	0.550826667	3.103861814	0.000409901	0.030019097
XLOC_101540	XLOC_101540	4:101676427-101716848	0.188112667	0	11.54618846	0.000413721	0.03013674
ENSMUSG00000041827	Oasl1	5:114923240-114937915	1.911931	7.181055	-1.650906507	0.000414052	0.03013674
XLOC_014408	XLOC_014408	10:89396205-89473954	0	0.103352667	-11.69928055	0.00041824	0.030252433

ENSMUSG00000066705	Fxyd6	9:45370185-45396159	16.31167533	7.646414667	1.205788339	0.000418709	0.030252433
ENSMUSG00000062908	Acadm	3:153922357-153944632	38.77472067	19.04352833	1.150789328	0.000419049	0.030252433
ENSMUSG00000117222	Gm49906	17:50339252-50399172	0.893049667	0.187139667	2.844691937	0.000422783	0.030337
ENSMUSG00000017697	Ada	2:163726584-163750239	4.407146	10.046142	-1.195033906	0.000427311	0.030493532
ENSMUSG00000098008	A930001A20Rik	3:14971201-15002727	0.047389	0	9.390666999	0.000430567	0.03058902
ENSMUSG00000021123	Rdh12	12:79208914-79222665	8.079787	16.60349833	-1.052551179	0.000431462	0.03058902
ENSMUSG00000112230	Ifngas1	10:118502035-118556525	0	0.028250333	-9.329219335	0.000434107	0.030715248
ENSMUSG00000030921	Trim30a	7:104409025-104465193	14.818572	33.326791	-1.137100924	0.000436308	0.030760569
XLOC_038985	XLOC_038985	13:56678646-56679764	0.401915333	0	7.661007058	0.00043648	0.030760569
XLOC_070043	XLOC_070043	18:25962950-25999134	0.162391	0	11.52251311	0.000440337	0.030970972
ENSMUSG00000038034	Igsf8	1:172261641-172319841	16.14294833	9.157732	1.440194592	0.000441513	0.030971544
XLOC_082853	XLOC_082853	2:149319511-149329629	0	0.150290333	-9.311583187	0.000443472	0.030971544
ENSMUSG00000109017	Gm38979	7:29005221-29030250	6.719031	2.703670333	1.48351933	0.000444327	0.030971544
ENSMUSG00000058297	Spock2	10:60106219-60135198	2.733132	0.933798	1.589851684	0.000448888	0.031201664
ENSMUSG00000048416	Mlf1	3:67374097-67400003	25.02549367	9.78983	1.460516796	0.000451904	0.031349974
ENSMUSG00000027716	Trpc3	3:36620482-36690167	2.058509667	0.721619	1.467510353	0.000453832	0.031422368
ENSMUSG00000025006	Sorbs1	19:40294753-40513779	30.538973	14.883424	1.124999146	0.000455345	0.031465777
ENSMUSG00000070424	Art5	7:102096879-102111145	7.979681	1.740774667	2.376239679	0.000459248	0.03167381
ENSMUSG00000026207	Speg	1:75375297-75432320	15.30555033	6.931964	1.458542917	0.000464631	0.031983032
ENSMUSG00000090053	Palm2	4:57434247-57712016	0.714176667	0.203217667	2.404900301	0.000467808	0.032077392
ENSMUSG00000026817	Ak1	2:32621758-32635058	86.99600467	37.70889633	1.315541992	0.000469768	0.032084167
XLOC_060311	XLOC_060311	16:15004988-15058156	0	0.147856	-11.6801995	0.000470617	0.032084167
ENSMUSG00000027792	Bche	3:73635808-73708415	2.376174667	1.042098	1.430188961	0.000472729	0.032095842
ENSMUSG00000001025	S100a6	3:90612882-90624181	108.0871227	244.2053883	-1.103938514	0.00047291	0.032095842
XLOC_147929	XLOC_147929	9:96385428-96396965	0	0.143666667	-9.439320515	0.000477317	0.03214441
ENSMUSG00000009350	Mpo	11:87793581-87804413	258.0370077	598.678477	-1.131711281	0.000477805	0.03214441
ENSMUSG00000030592	Ryr1	7:29003344-29125179	79.72203033	28.281754	1.442501797	0.000477836	0.03214441
ENSMUSG00000068794	Col28a1	6:7997808-8192617	0.847944333	0.379660667	1.176807371	0.000481262	0.032313722
ENSMUSG00000001604	Tcea3	4:136247729-136274898	14.61494367	5.463129333	1.527178617	0.000482978	0.032367705
ENSMUSG00000018574	Acadvl	11:70010183-70015411	57.39598833	30.03398067	1.267213896	0.000489723	0.032696371
ENSMUSG00000044574	5031434C07Rik	6:112273684-112330497	4.205191333	0.94228	2.270589123	0.000502542	0.033317085
ENSMUSG00000031709	Tbc1d9	8:83165352-83272934	1.0703	2.214696	-0.983215415	0.000502772	0.033317085
ENSMUSG00000114134	Gm47408	13:10700161-10830778	0.046309	0	11.36223159	0.000510615	0.033633668
ENSMUSG00000042082	Arsb	13:93771630-93943016	17.59781933	36.81313067	-1.039871916	0.000510647	0.033633668
ENSMUSG00000024049	Myom1	17:70994291-71126856	63.338894	27.95504333	1.358832727	0.000511337	0.033633668

ENSMUSG00000033377	Palmd	3:116918258-116968987	4.792548333	1.805654333	1.572728161	0.000512964	0.033678337
ENSMUSG00000060600	Eno3	11:70657202-70662513	327.9491677	136.237633	1.380079418	0.000516021	0.033700006
ENSMUSG00000055493	Epm2a	10:11343404-11459644	6.341632	3.028804	1.174426575	0.000516141	0.033700006
ENSMUSG00000036030	Prtg	9:72806874-72917291	0.355638	0.112172667	1.723529991	0.000517917	0.033753933
XLOC_146684	XLOC_146684	9:63093399-63099161	0.21575	0	9.142370266	0.000525881	0.034085344
XLOC_054787	XLOC_054787	15:41767460-41775587	0	0.190359667	-9.341080541	0.000530063	0.034293794
ENSMUSG00000027360	Hdc	2:126593667-126619299	31.49973433	67.77786167	-1.061107111	0.000533119	0.034428849
ENSMUSG00000055737	Ghr	15:3317760-3583492	26.93319	13.67764933	1.115736581	0.000541705	0.034856573
ENSMUSG00000111894	Gm47843	10:23573020-23609431	0	0.044060667	-9.401244442	0.000547137	0.035101501
ENSMUSG00000087264	Gad1os	2:70489940-70563357	0	0.029676333	-9.382930721	0.000547488	0.035101501
XLOC_157667	XLOC_157667	X:15981214-16028775	0.105347667	0	11.29346095	0.000553213	0.035340955
XLOC_140383	XLOC_140383	8:111223081-111259206	0	0.180986667	-11.40199469	0.000562258	0.035716797
XLOC_067694	XLOC_067694	17:45700029-45707217	0.183897667	0	9.130421376	0.000562783	0.035716797
ENSMUSG00000006498	Ptbp1	10:79854427-79864771	42.59977567	80.527738	-0.950953966	0.000574306	0.036277908
ENSMUSG00000025330	Padi4	4:140745865-140774236	38.28759633	72.73608533	-0.996120393	0.000576651	0.036277908
ENSMUSG00000062329	Cytl1	5:37735519-37739820	127.101593	62.30597933	1.072870576	0.000578721	0.036277908
ENSMUSG00000028150	Rorc	3:94372794-94398276	9.009652	2.683952333	1.866106399	0.000579046	0.036277908
ENSMUSG00000037940	Inpp4b	8:81342556-82127914	6.782241333	3.650341333	1.009522441	0.000579115	0.036277908
ENSMUSG00000024597	Slc12a2	18:57878678-57946821	11.08163	5.318613667	1.157732506	0.000586155	0.036654296
ENSMUSG00000017491	Rarb	14:16430839-16819156	1.622786333	0.857485333	1.231367603	0.000596238	0.037219297
XLOC_028053	XLOC_028053	11:87553081-87579007	0.183439333	0	11.21717708	0.00059955	0.037360407
ENSMUSG00000037071	Scd1	19:44394455-44407879	252.888321	120.9531047	1.109439381	0.000601152	0.037394614
ENSMUSG00000022584	Ly6c2	15:75045013-75111970	141.982862	342.4724633	-1.193462344	0.000611981	0.037752322
ENSMUSG00000096146	Kcnj11	7:46093953-46100764	6.964751667	2.262683667	1.822203395	0.000612876	0.037752322
ENSMUSG00000039914	Coq10a	10:128337754-128368997	26.95832633	10.696035	1.272413879	0.00061328	0.037752322
ENSMUSG00000058975	Kcnc1	7:46396497-46438704	5.046232	1.925001	1.531023079	0.000623243	0.038233134
ENSMUSG00000035357	Pdzrn3	6:101149609-101377897	5.38385	2.454672667	1.232723448	0.000630975	0.03857418
XLOC_134212	XLOC_134212	7:51803863-51808709	0.243085333	0	9.01261078	0.000632997	0.038631329
ENSMUSG00000046598	Bdh1	16:31422280-31458901	5.083396333	2.074348333	1.628858799	0.000638707	0.03884628
XLOC_038089	XLOC_038089	13:32484995-32530077	0.116571	0	11.16019384	0.000642361	0.038933531
ENSMUSG00000085872	Gm11505	11:87793145-87804479	27.74670467	72.04148	-1.064763991	0.00064343	0.038933531
ENSMUSG00000038522	Mfsd4b1	10:40001575-40025268	1.167587	0.281468	2.181269099	0.000655651	0.039471273
ENSMUSG00000098739	Gm27151	19:22438520-22448865	0.093036333	0	7.453857474	0.000657571	0.039504274
XLOC_068153	XLOC_068153	17:56988235-56990166	0	0.224199	-7.55207858	0.000673351	0.040196256
ENSMUSG00000111271	Gm48127	9:40342308-40342938	0	0.684761333	-7.515285892	0.0006853	0.040704295

ENSMUSG00000019929	Dcn	10:97479609-97518143	176.1184797	97.083201	0.914582358	0.000687962	0.040726376
ENSMUSG00000062980	Cped1	6:21985916-22256404	10.33629967	5.221707667	1.097778698	0.000689051	0.040726376
XLOC_059723	XLOC_059723	16:92222254-92254039	0.156973	0	11.08407756	0.000695211	0.040950586
ENSMUSG00000022860	Chodl	16:78930948-78951733	3.827541	1.357737	1.654517895	0.000699372	0.041118192
ENSMUSG00000043286	Pnpla1	17:28858411-28890309	0.437104667	1.318056667	-1.512839324	0.000702618	0.041118192
ENSMUSG00000087306	A230004M16Rik	11:41710342-41973117	0.176706333	0	9.029647631	0.000702687	0.041118192
ENSMUSG00000024726	Carnmt1	19:18670764-18707200	8.138223667	4.025698667	1.053360112	0.000704949	0.041182711
ENSMUSG00000043705	Capn13	17:73306464-73400866	0	0.024847667	-9.058010037	0.000711894	0.041338036
ENSMUSG00000045761	Togaram2	17:71673261-71729669	1.445457667	0.353994333	2.040948552	0.000712263	0.041338036
ENSMUSG00000006764	Tph2	10:115078641-115185022	0	0.012753	-9.009695923	0.000717392	0.041513589
ENSMUSG00000109689	Gm45646	8:26745929-26760561	0	0.137292333	-8.999626971	0.000718794	0.041513589
ENSMUSG00000052396	Mogat2	7:99219084-99238619	3.745836667	8.21243	-1.093543002	0.000722056	0.041634244
ENSMUSG00000030862	Cpxm2	7:132032687-132154739	3.324055667	1.657565667	1.038200629	0.000728594	0.041875302
ENSMUSG00000038257	Glra3	8:55940460-56130070	0	0.009331333	-9.034016457	0.000731738	0.041904996
ENSMUSG00000102069	1700012I11Rik	15:67226769-67377094	0.04225	0	11.02918663	0.00073775	0.041904996
XLOC_115932	XLOC_115932	5:112123805-112154913	0	0.184179333	-11.22461807	0.000740909	0.041904996
ENSMUSG00000043460	Elfn2	15:78667331-78718771	0	0.015726333	-7.408663309	0.000744029	0.04201458
ENSMUSG00000038239	Hrc	7:45335290-45338974	27.80247	10.44295833	1.514691791	0.00074727	0.042071109
ENSMUSG00000090066	1110002E22Rik	3:138065052-138081506	9.775876667	3.765057667	1.462820405	0.00074856	0.042071109
ENSMUSG00000022562	Oplah	15:76296601-76331104	7.874615333	3.65141	1.304198996	0.000748584	0.042071109
ENSMUSG00000025920	Stau2	1:16228674-16520112	5.840254667	2.831826	1.089382423	0.000752197	0.042173597
ENSMUSG00000003644	Rps6ka1	4:133847290-133887797	44.684355	89.887067	-1.025134419	0.000752782	0.042173597
ENSMUSG00000061723	Tnnt3	7:142498836-142516009	435.158905	168.8821693	1.501864358	0.000754556	0.042206396
ENSMUSG00000028223	Decr1	4:15917240-15945507	16.83153	8.456658333	1.169617844	0.000761526	0.042365181
XLOC_080635	XLOC_080635	2:72554811-72565244	0	0.137358667	-9.281654794	0.000762165	0.042365181
ENSMUSG00000028441	1110017D15Rik	4:41505009-41517333	0.036376333	0	7.333719925	0.000764001	0.042400884
ENSMUSG00000022383	Ppara	15:85734983-85802819	5.372448333	1.821335333	1.538709947	0.000769288	0.042602975
ENSMUSG00000097364	Gm26719	6:21985908-22234659	1.022065667	0.354123333	1.180588317	0.000770891	0.042602975
ENSMUSG00000038045	Sult6b1	17:78883938-78906992	0	0.053587667	-9.00933516	0.000771241	0.042602975
ENSMUSG00000031782	Coq9	8:94838321-94854895	38.02780733	18.040166	1.205086716	0.000793765	0.043508876
ENSMUSG00000025479	Cyp2e1	7:140763739-140774987	45.66080533	12.48918567	2.004675728	0.000803647	0.043914995
ENSMUSG00000040505	Abcg5	17:84658234-84683011	0.090171667	0	7.533129704	0.000806412	0.043949796
ENSMUSG00000113295	Gm49357	13:61655303-61743770	0.044186667	0	10.93490452	0.000815835	0.044313494
ENSMUSG00000027199	Gatm	2:122594467-122611303	14.46357967	29.96142767	-0.981937791	0.000819254	0.044416898
ENSMUSG00000027514	Zbp1	2:173206612-173218923	3.612647667	12.33682867	-1.526393032	0.000820335	0.044416898

ENSMUSG00000015889	Lta4h	10:93453411-93484875	89.46436567	179.4684703	-0.974248513	0.000821796	0.044425562
ENSMUSG00000030401	Rtn2	7:19282624-19296160	47.839971	19.373185	1.416876302	0.000822997	0.044425562
ENSMUSG00000021957	Tkt	14:30548359-30574720	120.5112763	231.622157	-1.019366207	0.000837719	0.045015032
ENSMUSG00000005360	Slc1a3	15:8634124-8710764	5.795611333	2.582436333	1.181019051	0.000856552	0.04595751
ENSMUSG00000006457	Actn3	19:4861216-4877909	142.1934997	57.80533467	1.412479982	0.000866124	0.04640098
ENSMUSG00000073600	Prob1	18:35650351-35655238	7.975731667	2.455229667	1.85483156	0.000869548	0.046405758
ENSMUSG00000031519	Asb5	8:54520454-54587842	24.45012	6.823950667	1.463434304	0.000869928	0.046405758
ENSMUSG00000022876	Samsn1	16:75858793-76022281	14.380611	32.63544933	-1.211259362	0.000870687	0.046405758
ENSMUSG00000107494	Gm8239	6:56581203-56652487	0.065264333	0	10.83689899	0.000875224	0.046484028
ENSMUSG00000015340	Cybb	X:9435252-9487771	110.5371857	233.3955383	-1.046050904	0.000885627	0.046819536
ENSMUSG00000002588	Pon1	6:5168090-5193946	0.735493667	0.113590333	2.816781658	0.0008858	0.046819536
XLOC_012040	XLOC_012040	10:20866941-20871539	0.218728333	0	8.932961517	0.000897756	0.04731067
ENSMUSG00000074207	Adh1	3:138260991-138290698	5.01235	2.051630333	1.362847201	0.000899953	0.047330041
ENSMUSG00000004383	Large1	8:72814599-73353540	6.779165667	3.240619667	1.222132314	0.000911396	0.047707092
ENSMUSG00000079110	Capn3	2:120456019-120504913	8.303796333	3.722421667	1.581335353	0.000911994	0.047707092
ENSMUSG00000093672	Gm20655	10:17790522-17845666	1.09981	0.320276667	1.978772606	0.000916182	0.047840307
ENSMUSG00000113303	4930423D22Rik	12:81716482-81783187	0.474752333	1.142428	-1.158629429	0.000917234	0.047840307
ENSMUSG00000066861	Oas1g	5:120871632-120887613	1.688981333	5.917646	-1.798334685	0.000928431	0.048353275
ENSMUSG00000055320	Tead1	7:112679318-112906807	13.08887733	6.621104667	1.191580843	0.000947676	0.049132026
ENSMUSG00000117671	AC130204.1	18:62903178-62922129	0.023121667	0	7.364269265	0.000951275	0.049182422
ENSMUSG00000042567	Nek10	14:14803415-15012059	0.058871667	0.009500667	2.854798769	0.000957621	0.04943858
XLOC_015052	XLOC_015052	10:109172906-109224150	0	0.090173333	-10.9012903	0.000959019	0.049438867

Table S6_lncRNA_Diff_gene.FPKM

gene_id	gene_name	gene_locus	M_K_1	M_K_2	M_K_3	M_W_1	M_W_2	M_W_3
ENSMUSG00000025900	Rp1	1:3999557-4409241	0.189229	0.431907	0.293561	0.118905	0.059795	0.103551
ENSMUSG00000057715	A830018L16Rik	1:11414105-11975901	0.082682	0.081312	0.101074	0.016573	0.009195	0.006349
ENSMUSG00000025938	Slco5a1	1:12866549-12992650	2.56374	5.609974	2.387259	1.342888	0.96999	1.716482
ENSMUSG00000025920	Stau2	1:16228674-16520112	6.051967	6.953719	4.515078	2.889068	2.089172	3.517238
ENSMUSG00000042686	Jph1	1:16964560-17097889	14.989416	19.823868	9.054596	4.499173	3.904521	9.200152
ENSMUSG00000099906	Gm28653	1:20669882-20684298	2.606201	6.317099	0.458821	0.382283	0.107167	0.420656
ENSMUSG00000026131	Dst	1:33908225-34308661	50.424458	79.738625	42.574829	23.98023	19.552919	26.194233
ENSMUSG00000096992	Gm26788	1:34011495-34308577	1.892993	3.413735	1.796071	1.035388	0.778364	0.962042
ENSMUSG00000026141	Col19a1	1:24261890-24587472	0.171826	0.315835	0.203268	0.065297	0.036743	0.075538
ENSMUSG00000101337	Dnah7c	1:46425592-46807476	0.003323	0.009069	0.003579	0	0	0
ENSMUSG00000026109	Tmeff2	1:50900647-51187270	0.433483	0.441909	0.35276	0.114856	0.099287	0.217605
ENSMUSG00000100120	Gm553	1:52522902-52582679	0.154506	0.189509	0.212116	0	0	0
XLOC_001142	XLOC_001142	1:56368465-56399157	0.08081	0	0.521544	0	0	0
ENSMUSG00000063558	Aox1	1:58029931-58106413	5.292553	6.473408	2.443835	1.036248	1.052245	1.963934
ENSMUSG00000087514	Gm16076	1:69091913-69106740	0.067754	0.238046	0.137657	0	0	0
ENSMUSG00000085653	Gm15179	1:75360322-75432318	15.559808	28.351999	8.638651	4.245792	4.016826	4.790942
ENSMUSG00000026208	Des	1:75360329-75368579	195.857941	270.260864	94.476875	54.267708	39.339714	85.936104
ENSMUSG00000086053	Gm15178	1:75375259-75432314	0	0	0.890621	0	0	0
ENSMUSG00000026207	Speg	1:75375297-75432320	13.116189	21.568518	11.231944	5.771243	5.099461	9.925188
ENSMUSG00000026430	Rassf5	1:131176410-131245258	21.320597	10.177802	7.738438	33.42432	43.468849	34.743103
ENSMUSG00000101168	Gm28892	1:134754744-134955938	0.936437	1.311386	0.705599	0.48824	0.382731	0.427013
ENSMUSG00000041801	Phlda3	1:135766119-135769136	27.704262	54.138802	16.942947	7.259149	9.815372	11.970333
ENSMUSG00000102717	Gm37759	1:136052534-136119924	2.83529	5.115926	2.074077	1.091212	1.026878	1.375481
ENSMUSG00000026407	Cacna1s	1:136052750-136119822	32.445843	55.893467	21.910622	11.006277	10.205235	21.228851
ENSMUSG00000066842	Hmcn1	1:150562524-150993435	1.135647	1.395617	1.143104	0.510105	0.535989	0.719996
ENSMUSG00000101880	Gm29282	1:155011332-155057179	0	0.310297	0.118199	0	0	0
ENSMUSG00000040265	Dnm3	1:161982453-162478034	2.510802	2.449904	1.310043	0.418714	0.598499	1.019971
ENSMUSG00000102548	Gm16701	1:166974023-167079312	0	0.418574	0.407109	0	0	0
ENSMUSG00000038034	Igsf8	1:172261641-172319841	14.042386	21.957678	12.428781	9.788151	9.294905	8.39014
ENSMUSG00000007097	Atp1a2	1:172271709-172298064	103.732658	160.243469	47.950241	26.043222	24.245926	38.186371
ENSMUSG00000037942	Crp	1:172698055-172833031	0.047866	0.055307	0	0	0	0
ENSMUSG00000026527	Rgs7	1:175059087-175492500	0.892413	1.015749	0.686395	0.341264	0.178581	0.422547
ENSMUSG00000026489	Coq8a	1:180165238-180199602	54.370831	96.542252	30.997538	20.244257	18.933928	29.731411
ENSMUSG00000055493	Epm2a	10:11343404-11459644	5.631817	8.771553	4.621526	2.368629	2.965129	3.752654

ENSMUSG00000093672	Gm20655	10:17790522-17845666	1.014853	1.611253	0.673324	0.347463	0.15825	0.455117
ENSMUSG00000039891	Txlnb	10:17796226-17845665	24.008047	33.174328	10.130969	4.666239	3.227106	9.146822
XLOC_012040	XLOC_012040	10:20866941-20871539	0.179985	0.4762	0	0	0	0
ENSMUSG00000019899	Lama2	10:26980036-27619758	11.414417	11.81463	6.577249	2.635841	3.14474	4.519952
ENSMUSG00000101636	4930579H20Rik	10:27264942-27616869	0.84525	0.97377	0.510422	0.132261	0.167038	0.191884
ENSMUSG00000019787	Trdn	10:33080554-33476709	30.45709	42.943554	17.477348	15.556572	7.234331	17.235401
ENSMUSG00000112707	D830005E20Rik	10:33083526-33475472	2.180963	3.432493	1.6162	0.708077	0.415165	0.864936
XLOC_012407	XLOC_012407	10:35313722-35323974	0.089513	0.405329	0	0	0	0
XLOC_012472	XLOC_012472	10:37541332-37615839	0	0	0	0.256823	0	0.509827
ENSMUSG00000038522	Mfsd4b1	10:40001575-40025268	1.191541	1.90288	0.40834	0.209739	0.283853	0.350812
ENSMUSG00000071317	Bves	10:45335772-45372479	4.225448	6.341542	2.595262	0.907808	1.128434	1.83416
ENSMUSG00000058297	Spock2	10:60106219-60135198	3.675873	2.782231	1.741292	0.508722	0.96264	1.330032
ENSMUSG00000069601	Ank3	10:69398773-70027438	18.868538	21.514423	11.611764	7.440314	5.348524	8.621415
ENSMUSG00000052613	Pcdh15	10:73099342-74649737	0.021268	0.071268	0.094017	0	0	0
ENSMUSG00000049422	Chchd10	10:75933130-75937747	83.851418	85.239159	69.817917	37.069389	27.390938	47.85191
XLOC_013803	XLOC_013803	10:77359458-77418254	0.198939	0.424564	0	0	0	0
ENSMUSG00000006498	Ptbp1	10:79854427-79864771	40.710178	35.23962	51.849529	76.123795	73.136299	92.32312
ENSMUSG00000057729	Prtn3	10:79874471-79883174	209.664215	99.324814	127.214119	245.099777	404.346954	381.293488
ENSMUSG00000020061	Mybpc1	10:88518279-88605152	112.02478	141.039856	53.428825	34.095097	25.873743	57.355186
XLOC_014408	XLOC_014408	10:89396205-89473954	0	0	0	0	0.310058	0
ENSMUSG00000015889	Lta4h	10:93453411-93484875	108.692589	68.366028	91.33448	142.812195	194.585358	201.007858
ENSMUSG00000019929	Dcn	10:97479609-97518143	161.311432	154.661713	212.382294	91.241745	97.552063	102.455795
ENSMUSG00000112810	Gm35101	10:99669514-99675623	0.08347	0.694626	0	0	0	0
ENSMUSG00000019892	Lrriq1	10:103046031-103236322	0.071942	0.006735	0.051668	0	0	0
ENSMUSG00000035948	Acss3	10:106933517-107123668	5.649817	2.962482	1.701647	0.924835	1.391783	1.537165
ENSMUSG00000112230	Ifngas1	10:118502035-118556525	0	0	0	0	0.026545	0.058206
ENSMUSG00000025432	Avil	10:127000709-127020994	2.093892	4.08933	1.113641	0.261777	0.422166	0.437545
ENSMUSG00000039914	Coq10a	10:128337754-128368997	24.824558	41.334358	14.716063	9.621404	10.38553	12.081171
ENSMUSG00000025348	Itga7	10:128933818-128958282	13.560992	15.533905	8.305347	3.244668	3.704493	7.159756
ENSMUSG00000057897	Camk2b	11:5969644-6066362	6.316861	11.324395	5.469607	2.745458	3.381536	4.849725
ENSMUSG00000020407	Upp1	11:9118103-9136170	7.617718	3.489452	3.417717	10.040064	15.895669	16.753523
ENSMUSG00000048834	Vstm2a	11:16257724-16427310	0.062143	0.086334	0	0	0	0
XLOC_025570	XLOC_025570	11:22534887-22536774	1.058293	1.219571	0	0	0	0
XLOC_025776	XLOC_025776	11:29500337-29502486	0	0	0	0.230432	0.171043	0.638324
ENSMUSG00000087306	A230004M16Rik	11:41710342-41973117	0	0.121854	0.408265	0	0	0

ENSMUSG00000020354	Sgcd	11:46896253-47989377	10.109759	15.440795	8.68861	4.273387	3.777876	7.263246
ENSMUSG00000072902	Gm10435	11:58993838-59139257	6.574646	14.843308	3.658172	3.182308	2.201576	3.361187
ENSMUSG00000061462	Obscn	11:58994256-59139170	88.657761	154.514023	62.381359	28.929325	17.003664	47.297676
ENSMUSG00000085479	9430073C21Rik	11:67171012-67199234	26.848156	34.305698	12.626121	11.720441	6.65231	8.818261
ENSMUSG00000085348	Myhas	11:67171012-67305975	93.294655	112.429749	63.550549	27.213585	20.035669	40.785233
ENSMUSG00000033196	Myh2	11:67171027-67197517	227.137894	274.677795	93.953239	82.388901	46.846966	88.225754
ENSMUSG00000018566	Slc2a4	11:69942539-69948188	47.446674	67.501335	30.16968	13.704762	12.417023	25.640417
ENSMUSG00000018574	Acadvl	11:70010183-70015411	56.961906	78.416489	36.80957	28.445547	29.382015	32.27438
ENSMUSG00000060600	Eno3	11:70657202-70662513	338.807678	428.484283	216.555542	101.439713	93.831688	213.441498
ENSMUSG00000045667	Smtnl2	11:72389164-72411713	18.757347	34.063667	17.059238	4.933288	5.337875	12.319098
ENSMUSG00000018845	Unc45b	11:82910550-82943403	10.918299	18.970388	7.817219	4.081529	3.868238	7.077353
XLOC_028053	XLOC_028053	11:87553081-87579007	0	0.550318	0	0	0	0
ENSMUSG00000085872	Gm11505	11:87793145-87804479	37.496536	18.950272	26.793306	67.045937	97.415947	51.662556
ENSMUSG00000009350	Mpo	11:87793581-87804413	354.045502	161.568756	258.496765	495.113617	647.235657	653.686157
ENSMUSG00000018428	Akap1	11:88830792-88864586	14.379584	19.468962	9.642833	6.428904	5.525415	6.939552
ENSMUSG00000038967	Pdk2	11:95026258-95041354	32.062119	45.411957	23.07398	11.935901	8.908084	18.863474
ENSMUSG00000085645	Hoxb5os	11:96291024-96306910	0.07142	0.310201	0.304606	0	0	0
ENSMUSG00000018411	Mapt	11:104231390-104332090	6.21826	6.900722	4.192693	1.722608	0.598792	2.515003
ENSMUSG00000001027	Scn4a	11:106318592-106353288	20.692854	24.071466	9.126823	4.272317	3.793212	8.49519
ENSMUSG00000041828	Abca8a	11:110025634-110095978	20.862169	19.2635	8.26364	6.273	4.911957	7.652058
ENSMUSG00000020738	Sumo2	11:115523102-115536276	2.632652	2.028026	2.908132	12.294086	8.535928	7.357289
ENSMUSG00000033880	Lgals3bp	11:118392751-118402092	20.784248	10.361292	30.428854	24.936468	49.550026	22.300621
ENSMUSG00000051510	Mafg	11:120625117-120633600	15.778861	9.501929	15.823712	29.528513	31.305891	27.858053
ENSMUSG00000021123	Rdh12	12:79208914-79222665	9.237092	4.901669	10.1006	15.441599	17.850721	16.518175
ENSMUSG00000113303	4930423D22Rik	12:81716482-81783187	0.515755	0.368322	0.54018	0.949201	1.401351	1.076732
ENSMUSG00000042724	Map3k9	12:81721010-81781175	9.172469	5.76801	6.938164	12.369067	15.818886	18.294062
ENSMUSG00000114150	Gm46367	12:83792603-83921910	0.445911	0.404109	0.097647	1.014329	1.572069	0.664321
ENSMUSG00000021238	Aldh6a1	12:84430717-84451004	23.68041	27.908684	13.766759	4.467451	9.918621	10.546184
ENSMUSG00000090185	Gm15523	12:103321142-103352460	3.643847	6.929307	3.915779	1.215433	1.126149	2.143126
ENSMUSG00000021200	Asb2	12:103321142-103356001	35.547394	59.200676	29.364798	8.459081	6.793862	22.613415
ENSMUSG00000114134	Gm47408	13:10700161-10830778	0.138927	0	0	0	0	0
ENSMUSG00000052374	Actn2	13:12269426-12340760	119.390175	138.900192	41.954262	33.524544	23.075748	43.737366
ENSMUSG00000047246	Hist1h2be	13:23551258-23698454	48.27515	42.410351	56.0098	297.158813	201.807129	117.435593
XLOC_038089	XLOC_038089	13:32484995-32530077	0.349713	0	0	0	0	0
ENSMUSG00000113935	Gm35732	13:31971086-31989599	0	0.168069	0.21206	0	0	0

ENSMUSG00000044951	Mylk4	13:32700834-32784017	12.567503	37.061981	22.661724	6.720384	5.771704	12.699156
ENSMUSG00000021373	Cap2	13:46501848-46650281	14.11479	18.310373	9.17102	3.99601	2.65569	7.457125
XLOC_038985	XLOC_038985	13:56678646-56679764	0.608684	0.26444	0.332622	0	0	0
ENSMUSG00000113295	Gm49357	13:61655303-61743770	0	0.13256	0	0	0	0
ENSMUSG00000021536	Adcy2	13:68620043-68999541	5.990838	6.129211	3.848713	2.046512	2.400636	3.260566
ENSMUSG00000021573	Tppp	13:74009407-74035753	4.441947	5.195082	2.264874	1.55596	1.518241	1.503627
ENSMUSG00000114372	2310067P03Rik	13:83065457-83194347	0.013885	0.136413	0.04233	0	0	0
ENSMUSG00000082488	1700119I11Rik	13:91853157-91876869	11.969909	18.094364	8.63302	3.07981	2.787398	3.47659
ENSMUSG00000021702	Thbs4	13:92751590-92794818	31.629574	32.755711	23.896994	8.302917	14.844624	14.42551
ENSMUSG00000114968	A630019I02Rik	13:93040547-93144604	3.382725	5.564912	2.151311	1.03853	0.934959	1.536978
ENSMUSG00000097582	Gm26527	13:93770950-93943089	0	0	0	0.336167	0	0.172686
ENSMUSG00000042082	Arsb	13:93771630-93943016	18.846676	10.430167	23.516615	37.380047	40.312275	32.74707
ENSMUSG00000042567	Nek10	14:14803415-15012059	0.09533	0.058454	0.022831	0.003801	0.011929	0.012772
ENSMUSG00000017491	Rarb	14:16430839-16819156	1.216089	1.922951	1.729319	0.747163	0.533505	1.291788
ENSMUSG00000068697	Myoz1	14:20649107-20656540	142.666779	189.636261	77.648804	32.000885	27.536366	64.586472
ENSMUSG00000021768	Dusp13	14:21733394-21797832	9.625962	15.560588	7.775581	1.608311	2.448981	5.631039
ENSMUSG00000021898	Asb14	14:26894557-26915258	7.905586	16.012392	5.340445	2.766828	2.212647	4.323972
ENSMUSG00000021957	Tkt	14:30548359-30574720	134.810059	79.23822	147.48555	166.083664	268.268799	260.514008
ENSMUSG00000071540	3425401B19Rik	14:32659119-32685293	14.952758	27.41263	9.138678	6.482332	4.121192	8.608951
ENSMUSG00000021798	Ldb3	14:34526603-34588682	75.041656	96.106789	40.719509	21.127724	12.964356	49.835182
ENSMUSG00000115410	2810457G06Rik	14:46882842-47053397	1.413339	1.636699	1.059255	0.118029	0.079545	0.16799
ENSMUSG00000021838	Samd4	14:46882854-47105815	10.228677	11.920414	7.974714	3.92377	3.78251	4.853072
ENSMUSG00000004558	Ndrg2	14:51905271-51914158	158.694672	233.03389	64.844406	43.437897	37.002785	59.227337
ENSMUSG00000040314	Ctsg	14:56099881-56102574	134.643692	55.720905	71.632675	157.406097	256.462219	257.390594
ENSMUSG00000035296	Sgcg	14:61219115-61258490	9.115052	14.976342	5.035398	2.854049	2.303936	4.079265
ENSMUSG00000087652	Gm15918	14:63602524-63607372	0.121399	0.251514	0.107865	0	0	0
ENSMUSG00000022032	Scara5	14:65666403-65764826	6.252192	4.671154	5.514922	1.884706	1.785563	2.770727
ENSMUSG00000045875	Adra1a	14:66635251-66771168	0.795704	1.152987	0.806968	0.259214	0.196884	0.347789
ENSMUSG00000022053	Ebf2	14:67233291-67430918	1.738544	3.046946	1.658443	0.891825	0.818924	1.385857
XLOC_049898	XLOC_049898	14:70443533-70462067	0.266302	0.231327	0	0	0	0
ENSMUSG00000054391	4930517O19Rik	14:100213142-100245339	0.053466	0.165875	0	0	0	0
ENSMUSG00000055737	Ghr	15:3317760-3583492	27.220072	27.519098	26.0604	11.906697	9.571865	19.554386
ENSMUSG00000005360	Slc1a3	15:8634124-8710764	7.272592	5.472443	4.641799	2.77904	2.504711	2.463558
XLOC_051387	XLOC_051387	15:33843805-33884624	0.157433	0.085118	0.877909	0	0	0
XLOC_054787	XLOC_054787	15:41767460-41775587	0	0	0	0	0.107425	0.463654

ENSMUSG00000097286	Gm26684	15:54887569-54964642	0.868567	1.088276	0.52916	0.149797	0.369117	0.162736
ENSMUSG00000086801	Gm15943	15:58176161-58324111	2.956234	3.57969	0.596267	0.248558	0.165516	0.222368
ENSMUSG00000022584	Ly6c2	15:75045013-75111970	210.022461	92.531754	123.394371	282.494904	477.557831	267.364655
ENSMUSG00000022562	Oplah	15:76296601-76331104	7.170881	11.019114	5.433851	3.672253	4.206752	3.075225
ENSMUSG00000018893	Mb	15:77014056-77057070	318.975983	421.783295	175.637482	81.237648	69.780724	107.752335
ENSMUSG00000116272	Gm49540	15:77014093-77015898	18.923668	34.343136	14.12046	6.106444	5.25597	4.976621
ENSMUSG00000071714	Csf2rb2	15:78282507-78305721	7.531548	4.117466	4.843844	9.803826	14.345797	13.383293
ENSMUSG00000071713	Csf2rb	15:78325752-78353847	21.243607	12.055823	14.183496	26.023102	37.978657	46.192463
ENSMUSG00000006369	Fbln1	15:85205949-85286535	4.415936	5.542073	5.127834	2.438004	2.475291	2.319554
ENSMUSG00000022383	Ppara	15:85734983-85802819	3.949977	8.835085	3.332283	2.393873	1.669592	1.400541
ENSMUSG00000078937	Cpt1b	15:89416405-89425863	34.765732	49.133801	17.748241	11.403525	7.367315	14.239826
ENSMUSG00000023019	Gpd1	15:99717515-99725005	39.529778	50.484715	39.278893	21.057611	16.911112	20.419518
ENSMUSG00000097003	D930007P13Rik	15:103123070-103146828	0.029628	0.051304	0.021531	0	0	0
ENSMUSG00000022519	Srl	16:4480216-4541816	66.851593	82.476471	38.522461	17.508316	16.821697	31.428219
XLOC_058158	XLOC_058158	16:25145203-25178075	0	0	0	0.478164	0.313234	0
ENSMUSG00000022548	Apod	16:31296192-31314808	11.977947	7.313138	6.060743	2.572932	2.701654	4.564178
ENSMUSG00000046598	Bdh1	16:31422280-31458901	4.685343	6.872138	3.692708	2.190344	2.146053	1.886648
ENSMUSG00000087689	Gm15845	16:36359497-36387406	0	0	0	0.07244	0.102253	0.028185
ENSMUSG00000116961	Gm49662	16:36569145-36598497	0	0	0	0.072373	0.100079	0.12244
ENSMUSG00000047746	Fbxo40	16:36963460-36990467	24.991873	34.869709	9.811453	3.874662	3.880787	8.250232
ENSMUSG00000022876	Samsn1	16:75858793-76022281	16.687382	5.866511	20.58794	31.175367	28.841333	37.889648
ENSMUSG00000022860	Chodl	16:78930948-78951733	3.515683	5.244725	2.722215	1.475525	1.135762	1.461924
ENSMUSG00000022935	Grik1	16:87895900-88290265	0.011099	0	0.047728	0	0	0
ENSMUSG00000116989	Gm49709	16:90349417-90362580	0.073418	0.133317	0.14105	0	0	0
XLOC_059723	XLOC_059723	16:92222254-92254039	0.470919	0	0	0	0	0
ENSMUSG00000045975	C2cd2	16:97855209-97962621	15.600654	13.752753	7.165874	7.114105	6.051224	7.484487
ENSMUSG00000004864	Mapk13	17:28769297-28780233	14.567048	7.204967	6.535522	12.214299	33.926224	21.049824
ENSMUSG00000043286	Pnpla1	17:28858411-28890309	0.611821	0.324123	0.37537	1.078298	1.42585	1.450022
XLOC_067356	XLOC_067356	17:35714814-35731414	0.484341	0.128141	0	0	0	0
XLOC_067694	XLOC_067694	17:45700029-45707217	0.358399	0	0.193294	0	0	0
ENSMUSG00000040694	Apobec2	17:48419231-48432930	67.650925	116.05278	46.424843	26.074371	17.253315	37.343361
ENSMUSG00000117222	Gm49906	17:50339252-50399172	0.596368	1.83008	0.252701	0.097307	0.161338	0.302774
XLOC_068078	XLOC_068078	17:55905746-55910656	0	0	0	0.359929	0.630439	0.128938
XLOC_068279	XLOC_068279	17:64217189-64234819	0.271362	0.028098	0.237093	0	0	0
ENSMUSG00000117231	Gm41609	17:70765839-70806059	0.009982	0.013912	0.080628	0	0	0

ENSMUSG00000045761	Togaram2	17:71673261-71729669	1.335486	2.123163	0.877724	0.512635	0.165068	0.38428
ENSMUSG00000024059	Clip4	17:71768473-71864273	9.263255	11.61393	5.457437	2.663038	2.155824	4.642004
ENSMUSG00000024049	Myom1	17:70994291-71126856	66.302429	85.669968	38.044285	25.024544	22.293051	36.547535
ENSMUSG00000038045	Sult6b1	17:78883938-78906992	0	0	0	0.11817	0.042593	0
ENSMUSG00000040505	Abcg5	17:84658234-84683011	0.238184	0.014989	0.017342	0	0	0
ENSMUSG00000117727	AC131675.1	18:35069325-35087623	0.123735	0.152842	0	0	0	0
ENSMUSG00000073600	Prob1	18:35650351-35655238	7.654097	11.884228	4.38887	2.124447	1.867164	3.374078
ENSMUSG00000086312	Gm15336	18:38993110-39364031	0.146569	1.198961	0.261647	0.053556	0.15227	0.058629
ENSMUSG00000024597	Slc12a2	18:57878678-57946821	10.46744	14.452013	8.325437	4.6554	4.609315	6.691126
ENSMUSG00000118370	AC124430.1	18:62142618-62146508	0.117244	0.350977	0.098722	0	0	0
ENSMUSG00000117671	AC130204.1	18:62903178-62922129	0.023054	0.03277	0.013541	0	0	0
ENSMUSG00000032845	Alpk2	18:65265529-65394066	9.741063	5.276732	4.993591	2.681571	1.771881	3.521634
ENSMUSG00000006457	Actn3	19:4861216-4877909	138.907822	186.523972	101.148705	39.876953	33.911671	99.62738
ENSMUSG00000084876	Gm14965	19:6384283-6418606	13.666636	20.607512	11.748583	6.486629	7.03571	7.651812
ENSMUSG00000032648	Pygm	19:6384399-6398459	229.82019	331.329895	157.124405	64.233887	50.256874	139.974991
XLOC_074422	XLOC_074422	19:6922224-6925167	0.231149	0.352388	0.06538	0	0	0
ENSMUSG00000024726	Carnmt1	19:18670764-18707200	6.318839	8.205927	9.889905	4.677574	3.316081	4.083441
ENSMUSG00000098739	Gm27151	19:22438520-22448865	0.02639	0.088669	0.16405	0	0	0
ENSMUSG00000071604	Fam189a2	19:23972750-24031019	3.866191	5.820934	2.747612	1.740358	1.796011	2.023666
ENSMUSG00000117979	AC150898.1	19:24295079-24558401	0	0	0	0	0.137589	0.042264
ENSMUSG00000041731	Pgm5	19:24683016-24861855	3.668835	7.691388	3.917106	1.667585	2.067634	3.024376
ENSMUSG00000118020	AC118724.1	19:27145316-27192009	0.021893	0.049609	0.009237	0	0	0
ENSMUSG00000024924	Vldlr	19:27216484-27254231	16.39431	17.778322	7.787628	4.518643	5.320346	6.173096
XLOC_077515	XLOC_077515	19:36852209-36915052	0.215699	0.501238	0	0	0	0
XLOC_075511	XLOC_075511	19:38420369-38430883	0.077791	0.48053	0	0	0	0
ENSMUSG00000114796	A930028N01Rik	19:40292041-40513752	1.483342	1.643782	0.990419	0.693063	0.77956	0.84178
ENSMUSG00000025006	Sorbs1	19:40294753-40513779	30.671438	36.298302	24.647179	14.341877	12.181499	18.126896
ENSMUSG00000074852	Hpse2	19:42786539-43388355	0.015501	0.016657	0.134808	0	0	0
ENSMUSG00000037071	Scd1	19:44394455-44407879	362.850769	178.503586	217.310608	120.578636	99.699089	142.581589
ENSMUSG00000043639	Rbm20	19:53677306-53867080	5.072065	7.103119	2.006209	1.465233	1.044614	2.201325
ENSMUSG00000087268	Gm14486	2:30658258-30678011	0.070336	0.027611	0.095375	0	0	0
ENSMUSG00000076441	Ass1	2:31470207-31520672	6.995034	3.567422	5.669792	10.198975	14.414454	11.684884
ENSMUSG00000055632	Hmcn2	2:31314415-31460738	11.233111	15.413557	9.121408	3.983041	2.882983	5.845851
ENSMUSG00000026817	Ak1	2:32621758-32635058	82.229973	109.031868	69.726173	27.544516	26.84697	58.735203
ENSMUSG00000017412	Cacnb4	2:52428320-52676831	0.162557	0.101158	0.086646	0.019408	0.036179	0.019885

ENSMUSG00000026950	Neb	2:52136647-52378474	120.106964	168.304184	73.056541	34.374599	22.654907	72.875298
ENSMUSG00000087518	Gm13561	2:62357071-62470913	0	0	0	0.008147	0.032593	0.02221
ENSMUSG00000087467	Gm13601	2:67446003-67526614	2.881283	5.074175	2.117879	0.725452	0.619301	0.990535
ENSMUSG00000087264	Gad1os	2:70489940-70563357	0	0	0	0	0.065545	0.023484
ENSMUSG00000051747	Ttn	2:76703980-76982547	325.878937	512.738586	192.332855	100.027985	60.429123	195.849152
ENSMUSG00000086354	Gm13938	2:76703984-76982555	13.320442	24.749712	8.92791	5.046041	2.600346	6.194265
ENSMUSG00000027210	Meis2	2:115863064-116065839	3.688862	4.025175	2.646539	1.294113	1.230157	1.899071
ENSMUSG00000087203	Gm13986	2:117857219-118111202	0.165205	0.371254	0.849751	0.102811	0.061	0.21128
ENSMUSG00000079110	Capn3	2:120456019-120504913	7.506831	12.479539	4.925019	4.254512	3.137667	3.775086
ENSMUSG00000027199	Gatm	2:122594467-122611303	17.669741	12.365108	13.35589	23.45125	32.098232	34.334801
ENSMUSG00000027360	Hdc	2:126593667-126619299	42.572933	20.621563	31.304707	54.50211	75.84816	72.983315
XLOC_088082	XLOC_088082	2:136535563-136624094	0	0.372795	0	0	0	0
ENSMUSG00000027438	Napb	2:148693864-148732467	2.119151	3.107188	1.060397	0.37601	0.418105	0.655467
ENSMUSG00000027488	Snta1	2:154376313-154408099	25.781422	28.324051	17.241861	8.02466	6.275601	12.938849
ENSMUSG00000017697	Ada	2:163726584-163750239	6.121356	3.016543	4.083539	7.757507	9.887567	12.493352
ENSMUSG00000017300	Tnnc2	2:164777161-164779967	327.755371	300.51828	178.606201	77.625221	44.099049	134.980408
ENSMUSG00000027514	Zbp1	2:173206612-173218923	4.460209	2.964027	3.413707	7.488238	20.865942	8.656306
ENSMUSG00000027513	Pck1	2:173153048-173159273	50.618282	69.674072	29.764284	16.315243	25.994909	20.867718
ENSMUSG00000027574	Nkain4	2:180934772-180954699	0.328298	0.139184	0	0	0	0
ENSMUSG00000043542	Zc2hc1a	3:7503483-7553836	3.548234	3.123228	2.163943	1.296046	1.473384	1.356447
ENSMUSG00000062515	Fabp4	3:10204088-10208576	287.324951	246.377258	166.938812	124.627258	90.09053	111.320717
ENSMUSG00000098008	A930001A20Rik	3:14971201-15002727	0.116628	0.025539	0	0	0	0
ENSMUSG00000102590	Mannr	3:29891014-29924191	0	0.135334	0.026243	0	0	0
ENSMUSG00000027716	Trpc3	3:36620482-36690167	1.763017	2.46746	1.945052	0.560744	0.591157	1.012956
ENSMUSG00000027737	Slc7a11	3:49892526-50443614	0.672875	0.160612	0.379323	0.870477	0.959628	1.792926
ENSMUSG00000027750	Postn	3:54361109-54391037	49.909554	46.6133	71.907722	21.002169	33.922642	28.813332
ENSMUSG00000048416	Mlf1	3:67374097-67400003	27.011593	32.786674	15.278214	9.788677	5.280916	14.299897
ENSMUSG00000027792	Bche	3:73635808-73708415	2.650239	3.129871	1.348414	0.722959	1.144778	1.258557
ENSMUSG00000028150	Rorc	3:94372794-94398276	9.35249	13.111726	4.56474	1.910991	2.126739	4.014127
ENSMUSG00000005628	Tmod4	3:95124476-95129209	32.05138	43.208496	19.802284	10.683309	7.911426	18.657408
ENSMUSG00000001025	S100a6	3:90612882-90624181	162.775116	70.852211	90.634041	215.103897	273.874573	243.637695
ENSMUSG00000093553	Gm20633	3:96238108-96247329	24.374084	11.397694	28.101305	73.727211	44.65728	34.855915
ENSMUSG00000074388	Gm5544	3:97930173-97967018	0	0	0	0.163253	0.023655	0.087694
ENSMUSG00000038170	Pde4dip	3:97689263-97888707	154.996674	227.241806	76.866882	42.147507	39.60421	73.673615
ENSMUSG00000105245	Gm31305	3:97689725-97868423	9.573303	14.716321	5.498835	2.853958	3.051326	3.81425

ENSMUSG00000105891	A230001M10Rik	3:102262405-102445132	0	0.008218	0.028739	0	0	0
ENSMUSG00000074264	Amy1	3:113555710-113606699	10.301716	11.670816	4.551882	2.463459	2.541562	3.828155
ENSMUSG00000033377	Palmd	3:116918258-116968987	4.168645	7.013927	3.195073	1.417124	1.162506	2.837333
ENSMUSG00000027961	Lrrc39	3:116562973-116583134	4.581731	6.609302	3.16371	2.02057	1.323913	2.001896
ENSMUSG00000028116	Myoz2	3:123006206-123035015	56.327526	57.510994	15.418513	13.473939	8.746342	14.161901
ENSMUSG00000050315	Synpo2	3:123076519-123236149	10.356476	3.903326	7.830533	1.554296	2.781612	2.78186
ENSMUSG00000090427	Gm17225	3:126432883-126439269	0.033242	0.071973	0.218647	0	0	0
ENSMUSG00000106245	Gm43824	3:128610987-128887161	0.248997	0.914656	0.105572	0	0.014903	0
ENSMUSG00000027999	Pla2g12a	3:129878606-129895825	15.837571	15.475362	12.095435	5.39325	4.438373	4.600812
ENSMUSG00000046818	Ddit4l	3:137621612-137628333	11.484389	25.100348	13.479694	3.913795	3.720282	7.49068
ENSMUSG00000090066	1110002E22Rik	3:138065052-138081506	10.094212	12.978111	6.255307	3.328026	2.013867	5.95328
ENSMUSG00000074207	Adh1	3:138260991-138290698	6.051908	5.6482	3.336942	2.151758	1.88547	2.117663
ENSMUSG00000036745	Ttll7	3:146852367-146984009	10.147112	8.078992	4.458247	1.8212	2.142288	3.669317
ENSMUSG00000106515	Gm30382	3:149284418-149445301	0.045049	0	0.023512	0	0	0
ENSMUSG00000062908	Acadm	3:153922357-153944632	40.892704	46.984257	28.447201	19.757324	15.883305	21.489956
ENSMUSG00000028177	1810013D15Rik	3:157925220-157938355	0.067002	0.192952	0.049982	0	0	0
ENSMUSG00000028223	Decr1	4:15917240-15945507	18.973486	21.024765	10.496339	7.744404	7.973293	9.652278
ENSMUSG00000028278	Rragd	4:32983037-33022180	10.753602	14.053856	7.106622	3.126675	3.347488	6.266987
ENSMUSG00000028427	Aqp7	4:41033074-41048139	8.173353	11.903798	5.100865	3.305241	2.963311	3.658704
ENSMUSG00000028441	1110017D15Rik	4:41505009-41517333	0.038074	0.034632	0.036423	0	0	0
ENSMUSG00000036162	Fam219aos	4:41517437-41569535	0.200467	0.344545	0	0	0	0
ENSMUSG00000028464	Tpm2	4:43514711-43523765	184.705429	218.027451	98.740059	64.303009	43.371605	94.65284
ENSMUSG00000090053	Palm2	4:57434247-57712016	0.403978	0.769783	0.968769	0.164039	0.431965	0.013649
ENSMUSG00000057280	Musk	4:58285960-58374303	6.703094	3.933741	2.202051	1.468257	0.9092	1.544514
ENSMUSG00000028396	2310002L09Rik	4:73939371-73950846	13.780335	20.728003	5.553471	2.090421	1.797797	4.046955
ENSMUSG00000086249	Gm12724	4:106241216-106290026	0.018301	0.098685	0	0	0	0
ENSMUSG00000028631	Kcnq4	4:120696138-120748612	1.73358	2.636094	0.930958	0.561996	0.359523	0.795433
ENSMUSG00000001333	Sync	4:129287617-129308559	5.881788	7.473801	3.42176	1.443167	1.120633	2.665413
ENSMUSG00000003644	Rps6ka1	4:133847290-133887797	57.24049	26.915747	49.896828	81.317291	99.407806	88.936104
ENSMUSG00000003038	Hmgn2	4:133964738-133968650	51.457088	29.793564	36.957123	161.385132	103.475395	86.691727
ENSMUSG00000028841	Cnksr1	4:134228041-134238399	7.281014	9.806435	1.939057	0.87783	0.542131	1.478563
ENSMUSG00000028834	Trim63	4:134315120-134329629	82.494316	44.462677	12.785818	8.367084	4.984142	7.954937
ENSMUSG00000037139	Myom3	4:135759715-135815564	15.611977	20.449221	7.252788	4.819408	3.404319	6.60485
ENSMUSG00000001604	Tcea3	4:136247729-136274898	12.28665	19.551701	12.00648	4.684535	3.290054	8.414799
ENSMUSG00000025330	Padi4	4:140745865-140774236	43.684849	32.373951	38.803989	53.5359	84.037964	80.634392

ENSMUSG00000028931	Kcnab2	4:152390742-152477910	9.546795	5.920547	9.686686	13.464388	19.250198	32.757553
ENSMUSG00000104982	Gm32554	5:11165537-11181825	0	0	0	0.061917	0.127825	0.035398
ENSMUSG00000104886	Gm43000	5:15934412-16371865	0.981547	1.568974	0.670384	0.395785	0.444056	0.404105
ENSMUSG00000040118	Cacna2d1	5:15934691-16374511	19.846191	23.376417	15.911866	6.54336	6.570269	11.46611
ENSMUSG00000049265	Kcnk3	5:30588170-30625271	2.745703	3.844807	1.963134	1.039178	1.081724	0.890867
ENSMUSG00000107265	Gm15469	5:31116556-31139243	1.513665	3.150943	1.177187	0.604332	0.276094	0.446193
ENSMUSG00000044716	Dok7	5:35056766-35087839	2.642431	3.441646	1.752482	0.872496	0.797618	0.942406
ENSMUSG00000029096	Htra3	5:35652023-35679782	20.325573	21.408447	20.01042	8.512339	10.030124	9.101528
ENSMUSG00000062329	Cytl1	5:37735519-37739820	175.133072	79.586792	126.584915	66.942032	64.758064	55.217842
ENSMUSG00000051596	Otop1	5:38275972-38304217	0.918289	2.002147	0.513923	0.167225	0.083294	0.141676
ENSMUSG00000060961	Slc4a4	5:88886818-89239653	4.079548	8.762353	2.617583	1.135627	1.284955	3.231344
ENSMUSG00000072720	Myo18b	5:112688876-112896362	18.991087	21.116631	8.512475	4.667921	3.753729	7.788217
ENSMUSG00000098230	1700095B10Rik	5:112688876-112896403	2.882207	3.290465	1.211056	0.849252	0.505091	0.766146
ENSMUSG00000042010	Acacb	5:114146535-114250761	22.974674	33.060917	12.328113	8.523938	6.65565	9.668551
ENSMUSG00000029561	Oasl2	5:114896936-114912234	14.083943	13.202185	13.731026	34.802559	69.841888	25.01973
ENSMUSG00000041827	Oasl1	5:114923240-114937915	1.817768	2.114944	1.803081	6.395544	11.358026	3.789595
ENSMUSG00000041548	Hspb8	5:116408491-116422864	35.439465	49.839783	19.864645	7.827689	7.703766	16.087257
ENSMUSG00000029361	Nos1	5:117781032-117958840	5.350559	9.830069	3.563759	2.539677	1.63826	2.896275
ENSMUSG00000098072	Gm26995	5:117781069-117955421	0.557887	0.965432	0.217597	0.114049	0.085754	0.09309
ENSMUSG00000032661	Oas3	5:120753098-120777661	17.963203	13.441889	16.673548	30.181379	60.195503	31.273039
ENSMUSG00000066861	Oas1g	5:120871632-120887613	1.514128	1.727677	1.825139	3.897707	10.121767	3.733464
ENSMUSG00000005373	Mlxipl	5:135089890-135138382	7.889306	14.706957	6.281682	2.684367	2.759969	4.4736
ENSMUSG00000107296	Gm43500	5:135090004-135138493	0.841915	1.36002	0.567242	0.29942	0.220227	0.157357
ENSMUSG00000002588	Pon1	6:5168090-5193946	0.448295	1.254733	0.503453	0.142872	0.03927	0.158629
ENSMUSG00000019577	Pdk4	6:5483351-5496309	196.34726	131.638016	35.122711	23.781073	21.199392	28.254171
ENSMUSG00000068794	Col28a1	6:7997808-8192617	1.182198	0.60028	0.761355	0.420856	0.419293	0.298833
ENSMUSG00000097364	Gm26719	6:21985908-22234659	0.829684	1.43622	0.800293	0.242058	0.436426	0.383886
ENSMUSG00000062980	Cped1	6:21985916-22256404	9.180496	13.751174	8.077229	4.661245	4.136992	6.866886
XLOC_122930	XLOC_122930	6:26752346-26766756	0.362202	0	0.053607	0	0	0
ENSMUSG00000024211	Grm8	6:27275119-28135178	0.241533	0.146469	0.500166	0.085486	0.039759	0.04104
ENSMUSG00000068699	Flnc	6:29433256-29461883	93.191925	101.316223	21.938105	17.092941	12.226638	23.054031
ENSMUSG00000003477	Inmt	6:55170626-55175043	7.041883	4.716788	1.242853	0.255015	0.338886	1.058579
ENSMUSG00000037709	Fam13a	6:58932090-59024549	8.067857	5.870786	2.26115	1.90097	1.918068	2.289836
ENSMUSG00000033182	Kbtbd12	6:88545114-88637950	2.385575	3.939582	2.155947	1.232362	0.927797	1.510257
ENSMUSG00000035357	Pdzrn3	6:101149609-101377897	4.245291	7.611697	4.294562	1.96057	2.352039	3.051409

ENSMUSG00000044574	5031434C07Rik	6:112273684-112330497	4.733029	6.330696	1.551849	0.723966	1.126597	0.976277
ENSMUSG00000030111	A2m	6:121635376-121679227	1.312113	0.724242	1.988556	4.811052	4.847731	2.41657
ENSMUSG00000107985	Gm35037	7:3451357-3475020	0	0	0	0.177032	0.230671	0.216541
ENSMUSG00000030401	Rtn2	7:19282624-19296160	41.739586	68.406593	33.373734	16.216421	12.379359	29.523775
ENSMUSG00000030399	Ckm	7:19404776-19422841	571.988037	737.431885	337.885071	134.076172	126.738258	308.635162
ENSMUSG00000040705	A930016O22Rik	7:19411019-19421633	163.765854	233.618881	88.305321	43.048805	43.130943	66.32093
ENSMUSG00000057101	Zfp180	7:24081924-24107713	2.473591	2.495514	3.57941	6.921985	2.930447	6.714542
ENSMUSG00000052212	Cd177	7:24743983-24760311	187.278641	97.686638	106.470894	219.069778	382.674469	379.081848
ENSMUSG00000030592	Ryr1	7:29003344-29125179	71.838737	126.838234	40.48912	23.156019	21.984829	39.704414
ENSMUSG00000109017	Gm38979	7:29005221-29030250	6.090543	10.510153	3.556397	2.939001	2.045484	3.126526
ENSMUSG00000036854	Hspb6	7:30552178-30555443	70.42907	117.103783	33.741276	10.977982	14.837252	35.583736
ENSMUSG00000109224	Tmem147os	7:30734739-30756126	0.006063	0	0	0.130546	0.117032	0.171257
ENSMUSG00000019194	Scn1b	7:31116524-31127003	37.143097	55.58699	28.97205	13.304977	13.992725	23.747435
ENSMUSG00000108607	Gm44646	7:44501689-44524670	11.601254	19.102322	8.817745	4.037475	3.297531	6.129159
ENSMUSG00000030739	Myh14	7:44605803-44670843	2.748072	5.342279	1.823642	0.985546	0.919619	1.309311
ENSMUSG00000038239	Hrc	7:45335290-45338974	26.081625	39.062725	18.26306	9.429741	5.611824	16.28731
ENSMUSG00000096146	Kcnj11	7:46093953-46100764	5.726388	10.541649	4.626218	1.492319	1.593075	3.702657
ENSMUSG00000058975	Kcnc1	7:46396497-46438704	5.168977	6.88288	3.086839	1.328094	1.220163	3.226746
ENSMUSG00000030470	Csrp3	7:48830398-48848033	84.833679	126.976074	19.894276	17.721628	14.321607	15.057315
ENSMUSG00000033510	Otud7a	7:63444751-63759028	0.011647	0.0393	0	0	0	0
ENSMUSG00000038763	Alpk3	7:81057600-81105612	17.269014	28.494181	8.747662	5.326618	3.877482	7.466403
ENSMUSG00000030562	Nox4	7:87246096-87398710	4.748874	3.405726	1.3073	0.596815	0.835995	0.86358
ENSMUSG00000052396	Mogat2	7:99219084-99238619	4.548224	2.541012	4.148274	6.729011	10.021544	7.886735
XLOC_135561	XLOC_135561	7:100432711-100436150	0.022851	0.215263	0.844706	0	0	0
ENSMUSG00000110301	Gm35363	7:101525376-101538330	0.061605	0.023028	0.066339	0	0	0
ENSMUSG00000070424	Art5	7:102096879-102111145	6.837045	14.438654	2.663344	2.004891	1.508225	1.709208
ENSMUSG00000030921	Trim30a	7:104409025-104465193	17.659306	12.623457	14.172953	23.167709	48.173321	28.639343
ENSMUSG00000085945	2310014F06Rik	7:112612560-112899129	2.085256	5.665073	2.292864	1.283828	0.54287	1.085305
ENSMUSG00000055320	Tead1	7:112679318-112906807	12.563759	17.661581	9.041292	6.873827	3.726031	9.263456
ENSMUSG00000087621	1700003G18Rik	7:116081759-116093159	0.056605	0.128325	0.09598	0	0	0
ENSMUSG00000109814	Gm45847	7:123031415-123068305	0.098431	0.192003	0.116182	0	0	0
ENSMUSG00000085899	Gm15338	7:124186718-124290270	0	0	0	0	0.018329	0.05022
ENSMUSG00000030727	Rabep2	7:126428759-126463103	111.147362	177.378784	76.303146	38.965443	35.536278	53.049446
ENSMUSG00000030672	Mylpf	7:127208890-127214298	638.270142	567.235291	333.54541	192.131912	117.268715	313.058289
ENSMUSG00000030786	Itgam	7:128062640-128118491	29.748968	18.023985	34.188301	43.707359	96.480957	72.026367

ENSMUSG00000030852	Tacc2	7:130577438-130764785	81.491547	99.968086	25.836329	20.798128	16.107763	22.325216
ENSMUSG00000030862	Cpxm2	7:132032687-132154739	2.920636	3.67139	3.380141	1.01982	1.759792	2.193085
ENSMUSG00000078566	Bnip3	7:138890836-138909519	65.425331	75.519249	24.932837	18.909075	18.715168	28.530859
ENSMUSG00000025473	Adam8	7:139978932-139992562	24.942036	17.690407	24.933825	35.36813	51.305012	56.494198
ENSMUSG00000025479	Cyp2e1	7:140763739-140774987	93.822601	34.017395	9.14242	9.215931	17.173466	11.07816
ENSMUSG00000031097	Tnni2	7:142441808-142444410	350.609955	369.912109	197.856964	99.058754	45.605801	149.502716
ENSMUSG00000061723	Tnnt3	7:142498836-142516009	510.147858	479.736053	315.592804	160.463852	65.490761	280.691895
ENSMUSG00000031461	Myom2	8:15057653-15133541	62.65015	84.13942	29.318474	14.137247	10.8009	32.55888
ENSMUSG00000109689	Gm45646	8:26745929-26760561	0	0	0	0.196424	0	0.215453
XLOC_141650	XLOC_141650	8:33141724-33145306	0	0	0	0.051149	0.458685	0.420891
ENSMUSG00000109773	Gm34474	8:35218638-35222071	0.203593	0.098986	0.241328	0	0	0
ENSMUSG00000031636	Pdlim3	8:45885461-45919548	52.773067	70.074478	19.588997	11.847183	7.821683	19.502911
ENSMUSG00000031633	Slc25a4	8:46206797-46211284	234.414154	289.013275	125.053024	77.575508	70.677017	113.733757
ENSMUSG00000031519	Asb5	8:54520454-54587842	27.349512	35.88163	10.119218	5.442784	5.440066	9.589002
XLOC_142230	XLOC_142230	8:55100395-55401752	0.830438	1.172493	0.359629	0.055563	0.028033	0.155542
XLOC_142238	XLOC_142238	8:55353318-55378548	4.709507	7.984171	2.530534	0.359983	0.324907	1.438882
ENSMUSG00000015568	Lpl	8:68880491-68907448	142.796829	189.123672	82.521324	46.578331	47.141281	50.591007
ENSMUSG00000031849	Comp	8:70373558-70382066	2.373765	3.627653	4.322325	75.675392	134.20929	47.896313
XLOC_139182	XLOC_139182	8:72231215-72238975	0	0.055642	0.123218	2.992515	1.493483	2.200503
ENSMUSG00000004383	Large1	8:72814599-73353540	6.178689	8.911057	5.247751	2.190112	2.853855	4.677892
ENSMUSG00000037940	Inpp4b	8:81342556-82127914	6.520096	7.46891	6.357718	3.723845	3.001013	4.226166
ENSMUSG00000031709	Tbc1d9	8:83165352-83272934	1.089778	0.825165	1.295957	2.222201	2.29037	2.131517
ENSMUSG00000034656	Cacna1a	8:84338639-84640427	2.104686	4.209404	3.507574	0.919677	0.832667	0.729024
ENSMUSG00000097193	Gm26664	8:84665128-84669406	0	0	0	0.899919	0	0.54337
XLOC_139675	XLOC_139675	8:85461249-85471904	0.146981	0.062774	0.237415	0	0	0
XLOC_139731	XLOC_139731	8:86798464-86809300	2.660318	4.464123	1.005738	0	0	0
ENSMUSG00000056973	Ces1d	8:93166068-93197838	25.526508	21.636831	12.158961	6.861288	10.29727	8.947692
ENSMUSG00000110534	Gm45708	8:93565977-93628624	0.044173	0.064024	0	0	0	0
ENSMUSG00000034361	Cpne2	8:94532990-94570531	20.007915	11.300915	17.867147	30.087252	45.952045	39.254467
ENSMUSG00000031782	Coq9	8:94838321-94854895	37.189945	52.159184	24.734293	17.494272	15.668142	20.958084
XLOC_140383	XLOC_140383	8:111223081-111259206	0	0	0	0	0.54296	0
ENSMUSG00000041624	Gucy1a2	9:3532778-3894736	1.433041	2.275757	1.731889	0.695316	0.792699	0.964422
ENSMUSG00000025317	Car5a	8:121916126-121944904	0	0	0	0.042277	0.007545	0.032791
ENSMUSG00000111271	Gm48127	9:40342308-40342938	0	0	0	0.314588	0.771643	0.968053
ENSMUSG00000066705	Fxyd6	9:45370185-45396159	15.480872	21.783075	11.671079	7.778628	7.244522	7.916094

ENSMUSG00000032060	Cryab	9:50751325-50756636	72.517059	92.065239	57.698311	20.451912	18.0481	30.033419
ENSMUSG00000032238	Rora	9:68621970-69388246	10.468107	12.849206	8.491377	3.863276	4.047357	6.084583
ENSMUSG00000036030	Prtg	9:72806874-72917291	0.327751	0.476445	0.262718	0.066761	0.17296	0.096797
ENSMUSG00000111610	Gm34829	9:77992599-78002261	0.17922	0.020446	0.100298	0	0	0
ENSMUSG00000034898	Filip1	9:79815051-80012851	7.509134	8.75172	3.623574	2.139419	1.394546	3.170795
ENSMUSG00000032369	Plscr1	9:92249750-92272278	12.126713	10.877908	7.94132	12.976647	28.408585	27.547121
XLOC_147929	XLOC_147929	9:96385428-96396965	0	0	0	0.054327	0.376673	0
ENSMUSG00000032561	Acpp	9:104288251-104337748	8.516845	5.139655	7.403999	10.950871	18.524906	20.487772
ENSMUSG00000032496	Ltf	9:111019271-111042767	760.31012	383.823395	544.168091	990.970093	1388.92004	1628.36096
ENSMUSG00000074001	Klhl40	9:121777607-121783818	20.029026	27.694986	7.660397	4.416673	3.374677	7.393768
XLOC_153739	XLOC_153739	X:9747754-9786815	0.216771	0.458987	0.196436	0	0	0
ENSMUSG00000015340	Cybb	X:9435252-9487771	155.552277	75.948051	100.111229	177.878754	267.157684	255.150177
ENSMUSG00000023092	Fhl1	X:56731787-56793346	157.076538	252.220932	64.800949	43.720482	39.381424	64.407692
ENSMUSG00000031400	G6pdx	X:74409483-74429194	75.708847	33.783077	41.43264	90.243599	129.008759	140.645752
ENSMUSG00000045103	Dmd	X:82948870-85206141	19.207094	30.731009	10.963307	5.477915	4.246281	11.490897
ENSMUSG00000035522	Tsga8	X:82948902-83955069	0.686226	1.199417	0.464249	0.184986	0.087812	0.434582
ENSMUSG00000034457	Eda2r	X:97333840-97377216	2.795545	4.677947	0.967853	0.822597	0.533146	0.755785
ENSMUSG00000085715	Tsix	X:103431517-103484977	0.094483	0.003339	0.080172	1.95128	1.305436	0.174857
ENSMUSG00000086503	Xist	X:103460366-103483254	0.004498	0.013739	0.010954	75.766724	78.852585	0.009129
ENSMUSG00000034055	Phka1	X:102513975-102644246	23.153721	30.722326	14.847357	6.410928	5.785163	11.460017
ENSMUSG00000059203	Il1rapl2	X:137570608-138846946	0.023606	0.010253	0	0	0	0
ENSMUSG00000031274	Col4a5	X:141475385-141689234	0.745708	1.058501	0.996213	0.370384	0.535127	0.38247
ENSMUSG00000041476	Smpx	X:157698910-157752591	30.490894	40.490688	15.16251	7.966287	4.908206	13.389468
ENSMUSG00000031382	Asb11	X:164436994-164459170	23.45471	32.926437	6.932832	3.488901	2.577153	7.545769
XLOC_015052	XLOC_015052	10:109172906-109224150	0	0	0	0	0.27052	0
ENSMUSG00000111894	Gm47843	10:23573020-23609431	0	0	0	0.017249	0	0.114933
ENSMUSG00000006764	Tph2	10:115078641-115185022	0	0	0	0	0.022663	0.015596
ENSMUSG00000086501	4930597A21Rik	11:44575191-44591343	0	0.034151	0	1.093601	0.971967	0.740732
ENSMUSG00000041014	Nrg3	14:38368952-39473088	0	0	0	0	0.007419	0.022709
XLOC_054886	XLOC_054886	15:48842102-48887572	0	0	0	0	0	1.683566
ENSMUSG00000043460	Elfn2	15:78667331-78718771	0	0	0	0.00965	0.015793	0.021736
ENSMUSG00000102069	1700012I11Rik	15:67226769-67377094	0	0.12675	0	0	0	0
XLOC_060311	XLOC_060311	16:15004988-15058156	0	0	0	0	0	0.443568
ENSMUSG00000041565	L3mbtl4	17:68273797-68780086	0	0	0	0	0.036512	0.06612
XLOC_068153	XLOC_068153	17:56988235-56990166	0	0	0	0.229946	0.341275	0.101376

XLOC_067665	XLOC_067665	17:45313717-45338018	0	0.07934	0.601161	0	0	0
ENSMUSG00000043705	Capn13	17:73306464-73400866	0	0	0	0.013912	0	0.060631
XLOC_070043	XLOC_070043	18:25962950-25999134	0	0.487173	0	0	0	0
XLOC_078968	XLOC_078968	2:19415396-19423529	0	0	0	0.207206	0	0.466736
ENSMUSG00000054510	Gm14461	2:78237547-78302230	0	0	0	0.035456	0.172701	0.030161
ENSMUSG00000100197	Gm28638	2:66125317-66175250	0	0	0	0.099781	0	0.109196
XLOC_082853	XLOC_082853	2:149319511-149329629	0	0	0	0	0.310601	0.14027
XLOC_080635	XLOC_080635	2:72554811-72565244	0	0	0	0.373019	0.039057	0
XLOC_094921	XLOC_094921	3:21437743-21489557	0	0	0	0.495543	0	0
ENSMUSG00000085931	Gm12648	4:94089576-94425588	0	0	0	0	0	0.116515
XLOC_100810	XLOC_100810	4:64572023-64693682	0	0	0	0	0.288288	0
XLOC_101540	XLOC_101540	4:101676427-101716848	0	0	0.564338	0	0	0
XLOC_115932	XLOC_115932	5:112123805-112154913	0	0	0	0	0	0.552538
ENSMUSG00000107494	Gm8239	6:56581203-56652487	0	0	0.195793	0	0	0
XLOC_134212	XLOC_134212	7:51803863-51808709	0	0.293657	0.435599	0	0	0
ENSMUSG00000038257	Glra3	8:55940460-56130070	0	0	0	0	0.009114	0.01888
XLOC_141003	XLOC_141003	8:8959708-8969766	0	0.157366	0.627049	0	0	0
ENSMUSG00000101734	4933400C23Rik	9:92717456-92795711	0	0	0	0.100303	0.184743	0.124814
XLOC_146684	XLOC_146684	9:63093399-63099161	0	0.3605	0.28675	0	0	0
XLOC_157667	XLOC_157667	X:15981214-16028775	0	0.316043	0	0	0	0
ENSMUSG00000041710	Trpc5	X:144381671-144688180	0	0	0	0.005073	0.02713	0
XLOC_159384	XLOC_159384	X:97827562-97923336	0	0	0.537103	0	0	0

Table S9_KEGG analysis of target genes list of co-expression of differential lncRNA_top20

pathway_term	rich_factor	qvalue	gene_number
Parkinson's disease	0.442176871	0.098238993	65
Carbon metabolism	0.459459459	0.098238993	51
Huntington's disease	0.412087912	0.098238993	75
Dilated cardiomyopathy	0.483146067	0.098238993	43
Citrate cycle (TCA cycle)	0.625	0.176545044	20
Alzheimer's disease	0.398843931	0.176545044	69
Propanoate metabolism	0.62962963	0.236830232	17
Valine, leucine and isoleucine degradation	0.5	0.236830232	26
Pyruvate metabolism	0.538461538	0.236830232	21
Oxidative phosphorylation	0.4	0.236830232	54
Fatty acid metabolism	0.5	0.236830232	25
Hypertrophic cardiomyopathy (HCM)	0.428571429	0.317457646	36
Leukocyte transendothelial migration	0.396694215	0.317657905	48
Cardiac muscle contraction	0.423076923	0.386217856	33
Fatty acid degradation	0.469387755	0.386217856	23
Non-alcoholic fatty liver disease (NAFLD)	0.37012987	0.413687139	57
Leishmaniasis	0.430769231	0.416085544	28
Regulation of actin cytoskeleton	0.350230415	0.416085544	76
Thyroid hormone signaling pathway	0.381355932	0.421212441	45
MAPK signaling pathway	0.339920949	0.456176515	86

Table S10_KEGG analysis of target genes list of co-location of differential lncRNA_top20

pathway_term	rich_factor	qvalue	gene_number
Asthma	0.291666667	0.015881829	7
Intestinal immune network for IgA production	0.19047619	0.031786152	8
Viral myocarditis	0.128205128	0.064719851	10
Antigen processing and presentation	0.12345679	0.064719851	10
HTLV-I infection	0.072202166	0.163101	20
Dilated cardiomyopathy	0.101123596	0.228648102	9
Arrhythmogenic right ventricular cardiomyopathy (ARVC)	0.108108108	0.228648102	8
Leishmaniasis	0.107692308	0.281636875	7
Rheumatoid arthritis	0.097560976	0.281636875	8
Staphylococcus aureus infection	0.117647059	0.281636875	6
Hypertrophic cardiomyopathy (HCM)	0.095238095	0.281636875	8
Allograft rejection	0.111111111	0.308850515	6
Graft-versus-host disease	0.107142857	0.317667156	6
Inflammatory bowel disease (IBD)	0.101694915	0.317667156	6
Spliceosome	0.075757576	0.317667156	10
Cardiac muscle contraction	0.08974359	0.317667156	7
Alzheimer's disease	0.069364162	0.317667156	12
Type I diabetes mellitus	0.098360656	0.317667156	6
Proteasome	0.111111111	0.317667156	5
Oxidative phosphorylation	0.074074074	0.317667156	10

Table S12_M_K_vs_M_W.DE

ID	M_K_readcount	M_W_readcount	log2FoldChange	pval	padj
mmu_circ_0001688	0	12.64393114	-6.0577	0.00021507	0.44994
mmu_circ_0000048	8.027217236	0	5.5875	0.0027037	0.92431
mmu_circ_0000061	0	5.058089287	-4.7419	0.025277	0.92431
mmu_circ_0000129	0	5.771931539	-4.929	0.015717	0.92431
mmu_circ_0000474	17.23506598	2.262909411	3.0921	0.0058037	0.92431
mmu_circ_0001447	23.53748951	5.963539197	1.9651	0.025724	0.92431
mmu_circ_0001624	102.782775	188.7246705	-0.87253	0.015067	0.92431
novel_circ_0000282	4.468952865	0	4.7406	0.035845	0.92431
novel_circ_0001576	3.950794985	0	4.5659	0.048958	0.92431
novel_circ_0001933	6.927830109	0	5.3855	0.0051764	0.92431
novel_circ_0002440	0	4.245645883	-4.4789	0.046222	0.92431
novel_circ_0002484	22.47746248	5.950492833	1.9863	0.028705	0.92431
novel_circ_0002546	4.072391023	0	4.6157	0.043432	0.92431
novel_circ_0003152	7.795127281	0.508761886	3.7983	0.046641	0.92431
novel_circ_0003426	4.847612228	0	4.8651	0.024417	0.92431

Table S14_KEGG analysis of differentially expressed circRNA_top20

pathway_term	rich_factor	qvalue	gene_number
Cell cycle	0.015873016	0.088864503	2
Circadian rhythm	0.032258065	0.180074619	1
Porphyrin and chlorophyll metabolism	0.025	0.180074619	1
Aminoacyl-tRNA biosynthesis	0.015151515	0.180074619	1
TGF-beta signaling pathway	0.012195122	0.180074619	1
Oocyte meiosis	0.009009009	0.180074619	1
Vascular smooth muscle contraction	0.0078125	0.180074619	1
Axon guidance	0.007751938	0.180074619	1
Ubiquitin mediated proteolysis	0.007194245	0.180074619	1
Wnt signaling pathway	0.006993007	0.180074619	1
Non-alcoholic fatty liver disease (NAFLD)	0.006493506	0.180074619	1
Oxytocin signaling pathway	0.006329114	0.180074619	1
Protein processing in endoplasmic reticulum	0.00591716	0.180074619	1
Proteoglycans in cancer	0.004878049	0.180074619	1
Herpes simplex infection	0.004878049	0.180074619	1
Focal adhesion	0.004830918	0.180074619	1
Regulation of actin cytoskeleton	0.004608295	0.180074619	1
Endocytosis	0.004329004	0.180074619	1
Metabolic pathways	0.000796178	0.644792953	1

Table S15_mapping small RNA reads to reference

Sample	Total sRNA	Mapped sRNA	"+" Mapped sRNA	"-" Mapped sRNA
M_K_1	14237397 (100.00%)	12993994 (91.27%)	9401216 (66.03%)	3592778 (25.23%)
M_K_2	14424737 (100.00%)	13155070 (91.20%)	10012320 (69.41%)	3142750 (21.79%)
M_K_3	10553176 (100.00%)	9596804 (90.94%)	7433584 (70.44%)	2163220 (20.50%)
M_W_1	11804012 (100.00%)	10660510 (90.31%)	7902613 (66.95%)	2757897 (23.36%)
M_W_2	14906706 (100.00%)	13419630 (90.02%)	9692714 (65.02%)	3726916 (25.00%)
M_W_3	13235855 (100.00%)	11913572 (90.01%)	8756641 (66.16%)	3156931 (23.85%)

Table S16_new miRNA and the comparison of each sample sRNA

Types	Total	M_K_1	M_K_2	M_K_3	M_W_1	M_W_2	M_W_3
Mapped mature	50	41	25	6	22	19	7
Mapped star	6	1		1	4	2	
Mapped hairpin	51	41	25	7	24	23	8
Mapped uniq sRNA	194	71	34	11	35	32	11
Mapped total sRNA	1681	830	180	43	246	324	58

Table S19_circRNA-miRNA-mRNA-interaction-with-cartilage

circRNA_ID	miRNA	target_mRNA	target_gene	
novel_circ_0001062	mmu-let-7g-5p	ENSMUST00000073554	ENSMUSG00000062329	
novel_circ_0001450	mmu-let-7g-5p	ENSMUST00000073554	ENSMUSG00000062329	
novel_circ_0001501	mmu-let-7g-5p	ENSMUST00000073554	ENSMUSG00000062329	
novel_circ_0001501	mmu-let-7g-5p	ENSMUST00000073554	ENSMUSG00000062329	
mmu_circ_0000040	mmu-let-7g-5p	ENSMUST00000073554	ENSMUSG00000062329	
mmu_circ_0000041	mmu-let-7g-5p	ENSMUST00000073554	ENSMUSG00000062329	
mmu_circ_0000042	mmu-let-7g-5p	ENSMUST00000073554	ENSMUSG00000062329	
mmu_circ_0001376	mmu-let-7g-5p	ENSMUST00000073554	ENSMUSG00000062329	
mmu_circ_0001263	mmu-let-7g-5p	ENSMUST00000073554	ENSMUSG00000062329	
mmu_circ_0001264	mmu-let-7g-5p	ENSMUST00000073554	ENSMUSG00000062329	
mmu_circ_0001469	mmu-let-7g-5p	ENSMUST00000073554	ENSMUSG00000062329	
mmu_circ_0001500	mmu-let-7g-5p	ENSMUST00000073554	ENSMUSG00000062329	
mmu_circ_0001804	mmu-let-7g-5p	ENSMUST00000073554	ENSMUSG00000062329	
mmu_circ_0001827	mmu-let-7g-5p	ENSMUST00000073554	ENSMUSG00000062329	
mmu_circ_0001675	mmu-let-7g-5p	ENSMUST00000073554	ENSMUSG00000062329	
mmu_circ_0000243	mmu-let-7g-5p	ENSMUST00000073554	ENSMUSG00000062329	
mmu_circ_0000277	mmu-let-7g-5p	ENSMUST00000073554	ENSMUSG00000062329	
mmu_circ_0000141	mmu-let-7g-5p	ENSMUST00000073554	ENSMUSG00000062329	
mmu_circ_0000499	mmu-let-7g-5p	ENSMUST00000073554	ENSMUSG00000062329	
mmu_circ_0000500	mmu-let-7g-5p	ENSMUST00000073554	ENSMUSG00000062329	
mmu_circ_0000349	mmu-let-7g-5p	ENSMUST00000073554	ENSMUSG00000062329	
mmu_circ_0000376	mmu-let-7g-5p	ENSMUST00000073554	ENSMUSG00000062329	
mmu_circ_0000628	mmu-let-7g-5p	ENSMUST00000073554	ENSMUSG00000062329	
mmu_circ_0000530	mmu-let-7g-5p	ENSMUST00000073554	ENSMUSG00000062329	
mmu_circ_0000804	mmu-let-7g-5p	ENSMUST00000073554	ENSMUSG00000062329	
mmu_circ_0000828	mmu-let-7g-5p	ENSMUST00000073554	ENSMUSG00000062329	
mmu_circ_0000829	mmu-let-7g-5p	ENSMUST00000073554	ENSMUSG00000062329	
mmu_circ_0000712	mmu-let-7g-5p	ENSMUST00000073554	ENSMUSG00000062329	
mmu_circ_0000964	mmu-let-7g-5p	ENSMUST00000073554	ENSMUSG00000062329	
mmu_circ_0000840	mmu-let-7g-5p	ENSMUST00000073554	ENSMUSG00000062329	
mmu_circ_0000884	mmu-let-7g-5p	ENSMUST00000073554	ENSMUSG00000062329	
mmu_circ_0000886	mmu-let-7g-5p	ENSMUST00000073554	ENSMUSG00000062329	
mmu_circ_0000905	mmu-let-7g-5p	ENSMUST00000073554	ENSMUSG00000062329	
mmu_circ_0000912	mmu-let-7g-5p	ENSMUST00000073554	ENSMUSG00000062329	
novel_circ_0001564	mmu-let-7g-5p	ENSMUST00000073554	ENSMUSG00000062329	
novel_circ_0001579	mmu-let-7g-5p	ENSMUST00000073554	ENSMUSG00000062329	
novel_circ_0001579	mmu-let-7g-5p	ENSMUST00000073554	ENSMUSG00000062329	
novel_circ_0001608	mmu-let-7g-5p	ENSMUST00000073554	ENSMUSG00000062329	
novel_circ_0001702	mmu-let-7g-5p	ENSMUST00000073554	ENSMUSG00000062329	
novel_circ_0001702	mmu-let-7g-5p	ENSMUST00000073554	ENSMUSG00000062329	
novel_circ_0001743	mmu-let-7g-5p	ENSMUST00000073554	ENSMUSG00000062329	
novel_circ_0002071	mmu-let-7g-5p	ENSMUST00000073554	ENSMUSG00000062329	
novel_circ_0002086	mmu-let-7g-5p	ENSMUST00000073554	ENSMUSG00000062329	
novel_circ_0002145	mmu-let-7g-5p	ENSMUST00000073554	ENSMUSG00000062329	
novel_circ_0001759	mmu-let-7g-5p	ENSMUST00000073554	ENSMUSG00000062329	
novel_circ_0001864	mmu-let-7g-5p	ENSMUST00000073554	ENSMUSG00000062329	
novel_circ_0001870	mmu-let-7g-5p	ENSMUST00000073554	ENSMUSG00000062329	
novel_circ_0001902	mmu-let-7g-5p	ENSMUST00000073554	ENSMUSG00000062329	
novel_circ_0001903	mmu-let-7g-5p	ENSMUST00000073554	ENSMUSG00000062329	
novel_circ_0002039	mmu-let-7g-5p	ENSMUST00000073554	ENSMUSG00000062329	
novel_circ_0002417	mmu-let-7g-5p	ENSMUST00000073554	ENSMUSG00000062329	
novel_circ_0002231	mmu-let-7g-5p	ENSMUST00000073554	ENSMUSG00000062329	
novel_circ_0002343	mmu-let-7g-5p	ENSMUST00000073554	ENSMUSG00000062329	
novel_circ_0002866	mmu-let-7g-5p	ENSMUST00000073554	ENSMUSG00000062329	
novel_circ_0002913	mmu-let-7g-5p	ENSMUST00000073554	ENSMUSG00000062329	
novel_circ_0002926	mmu-let-7g-5p	ENSMUST00000073554	ENSMUSG00000062329	
novel_circ_0002933	mmu-let-7g-5p	ENSMUST00000073554	ENSMUSG00000062329	

novel_circ_0002732	mmu-let-7g-5p	ENSMUST00000073554	ENSMUSG00000062329
novel_circ_0002782	mmu-let-7g-5p	ENSMUST00000073554	ENSMUSG00000062329
novel_circ_0002788	mmu-let-7g-5p	ENSMUST00000073554	ENSMUSG00000062329
novel_circ_0003190	mmu-let-7g-5p	ENSMUST00000073554	ENSMUSG00000062329
novel_circ_0003213	mmu-let-7g-5p	ENSMUST00000073554	ENSMUSG00000062329
novel_circ_0003304	mmu-let-7g-5p	ENSMUST00000073554	ENSMUSG00000062329
novel_circ_0003096	mmu-let-7g-5p	ENSMUST00000073554	ENSMUSG00000062329
novel_circ_0003507	mmu-let-7g-5p	ENSMUST00000073554	ENSMUSG00000062329
novel_circ_0003457	mmu-let-7g-5p	ENSMUST00000073554	ENSMUSG00000062329
novel_circ_0000200	mmu-let-7g-5p	ENSMUST00000073554	ENSMUSG00000062329
novel_circ_0000201	mmu-let-7g-5p	ENSMUST00000073554	ENSMUSG00000062329
novel_circ_0000204	mmu-let-7g-5p	ENSMUST00000073554	ENSMUSG00000062329
novel_circ_0000204	mmu-let-7g-5p	ENSMUST00000073554	ENSMUSG00000062329
novel_circ_0000220	mmu-let-7g-5p	ENSMUST00000073554	ENSMUSG00000062329
novel_circ_0000222	mmu-let-7g-5p	ENSMUST00000073554	ENSMUSG00000062329
novel_circ_0000016	mmu-let-7g-5p	ENSMUST00000073554	ENSMUSG00000062329
novel_circ_0000022	mmu-let-7g-5p	ENSMUST00000073554	ENSMUSG00000062329
novel_circ_0000093	mmu-let-7g-5p	ENSMUST00000073554	ENSMUSG00000062329
novel_circ_0000128	mmu-let-7g-5p	ENSMUST00000073554	ENSMUSG00000062329
novel_circ_0000154	mmu-let-7g-5p	ENSMUST00000073554	ENSMUSG00000062329
novel_circ_0000484	mmu-let-7g-5p	ENSMUST00000073554	ENSMUSG00000062329
novel_circ_0000521	mmu-let-7g-5p	ENSMUST00000073554	ENSMUSG00000062329
novel_circ_0000525	mmu-let-7g-5p	ENSMUST00000073554	ENSMUSG00000062329
novel_circ_0000534	mmu-let-7g-5p	ENSMUST00000073554	ENSMUSG00000062329
novel_circ_0000589	mmu-let-7g-5p	ENSMUST00000073554	ENSMUSG00000062329
novel_circ_0000605	mmu-let-7g-5p	ENSMUST00000073554	ENSMUSG00000062329
novel_circ_0000606	mmu-let-7g-5p	ENSMUST00000073554	ENSMUSG00000062329
novel_circ_0000987	mmu-let-7g-5p	ENSMUST00000073554	ENSMUSG00000062329
novel_circ_0001163	mmu-let-7g-5p	ENSMUST00000073554	ENSMUSG00000062329
novel_circ_0001223	mmu-let-7g-3p	ENSMUST00000063750	ENSMUSG00000017491
novel_circ_0001223	mmu-let-7g-3p	ENSMUST00000225921	ENSMUSG00000017491
novel_circ_0001228	mmu-let-7g-3p	ENSMUST00000063750	ENSMUSG00000017491
novel_circ_0001228	mmu-let-7g-3p	ENSMUST00000225921	ENSMUSG00000017491
novel_circ_0001245	mmu-let-7g-3p	ENSMUST00000063750	ENSMUSG00000017491
novel_circ_0001245	mmu-let-7g-3p	ENSMUST00000225921	ENSMUSG00000017491
novel_circ_0001245	mmu-let-7g-3p	ENSMUST00000063750	ENSMUSG00000017491
novel_circ_0001245	mmu-let-7g-3p	ENSMUST00000225921	ENSMUSG00000017491
novel_circ_0001137	mmu-let-7g-3p	ENSMUST00000063750	ENSMUSG00000017491
novel_circ_0001137	mmu-let-7g-3p	ENSMUST00000225921	ENSMUSG00000017491
novel_circ_0001482	mmu-let-7g-3p	ENSMUST00000063750	ENSMUSG00000017491
novel_circ_0001482	mmu-let-7g-3p	ENSMUST00000225921	ENSMUSG00000017491
novel_circ_0001536	mmu-let-7g-3p	ENSMUST00000063750	ENSMUSG00000017491
novel_circ_0001536	mmu-let-7g-3p	ENSMUST00000225921	ENSMUSG00000017491
novel_circ_0001358	mmu-let-7g-3p	ENSMUST00000063750	ENSMUSG00000017491
novel_circ_0001358	mmu-let-7g-3p	ENSMUST00000225921	ENSMUSG00000017491
novel_circ_0001398	mmu-let-7g-3p	ENSMUST00000063750	ENSMUSG00000017491
novel_circ_0001398	mmu-let-7g-3p	ENSMUST00000225921	ENSMUSG00000017491
novel_circ_0001399	mmu-let-7g-3p	ENSMUST00000063750	ENSMUSG00000017491
novel_circ_0001399	mmu-let-7g-3p	ENSMUST00000225921	ENSMUSG00000017491
novel_circ_0001400	mmu-let-7g-3p	ENSMUST00000063750	ENSMUSG00000017491
novel_circ_0001400	mmu-let-7g-3p	ENSMUST00000225921	ENSMUSG00000017491
mmu_circ_0000011	mmu-let-7g-3p	ENSMUST00000063750	ENSMUSG00000017491
mmu_circ_0000011	mmu-let-7g-3p	ENSMUST00000225921	ENSMUSG00000017491
mmu_circ_0000038	mmu-let-7g-3p	ENSMUST00000063750	ENSMUSG00000017491
mmu_circ_0000038	mmu-let-7g-3p	ENSMUST00000225921	ENSMUSG00000017491
mmu_circ_0000048	mmu-let-7g-3p	ENSMUST00000063750	ENSMUSG00000017491
mmu_circ_0000048	mmu-let-7g-3p	ENSMUST00000225921	ENSMUSG00000017491
mmu_circ_0000049	mmu-let-7g-3p	ENSMUST00000063750	ENSMUSG00000017491

mmu_circ_0000049	mmu-let-7g-3p	ENSMUST00000225921	ENSMUSG00000017491
mmu_circ_0001123	mmu-let-7g-3p	ENSMUST00000063750	ENSMUSG00000017491
mmu_circ_0001123	mmu-let-7g-3p	ENSMUST00000225921	ENSMUSG00000017491
mmu_circ_0001017	mmu-let-7g-3p	ENSMUST00000063750	ENSMUSG00000017491
mmu_circ_0001017	mmu-let-7g-3p	ENSMUST00000225921	ENSMUSG00000017491
mmu_circ_0001368	mmu-let-7g-3p	ENSMUST00000063750	ENSMUSG00000017491
mmu_circ_0001368	mmu-let-7g-3p	ENSMUST00000225921	ENSMUSG00000017491
mmu_circ_0001369	mmu-let-7g-3p	ENSMUST00000063750	ENSMUSG00000017491
mmu_circ_0001369	mmu-let-7g-3p	ENSMUST00000225921	ENSMUSG00000017491
mmu_circ_0001376	mmu-let-7g-3p	ENSMUST00000063750	ENSMUSG00000017491
mmu_circ_0001376	mmu-let-7g-3p	ENSMUST00000225921	ENSMUSG00000017491
mmu_circ_0001387	mmu-let-7g-3p	ENSMUST00000063750	ENSMUSG00000017491
mmu_circ_0001387	mmu-let-7g-3p	ENSMUST00000225921	ENSMUSG00000017491
mmu_circ_0001333	mmu-let-7g-3p	ENSMUST00000063750	ENSMUSG00000017491
mmu_circ_0001333	mmu-let-7g-3p	ENSMUST00000225921	ENSMUSG00000017491
mmu_circ_0001336	mmu-let-7g-3p	ENSMUST00000063750	ENSMUSG00000017491
mmu_circ_0001336	mmu-let-7g-3p	ENSMUST00000225921	ENSMUSG00000017491
mmu_circ_0001345	mmu-let-7g-3p	ENSMUST00000063750	ENSMUSG00000017491
mmu_circ_0001345	mmu-let-7g-3p	ENSMUST00000225921	ENSMUSG00000017491
mmu_circ_0001248	mmu-let-7g-3p	ENSMUST00000063750	ENSMUSG00000017491
mmu_circ_0001248	mmu-let-7g-3p	ENSMUST00000225921	ENSMUSG00000017491
mmu_circ_0001249	mmu-let-7g-3p	ENSMUST00000063750	ENSMUSG00000017491
mmu_circ_0001249	mmu-let-7g-3p	ENSMUST00000225921	ENSMUSG00000017491
mmu_circ_0001263	mmu-let-7g-3p	ENSMUST00000063750	ENSMUSG00000017491
mmu_circ_0001263	mmu-let-7g-3p	ENSMUST00000225921	ENSMUSG00000017491
mmu_circ_0001264	mmu-let-7g-3p	ENSMUST00000063750	ENSMUSG00000017491
mmu_circ_0001264	mmu-let-7g-3p	ENSMUST00000225921	ENSMUSG00000017491
mmu_circ_0001620	mmu-let-7g-3p	ENSMUST00000063750	ENSMUSG00000017491
mmu_circ_0001620	mmu-let-7g-3p	ENSMUST00000225921	ENSMUSG00000017491
mmu_circ_0001518	mmu-let-7g-3p	ENSMUST00000063750	ENSMUSG00000017491
mmu_circ_0001518	mmu-let-7g-3p	ENSMUST00000225921	ENSMUSG00000017491
mmu_circ_0001454	mmu-let-7g-3p	ENSMUST00000063750	ENSMUSG00000017491
mmu_circ_0001454	mmu-let-7g-3p	ENSMUST00000225921	ENSMUSG00000017491
mmu_circ_0001500	mmu-let-7g-3p	ENSMUST00000063750	ENSMUSG00000017491
mmu_circ_0001500	mmu-let-7g-3p	ENSMUST00000225921	ENSMUSG00000017491
mmu_circ_0001842	mmu-let-7g-3p	ENSMUST00000063750	ENSMUSG00000017491
mmu_circ_0001842	mmu-let-7g-3p	ENSMUST00000225921	ENSMUSG00000017491
mmu_circ_0001775	mmu-let-7g-3p	ENSMUST00000063750	ENSMUSG00000017491
mmu_circ_0001775	mmu-let-7g-3p	ENSMUST00000225921	ENSMUSG00000017491
mmu_circ_0001827	mmu-let-7g-3p	ENSMUST00000063750	ENSMUSG00000017491
mmu_circ_0001827	mmu-let-7g-3p	ENSMUST00000225921	ENSMUSG00000017491
mmu_circ_0001692	mmu-let-7g-3p	ENSMUST00000063750	ENSMUSG00000017491
mmu_circ_0001692	mmu-let-7g-3p	ENSMUST00000225921	ENSMUSG00000017491
mmu_circ_0001692	mmu-let-7g-3p	ENSMUST00000063750	ENSMUSG00000017491
mmu_circ_0001692	mmu-let-7g-3p	ENSMUST00000225921	ENSMUSG00000017491
mmu_circ_0000230	mmu-let-7g-3p	ENSMUST00000063750	ENSMUSG00000017491
mmu_circ_0000230	mmu-let-7g-3p	ENSMUST00000225921	ENSMUSG00000017491
mmu_circ_0000301	mmu-let-7g-3p	ENSMUST00000063750	ENSMUSG00000017491
mmu_circ_0000301	mmu-let-7g-3p	ENSMUST00000225921	ENSMUSG00000017491
mmu_circ_0000234	mmu-let-7g-3p	ENSMUST00000063750	ENSMUSG00000017491
mmu_circ_0000234	mmu-let-7g-3p	ENSMUST00000225921	ENSMUSG00000017491
mmu_circ_0000498	mmu-let-7g-3p	ENSMUST00000063750	ENSMUSG00000017491
mmu_circ_0000498	mmu-let-7g-3p	ENSMUST00000225921	ENSMUSG00000017491
mmu_circ_0000454	mmu-let-7g-3p	ENSMUST00000063750	ENSMUSG00000017491
mmu_circ_0000454	mmu-let-7g-3p	ENSMUST00000225921	ENSMUSG00000017491
mmu_circ_0000465	mmu-let-7g-3p	ENSMUST00000063750	ENSMUSG00000017491
mmu_circ_0000465	mmu-let-7g-3p	ENSMUST00000225921	ENSMUSG00000017491
mmu_circ_0000345	mmu-let-7g-3p	ENSMUST00000063750	ENSMUSG00000017491

mmu_circ_0000345	mmu-let-7g-3p	ENSMUST00000225921	ENSMUSG00000017491
mmu_circ_0000588	mmu-let-7g-3p	ENSMUST00000063750	ENSMUSG00000017491
mmu_circ_0000588	mmu-let-7g-3p	ENSMUST00000225921	ENSMUSG00000017491
mmu_circ_0000628	mmu-let-7g-3p	ENSMUST00000063750	ENSMUSG00000017491
mmu_circ_0000628	mmu-let-7g-3p	ENSMUST00000225921	ENSMUSG00000017491
mmu_circ_0000644	mmu-let-7g-3p	ENSMUST00000063750	ENSMUSG00000017491
mmu_circ_0000644	mmu-let-7g-3p	ENSMUST00000225921	ENSMUSG00000017491
mmu_circ_0000530	mmu-let-7g-3p	ENSMUST00000063750	ENSMUSG00000017491
mmu_circ_0000530	mmu-let-7g-3p	ENSMUST00000225921	ENSMUSG00000017491
mmu_circ_0000829	mmu-let-7g-3p	ENSMUST00000063750	ENSMUSG00000017491
mmu_circ_0000829	mmu-let-7g-3p	ENSMUST00000225921	ENSMUSG00000017491
mmu_circ_0000668	mmu-let-7g-3p	ENSMUST00000063750	ENSMUSG00000017491
mmu_circ_0000668	mmu-let-7g-3p	ENSMUST00000225921	ENSMUSG00000017491
mmu_circ_0000712	mmu-let-7g-3p	ENSMUST00000063750	ENSMUSG00000017491
mmu_circ_0000712	mmu-let-7g-3p	ENSMUST00000225921	ENSMUSG00000017491
mmu_circ_0000713	mmu-let-7g-3p	ENSMUST00000063750	ENSMUSG00000017491
mmu_circ_0000713	mmu-let-7g-3p	ENSMUST00000225921	ENSMUSG00000017491
mmu_circ_0000714	mmu-let-7g-3p	ENSMUST00000063750	ENSMUSG00000017491
mmu_circ_0000714	mmu-let-7g-3p	ENSMUST00000225921	ENSMUSG00000017491
mmu_circ_0000969	mmu-let-7g-3p	ENSMUST00000063750	ENSMUSG00000017491
mmu_circ_0000969	mmu-let-7g-3p	ENSMUST00000225921	ENSMUSG00000017491
mmu_circ_0000905	mmu-let-7g-3p	ENSMUST00000063750	ENSMUSG00000017491
mmu_circ_0000905	mmu-let-7g-3p	ENSMUST00000225921	ENSMUSG00000017491
mmu_circ_0000906	mmu-let-7g-3p	ENSMUST00000063750	ENSMUSG00000017491
mmu_circ_0000906	mmu-let-7g-3p	ENSMUST00000225921	ENSMUSG00000017491
mmu_circ_0000907	mmu-let-7g-3p	ENSMUST00000063750	ENSMUSG00000017491
mmu_circ_0000907	mmu-let-7g-3p	ENSMUST00000225921	ENSMUSG00000017491
mmu_circ_0000912	mmu-let-7g-3p	ENSMUST00000063750	ENSMUSG00000017491
mmu_circ_0000912	mmu-let-7g-3p	ENSMUST00000225921	ENSMUSG00000017491
novel_circ_0001551	mmu-let-7g-3p	ENSMUST00000063750	ENSMUSG00000017491
novel_circ_0001551	mmu-let-7g-3p	ENSMUST00000225921	ENSMUSG00000017491
novel_circ_0001561	mmu-let-7g-3p	ENSMUST00000063750	ENSMUSG00000017491
novel_circ_0001561	mmu-let-7g-3p	ENSMUST00000225921	ENSMUSG00000017491
novel_circ_0001567	mmu-let-7g-3p	ENSMUST00000063750	ENSMUSG00000017491
novel_circ_0001567	mmu-let-7g-3p	ENSMUST00000225921	ENSMUSG00000017491
novel_circ_0001603	mmu-let-7g-3p	ENSMUST00000063750	ENSMUSG00000017491
novel_circ_0001603	mmu-let-7g-3p	ENSMUST00000225921	ENSMUSG00000017491
novel_circ_0001625	mmu-let-7g-3p	ENSMUST00000063750	ENSMUSG00000017491
novel_circ_0001625	mmu-let-7g-3p	ENSMUST00000225921	ENSMUSG00000017491
novel_circ_0001626	mmu-let-7g-3p	ENSMUST00000063750	ENSMUSG00000017491
novel_circ_0001626	mmu-let-7g-3p	ENSMUST00000225921	ENSMUSG00000017491
novel_circ_0002192	mmu-let-7g-3p	ENSMUST00000063750	ENSMUSG00000017491
novel_circ_0002192	mmu-let-7g-3p	ENSMUST00000225921	ENSMUSG00000017491
novel_circ_0002192	mmu-let-7g-3p	ENSMUST00000063750	ENSMUSG00000017491
novel_circ_0002192	mmu-let-7g-3p	ENSMUST00000225921	ENSMUSG00000017491
novel_circ_0001916	mmu-let-7g-3p	ENSMUST00000063750	ENSMUSG00000017491
novel_circ_0001916	mmu-let-7g-3p	ENSMUST00000225921	ENSMUSG00000017491
novel_circ_0001916	mmu-let-7g-3p	ENSMUST00000063750	ENSMUSG00000017491
novel_circ_0001916	mmu-let-7g-3p	ENSMUST00000225921	ENSMUSG00000017491
novel_circ_0002455	mmu-let-7g-3p	ENSMUST00000063750	ENSMUSG00000017491
novel_circ_0002455	mmu-let-7g-3p	ENSMUST00000225921	ENSMUSG00000017491
novel_circ_0002456	mmu-let-7g-3p	ENSMUST00000063750	ENSMUSG00000017491
novel_circ_0002456	mmu-let-7g-3p	ENSMUST00000225921	ENSMUSG00000017491
novel_circ_0002518	mmu-let-7g-3p	ENSMUST00000063750	ENSMUSG00000017491
novel_circ_0002518	mmu-let-7g-3p	ENSMUST00000225921	ENSMUSG00000017491
novel_circ_0002547	mmu-let-7g-3p	ENSMUST00000063750	ENSMUSG00000017491
novel_circ_0002547	mmu-let-7g-3p	ENSMUST00000225921	ENSMUSG00000017491
novel_circ_0002557	mmu-let-7g-3p	ENSMUST00000063750	ENSMUSG00000017491

novel_circ_0002557	mmu-let-7g-3p	ENSMUST00000225921	ENSMUSG00000017491
novel_circ_0002849	mmu-let-7g-3p	ENSMUST00000063750	ENSMUSG00000017491
novel_circ_0002849	mmu-let-7g-3p	ENSMUST00000225921	ENSMUSG00000017491
novel_circ_0002673	mmu-let-7g-3p	ENSMUST00000063750	ENSMUSG00000017491
novel_circ_0002673	mmu-let-7g-3p	ENSMUST00000225921	ENSMUSG00000017491
novel_circ_0002719	mmu-let-7g-3p	ENSMUST00000063750	ENSMUSG00000017491
novel_circ_0002719	mmu-let-7g-3p	ENSMUST00000225921	ENSMUSG00000017491
novel_circ_0002719	mmu-let-7g-3p	ENSMUST00000063750	ENSMUSG00000017491
novel_circ_0002719	mmu-let-7g-3p	ENSMUST00000225921	ENSMUSG00000017491
novel_circ_0002728	mmu-let-7g-3p	ENSMUST00000063750	ENSMUSG00000017491
novel_circ_0002728	mmu-let-7g-3p	ENSMUST00000225921	ENSMUSG00000017491
novel_circ_0002728	mmu-let-7g-3p	ENSMUST00000063750	ENSMUSG00000017491
novel_circ_0002728	mmu-let-7g-3p	ENSMUST00000225921	ENSMUSG00000017491
novel_circ_0002729	mmu-let-7g-3p	ENSMUST00000063750	ENSMUSG00000017491
novel_circ_0002729	mmu-let-7g-3p	ENSMUST00000225921	ENSMUSG00000017491
novel_circ_0002729	mmu-let-7g-3p	ENSMUST00000063750	ENSMUSG00000017491
novel_circ_0002729	mmu-let-7g-3p	ENSMUST00000225921	ENSMUSG00000017491
novel_circ_0003187	mmu-let-7g-3p	ENSMUST00000063750	ENSMUSG00000017491
novel_circ_0003187	mmu-let-7g-3p	ENSMUST00000225921	ENSMUSG00000017491
novel_circ_0003187	mmu-let-7g-3p	ENSMUST00000063750	ENSMUSG00000017491
novel_circ_0003187	mmu-let-7g-3p	ENSMUST00000225921	ENSMUSG00000017491
novel_circ_0003189	mmu-let-7g-3p	ENSMUST00000063750	ENSMUSG00000017491
novel_circ_0003189	mmu-let-7g-3p	ENSMUST00000225921	ENSMUSG00000017491
novel_circ_0003189	mmu-let-7g-3p	ENSMUST00000063750	ENSMUSG00000017491
novel_circ_0003189	mmu-let-7g-3p	ENSMUST00000225921	ENSMUSG00000017491
novel_circ_0003083	mmu-let-7g-3p	ENSMUST00000063750	ENSMUSG00000017491
novel_circ_0003083	mmu-let-7g-3p	ENSMUST00000225921	ENSMUSG00000017491
novel_circ_0003433	mmu-let-7g-3p	ENSMUST00000063750	ENSMUSG00000017491
novel_circ_0003433	mmu-let-7g-3p	ENSMUST00000225921	ENSMUSG00000017491
novel_circ_0003485	mmu-let-7g-3p	ENSMUST00000063750	ENSMUSG00000017491
novel_circ_0003485	mmu-let-7g-3p	ENSMUST00000225921	ENSMUSG00000017491
novel_circ_0003503	mmu-let-7g-3p	ENSMUST00000063750	ENSMUSG00000017491
novel_circ_0003503	mmu-let-7g-3p	ENSMUST00000225921	ENSMUSG00000017491
novel_circ_0000186	mmu-let-7g-3p	ENSMUST00000063750	ENSMUSG00000017491
novel_circ_0000186	mmu-let-7g-3p	ENSMUST00000225921	ENSMUSG00000017491
novel_circ_0000200	mmu-let-7g-3p	ENSMUST00000063750	ENSMUSG00000017491
novel_circ_0000200	mmu-let-7g-3p	ENSMUST00000225921	ENSMUSG00000017491
novel_circ_0000023	mmu-let-7g-3p	ENSMUST00000063750	ENSMUSG00000017491
novel_circ_0000023	mmu-let-7g-3p	ENSMUST00000225921	ENSMUSG00000017491
novel_circ_0000023	mmu-let-7g-3p	ENSMUST00000063750	ENSMUSG00000017491
novel_circ_0000023	mmu-let-7g-3p	ENSMUST00000225921	ENSMUSG00000017491
novel_circ_0000029	mmu-let-7g-3p	ENSMUST00000063750	ENSMUSG00000017491
novel_circ_0000029	mmu-let-7g-3p	ENSMUST00000225921	ENSMUSG00000017491
novel_circ_0000029	mmu-let-7g-3p	ENSMUST00000063750	ENSMUSG00000017491
novel_circ_0000029	mmu-let-7g-3p	ENSMUST00000225921	ENSMUSG00000017491
novel_circ_0000083	mmu-let-7g-3p	ENSMUST00000063750	ENSMUSG00000017491
novel_circ_0000083	mmu-let-7g-3p	ENSMUST00000225921	ENSMUSG00000017491
novel_circ_0000095	mmu-let-7g-3p	ENSMUST00000063750	ENSMUSG00000017491
novel_circ_0000095	mmu-let-7g-3p	ENSMUST00000225921	ENSMUSG00000017491
novel_circ_0000632	mmu-let-7g-3p	ENSMUST00000063750	ENSMUSG00000017491
novel_circ_0000632	mmu-let-7g-3p	ENSMUST00000225921	ENSMUSG00000017491
novel_circ_0000762	mmu-let-7g-3p	ENSMUST00000063750	ENSMUSG00000017491
novel_circ_0000762	mmu-let-7g-3p	ENSMUST00000225921	ENSMUSG00000017491
novel_circ_0000780	mmu-let-7g-3p	ENSMUST00000063750	ENSMUSG00000017491
novel_circ_0000780	mmu-let-7g-3p	ENSMUST00000225921	ENSMUSG00000017491
novel_circ_0000567	mmu-let-7g-3p	ENSMUST00000063750	ENSMUSG00000017491
novel_circ_0000567	mmu-let-7g-3p	ENSMUST00000225921	ENSMUSG00000017491
novel_circ_0000567	mmu-let-7g-3p	ENSMUST00000063750	ENSMUSG00000017491

novel_circ_0000567	mmu-let-7g-3p	ENSMUST00000225921	ENSMUSG00000017491
novel_circ_0000975	mmu-let-7g-3p	ENSMUST00000063750	ENSMUSG00000017491
novel_circ_0000975	mmu-let-7g-3p	ENSMUST00000225921	ENSMUSG00000017491
novel_circ_0001039	mmu-let-7g-3p	ENSMUST00000063750	ENSMUSG00000017491
novel_circ_0001039	mmu-let-7g-3p	ENSMUST00000225921	ENSMUSG00000017491
novel_circ_0001207	mmu-let-7g-3p	ENSMUST00000063750	ENSMUSG00000017491
novel_circ_0001207	mmu-let-7g-3p	ENSMUST00000225921	ENSMUSG00000017491
novel_circ_0001208	mmu-let-7g-3p	ENSMUST00000063750	ENSMUSG00000017491
novel_circ_0001208	mmu-let-7g-3p	ENSMUST00000225921	ENSMUSG00000017491
novel_circ_0001119	mmu-miR-128-1-	ENSMUST00000213072	ENSMUSG00000031849
novel_circ_0001141	mmu-miR-128-1-	ENSMUST00000213072	ENSMUSG00000031849
novel_circ_0001142	mmu-miR-128-1-	ENSMUST00000213072	ENSMUSG00000031849
novel_circ_0001142	mmu-miR-128-1-	ENSMUST00000213072	ENSMUSG00000031849
novel_circ_0001501	mmu-miR-128-1-	ENSMUST00000213072	ENSMUSG00000031849
mmu_circ_0000052	mmu-miR-128-1-	ENSMUST00000213072	ENSMUSG00000031849
mmu_circ_0001123	mmu-miR-128-1-	ENSMUST00000213072	ENSMUSG00000031849
mmu_circ_0001376	mmu-miR-128-1-	ENSMUST00000213072	ENSMUSG00000031849
mmu_circ_0001732	mmu-miR-128-1-	ENSMUST00000213072	ENSMUSG00000031849
mmu_circ_0001718	mmu-miR-128-1-	ENSMUST00000213072	ENSMUSG00000031849
mmu_circ_0001894	mmu-miR-128-1-	ENSMUST00000213072	ENSMUSG00000031849
mmu_circ_0000215	mmu-miR-128-1-	ENSMUST00000213072	ENSMUSG00000031849
mmu_circ_0000831	mmu-miR-128-1-	ENSMUST00000213072	ENSMUSG00000031849
novel_circ_0001908	mmu-miR-128-1-	ENSMUST00000213072	ENSMUSG00000031849
novel_circ_0001943	mmu-miR-128-1-	ENSMUST00000213072	ENSMUSG00000031849
novel_circ_0002494	mmu-miR-128-1-	ENSMUST00000213072	ENSMUSG00000031849
novel_circ_0002246	mmu-miR-128-1-	ENSMUST00000213072	ENSMUSG00000031849
novel_circ_0002871	mmu-miR-128-1-	ENSMUST00000213072	ENSMUSG00000031849
novel_circ_0002673	mmu-miR-128-1-	ENSMUST00000213072	ENSMUSG00000031849
novel_circ_0002673	mmu-miR-128-1-	ENSMUST00000213072	ENSMUSG00000031849
novel_circ_0002675	mmu-miR-128-1-	ENSMUST00000213072	ENSMUSG00000031849
novel_circ_0003036	mmu-miR-128-1-	ENSMUST00000213072	ENSMUSG00000031849
novel_circ_0003456	mmu-miR-128-1-	ENSMUST00000213072	ENSMUSG00000031849
novel_circ_0003456	mmu-miR-128-1-	ENSMUST00000213072	ENSMUSG00000031849
novel_circ_0000301	mmu-miR-128-1-	ENSMUST00000213072	ENSMUSG00000031849
novel_circ_0000474	mmu-miR-128-1-	ENSMUST00000213072	ENSMUSG00000031849
novel_circ_0000527	mmu-miR-128-1-	ENSMUST00000213072	ENSMUSG00000031849
novel_circ_0000594	mmu-miR-128-1-	ENSMUST00000213072	ENSMUSG00000031849
novel_circ_0001213	mmu-miR-128-1-	ENSMUST00000213072	ENSMUSG00000031849
novel_circ_0001214	mmu-miR-128-1-	ENSMUST00000213072	ENSMUSG00000031849
novel_circ_0001245	mmu-miR-140-3p	ENSMUST00000003659	ENSMUSG00000031849
novel_circ_0001245	mmu-miR-140-3p	ENSMUST00000212488	ENSMUSG00000031849
novel_circ_0001251	mmu-miR-140-3p	ENSMUST00000003659	ENSMUSG00000031849
novel_circ_0001251	mmu-miR-140-3p	ENSMUST00000212488	ENSMUSG00000031849
novel_circ_0001277	mmu-miR-140-3p	ENSMUST00000003659	ENSMUSG00000031849
novel_circ_0001277	mmu-miR-140-3p	ENSMUST00000212488	ENSMUSG00000031849
novel_circ_0001292	mmu-miR-140-3p	ENSMUST00000003659	ENSMUSG00000031849
novel_circ_0001292	mmu-miR-140-3p	ENSMUST00000212488	ENSMUSG00000031849
novel_circ_0001084	mmu-miR-140-3p	ENSMUST00000003659	ENSMUSG00000031849
novel_circ_0001084	mmu-miR-140-3p	ENSMUST00000212488	ENSMUSG00000031849
novel_circ_0001114	mmu-miR-140-3p	ENSMUST00000003659	ENSMUSG00000031849
novel_circ_0001114	mmu-miR-140-3p	ENSMUST00000212488	ENSMUSG00000031849
novel_circ_0001115	mmu-miR-140-3p	ENSMUST00000003659	ENSMUSG00000031849
novel_circ_0001115	mmu-miR-140-3p	ENSMUST00000212488	ENSMUSG00000031849
novel_circ_0001137	mmu-miR-140-3p	ENSMUST00000003659	ENSMUSG00000031849
novel_circ_0001137	mmu-miR-140-3p	ENSMUST00000212488	ENSMUSG00000031849
novel_circ_0001458	mmu-miR-140-3p	ENSMUST00000003659	ENSMUSG00000031849
novel_circ_0001458	mmu-miR-140-3p	ENSMUST00000212488	ENSMUSG00000031849
novel_circ_0001371	mmu-miR-140-3p	ENSMUST00000003659	ENSMUSG00000031849

novel_circ_0001371	mmu-miR-140-3p	ENSMUST00000212488	ENSMUSG00000031849
novel_circ_0001374	mmu-miR-140-3p	ENSMUST00000003659	ENSMUSG00000031849
novel_circ_0001374	mmu-miR-140-3p	ENSMUST00000212488	ENSMUSG00000031849
novel_circ_0001414	mmu-miR-140-3p	ENSMUST00000003659	ENSMUSG00000031849
novel_circ_0001414	mmu-miR-140-3p	ENSMUST00000212488	ENSMUSG00000031849
novel_circ_0001430	mmu-miR-140-3p	ENSMUST00000003659	ENSMUSG00000031849
novel_circ_0001430	mmu-miR-140-3p	ENSMUST00000212488	ENSMUSG00000031849
mmu_circ_0000097	mmu-miR-140-3p	ENSMUST00000003659	ENSMUSG00000031849
mmu_circ_0000097	mmu-miR-140-3p	ENSMUST00000212488	ENSMUSG00000031849
mmu_circ_0000114	mmu-miR-140-3p	ENSMUST00000003659	ENSMUSG00000031849
mmu_circ_0000114	mmu-miR-140-3p	ENSMUST00000212488	ENSMUSG00000031849
mmu_circ_0000063	mmu-miR-140-3p	ENSMUST00000003659	ENSMUSG00000031849
mmu_circ_0000063	mmu-miR-140-3p	ENSMUST00000212488	ENSMUSG00000031849
mmu_circ_0001162	mmu-miR-140-3p	ENSMUST00000003659	ENSMUSG00000031849
mmu_circ_0001162	mmu-miR-140-3p	ENSMUST00000212488	ENSMUSG00000031849
mmu_circ_0001167	mmu-miR-140-3p	ENSMUST00000003659	ENSMUSG00000031849
mmu_circ_0001167	mmu-miR-140-3p	ENSMUST00000212488	ENSMUSG00000031849
mmu_circ_0001123	mmu-miR-140-3p	ENSMUST00000003659	ENSMUSG00000031849
mmu_circ_0001123	mmu-miR-140-3p	ENSMUST00000212488	ENSMUSG00000031849
mmu_circ_0001135	mmu-miR-140-3p	ENSMUST00000003659	ENSMUSG00000031849
mmu_circ_0001135	mmu-miR-140-3p	ENSMUST00000212488	ENSMUSG00000031849
mmu_circ_0001135	mmu-miR-140-3p	ENSMUST00000003659	ENSMUSG00000031849
mmu_circ_0001135	mmu-miR-140-3p	ENSMUST00000212488	ENSMUSG00000031849
mmu_circ_0001148	mmu-miR-140-3p	ENSMUST00000003659	ENSMUSG00000031849
mmu_circ_0001148	mmu-miR-140-3p	ENSMUST00000212488	ENSMUSG00000031849
mmu_circ_0001103	mmu-miR-140-3p	ENSMUST00000003659	ENSMUSG00000031849
mmu_circ_0001103	mmu-miR-140-3p	ENSMUST00000212488	ENSMUSG00000031849
mmu_circ_0001103	mmu-miR-140-3p	ENSMUST00000003659	ENSMUSG00000031849
mmu_circ_0001103	mmu-miR-140-3p	ENSMUST00000212488	ENSMUSG00000031849
mmu_circ_0001369	mmu-miR-140-3p	ENSMUST00000003659	ENSMUSG00000031849
mmu_circ_0001369	mmu-miR-140-3p	ENSMUST00000212488	ENSMUSG00000031849
mmu_circ_0001369	mmu-miR-140-3p	ENSMUST00000003659	ENSMUSG00000031849
mmu_circ_0001369	mmu-miR-140-3p	ENSMUST00000212488	ENSMUSG00000031849
mmu_circ_0001376	mmu-miR-140-3p	ENSMUST00000003659	ENSMUSG00000031849
mmu_circ_0001376	mmu-miR-140-3p	ENSMUST00000212488	ENSMUSG00000031849
mmu_circ_0001421	mmu-miR-140-3p	ENSMUST00000003659	ENSMUSG00000031849
mmu_circ_0001421	mmu-miR-140-3p	ENSMUST00000212488	ENSMUSG00000031849
mmu_circ_0001331	mmu-miR-140-3p	ENSMUST00000003659	ENSMUSG00000031849
mmu_circ_0001331	mmu-miR-140-3p	ENSMUST00000212488	ENSMUSG00000031849
mmu_circ_0001333	mmu-miR-140-3p	ENSMUST00000003659	ENSMUSG00000031849
mmu_circ_0001333	mmu-miR-140-3p	ENSMUST00000212488	ENSMUSG00000031849
mmu_circ_0001291	mmu-miR-140-3p	ENSMUST00000003659	ENSMUSG00000031849
mmu_circ_0001291	mmu-miR-140-3p	ENSMUST00000212488	ENSMUSG00000031849
mmu_circ_0001507	mmu-miR-140-3p	ENSMUST00000003659	ENSMUSG00000031849
mmu_circ_0001507	mmu-miR-140-3p	ENSMUST00000212488	ENSMUSG00000031849
mmu_circ_0001528	mmu-miR-140-3p	ENSMUST00000003659	ENSMUSG00000031849
mmu_circ_0001528	mmu-miR-140-3p	ENSMUST00000212488	ENSMUSG00000031849
mmu_circ_0001454	mmu-miR-140-3p	ENSMUST00000003659	ENSMUSG00000031849
mmu_circ_0001454	mmu-miR-140-3p	ENSMUST00000212488	ENSMUSG00000031849
mmu_circ_0001499	mmu-miR-140-3p	ENSMUST00000003659	ENSMUSG00000031849
mmu_circ_0001499	mmu-miR-140-3p	ENSMUST00000212488	ENSMUSG00000031849
mmu_circ_0001500	mmu-miR-140-3p	ENSMUST00000003659	ENSMUSG00000031849
mmu_circ_0001500	mmu-miR-140-3p	ENSMUST00000212488	ENSMUSG00000031849
mmu_circ_0001774	mmu-miR-140-3p	ENSMUST00000003659	ENSMUSG00000031849
mmu_circ_0001774	mmu-miR-140-3p	ENSMUST00000212488	ENSMUSG00000031849
mmu_circ_0001795	mmu-miR-140-3p	ENSMUST00000003659	ENSMUSG00000031849
mmu_circ_0001795	mmu-miR-140-3p	ENSMUST00000212488	ENSMUSG00000031849
mmu_circ_0001818	mmu-miR-140-3p	ENSMUST00000003659	ENSMUSG00000031849

mmu_circ_0001818	mmu-miR-140-3p	ENSMUST00000212488	ENSMUSG00000031849
mmu_circ_0001743	mmu-miR-140-3p	ENSMUST00000003659	ENSMUSG00000031849
mmu_circ_0001743	mmu-miR-140-3p	ENSMUST00000212488	ENSMUSG00000031849
mmu_circ_0001665	mmu-miR-140-3p	ENSMUST00000003659	ENSMUSG00000031849
mmu_circ_0001665	mmu-miR-140-3p	ENSMUST00000212488	ENSMUSG00000031849
mmu_circ_0000239	mmu-miR-140-3p	ENSMUST00000003659	ENSMUSG00000031849
mmu_circ_0000239	mmu-miR-140-3p	ENSMUST00000212488	ENSMUSG00000031849
mmu_circ_0000499	mmu-miR-140-3p	ENSMUST00000003659	ENSMUSG00000031849
mmu_circ_0000499	mmu-miR-140-3p	ENSMUST00000212488	ENSMUSG00000031849
mmu_circ_0000500	mmu-miR-140-3p	ENSMUST00000003659	ENSMUSG00000031849
mmu_circ_0000500	mmu-miR-140-3p	ENSMUST00000212488	ENSMUSG00000031849
mmu_circ_0000477	mmu-miR-140-3p	ENSMUST00000003659	ENSMUSG00000031849
mmu_circ_0000477	mmu-miR-140-3p	ENSMUST00000212488	ENSMUSG00000031849
mmu_circ_0000399	mmu-miR-140-3p	ENSMUST00000003659	ENSMUSG00000031849
mmu_circ_0000399	mmu-miR-140-3p	ENSMUST00000212488	ENSMUSG00000031849
mmu_circ_0000588	mmu-miR-140-3p	ENSMUST00000003659	ENSMUSG00000031849
mmu_circ_0000588	mmu-miR-140-3p	ENSMUST00000212488	ENSMUSG00000031849
mmu_circ_0000588	mmu-miR-140-3p	ENSMUST00000003659	ENSMUSG00000031849
mmu_circ_0000588	mmu-miR-140-3p	ENSMUST00000212488	ENSMUSG00000031849
mmu_circ_0000628	mmu-miR-140-3p	ENSMUST00000003659	ENSMUSG00000031849
mmu_circ_0000628	mmu-miR-140-3p	ENSMUST00000212488	ENSMUSG00000031849
mmu_circ_0000635	mmu-miR-140-3p	ENSMUST00000003659	ENSMUSG00000031849
mmu_circ_0000635	mmu-miR-140-3p	ENSMUST00000212488	ENSMUSG00000031849
mmu_circ_0000644	mmu-miR-140-3p	ENSMUST00000003659	ENSMUSG00000031849
mmu_circ_0000644	mmu-miR-140-3p	ENSMUST00000212488	ENSMUSG00000031849
mmu_circ_0000507	mmu-miR-140-3p	ENSMUST00000003659	ENSMUSG00000031849
mmu_circ_0000507	mmu-miR-140-3p	ENSMUST00000212488	ENSMUSG00000031849
mmu_circ_0000530	mmu-miR-140-3p	ENSMUST00000003659	ENSMUSG00000031849
mmu_circ_0000530	mmu-miR-140-3p	ENSMUST00000212488	ENSMUSG00000031849
mmu_circ_0000795	mmu-miR-140-3p	ENSMUST00000003659	ENSMUSG00000031849
mmu_circ_0000795	mmu-miR-140-3p	ENSMUST00000212488	ENSMUSG00000031849
mmu_circ_0000804	mmu-miR-140-3p	ENSMUST00000003659	ENSMUSG00000031849
mmu_circ_0000804	mmu-miR-140-3p	ENSMUST00000212488	ENSMUSG00000031849
mmu_circ_0000812	mmu-miR-140-3p	ENSMUST00000003659	ENSMUSG00000031849
mmu_circ_0000812	mmu-miR-140-3p	ENSMUST00000212488	ENSMUSG00000031849
mmu_circ_0000828	mmu-miR-140-3p	ENSMUST00000003659	ENSMUSG00000031849
mmu_circ_0000828	mmu-miR-140-3p	ENSMUST00000212488	ENSMUSG00000031849
mmu_circ_0000829	mmu-miR-140-3p	ENSMUST00000003659	ENSMUSG00000031849
mmu_circ_0000829	mmu-miR-140-3p	ENSMUST00000212488	ENSMUSG00000031849
mmu_circ_0000693	mmu-miR-140-3p	ENSMUST00000003659	ENSMUSG00000031849
mmu_circ_0000693	mmu-miR-140-3p	ENSMUST00000212488	ENSMUSG00000031849
mmu_circ_0000711	mmu-miR-140-3p	ENSMUST00000003659	ENSMUSG00000031849
mmu_circ_0000711	mmu-miR-140-3p	ENSMUST00000212488	ENSMUSG00000031849
mmu_circ_0000713	mmu-miR-140-3p	ENSMUST00000003659	ENSMUSG00000031849
mmu_circ_0000713	mmu-miR-140-3p	ENSMUST00000212488	ENSMUSG00000031849
mmu_circ_0000714	mmu-miR-140-3p	ENSMUST00000003659	ENSMUSG00000031849
mmu_circ_0000714	mmu-miR-140-3p	ENSMUST00000212488	ENSMUSG00000031849
mmu_circ_0000944	mmu-miR-140-3p	ENSMUST00000003659	ENSMUSG00000031849
mmu_circ_0000944	mmu-miR-140-3p	ENSMUST00000212488	ENSMUSG00000031849
mmu_circ_0000950	mmu-miR-140-3p	ENSMUST00000003659	ENSMUSG00000031849
mmu_circ_0000950	mmu-miR-140-3p	ENSMUST00000212488	ENSMUSG00000031849
mmu_circ_0000962	mmu-miR-140-3p	ENSMUST00000003659	ENSMUSG00000031849
mmu_circ_0000962	mmu-miR-140-3p	ENSMUST00000212488	ENSMUSG00000031849
mmu_circ_0000969	mmu-miR-140-3p	ENSMUST00000003659	ENSMUSG00000031849
mmu_circ_0000969	mmu-miR-140-3p	ENSMUST00000212488	ENSMUSG00000031849
mmu_circ_0000857	mmu-miR-140-3p	ENSMUST00000003659	ENSMUSG00000031849
mmu_circ_0000857	mmu-miR-140-3p	ENSMUST00000212488	ENSMUSG00000031849
mmu_circ_0000862	mmu-miR-140-3p	ENSMUST00000003659	ENSMUSG00000031849

mmu_circ_0000862	mmu-miR-140-3p	ENSMUST00000212488	ENSMUSG00000031849
mmu_circ_0000876	mmu-miR-140-3p	ENSMUST00000003659	ENSMUSG00000031849
mmu_circ_0000876	mmu-miR-140-3p	ENSMUST00000212488	ENSMUSG00000031849
mmu_circ_0000884	mmu-miR-140-3p	ENSMUST00000003659	ENSMUSG00000031849
mmu_circ_0000884	mmu-miR-140-3p	ENSMUST00000212488	ENSMUSG00000031849
mmu_circ_0000886	mmu-miR-140-3p	ENSMUST00000003659	ENSMUSG00000031849
mmu_circ_0000886	mmu-miR-140-3p	ENSMUST00000212488	ENSMUSG00000031849
mmu_circ_0000906	mmu-miR-140-3p	ENSMUST00000003659	ENSMUSG00000031849
mmu_circ_0000906	mmu-miR-140-3p	ENSMUST00000212488	ENSMUSG00000031849
mmu_circ_0000907	mmu-miR-140-3p	ENSMUST00000003659	ENSMUSG00000031849
mmu_circ_0000907	mmu-miR-140-3p	ENSMUST00000212488	ENSMUSG00000031849
mmu_circ_0000908	mmu-miR-140-3p	ENSMUST00000003659	ENSMUSG00000031849
mmu_circ_0000908	mmu-miR-140-3p	ENSMUST00000212488	ENSMUSG00000031849
mmu_circ_0000912	mmu-miR-140-3p	ENSMUST00000003659	ENSMUSG00000031849
mmu_circ_0000912	mmu-miR-140-3p	ENSMUST00000212488	ENSMUSG00000031849
novel_circ_0001552	mmu-miR-140-3p	ENSMUST00000003659	ENSMUSG00000031849
novel_circ_0001552	mmu-miR-140-3p	ENSMUST00000212488	ENSMUSG00000031849
novel_circ_0001552	mmu-miR-140-3p	ENSMUST00000003659	ENSMUSG00000031849
novel_circ_0001552	mmu-miR-140-3p	ENSMUST00000212488	ENSMUSG00000031849
novel_circ_0001553	mmu-miR-140-3p	ENSMUST00000003659	ENSMUSG00000031849
novel_circ_0001553	mmu-miR-140-3p	ENSMUST00000212488	ENSMUSG00000031849
novel_circ_0001553	mmu-miR-140-3p	ENSMUST00000003659	ENSMUSG00000031849
novel_circ_0001553	mmu-miR-140-3p	ENSMUST00000212488	ENSMUSG00000031849
novel_circ_0001570	mmu-miR-140-3p	ENSMUST00000003659	ENSMUSG00000031849
novel_circ_0001570	mmu-miR-140-3p	ENSMUST00000212488	ENSMUSG00000031849
novel_circ_0001608	mmu-miR-140-3p	ENSMUST00000003659	ENSMUSG00000031849
novel_circ_0001608	mmu-miR-140-3p	ENSMUST00000212488	ENSMUSG00000031849
novel_circ_0001676	mmu-miR-140-3p	ENSMUST00000003659	ENSMUSG00000031849
novel_circ_0001676	mmu-miR-140-3p	ENSMUST00000212488	ENSMUSG00000031849
novel_circ_0002115	mmu-miR-140-3p	ENSMUST00000003659	ENSMUSG00000031849
novel_circ_0002115	mmu-miR-140-3p	ENSMUST00000212488	ENSMUSG00000031849
novel_circ_0002123	mmu-miR-140-3p	ENSMUST00000003659	ENSMUSG00000031849
novel_circ_0002123	mmu-miR-140-3p	ENSMUST00000212488	ENSMUSG00000031849
novel_circ_0002142	mmu-miR-140-3p	ENSMUST00000003659	ENSMUSG00000031849
novel_circ_0002142	mmu-miR-140-3p	ENSMUST00000212488	ENSMUSG00000031849
novel_circ_0002192	mmu-miR-140-3p	ENSMUST00000003659	ENSMUSG00000031849
novel_circ_0002192	mmu-miR-140-3p	ENSMUST00000212488	ENSMUSG00000031849
novel_circ_0002198	mmu-miR-140-3p	ENSMUST00000003659	ENSMUSG00000031849
novel_circ_0002198	mmu-miR-140-3p	ENSMUST00000212488	ENSMUSG00000031849
novel_circ_0001761	mmu-miR-140-3p	ENSMUST00000003659	ENSMUSG00000031849
novel_circ_0001761	mmu-miR-140-3p	ENSMUST00000212488	ENSMUSG00000031849
novel_circ_0001800	mmu-miR-140-3p	ENSMUST00000003659	ENSMUSG00000031849
novel_circ_0001800	mmu-miR-140-3p	ENSMUST00000212488	ENSMUSG00000031849
novel_circ_0001859	mmu-miR-140-3p	ENSMUST00000003659	ENSMUSG00000031849
novel_circ_0001859	mmu-miR-140-3p	ENSMUST00000212488	ENSMUSG00000031849
novel_circ_0001905	mmu-miR-140-3p	ENSMUST00000003659	ENSMUSG00000031849
novel_circ_0001905	mmu-miR-140-3p	ENSMUST00000212488	ENSMUSG00000031849
novel_circ_0001905	mmu-miR-140-3p	ENSMUST00000003659	ENSMUSG00000031849
novel_circ_0001905	mmu-miR-140-3p	ENSMUST00000212488	ENSMUSG00000031849
novel_circ_0001912	mmu-miR-140-3p	ENSMUST00000003659	ENSMUSG00000031849
novel_circ_0001912	mmu-miR-140-3p	ENSMUST00000212488	ENSMUSG00000031849
novel_circ_0001912	mmu-miR-140-3p	ENSMUST00000003659	ENSMUSG00000031849
novel_circ_0001912	mmu-miR-140-3p	ENSMUST00000212488	ENSMUSG00000031849
novel_circ_0002468	mmu-miR-140-3p	ENSMUST00000003659	ENSMUSG00000031849
novel_circ_0002468	mmu-miR-140-3p	ENSMUST00000212488	ENSMUSG00000031849
novel_circ_0002479	mmu-miR-140-3p	ENSMUST00000003659	ENSMUSG00000031849
novel_circ_0002479	mmu-miR-140-3p	ENSMUST00000212488	ENSMUSG00000031849
novel_circ_0002489	mmu-miR-140-3p	ENSMUST00000003659	ENSMUSG00000031849

novel_circ_0002489	mmu-miR-140-3p	ENSMUST00000212488	ENSMUSG00000031849
novel_circ_0002499	mmu-miR-140-3p	ENSMUST00000003659	ENSMUSG00000031849
novel_circ_0002499	mmu-miR-140-3p	ENSMUST00000212488	ENSMUSG00000031849
novel_circ_0002500	mmu-miR-140-3p	ENSMUST00000003659	ENSMUSG00000031849
novel_circ_0002500	mmu-miR-140-3p	ENSMUST00000212488	ENSMUSG00000031849
novel_circ_0002501	mmu-miR-140-3p	ENSMUST00000003659	ENSMUSG00000031849
novel_circ_0002501	mmu-miR-140-3p	ENSMUST00000212488	ENSMUSG00000031849
novel_circ_0002555	mmu-miR-140-3p	ENSMUST00000003659	ENSMUSG00000031849
novel_circ_0002555	mmu-miR-140-3p	ENSMUST00000212488	ENSMUSG00000031849
novel_circ_0002617	mmu-miR-140-3p	ENSMUST00000003659	ENSMUSG00000031849
novel_circ_0002617	mmu-miR-140-3p	ENSMUST00000212488	ENSMUSG00000031849
novel_circ_0002318	mmu-miR-140-3p	ENSMUST00000003659	ENSMUSG00000031849
novel_circ_0002318	mmu-miR-140-3p	ENSMUST00000212488	ENSMUSG00000031849
novel_circ_0002319	mmu-miR-140-3p	ENSMUST00000003659	ENSMUSG00000031849
novel_circ_0002319	mmu-miR-140-3p	ENSMUST00000212488	ENSMUSG00000031849
novel_circ_0002348	mmu-miR-140-3p	ENSMUST00000003659	ENSMUSG00000031849
novel_circ_0002348	mmu-miR-140-3p	ENSMUST00000212488	ENSMUSG00000031849
novel_circ_0002398	mmu-miR-140-3p	ENSMUST00000003659	ENSMUSG00000031849
novel_circ_0002398	mmu-miR-140-3p	ENSMUST00000212488	ENSMUSG00000031849
novel_circ_0002917	mmu-miR-140-3p	ENSMUST00000003659	ENSMUSG00000031849
novel_circ_0002917	mmu-miR-140-3p	ENSMUST00000212488	ENSMUSG00000031849
novel_circ_0002928	mmu-miR-140-3p	ENSMUST00000003659	ENSMUSG00000031849
novel_circ_0002928	mmu-miR-140-3p	ENSMUST00000212488	ENSMUSG00000031849
novel_circ_0002944	mmu-miR-140-3p	ENSMUST00000003659	ENSMUSG00000031849
novel_circ_0002944	mmu-miR-140-3p	ENSMUST00000212488	ENSMUSG00000031849
novel_circ_0002946	mmu-miR-140-3p	ENSMUST00000003659	ENSMUSG00000031849
novel_circ_0002946	mmu-miR-140-3p	ENSMUST00000212488	ENSMUSG00000031849
novel_circ_0002946	mmu-miR-140-3p	ENSMUST00000003659	ENSMUSG00000031849
novel_circ_0002946	mmu-miR-140-3p	ENSMUST00000212488	ENSMUSG00000031849
novel_circ_0002970	mmu-miR-140-3p	ENSMUST00000003659	ENSMUSG00000031849
novel_circ_0002970	mmu-miR-140-3p	ENSMUST00000212488	ENSMUSG00000031849
novel_circ_0002982	mmu-miR-140-3p	ENSMUST00000003659	ENSMUSG00000031849
novel_circ_0002982	mmu-miR-140-3p	ENSMUST00000212488	ENSMUSG00000031849
novel_circ_0002991	mmu-miR-140-3p	ENSMUST00000003659	ENSMUSG00000031849
novel_circ_0002991	mmu-miR-140-3p	ENSMUST00000212488	ENSMUSG00000031849
novel_circ_0002680	mmu-miR-140-3p	ENSMUST00000003659	ENSMUSG00000031849
novel_circ_0002680	mmu-miR-140-3p	ENSMUST00000212488	ENSMUSG00000031849
novel_circ_0002788	mmu-miR-140-3p	ENSMUST00000003659	ENSMUSG00000031849
novel_circ_0002788	mmu-miR-140-3p	ENSMUST00000212488	ENSMUSG00000031849
novel_circ_0003337	mmu-miR-140-3p	ENSMUST00000003659	ENSMUSG00000031849
novel_circ_0003337	mmu-miR-140-3p	ENSMUST00000212488	ENSMUSG00000031849
novel_circ_0003350	mmu-miR-140-3p	ENSMUST00000003659	ENSMUSG00000031849
novel_circ_0003350	mmu-miR-140-3p	ENSMUST00000212488	ENSMUSG00000031849
novel_circ_0003377	mmu-miR-140-3p	ENSMUST00000003659	ENSMUSG00000031849
novel_circ_0003377	mmu-miR-140-3p	ENSMUST00000212488	ENSMUSG00000031849
novel_circ_0003382	mmu-miR-140-3p	ENSMUST00000003659	ENSMUSG00000031849
novel_circ_0003382	mmu-miR-140-3p	ENSMUST00000212488	ENSMUSG00000031849
novel_circ_0003065	mmu-miR-140-3p	ENSMUST00000003659	ENSMUSG00000031849
novel_circ_0003065	mmu-miR-140-3p	ENSMUST00000212488	ENSMUSG00000031849
novel_circ_0003067	mmu-miR-140-3p	ENSMUST00000003659	ENSMUSG00000031849
novel_circ_0003067	mmu-miR-140-3p	ENSMUST00000212488	ENSMUSG00000031849
novel_circ_0003076	mmu-miR-140-3p	ENSMUST00000003659	ENSMUSG00000031849
novel_circ_0003076	mmu-miR-140-3p	ENSMUST00000212488	ENSMUSG00000031849
novel_circ_0003491	mmu-miR-140-3p	ENSMUST00000003659	ENSMUSG00000031849
novel_circ_0003491	mmu-miR-140-3p	ENSMUST00000212488	ENSMUSG00000031849
novel_circ_0003491	mmu-miR-140-3p	ENSMUST00000003659	ENSMUSG00000031849
novel_circ_0003491	mmu-miR-140-3p	ENSMUST00000212488	ENSMUSG00000031849
novel_circ_0000164	mmu-miR-140-3p	ENSMUST00000003659	ENSMUSG00000031849

novel_circ_0000164	mmu-miR-140-3p	ENSMUST00000212488	ENSMUSG00000031849
novel_circ_0000192	mmu-miR-140-3p	ENSMUST00000003659	ENSMUSG00000031849
novel_circ_0000192	mmu-miR-140-3p	ENSMUST00000212488	ENSMUSG00000031849
novel_circ_0000240	mmu-miR-140-3p	ENSMUST00000003659	ENSMUSG00000031849
novel_circ_0000240	mmu-miR-140-3p	ENSMUST00000212488	ENSMUSG00000031849
novel_circ_0000264	mmu-miR-140-3p	ENSMUST00000003659	ENSMUSG00000031849
novel_circ_0000264	mmu-miR-140-3p	ENSMUST00000212488	ENSMUSG00000031849
novel_circ_0000280	mmu-miR-140-3p	ENSMUST00000003659	ENSMUSG00000031849
novel_circ_0000280	mmu-miR-140-3p	ENSMUST00000212488	ENSMUSG00000031849
novel_circ_0000120	mmu-miR-140-3p	ENSMUST00000003659	ENSMUSG00000031849
novel_circ_0000120	mmu-miR-140-3p	ENSMUST00000212488	ENSMUSG00000031849
novel_circ_0000691	mmu-miR-140-3p	ENSMUST00000003659	ENSMUSG00000031849
novel_circ_0000691	mmu-miR-140-3p	ENSMUST00000212488	ENSMUSG00000031849
novel_circ_0000698	mmu-miR-140-3p	ENSMUST00000003659	ENSMUSG00000031849
novel_circ_0000698	mmu-miR-140-3p	ENSMUST00000212488	ENSMUSG00000031849
novel_circ_0000731	mmu-miR-140-3p	ENSMUST00000003659	ENSMUSG00000031849
novel_circ_0000731	mmu-miR-140-3p	ENSMUST00000212488	ENSMUSG00000031849
novel_circ_0000733	mmu-miR-140-3p	ENSMUST00000003659	ENSMUSG00000031849
novel_circ_0000733	mmu-miR-140-3p	ENSMUST00000212488	ENSMUSG00000031849
novel_circ_0000775	mmu-miR-140-3p	ENSMUST00000003659	ENSMUSG00000031849
novel_circ_0000775	mmu-miR-140-3p	ENSMUST00000212488	ENSMUSG00000031849
novel_circ_0000501	mmu-miR-140-3p	ENSMUST00000003659	ENSMUSG00000031849
novel_circ_0000501	mmu-miR-140-3p	ENSMUST00000212488	ENSMUSG00000031849
novel_circ_0000505	mmu-miR-140-3p	ENSMUST00000003659	ENSMUSG00000031849
novel_circ_0000505	mmu-miR-140-3p	ENSMUST00000212488	ENSMUSG00000031849
novel_circ_0000595	mmu-miR-140-3p	ENSMUST00000003659	ENSMUSG00000031849
novel_circ_0000595	mmu-miR-140-3p	ENSMUST00000212488	ENSMUSG00000031849
novel_circ_0000942	mmu-miR-140-3p	ENSMUST00000003659	ENSMUSG00000031849
novel_circ_0000942	mmu-miR-140-3p	ENSMUST00000212488	ENSMUSG00000031849
novel_circ_0000942	mmu-miR-140-3p	ENSMUST00000003659	ENSMUSG00000031849
novel_circ_0000942	mmu-miR-140-3p	ENSMUST00000212488	ENSMUSG00000031849
novel_circ_0000954	mmu-miR-140-3p	ENSMUST00000003659	ENSMUSG00000031849
novel_circ_0000954	mmu-miR-140-3p	ENSMUST00000212488	ENSMUSG00000031849
novel_circ_0000954	mmu-miR-140-3p	ENSMUST00000003659	ENSMUSG00000031849
novel_circ_0000954	mmu-miR-140-3p	ENSMUST00000212488	ENSMUSG00000031849
novel_circ_0001001	mmu-miR-140-3p	ENSMUST00000003659	ENSMUSG00000031849
novel_circ_0001001	mmu-miR-140-3p	ENSMUST00000212488	ENSMUSG00000031849
novel_circ_0001015	mmu-miR-140-3p	ENSMUST00000003659	ENSMUSG00000031849
novel_circ_0001015	mmu-miR-140-3p	ENSMUST00000212488	ENSMUSG00000031849
novel_circ_0000835	mmu-miR-140-3p	ENSMUST00000003659	ENSMUSG00000031849
novel_circ_0000835	mmu-miR-140-3p	ENSMUST00000212488	ENSMUSG00000031849
novel_circ_0001194	mmu-miR-140-3p	ENSMUST00000003659	ENSMUSG00000031849
novel_circ_0001194	mmu-miR-140-3p	ENSMUST00000212488	ENSMUSG00000031849
novel_circ_0001207	mmu-miR-140-3p	ENSMUST00000003659	ENSMUSG00000031849
novel_circ_0001207	mmu-miR-140-3p	ENSMUST00000212488	ENSMUSG00000031849
novel_circ_0001208	mmu-miR-140-3p	ENSMUST00000003659	ENSMUSG00000031849
novel_circ_0001208	mmu-miR-140-3p	ENSMUST00000212488	ENSMUSG00000031849
novel_circ_0001433	mmu-miR-150-3p	ENSMUST00000063750	ENSMUSG00000017491
mmu_circ_0001145	mmu-miR-150-3p	ENSMUST00000063750	ENSMUSG00000017491
mmu_circ_0001033	mmu-miR-150-3p	ENSMUST00000063750	ENSMUSG00000017491
mmu_circ_0001376	mmu-miR-150-3p	ENSMUST00000063750	ENSMUSG00000017491
mmu_circ_0001333	mmu-miR-150-3p	ENSMUST00000063750	ENSMUSG00000017491
mmu_circ_0001210	mmu-miR-150-3p	ENSMUST00000063750	ENSMUSG00000017491
mmu_circ_0001500	mmu-miR-150-3p	ENSMUST00000063750	ENSMUSG00000017491
mmu_circ_0001842	mmu-miR-150-3p	ENSMUST00000063750	ENSMUSG00000017491
mmu_circ_0001743	mmu-miR-150-3p	ENSMUST00000063750	ENSMUSG00000017491
mmu_circ_0001827	mmu-miR-150-3p	ENSMUST00000063750	ENSMUSG00000017491
mmu_circ_0001664	mmu-miR-150-3p	ENSMUST00000063750	ENSMUSG00000017491

mmu_circ_0001676	mmu-miR-150-3p	ENSMUST00000063750	ENSMUSG00000017491
mmu_circ_0000153	mmu-miR-150-3p	ENSMUST00000063750	ENSMUSG00000017491
mmu_circ_0000499	mmu-miR-150-3p	ENSMUST00000063750	ENSMUSG00000017491
mmu_circ_0000500	mmu-miR-150-3p	ENSMUST00000063750	ENSMUSG00000017491
mmu_circ_0000628	mmu-miR-150-3p	ENSMUST00000063750	ENSMUSG00000017491
mmu_circ_0000530	mmu-miR-150-3p	ENSMUST00000063750	ENSMUSG00000017491
mmu_circ_0000552	mmu-miR-150-3p	ENSMUST00000063750	ENSMUSG00000017491
mmu_circ_0000820	mmu-miR-150-3p	ENSMUST00000063750	ENSMUSG00000017491
mmu_circ_0000829	mmu-miR-150-3p	ENSMUST00000063750	ENSMUSG00000017491
mmu_circ_0000711	mmu-miR-150-3p	ENSMUST00000063750	ENSMUSG00000017491
mmu_circ_0000714	mmu-miR-150-3p	ENSMUST00000063750	ENSMUSG00000017491
mmu_circ_0000950	mmu-miR-150-3p	ENSMUST00000063750	ENSMUSG00000017491
mmu_circ_0000919	mmu-miR-150-3p	ENSMUST00000063750	ENSMUSG00000017491
mmu_circ_0000921	mmu-miR-150-3p	ENSMUST00000063750	ENSMUSG00000017491
mmu_circ_0000905	mmu-miR-150-3p	ENSMUST00000063750	ENSMUSG00000017491
mmu_circ_0000906	mmu-miR-150-3p	ENSMUST00000063750	ENSMUSG00000017491
mmu_circ_0000907	mmu-miR-150-3p	ENSMUST00000063750	ENSMUSG00000017491
mmu_circ_0000908	mmu-miR-150-3p	ENSMUST00000063750	ENSMUSG00000017491
mmu_circ_0000912	mmu-miR-150-3p	ENSMUST00000063750	ENSMUSG00000017491
novel_circ_0001561	mmu-miR-150-3p	ENSMUST00000063750	ENSMUSG00000017491
novel_circ_0001624	mmu-miR-150-3p	ENSMUST00000063750	ENSMUSG00000017491
novel_circ_0001728	mmu-miR-150-3p	ENSMUST00000063750	ENSMUSG00000017491
novel_circ_0002214	mmu-miR-150-3p	ENSMUST00000063750	ENSMUSG00000017491
novel_circ_0001810	mmu-miR-150-3p	ENSMUST00000063750	ENSMUSG00000017491
novel_circ_0001994	mmu-miR-150-3p	ENSMUST00000063750	ENSMUSG00000017491
novel_circ_0002014	mmu-miR-150-3p	ENSMUST00000063750	ENSMUSG00000017491
novel_circ_0002456	mmu-miR-150-3p	ENSMUST00000063750	ENSMUSG00000017491
novel_circ_0002510	mmu-miR-150-3p	ENSMUST00000063750	ENSMUSG00000017491
novel_circ_0002511	mmu-miR-150-3p	ENSMUST00000063750	ENSMUSG00000017491
novel_circ_0002571	mmu-miR-150-3p	ENSMUST00000063750	ENSMUSG00000017491
novel_circ_0002282	mmu-miR-150-3p	ENSMUST00000063750	ENSMUSG00000017491
novel_circ_0002357	mmu-miR-150-3p	ENSMUST00000063750	ENSMUSG00000017491
novel_circ_0002836	mmu-miR-150-3p	ENSMUST00000063750	ENSMUSG00000017491
novel_circ_0002982	mmu-miR-150-3p	ENSMUST00000063750	ENSMUSG00000017491
novel_circ_0002682	mmu-miR-150-3p	ENSMUST00000063750	ENSMUSG00000017491
novel_circ_0002788	mmu-miR-150-3p	ENSMUST00000063750	ENSMUSG00000017491
novel_circ_0003185	mmu-miR-150-3p	ENSMUST00000063750	ENSMUSG00000017491
novel_circ_0003187	mmu-miR-150-3p	ENSMUST00000063750	ENSMUSG00000017491
novel_circ_0003312	mmu-miR-150-3p	ENSMUST00000063750	ENSMUSG00000017491
novel_circ_0003313	mmu-miR-150-3p	ENSMUST00000063750	ENSMUSG00000017491
novel_circ_0003313	mmu-miR-150-3p	ENSMUST00000063750	ENSMUSG00000017491
novel_circ_0000386	mmu-miR-150-3p	ENSMUST00000063750	ENSMUSG00000017491
novel_circ_0000069	mmu-miR-150-3p	ENSMUST00000063750	ENSMUSG00000017491
novel_circ_0000698	mmu-miR-150-3p	ENSMUST00000063750	ENSMUSG00000017491
novel_circ_0001048	mmu-miR-150-3p	ENSMUST00000063750	ENSMUSG00000017491
novel_circ_0000815	mmu-miR-150-3p	ENSMUST00000063750	ENSMUSG00000017491
novel_circ_0000816	mmu-miR-150-3p	ENSMUST00000063750	ENSMUSG00000017491
novel_circ_0000902	mmu-miR-150-3p	ENSMUST00000063750	ENSMUSG00000017491
novel_circ_0001189	mmu-miR-150-3p	ENSMUST00000063750	ENSMUSG00000017491
novel_circ_0001189	mmu-miR-150-3p	ENSMUST00000063750	ENSMUSG00000017491
novel_circ_0001198	mmu-miR-150-3p	ENSMUST00000063750	ENSMUSG00000017491
novel_circ_0001208	mmu-miR-150-3p	ENSMUST00000063750	ENSMUSG00000017491
novel_circ_0001106	mmu-miR-296-3p	ENSMUST00000000128	ENSMUSG00000000126
novel_circ_0001106	mmu-miR-296-3p	ENSMUST00000108783	ENSMUSG00000000126
novel_circ_0001141	mmu-miR-296-3p	ENSMUST00000000128	ENSMUSG00000000126
novel_circ_0001141	mmu-miR-296-3p	ENSMUST00000108783	ENSMUSG00000000126
novel_circ_0001493	mmu-miR-296-3p	ENSMUST00000000128	ENSMUSG00000000126
novel_circ_0001493	mmu-miR-296-3p	ENSMUST00000108783	ENSMUSG00000000126

novel_circ_0001340	mmu-miR-296-3p	ENSMUST00000000128	ENSMUSG00000000126
novel_circ_0001340	mmu-miR-296-3p	ENSMUST00000108783	ENSMUSG00000000126
mmu_circ_0000042	mmu-miR-296-3p	ENSMUST00000000128	ENSMUSG00000000126
mmu_circ_0000042	mmu-miR-296-3p	ENSMUST00000108783	ENSMUSG00000000126
mmu_circ_0001135	mmu-miR-296-3p	ENSMUST00000000128	ENSMUSG00000000126
mmu_circ_0001135	mmu-miR-296-3p	ENSMUST00000108783	ENSMUSG00000000126
mmu_circ_0001097	mmu-miR-296-3p	ENSMUST00000000128	ENSMUSG00000000126
mmu_circ_0001097	mmu-miR-296-3p	ENSMUST00000108783	ENSMUSG00000000126
mmu_circ_0001376	mmu-miR-296-3p	ENSMUST00000000128	ENSMUSG00000000126
mmu_circ_0001376	mmu-miR-296-3p	ENSMUST00000108783	ENSMUSG00000000126
mmu_circ_0001414	mmu-miR-296-3p	ENSMUST00000000128	ENSMUSG00000000126
mmu_circ_0001414	mmu-miR-296-3p	ENSMUST00000108783	ENSMUSG00000000126
mmu_circ_0001415	mmu-miR-296-3p	ENSMUST00000000128	ENSMUSG00000000126
mmu_circ_0001415	mmu-miR-296-3p	ENSMUST00000108783	ENSMUSG00000000126
mmu_circ_0001336	mmu-miR-296-3p	ENSMUST00000000128	ENSMUSG00000000126
mmu_circ_0001336	mmu-miR-296-3p	ENSMUST00000108783	ENSMUSG00000000126
mmu_circ_0001298	mmu-miR-296-3p	ENSMUST00000000128	ENSMUSG00000000126
mmu_circ_0001298	mmu-miR-296-3p	ENSMUST00000108783	ENSMUSG00000000126
mmu_circ_0001298	mmu-miR-296-3p	ENSMUST00000000128	ENSMUSG00000000126
mmu_circ_0001298	mmu-miR-296-3p	ENSMUST00000108783	ENSMUSG00000000126
mmu_circ_0001302	mmu-miR-296-3p	ENSMUST00000000128	ENSMUSG00000000126
mmu_circ_0001302	mmu-miR-296-3p	ENSMUST00000108783	ENSMUSG00000000126
mmu_circ_0001302	mmu-miR-296-3p	ENSMUST00000000128	ENSMUSG00000000126
mmu_circ_0001302	mmu-miR-296-3p	ENSMUST00000108783	ENSMUSG00000000126
mmu_circ_0001634	mmu-miR-296-3p	ENSMUST00000000128	ENSMUSG00000000126
mmu_circ_0001634	mmu-miR-296-3p	ENSMUST00000108783	ENSMUSG00000000126
mmu_circ_0001454	mmu-miR-296-3p	ENSMUST00000000128	ENSMUSG00000000126
mmu_circ_0001454	mmu-miR-296-3p	ENSMUST00000108783	ENSMUSG00000000126
mmu_circ_0001470	mmu-miR-296-3p	ENSMUST00000000128	ENSMUSG00000000126
mmu_circ_0001470	mmu-miR-296-3p	ENSMUST00000108783	ENSMUSG00000000126
mmu_circ_0001499	mmu-miR-296-3p	ENSMUST00000000128	ENSMUSG00000000126
mmu_circ_0001499	mmu-miR-296-3p	ENSMUST00000108783	ENSMUSG00000000126
mmu_circ_0001500	mmu-miR-296-3p	ENSMUST00000000128	ENSMUSG00000000126
mmu_circ_0001500	mmu-miR-296-3p	ENSMUST00000108783	ENSMUSG00000000126
mmu_circ_0001675	mmu-miR-296-3p	ENSMUST00000000128	ENSMUSG00000000126
mmu_circ_0001675	mmu-miR-296-3p	ENSMUST00000108783	ENSMUSG00000000126
mmu_circ_0001675	mmu-miR-296-3p	ENSMUST00000000128	ENSMUSG00000000126
mmu_circ_0001675	mmu-miR-296-3p	ENSMUST00000108783	ENSMUSG00000000126
mmu_circ_0000399	mmu-miR-296-3p	ENSMUST00000000128	ENSMUSG00000000126
mmu_circ_0000399	mmu-miR-296-3p	ENSMUST00000108783	ENSMUSG00000000126
mmu_circ_0000644	mmu-miR-296-3p	ENSMUST00000000128	ENSMUSG00000000126
mmu_circ_0000644	mmu-miR-296-3p	ENSMUST00000108783	ENSMUSG00000000126
mmu_circ_0000530	mmu-miR-296-3p	ENSMUST00000000128	ENSMUSG00000000126
mmu_circ_0000530	mmu-miR-296-3p	ENSMUST00000108783	ENSMUSG00000000126
mmu_circ_0000735	mmu-miR-296-3p	ENSMUST00000000128	ENSMUSG00000000126
mmu_circ_0000735	mmu-miR-296-3p	ENSMUST00000108783	ENSMUSG00000000126
mmu_circ_0000805	mmu-miR-296-3p	ENSMUST00000000128	ENSMUSG00000000126
mmu_circ_0000805	mmu-miR-296-3p	ENSMUST00000108783	ENSMUSG00000000126
mmu_circ_0000819	mmu-miR-296-3p	ENSMUST00000000128	ENSMUSG00000000126
mmu_circ_0000819	mmu-miR-296-3p	ENSMUST00000108783	ENSMUSG00000000126
mmu_circ_0000831	mmu-miR-296-3p	ENSMUST00000000128	ENSMUSG00000000126
mmu_circ_0000831	mmu-miR-296-3p	ENSMUST00000108783	ENSMUSG00000000126
mmu_circ_0000833	mmu-miR-296-3p	ENSMUST00000000128	ENSMUSG00000000126
mmu_circ_0000833	mmu-miR-296-3p	ENSMUST00000108783	ENSMUSG00000000126
mmu_circ_0000713	mmu-miR-296-3p	ENSMUST00000000128	ENSMUSG00000000126
mmu_circ_0000713	mmu-miR-296-3p	ENSMUST00000108783	ENSMUSG00000000126
mmu_circ_0000714	mmu-miR-296-3p	ENSMUST00000000128	ENSMUSG00000000126
mmu_circ_0000714	mmu-miR-296-3p	ENSMUST00000108783	ENSMUSG00000000126

mmu_circ_0000969	mmu-miR-296-3p	ENSMUST00000000128	ENSMUSG00000000126
mmu_circ_0000969	mmu-miR-296-3p	ENSMUST00000108783	ENSMUSG00000000126
mmu_circ_0000906	mmu-miR-296-3p	ENSMUST00000000128	ENSMUSG00000000126
mmu_circ_0000906	mmu-miR-296-3p	ENSMUST00000108783	ENSMUSG00000000126
novel_circ_0001859	mmu-miR-296-3p	ENSMUST00000000128	ENSMUSG00000000126
novel_circ_0001859	mmu-miR-296-3p	ENSMUST00000108783	ENSMUSG00000000126
novel_circ_0001861	mmu-miR-296-3p	ENSMUST00000000128	ENSMUSG00000000126
novel_circ_0001861	mmu-miR-296-3p	ENSMUST00000108783	ENSMUSG00000000126
novel_circ_0001902	mmu-miR-296-3p	ENSMUST00000000128	ENSMUSG00000000126
novel_circ_0001902	mmu-miR-296-3p	ENSMUST00000108783	ENSMUSG00000000126
novel_circ_0001903	mmu-miR-296-3p	ENSMUST00000000128	ENSMUSG00000000126
novel_circ_0001903	mmu-miR-296-3p	ENSMUST00000108783	ENSMUSG00000000126
novel_circ_0001914	mmu-miR-296-3p	ENSMUST00000000128	ENSMUSG00000000126
novel_circ_0001914	mmu-miR-296-3p	ENSMUST00000108783	ENSMUSG00000000126
novel_circ_0002506	mmu-miR-296-3p	ENSMUST00000000128	ENSMUSG00000000126
novel_circ_0002506	mmu-miR-296-3p	ENSMUST00000108783	ENSMUSG00000000126
novel_circ_0002839	mmu-miR-296-3p	ENSMUST00000000128	ENSMUSG00000000126
novel_circ_0002839	mmu-miR-296-3p	ENSMUST00000108783	ENSMUSG00000000126
novel_circ_0002732	mmu-miR-296-3p	ENSMUST00000000128	ENSMUSG00000000126
novel_circ_0002732	mmu-miR-296-3p	ENSMUST00000108783	ENSMUSG00000000126
novel_circ_0002788	mmu-miR-296-3p	ENSMUST00000000128	ENSMUSG00000000126
novel_circ_0002788	mmu-miR-296-3p	ENSMUST00000108783	ENSMUSG00000000126
novel_circ_0003213	mmu-miR-296-3p	ENSMUST00000000128	ENSMUSG00000000126
novel_circ_0003213	mmu-miR-296-3p	ENSMUST00000108783	ENSMUSG00000000126
novel_circ_0003370	mmu-miR-296-3p	ENSMUST00000000128	ENSMUSG00000000126
novel_circ_0003370	mmu-miR-296-3p	ENSMUST00000108783	ENSMUSG00000000126
novel_circ_0003468	mmu-miR-296-3p	ENSMUST00000000128	ENSMUSG00000000126
novel_circ_0003468	mmu-miR-296-3p	ENSMUST00000108783	ENSMUSG00000000126
novel_circ_0003475	mmu-miR-296-3p	ENSMUST00000000128	ENSMUSG00000000126
novel_circ_0003475	mmu-miR-296-3p	ENSMUST00000108783	ENSMUSG00000000126
novel_circ_0000323	mmu-miR-296-3p	ENSMUST00000000128	ENSMUSG00000000126
novel_circ_0000323	mmu-miR-296-3p	ENSMUST00000108783	ENSMUSG00000000126
novel_circ_0000343	mmu-miR-296-3p	ENSMUST00000000128	ENSMUSG00000000126
novel_circ_0000343	mmu-miR-296-3p	ENSMUST00000108783	ENSMUSG00000000126
novel_circ_0000021	mmu-miR-296-3p	ENSMUST00000000128	ENSMUSG00000000126
novel_circ_0000021	mmu-miR-296-3p	ENSMUST00000108783	ENSMUSG00000000126
novel_circ_0000141	mmu-miR-296-3p	ENSMUST00000000128	ENSMUSG00000000126
novel_circ_0000141	mmu-miR-296-3p	ENSMUST00000108783	ENSMUSG00000000126
novel_circ_0000692	mmu-miR-296-3p	ENSMUST00000000128	ENSMUSG00000000126
novel_circ_0000692	mmu-miR-296-3p	ENSMUST00000108783	ENSMUSG00000000126
novel_circ_0000538	mmu-miR-296-3p	ENSMUST00000000128	ENSMUSG00000000126
novel_circ_0000538	mmu-miR-296-3p	ENSMUST00000108783	ENSMUSG00000000126
novel_circ_0000589	mmu-miR-296-3p	ENSMUST00000000128	ENSMUSG00000000126
novel_circ_0000589	mmu-miR-296-3p	ENSMUST00000108783	ENSMUSG00000000126
novel_circ_0001002	mmu-miR-296-3p	ENSMUST00000000128	ENSMUSG00000000126
novel_circ_0001002	mmu-miR-296-3p	ENSMUST00000108783	ENSMUSG00000000126
novel_circ_0001003	mmu-miR-296-3p	ENSMUST00000000128	ENSMUSG00000000126
novel_circ_0001003	mmu-miR-296-3p	ENSMUST00000108783	ENSMUSG00000000126
novel_circ_0001177	mmu-miR-296-3p	ENSMUST00000000128	ENSMUSG00000000126
novel_circ_0001177	mmu-miR-296-3p	ENSMUST00000108783	ENSMUSG00000000126
novel_circ_0001179	mmu-miR-296-3p	ENSMUST00000000128	ENSMUSG00000000126
novel_circ_0001179	mmu-miR-296-3p	ENSMUST00000108783	ENSMUSG00000000126
novel_circ_0001222	mmu-miR-297a-5	ENSMUST00000110698	ENSMUSG00000055737
novel_circ_0001306	mmu-miR-297a-5	ENSMUST00000110698	ENSMUSG00000055737
novel_circ_0001145	mmu-miR-297a-5	ENSMUST00000110698	ENSMUSG00000055737
novel_circ_0001145	mmu-miR-297a-5	ENSMUST00000110698	ENSMUSG00000055737
novel_circ_0001346	mmu-miR-297a-5	ENSMUST00000110698	ENSMUSG00000055737
mmu_circ_0001162	mmu-miR-297a-5	ENSMUST00000110698	ENSMUSG00000055737

mmu_circ_0001123	mmu-miR-297a-5	ENSMUST00000110698	ENSMUSG00000055737
mmu_circ_0001136	mmu-miR-297a-5	ENSMUST00000110698	ENSMUSG00000055737
mmu_circ_0001376	mmu-miR-297a-5	ENSMUST00000110698	ENSMUSG00000055737
mmu_circ_0001333	mmu-miR-297a-5	ENSMUST00000110698	ENSMUSG00000055737
mmu_circ_0001335	mmu-miR-297a-5	ENSMUST00000110698	ENSMUSG00000055737
mmu_circ_0001336	mmu-miR-297a-5	ENSMUST00000110698	ENSMUSG00000055737
mmu_circ_0001507	mmu-miR-297a-5	ENSMUST00000110698	ENSMUSG00000055737
mmu_circ_0001454	mmu-miR-297a-5	ENSMUST00000110698	ENSMUSG00000055737
mmu_circ_0001500	mmu-miR-297a-5	ENSMUST00000110698	ENSMUSG00000055737
mmu_circ_0001755	mmu-miR-297a-5	ENSMUST00000110698	ENSMUSG00000055737
mmu_circ_0001755	mmu-miR-297a-5	ENSMUST00000110698	ENSMUSG00000055737
mmu_circ_0001827	mmu-miR-297a-5	ENSMUST00000110698	ENSMUSG00000055737
mmu_circ_0001886	mmu-miR-297a-5	ENSMUST00000110698	ENSMUSG00000055737
mmu_circ_0000271	mmu-miR-297a-5	ENSMUST00000110698	ENSMUSG00000055737
mmu_circ_0000230	mmu-miR-297a-5	ENSMUST00000110698	ENSMUSG00000055737
mmu_circ_0000499	mmu-miR-297a-5	ENSMUST00000110698	ENSMUSG00000055737
mmu_circ_0000500	mmu-miR-297a-5	ENSMUST00000110698	ENSMUSG00000055737
mmu_circ_0000798	mmu-miR-297a-5	ENSMUST00000110698	ENSMUSG00000055737
mmu_circ_0000812	mmu-miR-297a-5	ENSMUST00000110698	ENSMUSG00000055737
mmu_circ_0000829	mmu-miR-297a-5	ENSMUST00000110698	ENSMUSG00000055737
mmu_circ_0000681	mmu-miR-297a-5	ENSMUST00000110698	ENSMUSG00000055737
mmu_circ_0000712	mmu-miR-297a-5	ENSMUST00000110698	ENSMUSG00000055737
mmu_circ_0000713	mmu-miR-297a-5	ENSMUST00000110698	ENSMUSG00000055737
mmu_circ_0000714	mmu-miR-297a-5	ENSMUST00000110698	ENSMUSG00000055737
mmu_circ_0000950	mmu-miR-297a-5	ENSMUST00000110698	ENSMUSG00000055737
mmu_circ_0000969	mmu-miR-297a-5	ENSMUST00000110698	ENSMUSG00000055737
mmu_circ_0000884	mmu-miR-297a-5	ENSMUST00000110698	ENSMUSG00000055737
mmu_circ_0000886	mmu-miR-297a-5	ENSMUST00000110698	ENSMUSG00000055737
mmu_circ_0000887	mmu-miR-297a-5	ENSMUST00000110698	ENSMUSG00000055737
mmu_circ_0000905	mmu-miR-297a-5	ENSMUST00000110698	ENSMUSG00000055737
mmu_circ_0000906	mmu-miR-297a-5	ENSMUST00000110698	ENSMUSG00000055737
mmu_circ_0000907	mmu-miR-297a-5	ENSMUST00000110698	ENSMUSG00000055737
mmu_circ_0000908	mmu-miR-297a-5	ENSMUST00000110698	ENSMUSG00000055737
novel_circ_0001680	mmu-miR-297a-5	ENSMUST00000110698	ENSMUSG00000055737
novel_circ_0001721	mmu-miR-297a-5	ENSMUST00000110698	ENSMUSG00000055737
novel_circ_0002082	mmu-miR-297a-5	ENSMUST00000110698	ENSMUSG00000055737
novel_circ_0001859	mmu-miR-297a-5	ENSMUST00000110698	ENSMUSG00000055737
novel_circ_0001861	mmu-miR-297a-5	ENSMUST00000110698	ENSMUSG00000055737
novel_circ_0001933	mmu-miR-297a-5	ENSMUST00000110698	ENSMUSG00000055737
novel_circ_0001934	mmu-miR-297a-5	ENSMUST00000110698	ENSMUSG00000055737
novel_circ_0002507	mmu-miR-297a-5	ENSMUST00000110698	ENSMUSG00000055737
novel_circ_0002508	mmu-miR-297a-5	ENSMUST00000110698	ENSMUSG00000055737
novel_circ_0002581	mmu-miR-297a-5	ENSMUST00000110698	ENSMUSG00000055737
novel_circ_0002374	mmu-miR-297a-5	ENSMUST00000110698	ENSMUSG00000055737
novel_circ_0002978	mmu-miR-297a-5	ENSMUST00000110698	ENSMUSG00000055737
novel_circ_0002985	mmu-miR-297a-5	ENSMUST00000110698	ENSMUSG00000055737
novel_circ_0002990	mmu-miR-297a-5	ENSMUST00000110698	ENSMUSG00000055737
novel_circ_0002642	mmu-miR-297a-5	ENSMUST00000110698	ENSMUSG00000055737
novel_circ_0002695	mmu-miR-297a-5	ENSMUST00000110698	ENSMUSG00000055737
novel_circ_0002782	mmu-miR-297a-5	ENSMUST00000110698	ENSMUSG00000055737
novel_circ_0003016	mmu-miR-297a-5	ENSMUST00000110698	ENSMUSG00000055737
novel_circ_0000188	mmu-miR-297a-5	ENSMUST00000110698	ENSMUSG00000055737
novel_circ_0000268	mmu-miR-297a-5	ENSMUST00000110698	ENSMUSG00000055737
novel_circ_0000985	mmu-miR-297a-5	ENSMUST00000110698	ENSMUSG00000055737
novel_circ_0000807	mmu-miR-297a-5	ENSMUST00000110698	ENSMUSG00000055737
novel_circ_0001191	mmu-miR-297a-5	ENSMUST00000110698	ENSMUSG00000055737
novel_circ_0001191	mmu-miR-297a-5	ENSMUST00000110698	ENSMUSG00000055737
novel_circ_0001062	mmu-let-7d-5p	ENSMUST00000073554	ENSMUSG00000062329

novel_circ_0001450	mmu-let-7d-5p	ENSMUST00000073554	ENSMUSG00000062329
novel_circ_0001501	mmu-let-7d-5p	ENSMUST00000073554	ENSMUSG00000062329
novel_circ_0001501	mmu-let-7d-5p	ENSMUST00000073554	ENSMUSG00000062329
mmu_circ_0000040	mmu-let-7d-5p	ENSMUST00000073554	ENSMUSG00000062329
mmu_circ_0000041	mmu-let-7d-5p	ENSMUST00000073554	ENSMUSG00000062329
mmu_circ_0000042	mmu-let-7d-5p	ENSMUST00000073554	ENSMUSG00000062329
mmu_circ_0001376	mmu-let-7d-5p	ENSMUST00000073554	ENSMUSG00000062329
mmu_circ_0001263	mmu-let-7d-5p	ENSMUST00000073554	ENSMUSG00000062329
mmu_circ_0001264	mmu-let-7d-5p	ENSMUST00000073554	ENSMUSG00000062329
mmu_circ_0001469	mmu-let-7d-5p	ENSMUST00000073554	ENSMUSG00000062329
mmu_circ_0001500	mmu-let-7d-5p	ENSMUST00000073554	ENSMUSG00000062329
mmu_circ_0001804	mmu-let-7d-5p	ENSMUST00000073554	ENSMUSG00000062329
mmu_circ_0001827	mmu-let-7d-5p	ENSMUST00000073554	ENSMUSG00000062329
mmu_circ_0001675	mmu-let-7d-5p	ENSMUST00000073554	ENSMUSG00000062329
mmu_circ_0000243	mmu-let-7d-5p	ENSMUST00000073554	ENSMUSG00000062329
mmu_circ_0000277	mmu-let-7d-5p	ENSMUST00000073554	ENSMUSG00000062329
mmu_circ_0000141	mmu-let-7d-5p	ENSMUST00000073554	ENSMUSG00000062329
mmu_circ_0000499	mmu-let-7d-5p	ENSMUST00000073554	ENSMUSG00000062329
mmu_circ_0000500	mmu-let-7d-5p	ENSMUST00000073554	ENSMUSG00000062329
mmu_circ_0000349	mmu-let-7d-5p	ENSMUST00000073554	ENSMUSG00000062329
mmu_circ_0000376	mmu-let-7d-5p	ENSMUST00000073554	ENSMUSG00000062329
mmu_circ_0000628	mmu-let-7d-5p	ENSMUST00000073554	ENSMUSG00000062329
mmu_circ_0000530	mmu-let-7d-5p	ENSMUST00000073554	ENSMUSG00000062329
mmu_circ_0000804	mmu-let-7d-5p	ENSMUST00000073554	ENSMUSG00000062329
mmu_circ_0000828	mmu-let-7d-5p	ENSMUST00000073554	ENSMUSG00000062329
mmu_circ_0000829	mmu-let-7d-5p	ENSMUST00000073554	ENSMUSG00000062329
mmu_circ_0000712	mmu-let-7d-5p	ENSMUST00000073554	ENSMUSG00000062329
mmu_circ_0000964	mmu-let-7d-5p	ENSMUST00000073554	ENSMUSG00000062329
mmu_circ_0000840	mmu-let-7d-5p	ENSMUST00000073554	ENSMUSG00000062329
mmu_circ_0000884	mmu-let-7d-5p	ENSMUST00000073554	ENSMUSG00000062329
mmu_circ_0000886	mmu-let-7d-5p	ENSMUST00000073554	ENSMUSG00000062329
mmu_circ_0000905	mmu-let-7d-5p	ENSMUST00000073554	ENSMUSG00000062329
mmu_circ_0000912	mmu-let-7d-5p	ENSMUST00000073554	ENSMUSG00000062329
novel_circ_0001564	mmu-let-7d-5p	ENSMUST00000073554	ENSMUSG00000062329
novel_circ_0001579	mmu-let-7d-5p	ENSMUST00000073554	ENSMUSG00000062329
novel_circ_0001579	mmu-let-7d-5p	ENSMUST00000073554	ENSMUSG00000062329
novel_circ_0001608	mmu-let-7d-5p	ENSMUST00000073554	ENSMUSG00000062329
novel_circ_0001702	mmu-let-7d-5p	ENSMUST00000073554	ENSMUSG00000062329
novel_circ_0001702	mmu-let-7d-5p	ENSMUST00000073554	ENSMUSG00000062329
novel_circ_0001743	mmu-let-7d-5p	ENSMUST00000073554	ENSMUSG00000062329
novel_circ_0002071	mmu-let-7d-5p	ENSMUST00000073554	ENSMUSG00000062329
novel_circ_0002086	mmu-let-7d-5p	ENSMUST00000073554	ENSMUSG00000062329
novel_circ_0002145	mmu-let-7d-5p	ENSMUST00000073554	ENSMUSG00000062329
novel_circ_0001759	mmu-let-7d-5p	ENSMUST00000073554	ENSMUSG00000062329
novel_circ_0001864	mmu-let-7d-5p	ENSMUST00000073554	ENSMUSG00000062329
novel_circ_0001870	mmu-let-7d-5p	ENSMUST00000073554	ENSMUSG00000062329
novel_circ_0001902	mmu-let-7d-5p	ENSMUST00000073554	ENSMUSG00000062329
novel_circ_0001903	mmu-let-7d-5p	ENSMUST00000073554	ENSMUSG00000062329
novel_circ_0002039	mmu-let-7d-5p	ENSMUST00000073554	ENSMUSG00000062329
novel_circ_0002417	mmu-let-7d-5p	ENSMUST00000073554	ENSMUSG00000062329
novel_circ_0002231	mmu-let-7d-5p	ENSMUST00000073554	ENSMUSG00000062329
novel_circ_0002343	mmu-let-7d-5p	ENSMUST00000073554	ENSMUSG00000062329
novel_circ_0002866	mmu-let-7d-5p	ENSMUST00000073554	ENSMUSG00000062329
novel_circ_0002913	mmu-let-7d-5p	ENSMUST00000073554	ENSMUSG00000062329
novel_circ_0002926	mmu-let-7d-5p	ENSMUST00000073554	ENSMUSG00000062329
novel_circ_0002933	mmu-let-7d-5p	ENSMUST00000073554	ENSMUSG00000062329
novel_circ_0002732	mmu-let-7d-5p	ENSMUST00000073554	ENSMUSG00000062329
novel_circ_0002782	mmu-let-7d-5p	ENSMUST00000073554	ENSMUSG00000062329

novel_circ_0002788	mmu-let-7d-5p	ENSMUST00000073554	ENSMUSG00000062329
novel_circ_0003190	mmu-let-7d-5p	ENSMUST00000073554	ENSMUSG00000062329
novel_circ_0003213	mmu-let-7d-5p	ENSMUST00000073554	ENSMUSG00000062329
novel_circ_0003304	mmu-let-7d-5p	ENSMUST00000073554	ENSMUSG00000062329
novel_circ_0003096	mmu-let-7d-5p	ENSMUST00000073554	ENSMUSG00000062329
novel_circ_0003507	mmu-let-7d-5p	ENSMUST00000073554	ENSMUSG00000062329
novel_circ_0003457	mmu-let-7d-5p	ENSMUST00000073554	ENSMUSG00000062329
novel_circ_0000200	mmu-let-7d-5p	ENSMUST00000073554	ENSMUSG00000062329
novel_circ_0000201	mmu-let-7d-5p	ENSMUST00000073554	ENSMUSG00000062329
novel_circ_0000204	mmu-let-7d-5p	ENSMUST00000073554	ENSMUSG00000062329
novel_circ_0000204	mmu-let-7d-5p	ENSMUST00000073554	ENSMUSG00000062329
novel_circ_0000220	mmu-let-7d-5p	ENSMUST00000073554	ENSMUSG00000062329
novel_circ_0000222	mmu-let-7d-5p	ENSMUST00000073554	ENSMUSG00000062329
novel_circ_0000016	mmu-let-7d-5p	ENSMUST00000073554	ENSMUSG00000062329
novel_circ_0000022	mmu-let-7d-5p	ENSMUST00000073554	ENSMUSG00000062329
novel_circ_0000093	mmu-let-7d-5p	ENSMUST00000073554	ENSMUSG00000062329
novel_circ_0000128	mmu-let-7d-5p	ENSMUST00000073554	ENSMUSG00000062329
novel_circ_0000154	mmu-let-7d-5p	ENSMUST00000073554	ENSMUSG00000062329
novel_circ_0000484	mmu-let-7d-5p	ENSMUST00000073554	ENSMUSG00000062329
novel_circ_0000521	mmu-let-7d-5p	ENSMUST00000073554	ENSMUSG00000062329
novel_circ_0000525	mmu-let-7d-5p	ENSMUST00000073554	ENSMUSG00000062329
novel_circ_0000534	mmu-let-7d-5p	ENSMUST00000073554	ENSMUSG00000062329
novel_circ_0000589	mmu-let-7d-5p	ENSMUST00000073554	ENSMUSG00000062329
novel_circ_0000605	mmu-let-7d-5p	ENSMUST00000073554	ENSMUSG00000062329
novel_circ_0000606	mmu-let-7d-5p	ENSMUST00000073554	ENSMUSG00000062329
novel_circ_0000987	mmu-let-7d-5p	ENSMUST00000073554	ENSMUSG00000062329
novel_circ_0001163	mmu-let-7d-5p	ENSMUST00000073554	ENSMUSG00000062329
novel_circ_0001062	mmu-let-7e-5p	ENSMUST00000073554	ENSMUSG00000062329
novel_circ_0001450	mmu-let-7e-5p	ENSMUST00000073554	ENSMUSG00000062329
novel_circ_0001501	mmu-let-7e-5p	ENSMUST00000073554	ENSMUSG00000062329
novel_circ_0001501	mmu-let-7e-5p	ENSMUST00000073554	ENSMUSG00000062329
mmu_circ_0000040	mmu-let-7e-5p	ENSMUST00000073554	ENSMUSG00000062329
mmu_circ_0000041	mmu-let-7e-5p	ENSMUST00000073554	ENSMUSG00000062329
mmu_circ_0000042	mmu-let-7e-5p	ENSMUST00000073554	ENSMUSG00000062329
mmu_circ_0001376	mmu-let-7e-5p	ENSMUST00000073554	ENSMUSG00000062329
mmu_circ_0001263	mmu-let-7e-5p	ENSMUST00000073554	ENSMUSG00000062329
mmu_circ_0001264	mmu-let-7e-5p	ENSMUST00000073554	ENSMUSG00000062329
mmu_circ_0001469	mmu-let-7e-5p	ENSMUST00000073554	ENSMUSG00000062329
mmu_circ_0001500	mmu-let-7e-5p	ENSMUST00000073554	ENSMUSG00000062329
mmu_circ_0001804	mmu-let-7e-5p	ENSMUST00000073554	ENSMUSG00000062329
mmu_circ_0001827	mmu-let-7e-5p	ENSMUST00000073554	ENSMUSG00000062329
mmu_circ_0001675	mmu-let-7e-5p	ENSMUST00000073554	ENSMUSG00000062329
mmu_circ_0000243	mmu-let-7e-5p	ENSMUST00000073554	ENSMUSG00000062329
mmu_circ_0000277	mmu-let-7e-5p	ENSMUST00000073554	ENSMUSG00000062329
mmu_circ_0000141	mmu-let-7e-5p	ENSMUST00000073554	ENSMUSG00000062329
mmu_circ_0000499	mmu-let-7e-5p	ENSMUST00000073554	ENSMUSG00000062329
mmu_circ_0000500	mmu-let-7e-5p	ENSMUST00000073554	ENSMUSG00000062329
mmu_circ_0000349	mmu-let-7e-5p	ENSMUST00000073554	ENSMUSG00000062329
mmu_circ_0000376	mmu-let-7e-5p	ENSMUST00000073554	ENSMUSG00000062329
mmu_circ_0000628	mmu-let-7e-5p	ENSMUST00000073554	ENSMUSG00000062329
mmu_circ_0000530	mmu-let-7e-5p	ENSMUST00000073554	ENSMUSG00000062329
mmu_circ_0000804	mmu-let-7e-5p	ENSMUST00000073554	ENSMUSG00000062329
mmu_circ_0000828	mmu-let-7e-5p	ENSMUST00000073554	ENSMUSG00000062329
mmu_circ_0000829	mmu-let-7e-5p	ENSMUST00000073554	ENSMUSG00000062329
mmu_circ_0000712	mmu-let-7e-5p	ENSMUST00000073554	ENSMUSG00000062329
mmu_circ_0000964	mmu-let-7e-5p	ENSMUST00000073554	ENSMUSG00000062329
mmu_circ_0000840	mmu-let-7e-5p	ENSMUST00000073554	ENSMUSG00000062329
mmu_circ_0000884	mmu-let-7e-5p	ENSMUST00000073554	ENSMUSG00000062329

mmu_circ_0000886	mmu-let-7e-5p	ENSMUST00000073554	ENSMUSG00000062329
mmu_circ_0000905	mmu-let-7e-5p	ENSMUST00000073554	ENSMUSG00000062329
mmu_circ_0000912	mmu-let-7e-5p	ENSMUST00000073554	ENSMUSG00000062329
novel_circ_0001564	mmu-let-7e-5p	ENSMUST00000073554	ENSMUSG00000062329
novel_circ_0001579	mmu-let-7e-5p	ENSMUST00000073554	ENSMUSG00000062329
novel_circ_0001579	mmu-let-7e-5p	ENSMUST00000073554	ENSMUSG00000062329
novel_circ_0001608	mmu-let-7e-5p	ENSMUST00000073554	ENSMUSG00000062329
novel_circ_0001702	mmu-let-7e-5p	ENSMUST00000073554	ENSMUSG00000062329
novel_circ_0001702	mmu-let-7e-5p	ENSMUST00000073554	ENSMUSG00000062329
novel_circ_0001743	mmu-let-7e-5p	ENSMUST00000073554	ENSMUSG00000062329
novel_circ_0002071	mmu-let-7e-5p	ENSMUST00000073554	ENSMUSG00000062329
novel_circ_0002086	mmu-let-7e-5p	ENSMUST00000073554	ENSMUSG00000062329
novel_circ_0002145	mmu-let-7e-5p	ENSMUST00000073554	ENSMUSG00000062329
novel_circ_0001759	mmu-let-7e-5p	ENSMUST00000073554	ENSMUSG00000062329
novel_circ_0001864	mmu-let-7e-5p	ENSMUST00000073554	ENSMUSG00000062329
novel_circ_0001870	mmu-let-7e-5p	ENSMUST00000073554	ENSMUSG00000062329
novel_circ_0001902	mmu-let-7e-5p	ENSMUST00000073554	ENSMUSG00000062329
novel_circ_0001903	mmu-let-7e-5p	ENSMUST00000073554	ENSMUSG00000062329
novel_circ_0002039	mmu-let-7e-5p	ENSMUST00000073554	ENSMUSG00000062329
novel_circ_0002417	mmu-let-7e-5p	ENSMUST00000073554	ENSMUSG00000062329
novel_circ_0002231	mmu-let-7e-5p	ENSMUST00000073554	ENSMUSG00000062329
novel_circ_0002343	mmu-let-7e-5p	ENSMUST00000073554	ENSMUSG00000062329
novel_circ_0002866	mmu-let-7e-5p	ENSMUST00000073554	ENSMUSG00000062329
novel_circ_0002913	mmu-let-7e-5p	ENSMUST00000073554	ENSMUSG00000062329
novel_circ_0002926	mmu-let-7e-5p	ENSMUST00000073554	ENSMUSG00000062329
novel_circ_0002933	mmu-let-7e-5p	ENSMUST00000073554	ENSMUSG00000062329
novel_circ_0002732	mmu-let-7e-5p	ENSMUST00000073554	ENSMUSG00000062329
novel_circ_0002782	mmu-let-7e-5p	ENSMUST00000073554	ENSMUSG00000062329
novel_circ_0002788	mmu-let-7e-5p	ENSMUST00000073554	ENSMUSG00000062329
novel_circ_0003190	mmu-let-7e-5p	ENSMUST00000073554	ENSMUSG00000062329
novel_circ_0003213	mmu-let-7e-5p	ENSMUST00000073554	ENSMUSG00000062329
novel_circ_0003304	mmu-let-7e-5p	ENSMUST00000073554	ENSMUSG00000062329
novel_circ_0003096	mmu-let-7e-5p	ENSMUST00000073554	ENSMUSG00000062329
novel_circ_0003507	mmu-let-7e-5p	ENSMUST00000073554	ENSMUSG00000062329
novel_circ_0003457	mmu-let-7e-5p	ENSMUST00000073554	ENSMUSG00000062329
novel_circ_0000200	mmu-let-7e-5p	ENSMUST00000073554	ENSMUSG00000062329
novel_circ_0000201	mmu-let-7e-5p	ENSMUST00000073554	ENSMUSG00000062329
novel_circ_0000204	mmu-let-7e-5p	ENSMUST00000073554	ENSMUSG00000062329
novel_circ_0000204	mmu-let-7e-5p	ENSMUST00000073554	ENSMUSG00000062329
novel_circ_0000220	mmu-let-7e-5p	ENSMUST00000073554	ENSMUSG00000062329
novel_circ_0000222	mmu-let-7e-5p	ENSMUST00000073554	ENSMUSG00000062329
novel_circ_0000016	mmu-let-7e-5p	ENSMUST00000073554	ENSMUSG00000062329
novel_circ_0000022	mmu-let-7e-5p	ENSMUST00000073554	ENSMUSG00000062329
novel_circ_0000093	mmu-let-7e-5p	ENSMUST00000073554	ENSMUSG00000062329
novel_circ_0000128	mmu-let-7e-5p	ENSMUST00000073554	ENSMUSG00000062329
novel_circ_0000154	mmu-let-7e-5p	ENSMUST00000073554	ENSMUSG00000062329
novel_circ_0000484	mmu-let-7e-5p	ENSMUST00000073554	ENSMUSG00000062329
novel_circ_0000521	mmu-let-7e-5p	ENSMUST00000073554	ENSMUSG00000062329
novel_circ_0000525	mmu-let-7e-5p	ENSMUST00000073554	ENSMUSG00000062329
novel_circ_0000534	mmu-let-7e-5p	ENSMUST00000073554	ENSMUSG00000062329
novel_circ_0000589	mmu-let-7e-5p	ENSMUST00000073554	ENSMUSG00000062329
novel_circ_0000605	mmu-let-7e-5p	ENSMUST00000073554	ENSMUSG00000062329
novel_circ_0000606	mmu-let-7e-5p	ENSMUST00000073554	ENSMUSG00000062329
novel_circ_0000987	mmu-let-7e-5p	ENSMUST00000073554	ENSMUSG00000062329
novel_circ_0001163	mmu-let-7e-5p	ENSMUST00000073554	ENSMUSG00000062329
novel_circ_0001455	mmu-miR-23a-5p	ENSMUST00000000128	ENSMUSG00000000126
novel_circ_0001455	mmu-miR-23a-5p	ENSMUST00000108783	ENSMUSG00000000126
novel_circ_0001468	mmu-miR-23a-5p	ENSMUST00000000128	ENSMUSG00000000126

novel_circ_0001468	mmu-miR-23a-5p	ENSMUST00000108783	ENSMUSG00000000126
novel_circ_0001480	mmu-miR-23a-5p	ENSMUST00000000128	ENSMUSG00000000126
novel_circ_0001480	mmu-miR-23a-5p	ENSMUST00000108783	ENSMUSG00000000126
novel_circ_0001315	mmu-miR-23a-5p	ENSMUST00000000128	ENSMUSG00000000126
novel_circ_0001315	mmu-miR-23a-5p	ENSMUST00000108783	ENSMUSG00000000126
novel_circ_0001315	mmu-miR-23a-5p	ENSMUST00000000128	ENSMUSG00000000126
novel_circ_0001315	mmu-miR-23a-5p	ENSMUST00000108783	ENSMUSG00000000126
novel_circ_0001375	mmu-miR-23a-5p	ENSMUST00000000128	ENSMUSG00000000126
novel_circ_0001375	mmu-miR-23a-5p	ENSMUST00000108783	ENSMUSG00000000126
mmu_circ_0001123	mmu-miR-23a-5p	ENSMUST00000000128	ENSMUSG00000000126
mmu_circ_0001123	mmu-miR-23a-5p	ENSMUST00000108783	ENSMUSG00000000126
mmu_circ_0001311	mmu-miR-23a-5p	ENSMUST00000000128	ENSMUSG00000000126
mmu_circ_0001311	mmu-miR-23a-5p	ENSMUST00000108783	ENSMUSG00000000126
mmu_circ_0001634	mmu-miR-23a-5p	ENSMUST00000000128	ENSMUSG00000000126
mmu_circ_0001634	mmu-miR-23a-5p	ENSMUST00000108783	ENSMUSG00000000126
mmu_circ_0001499	mmu-miR-23a-5p	ENSMUST00000000128	ENSMUSG00000000126
mmu_circ_0001499	mmu-miR-23a-5p	ENSMUST00000108783	ENSMUSG00000000126
mmu_circ_0001500	mmu-miR-23a-5p	ENSMUST00000000128	ENSMUSG00000000126
mmu_circ_0001500	mmu-miR-23a-5p	ENSMUST00000108783	ENSMUSG00000000126
mmu_circ_0001688	mmu-miR-23a-5p	ENSMUST00000000128	ENSMUSG00000000126
mmu_circ_0001688	mmu-miR-23a-5p	ENSMUST00000108783	ENSMUSG00000000126
mmu_circ_0000243	mmu-miR-23a-5p	ENSMUST00000000128	ENSMUSG00000000126
mmu_circ_0000243	mmu-miR-23a-5p	ENSMUST00000108783	ENSMUSG00000000126
mmu_circ_0000294	mmu-miR-23a-5p	ENSMUST00000000128	ENSMUSG00000000126
mmu_circ_0000294	mmu-miR-23a-5p	ENSMUST00000108783	ENSMUSG00000000126
mmu_circ_0000144	mmu-miR-23a-5p	ENSMUST00000000128	ENSMUSG00000000126
mmu_circ_0000144	mmu-miR-23a-5p	ENSMUST00000108783	ENSMUSG00000000126
mmu_circ_0000145	mmu-miR-23a-5p	ENSMUST00000000128	ENSMUSG00000000126
mmu_circ_0000145	mmu-miR-23a-5p	ENSMUST00000108783	ENSMUSG00000000126
mmu_circ_0000153	mmu-miR-23a-5p	ENSMUST00000000128	ENSMUSG00000000126
mmu_circ_0000153	mmu-miR-23a-5p	ENSMUST00000108783	ENSMUSG00000000126
mmu_circ_0000154	mmu-miR-23a-5p	ENSMUST00000000128	ENSMUSG00000000126
mmu_circ_0000154	mmu-miR-23a-5p	ENSMUST00000108783	ENSMUSG00000000126
mmu_circ_0000499	mmu-miR-23a-5p	ENSMUST00000000128	ENSMUSG00000000126
mmu_circ_0000499	mmu-miR-23a-5p	ENSMUST00000108783	ENSMUSG00000000126
mmu_circ_0000500	mmu-miR-23a-5p	ENSMUST00000000128	ENSMUSG00000000126
mmu_circ_0000500	mmu-miR-23a-5p	ENSMUST00000108783	ENSMUSG00000000126
mmu_circ_0000371	mmu-miR-23a-5p	ENSMUST00000000128	ENSMUSG00000000126
mmu_circ_0000371	mmu-miR-23a-5p	ENSMUST00000108783	ENSMUSG00000000126
mmu_circ_0000399	mmu-miR-23a-5p	ENSMUST00000000128	ENSMUSG00000000126
mmu_circ_0000399	mmu-miR-23a-5p	ENSMUST00000108783	ENSMUSG00000000126
mmu_circ_0000628	mmu-miR-23a-5p	ENSMUST00000000128	ENSMUSG00000000126
mmu_circ_0000628	mmu-miR-23a-5p	ENSMUST00000108783	ENSMUSG00000000126
mmu_circ_0000530	mmu-miR-23a-5p	ENSMUST00000000128	ENSMUSG00000000126
mmu_circ_0000530	mmu-miR-23a-5p	ENSMUST00000108783	ENSMUSG00000000126
mmu_circ_0000829	mmu-miR-23a-5p	ENSMUST00000000128	ENSMUSG00000000126
mmu_circ_0000829	mmu-miR-23a-5p	ENSMUST00000108783	ENSMUSG00000000126
mmu_circ_0000693	mmu-miR-23a-5p	ENSMUST00000000128	ENSMUSG00000000126
mmu_circ_0000693	mmu-miR-23a-5p	ENSMUST00000108783	ENSMUSG00000000126
mmu_circ_0000711	mmu-miR-23a-5p	ENSMUST00000000128	ENSMUSG00000000126
mmu_circ_0000711	mmu-miR-23a-5p	ENSMUST00000108783	ENSMUSG00000000126
mmu_circ_0000712	mmu-miR-23a-5p	ENSMUST00000000128	ENSMUSG00000000126
mmu_circ_0000712	mmu-miR-23a-5p	ENSMUST00000108783	ENSMUSG00000000126
mmu_circ_0000713	mmu-miR-23a-5p	ENSMUST00000000128	ENSMUSG00000000126
mmu_circ_0000713	mmu-miR-23a-5p	ENSMUST00000108783	ENSMUSG00000000126
mmu_circ_0000714	mmu-miR-23a-5p	ENSMUST00000000128	ENSMUSG00000000126
mmu_circ_0000714	mmu-miR-23a-5p	ENSMUST00000108783	ENSMUSG00000000126
mmu_circ_0000884	mmu-miR-23a-5p	ENSMUST00000000128	ENSMUSG00000000126

mmu_circ_0000884	mmu-miR-23a-5p	ENSMUST00000108783	ENSMUSG00000000126
mmu_circ_0000886	mmu-miR-23a-5p	ENSMUST00000000128	ENSMUSG00000000126
mmu_circ_0000886	mmu-miR-23a-5p	ENSMUST00000108783	ENSMUSG00000000126
mmu_circ_0000887	mmu-miR-23a-5p	ENSMUST00000000128	ENSMUSG00000000126
mmu_circ_0000887	mmu-miR-23a-5p	ENSMUST00000108783	ENSMUSG00000000126
mmu_circ_0000897	mmu-miR-23a-5p	ENSMUST00000000128	ENSMUSG00000000126
mmu_circ_0000897	mmu-miR-23a-5p	ENSMUST00000108783	ENSMUSG00000000126
novel_circ_0001554	mmu-miR-23a-5p	ENSMUST00000000128	ENSMUSG00000000126
novel_circ_0001554	mmu-miR-23a-5p	ENSMUST00000108783	ENSMUSG00000000126
novel_circ_0001579	mmu-miR-23a-5p	ENSMUST00000000128	ENSMUSG00000000126
novel_circ_0001579	mmu-miR-23a-5p	ENSMUST00000108783	ENSMUSG00000000126
novel_circ_0001604	mmu-miR-23a-5p	ENSMUST00000000128	ENSMUSG00000000126
novel_circ_0001604	mmu-miR-23a-5p	ENSMUST00000108783	ENSMUSG00000000126
novel_circ_0001678	mmu-miR-23a-5p	ENSMUST00000000128	ENSMUSG00000000126
novel_circ_0001678	mmu-miR-23a-5p	ENSMUST00000108783	ENSMUSG00000000126
novel_circ_0001680	mmu-miR-23a-5p	ENSMUST00000000128	ENSMUSG00000000126
novel_circ_0001680	mmu-miR-23a-5p	ENSMUST00000108783	ENSMUSG00000000126
novel_circ_0001710	mmu-miR-23a-5p	ENSMUST00000000128	ENSMUSG00000000126
novel_circ_0001710	mmu-miR-23a-5p	ENSMUST00000108783	ENSMUSG00000000126
novel_circ_0002124	mmu-miR-23a-5p	ENSMUST00000000128	ENSMUSG00000000126
novel_circ_0002124	mmu-miR-23a-5p	ENSMUST00000108783	ENSMUSG00000000126
novel_circ_0001758	mmu-miR-23a-5p	ENSMUST00000000128	ENSMUSG00000000126
novel_circ_0001758	mmu-miR-23a-5p	ENSMUST00000108783	ENSMUSG00000000126
novel_circ_0001758	mmu-miR-23a-5p	ENSMUST00000000128	ENSMUSG00000000126
novel_circ_0001758	mmu-miR-23a-5p	ENSMUST00000108783	ENSMUSG00000000126
novel_circ_0001934	mmu-miR-23a-5p	ENSMUST00000000128	ENSMUSG00000000126
novel_circ_0001934	mmu-miR-23a-5p	ENSMUST00000108783	ENSMUSG00000000126
novel_circ_0001980	mmu-miR-23a-5p	ENSMUST00000000128	ENSMUSG00000000126
novel_circ_0001980	mmu-miR-23a-5p	ENSMUST00000108783	ENSMUSG00000000126
novel_circ_0002002	mmu-miR-23a-5p	ENSMUST00000000128	ENSMUSG00000000126
novel_circ_0002002	mmu-miR-23a-5p	ENSMUST00000108783	ENSMUSG00000000126
novel_circ_0002526	mmu-miR-23a-5p	ENSMUST00000000128	ENSMUSG00000000126
novel_circ_0002526	mmu-miR-23a-5p	ENSMUST00000108783	ENSMUSG00000000126
novel_circ_0002526	mmu-miR-23a-5p	ENSMUST00000000128	ENSMUSG00000000126
novel_circ_0002526	mmu-miR-23a-5p	ENSMUST00000108783	ENSMUSG00000000126
novel_circ_0002578	mmu-miR-23a-5p	ENSMUST00000000128	ENSMUSG00000000126
novel_circ_0002578	mmu-miR-23a-5p	ENSMUST00000108783	ENSMUSG00000000126
novel_circ_0002621	mmu-miR-23a-5p	ENSMUST00000000128	ENSMUSG00000000126
novel_circ_0002621	mmu-miR-23a-5p	ENSMUST00000108783	ENSMUSG00000000126
novel_circ_0002252	mmu-miR-23a-5p	ENSMUST00000000128	ENSMUSG00000000126
novel_circ_0002252	mmu-miR-23a-5p	ENSMUST00000108783	ENSMUSG00000000126
novel_circ_0002257	mmu-miR-23a-5p	ENSMUST00000000128	ENSMUSG00000000126
novel_circ_0002257	mmu-miR-23a-5p	ENSMUST00000108783	ENSMUSG00000000126
novel_circ_0002289	mmu-miR-23a-5p	ENSMUST00000000128	ENSMUSG00000000126
novel_circ_0002289	mmu-miR-23a-5p	ENSMUST00000108783	ENSMUSG00000000126
novel_circ_0002879	mmu-miR-23a-5p	ENSMUST00000000128	ENSMUSG00000000126
novel_circ_0002879	mmu-miR-23a-5p	ENSMUST00000108783	ENSMUSG00000000126
novel_circ_0002900	mmu-miR-23a-5p	ENSMUST00000000128	ENSMUSG00000000126
novel_circ_0002900	mmu-miR-23a-5p	ENSMUST00000108783	ENSMUSG00000000126
novel_circ_0002921	mmu-miR-23a-5p	ENSMUST00000000128	ENSMUSG00000000126
novel_circ_0002921	mmu-miR-23a-5p	ENSMUST00000108783	ENSMUSG00000000126
novel_circ_0002941	mmu-miR-23a-5p	ENSMUST00000000128	ENSMUSG00000000126
novel_circ_0002941	mmu-miR-23a-5p	ENSMUST00000108783	ENSMUSG00000000126
novel_circ_0002766	mmu-miR-23a-5p	ENSMUST00000000128	ENSMUSG00000000126
novel_circ_0002766	mmu-miR-23a-5p	ENSMUST00000108783	ENSMUSG00000000126
novel_circ_0002820	mmu-miR-23a-5p	ENSMUST00000000128	ENSMUSG00000000126
novel_circ_0002820	mmu-miR-23a-5p	ENSMUST00000108783	ENSMUSG00000000126
novel_circ_0003175	mmu-miR-23a-5p	ENSMUST00000000128	ENSMUSG00000000126

novel_circ_0003175	mmu-miR-23a-5p	ENSMUST00000108783	ENSMUSG00000000126
novel_circ_0003175	mmu-miR-23a-5p	ENSMUST00000000128	ENSMUSG00000000126
novel_circ_0003175	mmu-miR-23a-5p	ENSMUST00000108783	ENSMUSG00000000126
novel_circ_0003187	mmu-miR-23a-5p	ENSMUST00000000128	ENSMUSG00000000126
novel_circ_0003187	mmu-miR-23a-5p	ENSMUST00000108783	ENSMUSG00000000126
novel_circ_0003053	mmu-miR-23a-5p	ENSMUST00000000128	ENSMUSG00000000126
novel_circ_0003053	mmu-miR-23a-5p	ENSMUST00000108783	ENSMUSG00000000126
novel_circ_0003491	mmu-miR-23a-5p	ENSMUST00000000128	ENSMUSG00000000126
novel_circ_0003491	mmu-miR-23a-5p	ENSMUST00000108783	ENSMUSG00000000126
novel_circ_0000208	mmu-miR-23a-5p	ENSMUST00000000128	ENSMUSG00000000126
novel_circ_0000208	mmu-miR-23a-5p	ENSMUST00000108783	ENSMUSG00000000126
novel_circ_0000220	mmu-miR-23a-5p	ENSMUST00000000128	ENSMUSG00000000126
novel_circ_0000220	mmu-miR-23a-5p	ENSMUST00000108783	ENSMUSG00000000126
novel_circ_0000222	mmu-miR-23a-5p	ENSMUST00000000128	ENSMUSG00000000126
novel_circ_0000222	mmu-miR-23a-5p	ENSMUST00000108783	ENSMUSG00000000126
novel_circ_0000277	mmu-miR-23a-5p	ENSMUST00000000128	ENSMUSG00000000126
novel_circ_0000277	mmu-miR-23a-5p	ENSMUST00000108783	ENSMUSG00000000126
novel_circ_0000022	mmu-miR-23a-5p	ENSMUST00000000128	ENSMUSG00000000126
novel_circ_0000022	mmu-miR-23a-5p	ENSMUST00000108783	ENSMUSG00000000126
novel_circ_0000088	mmu-miR-23a-5p	ENSMUST00000000128	ENSMUSG00000000126
novel_circ_0000088	mmu-miR-23a-5p	ENSMUST00000108783	ENSMUSG00000000126
novel_circ_0000483	mmu-miR-23a-5p	ENSMUST00000000128	ENSMUSG00000000126
novel_circ_0000483	mmu-miR-23a-5p	ENSMUST00000108783	ENSMUSG00000000126
novel_circ_0000494	mmu-miR-23a-5p	ENSMUST00000000128	ENSMUSG00000000126
novel_circ_0000494	mmu-miR-23a-5p	ENSMUST00000108783	ENSMUSG00000000126
novel_circ_0000494	mmu-miR-23a-5p	ENSMUST00000000128	ENSMUSG00000000126
novel_circ_0000494	mmu-miR-23a-5p	ENSMUST00000108783	ENSMUSG00000000126
novel_circ_0000514	mmu-miR-23a-5p	ENSMUST00000000128	ENSMUSG00000000126
novel_circ_0000514	mmu-miR-23a-5p	ENSMUST00000108783	ENSMUSG00000000126
novel_circ_0000537	mmu-miR-23a-5p	ENSMUST00000000128	ENSMUSG00000000126
novel_circ_0000537	mmu-miR-23a-5p	ENSMUST00000108783	ENSMUSG00000000126
novel_circ_0000951	mmu-miR-23a-5p	ENSMUST00000000128	ENSMUSG00000000126
novel_circ_0000951	mmu-miR-23a-5p	ENSMUST00000108783	ENSMUSG00000000126
novel_circ_0000855	mmu-miR-23a-5p	ENSMUST00000000128	ENSMUSG00000000126
novel_circ_0000855	mmu-miR-23a-5p	ENSMUST00000108783	ENSMUSG00000000126
novel_circ_0001062	mmu-miR-329-3p	ENSMUST00000003659	ENSMUSG00000031849
novel_circ_0001062	mmu-miR-329-3p	ENSMUST00000212488	ENSMUSG00000031849
novel_circ_0001132	mmu-miR-329-3p	ENSMUST00000003659	ENSMUSG00000031849
novel_circ_0001132	mmu-miR-329-3p	ENSMUST00000212488	ENSMUSG00000031849
novel_circ_0001491	mmu-miR-329-3p	ENSMUST00000003659	ENSMUSG00000031849
novel_circ_0001491	mmu-miR-329-3p	ENSMUST00000212488	ENSMUSG00000031849
novel_circ_0001495	mmu-miR-329-3p	ENSMUST00000003659	ENSMUSG00000031849
novel_circ_0001495	mmu-miR-329-3p	ENSMUST00000212488	ENSMUSG00000031849
mmu_circ_0000049	mmu-miR-329-3p	ENSMUST00000003659	ENSMUSG00000031849
mmu_circ_0000049	mmu-miR-329-3p	ENSMUST00000212488	ENSMUSG00000031849
mmu_circ_0000057	mmu-miR-329-3p	ENSMUST00000003659	ENSMUSG00000031849
mmu_circ_0000057	mmu-miR-329-3p	ENSMUST00000212488	ENSMUSG00000031849
mmu_circ_0000063	mmu-miR-329-3p	ENSMUST00000003659	ENSMUSG00000031849
mmu_circ_0000063	mmu-miR-329-3p	ENSMUST00000212488	ENSMUSG00000031849
mmu_circ_0001154	mmu-miR-329-3p	ENSMUST00000003659	ENSMUSG00000031849
mmu_circ_0001154	mmu-miR-329-3p	ENSMUST00000212488	ENSMUSG00000031849
mmu_circ_0001434	mmu-miR-329-3p	ENSMUST00000003659	ENSMUSG00000031849
mmu_circ_0001434	mmu-miR-329-3p	ENSMUST00000212488	ENSMUSG00000031849
mmu_circ_0001333	mmu-miR-329-3p	ENSMUST00000003659	ENSMUSG00000031849
mmu_circ_0001333	mmu-miR-329-3p	ENSMUST00000212488	ENSMUSG00000031849
mmu_circ_0001528	mmu-miR-329-3p	ENSMUST00000003659	ENSMUSG00000031849
mmu_circ_0001528	mmu-miR-329-3p	ENSMUST00000212488	ENSMUSG00000031849
mmu_circ_0001500	mmu-miR-329-3p	ENSMUST00000003659	ENSMUSG00000031849

mmu_circ_0001500	mmu-miR-329-3p	ENSMUST00000212488	ENSMUSG00000031849
mmu_circ_0001755	mmu-miR-329-3p	ENSMUST00000003659	ENSMUSG00000031849
mmu_circ_0001755	mmu-miR-329-3p	ENSMUST00000212488	ENSMUSG00000031849
mmu_circ_0001774	mmu-miR-329-3p	ENSMUST00000003659	ENSMUSG00000031849
mmu_circ_0001774	mmu-miR-329-3p	ENSMUST00000212488	ENSMUSG00000031849
mmu_circ_0001827	mmu-miR-329-3p	ENSMUST00000003659	ENSMUSG00000031849
mmu_circ_0001827	mmu-miR-329-3p	ENSMUST00000212488	ENSMUSG00000031849
mmu_circ_0000230	mmu-miR-329-3p	ENSMUST00000003659	ENSMUSG00000031849
mmu_circ_0000230	mmu-miR-329-3p	ENSMUST00000212488	ENSMUSG00000031849
mmu_circ_0000499	mmu-miR-329-3p	ENSMUST00000003659	ENSMUSG00000031849
mmu_circ_0000499	mmu-miR-329-3p	ENSMUST00000212488	ENSMUSG00000031849
mmu_circ_0000500	mmu-miR-329-3p	ENSMUST00000003659	ENSMUSG00000031849
mmu_circ_0000500	mmu-miR-329-3p	ENSMUST00000212488	ENSMUSG00000031849
mmu_circ_0000454	mmu-miR-329-3p	ENSMUST00000003659	ENSMUSG00000031849
mmu_circ_0000454	mmu-miR-329-3p	ENSMUST00000212488	ENSMUSG00000031849
mmu_circ_0000628	mmu-miR-329-3p	ENSMUST00000003659	ENSMUSG00000031849
mmu_circ_0000628	mmu-miR-329-3p	ENSMUST00000212488	ENSMUSG00000031849
mmu_circ_0000530	mmu-miR-329-3p	ENSMUST00000003659	ENSMUSG00000031849
mmu_circ_0000530	mmu-miR-329-3p	ENSMUST00000212488	ENSMUSG00000031849
mmu_circ_0000829	mmu-miR-329-3p	ENSMUST00000003659	ENSMUSG00000031849
mmu_circ_0000829	mmu-miR-329-3p	ENSMUST00000212488	ENSMUSG00000031849
mmu_circ_0000831	mmu-miR-329-3p	ENSMUST00000003659	ENSMUSG00000031849
mmu_circ_0000831	mmu-miR-329-3p	ENSMUST00000212488	ENSMUSG00000031849
mmu_circ_0000833	mmu-miR-329-3p	ENSMUST00000003659	ENSMUSG00000031849
mmu_circ_0000833	mmu-miR-329-3p	ENSMUST00000212488	ENSMUSG00000031849
mmu_circ_0000711	mmu-miR-329-3p	ENSMUST00000003659	ENSMUSG00000031849
mmu_circ_0000711	mmu-miR-329-3p	ENSMUST00000212488	ENSMUSG00000031849
mmu_circ_0000712	mmu-miR-329-3p	ENSMUST00000003659	ENSMUSG00000031849
mmu_circ_0000712	mmu-miR-329-3p	ENSMUST00000212488	ENSMUSG00000031849
mmu_circ_0000969	mmu-miR-329-3p	ENSMUST00000003659	ENSMUSG00000031849
mmu_circ_0000969	mmu-miR-329-3p	ENSMUST00000212488	ENSMUSG00000031849
mmu_circ_0000884	mmu-miR-329-3p	ENSMUST00000003659	ENSMUSG00000031849
mmu_circ_0000884	mmu-miR-329-3p	ENSMUST00000212488	ENSMUSG00000031849
mmu_circ_0000886	mmu-miR-329-3p	ENSMUST00000003659	ENSMUSG00000031849
mmu_circ_0000886	mmu-miR-329-3p	ENSMUST00000212488	ENSMUSG00000031849
mmu_circ_0000887	mmu-miR-329-3p	ENSMUST00000003659	ENSMUSG00000031849
mmu_circ_0000887	mmu-miR-329-3p	ENSMUST00000212488	ENSMUSG00000031849
mmu_circ_0000905	mmu-miR-329-3p	ENSMUST00000003659	ENSMUSG00000031849
mmu_circ_0000905	mmu-miR-329-3p	ENSMUST00000212488	ENSMUSG00000031849
mmu_circ_0000906	mmu-miR-329-3p	ENSMUST00000003659	ENSMUSG00000031849
mmu_circ_0000906	mmu-miR-329-3p	ENSMUST00000212488	ENSMUSG00000031849
mmu_circ_0000907	mmu-miR-329-3p	ENSMUST00000003659	ENSMUSG00000031849
mmu_circ_0000907	mmu-miR-329-3p	ENSMUST00000212488	ENSMUSG00000031849
mmu_circ_0000908	mmu-miR-329-3p	ENSMUST00000003659	ENSMUSG00000031849
mmu_circ_0000908	mmu-miR-329-3p	ENSMUST00000212488	ENSMUSG00000031849
mmu_circ_0000912	mmu-miR-329-3p	ENSMUST00000003659	ENSMUSG00000031849
mmu_circ_0000912	mmu-miR-329-3p	ENSMUST00000212488	ENSMUSG00000031849
novel_circ_0001561	mmu-miR-329-3p	ENSMUST00000003659	ENSMUSG00000031849
novel_circ_0001561	mmu-miR-329-3p	ENSMUST00000212488	ENSMUSG00000031849
novel_circ_0001682	mmu-miR-329-3p	ENSMUST00000003659	ENSMUSG00000031849
novel_circ_0001682	mmu-miR-329-3p	ENSMUST00000212488	ENSMUSG00000031849
novel_circ_0001685	mmu-miR-329-3p	ENSMUST00000003659	ENSMUSG00000031849
novel_circ_0001685	mmu-miR-329-3p	ENSMUST00000212488	ENSMUSG00000031849
novel_circ_0001772	mmu-miR-329-3p	ENSMUST00000003659	ENSMUSG00000031849
novel_circ_0001772	mmu-miR-329-3p	ENSMUST00000212488	ENSMUSG00000031849
novel_circ_0001859	mmu-miR-329-3p	ENSMUST00000003659	ENSMUSG00000031849
novel_circ_0001859	mmu-miR-329-3p	ENSMUST00000212488	ENSMUSG00000031849
novel_circ_0001899	mmu-miR-329-3p	ENSMUST00000003659	ENSMUSG00000031849

novel_circ_0001899	mmu-miR-329-3p	ENSMUST00000212488	ENSMUSG00000031849
novel_circ_0002536	mmu-miR-329-3p	ENSMUST00000003659	ENSMUSG00000031849
novel_circ_0002536	mmu-miR-329-3p	ENSMUST00000212488	ENSMUSG00000031849
novel_circ_0002595	mmu-miR-329-3p	ENSMUST00000003659	ENSMUSG00000031849
novel_circ_0002595	mmu-miR-329-3p	ENSMUST00000212488	ENSMUSG00000031849
novel_circ_0002597	mmu-miR-329-3p	ENSMUST00000003659	ENSMUSG00000031849
novel_circ_0002597	mmu-miR-329-3p	ENSMUST00000212488	ENSMUSG00000031849
novel_circ_0002360	mmu-miR-329-3p	ENSMUST00000003659	ENSMUSG00000031849
novel_circ_0002360	mmu-miR-329-3p	ENSMUST00000212488	ENSMUSG00000031849
novel_circ_0002362	mmu-miR-329-3p	ENSMUST00000003659	ENSMUSG00000031849
novel_circ_0002362	mmu-miR-329-3p	ENSMUST00000212488	ENSMUSG00000031849
novel_circ_0002363	mmu-miR-329-3p	ENSMUST00000003659	ENSMUSG00000031849
novel_circ_0002363	mmu-miR-329-3p	ENSMUST00000212488	ENSMUSG00000031849
novel_circ_0002364	mmu-miR-329-3p	ENSMUST00000003659	ENSMUSG00000031849
novel_circ_0002364	mmu-miR-329-3p	ENSMUST00000212488	ENSMUSG00000031849
novel_circ_0002366	mmu-miR-329-3p	ENSMUST00000003659	ENSMUSG00000031849
novel_circ_0002366	mmu-miR-329-3p	ENSMUST00000212488	ENSMUSG00000031849
novel_circ_0002371	mmu-miR-329-3p	ENSMUST00000003659	ENSMUSG00000031849
novel_circ_0002371	mmu-miR-329-3p	ENSMUST00000212488	ENSMUSG00000031849
novel_circ_0002982	mmu-miR-329-3p	ENSMUST00000003659	ENSMUSG00000031849
novel_circ_0002982	mmu-miR-329-3p	ENSMUST00000212488	ENSMUSG00000031849
novel_circ_0002769	mmu-miR-329-3p	ENSMUST00000003659	ENSMUSG00000031849
novel_circ_0002769	mmu-miR-329-3p	ENSMUST00000212488	ENSMUSG00000031849
novel_circ_0003177	mmu-miR-329-3p	ENSMUST00000003659	ENSMUSG00000031849
novel_circ_0003177	mmu-miR-329-3p	ENSMUST00000212488	ENSMUSG00000031849
novel_circ_0003244	mmu-miR-329-3p	ENSMUST00000003659	ENSMUSG00000031849
novel_circ_0003244	mmu-miR-329-3p	ENSMUST00000212488	ENSMUSG00000031849
novel_circ_0003298	mmu-miR-329-3p	ENSMUST00000003659	ENSMUSG00000031849
novel_circ_0003298	mmu-miR-329-3p	ENSMUST00000212488	ENSMUSG00000031849
novel_circ_0003303	mmu-miR-329-3p	ENSMUST00000003659	ENSMUSG00000031849
novel_circ_0003303	mmu-miR-329-3p	ENSMUST00000212488	ENSMUSG00000031849
novel_circ_0003304	mmu-miR-329-3p	ENSMUST00000003659	ENSMUSG00000031849
novel_circ_0003304	mmu-miR-329-3p	ENSMUST00000212488	ENSMUSG00000031849
novel_circ_0003045	mmu-miR-329-3p	ENSMUST00000003659	ENSMUSG00000031849
novel_circ_0003045	mmu-miR-329-3p	ENSMUST00000212488	ENSMUSG00000031849
novel_circ_0000022	mmu-miR-329-3p	ENSMUST00000003659	ENSMUSG00000031849
novel_circ_0000022	mmu-miR-329-3p	ENSMUST00000212488	ENSMUSG00000031849
novel_circ_0000702	mmu-miR-329-3p	ENSMUST00000003659	ENSMUSG00000031849
novel_circ_0000702	mmu-miR-329-3p	ENSMUST00000212488	ENSMUSG00000031849
novel_circ_0000560	mmu-miR-329-3p	ENSMUST00000003659	ENSMUSG00000031849
novel_circ_0000560	mmu-miR-329-3p	ENSMUST00000212488	ENSMUSG00000031849
novel_circ_0000560	mmu-miR-329-3p	ENSMUST00000003659	ENSMUSG00000031849
novel_circ_0000560	mmu-miR-329-3p	ENSMUST00000212488	ENSMUSG00000031849
novel_circ_0000595	mmu-miR-329-3p	ENSMUST00000003659	ENSMUSG00000031849
novel_circ_0000595	mmu-miR-329-3p	ENSMUST00000212488	ENSMUSG00000031849
novel_circ_0000959	mmu-miR-329-3p	ENSMUST00000003659	ENSMUSG00000031849
novel_circ_0000959	mmu-miR-329-3p	ENSMUST00000212488	ENSMUSG00000031849
novel_circ_0001048	mmu-miR-329-3p	ENSMUST00000003659	ENSMUSG00000031849
novel_circ_0001048	mmu-miR-329-3p	ENSMUST00000212488	ENSMUSG00000031849
novel_circ_0000793	mmu-miR-329-3p	ENSMUST00000003659	ENSMUSG00000031849
novel_circ_0000793	mmu-miR-329-3p	ENSMUST00000212488	ENSMUSG00000031849
novel_circ_0001371	mmu-miR-339-5p	ENSMUST00000000128	ENSMUSG00000000126
novel_circ_0001371	mmu-miR-339-5p	ENSMUST00000108783	ENSMUSG00000000126
novel_circ_0001413	mmu-miR-339-5p	ENSMUST00000000128	ENSMUSG00000000126
novel_circ_0001413	mmu-miR-339-5p	ENSMUST00000108783	ENSMUSG00000000126
novel_circ_0001416	mmu-miR-339-5p	ENSMUST00000000128	ENSMUSG00000000126
novel_circ_0001416	mmu-miR-339-5p	ENSMUST00000108783	ENSMUSG00000000126
mmu_circ_0000028	mmu-miR-339-5p	ENSMUST00000000128	ENSMUSG00000000126

mmu_circ_0000028	mmu-miR-339-5p	ENSMUST00000108783	ENSMUSG00000000126
mmu_circ_0001123	mmu-miR-339-5p	ENSMUST00000000128	ENSMUSG00000000126
mmu_circ_0001123	mmu-miR-339-5p	ENSMUST00000108783	ENSMUSG00000000126
mmu_circ_0001376	mmu-miR-339-5p	ENSMUST00000000128	ENSMUSG00000000126
mmu_circ_0001376	mmu-miR-339-5p	ENSMUST00000108783	ENSMUSG00000000126
mmu_circ_0001528	mmu-miR-339-5p	ENSMUST00000000128	ENSMUSG00000000126
mmu_circ_0001528	mmu-miR-339-5p	ENSMUST00000108783	ENSMUSG00000000126
mmu_circ_0001537	mmu-miR-339-5p	ENSMUST00000000128	ENSMUSG00000000126
mmu_circ_0001537	mmu-miR-339-5p	ENSMUST00000108783	ENSMUSG00000000126
mmu_circ_0001454	mmu-miR-339-5p	ENSMUST00000000128	ENSMUSG00000000126
mmu_circ_0001454	mmu-miR-339-5p	ENSMUST00000108783	ENSMUSG00000000126
mmu_circ_0001499	mmu-miR-339-5p	ENSMUST00000000128	ENSMUSG00000000126
mmu_circ_0001499	mmu-miR-339-5p	ENSMUST00000108783	ENSMUSG00000000126
mmu_circ_0001500	mmu-miR-339-5p	ENSMUST00000000128	ENSMUSG00000000126
mmu_circ_0001500	mmu-miR-339-5p	ENSMUST00000108783	ENSMUSG00000000126
mmu_circ_0001827	mmu-miR-339-5p	ENSMUST00000000128	ENSMUSG00000000126
mmu_circ_0001827	mmu-miR-339-5p	ENSMUST00000108783	ENSMUSG00000000126
mmu_circ_0000239	mmu-miR-339-5p	ENSMUST00000000128	ENSMUSG00000000126
mmu_circ_0000239	mmu-miR-339-5p	ENSMUST00000108783	ENSMUSG00000000126
mmu_circ_0000151	mmu-miR-339-5p	ENSMUST00000000128	ENSMUSG00000000126
mmu_circ_0000151	mmu-miR-339-5p	ENSMUST00000108783	ENSMUSG00000000126
mmu_circ_0000499	mmu-miR-339-5p	ENSMUST00000000128	ENSMUSG00000000126
mmu_circ_0000499	mmu-miR-339-5p	ENSMUST00000108783	ENSMUSG00000000126
mmu_circ_0000500	mmu-miR-339-5p	ENSMUST00000000128	ENSMUSG00000000126
mmu_circ_0000500	mmu-miR-339-5p	ENSMUST00000108783	ENSMUSG00000000126
mmu_circ_0000402	mmu-miR-339-5p	ENSMUST00000000128	ENSMUSG00000000126
mmu_circ_0000402	mmu-miR-339-5p	ENSMUST00000108783	ENSMUSG00000000126
mmu_circ_0000530	mmu-miR-339-5p	ENSMUST00000000128	ENSMUSG00000000126
mmu_circ_0000530	mmu-miR-339-5p	ENSMUST00000108783	ENSMUSG00000000126
mmu_circ_0000828	mmu-miR-339-5p	ENSMUST00000000128	ENSMUSG00000000126
mmu_circ_0000828	mmu-miR-339-5p	ENSMUST00000108783	ENSMUSG00000000126
mmu_circ_0000829	mmu-miR-339-5p	ENSMUST00000000128	ENSMUSG00000000126
mmu_circ_0000829	mmu-miR-339-5p	ENSMUST00000108783	ENSMUSG00000000126
mmu_circ_0000711	mmu-miR-339-5p	ENSMUST00000000128	ENSMUSG00000000126
mmu_circ_0000711	mmu-miR-339-5p	ENSMUST00000108783	ENSMUSG00000000126
mmu_circ_0000712	mmu-miR-339-5p	ENSMUST00000000128	ENSMUSG00000000126
mmu_circ_0000712	mmu-miR-339-5p	ENSMUST00000108783	ENSMUSG00000000126
mmu_circ_0000713	mmu-miR-339-5p	ENSMUST00000000128	ENSMUSG00000000126
mmu_circ_0000713	mmu-miR-339-5p	ENSMUST00000108783	ENSMUSG00000000126
mmu_circ_0000714	mmu-miR-339-5p	ENSMUST00000000128	ENSMUSG00000000126
mmu_circ_0000714	mmu-miR-339-5p	ENSMUST00000108783	ENSMUSG00000000126
mmu_circ_0000950	mmu-miR-339-5p	ENSMUST00000000128	ENSMUSG00000000126
mmu_circ_0000950	mmu-miR-339-5p	ENSMUST00000108783	ENSMUSG00000000126
mmu_circ_0000962	mmu-miR-339-5p	ENSMUST00000000128	ENSMUSG00000000126
mmu_circ_0000962	mmu-miR-339-5p	ENSMUST00000108783	ENSMUSG00000000126
mmu_circ_0000969	mmu-miR-339-5p	ENSMUST00000000128	ENSMUSG00000000126
mmu_circ_0000969	mmu-miR-339-5p	ENSMUST00000108783	ENSMUSG00000000126
mmu_circ_0000884	mmu-miR-339-5p	ENSMUST00000000128	ENSMUSG00000000126
mmu_circ_0000884	mmu-miR-339-5p	ENSMUST00000108783	ENSMUSG00000000126
mmu_circ_0000886	mmu-miR-339-5p	ENSMUST00000000128	ENSMUSG00000000126
mmu_circ_0000886	mmu-miR-339-5p	ENSMUST00000108783	ENSMUSG00000000126
mmu_circ_0000887	mmu-miR-339-5p	ENSMUST00000000128	ENSMUSG00000000126
mmu_circ_0000887	mmu-miR-339-5p	ENSMUST00000108783	ENSMUSG00000000126
mmu_circ_0000905	mmu-miR-339-5p	ENSMUST00000000128	ENSMUSG00000000126
mmu_circ_0000905	mmu-miR-339-5p	ENSMUST00000108783	ENSMUSG00000000126
mmu_circ_0000906	mmu-miR-339-5p	ENSMUST00000000128	ENSMUSG00000000126
mmu_circ_0000906	mmu-miR-339-5p	ENSMUST00000108783	ENSMUSG00000000126
mmu_circ_0000907	mmu-miR-339-5p	ENSMUST00000000128	ENSMUSG00000000126

mmu_circ_0000907	mmu-miR-339-5p	ENSMUST00000108783	ENSMUSG00000000126
novel_circ_0002115	mmu-miR-339-5p	ENSMUST00000000128	ENSMUSG00000000126
novel_circ_0002115	mmu-miR-339-5p	ENSMUST00000108783	ENSMUSG00000000126
novel_circ_0001913	mmu-miR-339-5p	ENSMUST00000000128	ENSMUSG00000000126
novel_circ_0001913	mmu-miR-339-5p	ENSMUST00000108783	ENSMUSG00000000126
novel_circ_0002019	mmu-miR-339-5p	ENSMUST00000000128	ENSMUSG00000000126
novel_circ_0002019	mmu-miR-339-5p	ENSMUST00000108783	ENSMUSG00000000126
novel_circ_0002412	mmu-miR-339-5p	ENSMUST00000000128	ENSMUSG00000000126
novel_circ_0002412	mmu-miR-339-5p	ENSMUST00000108783	ENSMUSG00000000126
novel_circ_0002221	mmu-miR-339-5p	ENSMUST00000000128	ENSMUSG00000000126
novel_circ_0002221	mmu-miR-339-5p	ENSMUST00000108783	ENSMUSG00000000126
novel_circ_0002246	mmu-miR-339-5p	ENSMUST00000000128	ENSMUSG00000000126
novel_circ_0002246	mmu-miR-339-5p	ENSMUST00000108783	ENSMUSG00000000126
novel_circ_0002292	mmu-miR-339-5p	ENSMUST00000000128	ENSMUSG00000000126
novel_circ_0002292	mmu-miR-339-5p	ENSMUST00000108783	ENSMUSG00000000126
novel_circ_0002392	mmu-miR-339-5p	ENSMUST00000000128	ENSMUSG00000000126
novel_circ_0002392	mmu-miR-339-5p	ENSMUST00000108783	ENSMUSG00000000126
novel_circ_0002392	mmu-miR-339-5p	ENSMUST00000000128	ENSMUSG00000000126
novel_circ_0002392	mmu-miR-339-5p	ENSMUST00000108783	ENSMUSG00000000126
novel_circ_0002924	mmu-miR-339-5p	ENSMUST00000000128	ENSMUSG00000000126
novel_circ_0002924	mmu-miR-339-5p	ENSMUST00000108783	ENSMUSG00000000126
novel_circ_0002925	mmu-miR-339-5p	ENSMUST00000000128	ENSMUSG00000000126
novel_circ_0002925	mmu-miR-339-5p	ENSMUST00000108783	ENSMUSG00000000126
novel_circ_0002946	mmu-miR-339-5p	ENSMUST00000000128	ENSMUSG00000000126
novel_circ_0002946	mmu-miR-339-5p	ENSMUST00000108783	ENSMUSG00000000126
novel_circ_0002631	mmu-miR-339-5p	ENSMUST00000000128	ENSMUSG00000000126
novel_circ_0002631	mmu-miR-339-5p	ENSMUST00000108783	ENSMUSG00000000126
novel_circ_0003393	mmu-miR-339-5p	ENSMUST00000000128	ENSMUSG00000000126
novel_circ_0003393	mmu-miR-339-5p	ENSMUST00000108783	ENSMUSG00000000126
novel_circ_0003083	mmu-miR-339-5p	ENSMUST00000000128	ENSMUSG00000000126
novel_circ_0003083	mmu-miR-339-5p	ENSMUST00000108783	ENSMUSG00000000126
novel_circ_0000447	mmu-miR-339-5p	ENSMUST00000000128	ENSMUSG00000000126
novel_circ_0000447	mmu-miR-339-5p	ENSMUST00000108783	ENSMUSG00000000126
novel_circ_0000078	mmu-miR-339-5p	ENSMUST00000000128	ENSMUSG00000000126
novel_circ_0000078	mmu-miR-339-5p	ENSMUST00000108783	ENSMUSG00000000126
novel_circ_0000687	mmu-miR-339-5p	ENSMUST00000000128	ENSMUSG00000000126
novel_circ_0000687	mmu-miR-339-5p	ENSMUST00000108783	ENSMUSG00000000126
novel_circ_0000453	mmu-miR-339-5p	ENSMUST00000000128	ENSMUSG00000000126
novel_circ_0000453	mmu-miR-339-5p	ENSMUST00000108783	ENSMUSG00000000126
novel_circ_0001025	mmu-miR-339-5p	ENSMUST00000000128	ENSMUSG00000000126
novel_circ_0001025	mmu-miR-339-5p	ENSMUST00000108783	ENSMUSG00000000126
novel_circ_0000868	mmu-miR-339-5p	ENSMUST00000000128	ENSMUSG00000000126
novel_circ_0000868	mmu-miR-339-5p	ENSMUST00000108783	ENSMUSG00000000126
novel_circ_0001224	mmu-miR-17-3p	ENSMUST00000225594	ENSMUSG00000017491
novel_circ_0001230	mmu-miR-17-3p	ENSMUST00000225594	ENSMUSG00000017491
novel_circ_0001290	mmu-miR-17-3p	ENSMUST00000225594	ENSMUSG00000017491
novel_circ_0001291	mmu-miR-17-3p	ENSMUST00000225594	ENSMUSG00000017491
novel_circ_0001076	mmu-miR-17-3p	ENSMUST00000225594	ENSMUSG00000017491
novel_circ_0001097	mmu-miR-17-3p	ENSMUST00000225594	ENSMUSG00000017491
novel_circ_0001146	mmu-miR-17-3p	ENSMUST00000225594	ENSMUSG00000017491
novel_circ_0001148	mmu-miR-17-3p	ENSMUST00000225594	ENSMUSG00000017491
novel_circ_0001464	mmu-miR-17-3p	ENSMUST00000225594	ENSMUSG00000017491
novel_circ_0001495	mmu-miR-17-3p	ENSMUST00000225594	ENSMUSG00000017491
novel_circ_0001498	mmu-miR-17-3p	ENSMUST00000225594	ENSMUSG00000017491
novel_circ_0001498	mmu-miR-17-3p	ENSMUST00000225594	ENSMUSG00000017491
novel_circ_0001499	mmu-miR-17-3p	ENSMUST00000225594	ENSMUSG00000017491
novel_circ_0001499	mmu-miR-17-3p	ENSMUST00000225594	ENSMUSG00000017491
mmu_circ_0000048	mmu-miR-17-3p	ENSMUST00000225594	ENSMUSG00000017491

mmu_circ_0000049	mmu-miR-17-3p	ENSMUST00000225594	ENSMUSG00000017491
mmu_circ_0001123	mmu-miR-17-3p	ENSMUST00000225594	ENSMUSG00000017491
mmu_circ_0001097	mmu-miR-17-3p	ENSMUST00000225594	ENSMUSG00000017491
mmu_circ_0001103	mmu-miR-17-3p	ENSMUST00000225594	ENSMUSG00000017491
mmu_circ_0001021	mmu-miR-17-3p	ENSMUST00000225594	ENSMUSG00000017491
mmu_circ_0001021	mmu-miR-17-3p	ENSMUST00000225594	ENSMUSG00000017491
mmu_circ_0001033	mmu-miR-17-3p	ENSMUST00000225594	ENSMUSG00000017491
mmu_circ_0001376	mmu-miR-17-3p	ENSMUST00000225594	ENSMUSG00000017491
mmu_circ_0001387	mmu-miR-17-3p	ENSMUST00000225594	ENSMUSG00000017491
mmu_circ_0001390	mmu-miR-17-3p	ENSMUST00000225594	ENSMUSG00000017491
mmu_circ_0001396	mmu-miR-17-3p	ENSMUST00000225594	ENSMUSG00000017491
mmu_circ_0001396	mmu-miR-17-3p	ENSMUST00000225594	ENSMUSG00000017491
mmu_circ_0001332	mmu-miR-17-3p	ENSMUST00000225594	ENSMUSG00000017491
mmu_circ_0001333	mmu-miR-17-3p	ENSMUST00000225594	ENSMUSG00000017491
mmu_circ_0001311	mmu-miR-17-3p	ENSMUST00000225594	ENSMUSG00000017491
mmu_circ_0001190	mmu-miR-17-3p	ENSMUST00000225594	ENSMUSG00000017491
mmu_circ_0001222	mmu-miR-17-3p	ENSMUST00000225594	ENSMUSG00000017491
mmu_circ_0001632	mmu-miR-17-3p	ENSMUST00000225594	ENSMUSG00000017491
mmu_circ_0001633	mmu-miR-17-3p	ENSMUST00000225594	ENSMUSG00000017491
mmu_circ_0001645	mmu-miR-17-3p	ENSMUST00000225594	ENSMUSG00000017491
mmu_circ_0001645	mmu-miR-17-3p	ENSMUST00000225594	ENSMUSG00000017491
mmu_circ_0001589	mmu-miR-17-3p	ENSMUST00000225594	ENSMUSG00000017491
mmu_circ_0001605	mmu-miR-17-3p	ENSMUST00000225594	ENSMUSG00000017491
mmu_circ_0001516	mmu-miR-17-3p	ENSMUST00000225594	ENSMUSG00000017491
mmu_circ_0001518	mmu-miR-17-3p	ENSMUST00000225594	ENSMUSG00000017491
mmu_circ_0001436	mmu-miR-17-3p	ENSMUST00000225594	ENSMUSG00000017491
mmu_circ_0001470	mmu-miR-17-3p	ENSMUST00000225594	ENSMUSG00000017491
mmu_circ_0001500	mmu-miR-17-3p	ENSMUST00000225594	ENSMUSG00000017491
mmu_circ_0001853	mmu-miR-17-3p	ENSMUST00000225594	ENSMUSG00000017491
mmu_circ_0001755	mmu-miR-17-3p	ENSMUST00000225594	ENSMUSG00000017491
mmu_circ_0001767	mmu-miR-17-3p	ENSMUST00000225594	ENSMUSG00000017491
mmu_circ_0001821	mmu-miR-17-3p	ENSMUST00000225594	ENSMUSG00000017491
mmu_circ_0001829	mmu-miR-17-3p	ENSMUST00000225594	ENSMUSG00000017491
mmu_circ_0001830	mmu-miR-17-3p	ENSMUST00000225594	ENSMUSG00000017491
mmu_circ_0000230	mmu-miR-17-3p	ENSMUST00000225594	ENSMUSG00000017491
mmu_circ_0000277	mmu-miR-17-3p	ENSMUST00000225594	ENSMUSG00000017491
mmu_circ_0000284	mmu-miR-17-3p	ENSMUST00000225594	ENSMUSG00000017491
mmu_circ_0000172	mmu-miR-17-3p	ENSMUST00000225594	ENSMUSG00000017491
mmu_circ_0000173	mmu-miR-17-3p	ENSMUST00000225594	ENSMUSG00000017491
mmu_circ_0000499	mmu-miR-17-3p	ENSMUST00000225594	ENSMUSG00000017491
mmu_circ_0000371	mmu-miR-17-3p	ENSMUST00000225594	ENSMUSG00000017491
mmu_circ_0000372	mmu-miR-17-3p	ENSMUST00000225594	ENSMUSG00000017491
mmu_circ_0000383	mmu-miR-17-3p	ENSMUST00000225594	ENSMUSG00000017491
mmu_circ_0000399	mmu-miR-17-3p	ENSMUST00000225594	ENSMUSG00000017491
mmu_circ_0000402	mmu-miR-17-3p	ENSMUST00000225594	ENSMUSG00000017491
mmu_circ_0000644	mmu-miR-17-3p	ENSMUST00000225594	ENSMUSG00000017491
mmu_circ_0000530	mmu-miR-17-3p	ENSMUST00000225594	ENSMUSG00000017491
mmu_circ_0000541	mmu-miR-17-3p	ENSMUST00000225594	ENSMUSG00000017491
mmu_circ_0000738	mmu-miR-17-3p	ENSMUST00000225594	ENSMUSG00000017491
mmu_circ_0000802	mmu-miR-17-3p	ENSMUST00000225594	ENSMUSG00000017491
mmu_circ_0000829	mmu-miR-17-3p	ENSMUST00000225594	ENSMUSG00000017491
mmu_circ_0000831	mmu-miR-17-3p	ENSMUST00000225594	ENSMUSG00000017491
mmu_circ_0000833	mmu-miR-17-3p	ENSMUST00000225594	ENSMUSG00000017491
mmu_circ_0000684	mmu-miR-17-3p	ENSMUST00000225594	ENSMUSG00000017491
mmu_circ_0000684	mmu-miR-17-3p	ENSMUST00000225594	ENSMUSG00000017491
mmu_circ_0000705	mmu-miR-17-3p	ENSMUST00000225594	ENSMUSG00000017491
mmu_circ_0000711	mmu-miR-17-3p	ENSMUST00000225594	ENSMUSG00000017491
mmu_circ_0000712	mmu-miR-17-3p	ENSMUST00000225594	ENSMUSG00000017491

mmu_circ_0000714	mmu-miR-17-3p	ENSMUST00000225594	ENSMUSG00000017491
mmu_circ_0000917	mmu-miR-17-3p	ENSMUST00000225594	ENSMUSG00000017491
mmu_circ_0000918	mmu-miR-17-3p	ENSMUST00000225594	ENSMUSG00000017491
mmu_circ_0000919	mmu-miR-17-3p	ENSMUST00000225594	ENSMUSG00000017491
mmu_circ_0000921	mmu-miR-17-3p	ENSMUST00000225594	ENSMUSG00000017491
mmu_circ_0000931	mmu-miR-17-3p	ENSMUST00000225594	ENSMUSG00000017491
mmu_circ_0000840	mmu-miR-17-3p	ENSMUST00000225594	ENSMUSG00000017491
mmu_circ_0000894	mmu-miR-17-3p	ENSMUST00000225594	ENSMUSG00000017491
mmu_circ_0000894	mmu-miR-17-3p	ENSMUST00000225594	ENSMUSG00000017491
mmu_circ_0000906	mmu-miR-17-3p	ENSMUST00000225594	ENSMUSG00000017491
mmu_circ_0000907	mmu-miR-17-3p	ENSMUST00000225594	ENSMUSG00000017491
mmu_circ_0000908	mmu-miR-17-3p	ENSMUST00000225594	ENSMUSG00000017491
mmu_circ_0000912	mmu-miR-17-3p	ENSMUST00000225594	ENSMUSG00000017491
novel_circ_0001570	mmu-miR-17-3p	ENSMUST00000225594	ENSMUSG00000017491
novel_circ_0001570	mmu-miR-17-3p	ENSMUST00000225594	ENSMUSG00000017491
novel_circ_0001679	mmu-miR-17-3p	ENSMUST00000225594	ENSMUSG00000017491
novel_circ_0001679	mmu-miR-17-3p	ENSMUST00000225594	ENSMUSG00000017491
novel_circ_0001680	mmu-miR-17-3p	ENSMUST00000225594	ENSMUSG00000017491
novel_circ_0001686	mmu-miR-17-3p	ENSMUST00000225594	ENSMUSG00000017491
novel_circ_0001746	mmu-miR-17-3p	ENSMUST00000225594	ENSMUSG00000017491
novel_circ_0001752	mmu-miR-17-3p	ENSMUST00000225594	ENSMUSG00000017491
novel_circ_0001772	mmu-miR-17-3p	ENSMUST00000225594	ENSMUSG00000017491
novel_circ_0001836	mmu-miR-17-3p	ENSMUST00000225594	ENSMUSG00000017491
novel_circ_0001841	mmu-miR-17-3p	ENSMUST00000225594	ENSMUSG00000017491
novel_circ_0001905	mmu-miR-17-3p	ENSMUST00000225594	ENSMUSG00000017491
novel_circ_0001940	mmu-miR-17-3p	ENSMUST00000225594	ENSMUSG00000017491
novel_circ_0001990	mmu-miR-17-3p	ENSMUST00000225594	ENSMUSG00000017491
novel_circ_0001990	mmu-miR-17-3p	ENSMUST00000225594	ENSMUSG00000017491
novel_circ_0002003	mmu-miR-17-3p	ENSMUST00000225594	ENSMUSG00000017491
novel_circ_0002491	mmu-miR-17-3p	ENSMUST00000225594	ENSMUSG00000017491
novel_circ_0002493	mmu-miR-17-3p	ENSMUST00000225594	ENSMUSG00000017491
novel_circ_0002497	mmu-miR-17-3p	ENSMUST00000225594	ENSMUSG00000017491
novel_circ_0002508	mmu-miR-17-3p	ENSMUST00000225594	ENSMUSG00000017491
novel_circ_0002577	mmu-miR-17-3p	ENSMUST00000225594	ENSMUSG00000017491
novel_circ_0002582	mmu-miR-17-3p	ENSMUST00000225594	ENSMUSG00000017491
novel_circ_0002582	mmu-miR-17-3p	ENSMUST00000225594	ENSMUSG00000017491
novel_circ_0002597	mmu-miR-17-3p	ENSMUST00000225594	ENSMUSG00000017491
novel_circ_0002598	mmu-miR-17-3p	ENSMUST00000225594	ENSMUSG00000017491
novel_circ_0002598	mmu-miR-17-3p	ENSMUST00000225594	ENSMUSG00000017491
novel_circ_0002221	mmu-miR-17-3p	ENSMUST00000225594	ENSMUSG00000017491
novel_circ_0002233	mmu-miR-17-3p	ENSMUST00000225594	ENSMUSG00000017491
novel_circ_0002233	mmu-miR-17-3p	ENSMUST00000225594	ENSMUSG00000017491
novel_circ_0002274	mmu-miR-17-3p	ENSMUST00000225594	ENSMUSG00000017491
novel_circ_0002283	mmu-miR-17-3p	ENSMUST00000225594	ENSMUSG00000017491
novel_circ_0002289	mmu-miR-17-3p	ENSMUST00000225594	ENSMUSG00000017491
novel_circ_0002294	mmu-miR-17-3p	ENSMUST00000225594	ENSMUSG00000017491
novel_circ_0002327	mmu-miR-17-3p	ENSMUST00000225594	ENSMUSG00000017491
novel_circ_0002331	mmu-miR-17-3p	ENSMUST00000225594	ENSMUSG00000017491
novel_circ_0002332	mmu-miR-17-3p	ENSMUST00000225594	ENSMUSG00000017491
novel_circ_0002368	mmu-miR-17-3p	ENSMUST00000225594	ENSMUSG00000017491
novel_circ_0002368	mmu-miR-17-3p	ENSMUST00000225594	ENSMUSG00000017491
novel_circ_0002399	mmu-miR-17-3p	ENSMUST00000225594	ENSMUSG00000017491
novel_circ_0002400	mmu-miR-17-3p	ENSMUST00000225594	ENSMUSG00000017491
novel_circ_0002861	mmu-miR-17-3p	ENSMUST00000225594	ENSMUSG00000017491
novel_circ_0002861	mmu-miR-17-3p	ENSMUST00000225594	ENSMUSG00000017491
novel_circ_0002890	mmu-miR-17-3p	ENSMUST00000225594	ENSMUSG00000017491
novel_circ_0002921	mmu-miR-17-3p	ENSMUST00000225594	ENSMUSG00000017491
novel_circ_0002695	mmu-miR-17-3p	ENSMUST00000225594	ENSMUSG00000017491

novel_circ_0002782	mmu-miR-17-3p	ENSMUST00000225594	ENSMUSG00000017491
novel_circ_0002816	mmu-miR-17-3p	ENSMUST00000225594	ENSMUSG00000017491
novel_circ_0003261	mmu-miR-17-3p	ENSMUST00000225594	ENSMUSG00000017491
novel_circ_0003262	mmu-miR-17-3p	ENSMUST00000225594	ENSMUSG00000017491
novel_circ_0003270	mmu-miR-17-3p	ENSMUST00000225594	ENSMUSG00000017491
novel_circ_0003288	mmu-miR-17-3p	ENSMUST00000225594	ENSMUSG00000017491
novel_circ_0003293	mmu-miR-17-3p	ENSMUST00000225594	ENSMUSG00000017491
novel_circ_0003302	mmu-miR-17-3p	ENSMUST00000225594	ENSMUSG00000017491
novel_circ_0003316	mmu-miR-17-3p	ENSMUST00000225594	ENSMUSG00000017491
novel_circ_0003316	mmu-miR-17-3p	ENSMUST00000225594	ENSMUSG00000017491
novel_circ_0003386	mmu-miR-17-3p	ENSMUST00000225594	ENSMUSG00000017491
novel_circ_0003419	mmu-miR-17-3p	ENSMUST00000225594	ENSMUSG00000017491
novel_circ_0003420	mmu-miR-17-3p	ENSMUST00000225594	ENSMUSG00000017491
novel_circ_0003472	mmu-miR-17-3p	ENSMUST00000225594	ENSMUSG00000017491
novel_circ_0000218	mmu-miR-17-3p	ENSMUST00000225594	ENSMUSG00000017491
novel_circ_0000244	mmu-miR-17-3p	ENSMUST00000225594	ENSMUSG00000017491
novel_circ_0000267	mmu-miR-17-3p	ENSMUST00000225594	ENSMUSG00000017491
novel_circ_0000284	mmu-miR-17-3p	ENSMUST00000225594	ENSMUSG00000017491
novel_circ_0000315	mmu-miR-17-3p	ENSMUST00000225594	ENSMUSG00000017491
novel_circ_0000344	mmu-miR-17-3p	ENSMUST00000225594	ENSMUSG00000017491
novel_circ_0000009	mmu-miR-17-3p	ENSMUST00000225594	ENSMUSG00000017491
novel_circ_0000010	mmu-miR-17-3p	ENSMUST00000225594	ENSMUSG00000017491
novel_circ_0000029	mmu-miR-17-3p	ENSMUST00000225594	ENSMUSG00000017491
novel_circ_0000030	mmu-miR-17-3p	ENSMUST00000225594	ENSMUSG00000017491
novel_circ_0000095	mmu-miR-17-3p	ENSMUST00000225594	ENSMUSG00000017491
novel_circ_0000113	mmu-miR-17-3p	ENSMUST00000225594	ENSMUSG00000017491
novel_circ_0000113	mmu-miR-17-3p	ENSMUST00000225594	ENSMUSG00000017491
novel_circ_0000125	mmu-miR-17-3p	ENSMUST00000225594	ENSMUSG00000017491
novel_circ_0000125	mmu-miR-17-3p	ENSMUST00000225594	ENSMUSG00000017491
novel_circ_0000129	mmu-miR-17-3p	ENSMUST00000225594	ENSMUSG00000017491
novel_circ_0000145	mmu-miR-17-3p	ENSMUST00000225594	ENSMUSG00000017491
novel_circ_0000153	mmu-miR-17-3p	ENSMUST00000225594	ENSMUSG00000017491
novel_circ_0000668	mmu-miR-17-3p	ENSMUST00000225594	ENSMUSG00000017491
novel_circ_0000680	mmu-miR-17-3p	ENSMUST00000225594	ENSMUSG00000017491
novel_circ_0000680	mmu-miR-17-3p	ENSMUST00000225594	ENSMUSG00000017491
novel_circ_0000682	mmu-miR-17-3p	ENSMUST00000225594	ENSMUSG00000017491
novel_circ_0000698	mmu-miR-17-3p	ENSMUST00000225594	ENSMUSG00000017491
novel_circ_0000766	mmu-miR-17-3p	ENSMUST00000225594	ENSMUSG00000017491
novel_circ_0000771	mmu-miR-17-3p	ENSMUST00000225594	ENSMUSG00000017491
novel_circ_0000474	mmu-miR-17-3p	ENSMUST00000225594	ENSMUSG00000017491
novel_circ_0000486	mmu-miR-17-3p	ENSMUST00000225594	ENSMUSG00000017491
novel_circ_0000486	mmu-miR-17-3p	ENSMUST00000225594	ENSMUSG00000017491
novel_circ_0000501	mmu-miR-17-3p	ENSMUST00000225594	ENSMUSG00000017491
novel_circ_0000556	mmu-miR-17-3p	ENSMUST00000225594	ENSMUSG00000017491
novel_circ_0000556	mmu-miR-17-3p	ENSMUST00000225594	ENSMUSG00000017491
novel_circ_0000571	mmu-miR-17-3p	ENSMUST00000225594	ENSMUSG00000017491
novel_circ_0000589	mmu-miR-17-3p	ENSMUST00000225594	ENSMUSG00000017491
novel_circ_0000956	mmu-miR-17-3p	ENSMUST00000225594	ENSMUSG00000017491
novel_circ_0000989	mmu-miR-17-3p	ENSMUST00000225594	ENSMUSG00000017491
novel_circ_0000856	mmu-miR-17-3p	ENSMUST00000225594	ENSMUSG00000017491
novel_circ_0000896	mmu-miR-17-3p	ENSMUST00000225594	ENSMUSG00000017491
novel_circ_0000902	mmu-miR-17-3p	ENSMUST00000225594	ENSMUSG00000017491
novel_circ_0000902	mmu-miR-17-3p	ENSMUST00000225594	ENSMUSG00000017491
novel_circ_0000903	mmu-miR-17-3p	ENSMUST00000225594	ENSMUSG00000017491
novel_circ_0000903	mmu-miR-17-3p	ENSMUST00000225594	ENSMUSG00000017491
novel_circ_0001119	mmu-miR-128-2-	ENSMUST00000213072	ENSMUSG00000031849
novel_circ_0001141	mmu-miR-128-2-	ENSMUST00000213072	ENSMUSG00000031849
novel_circ_0001142	mmu-miR-128-2-	ENSMUST00000213072	ENSMUSG00000031849

novel_circ_0001142	mmu-miR-128-2-	ENSMUST00000213072	ENSMUSG00000031849
novel_circ_0001501	mmu-miR-128-2-	ENSMUST00000213072	ENSMUSG00000031849
mmu_circ_0000052	mmu-miR-128-2-	ENSMUST00000213072	ENSMUSG00000031849
mmu_circ_0001123	mmu-miR-128-2-	ENSMUST00000213072	ENSMUSG00000031849
mmu_circ_0001376	mmu-miR-128-2-	ENSMUST00000213072	ENSMUSG00000031849
mmu_circ_0001732	mmu-miR-128-2-	ENSMUST00000213072	ENSMUSG00000031849
mmu_circ_0001718	mmu-miR-128-2-	ENSMUST00000213072	ENSMUSG00000031849
mmu_circ_0001894	mmu-miR-128-2-	ENSMUST00000213072	ENSMUSG00000031849
mmu_circ_0000215	mmu-miR-128-2-	ENSMUST00000213072	ENSMUSG00000031849
mmu_circ_0000831	mmu-miR-128-2-	ENSMUST00000213072	ENSMUSG00000031849
novel_circ_0001908	mmu-miR-128-2-	ENSMUST00000213072	ENSMUSG00000031849
novel_circ_0001943	mmu-miR-128-2-	ENSMUST00000213072	ENSMUSG00000031849
novel_circ_0002494	mmu-miR-128-2-	ENSMUST00000213072	ENSMUSG00000031849
novel_circ_0002246	mmu-miR-128-2-	ENSMUST00000213072	ENSMUSG00000031849
novel_circ_0002871	mmu-miR-128-2-	ENSMUST00000213072	ENSMUSG00000031849
novel_circ_0002673	mmu-miR-128-2-	ENSMUST00000213072	ENSMUSG00000031849
novel_circ_0002673	mmu-miR-128-2-	ENSMUST00000213072	ENSMUSG00000031849
novel_circ_0002675	mmu-miR-128-2-	ENSMUST00000213072	ENSMUSG00000031849
novel_circ_0003036	mmu-miR-128-2-	ENSMUST00000213072	ENSMUSG00000031849
novel_circ_0003456	mmu-miR-128-2-	ENSMUST00000213072	ENSMUSG00000031849
novel_circ_0003456	mmu-miR-128-2-	ENSMUST00000213072	ENSMUSG00000031849
novel_circ_0000301	mmu-miR-128-2-	ENSMUST00000213072	ENSMUSG00000031849
novel_circ_0000474	mmu-miR-128-2-	ENSMUST00000213072	ENSMUSG00000031849
novel_circ_0000527	mmu-miR-128-2-	ENSMUST00000213072	ENSMUSG00000031849
novel_circ_0000594	mmu-miR-128-2-	ENSMUST00000213072	ENSMUSG00000031849
novel_circ_0001213	mmu-miR-128-2-	ENSMUST00000213072	ENSMUSG00000031849
novel_circ_0001214	mmu-miR-128-2-	ENSMUST00000213072	ENSMUSG00000031849
novel_circ_0001257	mmu-miR-215-3p	ENSMUST00000225594	ENSMUSG00000017491
novel_circ_0001270	mmu-miR-215-3p	ENSMUST00000225594	ENSMUSG00000017491
novel_circ_0001119	mmu-miR-215-3p	ENSMUST00000225594	ENSMUSG00000017491
novel_circ_0001449	mmu-miR-215-3p	ENSMUST00000225594	ENSMUSG00000017491
novel_circ_0001473	mmu-miR-215-3p	ENSMUST00000225594	ENSMUSG00000017491
novel_circ_0001337	mmu-miR-215-3p	ENSMUST00000225594	ENSMUSG00000017491
novel_circ_0001380	mmu-miR-215-3p	ENSMUST00000225594	ENSMUSG00000017491
mmu_circ_0000129	mmu-miR-215-3p	ENSMUST00000225594	ENSMUSG00000017491
mmu_circ_0000046	mmu-miR-215-3p	ENSMUST00000225594	ENSMUSG00000017491
mmu_circ_0001123	mmu-miR-215-3p	ENSMUST00000225594	ENSMUSG00000017491
mmu_circ_0001132	mmu-miR-215-3p	ENSMUST00000225594	ENSMUSG00000017491
mmu_circ_0001017	mmu-miR-215-3p	ENSMUST00000225594	ENSMUSG00000017491
mmu_circ_0001021	mmu-miR-215-3p	ENSMUST00000225594	ENSMUSG00000017491
mmu_circ_0001045	mmu-miR-215-3p	ENSMUST00000225594	ENSMUSG00000017491
mmu_circ_0001376	mmu-miR-215-3p	ENSMUST00000225594	ENSMUSG00000017491
mmu_circ_0001431	mmu-miR-215-3p	ENSMUST00000225594	ENSMUSG00000017491
mmu_circ_0001261	mmu-miR-215-3p	ENSMUST00000225594	ENSMUSG00000017491
mmu_circ_0001295	mmu-miR-215-3p	ENSMUST00000225594	ENSMUSG00000017491
mmu_circ_0001634	mmu-miR-215-3p	ENSMUST00000225594	ENSMUSG00000017491
mmu_circ_0001645	mmu-miR-215-3p	ENSMUST00000225594	ENSMUSG00000017491
mmu_circ_0001528	mmu-miR-215-3p	ENSMUST00000225594	ENSMUSG00000017491
mmu_circ_0001454	mmu-miR-215-3p	ENSMUST00000225594	ENSMUSG00000017491
mmu_circ_0001457	mmu-miR-215-3p	ENSMUST00000225594	ENSMUSG00000017491
mmu_circ_0001436	mmu-miR-215-3p	ENSMUST00000225594	ENSMUSG00000017491
mmu_circ_0001500	mmu-miR-215-3p	ENSMUST00000225594	ENSMUSG00000017491
mmu_circ_0001768	mmu-miR-215-3p	ENSMUST00000225594	ENSMUSG00000017491
mmu_circ_0001768	mmu-miR-215-3p	ENSMUST00000225594	ENSMUSG00000017491
mmu_circ_0001770	mmu-miR-215-3p	ENSMUST00000225594	ENSMUSG00000017491
mmu_circ_0001779	mmu-miR-215-3p	ENSMUST00000225594	ENSMUSG00000017491
mmu_circ_0001825	mmu-miR-215-3p	ENSMUST00000225594	ENSMUSG00000017491
mmu_circ_0001692	mmu-miR-215-3p	ENSMUST00000225594	ENSMUSG00000017491

mmu_circ_0001897	mmu-miR-215-3p	ENSMUST00000225594	ENSMUSG00000017491
mmu_circ_0000277	mmu-miR-215-3p	ENSMUST00000225594	ENSMUSG00000017491
mmu_circ_0000277	mmu-miR-215-3p	ENSMUST00000225594	ENSMUSG00000017491
mmu_circ_0000317	mmu-miR-215-3p	ENSMUST00000225594	ENSMUSG00000017491
mmu_circ_0000499	mmu-miR-215-3p	ENSMUST00000225594	ENSMUSG00000017491
mmu_circ_0000500	mmu-miR-215-3p	ENSMUST00000225594	ENSMUSG00000017491
mmu_circ_0000454	mmu-miR-215-3p	ENSMUST00000225594	ENSMUSG00000017491
mmu_circ_0000465	mmu-miR-215-3p	ENSMUST00000225594	ENSMUSG00000017491
mmu_circ_0000491	mmu-miR-215-3p	ENSMUST00000225594	ENSMUSG00000017491
mmu_circ_0000369	mmu-miR-215-3p	ENSMUST00000225594	ENSMUSG00000017491
mmu_circ_0000371	mmu-miR-215-3p	ENSMUST00000225594	ENSMUSG00000017491
mmu_circ_0000372	mmu-miR-215-3p	ENSMUST00000225594	ENSMUSG00000017491
mmu_circ_0000628	mmu-miR-215-3p	ENSMUST00000225594	ENSMUSG00000017491
mmu_circ_0000526	mmu-miR-215-3p	ENSMUST00000225594	ENSMUSG00000017491
mmu_circ_0000530	mmu-miR-215-3p	ENSMUST00000225594	ENSMUSG00000017491
mmu_circ_0000735	mmu-miR-215-3p	ENSMUST00000225594	ENSMUSG00000017491
mmu_circ_0000819	mmu-miR-215-3p	ENSMUST00000225594	ENSMUSG00000017491
mmu_circ_0000820	mmu-miR-215-3p	ENSMUST00000225594	ENSMUSG00000017491
mmu_circ_0000828	mmu-miR-215-3p	ENSMUST00000225594	ENSMUSG00000017491
mmu_circ_0000829	mmu-miR-215-3p	ENSMUST00000225594	ENSMUSG00000017491
mmu_circ_0000831	mmu-miR-215-3p	ENSMUST00000225594	ENSMUSG00000017491
mmu_circ_0000714	mmu-miR-215-3p	ENSMUST00000225594	ENSMUSG00000017491
mmu_circ_0000918	mmu-miR-215-3p	ENSMUST00000225594	ENSMUSG00000017491
mmu_circ_0000919	mmu-miR-215-3p	ENSMUST00000225594	ENSMUSG00000017491
mmu_circ_0000921	mmu-miR-215-3p	ENSMUST00000225594	ENSMUSG00000017491
mmu_circ_0000884	mmu-miR-215-3p	ENSMUST00000225594	ENSMUSG00000017491
mmu_circ_0000886	mmu-miR-215-3p	ENSMUST00000225594	ENSMUSG00000017491
mmu_circ_0000887	mmu-miR-215-3p	ENSMUST00000225594	ENSMUSG00000017491
mmu_circ_0000905	mmu-miR-215-3p	ENSMUST00000225594	ENSMUSG00000017491
mmu_circ_0000906	mmu-miR-215-3p	ENSMUST00000225594	ENSMUSG00000017491
mmu_circ_0000907	mmu-miR-215-3p	ENSMUST00000225594	ENSMUSG00000017491
mmu_circ_0000912	mmu-miR-215-3p	ENSMUST00000225594	ENSMUSG00000017491
novel_circ_0001561	mmu-miR-215-3p	ENSMUST00000225594	ENSMUSG00000017491
novel_circ_0001597	mmu-miR-215-3p	ENSMUST00000225594	ENSMUSG00000017491
novel_circ_0002123	mmu-miR-215-3p	ENSMUST00000225594	ENSMUSG00000017491
novel_circ_0002175	mmu-miR-215-3p	ENSMUST00000225594	ENSMUSG00000017491
novel_circ_0001874	mmu-miR-215-3p	ENSMUST00000225594	ENSMUSG00000017491
novel_circ_0001878	mmu-miR-215-3p	ENSMUST00000225594	ENSMUSG00000017491
novel_circ_0001880	mmu-miR-215-3p	ENSMUST00000225594	ENSMUSG00000017491
novel_circ_0001910	mmu-miR-215-3p	ENSMUST00000225594	ENSMUSG00000017491
novel_circ_0002031	mmu-miR-215-3p	ENSMUST00000225594	ENSMUSG00000017491
novel_circ_0002051	mmu-miR-215-3p	ENSMUST00000225594	ENSMUSG00000017491
novel_circ_0002412	mmu-miR-215-3p	ENSMUST00000225594	ENSMUSG00000017491
novel_circ_0002417	mmu-miR-215-3p	ENSMUST00000225594	ENSMUSG00000017491
novel_circ_0002482	mmu-miR-215-3p	ENSMUST00000225594	ENSMUSG00000017491
novel_circ_0002482	mmu-miR-215-3p	ENSMUST00000225594	ENSMUSG00000017491
novel_circ_0002486	mmu-miR-215-3p	ENSMUST00000225594	ENSMUSG00000017491
novel_circ_0002486	mmu-miR-215-3p	ENSMUST00000225594	ENSMUSG00000017491
novel_circ_0002499	mmu-miR-215-3p	ENSMUST00000225594	ENSMUSG00000017491
novel_circ_0002289	mmu-miR-215-3p	ENSMUST00000225594	ENSMUSG00000017491
novel_circ_0002290	mmu-miR-215-3p	ENSMUST00000225594	ENSMUSG00000017491
novel_circ_0002831	mmu-miR-215-3p	ENSMUST00000225594	ENSMUSG00000017491
novel_circ_0002872	mmu-miR-215-3p	ENSMUST00000225594	ENSMUSG00000017491
novel_circ_0002872	mmu-miR-215-3p	ENSMUST00000225594	ENSMUSG00000017491
novel_circ_0002873	mmu-miR-215-3p	ENSMUST00000225594	ENSMUSG00000017491
novel_circ_0002874	mmu-miR-215-3p	ENSMUST00000225594	ENSMUSG00000017491
novel_circ_0002875	mmu-miR-215-3p	ENSMUST00000225594	ENSMUSG00000017491
novel_circ_0002895	mmu-miR-215-3p	ENSMUST00000225594	ENSMUSG00000017491

novel_circ_0002895	mmu-miR-215-3p	ENSMUST00000225594	ENSMUSG00000017491
novel_circ_0002917	mmu-miR-215-3p	ENSMUST00000225594	ENSMUSG00000017491
novel_circ_0002925	mmu-miR-215-3p	ENSMUST00000225594	ENSMUSG00000017491
novel_circ_0002961	mmu-miR-215-3p	ENSMUST00000225594	ENSMUSG00000017491
novel_circ_0002649	mmu-miR-215-3p	ENSMUST00000225594	ENSMUSG00000017491
novel_circ_0002649	mmu-miR-215-3p	ENSMUST00000225594	ENSMUSG00000017491
novel_circ_0002675	mmu-miR-215-3p	ENSMUST00000225594	ENSMUSG00000017491
novel_circ_0002782	mmu-miR-215-3p	ENSMUST00000225594	ENSMUSG00000017491
novel_circ_0002782	mmu-miR-215-3p	ENSMUST00000225594	ENSMUSG00000017491
novel_circ_0003171	mmu-miR-215-3p	ENSMUST00000225594	ENSMUSG00000017491
novel_circ_0003172	mmu-miR-215-3p	ENSMUST00000225594	ENSMUSG00000017491
novel_circ_0003208	mmu-miR-215-3p	ENSMUST00000225594	ENSMUSG00000017491
novel_circ_0003397	mmu-miR-215-3p	ENSMUST00000225594	ENSMUSG00000017491
novel_circ_0003397	mmu-miR-215-3p	ENSMUST00000225594	ENSMUSG00000017491
novel_circ_0003112	mmu-miR-215-3p	ENSMUST00000225594	ENSMUSG00000017491
novel_circ_0003113	mmu-miR-215-3p	ENSMUST00000225594	ENSMUSG00000017491
novel_circ_0003156	mmu-miR-215-3p	ENSMUST00000225594	ENSMUSG00000017491
novel_circ_0003491	mmu-miR-215-3p	ENSMUST00000225594	ENSMUSG00000017491
novel_circ_0000182	mmu-miR-215-3p	ENSMUST00000225594	ENSMUSG00000017491
novel_circ_0000205	mmu-miR-215-3p	ENSMUST00000225594	ENSMUSG00000017491
novel_circ_0000205	mmu-miR-215-3p	ENSMUST00000225594	ENSMUSG00000017491
novel_circ_0000389	mmu-miR-215-3p	ENSMUST00000225594	ENSMUSG00000017491
novel_circ_0000389	mmu-miR-215-3p	ENSMUST00000225594	ENSMUSG00000017491
novel_circ_0000452	mmu-miR-215-3p	ENSMUST00000225594	ENSMUSG00000017491
novel_circ_0000011	mmu-miR-215-3p	ENSMUST00000225594	ENSMUSG00000017491
novel_circ_0000087	mmu-miR-215-3p	ENSMUST00000225594	ENSMUSG00000017491
novel_circ_0000087	mmu-miR-215-3p	ENSMUST00000225594	ENSMUSG00000017491
novel_circ_0000088	mmu-miR-215-3p	ENSMUST00000225594	ENSMUSG00000017491
novel_circ_0000148	mmu-miR-215-3p	ENSMUST00000225594	ENSMUSG00000017491
novel_circ_0000624	mmu-miR-215-3p	ENSMUST00000225594	ENSMUSG00000017491
novel_circ_0000668	mmu-miR-215-3p	ENSMUST00000225594	ENSMUSG00000017491
novel_circ_0000743	mmu-miR-215-3p	ENSMUST00000225594	ENSMUSG00000017491
novel_circ_0000748	mmu-miR-215-3p	ENSMUST00000225594	ENSMUSG00000017491
novel_circ_0000769	mmu-miR-215-3p	ENSMUST00000225594	ENSMUSG00000017491
novel_circ_0000769	mmu-miR-215-3p	ENSMUST00000225594	ENSMUSG00000017491
novel_circ_0000789	mmu-miR-215-3p	ENSMUST00000225594	ENSMUSG00000017491
novel_circ_0000789	mmu-miR-215-3p	ENSMUST00000225594	ENSMUSG00000017491
novel_circ_0000467	mmu-miR-215-3p	ENSMUST00000225594	ENSMUSG00000017491
novel_circ_0000475	mmu-miR-215-3p	ENSMUST00000225594	ENSMUSG00000017491
novel_circ_0000608	mmu-miR-215-3p	ENSMUST00000225594	ENSMUSG00000017491
novel_circ_0001024	mmu-miR-215-3p	ENSMUST00000225594	ENSMUSG00000017491
novel_circ_0000852	mmu-miR-215-3p	ENSMUST00000225594	ENSMUSG00000017491
novel_circ_0000876	mmu-miR-215-3p	ENSMUST00000225594	ENSMUSG00000017491
novel_circ_0000888	mmu-miR-215-3p	ENSMUST00000225594	ENSMUSG00000017491
novel_circ_0001177	mmu-miR-215-3p	ENSMUST00000225594	ENSMUSG00000017491
novel_circ_0001179	mmu-miR-215-3p	ENSMUST00000225594	ENSMUSG00000017491
novel_circ_0001230	mmu-miR-483-3p	ENSMUST00000000128	ENSMUSG00000000126
novel_circ_0001230	mmu-miR-483-3p	ENSMUST00000108783	ENSMUSG00000000126
novel_circ_0001241	mmu-miR-483-3p	ENSMUST00000000128	ENSMUSG00000000126
novel_circ_0001241	mmu-miR-483-3p	ENSMUST00000108783	ENSMUSG00000000126
novel_circ_0001241	mmu-miR-483-3p	ENSMUST00000000128	ENSMUSG00000000126
novel_circ_0001241	mmu-miR-483-3p	ENSMUST00000108783	ENSMUSG00000000126
novel_circ_0001086	mmu-miR-483-3p	ENSMUST00000000128	ENSMUSG00000000126
novel_circ_0001086	mmu-miR-483-3p	ENSMUST00000108783	ENSMUSG00000000126
novel_circ_0001534	mmu-miR-483-3p	ENSMUST00000000128	ENSMUSG00000000126
novel_circ_0001534	mmu-miR-483-3p	ENSMUST00000108783	ENSMUSG00000000126
novel_circ_0001318	mmu-miR-483-3p	ENSMUST00000000128	ENSMUSG00000000126
novel_circ_0001318	mmu-miR-483-3p	ENSMUST00000108783	ENSMUSG00000000126

novel_circ_0001324	mmu-miR-483-3p	ENSMUST00000000128	ENSMUSG00000000126
novel_circ_0001324	mmu-miR-483-3p	ENSMUST00000108783	ENSMUSG00000000126
novel_circ_0001340	mmu-miR-483-3p	ENSMUST00000000128	ENSMUSG00000000126
novel_circ_0001340	mmu-miR-483-3p	ENSMUST00000108783	ENSMUSG00000000126
novel_circ_0001340	mmu-miR-483-3p	ENSMUST00000000128	ENSMUSG00000000126
novel_circ_0001340	mmu-miR-483-3p	ENSMUST00000108783	ENSMUSG00000000126
novel_circ_0001398	mmu-miR-483-3p	ENSMUST00000000128	ENSMUSG00000000126
novel_circ_0001398	mmu-miR-483-3p	ENSMUST00000108783	ENSMUSG00000000126
novel_circ_0001399	mmu-miR-483-3p	ENSMUST00000000128	ENSMUSG00000000126
novel_circ_0001399	mmu-miR-483-3p	ENSMUST00000108783	ENSMUSG00000000126
novel_circ_0001400	mmu-miR-483-3p	ENSMUST00000000128	ENSMUSG00000000126
novel_circ_0001400	mmu-miR-483-3p	ENSMUST00000108783	ENSMUSG00000000126
mmu_circ_0000075	mmu-miR-483-3p	ENSMUST00000000128	ENSMUSG00000000126
mmu_circ_0000075	mmu-miR-483-3p	ENSMUST00000108783	ENSMUSG00000000126
mmu_circ_0000087	mmu-miR-483-3p	ENSMUST00000000128	ENSMUSG00000000126
mmu_circ_0000087	mmu-miR-483-3p	ENSMUST00000108783	ENSMUSG00000000126
mmu_circ_0000048	mmu-miR-483-3p	ENSMUST00000000128	ENSMUSG00000000126
mmu_circ_0000048	mmu-miR-483-3p	ENSMUST00000108783	ENSMUSG00000000126
mmu_circ_0000063	mmu-miR-483-3p	ENSMUST00000000128	ENSMUSG00000000126
mmu_circ_0000063	mmu-miR-483-3p	ENSMUST00000108783	ENSMUSG00000000126
mmu_circ_0001132	mmu-miR-483-3p	ENSMUST00000000128	ENSMUSG00000000126
mmu_circ_0001132	mmu-miR-483-3p	ENSMUST00000108783	ENSMUSG00000000126
mmu_circ_0001017	mmu-miR-483-3p	ENSMUST00000000128	ENSMUSG00000000126
mmu_circ_0001017	mmu-miR-483-3p	ENSMUST00000108783	ENSMUSG00000000126
mmu_circ_0001031	mmu-miR-483-3p	ENSMUST00000000128	ENSMUSG00000000126
mmu_circ_0001031	mmu-miR-483-3p	ENSMUST00000108783	ENSMUSG00000000126
mmu_circ_0001049	mmu-miR-483-3p	ENSMUST00000000128	ENSMUSG00000000126
mmu_circ_0001049	mmu-miR-483-3p	ENSMUST00000108783	ENSMUSG00000000126
mmu_circ_0001376	mmu-miR-483-3p	ENSMUST00000000128	ENSMUSG00000000126
mmu_circ_0001376	mmu-miR-483-3p	ENSMUST00000108783	ENSMUSG00000000126
mmu_circ_0001345	mmu-miR-483-3p	ENSMUST00000000128	ENSMUSG00000000126
mmu_circ_0001345	mmu-miR-483-3p	ENSMUST00000108783	ENSMUSG00000000126
mmu_circ_0001364	mmu-miR-483-3p	ENSMUST00000000128	ENSMUSG00000000126
mmu_circ_0001364	mmu-miR-483-3p	ENSMUST00000108783	ENSMUSG00000000126
mmu_circ_0001255	mmu-miR-483-3p	ENSMUST00000000128	ENSMUSG00000000126
mmu_circ_0001255	mmu-miR-483-3p	ENSMUST00000108783	ENSMUSG00000000126
mmu_circ_0001256	mmu-miR-483-3p	ENSMUST00000000128	ENSMUSG00000000126
mmu_circ_0001256	mmu-miR-483-3p	ENSMUST00000108783	ENSMUSG00000000126
mmu_circ_0001279	mmu-miR-483-3p	ENSMUST00000000128	ENSMUSG00000000126
mmu_circ_0001279	mmu-miR-483-3p	ENSMUST00000108783	ENSMUSG00000000126
mmu_circ_0001620	mmu-miR-483-3p	ENSMUST00000000128	ENSMUSG00000000126
mmu_circ_0001620	mmu-miR-483-3p	ENSMUST00000108783	ENSMUSG00000000126
mmu_circ_0001623	mmu-miR-483-3p	ENSMUST00000000128	ENSMUSG00000000126
mmu_circ_0001623	mmu-miR-483-3p	ENSMUST00000108783	ENSMUSG00000000126
mmu_circ_0001569	mmu-miR-483-3p	ENSMUST00000000128	ENSMUSG00000000126
mmu_circ_0001569	mmu-miR-483-3p	ENSMUST00000108783	ENSMUSG00000000126
mmu_circ_0001569	mmu-miR-483-3p	ENSMUST00000000128	ENSMUSG00000000126
mmu_circ_0001569	mmu-miR-483-3p	ENSMUST00000108783	ENSMUSG00000000126
mmu_circ_0001589	mmu-miR-483-3p	ENSMUST00000000128	ENSMUSG00000000126
mmu_circ_0001589	mmu-miR-483-3p	ENSMUST00000108783	ENSMUSG00000000126
mmu_circ_0001500	mmu-miR-483-3p	ENSMUST00000000128	ENSMUSG00000000126
mmu_circ_0001500	mmu-miR-483-3p	ENSMUST00000108783	ENSMUSG00000000126
mmu_circ_0001795	mmu-miR-483-3p	ENSMUST00000000128	ENSMUSG00000000126
mmu_circ_0001795	mmu-miR-483-3p	ENSMUST00000108783	ENSMUSG00000000126
mmu_circ_0001664	mmu-miR-483-3p	ENSMUST00000000128	ENSMUSG00000000126
mmu_circ_0001664	mmu-miR-483-3p	ENSMUST00000108783	ENSMUSG00000000126
mmu_circ_0001665	mmu-miR-483-3p	ENSMUST00000000128	ENSMUSG00000000126
mmu_circ_0001665	mmu-miR-483-3p	ENSMUST00000108783	ENSMUSG00000000126

mmu_circ_0000172	mmu-miR-483-3p	ENSMUST00000000128	ENSMUSG00000000126
mmu_circ_0000172	mmu-miR-483-3p	ENSMUST00000108783	ENSMUSG00000000126
mmu_circ_0000499	mmu-miR-483-3p	ENSMUST00000000128	ENSMUSG00000000126
mmu_circ_0000499	mmu-miR-483-3p	ENSMUST00000108783	ENSMUSG00000000126
mmu_circ_0000500	mmu-miR-483-3p	ENSMUST00000000128	ENSMUSG00000000126
mmu_circ_0000500	mmu-miR-483-3p	ENSMUST00000108783	ENSMUSG00000000126
mmu_circ_0000454	mmu-miR-483-3p	ENSMUST00000000128	ENSMUSG00000000126
mmu_circ_0000454	mmu-miR-483-3p	ENSMUST00000108783	ENSMUSG00000000126
mmu_circ_0000413	mmu-miR-483-3p	ENSMUST00000000128	ENSMUSG00000000126
mmu_circ_0000413	mmu-miR-483-3p	ENSMUST00000108783	ENSMUSG00000000126
mmu_circ_0000423	mmu-miR-483-3p	ENSMUST00000000128	ENSMUSG00000000126
mmu_circ_0000423	mmu-miR-483-3p	ENSMUST00000108783	ENSMUSG00000000126
mmu_circ_0000362	mmu-miR-483-3p	ENSMUST00000000128	ENSMUSG00000000126
mmu_circ_0000362	mmu-miR-483-3p	ENSMUST00000108783	ENSMUSG00000000126
mmu_circ_0000362	mmu-miR-483-3p	ENSMUST00000000128	ENSMUSG00000000126
mmu_circ_0000362	mmu-miR-483-3p	ENSMUST00000108783	ENSMUSG00000000126
mmu_circ_0000399	mmu-miR-483-3p	ENSMUST00000000128	ENSMUSG00000000126
mmu_circ_0000399	mmu-miR-483-3p	ENSMUST00000108783	ENSMUSG00000000126
mmu_circ_0000628	mmu-miR-483-3p	ENSMUST00000000128	ENSMUSG00000000126
mmu_circ_0000628	mmu-miR-483-3p	ENSMUST00000108783	ENSMUSG00000000126
mmu_circ_0000635	mmu-miR-483-3p	ENSMUST00000000128	ENSMUSG00000000126
mmu_circ_0000635	mmu-miR-483-3p	ENSMUST00000108783	ENSMUSG00000000126
mmu_circ_0000526	mmu-miR-483-3p	ENSMUST00000000128	ENSMUSG00000000126
mmu_circ_0000526	mmu-miR-483-3p	ENSMUST00000108783	ENSMUSG00000000126
mmu_circ_0000530	mmu-miR-483-3p	ENSMUST00000000128	ENSMUSG00000000126
mmu_circ_0000530	mmu-miR-483-3p	ENSMUST00000108783	ENSMUSG00000000126
mmu_circ_0000829	mmu-miR-483-3p	ENSMUST00000000128	ENSMUSG00000000126
mmu_circ_0000829	mmu-miR-483-3p	ENSMUST00000108783	ENSMUSG00000000126
mmu_circ_0000831	mmu-miR-483-3p	ENSMUST00000000128	ENSMUSG00000000126
mmu_circ_0000831	mmu-miR-483-3p	ENSMUST00000108783	ENSMUSG00000000126
mmu_circ_0000711	mmu-miR-483-3p	ENSMUST00000000128	ENSMUSG00000000126
mmu_circ_0000711	mmu-miR-483-3p	ENSMUST00000108783	ENSMUSG00000000126
mmu_circ_0000969	mmu-miR-483-3p	ENSMUST00000000128	ENSMUSG00000000126
mmu_circ_0000969	mmu-miR-483-3p	ENSMUST00000108783	ENSMUSG00000000126
mmu_circ_0000884	mmu-miR-483-3p	ENSMUST00000000128	ENSMUSG00000000126
mmu_circ_0000884	mmu-miR-483-3p	ENSMUST00000108783	ENSMUSG00000000126
mmu_circ_0000886	mmu-miR-483-3p	ENSMUST00000000128	ENSMUSG00000000126
mmu_circ_0000886	mmu-miR-483-3p	ENSMUST00000108783	ENSMUSG00000000126
mmu_circ_0000905	mmu-miR-483-3p	ENSMUST00000000128	ENSMUSG00000000126
mmu_circ_0000905	mmu-miR-483-3p	ENSMUST00000108783	ENSMUSG00000000126
mmu_circ_0000906	mmu-miR-483-3p	ENSMUST00000000128	ENSMUSG00000000126
mmu_circ_0000906	mmu-miR-483-3p	ENSMUST00000108783	ENSMUSG00000000126
mmu_circ_0000907	mmu-miR-483-3p	ENSMUST00000000128	ENSMUSG00000000126
mmu_circ_0000907	mmu-miR-483-3p	ENSMUST00000108783	ENSMUSG00000000126
mmu_circ_0000908	mmu-miR-483-3p	ENSMUST00000000128	ENSMUSG00000000126
mmu_circ_0000908	mmu-miR-483-3p	ENSMUST00000108783	ENSMUSG00000000126
mmu_circ_0000912	mmu-miR-483-3p	ENSMUST00000000128	ENSMUSG00000000126
mmu_circ_0000912	mmu-miR-483-3p	ENSMUST00000108783	ENSMUSG00000000126
novel_circ_0001662	mmu-miR-483-3p	ENSMUST00000000128	ENSMUSG00000000126
novel_circ_0001662	mmu-miR-483-3p	ENSMUST00000108783	ENSMUSG00000000126
novel_circ_0001663	mmu-miR-483-3p	ENSMUST00000000128	ENSMUSG00000000126
novel_circ_0001663	mmu-miR-483-3p	ENSMUST00000108783	ENSMUSG00000000126
novel_circ_0001676	mmu-miR-483-3p	ENSMUST00000000128	ENSMUSG00000000126
novel_circ_0001676	mmu-miR-483-3p	ENSMUST00000108783	ENSMUSG00000000126
novel_circ_0001680	mmu-miR-483-3p	ENSMUST00000000128	ENSMUSG00000000126
novel_circ_0001680	mmu-miR-483-3p	ENSMUST00000108783	ENSMUSG00000000126
novel_circ_0001710	mmu-miR-483-3p	ENSMUST00000000128	ENSMUSG00000000126
novel_circ_0001710	mmu-miR-483-3p	ENSMUST00000108783	ENSMUSG00000000126

novel_circ_0001728	mmu-miR-483-3p	ENSMUST00000000128	ENSMUSG00000000126
novel_circ_0001728	mmu-miR-483-3p	ENSMUST00000108783	ENSMUSG00000000126
novel_circ_0002203	mmu-miR-483-3p	ENSMUST00000000128	ENSMUSG00000000126
novel_circ_0002203	mmu-miR-483-3p	ENSMUST00000108783	ENSMUSG00000000126
novel_circ_0001773	mmu-miR-483-3p	ENSMUST00000000128	ENSMUSG00000000126
novel_circ_0001773	mmu-miR-483-3p	ENSMUST00000108783	ENSMUSG00000000126
novel_circ_0001774	mmu-miR-483-3p	ENSMUST00000000128	ENSMUSG00000000126
novel_circ_0001774	mmu-miR-483-3p	ENSMUST00000108783	ENSMUSG00000000126
novel_circ_0001835	mmu-miR-483-3p	ENSMUST00000000128	ENSMUSG00000000126
novel_circ_0001835	mmu-miR-483-3p	ENSMUST00000108783	ENSMUSG00000000126
novel_circ_0001842	mmu-miR-483-3p	ENSMUST00000000128	ENSMUSG00000000126
novel_circ_0001842	mmu-miR-483-3p	ENSMUST00000108783	ENSMUSG00000000126
novel_circ_0002417	mmu-miR-483-3p	ENSMUST00000000128	ENSMUSG00000000126
novel_circ_0002417	mmu-miR-483-3p	ENSMUST00000108783	ENSMUSG00000000126
novel_circ_0002515	mmu-miR-483-3p	ENSMUST00000000128	ENSMUSG00000000126
novel_circ_0002515	mmu-miR-483-3p	ENSMUST00000108783	ENSMUSG00000000126
novel_circ_0002530	mmu-miR-483-3p	ENSMUST00000000128	ENSMUSG00000000126
novel_circ_0002530	mmu-miR-483-3p	ENSMUST00000108783	ENSMUSG00000000126
novel_circ_0002609	mmu-miR-483-3p	ENSMUST00000000128	ENSMUSG00000000126
novel_circ_0002609	mmu-miR-483-3p	ENSMUST00000108783	ENSMUSG00000000126
novel_circ_0002257	mmu-miR-483-3p	ENSMUST00000000128	ENSMUSG00000000126
novel_circ_0002257	mmu-miR-483-3p	ENSMUST00000108783	ENSMUSG00000000126
novel_circ_0002290	mmu-miR-483-3p	ENSMUST00000000128	ENSMUSG00000000126
novel_circ_0002290	mmu-miR-483-3p	ENSMUST00000108783	ENSMUSG00000000126
novel_circ_0002327	mmu-miR-483-3p	ENSMUST00000000128	ENSMUSG00000000126
novel_circ_0002327	mmu-miR-483-3p	ENSMUST00000108783	ENSMUSG00000000126
novel_circ_0002849	mmu-miR-483-3p	ENSMUST00000000128	ENSMUSG00000000126
novel_circ_0002849	mmu-miR-483-3p	ENSMUST00000108783	ENSMUSG00000000126
novel_circ_0002868	mmu-miR-483-3p	ENSMUST00000000128	ENSMUSG00000000126
novel_circ_0002868	mmu-miR-483-3p	ENSMUST00000108783	ENSMUSG00000000126
novel_circ_0002884	mmu-miR-483-3p	ENSMUST00000000128	ENSMUSG00000000126
novel_circ_0002884	mmu-miR-483-3p	ENSMUST00000108783	ENSMUSG00000000126
novel_circ_0002926	mmu-miR-483-3p	ENSMUST00000000128	ENSMUSG00000000126
novel_circ_0002926	mmu-miR-483-3p	ENSMUST00000108783	ENSMUSG00000000126
novel_circ_0002930	mmu-miR-483-3p	ENSMUST00000000128	ENSMUSG00000000126
novel_circ_0002930	mmu-miR-483-3p	ENSMUST00000108783	ENSMUSG00000000126
novel_circ_0002936	mmu-miR-483-3p	ENSMUST00000000128	ENSMUSG00000000126
novel_circ_0002936	mmu-miR-483-3p	ENSMUST00000108783	ENSMUSG00000000126
novel_circ_0002944	mmu-miR-483-3p	ENSMUST00000000128	ENSMUSG00000000126
novel_circ_0002944	mmu-miR-483-3p	ENSMUST00000108783	ENSMUSG00000000126
novel_circ_0002950	mmu-miR-483-3p	ENSMUST00000000128	ENSMUSG00000000126
novel_circ_0002950	mmu-miR-483-3p	ENSMUST00000108783	ENSMUSG00000000126
novel_circ_0002656	mmu-miR-483-3p	ENSMUST00000000128	ENSMUSG00000000126
novel_circ_0002656	mmu-miR-483-3p	ENSMUST00000108783	ENSMUSG00000000126
novel_circ_0002658	mmu-miR-483-3p	ENSMUST00000000128	ENSMUSG00000000126
novel_circ_0002658	mmu-miR-483-3p	ENSMUST00000108783	ENSMUSG00000000126
novel_circ_0003195	mmu-miR-483-3p	ENSMUST00000000128	ENSMUSG00000000126
novel_circ_0003195	mmu-miR-483-3p	ENSMUST00000108783	ENSMUSG00000000126
novel_circ_0003225	mmu-miR-483-3p	ENSMUST00000000128	ENSMUSG00000000126
novel_circ_0003225	mmu-miR-483-3p	ENSMUST00000108783	ENSMUSG00000000126
novel_circ_0003053	mmu-miR-483-3p	ENSMUST00000000128	ENSMUSG00000000126
novel_circ_0003053	mmu-miR-483-3p	ENSMUST00000108783	ENSMUSG00000000126
novel_circ_0003434	mmu-miR-483-3p	ENSMUST00000000128	ENSMUSG00000000126
novel_circ_0003434	mmu-miR-483-3p	ENSMUST00000108783	ENSMUSG00000000126
novel_circ_0003456	mmu-miR-483-3p	ENSMUST00000000128	ENSMUSG00000000126
novel_circ_0003456	mmu-miR-483-3p	ENSMUST00000108783	ENSMUSG00000000126
novel_circ_0000319	mmu-miR-483-3p	ENSMUST00000000128	ENSMUSG00000000126
novel_circ_0000319	mmu-miR-483-3p	ENSMUST00000108783	ENSMUSG00000000126

novel_circ_0000324	mmu-miR-483-3p	ENSMUST00000000128	ENSMUSG00000000126
novel_circ_0000324	mmu-miR-483-3p	ENSMUST00000108783	ENSMUSG00000000126
novel_circ_0000084	mmu-miR-483-3p	ENSMUST00000000128	ENSMUSG00000000126
novel_circ_0000084	mmu-miR-483-3p	ENSMUST00000108783	ENSMUSG00000000126
novel_circ_0000108	mmu-miR-483-3p	ENSMUST00000000128	ENSMUSG00000000126
novel_circ_0000108	mmu-miR-483-3p	ENSMUST00000108783	ENSMUSG00000000126
novel_circ_0000134	mmu-miR-483-3p	ENSMUST00000000128	ENSMUSG00000000126
novel_circ_0000134	mmu-miR-483-3p	ENSMUST00000108783	ENSMUSG00000000126
novel_circ_0000692	mmu-miR-483-3p	ENSMUST00000000128	ENSMUSG00000000126
novel_circ_0000692	mmu-miR-483-3p	ENSMUST00000108783	ENSMUSG00000000126
novel_circ_0000733	mmu-miR-483-3p	ENSMUST00000000128	ENSMUSG00000000126
novel_circ_0000733	mmu-miR-483-3p	ENSMUST00000108783	ENSMUSG00000000126
novel_circ_0000515	mmu-miR-483-3p	ENSMUST00000000128	ENSMUSG00000000126
novel_circ_0000515	mmu-miR-483-3p	ENSMUST00000108783	ENSMUSG00000000126
novel_circ_0000516	mmu-miR-483-3p	ENSMUST00000000128	ENSMUSG00000000126
novel_circ_0000516	mmu-miR-483-3p	ENSMUST00000108783	ENSMUSG00000000126
novel_circ_0000584	mmu-miR-483-3p	ENSMUST00000000128	ENSMUSG00000000126
novel_circ_0000584	mmu-miR-483-3p	ENSMUST00000108783	ENSMUSG00000000126
novel_circ_0000958	mmu-miR-483-3p	ENSMUST00000000128	ENSMUSG00000000126
novel_circ_0000958	mmu-miR-483-3p	ENSMUST00000108783	ENSMUSG00000000126
novel_circ_0000995	mmu-miR-483-3p	ENSMUST00000000128	ENSMUSG00000000126
novel_circ_0000995	mmu-miR-483-3p	ENSMUST00000108783	ENSMUSG00000000126
novel_circ_0001029	mmu-miR-483-3p	ENSMUST00000000128	ENSMUSG00000000126
novel_circ_0001029	mmu-miR-483-3p	ENSMUST00000108783	ENSMUSG00000000126
novel_circ_0001033	mmu-miR-483-3p	ENSMUST00000000128	ENSMUSG00000000126
novel_circ_0001033	mmu-miR-483-3p	ENSMUST00000108783	ENSMUSG00000000126
novel_circ_0000819	mmu-miR-483-3p	ENSMUST00000000128	ENSMUSG00000000126
novel_circ_0000819	mmu-miR-483-3p	ENSMUST00000108783	ENSMUSG00000000126
novel_circ_0001188	mmu-miR-483-3p	ENSMUST00000000128	ENSMUSG00000000126
novel_circ_0001188	mmu-miR-483-3p	ENSMUST00000108783	ENSMUSG00000000126
novel_circ_0001189	mmu-miR-483-3p	ENSMUST00000000128	ENSMUSG00000000126
novel_circ_0001189	mmu-miR-483-3p	ENSMUST00000108783	ENSMUSG00000000126
novel_circ_0001194	mmu-miR-483-3p	ENSMUST00000000128	ENSMUSG00000000126
novel_circ_0001194	mmu-miR-483-3p	ENSMUST00000108783	ENSMUSG00000000126
novel_circ_0001198	mmu-miR-483-3p	ENSMUST00000000128	ENSMUSG00000000126
novel_circ_0001198	mmu-miR-483-3p	ENSMUST00000108783	ENSMUSG00000000126
novel_circ_0001136	mmu-miR-486a-3	ENSMUST00000000128	ENSMUSG00000000126
novel_circ_0001136	mmu-miR-486a-3	ENSMUST00000108783	ENSMUSG00000000126
novel_circ_0001136	mmu-miR-486a-3	ENSMUST00000213072	ENSMUSG00000031849
novel_circ_0001471	mmu-miR-486a-3	ENSMUST00000000128	ENSMUSG00000000126
novel_circ_0001471	mmu-miR-486a-3	ENSMUST00000108783	ENSMUSG00000000126
novel_circ_0001471	mmu-miR-486a-3	ENSMUST00000213072	ENSMUSG00000031849
novel_circ_0001495	mmu-miR-486a-3	ENSMUST00000000128	ENSMUSG00000000126
novel_circ_0001495	mmu-miR-486a-3	ENSMUST00000108783	ENSMUSG00000000126
novel_circ_0001495	mmu-miR-486a-3	ENSMUST00000213072	ENSMUSG00000031849
novel_circ_0001388	mmu-miR-486a-3	ENSMUST00000000128	ENSMUSG00000000126
novel_circ_0001388	mmu-miR-486a-3	ENSMUST00000108783	ENSMUSG00000000126
novel_circ_0001388	mmu-miR-486a-3	ENSMUST00000213072	ENSMUSG00000031849
novel_circ_0001414	mmu-miR-486a-3	ENSMUST00000000128	ENSMUSG00000000126
novel_circ_0001414	mmu-miR-486a-3	ENSMUST00000108783	ENSMUSG00000000126
novel_circ_0001414	mmu-miR-486a-3	ENSMUST00000213072	ENSMUSG00000031849
novel_circ_0001418	mmu-miR-486a-3	ENSMUST00000000128	ENSMUSG00000000126
novel_circ_0001418	mmu-miR-486a-3	ENSMUST00000108783	ENSMUSG00000000126
novel_circ_0001418	mmu-miR-486a-3	ENSMUST00000213072	ENSMUSG00000031849
mmu_circ_0000040	mmu-miR-486a-3	ENSMUST00000000128	ENSMUSG00000000126
mmu_circ_0000040	mmu-miR-486a-3	ENSMUST00000108783	ENSMUSG00000000126
mmu_circ_0000040	mmu-miR-486a-3	ENSMUST00000213072	ENSMUSG00000031849
mmu_circ_0000041	mmu-miR-486a-3	ENSMUST00000000128	ENSMUSG00000000126

mmu_circ_0000041	mmu-miR-486a-3	ENSMUST00000108783	ENSMUSG00000000126
mmu_circ_0000041	mmu-miR-486a-3	ENSMUST00000213072	ENSMUSG00000031849
mmu_circ_0000063	mmu-miR-486a-3	ENSMUST00000000128	ENSMUSG00000000126
mmu_circ_0000063	mmu-miR-486a-3	ENSMUST00000108783	ENSMUSG00000000126
mmu_circ_0000063	mmu-miR-486a-3	ENSMUST00000213072	ENSMUSG00000031849
mmu_circ_0001376	mmu-miR-486a-3	ENSMUST00000000128	ENSMUSG00000000126
mmu_circ_0001376	mmu-miR-486a-3	ENSMUST00000108783	ENSMUSG00000000126
mmu_circ_0001376	mmu-miR-486a-3	ENSMUST00000213072	ENSMUSG00000031849
mmu_circ_0001331	mmu-miR-486a-3	ENSMUST00000000128	ENSMUSG00000000126
mmu_circ_0001331	mmu-miR-486a-3	ENSMUST00000108783	ENSMUSG00000000126
mmu_circ_0001331	mmu-miR-486a-3	ENSMUST00000213072	ENSMUSG00000031849
mmu_circ_0001333	mmu-miR-486a-3	ENSMUST00000000128	ENSMUSG00000000126
mmu_circ_0001333	mmu-miR-486a-3	ENSMUST00000108783	ENSMUSG00000000126
mmu_circ_0001333	mmu-miR-486a-3	ENSMUST00000213072	ENSMUSG00000031849
mmu_circ_0001255	mmu-miR-486a-3	ENSMUST00000000128	ENSMUSG00000000126
mmu_circ_0001255	mmu-miR-486a-3	ENSMUST00000108783	ENSMUSG00000000126
mmu_circ_0001255	mmu-miR-486a-3	ENSMUST00000213072	ENSMUSG00000031849
mmu_circ_0001256	mmu-miR-486a-3	ENSMUST00000000128	ENSMUSG00000000126
mmu_circ_0001256	mmu-miR-486a-3	ENSMUST00000108783	ENSMUSG00000000126
mmu_circ_0001256	mmu-miR-486a-3	ENSMUST00000213072	ENSMUSG00000031849
mmu_circ_0001623	mmu-miR-486a-3	ENSMUST00000000128	ENSMUSG00000000126
mmu_circ_0001623	mmu-miR-486a-3	ENSMUST00000108783	ENSMUSG00000000126
mmu_circ_0001623	mmu-miR-486a-3	ENSMUST00000213072	ENSMUSG00000031849
mmu_circ_0001505	mmu-miR-486a-3	ENSMUST00000000128	ENSMUSG00000000126
mmu_circ_0001505	mmu-miR-486a-3	ENSMUST00000108783	ENSMUSG00000000126
mmu_circ_0001505	mmu-miR-486a-3	ENSMUST00000213072	ENSMUSG00000031849
mmu_circ_0001528	mmu-miR-486a-3	ENSMUST00000000128	ENSMUSG00000000126
mmu_circ_0001528	mmu-miR-486a-3	ENSMUST00000108783	ENSMUSG00000000126
mmu_circ_0001528	mmu-miR-486a-3	ENSMUST00000213072	ENSMUSG00000031849
mmu_circ_0001454	mmu-miR-486a-3	ENSMUST00000000128	ENSMUSG00000000126
mmu_circ_0001454	mmu-miR-486a-3	ENSMUST00000108783	ENSMUSG00000000126
mmu_circ_0001454	mmu-miR-486a-3	ENSMUST00000213072	ENSMUSG00000031849
mmu_circ_0001500	mmu-miR-486a-3	ENSMUST00000000128	ENSMUSG00000000126
mmu_circ_0001500	mmu-miR-486a-3	ENSMUST00000108783	ENSMUSG00000000126
mmu_circ_0001500	mmu-miR-486a-3	ENSMUST00000213072	ENSMUSG00000031849
mmu_circ_0001501	mmu-miR-486a-3	ENSMUST00000000128	ENSMUSG00000000126
mmu_circ_0001501	mmu-miR-486a-3	ENSMUST00000108783	ENSMUSG00000000126
mmu_circ_0001501	mmu-miR-486a-3	ENSMUST00000213072	ENSMUSG00000031849
mmu_circ_0001755	mmu-miR-486a-3	ENSMUST00000000128	ENSMUSG00000000126
mmu_circ_0001755	mmu-miR-486a-3	ENSMUST00000108783	ENSMUSG00000000126
mmu_circ_0001755	mmu-miR-486a-3	ENSMUST00000213072	ENSMUSG00000031849
mmu_circ_0001886	mmu-miR-486a-3	ENSMUST00000000128	ENSMUSG00000000126
mmu_circ_0001886	mmu-miR-486a-3	ENSMUST00000108783	ENSMUSG00000000126
mmu_circ_0001886	mmu-miR-486a-3	ENSMUST00000213072	ENSMUSG00000031849
mmu_circ_0000325	mmu-miR-486a-3	ENSMUST00000000128	ENSMUSG00000000126
mmu_circ_0000325	mmu-miR-486a-3	ENSMUST00000108783	ENSMUSG00000000126
mmu_circ_0000325	mmu-miR-486a-3	ENSMUST00000213072	ENSMUSG00000031849
mmu_circ_0000498	mmu-miR-486a-3	ENSMUST00000000128	ENSMUSG00000000126
mmu_circ_0000498	mmu-miR-486a-3	ENSMUST00000108783	ENSMUSG00000000126
mmu_circ_0000498	mmu-miR-486a-3	ENSMUST00000213072	ENSMUSG00000031849
mmu_circ_0000499	mmu-miR-486a-3	ENSMUST00000000128	ENSMUSG00000000126
mmu_circ_0000499	mmu-miR-486a-3	ENSMUST00000108783	ENSMUSG00000000126
mmu_circ_0000499	mmu-miR-486a-3	ENSMUST00000213072	ENSMUSG00000031849
mmu_circ_0000500	mmu-miR-486a-3	ENSMUST00000000128	ENSMUSG00000000126
mmu_circ_0000500	mmu-miR-486a-3	ENSMUST00000108783	ENSMUSG00000000126
mmu_circ_0000500	mmu-miR-486a-3	ENSMUST00000213072	ENSMUSG00000031849
mmu_circ_0000371	mmu-miR-486a-3	ENSMUST00000000128	ENSMUSG00000000126
mmu_circ_0000371	mmu-miR-486a-3	ENSMUST00000108783	ENSMUSG00000000126

mmu_circ_0000371	mmu-miR-486a-3	ENSMUST00000213072	ENSMUSG00000031849
mmu_circ_0000399	mmu-miR-486a-3	ENSMUST00000000128	ENSMUSG00000000126
mmu_circ_0000399	mmu-miR-486a-3	ENSMUST00000108783	ENSMUSG00000000126
mmu_circ_0000399	mmu-miR-486a-3	ENSMUST00000213072	ENSMUSG00000031849
mmu_circ_0000577	mmu-miR-486a-3	ENSMUST00000000128	ENSMUSG00000000126
mmu_circ_0000577	mmu-miR-486a-3	ENSMUST00000108783	ENSMUSG00000000126
mmu_circ_0000577	mmu-miR-486a-3	ENSMUST00000213072	ENSMUSG00000031849
mmu_circ_0000628	mmu-miR-486a-3	ENSMUST00000000128	ENSMUSG00000000126
mmu_circ_0000628	mmu-miR-486a-3	ENSMUST00000108783	ENSMUSG00000000126
mmu_circ_0000628	mmu-miR-486a-3	ENSMUST00000213072	ENSMUSG00000031849
mmu_circ_0000635	mmu-miR-486a-3	ENSMUST00000000128	ENSMUSG00000000126
mmu_circ_0000635	mmu-miR-486a-3	ENSMUST00000108783	ENSMUSG00000000126
mmu_circ_0000635	mmu-miR-486a-3	ENSMUST00000213072	ENSMUSG00000031849
mmu_circ_0000644	mmu-miR-486a-3	ENSMUST00000000128	ENSMUSG00000000126
mmu_circ_0000644	mmu-miR-486a-3	ENSMUST00000108783	ENSMUSG00000000126
mmu_circ_0000644	mmu-miR-486a-3	ENSMUST00000213072	ENSMUSG00000031849
mmu_circ_0000530	mmu-miR-486a-3	ENSMUST00000000128	ENSMUSG00000000126
mmu_circ_0000530	mmu-miR-486a-3	ENSMUST00000108783	ENSMUSG00000000126
mmu_circ_0000530	mmu-miR-486a-3	ENSMUST00000213072	ENSMUSG00000031849
mmu_circ_0000554	mmu-miR-486a-3	ENSMUST00000000128	ENSMUSG00000000126
mmu_circ_0000554	mmu-miR-486a-3	ENSMUST00000108783	ENSMUSG00000000126
mmu_circ_0000554	mmu-miR-486a-3	ENSMUST00000213072	ENSMUSG00000031849
mmu_circ_0000735	mmu-miR-486a-3	ENSMUST00000000128	ENSMUSG00000000126
mmu_circ_0000735	mmu-miR-486a-3	ENSMUST00000108783	ENSMUSG00000000126
mmu_circ_0000735	mmu-miR-486a-3	ENSMUST00000213072	ENSMUSG00000031849
mmu_circ_0000806	mmu-miR-486a-3	ENSMUST00000000128	ENSMUSG00000000126
mmu_circ_0000806	mmu-miR-486a-3	ENSMUST00000108783	ENSMUSG00000000126
mmu_circ_0000806	mmu-miR-486a-3	ENSMUST00000213072	ENSMUSG00000031849
mmu_circ_0000820	mmu-miR-486a-3	ENSMUST00000000128	ENSMUSG00000000126
mmu_circ_0000820	mmu-miR-486a-3	ENSMUST00000108783	ENSMUSG00000000126
mmu_circ_0000820	mmu-miR-486a-3	ENSMUST00000213072	ENSMUSG00000031849
mmu_circ_0000828	mmu-miR-486a-3	ENSMUST00000000128	ENSMUSG00000000126
mmu_circ_0000828	mmu-miR-486a-3	ENSMUST00000108783	ENSMUSG00000000126
mmu_circ_0000828	mmu-miR-486a-3	ENSMUST00000213072	ENSMUSG00000031849
mmu_circ_0000829	mmu-miR-486a-3	ENSMUST00000000128	ENSMUSG00000000126
mmu_circ_0000829	mmu-miR-486a-3	ENSMUST00000108783	ENSMUSG00000000126
mmu_circ_0000829	mmu-miR-486a-3	ENSMUST00000213072	ENSMUSG00000031849
mmu_circ_0000688	mmu-miR-486a-3	ENSMUST00000000128	ENSMUSG00000000126
mmu_circ_0000688	mmu-miR-486a-3	ENSMUST00000108783	ENSMUSG00000000126
mmu_circ_0000688	mmu-miR-486a-3	ENSMUST00000213072	ENSMUSG00000031849
mmu_circ_0000712	mmu-miR-486a-3	ENSMUST00000000128	ENSMUSG00000000126
mmu_circ_0000712	mmu-miR-486a-3	ENSMUST00000108783	ENSMUSG00000000126
mmu_circ_0000712	mmu-miR-486a-3	ENSMUST00000213072	ENSMUSG00000031849
mmu_circ_0000713	mmu-miR-486a-3	ENSMUST00000000128	ENSMUSG00000000126
mmu_circ_0000713	mmu-miR-486a-3	ENSMUST00000108783	ENSMUSG00000000126
mmu_circ_0000713	mmu-miR-486a-3	ENSMUST00000213072	ENSMUSG00000031849
mmu_circ_0000714	mmu-miR-486a-3	ENSMUST00000000128	ENSMUSG00000000126
mmu_circ_0000714	mmu-miR-486a-3	ENSMUST00000108783	ENSMUSG00000000126
mmu_circ_0000714	mmu-miR-486a-3	ENSMUST00000213072	ENSMUSG00000031849
mmu_circ_0000919	mmu-miR-486a-3	ENSMUST00000000128	ENSMUSG00000000126
mmu_circ_0000919	mmu-miR-486a-3	ENSMUST00000108783	ENSMUSG00000000126
mmu_circ_0000919	mmu-miR-486a-3	ENSMUST00000213072	ENSMUSG00000031849
mmu_circ_0000921	mmu-miR-486a-3	ENSMUST00000000128	ENSMUSG00000000126
mmu_circ_0000921	mmu-miR-486a-3	ENSMUST00000108783	ENSMUSG00000000126
mmu_circ_0000921	mmu-miR-486a-3	ENSMUST00000213072	ENSMUSG00000031849
mmu_circ_0000969	mmu-miR-486a-3	ENSMUST00000000128	ENSMUSG00000000126
mmu_circ_0000969	mmu-miR-486a-3	ENSMUST00000108783	ENSMUSG00000000126
mmu_circ_0000969	mmu-miR-486a-3	ENSMUST00000213072	ENSMUSG00000031849

mmu_circ_0000884	mmu-miR-486a-3	ENSMUST00000000128	ENSMUSG00000000126
mmu_circ_0000884	mmu-miR-486a-3	ENSMUST00000108783	ENSMUSG00000000126
mmu_circ_0000884	mmu-miR-486a-3	ENSMUST00000213072	ENSMUSG00000031849
mmu_circ_0000886	mmu-miR-486a-3	ENSMUST00000000128	ENSMUSG00000000126
mmu_circ_0000886	mmu-miR-486a-3	ENSMUST00000108783	ENSMUSG00000000126
mmu_circ_0000886	mmu-miR-486a-3	ENSMUST00000213072	ENSMUSG00000031849
mmu_circ_0000887	mmu-miR-486a-3	ENSMUST00000000128	ENSMUSG00000000126
mmu_circ_0000887	mmu-miR-486a-3	ENSMUST00000108783	ENSMUSG00000000126
mmu_circ_0000887	mmu-miR-486a-3	ENSMUST00000213072	ENSMUSG00000031849
mmu_circ_0000905	mmu-miR-486a-3	ENSMUST00000000128	ENSMUSG00000000126
mmu_circ_0000905	mmu-miR-486a-3	ENSMUST00000108783	ENSMUSG00000000126
mmu_circ_0000905	mmu-miR-486a-3	ENSMUST00000213072	ENSMUSG00000031849
mmu_circ_0000906	mmu-miR-486a-3	ENSMUST00000000128	ENSMUSG00000000126
mmu_circ_0000906	mmu-miR-486a-3	ENSMUST00000108783	ENSMUSG00000000126
mmu_circ_0000906	mmu-miR-486a-3	ENSMUST00000213072	ENSMUSG00000031849
mmu_circ_0000907	mmu-miR-486a-3	ENSMUST00000000128	ENSMUSG00000000126
mmu_circ_0000907	mmu-miR-486a-3	ENSMUST00000108783	ENSMUSG00000000126
mmu_circ_0000907	mmu-miR-486a-3	ENSMUST00000213072	ENSMUSG00000031849
mmu_circ_0000908	mmu-miR-486a-3	ENSMUST00000000128	ENSMUSG00000000126
mmu_circ_0000908	mmu-miR-486a-3	ENSMUST00000108783	ENSMUSG00000000126
mmu_circ_0000908	mmu-miR-486a-3	ENSMUST00000213072	ENSMUSG00000031849
mmu_circ_0000912	mmu-miR-486a-3	ENSMUST00000000128	ENSMUSG00000000126
mmu_circ_0000912	mmu-miR-486a-3	ENSMUST00000108783	ENSMUSG00000000126
mmu_circ_0000912	mmu-miR-486a-3	ENSMUST00000213072	ENSMUSG00000031849
novel_circ_0001676	mmu-miR-486a-3	ENSMUST00000000128	ENSMUSG00000000126
novel_circ_0001676	mmu-miR-486a-3	ENSMUST00000108783	ENSMUSG00000000126
novel_circ_0001676	mmu-miR-486a-3	ENSMUST00000213072	ENSMUSG00000031849
novel_circ_0001726	mmu-miR-486a-3	ENSMUST00000000128	ENSMUSG00000000126
novel_circ_0001726	mmu-miR-486a-3	ENSMUST00000108783	ENSMUSG00000000126
novel_circ_0001726	mmu-miR-486a-3	ENSMUST00000213072	ENSMUSG00000031849
novel_circ_0002100	mmu-miR-486a-3	ENSMUST00000000128	ENSMUSG00000000126
novel_circ_0002100	mmu-miR-486a-3	ENSMUST00000108783	ENSMUSG00000000126
novel_circ_0002100	mmu-miR-486a-3	ENSMUST00000213072	ENSMUSG00000031849
novel_circ_0002180	mmu-miR-486a-3	ENSMUST00000000128	ENSMUSG00000000126
novel_circ_0002180	mmu-miR-486a-3	ENSMUST00000108783	ENSMUSG00000000126
novel_circ_0002180	mmu-miR-486a-3	ENSMUST00000213072	ENSMUSG00000031849
novel_circ_0002217	mmu-miR-486a-3	ENSMUST00000000128	ENSMUSG00000000126
novel_circ_0002217	mmu-miR-486a-3	ENSMUST00000108783	ENSMUSG00000000126
novel_circ_0002217	mmu-miR-486a-3	ENSMUST00000213072	ENSMUSG00000031849
novel_circ_0001751	mmu-miR-486a-3	ENSMUST00000000128	ENSMUSG00000000126
novel_circ_0001751	mmu-miR-486a-3	ENSMUST00000108783	ENSMUSG00000000126
novel_circ_0001751	mmu-miR-486a-3	ENSMUST00000213072	ENSMUSG00000031849
novel_circ_0001795	mmu-miR-486a-3	ENSMUST00000000128	ENSMUSG00000000126
novel_circ_0001795	mmu-miR-486a-3	ENSMUST00000108783	ENSMUSG00000000126
novel_circ_0001795	mmu-miR-486a-3	ENSMUST00000213072	ENSMUSG00000031849
novel_circ_0002027	mmu-miR-486a-3	ENSMUST00000000128	ENSMUSG00000000126
novel_circ_0002027	mmu-miR-486a-3	ENSMUST00000108783	ENSMUSG00000000126
novel_circ_0002027	mmu-miR-486a-3	ENSMUST00000213072	ENSMUSG00000031849
novel_circ_0002027	mmu-miR-486a-3	ENSMUST00000000128	ENSMUSG00000000126
novel_circ_0002027	mmu-miR-486a-3	ENSMUST00000108783	ENSMUSG00000000126
novel_circ_0002027	mmu-miR-486a-3	ENSMUST00000213072	ENSMUSG00000031849
novel_circ_0002447	mmu-miR-486a-3	ENSMUST00000000128	ENSMUSG00000000126
novel_circ_0002447	mmu-miR-486a-3	ENSMUST00000108783	ENSMUSG00000000126
novel_circ_0002447	mmu-miR-486a-3	ENSMUST00000213072	ENSMUSG00000031849
novel_circ_0002448	mmu-miR-486a-3	ENSMUST00000000128	ENSMUSG00000000126
novel_circ_0002448	mmu-miR-486a-3	ENSMUST00000108783	ENSMUSG00000000126
novel_circ_0002448	mmu-miR-486a-3	ENSMUST00000213072	ENSMUSG00000031849
novel_circ_0002484	mmu-miR-486a-3	ENSMUST00000000128	ENSMUSG00000000126

novel_circ_0002484	mmu-miR-486a-3	ENSMUST00000108783	ENSMUSG00000000126
novel_circ_0002484	mmu-miR-486a-3	ENSMUST00000213072	ENSMUSG00000031849
novel_circ_0002485	mmu-miR-486a-3	ENSMUST00000000128	ENSMUSG00000000126
novel_circ_0002485	mmu-miR-486a-3	ENSMUST00000108783	ENSMUSG00000000126
novel_circ_0002485	mmu-miR-486a-3	ENSMUST00000213072	ENSMUSG00000031849
novel_circ_0002494	mmu-miR-486a-3	ENSMUST00000000128	ENSMUSG00000000126
novel_circ_0002494	mmu-miR-486a-3	ENSMUST00000108783	ENSMUSG00000000126
novel_circ_0002494	mmu-miR-486a-3	ENSMUST00000213072	ENSMUSG00000031849
novel_circ_0002497	mmu-miR-486a-3	ENSMUST00000000128	ENSMUSG00000000126
novel_circ_0002497	mmu-miR-486a-3	ENSMUST00000108783	ENSMUSG00000000126
novel_circ_0002497	mmu-miR-486a-3	ENSMUST00000213072	ENSMUSG00000031849
novel_circ_0002597	mmu-miR-486a-3	ENSMUST00000000128	ENSMUSG00000000126
novel_circ_0002597	mmu-miR-486a-3	ENSMUST00000108783	ENSMUSG00000000126
novel_circ_0002597	mmu-miR-486a-3	ENSMUST00000213072	ENSMUSG00000031849
novel_circ_0002241	mmu-miR-486a-3	ENSMUST00000000128	ENSMUSG00000000126
novel_circ_0002241	mmu-miR-486a-3	ENSMUST00000108783	ENSMUSG00000000126
novel_circ_0002241	mmu-miR-486a-3	ENSMUST00000213072	ENSMUSG00000031849
novel_circ_0002257	mmu-miR-486a-3	ENSMUST00000000128	ENSMUSG00000000126
novel_circ_0002257	mmu-miR-486a-3	ENSMUST00000108783	ENSMUSG00000000126
novel_circ_0002257	mmu-miR-486a-3	ENSMUST00000213072	ENSMUSG00000031849
novel_circ_0002299	mmu-miR-486a-3	ENSMUST00000000128	ENSMUSG00000000126
novel_circ_0002299	mmu-miR-486a-3	ENSMUST00000108783	ENSMUSG00000000126
novel_circ_0002299	mmu-miR-486a-3	ENSMUST00000213072	ENSMUSG00000031849
novel_circ_0002336	mmu-miR-486a-3	ENSMUST00000000128	ENSMUSG00000000126
novel_circ_0002336	mmu-miR-486a-3	ENSMUST00000108783	ENSMUSG00000000126
novel_circ_0002336	mmu-miR-486a-3	ENSMUST00000213072	ENSMUSG00000031849
novel_circ_0002336	mmu-miR-486a-3	ENSMUST00000000128	ENSMUSG00000000126
novel_circ_0002336	mmu-miR-486a-3	ENSMUST00000108783	ENSMUSG00000000126
novel_circ_0002336	mmu-miR-486a-3	ENSMUST00000213072	ENSMUSG00000031849
novel_circ_0002343	mmu-miR-486a-3	ENSMUST00000000128	ENSMUSG00000000126
novel_circ_0002343	mmu-miR-486a-3	ENSMUST00000108783	ENSMUSG00000000126
novel_circ_0002343	mmu-miR-486a-3	ENSMUST00000213072	ENSMUSG00000031849
novel_circ_0002914	mmu-miR-486a-3	ENSMUST00000000128	ENSMUSG00000000126
novel_circ_0002914	mmu-miR-486a-3	ENSMUST00000108783	ENSMUSG00000000126
novel_circ_0002914	mmu-miR-486a-3	ENSMUST00000213072	ENSMUSG00000031849
novel_circ_0002917	mmu-miR-486a-3	ENSMUST00000000128	ENSMUSG00000000126
novel_circ_0002917	mmu-miR-486a-3	ENSMUST00000108783	ENSMUSG00000000126
novel_circ_0002917	mmu-miR-486a-3	ENSMUST00000213072	ENSMUSG00000031849
novel_circ_0002943	mmu-miR-486a-3	ENSMUST00000000128	ENSMUSG00000000126
novel_circ_0002943	mmu-miR-486a-3	ENSMUST00000108783	ENSMUSG00000000126
novel_circ_0002943	mmu-miR-486a-3	ENSMUST00000213072	ENSMUSG00000031849
novel_circ_0002943	mmu-miR-486a-3	ENSMUST00000000128	ENSMUSG00000000126
novel_circ_0002943	mmu-miR-486a-3	ENSMUST00000108783	ENSMUSG00000000126
novel_circ_0002943	mmu-miR-486a-3	ENSMUST00000213072	ENSMUSG00000031849
novel_circ_0002648	mmu-miR-486a-3	ENSMUST00000000128	ENSMUSG00000000126
novel_circ_0002648	mmu-miR-486a-3	ENSMUST00000108783	ENSMUSG00000000126
novel_circ_0002648	mmu-miR-486a-3	ENSMUST00000213072	ENSMUSG00000031849
novel_circ_0002722	mmu-miR-486a-3	ENSMUST00000000128	ENSMUSG00000000126
novel_circ_0002722	mmu-miR-486a-3	ENSMUST00000108783	ENSMUSG00000000126
novel_circ_0002722	mmu-miR-486a-3	ENSMUST00000213072	ENSMUSG00000031849
novel_circ_0002788	mmu-miR-486a-3	ENSMUST00000000128	ENSMUSG00000000126
novel_circ_0002788	mmu-miR-486a-3	ENSMUST00000108783	ENSMUSG00000000126
novel_circ_0002788	mmu-miR-486a-3	ENSMUST00000213072	ENSMUSG00000031849
novel_circ_0003213	mmu-miR-486a-3	ENSMUST00000000128	ENSMUSG00000000126
novel_circ_0003213	mmu-miR-486a-3	ENSMUST00000108783	ENSMUSG00000000126
novel_circ_0003213	mmu-miR-486a-3	ENSMUST00000213072	ENSMUSG00000031849
novel_circ_0003262	mmu-miR-486a-3	ENSMUST00000000128	ENSMUSG00000000126
novel_circ_0003262	mmu-miR-486a-3	ENSMUST00000108783	ENSMUSG00000000126

novel_circ_0003262	mmu-miR-486a-3	ENSMUST00000213072	ENSMUSG00000031849
novel_circ_0003262	mmu-miR-486a-3	ENSMUST00000000128	ENSMUSG00000000126
novel_circ_0003262	mmu-miR-486a-3	ENSMUST00000108783	ENSMUSG00000000126
novel_circ_0003262	mmu-miR-486a-3	ENSMUST00000213072	ENSMUSG00000031849
novel_circ_0003309	mmu-miR-486a-3	ENSMUST00000000128	ENSMUSG00000000126
novel_circ_0003309	mmu-miR-486a-3	ENSMUST00000108783	ENSMUSG00000000126
novel_circ_0003309	mmu-miR-486a-3	ENSMUST00000213072	ENSMUSG00000031849
novel_circ_0003053	mmu-miR-486a-3	ENSMUST00000000128	ENSMUSG00000000126
novel_circ_0003053	mmu-miR-486a-3	ENSMUST00000108783	ENSMUSG00000000126
novel_circ_0003053	mmu-miR-486a-3	ENSMUST00000213072	ENSMUSG00000031849
novel_circ_0003462	mmu-miR-486a-3	ENSMUST00000000128	ENSMUSG00000000126
novel_circ_0003462	mmu-miR-486a-3	ENSMUST00000108783	ENSMUSG00000000126
novel_circ_0003462	mmu-miR-486a-3	ENSMUST00000213072	ENSMUSG00000031849
novel_circ_0000263	mmu-miR-486a-3	ENSMUST00000000128	ENSMUSG00000000126
novel_circ_0000263	mmu-miR-486a-3	ENSMUST00000108783	ENSMUSG00000000126
novel_circ_0000263	mmu-miR-486a-3	ENSMUST00000213072	ENSMUSG00000031849
novel_circ_0000287	mmu-miR-486a-3	ENSMUST00000000128	ENSMUSG00000000126
novel_circ_0000287	mmu-miR-486a-3	ENSMUST00000108783	ENSMUSG00000000126
novel_circ_0000287	mmu-miR-486a-3	ENSMUST00000213072	ENSMUSG00000031849
novel_circ_0000291	mmu-miR-486a-3	ENSMUST00000000128	ENSMUSG00000000126
novel_circ_0000291	mmu-miR-486a-3	ENSMUST00000108783	ENSMUSG00000000126
novel_circ_0000291	mmu-miR-486a-3	ENSMUST00000213072	ENSMUSG00000031849
novel_circ_0000291	mmu-miR-486a-3	ENSMUST00000000128	ENSMUSG00000000126
novel_circ_0000291	mmu-miR-486a-3	ENSMUST00000108783	ENSMUSG00000000126
novel_circ_0000291	mmu-miR-486a-3	ENSMUST00000213072	ENSMUSG00000031849
novel_circ_0000292	mmu-miR-486a-3	ENSMUST00000000128	ENSMUSG00000000126
novel_circ_0000292	mmu-miR-486a-3	ENSMUST00000108783	ENSMUSG00000000126
novel_circ_0000292	mmu-miR-486a-3	ENSMUST00000213072	ENSMUSG00000031849
novel_circ_0000292	mmu-miR-486a-3	ENSMUST00000000128	ENSMUSG00000000126
novel_circ_0000292	mmu-miR-486a-3	ENSMUST00000108783	ENSMUSG00000000126
novel_circ_0000292	mmu-miR-486a-3	ENSMUST00000213072	ENSMUSG00000031849
novel_circ_0000400	mmu-miR-486a-3	ENSMUST00000000128	ENSMUSG00000000126
novel_circ_0000400	mmu-miR-486a-3	ENSMUST00000108783	ENSMUSG00000000126
novel_circ_0000400	mmu-miR-486a-3	ENSMUST00000213072	ENSMUSG00000031849
novel_circ_0000022	mmu-miR-486a-3	ENSMUST00000000128	ENSMUSG00000000126
novel_circ_0000022	mmu-miR-486a-3	ENSMUST00000108783	ENSMUSG00000000126
novel_circ_0000022	mmu-miR-486a-3	ENSMUST00000213072	ENSMUSG00000031849
novel_circ_0000068	mmu-miR-486a-3	ENSMUST00000000128	ENSMUSG00000000126
novel_circ_0000068	mmu-miR-486a-3	ENSMUST00000108783	ENSMUSG00000000126
novel_circ_0000068	mmu-miR-486a-3	ENSMUST00000213072	ENSMUSG00000031849
novel_circ_0000129	mmu-miR-486a-3	ENSMUST00000000128	ENSMUSG00000000126
novel_circ_0000129	mmu-miR-486a-3	ENSMUST00000108783	ENSMUSG00000000126
novel_circ_0000129	mmu-miR-486a-3	ENSMUST00000213072	ENSMUSG00000031849
novel_circ_0000131	mmu-miR-486a-3	ENSMUST00000000128	ENSMUSG00000000126
novel_circ_0000131	mmu-miR-486a-3	ENSMUST00000108783	ENSMUSG00000000126
novel_circ_0000131	mmu-miR-486a-3	ENSMUST00000213072	ENSMUSG00000031849
novel_circ_0000132	mmu-miR-486a-3	ENSMUST00000000128	ENSMUSG00000000126
novel_circ_0000132	mmu-miR-486a-3	ENSMUST00000108783	ENSMUSG00000000126
novel_circ_0000132	mmu-miR-486a-3	ENSMUST00000213072	ENSMUSG00000031849
novel_circ_0000687	mmu-miR-486a-3	ENSMUST00000000128	ENSMUSG00000000126
novel_circ_0000687	mmu-miR-486a-3	ENSMUST00000108783	ENSMUSG00000000126
novel_circ_0000687	mmu-miR-486a-3	ENSMUST00000213072	ENSMUSG00000031849
novel_circ_0000692	mmu-miR-486a-3	ENSMUST00000000128	ENSMUSG00000000126
novel_circ_0000692	mmu-miR-486a-3	ENSMUST00000108783	ENSMUSG00000000126
novel_circ_0000692	mmu-miR-486a-3	ENSMUST00000213072	ENSMUSG00000031849
novel_circ_0000696	mmu-miR-486a-3	ENSMUST00000000128	ENSMUSG00000000126
novel_circ_0000696	mmu-miR-486a-3	ENSMUST00000108783	ENSMUSG00000000126
novel_circ_0000696	mmu-miR-486a-3	ENSMUST00000213072	ENSMUSG00000031849

novel_circ_0000698	mmu-miR-486a-3	ENSMUST00000000128	ENSMUSG00000000126
novel_circ_0000698	mmu-miR-486a-3	ENSMUST00000108783	ENSMUSG00000000126
novel_circ_0000698	mmu-miR-486a-3	ENSMUST00000213072	ENSMUSG00000031849
novel_circ_0000772	mmu-miR-486a-3	ENSMUST00000000128	ENSMUSG00000000126
novel_circ_0000772	mmu-miR-486a-3	ENSMUST00000108783	ENSMUSG00000000126
novel_circ_0000772	mmu-miR-486a-3	ENSMUST00000213072	ENSMUSG00000031849
novel_circ_0000589	mmu-miR-486a-3	ENSMUST00000000128	ENSMUSG00000000126
novel_circ_0000589	mmu-miR-486a-3	ENSMUST00000108783	ENSMUSG00000000126
novel_circ_0000589	mmu-miR-486a-3	ENSMUST00000213072	ENSMUSG00000031849
novel_circ_0000594	mmu-miR-486a-3	ENSMUST00000000128	ENSMUSG00000000126
novel_circ_0000594	mmu-miR-486a-3	ENSMUST00000108783	ENSMUSG00000000126
novel_circ_0000594	mmu-miR-486a-3	ENSMUST00000213072	ENSMUSG00000031849
novel_circ_0001046	mmu-miR-486a-3	ENSMUST00000000128	ENSMUSG00000000126
novel_circ_0001046	mmu-miR-486a-3	ENSMUST00000108783	ENSMUSG00000000126
novel_circ_0001046	mmu-miR-486a-3	ENSMUST00000213072	ENSMUSG00000031849
novel_circ_0000900	mmu-miR-486a-3	ENSMUST00000000128	ENSMUSG00000000126
novel_circ_0000900	mmu-miR-486a-3	ENSMUST00000108783	ENSMUSG00000000126
novel_circ_0000900	mmu-miR-486a-3	ENSMUST00000213072	ENSMUSG00000031849
novel_circ_0000901	mmu-miR-486a-3	ENSMUST00000000128	ENSMUSG00000000126
novel_circ_0000901	mmu-miR-486a-3	ENSMUST00000108783	ENSMUSG00000000126
novel_circ_0000901	mmu-miR-486a-3	ENSMUST00000213072	ENSMUSG00000031849
novel_circ_0001177	mmu-miR-486a-3	ENSMUST00000000128	ENSMUSG00000000126
novel_circ_0001177	mmu-miR-486a-3	ENSMUST00000108783	ENSMUSG00000000126
novel_circ_0001177	mmu-miR-486a-3	ENSMUST00000213072	ENSMUSG00000031849
novel_circ_0001179	mmu-miR-486a-3	ENSMUST00000000128	ENSMUSG00000000126
novel_circ_0001179	mmu-miR-486a-3	ENSMUST00000108783	ENSMUSG00000000126
novel_circ_0001179	mmu-miR-486a-3	ENSMUST00000213072	ENSMUSG00000031849
novel_circ_0001206	mmu-miR-486a-3	ENSMUST00000000128	ENSMUSG00000000126
novel_circ_0001206	mmu-miR-486a-3	ENSMUST00000108783	ENSMUSG00000000126
novel_circ_0001206	mmu-miR-486a-3	ENSMUST00000213072	ENSMUSG00000031849
novel_circ_0001083	mmu-miR-540-5p	ENSMUST00000000128	ENSMUSG00000000126
novel_circ_0001083	mmu-miR-540-5p	ENSMUST00000108783	ENSMUSG00000000126
novel_circ_0001086	mmu-miR-540-5p	ENSMUST00000000128	ENSMUSG00000000126
novel_circ_0001086	mmu-miR-540-5p	ENSMUST00000108783	ENSMUSG00000000126
novel_circ_0001112	mmu-miR-540-5p	ENSMUST00000000128	ENSMUSG00000000126
novel_circ_0001112	mmu-miR-540-5p	ENSMUST00000108783	ENSMUSG00000000126
novel_circ_0001458	mmu-miR-540-5p	ENSMUST00000000128	ENSMUSG00000000126
novel_circ_0001458	mmu-miR-540-5p	ENSMUST00000108783	ENSMUSG00000000126
novel_circ_0001363	mmu-miR-540-5p	ENSMUST00000000128	ENSMUSG00000000126
novel_circ_0001363	mmu-miR-540-5p	ENSMUST00000108783	ENSMUSG00000000126
novel_circ_0001363	mmu-miR-540-5p	ENSMUST00000000128	ENSMUSG00000000126
novel_circ_0001363	mmu-miR-540-5p	ENSMUST00000108783	ENSMUSG00000000126
mmu_circ_0000042	mmu-miR-540-5p	ENSMUST00000000128	ENSMUSG00000000126
mmu_circ_0000042	mmu-miR-540-5p	ENSMUST00000108783	ENSMUSG00000000126
mmu_circ_0001123	mmu-miR-540-5p	ENSMUST00000000128	ENSMUSG00000000126
mmu_circ_0001123	mmu-miR-540-5p	ENSMUST00000108783	ENSMUSG00000000126
mmu_circ_0001099	mmu-miR-540-5p	ENSMUST00000000128	ENSMUSG00000000126
mmu_circ_0001099	mmu-miR-540-5p	ENSMUST00000108783	ENSMUSG00000000126
mmu_circ_0001023	mmu-miR-540-5p	ENSMUST00000000128	ENSMUSG00000000126
mmu_circ_0001023	mmu-miR-540-5p	ENSMUST00000108783	ENSMUSG00000000126
mmu_circ_0001368	mmu-miR-540-5p	ENSMUST00000000128	ENSMUSG00000000126
mmu_circ_0001368	mmu-miR-540-5p	ENSMUST00000108783	ENSMUSG00000000126
mmu_circ_0001376	mmu-miR-540-5p	ENSMUST00000000128	ENSMUSG00000000126
mmu_circ_0001376	mmu-miR-540-5p	ENSMUST00000108783	ENSMUSG00000000126
mmu_circ_0001190	mmu-miR-540-5p	ENSMUST00000000128	ENSMUSG00000000126
mmu_circ_0001190	mmu-miR-540-5p	ENSMUST00000108783	ENSMUSG00000000126
mmu_circ_0001842	mmu-miR-540-5p	ENSMUST00000000128	ENSMUSG00000000126
mmu_circ_0001842	mmu-miR-540-5p	ENSMUST00000108783	ENSMUSG00000000126

mmu_circ_0001754	mmu-miR-540-5p	ENSMUST00000000128	ENSMUSG00000000126
mmu_circ_0001754	mmu-miR-540-5p	ENSMUST00000108783	ENSMUSG00000000126
mmu_circ_0001754	mmu-miR-540-5p	ENSMUST00000000128	ENSMUSG00000000126
mmu_circ_0001754	mmu-miR-540-5p	ENSMUST00000108783	ENSMUSG00000000126
mmu_circ_0001827	mmu-miR-540-5p	ENSMUST00000000128	ENSMUSG00000000126
mmu_circ_0001827	mmu-miR-540-5p	ENSMUST00000108783	ENSMUSG00000000126
mmu_circ_0000271	mmu-miR-540-5p	ENSMUST00000000128	ENSMUSG00000000126
mmu_circ_0000271	mmu-miR-540-5p	ENSMUST00000108783	ENSMUSG00000000126
mmu_circ_0000499	mmu-miR-540-5p	ENSMUST00000000128	ENSMUSG00000000126
mmu_circ_0000499	mmu-miR-540-5p	ENSMUST00000108783	ENSMUSG00000000126
mmu_circ_0000500	mmu-miR-540-5p	ENSMUST00000000128	ENSMUSG00000000126
mmu_circ_0000500	mmu-miR-540-5p	ENSMUST00000108783	ENSMUSG00000000126
mmu_circ_0000448	mmu-miR-540-5p	ENSMUST00000000128	ENSMUSG00000000126
mmu_circ_0000448	mmu-miR-540-5p	ENSMUST00000108783	ENSMUSG00000000126
mmu_circ_0000457	mmu-miR-540-5p	ENSMUST00000000128	ENSMUSG00000000126
mmu_circ_0000457	mmu-miR-540-5p	ENSMUST00000108783	ENSMUSG00000000126
mmu_circ_0000465	mmu-miR-540-5p	ENSMUST00000000128	ENSMUSG00000000126
mmu_circ_0000465	mmu-miR-540-5p	ENSMUST00000108783	ENSMUSG00000000126
mmu_circ_0000399	mmu-miR-540-5p	ENSMUST00000000128	ENSMUSG00000000126
mmu_circ_0000399	mmu-miR-540-5p	ENSMUST00000108783	ENSMUSG00000000126
mmu_circ_0000628	mmu-miR-540-5p	ENSMUST00000000128	ENSMUSG00000000126
mmu_circ_0000628	mmu-miR-540-5p	ENSMUST00000108783	ENSMUSG00000000126
mmu_circ_0000530	mmu-miR-540-5p	ENSMUST00000000128	ENSMUSG00000000126
mmu_circ_0000530	mmu-miR-540-5p	ENSMUST00000108783	ENSMUSG00000000126
mmu_circ_0000555	mmu-miR-540-5p	ENSMUST00000000128	ENSMUSG00000000126
mmu_circ_0000555	mmu-miR-540-5p	ENSMUST00000108783	ENSMUSG00000000126
mmu_circ_0000798	mmu-miR-540-5p	ENSMUST00000000128	ENSMUSG00000000126
mmu_circ_0000798	mmu-miR-540-5p	ENSMUST00000108783	ENSMUSG00000000126
mmu_circ_0000829	mmu-miR-540-5p	ENSMUST00000000128	ENSMUSG00000000126
mmu_circ_0000829	mmu-miR-540-5p	ENSMUST00000108783	ENSMUSG00000000126
mmu_circ_0000831	mmu-miR-540-5p	ENSMUST00000000128	ENSMUSG00000000126
mmu_circ_0000831	mmu-miR-540-5p	ENSMUST00000108783	ENSMUSG00000000126
mmu_circ_0000944	mmu-miR-540-5p	ENSMUST00000000128	ENSMUSG00000000126
mmu_circ_0000944	mmu-miR-540-5p	ENSMUST00000108783	ENSMUSG00000000126
mmu_circ_0000969	mmu-miR-540-5p	ENSMUST00000000128	ENSMUSG00000000126
mmu_circ_0000969	mmu-miR-540-5p	ENSMUST00000108783	ENSMUSG00000000126
mmu_circ_0000903	mmu-miR-540-5p	ENSMUST00000000128	ENSMUSG00000000126
mmu_circ_0000903	mmu-miR-540-5p	ENSMUST00000108783	ENSMUSG00000000126
mmu_circ_0000912	mmu-miR-540-5p	ENSMUST00000000128	ENSMUSG00000000126
mmu_circ_0000912	mmu-miR-540-5p	ENSMUST00000108783	ENSMUSG00000000126
novel_circ_0001661	mmu-miR-540-5p	ENSMUST00000000128	ENSMUSG00000000126
novel_circ_0001661	mmu-miR-540-5p	ENSMUST00000108783	ENSMUSG00000000126
novel_circ_0001661	mmu-miR-540-5p	ENSMUST00000000128	ENSMUSG00000000126
novel_circ_0001661	mmu-miR-540-5p	ENSMUST00000108783	ENSMUSG00000000126
novel_circ_0002100	mmu-miR-540-5p	ENSMUST00000000128	ENSMUSG00000000126
novel_circ_0002100	mmu-miR-540-5p	ENSMUST00000108783	ENSMUSG00000000126
novel_circ_0001782	mmu-miR-540-5p	ENSMUST00000000128	ENSMUSG00000000126
novel_circ_0001782	mmu-miR-540-5p	ENSMUST00000108783	ENSMUSG00000000126
novel_circ_0001783	mmu-miR-540-5p	ENSMUST00000000128	ENSMUSG00000000126
novel_circ_0001783	mmu-miR-540-5p	ENSMUST00000108783	ENSMUSG00000000126
novel_circ_0001871	mmu-miR-540-5p	ENSMUST00000000128	ENSMUSG00000000126
novel_circ_0001871	mmu-miR-540-5p	ENSMUST00000108783	ENSMUSG00000000126
novel_circ_0002468	mmu-miR-540-5p	ENSMUST00000000128	ENSMUSG00000000126
novel_circ_0002468	mmu-miR-540-5p	ENSMUST00000108783	ENSMUSG00000000126
novel_circ_0002225	mmu-miR-540-5p	ENSMUST00000000128	ENSMUSG00000000126
novel_circ_0002225	mmu-miR-540-5p	ENSMUST00000108783	ENSMUSG00000000126
novel_circ_0002321	mmu-miR-540-5p	ENSMUST00000000128	ENSMUSG00000000126
novel_circ_0002321	mmu-miR-540-5p	ENSMUST00000108783	ENSMUSG00000000126

novel_circ_0002322	mmu-miR-540-5p	ENSMUST00000000128	ENSMUSG00000000126
novel_circ_0002322	mmu-miR-540-5p	ENSMUST00000108783	ENSMUSG00000000126
novel_circ_0002831	mmu-miR-540-5p	ENSMUST00000000128	ENSMUSG00000000126
novel_circ_0002831	mmu-miR-540-5p	ENSMUST00000108783	ENSMUSG00000000126
novel_circ_0002866	mmu-miR-540-5p	ENSMUST00000000128	ENSMUSG00000000126
novel_circ_0002866	mmu-miR-540-5p	ENSMUST00000108783	ENSMUSG00000000126
novel_circ_0002691	mmu-miR-540-5p	ENSMUST00000000128	ENSMUSG00000000126
novel_circ_0002691	mmu-miR-540-5p	ENSMUST00000108783	ENSMUSG00000000126
novel_circ_0002746	mmu-miR-540-5p	ENSMUST00000000128	ENSMUSG00000000126
novel_circ_0002746	mmu-miR-540-5p	ENSMUST00000108783	ENSMUSG00000000126
novel_circ_0003292	mmu-miR-540-5p	ENSMUST00000000128	ENSMUSG00000000126
novel_circ_0003292	mmu-miR-540-5p	ENSMUST00000108783	ENSMUSG00000000126
novel_circ_0003309	mmu-miR-540-5p	ENSMUST00000000128	ENSMUSG00000000126
novel_circ_0003309	mmu-miR-540-5p	ENSMUST00000108783	ENSMUSG00000000126
novel_circ_0003081	mmu-miR-540-5p	ENSMUST00000000128	ENSMUSG00000000126
novel_circ_0003081	mmu-miR-540-5p	ENSMUST00000108783	ENSMUSG00000000126
novel_circ_0003468	mmu-miR-540-5p	ENSMUST00000000128	ENSMUSG00000000126
novel_circ_0003468	mmu-miR-540-5p	ENSMUST00000108783	ENSMUSG00000000126
novel_circ_0000186	mmu-miR-540-5p	ENSMUST00000000128	ENSMUSG00000000126
novel_circ_0000186	mmu-miR-540-5p	ENSMUST00000108783	ENSMUSG00000000126
novel_circ_0000249	mmu-miR-540-5p	ENSMUST00000000128	ENSMUSG00000000126
novel_circ_0000249	mmu-miR-540-5p	ENSMUST00000108783	ENSMUSG00000000126
novel_circ_0000249	mmu-miR-540-5p	ENSMUST00000000128	ENSMUSG00000000126
novel_circ_0000249	mmu-miR-540-5p	ENSMUST00000108783	ENSMUSG00000000126
novel_circ_0000288	mmu-miR-540-5p	ENSMUST00000000128	ENSMUSG00000000126
novel_circ_0000288	mmu-miR-540-5p	ENSMUST00000108783	ENSMUSG00000000126
novel_circ_0000343	mmu-miR-540-5p	ENSMUST00000000128	ENSMUSG00000000126
novel_circ_0000343	mmu-miR-540-5p	ENSMUST00000108783	ENSMUSG00000000126
novel_circ_0000034	mmu-miR-540-5p	ENSMUST00000000128	ENSMUSG00000000126
novel_circ_0000034	mmu-miR-540-5p	ENSMUST00000108783	ENSMUSG00000000126
novel_circ_0000059	mmu-miR-540-5p	ENSMUST00000000128	ENSMUSG00000000126
novel_circ_0000059	mmu-miR-540-5p	ENSMUST00000108783	ENSMUSG00000000126
novel_circ_0000065	mmu-miR-540-5p	ENSMUST00000000128	ENSMUSG00000000126
novel_circ_0000065	mmu-miR-540-5p	ENSMUST00000108783	ENSMUSG00000000126
novel_circ_0000753	mmu-miR-540-5p	ENSMUST00000000128	ENSMUSG00000000126
novel_circ_0000753	mmu-miR-540-5p	ENSMUST00000108783	ENSMUSG00000000126
novel_circ_0000519	mmu-miR-540-5p	ENSMUST00000000128	ENSMUSG00000000126
novel_circ_0000519	mmu-miR-540-5p	ENSMUST00000108783	ENSMUSG00000000126
novel_circ_0000520	mmu-miR-540-5p	ENSMUST00000000128	ENSMUSG00000000126
novel_circ_0000520	mmu-miR-540-5p	ENSMUST00000108783	ENSMUSG00000000126
novel_circ_0000521	mmu-miR-540-5p	ENSMUST00000000128	ENSMUSG00000000126
novel_circ_0000521	mmu-miR-540-5p	ENSMUST00000108783	ENSMUSG00000000126
novel_circ_0001022	mmu-miR-540-5p	ENSMUST00000000128	ENSMUSG00000000126
novel_circ_0001022	mmu-miR-540-5p	ENSMUST00000108783	ENSMUSG00000000126
novel_circ_0001022	mmu-miR-540-5p	ENSMUST00000000128	ENSMUSG00000000126
novel_circ_0001022	mmu-miR-540-5p	ENSMUST00000108783	ENSMUSG00000000126
novel_circ_0001023	mmu-miR-540-5p	ENSMUST00000000128	ENSMUSG00000000126
novel_circ_0001023	mmu-miR-540-5p	ENSMUST00000108783	ENSMUSG00000000126
novel_circ_0001023	mmu-miR-540-5p	ENSMUST00000000128	ENSMUSG00000000126
novel_circ_0001023	mmu-miR-540-5p	ENSMUST00000108783	ENSMUSG00000000126
novel_circ_0001236	mmu-miR-1247-3	ENSMUST00000213072	ENSMUSG00000031849
novel_circ_0001292	mmu-miR-1247-3	ENSMUST00000213072	ENSMUSG00000031849
mmu_circ_0000042	mmu-miR-1247-3	ENSMUST00000213072	ENSMUSG00000031849
mmu_circ_0001021	mmu-miR-1247-3	ENSMUST00000213072	ENSMUSG00000031849
mmu_circ_0001376	mmu-miR-1247-3	ENSMUST00000213072	ENSMUSG00000031849
mmu_circ_0001271	mmu-miR-1247-3	ENSMUST00000213072	ENSMUSG00000031849
mmu_circ_0001213	mmu-miR-1247-3	ENSMUST00000213072	ENSMUSG00000031849
mmu_circ_0001213	mmu-miR-1247-3	ENSMUST00000213072	ENSMUSG00000031849

mmu_circ_0001499	mmu-miR-1247-3	ENSMUST00000213072	ENSMUSG00000031849
mmu_circ_0000277	mmu-miR-1247-3	ENSMUST00000213072	ENSMUSG00000031849
mmu_circ_0000172	mmu-miR-1247-3	ENSMUST00000213072	ENSMUSG00000031849
mmu_circ_0000454	mmu-miR-1247-3	ENSMUST00000213072	ENSMUSG00000031849
mmu_circ_0000384	mmu-miR-1247-3	ENSMUST00000213072	ENSMUSG00000031849
mmu_circ_0000530	mmu-miR-1247-3	ENSMUST00000213072	ENSMUSG00000031849
mmu_circ_0000555	mmu-miR-1247-3	ENSMUST00000213072	ENSMUSG00000031849
mmu_circ_0000555	mmu-miR-1247-3	ENSMUST00000213072	ENSMUSG00000031849
mmu_circ_0000829	mmu-miR-1247-3	ENSMUST00000213072	ENSMUSG00000031849
mmu_circ_0000705	mmu-miR-1247-3	ENSMUST00000213072	ENSMUSG00000031849
mmu_circ_0000884	mmu-miR-1247-3	ENSMUST00000213072	ENSMUSG00000031849
mmu_circ_0000886	mmu-miR-1247-3	ENSMUST00000213072	ENSMUSG00000031849
mmu_circ_0000887	mmu-miR-1247-3	ENSMUST00000213072	ENSMUSG00000031849
mmu_circ_0000898	mmu-miR-1247-3	ENSMUST00000213072	ENSMUSG00000031849
mmu_circ_0000905	mmu-miR-1247-3	ENSMUST00000213072	ENSMUSG00000031849
mmu_circ_0000912	mmu-miR-1247-3	ENSMUST00000213072	ENSMUSG00000031849
novel_circ_0001642	mmu-miR-1247-3	ENSMUST00000213072	ENSMUSG00000031849
novel_circ_0001710	mmu-miR-1247-3	ENSMUST00000213072	ENSMUSG00000031849
novel_circ_0001728	mmu-miR-1247-3	ENSMUST00000213072	ENSMUSG00000031849
novel_circ_0002126	mmu-miR-1247-3	ENSMUST00000213072	ENSMUSG00000031849
novel_circ_0002129	mmu-miR-1247-3	ENSMUST00000213072	ENSMUSG00000031849
novel_circ_0001761	mmu-miR-1247-3	ENSMUST00000213072	ENSMUSG00000031849
novel_circ_0001903	mmu-miR-1247-3	ENSMUST00000213072	ENSMUSG00000031849
novel_circ_0002353	mmu-miR-1247-3	ENSMUST00000213072	ENSMUSG00000031849
novel_circ_0002353	mmu-miR-1247-3	ENSMUST00000213072	ENSMUSG00000031849
novel_circ_0002393	mmu-miR-1247-3	ENSMUST00000213072	ENSMUSG00000031849
novel_circ_0002872	mmu-miR-1247-3	ENSMUST00000213072	ENSMUSG00000031849
novel_circ_0002873	mmu-miR-1247-3	ENSMUST00000213072	ENSMUSG00000031849
novel_circ_0002874	mmu-miR-1247-3	ENSMUST00000213072	ENSMUSG00000031849
novel_circ_0002875	mmu-miR-1247-3	ENSMUST00000213072	ENSMUSG00000031849
novel_circ_0003312	mmu-miR-1247-3	ENSMUST00000213072	ENSMUSG00000031849
novel_circ_0003313	mmu-miR-1247-3	ENSMUST00000213072	ENSMUSG00000031849
novel_circ_0000013	mmu-miR-1247-3	ENSMUST00000213072	ENSMUSG00000031849
novel_circ_0000103	mmu-miR-1247-3	ENSMUST00000213072	ENSMUSG00000031849
novel_circ_0000127	mmu-miR-1247-3	ENSMUST00000213072	ENSMUSG00000031849
novel_circ_0000620	mmu-miR-1247-3	ENSMUST00000213072	ENSMUSG00000031849
novel_circ_0001194	mmu-miR-1247-3	ENSMUST00000213072	ENSMUSG00000031849
novel_circ_0001224	mmu-miR-667-5p	ENSMUST00000000128	ENSMUSG00000000126
novel_circ_0001224	mmu-miR-667-5p	ENSMUST00000108783	ENSMUSG00000000126
novel_circ_0001236	mmu-miR-667-5p	ENSMUST00000000128	ENSMUSG00000000126
novel_circ_0001236	mmu-miR-667-5p	ENSMUST00000108783	ENSMUSG00000000126
novel_circ_0001250	mmu-miR-667-5p	ENSMUST00000000128	ENSMUSG00000000126
novel_circ_0001250	mmu-miR-667-5p	ENSMUST00000108783	ENSMUSG00000000126
novel_circ_0001270	mmu-miR-667-5p	ENSMUST00000000128	ENSMUSG00000000126
novel_circ_0001270	mmu-miR-667-5p	ENSMUST00000108783	ENSMUSG00000000126
novel_circ_0001290	mmu-miR-667-5p	ENSMUST00000000128	ENSMUSG00000000126
novel_circ_0001290	mmu-miR-667-5p	ENSMUST00000108783	ENSMUSG00000000126
novel_circ_0001306	mmu-miR-667-5p	ENSMUST00000000128	ENSMUSG00000000126
novel_circ_0001306	mmu-miR-667-5p	ENSMUST00000108783	ENSMUSG00000000126
novel_circ_0001070	mmu-miR-667-5p	ENSMUST00000000128	ENSMUSG00000000126
novel_circ_0001070	mmu-miR-667-5p	ENSMUST00000108783	ENSMUSG00000000126
novel_circ_0001076	mmu-miR-667-5p	ENSMUST00000000128	ENSMUSG00000000126
novel_circ_0001076	mmu-miR-667-5p	ENSMUST00000108783	ENSMUSG00000000126
novel_circ_0001106	mmu-miR-667-5p	ENSMUST00000000128	ENSMUSG00000000126
novel_circ_0001106	mmu-miR-667-5p	ENSMUST00000108783	ENSMUSG00000000126
novel_circ_0001438	mmu-miR-667-5p	ENSMUST00000000128	ENSMUSG00000000126
novel_circ_0001438	mmu-miR-667-5p	ENSMUST00000108783	ENSMUSG00000000126
novel_circ_0001505	mmu-miR-667-5p	ENSMUST00000000128	ENSMUSG00000000126

novel_circ_0001505	mmu-miR-667-5p	ENSMUST00000108783	ENSMUSG00000000126
novel_circ_0001349	mmu-miR-667-5p	ENSMUST00000000128	ENSMUSG00000000126
novel_circ_0001349	mmu-miR-667-5p	ENSMUST00000108783	ENSMUSG00000000126
novel_circ_0001385	mmu-miR-667-5p	ENSMUST00000000128	ENSMUSG00000000126
novel_circ_0001385	mmu-miR-667-5p	ENSMUST00000108783	ENSMUSG00000000126
novel_circ_0001399	mmu-miR-667-5p	ENSMUST00000000128	ENSMUSG00000000126
novel_circ_0001399	mmu-miR-667-5p	ENSMUST00000108783	ENSMUSG00000000126
novel_circ_0001401	mmu-miR-667-5p	ENSMUST00000000128	ENSMUSG00000000126
novel_circ_0001401	mmu-miR-667-5p	ENSMUST00000108783	ENSMUSG00000000126
novel_circ_0001402	mmu-miR-667-5p	ENSMUST00000000128	ENSMUSG00000000126
novel_circ_0001402	mmu-miR-667-5p	ENSMUST00000108783	ENSMUSG00000000126
novel_circ_0001414	mmu-miR-667-5p	ENSMUST00000000128	ENSMUSG00000000126
novel_circ_0001414	mmu-miR-667-5p	ENSMUST00000108783	ENSMUSG00000000126
mmu_circ_0000075	mmu-miR-667-5p	ENSMUST00000000128	ENSMUSG00000000126
mmu_circ_0000075	mmu-miR-667-5p	ENSMUST00000108783	ENSMUSG00000000126
mmu_circ_0000094	mmu-miR-667-5p	ENSMUST00000000128	ENSMUSG00000000126
mmu_circ_0000094	mmu-miR-667-5p	ENSMUST00000108783	ENSMUSG00000000126
mmu_circ_0000028	mmu-miR-667-5p	ENSMUST00000000128	ENSMUSG00000000126
mmu_circ_0000028	mmu-miR-667-5p	ENSMUST00000108783	ENSMUSG00000000126
mmu_circ_0000046	mmu-miR-667-5p	ENSMUST00000000128	ENSMUSG00000000126
mmu_circ_0000046	mmu-miR-667-5p	ENSMUST00000108783	ENSMUSG00000000126
mmu_circ_0000051	mmu-miR-667-5p	ENSMUST00000000128	ENSMUSG00000000126
mmu_circ_0000051	mmu-miR-667-5p	ENSMUST00000108783	ENSMUSG00000000126
mmu_circ_0000058	mmu-miR-667-5p	ENSMUST00000000128	ENSMUSG00000000126
mmu_circ_0000058	mmu-miR-667-5p	ENSMUST00000108783	ENSMUSG00000000126
mmu_circ_0000063	mmu-miR-667-5p	ENSMUST00000000128	ENSMUSG00000000126
mmu_circ_0000063	mmu-miR-667-5p	ENSMUST00000108783	ENSMUSG00000000126
mmu_circ_0001162	mmu-miR-667-5p	ENSMUST00000000128	ENSMUSG00000000126
mmu_circ_0001162	mmu-miR-667-5p	ENSMUST00000108783	ENSMUSG00000000126
mmu_circ_0001173	mmu-miR-667-5p	ENSMUST00000000128	ENSMUSG00000000126
mmu_circ_0001173	mmu-miR-667-5p	ENSMUST00000108783	ENSMUSG00000000126
mmu_circ_0001123	mmu-miR-667-5p	ENSMUST00000000128	ENSMUSG00000000126
mmu_circ_0001123	mmu-miR-667-5p	ENSMUST00000108783	ENSMUSG00000000126
mmu_circ_0001021	mmu-miR-667-5p	ENSMUST00000000128	ENSMUSG00000000126
mmu_circ_0001021	mmu-miR-667-5p	ENSMUST00000108783	ENSMUSG00000000126
mmu_circ_0001431	mmu-miR-667-5p	ENSMUST00000000128	ENSMUSG00000000126
mmu_circ_0001431	mmu-miR-667-5p	ENSMUST00000108783	ENSMUSG00000000126
mmu_circ_0001333	mmu-miR-667-5p	ENSMUST00000000128	ENSMUSG00000000126
mmu_circ_0001333	mmu-miR-667-5p	ENSMUST00000108783	ENSMUSG00000000126
mmu_circ_0001273	mmu-miR-667-5p	ENSMUST00000000128	ENSMUSG00000000126
mmu_circ_0001273	mmu-miR-667-5p	ENSMUST00000108783	ENSMUSG00000000126
mmu_circ_0001298	mmu-miR-667-5p	ENSMUST00000000128	ENSMUSG00000000126
mmu_circ_0001298	mmu-miR-667-5p	ENSMUST00000108783	ENSMUSG00000000126
mmu_circ_0001213	mmu-miR-667-5p	ENSMUST00000000128	ENSMUSG00000000126
mmu_circ_0001213	mmu-miR-667-5p	ENSMUST00000108783	ENSMUSG00000000126
mmu_circ_0001226	mmu-miR-667-5p	ENSMUST00000000128	ENSMUSG00000000126
mmu_circ_0001226	mmu-miR-667-5p	ENSMUST00000108783	ENSMUSG00000000126
mmu_circ_0001516	mmu-miR-667-5p	ENSMUST00000000128	ENSMUSG00000000126
mmu_circ_0001516	mmu-miR-667-5p	ENSMUST00000108783	ENSMUSG00000000126
mmu_circ_0001454	mmu-miR-667-5p	ENSMUST00000000128	ENSMUSG00000000126
mmu_circ_0001454	mmu-miR-667-5p	ENSMUST00000108783	ENSMUSG00000000126
mmu_circ_0001470	mmu-miR-667-5p	ENSMUST00000000128	ENSMUSG00000000126
mmu_circ_0001470	mmu-miR-667-5p	ENSMUST00000108783	ENSMUSG00000000126
mmu_circ_0001478	mmu-miR-667-5p	ENSMUST00000000128	ENSMUSG00000000126
mmu_circ_0001478	mmu-miR-667-5p	ENSMUST00000108783	ENSMUSG00000000126
mmu_circ_0001500	mmu-miR-667-5p	ENSMUST00000000128	ENSMUSG00000000126
mmu_circ_0001500	mmu-miR-667-5p	ENSMUST00000108783	ENSMUSG00000000126
mmu_circ_0001755	mmu-miR-667-5p	ENSMUST00000000128	ENSMUSG00000000126

mmu_circ_0001755	mmu-miR-667-5p	ENSMUST00000108783	ENSMUSG00000000126
mmu_circ_0001827	mmu-miR-667-5p	ENSMUST00000000128	ENSMUSG00000000126
mmu_circ_0001827	mmu-miR-667-5p	ENSMUST00000108783	ENSMUSG00000000126
mmu_circ_0001705	mmu-miR-667-5p	ENSMUST00000000128	ENSMUSG00000000126
mmu_circ_0001705	mmu-miR-667-5p	ENSMUST00000108783	ENSMUSG00000000126
mmu_circ_0000284	mmu-miR-667-5p	ENSMUST00000000128	ENSMUSG00000000126
mmu_circ_0000284	mmu-miR-667-5p	ENSMUST00000108783	ENSMUSG00000000126
mmu_circ_0000284	mmu-miR-667-5p	ENSMUST00000000128	ENSMUSG00000000126
mmu_circ_0000284	mmu-miR-667-5p	ENSMUST00000108783	ENSMUSG00000000126
mmu_circ_0000163	mmu-miR-667-5p	ENSMUST00000000128	ENSMUSG00000000126
mmu_circ_0000163	mmu-miR-667-5p	ENSMUST00000108783	ENSMUSG00000000126
mmu_circ_0000174	mmu-miR-667-5p	ENSMUST00000000128	ENSMUSG00000000126
mmu_circ_0000174	mmu-miR-667-5p	ENSMUST00000108783	ENSMUSG00000000126
mmu_circ_0000174	mmu-miR-667-5p	ENSMUST00000000128	ENSMUSG00000000126
mmu_circ_0000174	mmu-miR-667-5p	ENSMUST00000108783	ENSMUSG00000000126
mmu_circ_0000141	mmu-miR-667-5p	ENSMUST00000000128	ENSMUSG00000000126
mmu_circ_0000141	mmu-miR-667-5p	ENSMUST00000108783	ENSMUSG00000000126
mmu_circ_0000499	mmu-miR-667-5p	ENSMUST00000000128	ENSMUSG00000000126
mmu_circ_0000499	mmu-miR-667-5p	ENSMUST00000108783	ENSMUSG00000000126
mmu_circ_0000454	mmu-miR-667-5p	ENSMUST00000000128	ENSMUSG00000000126
mmu_circ_0000454	mmu-miR-667-5p	ENSMUST00000108783	ENSMUSG00000000126
mmu_circ_0000425	mmu-miR-667-5p	ENSMUST00000000128	ENSMUSG00000000126
mmu_circ_0000425	mmu-miR-667-5p	ENSMUST00000108783	ENSMUSG00000000126
mmu_circ_0000349	mmu-miR-667-5p	ENSMUST00000000128	ENSMUSG00000000126
mmu_circ_0000349	mmu-miR-667-5p	ENSMUST00000108783	ENSMUSG00000000126
mmu_circ_0000371	mmu-miR-667-5p	ENSMUST00000000128	ENSMUSG00000000126
mmu_circ_0000371	mmu-miR-667-5p	ENSMUST00000108783	ENSMUSG00000000126
mmu_circ_0000372	mmu-miR-667-5p	ENSMUST00000000128	ENSMUSG00000000126
mmu_circ_0000372	mmu-miR-667-5p	ENSMUST00000108783	ENSMUSG00000000126
mmu_circ_0000373	mmu-miR-667-5p	ENSMUST00000000128	ENSMUSG00000000126
mmu_circ_0000373	mmu-miR-667-5p	ENSMUST00000108783	ENSMUSG00000000126
mmu_circ_0000402	mmu-miR-667-5p	ENSMUST00000000128	ENSMUSG00000000126
mmu_circ_0000402	mmu-miR-667-5p	ENSMUST00000108783	ENSMUSG00000000126
mmu_circ_0000628	mmu-miR-667-5p	ENSMUST00000000128	ENSMUSG00000000126
mmu_circ_0000628	mmu-miR-667-5p	ENSMUST00000108783	ENSMUSG00000000126
mmu_circ_0000507	mmu-miR-667-5p	ENSMUST00000000128	ENSMUSG00000000126
mmu_circ_0000507	mmu-miR-667-5p	ENSMUST00000108783	ENSMUSG00000000126
mmu_circ_0000530	mmu-miR-667-5p	ENSMUST00000000128	ENSMUSG00000000126
mmu_circ_0000530	mmu-miR-667-5p	ENSMUST00000108783	ENSMUSG00000000126
mmu_circ_0000561	mmu-miR-667-5p	ENSMUST00000000128	ENSMUSG00000000126
mmu_circ_0000561	mmu-miR-667-5p	ENSMUST00000108783	ENSMUSG00000000126
mmu_circ_0000729	mmu-miR-667-5p	ENSMUST00000000128	ENSMUSG00000000126
mmu_circ_0000729	mmu-miR-667-5p	ENSMUST00000108783	ENSMUSG00000000126
mmu_circ_0000805	mmu-miR-667-5p	ENSMUST00000000128	ENSMUSG00000000126
mmu_circ_0000805	mmu-miR-667-5p	ENSMUST00000108783	ENSMUSG00000000126
mmu_circ_0000833	mmu-miR-667-5p	ENSMUST00000000128	ENSMUSG00000000126
mmu_circ_0000833	mmu-miR-667-5p	ENSMUST00000108783	ENSMUSG00000000126
mmu_circ_0000668	mmu-miR-667-5p	ENSMUST00000000128	ENSMUSG00000000126
mmu_circ_0000668	mmu-miR-667-5p	ENSMUST00000108783	ENSMUSG00000000126
mmu_circ_0000705	mmu-miR-667-5p	ENSMUST00000000128	ENSMUSG00000000126
mmu_circ_0000705	mmu-miR-667-5p	ENSMUST00000108783	ENSMUSG00000000126
mmu_circ_0000705	mmu-miR-667-5p	ENSMUST00000000128	ENSMUSG00000000126
mmu_circ_0000705	mmu-miR-667-5p	ENSMUST00000108783	ENSMUSG00000000126
mmu_circ_0000712	mmu-miR-667-5p	ENSMUST00000000128	ENSMUSG00000000126
mmu_circ_0000712	mmu-miR-667-5p	ENSMUST00000108783	ENSMUSG00000000126
mmu_circ_0000714	mmu-miR-667-5p	ENSMUST00000000128	ENSMUSG00000000126
mmu_circ_0000714	mmu-miR-667-5p	ENSMUST00000108783	ENSMUSG00000000126
mmu_circ_0000955	mmu-miR-667-5p	ENSMUST00000000128	ENSMUSG00000000126

mmu_circ_0000955	mmu-miR-667-5p	ENSMUST00000108783	ENSMUSG00000000126
mmu_circ_0000956	mmu-miR-667-5p	ENSMUST00000000128	ENSMUSG00000000126
mmu_circ_0000956	mmu-miR-667-5p	ENSMUST00000108783	ENSMUSG00000000126
mmu_circ_0000854	mmu-miR-667-5p	ENSMUST00000000128	ENSMUSG00000000126
mmu_circ_0000854	mmu-miR-667-5p	ENSMUST00000108783	ENSMUSG00000000126
mmu_circ_0000874	mmu-miR-667-5p	ENSMUST00000000128	ENSMUSG00000000126
mmu_circ_0000874	mmu-miR-667-5p	ENSMUST00000108783	ENSMUSG00000000126
mmu_circ_0000884	mmu-miR-667-5p	ENSMUST00000000128	ENSMUSG00000000126
mmu_circ_0000884	mmu-miR-667-5p	ENSMUST00000108783	ENSMUSG00000000126
mmu_circ_0000886	mmu-miR-667-5p	ENSMUST00000000128	ENSMUSG00000000126
mmu_circ_0000886	mmu-miR-667-5p	ENSMUST00000108783	ENSMUSG00000000126
mmu_circ_0000905	mmu-miR-667-5p	ENSMUST00000000128	ENSMUSG00000000126
mmu_circ_0000905	mmu-miR-667-5p	ENSMUST00000108783	ENSMUSG00000000126
novel_circ_0001561	mmu-miR-667-5p	ENSMUST00000000128	ENSMUSG00000000126
novel_circ_0001561	mmu-miR-667-5p	ENSMUST00000108783	ENSMUSG00000000126
novel_circ_0001576	mmu-miR-667-5p	ENSMUST00000000128	ENSMUSG00000000126
novel_circ_0001576	mmu-miR-667-5p	ENSMUST00000108783	ENSMUSG00000000126
novel_circ_0001708	mmu-miR-667-5p	ENSMUST00000000128	ENSMUSG00000000126
novel_circ_0001708	mmu-miR-667-5p	ENSMUST00000108783	ENSMUSG00000000126
novel_circ_0001721	mmu-miR-667-5p	ENSMUST00000000128	ENSMUSG00000000126
novel_circ_0001721	mmu-miR-667-5p	ENSMUST00000108783	ENSMUSG00000000126
novel_circ_0002082	mmu-miR-667-5p	ENSMUST00000000128	ENSMUSG00000000126
novel_circ_0002082	mmu-miR-667-5p	ENSMUST00000108783	ENSMUSG00000000126
novel_circ_0002097	mmu-miR-667-5p	ENSMUST00000000128	ENSMUSG00000000126
novel_circ_0002097	mmu-miR-667-5p	ENSMUST00000108783	ENSMUSG00000000126
novel_circ_0002111	mmu-miR-667-5p	ENSMUST00000000128	ENSMUSG00000000126
novel_circ_0002111	mmu-miR-667-5p	ENSMUST00000108783	ENSMUSG00000000126
novel_circ_0002114	mmu-miR-667-5p	ENSMUST00000000128	ENSMUSG00000000126
novel_circ_0002114	mmu-miR-667-5p	ENSMUST00000108783	ENSMUSG00000000126
novel_circ_0002123	mmu-miR-667-5p	ENSMUST00000000128	ENSMUSG00000000126
novel_circ_0002123	mmu-miR-667-5p	ENSMUST00000108783	ENSMUSG00000000126
novel_circ_0002129	mmu-miR-667-5p	ENSMUST00000000128	ENSMUSG00000000126
novel_circ_0002129	mmu-miR-667-5p	ENSMUST00000108783	ENSMUSG00000000126
novel_circ_0002180	mmu-miR-667-5p	ENSMUST00000000128	ENSMUSG00000000126
novel_circ_0002180	mmu-miR-667-5p	ENSMUST00000108783	ENSMUSG00000000126
novel_circ_0001763	mmu-miR-667-5p	ENSMUST00000000128	ENSMUSG00000000126
novel_circ_0001763	mmu-miR-667-5p	ENSMUST00000108783	ENSMUSG00000000126
novel_circ_0001842	mmu-miR-667-5p	ENSMUST00000000128	ENSMUSG00000000126
novel_circ_0001842	mmu-miR-667-5p	ENSMUST00000108783	ENSMUSG00000000126
novel_circ_0001844	mmu-miR-667-5p	ENSMUST00000000128	ENSMUSG00000000126
novel_circ_0001844	mmu-miR-667-5p	ENSMUST00000108783	ENSMUSG00000000126
novel_circ_0001847	mmu-miR-667-5p	ENSMUST00000000128	ENSMUSG00000000126
novel_circ_0001847	mmu-miR-667-5p	ENSMUST00000108783	ENSMUSG00000000126
novel_circ_0001847	mmu-miR-667-5p	ENSMUST00000000128	ENSMUSG00000000126
novel_circ_0001847	mmu-miR-667-5p	ENSMUST00000108783	ENSMUSG00000000126
novel_circ_0001848	mmu-miR-667-5p	ENSMUST00000000128	ENSMUSG00000000126
novel_circ_0001848	mmu-miR-667-5p	ENSMUST00000108783	ENSMUSG00000000126
novel_circ_0001848	mmu-miR-667-5p	ENSMUST00000000128	ENSMUSG00000000126
novel_circ_0001848	mmu-miR-667-5p	ENSMUST00000108783	ENSMUSG00000000126
novel_circ_0001899	mmu-miR-667-5p	ENSMUST00000000128	ENSMUSG00000000126
novel_circ_0001899	mmu-miR-667-5p	ENSMUST00000108783	ENSMUSG00000000126
novel_circ_0001899	mmu-miR-667-5p	ENSMUST00000000128	ENSMUSG00000000126
novel_circ_0001899	mmu-miR-667-5p	ENSMUST00000108783	ENSMUSG00000000126
novel_circ_0001913	mmu-miR-667-5p	ENSMUST00000000128	ENSMUSG00000000126
novel_circ_0001913	mmu-miR-667-5p	ENSMUST00000108783	ENSMUSG00000000126
novel_circ_0001916	mmu-miR-667-5p	ENSMUST00000000128	ENSMUSG00000000126
novel_circ_0001916	mmu-miR-667-5p	ENSMUST00000108783	ENSMUSG00000000126
novel_circ_0002023	mmu-miR-667-5p	ENSMUST00000000128	ENSMUSG00000000126

novel_circ_0002023	mmu-miR-667-5p	ENSMUST00000108783	ENSMUSG00000000126
novel_circ_0002408	mmu-miR-667-5p	ENSMUST00000000128	ENSMUSG00000000126
novel_circ_0002408	mmu-miR-667-5p	ENSMUST00000108783	ENSMUSG00000000126
novel_circ_0002413	mmu-miR-667-5p	ENSMUST00000000128	ENSMUSG00000000126
novel_circ_0002413	mmu-miR-667-5p	ENSMUST00000108783	ENSMUSG00000000126
novel_circ_0002450	mmu-miR-667-5p	ENSMUST00000000128	ENSMUSG00000000126
novel_circ_0002450	mmu-miR-667-5p	ENSMUST00000108783	ENSMUSG00000000126
novel_circ_0002451	mmu-miR-667-5p	ENSMUST00000000128	ENSMUSG00000000126
novel_circ_0002451	mmu-miR-667-5p	ENSMUST00000108783	ENSMUSG00000000126
novel_circ_0002452	mmu-miR-667-5p	ENSMUST00000000128	ENSMUSG00000000126
novel_circ_0002452	mmu-miR-667-5p	ENSMUST00000108783	ENSMUSG00000000126
novel_circ_0002515	mmu-miR-667-5p	ENSMUST00000000128	ENSMUSG00000000126
novel_circ_0002515	mmu-miR-667-5p	ENSMUST00000108783	ENSMUSG00000000126
novel_circ_0002518	mmu-miR-667-5p	ENSMUST00000000128	ENSMUSG00000000126
novel_circ_0002518	mmu-miR-667-5p	ENSMUST00000108783	ENSMUSG00000000126
novel_circ_0002519	mmu-miR-667-5p	ENSMUST00000000128	ENSMUSG00000000126
novel_circ_0002519	mmu-miR-667-5p	ENSMUST00000108783	ENSMUSG00000000126
novel_circ_0002554	mmu-miR-667-5p	ENSMUST00000000128	ENSMUSG00000000126
novel_circ_0002554	mmu-miR-667-5p	ENSMUST00000108783	ENSMUSG00000000126
novel_circ_0002578	mmu-miR-667-5p	ENSMUST00000000128	ENSMUSG00000000126
novel_circ_0002578	mmu-miR-667-5p	ENSMUST00000108783	ENSMUSG00000000126
novel_circ_0002578	mmu-miR-667-5p	ENSMUST00000000128	ENSMUSG00000000126
novel_circ_0002578	mmu-miR-667-5p	ENSMUST00000108783	ENSMUSG00000000126
novel_circ_0002248	mmu-miR-667-5p	ENSMUST00000000128	ENSMUSG00000000126
novel_circ_0002248	mmu-miR-667-5p	ENSMUST00000108783	ENSMUSG00000000126
novel_circ_0002252	mmu-miR-667-5p	ENSMUST00000000128	ENSMUSG00000000126
novel_circ_0002252	mmu-miR-667-5p	ENSMUST00000108783	ENSMUSG00000000126
novel_circ_0002267	mmu-miR-667-5p	ENSMUST00000000128	ENSMUSG00000000126
novel_circ_0002267	mmu-miR-667-5p	ENSMUST00000108783	ENSMUSG00000000126
novel_circ_0002303	mmu-miR-667-5p	ENSMUST00000000128	ENSMUSG00000000126
novel_circ_0002303	mmu-miR-667-5p	ENSMUST00000108783	ENSMUSG00000000126
novel_circ_0002323	mmu-miR-667-5p	ENSMUST00000000128	ENSMUSG00000000126
novel_circ_0002323	mmu-miR-667-5p	ENSMUST00000108783	ENSMUSG00000000126
novel_circ_0002356	mmu-miR-667-5p	ENSMUST00000000128	ENSMUSG00000000126
novel_circ_0002356	mmu-miR-667-5p	ENSMUST00000108783	ENSMUSG00000000126
novel_circ_0002927	mmu-miR-667-5p	ENSMUST00000000128	ENSMUSG00000000126
novel_circ_0002927	mmu-miR-667-5p	ENSMUST00000108783	ENSMUSG00000000126
novel_circ_0002943	mmu-miR-667-5p	ENSMUST00000000128	ENSMUSG00000000126
novel_circ_0002943	mmu-miR-667-5p	ENSMUST00000108783	ENSMUSG00000000126
novel_circ_0002951	mmu-miR-667-5p	ENSMUST00000000128	ENSMUSG00000000126
novel_circ_0002951	mmu-miR-667-5p	ENSMUST00000108783	ENSMUSG00000000126
novel_circ_0002967	mmu-miR-667-5p	ENSMUST00000000128	ENSMUSG00000000126
novel_circ_0002967	mmu-miR-667-5p	ENSMUST00000108783	ENSMUSG00000000126
novel_circ_0002631	mmu-miR-667-5p	ENSMUST00000000128	ENSMUSG00000000126
novel_circ_0002631	mmu-miR-667-5p	ENSMUST00000108783	ENSMUSG00000000126
novel_circ_0002642	mmu-miR-667-5p	ENSMUST00000000128	ENSMUSG00000000126
novel_circ_0002642	mmu-miR-667-5p	ENSMUST00000108783	ENSMUSG00000000126
novel_circ_0002656	mmu-miR-667-5p	ENSMUST00000000128	ENSMUSG00000000126
novel_circ_0002656	mmu-miR-667-5p	ENSMUST00000108783	ENSMUSG00000000126
novel_circ_0002657	mmu-miR-667-5p	ENSMUST00000000128	ENSMUSG00000000126
novel_circ_0002657	mmu-miR-667-5p	ENSMUST00000108783	ENSMUSG00000000126
novel_circ_0002658	mmu-miR-667-5p	ENSMUST00000000128	ENSMUSG00000000126
novel_circ_0002658	mmu-miR-667-5p	ENSMUST00000108783	ENSMUSG00000000126
novel_circ_0002674	mmu-miR-667-5p	ENSMUST00000000128	ENSMUSG00000000126
novel_circ_0002674	mmu-miR-667-5p	ENSMUST00000108783	ENSMUSG00000000126
novel_circ_0002693	mmu-miR-667-5p	ENSMUST00000000128	ENSMUSG00000000126
novel_circ_0002693	mmu-miR-667-5p	ENSMUST00000108783	ENSMUSG00000000126
novel_circ_0002732	mmu-miR-667-5p	ENSMUST00000000128	ENSMUSG00000000126

novel_circ_0002732	mmu-miR-667-5p	ENSMUST00000108783	ENSMUSG00000000126
novel_circ_0002745	mmu-miR-667-5p	ENSMUST00000000128	ENSMUSG00000000126
novel_circ_0002745	mmu-miR-667-5p	ENSMUST00000108783	ENSMUSG00000000126
novel_circ_0002752	mmu-miR-667-5p	ENSMUST00000000128	ENSMUSG00000000126
novel_circ_0002752	mmu-miR-667-5p	ENSMUST00000108783	ENSMUSG00000000126
novel_circ_0003228	mmu-miR-667-5p	ENSMUST00000000128	ENSMUSG00000000126
novel_circ_0003228	mmu-miR-667-5p	ENSMUST00000108783	ENSMUSG00000000126
novel_circ_0003249	mmu-miR-667-5p	ENSMUST00000000128	ENSMUSG00000000126
novel_circ_0003249	mmu-miR-667-5p	ENSMUST00000108783	ENSMUSG00000000126
novel_circ_0003358	mmu-miR-667-5p	ENSMUST00000000128	ENSMUSG00000000126
novel_circ_0003358	mmu-miR-667-5p	ENSMUST00000108783	ENSMUSG00000000126
novel_circ_0003051	mmu-miR-667-5p	ENSMUST00000000128	ENSMUSG00000000126
novel_circ_0003051	mmu-miR-667-5p	ENSMUST00000108783	ENSMUSG00000000126
novel_circ_0003078	mmu-miR-667-5p	ENSMUST00000000128	ENSMUSG00000000126
novel_circ_0003078	mmu-miR-667-5p	ENSMUST00000108783	ENSMUSG00000000126
novel_circ_0003078	mmu-miR-667-5p	ENSMUST00000000128	ENSMUSG00000000126
novel_circ_0003078	mmu-miR-667-5p	ENSMUST00000108783	ENSMUSG00000000126
novel_circ_0003164	mmu-miR-667-5p	ENSMUST00000000128	ENSMUSG00000000126
novel_circ_0003164	mmu-miR-667-5p	ENSMUST00000108783	ENSMUSG00000000126
novel_circ_0003455	mmu-miR-667-5p	ENSMUST00000000128	ENSMUSG00000000126
novel_circ_0003455	mmu-miR-667-5p	ENSMUST00000108783	ENSMUSG00000000126
novel_circ_0003484	mmu-miR-667-5p	ENSMUST00000000128	ENSMUSG00000000126
novel_circ_0003484	mmu-miR-667-5p	ENSMUST00000108783	ENSMUSG00000000126
novel_circ_0000170	mmu-miR-667-5p	ENSMUST00000000128	ENSMUSG00000000126
novel_circ_0000170	mmu-miR-667-5p	ENSMUST00000108783	ENSMUSG00000000126
novel_circ_0000170	mmu-miR-667-5p	ENSMUST00000000128	ENSMUSG00000000126
novel_circ_0000170	mmu-miR-667-5p	ENSMUST00000108783	ENSMUSG00000000126
novel_circ_0000262	mmu-miR-667-5p	ENSMUST00000000128	ENSMUSG00000000126
novel_circ_0000262	mmu-miR-667-5p	ENSMUST00000108783	ENSMUSG00000000126
novel_circ_0000319	mmu-miR-667-5p	ENSMUST00000000128	ENSMUSG00000000126
novel_circ_0000319	mmu-miR-667-5p	ENSMUST00000108783	ENSMUSG00000000126
novel_circ_0000413	mmu-miR-667-5p	ENSMUST00000000128	ENSMUSG00000000126
novel_circ_0000413	mmu-miR-667-5p	ENSMUST00000108783	ENSMUSG00000000126
novel_circ_0000441	mmu-miR-667-5p	ENSMUST00000000128	ENSMUSG00000000126
novel_circ_0000441	mmu-miR-667-5p	ENSMUST00000108783	ENSMUSG00000000126
novel_circ_0000444	mmu-miR-667-5p	ENSMUST00000000128	ENSMUSG00000000126
novel_circ_0000444	mmu-miR-667-5p	ENSMUST00000108783	ENSMUSG00000000126
novel_circ_0000008	mmu-miR-667-5p	ENSMUST00000000128	ENSMUSG00000000126
novel_circ_0000008	mmu-miR-667-5p	ENSMUST00000108783	ENSMUSG00000000126
novel_circ_0000017	mmu-miR-667-5p	ENSMUST00000000128	ENSMUSG00000000126
novel_circ_0000017	mmu-miR-667-5p	ENSMUST00000108783	ENSMUSG00000000126
novel_circ_0000068	mmu-miR-667-5p	ENSMUST00000000128	ENSMUSG00000000126
novel_circ_0000068	mmu-miR-667-5p	ENSMUST00000108783	ENSMUSG00000000126
novel_circ_0000125	mmu-miR-667-5p	ENSMUST00000000128	ENSMUSG00000000126
novel_circ_0000125	mmu-miR-667-5p	ENSMUST00000108783	ENSMUSG00000000126
novel_circ_0000145	mmu-miR-667-5p	ENSMUST00000000128	ENSMUSG00000000126
novel_circ_0000145	mmu-miR-667-5p	ENSMUST00000108783	ENSMUSG00000000126
novel_circ_0000154	mmu-miR-667-5p	ENSMUST00000000128	ENSMUSG00000000126
novel_circ_0000154	mmu-miR-667-5p	ENSMUST00000108783	ENSMUSG00000000126
novel_circ_0000668	mmu-miR-667-5p	ENSMUST00000000128	ENSMUSG00000000126
novel_circ_0000668	mmu-miR-667-5p	ENSMUST00000108783	ENSMUSG00000000126
novel_circ_0000710	mmu-miR-667-5p	ENSMUST00000000128	ENSMUSG00000000126
novel_circ_0000710	mmu-miR-667-5p	ENSMUST00000108783	ENSMUSG00000000126
novel_circ_0000525	mmu-miR-667-5p	ENSMUST00000000128	ENSMUSG00000000126
novel_circ_0000525	mmu-miR-667-5p	ENSMUST00000108783	ENSMUSG00000000126
novel_circ_0000589	mmu-miR-667-5p	ENSMUST00000000128	ENSMUSG00000000126
novel_circ_0000589	mmu-miR-667-5p	ENSMUST00000108783	ENSMUSG00000000126
novel_circ_0000594	mmu-miR-667-5p	ENSMUST00000000128	ENSMUSG00000000126

novel_circ_0000594	mmu-miR-667-5p	ENSMUST00000108783	ENSMUSG00000000126
novel_circ_0000814	mmu-miR-667-5p	ENSMUST00000000128	ENSMUSG00000000126
novel_circ_0000814	mmu-miR-667-5p	ENSMUST00000108783	ENSMUSG00000000126
novel_circ_0000914	mmu-miR-667-5p	ENSMUST00000000128	ENSMUSG00000000126
novel_circ_0000914	mmu-miR-667-5p	ENSMUST00000108783	ENSMUSG00000000126
novel_circ_0001196	mmu-miR-667-5p	ENSMUST00000000128	ENSMUSG00000000126
novel_circ_0001196	mmu-miR-667-5p	ENSMUST00000108783	ENSMUSG00000000126
novel_circ_0001197	mmu-miR-667-5p	ENSMUST00000000128	ENSMUSG00000000126
novel_circ_0001197	mmu-miR-667-5p	ENSMUST00000108783	ENSMUSG00000000126
novel_circ_0001211	mmu-miR-667-5p	ENSMUST00000000128	ENSMUSG00000000126
novel_circ_0001211	mmu-miR-667-5p	ENSMUST00000108783	ENSMUSG00000000126
novel_circ_0001278	mmu-miR-3058-3	ENSMUST00000225594	ENSMUSG00000017491
novel_circ_0001280	mmu-miR-3058-3	ENSMUST00000225594	ENSMUSG00000017491
novel_circ_0001060	mmu-miR-3058-3	ENSMUST00000225594	ENSMUSG00000017491
novel_circ_0001110	mmu-miR-3058-3	ENSMUST00000225594	ENSMUSG00000017491
novel_circ_0001449	mmu-miR-3058-3	ENSMUST00000225594	ENSMUSG00000017491
novel_circ_0001461	mmu-miR-3058-3	ENSMUST00000225594	ENSMUSG00000017491
novel_circ_0001505	mmu-miR-3058-3	ENSMUST00000225594	ENSMUSG00000017491
novel_circ_0003422	mmu-miR-3058-3	ENSMUST00000225594	ENSMUSG00000017491
mmu_circ_0000057	mmu-miR-3058-3	ENSMUST00000225594	ENSMUSG00000017491
mmu_circ_0001148	mmu-miR-3058-3	ENSMUST00000225594	ENSMUSG00000017491
mmu_circ_0001017	mmu-miR-3058-3	ENSMUST00000225594	ENSMUSG00000017491
mmu_circ_0001331	mmu-miR-3058-3	ENSMUST00000225594	ENSMUSG00000017491
mmu_circ_0001636	mmu-miR-3058-3	ENSMUST00000225594	ENSMUSG00000017491
mmu_circ_0001580	mmu-miR-3058-3	ENSMUST00000225594	ENSMUSG00000017491
mmu_circ_0001598	mmu-miR-3058-3	ENSMUST00000225594	ENSMUSG00000017491
mmu_circ_0001454	mmu-miR-3058-3	ENSMUST00000225594	ENSMUSG00000017491
mmu_circ_0001442	mmu-miR-3058-3	ENSMUST00000225594	ENSMUSG00000017491
mmu_circ_0001779	mmu-miR-3058-3	ENSMUST00000225594	ENSMUSG00000017491
mmu_circ_0001827	mmu-miR-3058-3	ENSMUST00000225594	ENSMUSG00000017491
mmu_circ_0001718	mmu-miR-3058-3	ENSMUST00000225594	ENSMUSG00000017491
mmu_circ_0000333	mmu-miR-3058-3	ENSMUST00000225594	ENSMUSG00000017491
mmu_circ_0000243	mmu-miR-3058-3	ENSMUST00000225594	ENSMUSG00000017491
mmu_circ_0000277	mmu-miR-3058-3	ENSMUST00000225594	ENSMUSG00000017491
mmu_circ_0000287	mmu-miR-3058-3	ENSMUST00000225594	ENSMUSG00000017491
mmu_circ_0000500	mmu-miR-3058-3	ENSMUST00000225594	ENSMUSG00000017491
mmu_circ_0000454	mmu-miR-3058-3	ENSMUST00000225594	ENSMUSG00000017491
mmu_circ_0000465	mmu-miR-3058-3	ENSMUST00000225594	ENSMUSG00000017491
mmu_circ_0000635	mmu-miR-3058-3	ENSMUST00000225594	ENSMUSG00000017491
mmu_circ_0000522	mmu-miR-3058-3	ENSMUST00000225594	ENSMUSG00000017491
mmu_circ_0000526	mmu-miR-3058-3	ENSMUST00000225594	ENSMUSG00000017491
mmu_circ_0000547	mmu-miR-3058-3	ENSMUST00000225594	ENSMUSG00000017491
mmu_circ_0000735	mmu-miR-3058-3	ENSMUST00000225594	ENSMUSG00000017491
mmu_circ_0000798	mmu-miR-3058-3	ENSMUST00000225594	ENSMUSG00000017491
mmu_circ_0000829	mmu-miR-3058-3	ENSMUST00000225594	ENSMUSG00000017491
mmu_circ_0000831	mmu-miR-3058-3	ENSMUST00000225594	ENSMUSG00000017491
mmu_circ_0000711	mmu-miR-3058-3	ENSMUST00000225594	ENSMUSG00000017491
mmu_circ_0000712	mmu-miR-3058-3	ENSMUST00000225594	ENSMUSG00000017491
mmu_circ_0000713	mmu-miR-3058-3	ENSMUST00000225594	ENSMUSG00000017491
mmu_circ_0000714	mmu-miR-3058-3	ENSMUST00000225594	ENSMUSG00000017491
mmu_circ_0000955	mmu-miR-3058-3	ENSMUST00000225594	ENSMUSG00000017491
mmu_circ_0000956	mmu-miR-3058-3	ENSMUST00000225594	ENSMUSG00000017491
mmu_circ_0000956	mmu-miR-3058-3	ENSMUST00000225594	ENSMUSG00000017491
mmu_circ_0000969	mmu-miR-3058-3	ENSMUST00000225594	ENSMUSG00000017491
mmu_circ_0000884	mmu-miR-3058-3	ENSMUST00000225594	ENSMUSG00000017491
mmu_circ_0000886	mmu-miR-3058-3	ENSMUST00000225594	ENSMUSG00000017491
mmu_circ_0000912	mmu-miR-3058-3	ENSMUST00000225594	ENSMUSG00000017491
novel_circ_0001561	mmu-miR-3058-3	ENSMUST00000225594	ENSMUSG00000017491

novel_circ_0001625	mmu-miR-3058-3	ENSMUST00000225594	ENSMUSG00000017491
novel_circ_0001625	mmu-miR-3058-3	ENSMUST00000225594	ENSMUSG00000017491
novel_circ_0001672	mmu-miR-3058-3	ENSMUST00000225594	ENSMUSG00000017491
novel_circ_0001672	mmu-miR-3058-3	ENSMUST00000225594	ENSMUSG00000017491
novel_circ_0002106	mmu-miR-3058-3	ENSMUST00000225594	ENSMUSG00000017491
novel_circ_0002167	mmu-miR-3058-3	ENSMUST00000225594	ENSMUSG00000017491
novel_circ_0002167	mmu-miR-3058-3	ENSMUST00000225594	ENSMUSG00000017491
novel_circ_0002192	mmu-miR-3058-3	ENSMUST00000225594	ENSMUSG00000017491
novel_circ_0002215	mmu-miR-3058-3	ENSMUST00000225594	ENSMUSG00000017491
novel_circ_0001756	mmu-miR-3058-3	ENSMUST00000225594	ENSMUSG00000017491
novel_circ_0001891	mmu-miR-3058-3	ENSMUST00000225594	ENSMUSG00000017491
novel_circ_0001910	mmu-miR-3058-3	ENSMUST00000225594	ENSMUSG00000017491
novel_circ_0001940	mmu-miR-3058-3	ENSMUST00000225594	ENSMUSG00000017491
novel_circ_0001994	mmu-miR-3058-3	ENSMUST00000225594	ENSMUSG00000017491
novel_circ_0002031	mmu-miR-3058-3	ENSMUST00000225594	ENSMUSG00000017491
novel_circ_0002035	mmu-miR-3058-3	ENSMUST00000225594	ENSMUSG00000017491
novel_circ_0002488	mmu-miR-3058-3	ENSMUST00000225594	ENSMUSG00000017491
novel_circ_0002518	mmu-miR-3058-3	ENSMUST00000225594	ENSMUSG00000017491
novel_circ_0002554	mmu-miR-3058-3	ENSMUST00000225594	ENSMUSG00000017491
novel_circ_0002554	mmu-miR-3058-3	ENSMUST00000225594	ENSMUSG00000017491
novel_circ_0002288	mmu-miR-3058-3	ENSMUST00000225594	ENSMUSG00000017491
novel_circ_0002292	mmu-miR-3058-3	ENSMUST00000225594	ENSMUSG00000017491
novel_circ_0002332	mmu-miR-3058-3	ENSMUST00000225594	ENSMUSG00000017491
novel_circ_0002868	mmu-miR-3058-3	ENSMUST00000225594	ENSMUSG00000017491
novel_circ_0002882	mmu-miR-3058-3	ENSMUST00000225594	ENSMUSG00000017491
novel_circ_0002882	mmu-miR-3058-3	ENSMUST00000225594	ENSMUSG00000017491
novel_circ_0002973	mmu-miR-3058-3	ENSMUST00000225594	ENSMUSG00000017491
novel_circ_0002973	mmu-miR-3058-3	ENSMUST00000225594	ENSMUSG00000017491
novel_circ_0002682	mmu-miR-3058-3	ENSMUST00000225594	ENSMUSG00000017491
novel_circ_0002700	mmu-miR-3058-3	ENSMUST00000225594	ENSMUSG00000017491
novel_circ_0002769	mmu-miR-3058-3	ENSMUST00000225594	ENSMUSG00000017491
novel_circ_0002788	mmu-miR-3058-3	ENSMUST00000225594	ENSMUSG00000017491
novel_circ_0002812	mmu-miR-3058-3	ENSMUST00000225594	ENSMUSG00000017491
novel_circ_0002812	mmu-miR-3058-3	ENSMUST00000225594	ENSMUSG00000017491
novel_circ_0003273	mmu-miR-3058-3	ENSMUST00000225594	ENSMUSG00000017491
novel_circ_0003273	mmu-miR-3058-3	ENSMUST00000225594	ENSMUSG00000017491
novel_circ_0003420	mmu-miR-3058-3	ENSMUST00000225594	ENSMUSG00000017491
novel_circ_0003054	mmu-miR-3058-3	ENSMUST00000225594	ENSMUSG00000017491
novel_circ_0003124	mmu-miR-3058-3	ENSMUST00000225594	ENSMUSG00000017491
novel_circ_0003483	mmu-miR-3058-3	ENSMUST00000225594	ENSMUSG00000017491
novel_circ_0000190	mmu-miR-3058-3	ENSMUST00000225594	ENSMUSG00000017491
novel_circ_0000193	mmu-miR-3058-3	ENSMUST00000225594	ENSMUSG00000017491
novel_circ_0000208	mmu-miR-3058-3	ENSMUST00000225594	ENSMUSG00000017491
novel_circ_0000218	mmu-miR-3058-3	ENSMUST00000225594	ENSMUSG00000017491
novel_circ_0000298	mmu-miR-3058-3	ENSMUST00000225594	ENSMUSG00000017491
novel_circ_0000312	mmu-miR-3058-3	ENSMUST00000225594	ENSMUSG00000017491
novel_circ_0000069	mmu-miR-3058-3	ENSMUST00000225594	ENSMUSG00000017491
novel_circ_0000677	mmu-miR-3058-3	ENSMUST00000225594	ENSMUSG00000017491
novel_circ_0000677	mmu-miR-3058-3	ENSMUST00000225594	ENSMUSG00000017491
novel_circ_0000692	mmu-miR-3058-3	ENSMUST00000225594	ENSMUSG00000017491
novel_circ_0000743	mmu-miR-3058-3	ENSMUST00000225594	ENSMUSG00000017491
novel_circ_0000769	mmu-miR-3058-3	ENSMUST00000225594	ENSMUSG00000017491
novel_circ_0000769	mmu-miR-3058-3	ENSMUST00000225594	ENSMUSG00000017491
novel_circ_0000534	mmu-miR-3058-3	ENSMUST00000225594	ENSMUSG00000017491
novel_circ_0000985	mmu-miR-3058-3	ENSMUST00000225594	ENSMUSG00000017491
novel_circ_0000899	mmu-miR-3058-3	ENSMUST00000225594	ENSMUSG00000017491
novel_circ_0001177	mmu-miR-3058-3	ENSMUST00000225594	ENSMUSG00000017491
novel_circ_0001179	mmu-miR-3058-3	ENSMUST00000225594	ENSMUSG00000017491

novel_circ_0001222	mmu-miR-297b-5	ENSMUST00000110698	ENSMUSG00000055737
novel_circ_0001306	mmu-miR-297b-5	ENSMUST00000110698	ENSMUSG00000055737
novel_circ_0001346	mmu-miR-297b-5	ENSMUST00000110698	ENSMUSG00000055737
mmu_circ_0001162	mmu-miR-297b-5	ENSMUST00000110698	ENSMUSG00000055737
mmu_circ_0001123	mmu-miR-297b-5	ENSMUST00000110698	ENSMUSG00000055737
mmu_circ_0001136	mmu-miR-297b-5	ENSMUST00000110698	ENSMUSG00000055737
mmu_circ_0001376	mmu-miR-297b-5	ENSMUST00000110698	ENSMUSG00000055737
mmu_circ_0001333	mmu-miR-297b-5	ENSMUST00000110698	ENSMUSG00000055737
mmu_circ_0001335	mmu-miR-297b-5	ENSMUST00000110698	ENSMUSG00000055737
mmu_circ_0001336	mmu-miR-297b-5	ENSMUST00000110698	ENSMUSG00000055737
mmu_circ_0001507	mmu-miR-297b-5	ENSMUST00000110698	ENSMUSG00000055737
mmu_circ_0001454	mmu-miR-297b-5	ENSMUST00000110698	ENSMUSG00000055737
mmu_circ_0001755	mmu-miR-297b-5	ENSMUST00000110698	ENSMUSG00000055737
mmu_circ_0001755	mmu-miR-297b-5	ENSMUST00000110698	ENSMUSG00000055737
mmu_circ_0001827	mmu-miR-297b-5	ENSMUST00000110698	ENSMUSG00000055737
mmu_circ_0001886	mmu-miR-297b-5	ENSMUST00000110698	ENSMUSG00000055737
mmu_circ_0000271	mmu-miR-297b-5	ENSMUST00000110698	ENSMUSG00000055737
mmu_circ_0000230	mmu-miR-297b-5	ENSMUST00000110698	ENSMUSG00000055737
mmu_circ_0000499	mmu-miR-297b-5	ENSMUST00000110698	ENSMUSG00000055737
mmu_circ_0000500	mmu-miR-297b-5	ENSMUST00000110698	ENSMUSG00000055737
mmu_circ_0000798	mmu-miR-297b-5	ENSMUST00000110698	ENSMUSG00000055737
mmu_circ_0000812	mmu-miR-297b-5	ENSMUST00000110698	ENSMUSG00000055737
mmu_circ_0000829	mmu-miR-297b-5	ENSMUST00000110698	ENSMUSG00000055737
mmu_circ_0000681	mmu-miR-297b-5	ENSMUST00000110698	ENSMUSG00000055737
mmu_circ_0000712	mmu-miR-297b-5	ENSMUST00000110698	ENSMUSG00000055737
mmu_circ_0000713	mmu-miR-297b-5	ENSMUST00000110698	ENSMUSG00000055737
mmu_circ_0000714	mmu-miR-297b-5	ENSMUST00000110698	ENSMUSG00000055737
mmu_circ_0000950	mmu-miR-297b-5	ENSMUST00000110698	ENSMUSG00000055737
mmu_circ_0000969	mmu-miR-297b-5	ENSMUST00000110698	ENSMUSG00000055737
mmu_circ_0000884	mmu-miR-297b-5	ENSMUST00000110698	ENSMUSG00000055737
mmu_circ_0000886	mmu-miR-297b-5	ENSMUST00000110698	ENSMUSG00000055737
mmu_circ_0000887	mmu-miR-297b-5	ENSMUST00000110698	ENSMUSG00000055737
mmu_circ_0000905	mmu-miR-297b-5	ENSMUST00000110698	ENSMUSG00000055737
mmu_circ_0000906	mmu-miR-297b-5	ENSMUST00000110698	ENSMUSG00000055737
mmu_circ_0000907	mmu-miR-297b-5	ENSMUST00000110698	ENSMUSG00000055737
mmu_circ_0000908	mmu-miR-297b-5	ENSMUST00000110698	ENSMUSG00000055737
novel_circ_0001680	mmu-miR-297b-5	ENSMUST00000110698	ENSMUSG00000055737
novel_circ_0001721	mmu-miR-297b-5	ENSMUST00000110698	ENSMUSG00000055737
novel_circ_0002082	mmu-miR-297b-5	ENSMUST00000110698	ENSMUSG00000055737
novel_circ_0001859	mmu-miR-297b-5	ENSMUST00000110698	ENSMUSG00000055737
novel_circ_0001861	mmu-miR-297b-5	ENSMUST00000110698	ENSMUSG00000055737
novel_circ_0001933	mmu-miR-297b-5	ENSMUST00000110698	ENSMUSG00000055737
novel_circ_0001934	mmu-miR-297b-5	ENSMUST00000110698	ENSMUSG00000055737
novel_circ_0002507	mmu-miR-297b-5	ENSMUST00000110698	ENSMUSG00000055737
novel_circ_0002508	mmu-miR-297b-5	ENSMUST00000110698	ENSMUSG00000055737
novel_circ_0002581	mmu-miR-297b-5	ENSMUST00000110698	ENSMUSG00000055737
novel_circ_0002374	mmu-miR-297b-5	ENSMUST00000110698	ENSMUSG00000055737
novel_circ_0002978	mmu-miR-297b-5	ENSMUST00000110698	ENSMUSG00000055737
novel_circ_0002990	mmu-miR-297b-5	ENSMUST00000110698	ENSMUSG00000055737
novel_circ_0002642	mmu-miR-297b-5	ENSMUST00000110698	ENSMUSG00000055737
novel_circ_0002695	mmu-miR-297b-5	ENSMUST00000110698	ENSMUSG00000055737
novel_circ_0002782	mmu-miR-297b-5	ENSMUST00000110698	ENSMUSG00000055737
novel_circ_0003016	mmu-miR-297b-5	ENSMUST00000110698	ENSMUSG00000055737
novel_circ_0000188	mmu-miR-297b-5	ENSMUST00000110698	ENSMUSG00000055737
novel_circ_0000268	mmu-miR-297b-5	ENSMUST00000110698	ENSMUSG00000055737
novel_circ_0000985	mmu-miR-297b-5	ENSMUST00000110698	ENSMUSG00000055737
novel_circ_0000807	mmu-miR-297b-5	ENSMUST00000110698	ENSMUSG00000055737
novel_circ_0001191	mmu-miR-297b-5	ENSMUST00000110698	ENSMUSG00000055737

novel_circ_0001191	mmu-miR-297b-5	ENSMUST00000110698	ENSMUSG00000055737
novel_circ_0001256	mmu-miR-455-3p	ENSMUST00000003659	ENSMUSG00000031849
novel_circ_0001256	mmu-miR-455-3p	ENSMUST00000212488	ENSMUSG00000031849
novel_circ_0001290	mmu-miR-455-3p	ENSMUST00000003659	ENSMUSG00000031849
novel_circ_0001290	mmu-miR-455-3p	ENSMUST00000212488	ENSMUSG00000031849
novel_circ_0001086	mmu-miR-455-3p	ENSMUST00000003659	ENSMUSG00000031849
novel_circ_0001086	mmu-miR-455-3p	ENSMUST00000212488	ENSMUSG00000031849
novel_circ_0001134	mmu-miR-455-3p	ENSMUST00000003659	ENSMUSG00000031849
novel_circ_0001134	mmu-miR-455-3p	ENSMUST00000212488	ENSMUSG00000031849
novel_circ_0001134	mmu-miR-455-3p	ENSMUST00000003659	ENSMUSG00000031849
novel_circ_0001134	mmu-miR-455-3p	ENSMUST00000212488	ENSMUSG00000031849
novel_circ_0001505	mmu-miR-455-3p	ENSMUST00000003659	ENSMUSG00000031849
novel_circ_0001505	mmu-miR-455-3p	ENSMUST00000212488	ENSMUSG00000031849
novel_circ_0001349	mmu-miR-455-3p	ENSMUST00000003659	ENSMUSG00000031849
novel_circ_0001349	mmu-miR-455-3p	ENSMUST00000212488	ENSMUSG00000031849
novel_circ_0001401	mmu-miR-455-3p	ENSMUST00000003659	ENSMUSG00000031849
novel_circ_0001401	mmu-miR-455-3p	ENSMUST00000212488	ENSMUSG00000031849
novel_circ_0001402	mmu-miR-455-3p	ENSMUST00000003659	ENSMUSG00000031849
novel_circ_0001402	mmu-miR-455-3p	ENSMUST00000212488	ENSMUSG00000031849
mmu_circ_0000116	mmu-miR-455-3p	ENSMUST00000003659	ENSMUSG00000031849
mmu_circ_0000116	mmu-miR-455-3p	ENSMUST00000212488	ENSMUSG00000031849
mmu_circ_0000027	mmu-miR-455-3p	ENSMUST00000003659	ENSMUSG00000031849
mmu_circ_0000027	mmu-miR-455-3p	ENSMUST00000212488	ENSMUSG00000031849
mmu_circ_0000028	mmu-miR-455-3p	ENSMUST00000003659	ENSMUSG00000031849
mmu_circ_0000028	mmu-miR-455-3p	ENSMUST00000212488	ENSMUSG00000031849
mmu_circ_0001097	mmu-miR-455-3p	ENSMUST00000003659	ENSMUSG00000031849
mmu_circ_0001097	mmu-miR-455-3p	ENSMUST00000212488	ENSMUSG00000031849
mmu_circ_0001376	mmu-miR-455-3p	ENSMUST00000003659	ENSMUSG00000031849
mmu_circ_0001376	mmu-miR-455-3p	ENSMUST00000212488	ENSMUSG00000031849
mmu_circ_0001345	mmu-miR-455-3p	ENSMUST00000003659	ENSMUSG00000031849
mmu_circ_0001345	mmu-miR-455-3p	ENSMUST00000212488	ENSMUSG00000031849
mmu_circ_0001356	mmu-miR-455-3p	ENSMUST00000003659	ENSMUSG00000031849
mmu_circ_0001356	mmu-miR-455-3p	ENSMUST00000212488	ENSMUSG00000031849
mmu_circ_0001279	mmu-miR-455-3p	ENSMUST00000003659	ENSMUSG00000031849
mmu_circ_0001279	mmu-miR-455-3p	ENSMUST00000212488	ENSMUSG00000031849
mmu_circ_0001610	mmu-miR-455-3p	ENSMUST00000003659	ENSMUSG00000031849
mmu_circ_0001610	mmu-miR-455-3p	ENSMUST00000212488	ENSMUSG00000031849
mmu_circ_0001598	mmu-miR-455-3p	ENSMUST00000003659	ENSMUSG00000031849
mmu_circ_0001598	mmu-miR-455-3p	ENSMUST00000212488	ENSMUSG00000031849
mmu_circ_0001528	mmu-miR-455-3p	ENSMUST00000003659	ENSMUSG00000031849
mmu_circ_0001528	mmu-miR-455-3p	ENSMUST00000212488	ENSMUSG00000031849
mmu_circ_0001529	mmu-miR-455-3p	ENSMUST00000003659	ENSMUSG00000031849
mmu_circ_0001529	mmu-miR-455-3p	ENSMUST00000212488	ENSMUSG00000031849
mmu_circ_0001454	mmu-miR-455-3p	ENSMUST00000003659	ENSMUSG00000031849
mmu_circ_0001454	mmu-miR-455-3p	ENSMUST00000212488	ENSMUSG00000031849
mmu_circ_0001469	mmu-miR-455-3p	ENSMUST00000003659	ENSMUSG00000031849
mmu_circ_0001469	mmu-miR-455-3p	ENSMUST00000212488	ENSMUSG00000031849
mmu_circ_0001500	mmu-miR-455-3p	ENSMUST00000003659	ENSMUSG00000031849
mmu_circ_0001500	mmu-miR-455-3p	ENSMUST00000212488	ENSMUSG00000031849
mmu_circ_0001766	mmu-miR-455-3p	ENSMUST00000003659	ENSMUSG00000031849
mmu_circ_0001766	mmu-miR-455-3p	ENSMUST00000212488	ENSMUSG00000031849
mmu_circ_0001767	mmu-miR-455-3p	ENSMUST00000003659	ENSMUSG00000031849
mmu_circ_0001767	mmu-miR-455-3p	ENSMUST00000212488	ENSMUSG00000031849
mmu_circ_0001770	mmu-miR-455-3p	ENSMUST00000003659	ENSMUSG00000031849
mmu_circ_0001770	mmu-miR-455-3p	ENSMUST00000212488	ENSMUSG00000031849
mmu_circ_0001774	mmu-miR-455-3p	ENSMUST00000003659	ENSMUSG00000031849
mmu_circ_0001774	mmu-miR-455-3p	ENSMUST00000212488	ENSMUSG00000031849
mmu_circ_0001795	mmu-miR-455-3p	ENSMUST00000003659	ENSMUSG00000031849

mmu_circ_0001795	mmu-miR-455-3p	ENSMUST00000212488	ENSMUSG00000031849
mmu_circ_0001827	mmu-miR-455-3p	ENSMUST00000003659	ENSMUSG00000031849
mmu_circ_0001827	mmu-miR-455-3p	ENSMUST00000212488	ENSMUSG00000031849
mmu_circ_0000243	mmu-miR-455-3p	ENSMUST00000003659	ENSMUSG00000031849
mmu_circ_0000243	mmu-miR-455-3p	ENSMUST00000212488	ENSMUSG00000031849
mmu_circ_0000141	mmu-miR-455-3p	ENSMUST00000003659	ENSMUSG00000031849
mmu_circ_0000141	mmu-miR-455-3p	ENSMUST00000212488	ENSMUSG00000031849
mmu_circ_0000500	mmu-miR-455-3p	ENSMUST00000003659	ENSMUSG00000031849
mmu_circ_0000500	mmu-miR-455-3p	ENSMUST00000212488	ENSMUSG00000031849
mmu_circ_0000491	mmu-miR-455-3p	ENSMUST00000003659	ENSMUSG00000031849
mmu_circ_0000491	mmu-miR-455-3p	ENSMUST00000212488	ENSMUSG00000031849
mmu_circ_0000371	mmu-miR-455-3p	ENSMUST00000003659	ENSMUSG00000031849
mmu_circ_0000371	mmu-miR-455-3p	ENSMUST00000212488	ENSMUSG00000031849
mmu_circ_0000372	mmu-miR-455-3p	ENSMUST00000003659	ENSMUSG00000031849
mmu_circ_0000372	mmu-miR-455-3p	ENSMUST00000212488	ENSMUSG00000031849
mmu_circ_0000399	mmu-miR-455-3p	ENSMUST00000003659	ENSMUSG00000031849
mmu_circ_0000399	mmu-miR-455-3p	ENSMUST00000212488	ENSMUSG00000031849
mmu_circ_0000402	mmu-miR-455-3p	ENSMUST00000003659	ENSMUSG00000031849
mmu_circ_0000402	mmu-miR-455-3p	ENSMUST00000212488	ENSMUSG00000031849
mmu_circ_0000530	mmu-miR-455-3p	ENSMUST00000003659	ENSMUSG00000031849
mmu_circ_0000530	mmu-miR-455-3p	ENSMUST00000212488	ENSMUSG00000031849
mmu_circ_0000829	mmu-miR-455-3p	ENSMUST00000003659	ENSMUSG00000031849
mmu_circ_0000829	mmu-miR-455-3p	ENSMUST00000212488	ENSMUSG00000031849
mmu_circ_0000831	mmu-miR-455-3p	ENSMUST00000003659	ENSMUSG00000031849
mmu_circ_0000831	mmu-miR-455-3p	ENSMUST00000212488	ENSMUSG00000031849
mmu_circ_0000945	mmu-miR-455-3p	ENSMUST00000003659	ENSMUSG00000031849
mmu_circ_0000945	mmu-miR-455-3p	ENSMUST00000212488	ENSMUSG00000031849
mmu_circ_0000955	mmu-miR-455-3p	ENSMUST00000003659	ENSMUSG00000031849
mmu_circ_0000955	mmu-miR-455-3p	ENSMUST00000212488	ENSMUSG00000031849
mmu_circ_0000956	mmu-miR-455-3p	ENSMUST00000003659	ENSMUSG00000031849
mmu_circ_0000956	mmu-miR-455-3p	ENSMUST00000212488	ENSMUSG00000031849
mmu_circ_0000969	mmu-miR-455-3p	ENSMUST00000003659	ENSMUSG00000031849
mmu_circ_0000969	mmu-miR-455-3p	ENSMUST00000212488	ENSMUSG00000031849
mmu_circ_0000884	mmu-miR-455-3p	ENSMUST00000003659	ENSMUSG00000031849
mmu_circ_0000884	mmu-miR-455-3p	ENSMUST00000212488	ENSMUSG00000031849
mmu_circ_0000897	mmu-miR-455-3p	ENSMUST00000003659	ENSMUSG00000031849
mmu_circ_0000897	mmu-miR-455-3p	ENSMUST00000212488	ENSMUSG00000031849
mmu_circ_0000897	mmu-miR-455-3p	ENSMUST00000003659	ENSMUSG00000031849
mmu_circ_0000897	mmu-miR-455-3p	ENSMUST00000212488	ENSMUSG00000031849
mmu_circ_0000898	mmu-miR-455-3p	ENSMUST00000003659	ENSMUSG00000031849
mmu_circ_0000898	mmu-miR-455-3p	ENSMUST00000212488	ENSMUSG00000031849
mmu_circ_0000898	mmu-miR-455-3p	ENSMUST00000003659	ENSMUSG00000031849
mmu_circ_0000898	mmu-miR-455-3p	ENSMUST00000212488	ENSMUSG00000031849
mmu_circ_0000906	mmu-miR-455-3p	ENSMUST00000003659	ENSMUSG00000031849
mmu_circ_0000906	mmu-miR-455-3p	ENSMUST00000212488	ENSMUSG00000031849
mmu_circ_0000907	mmu-miR-455-3p	ENSMUST00000003659	ENSMUSG00000031849
mmu_circ_0000907	mmu-miR-455-3p	ENSMUST00000212488	ENSMUSG00000031849
mmu_circ_0000912	mmu-miR-455-3p	ENSMUST00000003659	ENSMUSG00000031849
mmu_circ_0000912	mmu-miR-455-3p	ENSMUST00000212488	ENSMUSG00000031849
novel_circ_0001567	mmu-miR-455-3p	ENSMUST00000003659	ENSMUSG00000031849
novel_circ_0001567	mmu-miR-455-3p	ENSMUST00000212488	ENSMUSG00000031849
novel_circ_0002066	mmu-miR-455-3p	ENSMUST00000003659	ENSMUSG00000031849
novel_circ_0002066	mmu-miR-455-3p	ENSMUST00000212488	ENSMUSG00000031849
novel_circ_0002129	mmu-miR-455-3p	ENSMUST00000003659	ENSMUSG00000031849
novel_circ_0002129	mmu-miR-455-3p	ENSMUST00000212488	ENSMUSG00000031849
novel_circ_0002187	mmu-miR-455-3p	ENSMUST00000003659	ENSMUSG00000031849
novel_circ_0002187	mmu-miR-455-3p	ENSMUST00000212488	ENSMUSG00000031849
novel_circ_0002209	mmu-miR-455-3p	ENSMUST00000003659	ENSMUSG00000031849

novel_circ_0002209	mmu-miR-455-3p	ENSMUST00000212488	ENSMUSG00000031849
novel_circ_0002217	mmu-miR-455-3p	ENSMUST00000003659	ENSMUSG00000031849
novel_circ_0002217	mmu-miR-455-3p	ENSMUST00000212488	ENSMUSG00000031849
novel_circ_0002217	mmu-miR-455-3p	ENSMUST00000003659	ENSMUSG00000031849
novel_circ_0002217	mmu-miR-455-3p	ENSMUST00000212488	ENSMUSG00000031849
novel_circ_0001782	mmu-miR-455-3p	ENSMUST00000003659	ENSMUSG00000031849
novel_circ_0001782	mmu-miR-455-3p	ENSMUST00000212488	ENSMUSG00000031849
novel_circ_0001783	mmu-miR-455-3p	ENSMUST00000003659	ENSMUSG00000031849
novel_circ_0001783	mmu-miR-455-3p	ENSMUST00000212488	ENSMUSG00000031849
novel_circ_0001903	mmu-miR-455-3p	ENSMUST00000003659	ENSMUSG00000031849
novel_circ_0001903	mmu-miR-455-3p	ENSMUST00000212488	ENSMUSG00000031849
novel_circ_0001911	mmu-miR-455-3p	ENSMUST00000003659	ENSMUSG00000031849
novel_circ_0001911	mmu-miR-455-3p	ENSMUST00000212488	ENSMUSG00000031849
novel_circ_0001927	mmu-miR-455-3p	ENSMUST00000003659	ENSMUSG00000031849
novel_circ_0001927	mmu-miR-455-3p	ENSMUST00000212488	ENSMUSG00000031849
novel_circ_0001997	mmu-miR-455-3p	ENSMUST00000003659	ENSMUSG00000031849
novel_circ_0001997	mmu-miR-455-3p	ENSMUST00000212488	ENSMUSG00000031849
novel_circ_0001997	mmu-miR-455-3p	ENSMUST00000003659	ENSMUSG00000031849
novel_circ_0001997	mmu-miR-455-3p	ENSMUST00000212488	ENSMUSG00000031849
novel_circ_0002035	mmu-miR-455-3p	ENSMUST00000003659	ENSMUSG00000031849
novel_circ_0002035	mmu-miR-455-3p	ENSMUST00000212488	ENSMUSG00000031849
novel_circ_0002035	mmu-miR-455-3p	ENSMUST00000003659	ENSMUSG00000031849
novel_circ_0002035	mmu-miR-455-3p	ENSMUST00000212488	ENSMUSG00000031849
novel_circ_0002417	mmu-miR-455-3p	ENSMUST00000003659	ENSMUSG00000031849
novel_circ_0002417	mmu-miR-455-3p	ENSMUST00000212488	ENSMUSG00000031849
novel_circ_0002440	mmu-miR-455-3p	ENSMUST00000003659	ENSMUSG00000031849
novel_circ_0002440	mmu-miR-455-3p	ENSMUST00000212488	ENSMUSG00000031849
novel_circ_0002484	mmu-miR-455-3p	ENSMUST00000003659	ENSMUSG00000031849
novel_circ_0002484	mmu-miR-455-3p	ENSMUST00000212488	ENSMUSG00000031849
novel_circ_0002485	mmu-miR-455-3p	ENSMUST00000003659	ENSMUSG00000031849
novel_circ_0002485	mmu-miR-455-3p	ENSMUST00000212488	ENSMUSG00000031849
novel_circ_0002499	mmu-miR-455-3p	ENSMUST00000003659	ENSMUSG00000031849
novel_circ_0002499	mmu-miR-455-3p	ENSMUST00000212488	ENSMUSG00000031849
novel_circ_0002508	mmu-miR-455-3p	ENSMUST00000003659	ENSMUSG00000031849
novel_circ_0002508	mmu-miR-455-3p	ENSMUST00000212488	ENSMUSG00000031849
novel_circ_0002221	mmu-miR-455-3p	ENSMUST00000003659	ENSMUSG00000031849
novel_circ_0002221	mmu-miR-455-3p	ENSMUST00000212488	ENSMUSG00000031849
novel_circ_0002276	mmu-miR-455-3p	ENSMUST00000003659	ENSMUSG00000031849
novel_circ_0002276	mmu-miR-455-3p	ENSMUST00000212488	ENSMUSG00000031849
novel_circ_0002299	mmu-miR-455-3p	ENSMUST00000003659	ENSMUSG00000031849
novel_circ_0002299	mmu-miR-455-3p	ENSMUST00000212488	ENSMUSG00000031849
novel_circ_0002299	mmu-miR-455-3p	ENSMUST00000003659	ENSMUSG00000031849
novel_circ_0002299	mmu-miR-455-3p	ENSMUST00000212488	ENSMUSG00000031849
novel_circ_0002324	mmu-miR-455-3p	ENSMUST00000003659	ENSMUSG00000031849
novel_circ_0002324	mmu-miR-455-3p	ENSMUST00000212488	ENSMUSG00000031849
novel_circ_0002331	mmu-miR-455-3p	ENSMUST00000003659	ENSMUSG00000031849
novel_circ_0002331	mmu-miR-455-3p	ENSMUST00000212488	ENSMUSG00000031849
novel_circ_0002344	mmu-miR-455-3p	ENSMUST00000003659	ENSMUSG00000031849
novel_circ_0002344	mmu-miR-455-3p	ENSMUST00000212488	ENSMUSG00000031849
novel_circ_0002378	mmu-miR-455-3p	ENSMUST00000003659	ENSMUSG00000031849
novel_circ_0002378	mmu-miR-455-3p	ENSMUST00000212488	ENSMUSG00000031849
novel_circ_0002379	mmu-miR-455-3p	ENSMUST00000003659	ENSMUSG00000031849
novel_circ_0002379	mmu-miR-455-3p	ENSMUST00000212488	ENSMUSG00000031849
novel_circ_0002399	mmu-miR-455-3p	ENSMUST00000003659	ENSMUSG00000031849
novel_circ_0002399	mmu-miR-455-3p	ENSMUST00000212488	ENSMUSG00000031849
novel_circ_0002838	mmu-miR-455-3p	ENSMUST00000003659	ENSMUSG00000031849
novel_circ_0002838	mmu-miR-455-3p	ENSMUST00000212488	ENSMUSG00000031849
novel_circ_0002964	mmu-miR-455-3p	ENSMUST00000003659	ENSMUSG00000031849

novel_circ_0002964	mmu-miR-455-3p	ENSMUST00000212488	ENSMUSG00000031849
novel_circ_0002642	mmu-miR-455-3p	ENSMUST00000003659	ENSMUSG00000031849
novel_circ_0002642	mmu-miR-455-3p	ENSMUST00000212488	ENSMUSG00000031849
novel_circ_0002699	mmu-miR-455-3p	ENSMUST00000003659	ENSMUSG00000031849
novel_circ_0002699	mmu-miR-455-3p	ENSMUST00000212488	ENSMUSG00000031849
novel_circ_0002719	mmu-miR-455-3p	ENSMUST00000003659	ENSMUSG00000031849
novel_circ_0002719	mmu-miR-455-3p	ENSMUST00000212488	ENSMUSG00000031849
novel_circ_0002719	mmu-miR-455-3p	ENSMUST00000003659	ENSMUSG00000031849
novel_circ_0002719	mmu-miR-455-3p	ENSMUST00000212488	ENSMUSG00000031849
novel_circ_0002755	mmu-miR-455-3p	ENSMUST00000003659	ENSMUSG00000031849
novel_circ_0002755	mmu-miR-455-3p	ENSMUST00000212488	ENSMUSG00000031849
novel_circ_0002769	mmu-miR-455-3p	ENSMUST00000003659	ENSMUSG00000031849
novel_circ_0002769	mmu-miR-455-3p	ENSMUST00000212488	ENSMUSG00000031849
novel_circ_0003242	mmu-miR-455-3p	ENSMUST00000003659	ENSMUSG00000031849
novel_circ_0003242	mmu-miR-455-3p	ENSMUST00000212488	ENSMUSG00000031849
novel_circ_0003280	mmu-miR-455-3p	ENSMUST00000003659	ENSMUSG00000031849
novel_circ_0003280	mmu-miR-455-3p	ENSMUST00000212488	ENSMUSG00000031849
novel_circ_0003280	mmu-miR-455-3p	ENSMUST00000003659	ENSMUSG00000031849
novel_circ_0003280	mmu-miR-455-3p	ENSMUST00000212488	ENSMUSG00000031849
novel_circ_0003316	mmu-miR-455-3p	ENSMUST00000003659	ENSMUSG00000031849
novel_circ_0003316	mmu-miR-455-3p	ENSMUST00000212488	ENSMUSG00000031849
novel_circ_0003316	mmu-miR-455-3p	ENSMUST00000003659	ENSMUSG00000031849
novel_circ_0003316	mmu-miR-455-3p	ENSMUST00000212488	ENSMUSG00000031849
novel_circ_0003420	mmu-miR-455-3p	ENSMUST00000003659	ENSMUSG00000031849
novel_circ_0003420	mmu-miR-455-3p	ENSMUST00000212488	ENSMUSG00000031849
novel_circ_0003159	mmu-miR-455-3p	ENSMUST00000003659	ENSMUSG00000031849
novel_circ_0003159	mmu-miR-455-3p	ENSMUST00000212488	ENSMUSG00000031849
novel_circ_0003160	mmu-miR-455-3p	ENSMUST00000003659	ENSMUSG00000031849
novel_circ_0003160	mmu-miR-455-3p	ENSMUST00000212488	ENSMUSG00000031849
novel_circ_0003471	mmu-miR-455-3p	ENSMUST00000003659	ENSMUSG00000031849
novel_circ_0003471	mmu-miR-455-3p	ENSMUST00000212488	ENSMUSG00000031849
novel_circ_0003483	mmu-miR-455-3p	ENSMUST00000003659	ENSMUSG00000031849
novel_circ_0003483	mmu-miR-455-3p	ENSMUST00000212488	ENSMUSG00000031849
novel_circ_0000178	mmu-miR-455-3p	ENSMUST00000003659	ENSMUSG00000031849
novel_circ_0000178	mmu-miR-455-3p	ENSMUST00000212488	ENSMUSG00000031849
novel_circ_0000220	mmu-miR-455-3p	ENSMUST00000003659	ENSMUSG00000031849
novel_circ_0000220	mmu-miR-455-3p	ENSMUST00000212488	ENSMUSG00000031849
novel_circ_0000230	mmu-miR-455-3p	ENSMUST00000003659	ENSMUSG00000031849
novel_circ_0000230	mmu-miR-455-3p	ENSMUST00000212488	ENSMUSG00000031849
novel_circ_0000230	mmu-miR-455-3p	ENSMUST00000003659	ENSMUSG00000031849
novel_circ_0000230	mmu-miR-455-3p	ENSMUST00000212488	ENSMUSG00000031849
novel_circ_0000237	mmu-miR-455-3p	ENSMUST00000003659	ENSMUSG00000031849
novel_circ_0000237	mmu-miR-455-3p	ENSMUST00000212488	ENSMUSG00000031849
novel_circ_0000240	mmu-miR-455-3p	ENSMUST00000003659	ENSMUSG00000031849
novel_circ_0000240	mmu-miR-455-3p	ENSMUST00000212488	ENSMUSG00000031849
novel_circ_0000267	mmu-miR-455-3p	ENSMUST00000003659	ENSMUSG00000031849
novel_circ_0000267	mmu-miR-455-3p	ENSMUST00000212488	ENSMUSG00000031849
novel_circ_0000308	mmu-miR-455-3p	ENSMUST00000003659	ENSMUSG00000031849
novel_circ_0000308	mmu-miR-455-3p	ENSMUST00000212488	ENSMUSG00000031849
novel_circ_0000386	mmu-miR-455-3p	ENSMUST00000003659	ENSMUSG00000031849
novel_circ_0000386	mmu-miR-455-3p	ENSMUST00000212488	ENSMUSG00000031849
novel_circ_0000062	mmu-miR-455-3p	ENSMUST00000003659	ENSMUSG00000031849
novel_circ_0000062	mmu-miR-455-3p	ENSMUST00000212488	ENSMUSG00000031849
novel_circ_0000078	mmu-miR-455-3p	ENSMUST00000003659	ENSMUSG00000031849
novel_circ_0000078	mmu-miR-455-3p	ENSMUST00000212488	ENSMUSG00000031849
novel_circ_0000078	mmu-miR-455-3p	ENSMUST00000003659	ENSMUSG00000031849
novel_circ_0000078	mmu-miR-455-3p	ENSMUST00000212488	ENSMUSG00000031849
novel_circ_0000107	mmu-miR-455-3p	ENSMUST00000003659	ENSMUSG00000031849

novel_circ_0000107	mmu-miR-455-3p	ENSMUST00000212488	ENSMUSG00000031849
novel_circ_0000108	mmu-miR-455-3p	ENSMUST00000003659	ENSMUSG00000031849
novel_circ_0000108	mmu-miR-455-3p	ENSMUST00000212488	ENSMUSG00000031849
novel_circ_0000154	mmu-miR-455-3p	ENSMUST00000003659	ENSMUSG00000031849
novel_circ_0000154	mmu-miR-455-3p	ENSMUST00000212488	ENSMUSG00000031849
novel_circ_0000781	mmu-miR-455-3p	ENSMUST00000003659	ENSMUSG00000031849
novel_circ_0000781	mmu-miR-455-3p	ENSMUST00000212488	ENSMUSG00000031849
novel_circ_0000514	mmu-miR-455-3p	ENSMUST00000003659	ENSMUSG00000031849
novel_circ_0000514	mmu-miR-455-3p	ENSMUST00000212488	ENSMUSG00000031849
novel_circ_0000549	mmu-miR-455-3p	ENSMUST00000003659	ENSMUSG00000031849
novel_circ_0000549	mmu-miR-455-3p	ENSMUST00000212488	ENSMUSG00000031849
novel_circ_0000578	mmu-miR-455-3p	ENSMUST00000003659	ENSMUSG00000031849
novel_circ_0000578	mmu-miR-455-3p	ENSMUST00000212488	ENSMUSG00000031849
novel_circ_0000589	mmu-miR-455-3p	ENSMUST00000003659	ENSMUSG00000031849
novel_circ_0000589	mmu-miR-455-3p	ENSMUST00000212488	ENSMUSG00000031849
novel_circ_0000987	mmu-miR-455-3p	ENSMUST00000003659	ENSMUSG00000031849
novel_circ_0000987	mmu-miR-455-3p	ENSMUST00000212488	ENSMUSG00000031849
novel_circ_0001027	mmu-miR-455-3p	ENSMUST00000003659	ENSMUSG00000031849
novel_circ_0001027	mmu-miR-455-3p	ENSMUST00000212488	ENSMUSG00000031849
novel_circ_0001048	mmu-miR-455-3p	ENSMUST00000003659	ENSMUSG00000031849
novel_circ_0001048	mmu-miR-455-3p	ENSMUST00000212488	ENSMUSG00000031849
novel_circ_0001257	mmu-miR-709	ENSMUST00000073554	ENSMUSG00000062329
novel_circ_0001103	mmu-miR-709	ENSMUST00000073554	ENSMUSG00000062329
novel_circ_0001106	mmu-miR-709	ENSMUST00000073554	ENSMUSG00000062329
novel_circ_0001136	mmu-miR-709	ENSMUST00000073554	ENSMUSG00000062329
novel_circ_0001493	mmu-miR-709	ENSMUST00000073554	ENSMUSG00000062329
novel_circ_0001533	mmu-miR-709	ENSMUST00000073554	ENSMUSG00000062329
mmu_circ_0000075	mmu-miR-709	ENSMUST00000073554	ENSMUSG00000062329
mmu_circ_0000028	mmu-miR-709	ENSMUST00000073554	ENSMUSG00000062329
mmu_circ_0000040	mmu-miR-709	ENSMUST00000073554	ENSMUSG00000062329
mmu_circ_0000041	mmu-miR-709	ENSMUST00000073554	ENSMUSG00000062329
mmu_circ_0000042	mmu-miR-709	ENSMUST00000073554	ENSMUSG00000062329
mmu_circ_0000046	mmu-miR-709	ENSMUST00000073554	ENSMUSG00000062329
mmu_circ_0000049	mmu-miR-709	ENSMUST00000073554	ENSMUSG00000062329
mmu_circ_0000063	mmu-miR-709	ENSMUST00000073554	ENSMUSG00000062329
mmu_circ_0001162	mmu-miR-709	ENSMUST00000073554	ENSMUSG00000062329
mmu_circ_0001123	mmu-miR-709	ENSMUST00000073554	ENSMUSG00000062329
mmu_circ_0001135	mmu-miR-709	ENSMUST00000073554	ENSMUSG00000062329
mmu_circ_0001070	mmu-miR-709	ENSMUST00000073554	ENSMUSG00000062329
mmu_circ_0001099	mmu-miR-709	ENSMUST00000073554	ENSMUSG00000062329
mmu_circ_0001021	mmu-miR-709	ENSMUST00000073554	ENSMUSG00000062329
mmu_circ_0001376	mmu-miR-709	ENSMUST00000073554	ENSMUSG00000062329
mmu_circ_0001405	mmu-miR-709	ENSMUST00000073554	ENSMUSG00000062329
mmu_circ_0001333	mmu-miR-709	ENSMUST00000073554	ENSMUSG00000062329
mmu_circ_0001333	mmu-miR-709	ENSMUST00000073554	ENSMUSG00000062329
mmu_circ_0001255	mmu-miR-709	ENSMUST00000073554	ENSMUSG00000062329
mmu_circ_0001256	mmu-miR-709	ENSMUST00000073554	ENSMUSG00000062329
mmu_circ_0001263	mmu-miR-709	ENSMUST00000073554	ENSMUSG00000062329
mmu_circ_0001264	mmu-miR-709	ENSMUST00000073554	ENSMUSG00000062329
mmu_circ_0001634	mmu-miR-709	ENSMUST00000073554	ENSMUSG00000062329
mmu_circ_0001454	mmu-miR-709	ENSMUST00000073554	ENSMUSG00000062329
mmu_circ_0001465	mmu-miR-709	ENSMUST00000073554	ENSMUSG00000062329
mmu_circ_0001470	mmu-miR-709	ENSMUST00000073554	ENSMUSG00000062329
mmu_circ_0001499	mmu-miR-709	ENSMUST00000073554	ENSMUSG00000062329
mmu_circ_0001500	mmu-miR-709	ENSMUST00000073554	ENSMUSG00000062329
mmu_circ_0001745	mmu-miR-709	ENSMUST00000073554	ENSMUSG00000062329
mmu_circ_0001827	mmu-miR-709	ENSMUST00000073554	ENSMUSG00000062329
mmu_circ_0001664	mmu-miR-709	ENSMUST00000073554	ENSMUSG00000062329

mmu_circ_0001695	mmu-miR-709	ENSMUST00000073554	ENSMUSG00000062329
mmu_circ_0001704	mmu-miR-709	ENSMUST00000073554	ENSMUSG00000062329
mmu_circ_0000230	mmu-miR-709	ENSMUST00000073554	ENSMUSG00000062329
mmu_circ_0000294	mmu-miR-709	ENSMUST00000073554	ENSMUSG00000062329
mmu_circ_0000301	mmu-miR-709	ENSMUST00000073554	ENSMUSG00000062329
mmu_circ_0000172	mmu-miR-709	ENSMUST00000073554	ENSMUSG00000062329
mmu_circ_0000498	mmu-miR-709	ENSMUST00000073554	ENSMUSG00000062329
mmu_circ_0000499	mmu-miR-709	ENSMUST00000073554	ENSMUSG00000062329
mmu_circ_0000500	mmu-miR-709	ENSMUST00000073554	ENSMUSG00000062329
mmu_circ_0000454	mmu-miR-709	ENSMUST00000073554	ENSMUSG00000062329
mmu_circ_0000470	mmu-miR-709	ENSMUST00000073554	ENSMUSG00000062329
mmu_circ_0000371	mmu-miR-709	ENSMUST00000073554	ENSMUSG00000062329
mmu_circ_0000652	mmu-miR-709	ENSMUST00000073554	ENSMUSG00000062329
mmu_circ_0000623	mmu-miR-709	ENSMUST00000073554	ENSMUSG00000062329
mmu_circ_0000628	mmu-miR-709	ENSMUST00000073554	ENSMUSG00000062329
mmu_circ_0000530	mmu-miR-709	ENSMUST00000073554	ENSMUSG00000062329
mmu_circ_0000828	mmu-miR-709	ENSMUST00000073554	ENSMUSG00000062329
mmu_circ_0000829	mmu-miR-709	ENSMUST00000073554	ENSMUSG00000062329
mmu_circ_0000831	mmu-miR-709	ENSMUST00000073554	ENSMUSG00000062329
mmu_circ_0000833	mmu-miR-709	ENSMUST00000073554	ENSMUSG00000062329
mmu_circ_0000711	mmu-miR-709	ENSMUST00000073554	ENSMUSG00000062329
mmu_circ_0000712	mmu-miR-709	ENSMUST00000073554	ENSMUSG00000062329
mmu_circ_0000713	mmu-miR-709	ENSMUST00000073554	ENSMUSG00000062329
mmu_circ_0000714	mmu-miR-709	ENSMUST00000073554	ENSMUSG00000062329
mmu_circ_0000950	mmu-miR-709	ENSMUST00000073554	ENSMUSG00000062329
mmu_circ_0000918	mmu-miR-709	ENSMUST00000073554	ENSMUSG00000062329
mmu_circ_0000919	mmu-miR-709	ENSMUST00000073554	ENSMUSG00000062329
mmu_circ_0000921	mmu-miR-709	ENSMUST00000073554	ENSMUSG00000062329
mmu_circ_0000969	mmu-miR-709	ENSMUST00000073554	ENSMUSG00000062329
mmu_circ_0000854	mmu-miR-709	ENSMUST00000073554	ENSMUSG00000062329
mmu_circ_0000884	mmu-miR-709	ENSMUST00000073554	ENSMUSG00000062329
mmu_circ_0000886	mmu-miR-709	ENSMUST00000073554	ENSMUSG00000062329
mmu_circ_0000887	mmu-miR-709	ENSMUST00000073554	ENSMUSG00000062329
mmu_circ_0000905	mmu-miR-709	ENSMUST00000073554	ENSMUSG00000062329
mmu_circ_0000906	mmu-miR-709	ENSMUST00000073554	ENSMUSG00000062329
mmu_circ_0000907	mmu-miR-709	ENSMUST00000073554	ENSMUSG00000062329
mmu_circ_0000908	mmu-miR-709	ENSMUST00000073554	ENSMUSG00000062329
mmu_circ_0000912	mmu-miR-709	ENSMUST00000073554	ENSMUSG00000062329
novel_circ_0001561	mmu-miR-709	ENSMUST00000073554	ENSMUSG00000062329
novel_circ_0001576	mmu-miR-709	ENSMUST00000073554	ENSMUSG00000062329
novel_circ_0001696	mmu-miR-709	ENSMUST00000073554	ENSMUSG00000062329
novel_circ_0001708	mmu-miR-709	ENSMUST00000073554	ENSMUSG00000062329
novel_circ_0001728	mmu-miR-709	ENSMUST00000073554	ENSMUSG00000062329
novel_circ_0002122	mmu-miR-709	ENSMUST00000073554	ENSMUSG00000062329
novel_circ_0002129	mmu-miR-709	ENSMUST00000073554	ENSMUSG00000062329
novel_circ_0002162	mmu-miR-709	ENSMUST00000073554	ENSMUSG00000062329
novel_circ_0002183	mmu-miR-709	ENSMUST00000073554	ENSMUSG00000062329
novel_circ_0002192	mmu-miR-709	ENSMUST00000073554	ENSMUSG00000062329
novel_circ_0002216	mmu-miR-709	ENSMUST00000073554	ENSMUSG00000062329
novel_circ_0001756	mmu-miR-709	ENSMUST00000073554	ENSMUSG00000062329
novel_circ_0001842	mmu-miR-709	ENSMUST00000073554	ENSMUSG00000062329
novel_circ_0001859	mmu-miR-709	ENSMUST00000073554	ENSMUSG00000062329
novel_circ_0001861	mmu-miR-709	ENSMUST00000073554	ENSMUSG00000062329
novel_circ_0001864	mmu-miR-709	ENSMUST00000073554	ENSMUSG00000062329
novel_circ_0001871	mmu-miR-709	ENSMUST00000073554	ENSMUSG00000062329
novel_circ_0001873	mmu-miR-709	ENSMUST00000073554	ENSMUSG00000062329
novel_circ_0001874	mmu-miR-709	ENSMUST00000073554	ENSMUSG00000062329
novel_circ_0001946	mmu-miR-709	ENSMUST00000073554	ENSMUSG00000062329

novel_circ_0002486	mmu-miR-709	ENSMUST00000073554	ENSMUSG00000062329
novel_circ_0002503	mmu-miR-709	ENSMUST00000073554	ENSMUSG00000062329
novel_circ_0002504	mmu-miR-709	ENSMUST00000073554	ENSMUSG00000062329
novel_circ_0002539	mmu-miR-709	ENSMUST00000073554	ENSMUSG00000062329
novel_circ_0002539	mmu-miR-709	ENSMUST00000073554	ENSMUSG00000062329
novel_circ_0002233	mmu-miR-709	ENSMUST00000073554	ENSMUSG00000062329
novel_circ_0002343	mmu-miR-709	ENSMUST00000073554	ENSMUSG00000062329
novel_circ_0002399	mmu-miR-709	ENSMUST00000073554	ENSMUSG00000062329
novel_circ_0002400	mmu-miR-709	ENSMUST00000073554	ENSMUSG00000062329
novel_circ_0002831	mmu-miR-709	ENSMUST00000073554	ENSMUSG00000062329
novel_circ_0002836	mmu-miR-709	ENSMUST00000073554	ENSMUSG00000062329
novel_circ_0002871	mmu-miR-709	ENSMUST00000073554	ENSMUSG00000062329
novel_circ_0002872	mmu-miR-709	ENSMUST00000073554	ENSMUSG00000062329
novel_circ_0002884	mmu-miR-709	ENSMUST00000073554	ENSMUSG00000062329
novel_circ_0002921	mmu-miR-709	ENSMUST00000073554	ENSMUSG00000062329
novel_circ_0002936	mmu-miR-709	ENSMUST00000073554	ENSMUSG00000062329
novel_circ_0002969	mmu-miR-709	ENSMUST00000073554	ENSMUSG00000062329
novel_circ_0002973	mmu-miR-709	ENSMUST00000073554	ENSMUSG00000062329
novel_circ_0002982	mmu-miR-709	ENSMUST00000073554	ENSMUSG00000062329
novel_circ_0002658	mmu-miR-709	ENSMUST00000073554	ENSMUSG00000062329
novel_circ_0002732	mmu-miR-709	ENSMUST00000073554	ENSMUSG00000062329
novel_circ_0002761	mmu-miR-709	ENSMUST00000073554	ENSMUSG00000062329
novel_circ_0002762	mmu-miR-709	ENSMUST00000073554	ENSMUSG00000062329
novel_circ_0002788	mmu-miR-709	ENSMUST00000073554	ENSMUSG00000062329
novel_circ_0003201	mmu-miR-709	ENSMUST00000073554	ENSMUSG00000062329
novel_circ_0003201	mmu-miR-709	ENSMUST00000073554	ENSMUSG00000062329
novel_circ_0003298	mmu-miR-709	ENSMUST00000073554	ENSMUSG00000062329
novel_circ_0003301	mmu-miR-709	ENSMUST00000073554	ENSMUSG00000062329
novel_circ_0003341	mmu-miR-709	ENSMUST00000073554	ENSMUSG00000062329
novel_circ_0003357	mmu-miR-709	ENSMUST00000073554	ENSMUSG00000062329
novel_circ_0003370	mmu-miR-709	ENSMUST00000073554	ENSMUSG00000062329
novel_circ_0003027	mmu-miR-709	ENSMUST00000073554	ENSMUSG00000062329
novel_circ_0003507	mmu-miR-709	ENSMUST00000073554	ENSMUSG00000062329
novel_circ_0003434	mmu-miR-709	ENSMUST00000073554	ENSMUSG00000062329
novel_circ_0000247	mmu-miR-709	ENSMUST00000073554	ENSMUSG00000062329
novel_circ_0000247	mmu-miR-709	ENSMUST00000073554	ENSMUSG00000062329
novel_circ_0000264	mmu-miR-709	ENSMUST00000073554	ENSMUSG00000062329
novel_circ_0000264	mmu-miR-709	ENSMUST00000073554	ENSMUSG00000062329
novel_circ_0000344	mmu-miR-709	ENSMUST00000073554	ENSMUSG00000062329
novel_circ_0000358	mmu-miR-709	ENSMUST00000073554	ENSMUSG00000062329
novel_circ_0000386	mmu-miR-709	ENSMUST00000073554	ENSMUSG00000062329
novel_circ_0000042	mmu-miR-709	ENSMUST00000073554	ENSMUSG00000062329
novel_circ_0000100	mmu-miR-709	ENSMUST00000073554	ENSMUSG00000062329
novel_circ_0000105	mmu-miR-709	ENSMUST00000073554	ENSMUSG00000062329
novel_circ_0000160	mmu-miR-709	ENSMUST00000073554	ENSMUSG00000062329
novel_circ_0000633	mmu-miR-709	ENSMUST00000073554	ENSMUSG00000062329
novel_circ_0000670	mmu-miR-709	ENSMUST00000073554	ENSMUSG00000062329
novel_circ_0000757	mmu-miR-709	ENSMUST00000073554	ENSMUSG00000062329
novel_circ_0000462	mmu-miR-709	ENSMUST00000073554	ENSMUSG00000062329
novel_circ_0000469	mmu-miR-709	ENSMUST00000073554	ENSMUSG00000062329
novel_circ_0000589	mmu-miR-709	ENSMUST00000073554	ENSMUSG00000062329
novel_circ_0000985	mmu-miR-709	ENSMUST00000073554	ENSMUSG00000062329
novel_circ_0001048	mmu-miR-709	ENSMUST00000073554	ENSMUSG00000062329
novel_circ_0000896	mmu-miR-709	ENSMUST00000073554	ENSMUSG00000062329
novel_circ_0000902	mmu-miR-709	ENSMUST00000073554	ENSMUSG00000062329
novel_circ_0000903	mmu-miR-709	ENSMUST00000073554	ENSMUSG00000062329
novel_circ_0001191	mmu-miR-709	ENSMUST00000073554	ENSMUSG00000062329
novel_circ_0001222	mmu-miR-297c-5	ENSMUST00000110698	ENSMUSG00000055737

novel_circ_0001306	mmu-miR-297c-5	ENSMUST00000110698	ENSMUSG00000055737
novel_circ_0001346	mmu-miR-297c-5	ENSMUST00000110698	ENSMUSG00000055737
mmu_circ_0001162	mmu-miR-297c-5	ENSMUST00000110698	ENSMUSG00000055737
mmu_circ_0001123	mmu-miR-297c-5	ENSMUST00000110698	ENSMUSG00000055737
mmu_circ_0001136	mmu-miR-297c-5	ENSMUST00000110698	ENSMUSG00000055737
mmu_circ_0001376	mmu-miR-297c-5	ENSMUST00000110698	ENSMUSG00000055737
mmu_circ_0001333	mmu-miR-297c-5	ENSMUST00000110698	ENSMUSG00000055737
mmu_circ_0001335	mmu-miR-297c-5	ENSMUST00000110698	ENSMUSG00000055737
mmu_circ_0001336	mmu-miR-297c-5	ENSMUST00000110698	ENSMUSG00000055737
mmu_circ_0001507	mmu-miR-297c-5	ENSMUST00000110698	ENSMUSG00000055737
mmu_circ_0001454	mmu-miR-297c-5	ENSMUST00000110698	ENSMUSG00000055737
mmu_circ_0001755	mmu-miR-297c-5	ENSMUST00000110698	ENSMUSG00000055737
mmu_circ_0001755	mmu-miR-297c-5	ENSMUST00000110698	ENSMUSG00000055737
mmu_circ_0001827	mmu-miR-297c-5	ENSMUST00000110698	ENSMUSG00000055737
mmu_circ_0001886	mmu-miR-297c-5	ENSMUST00000110698	ENSMUSG00000055737
mmu_circ_0000271	mmu-miR-297c-5	ENSMUST00000110698	ENSMUSG00000055737
mmu_circ_0000230	mmu-miR-297c-5	ENSMUST00000110698	ENSMUSG00000055737
mmu_circ_0000499	mmu-miR-297c-5	ENSMUST00000110698	ENSMUSG00000055737
mmu_circ_0000500	mmu-miR-297c-5	ENSMUST00000110698	ENSMUSG00000055737
mmu_circ_0000812	mmu-miR-297c-5	ENSMUST00000110698	ENSMUSG00000055737
mmu_circ_0000829	mmu-miR-297c-5	ENSMUST00000110698	ENSMUSG00000055737
mmu_circ_0000681	mmu-miR-297c-5	ENSMUST00000110698	ENSMUSG00000055737
mmu_circ_0000712	mmu-miR-297c-5	ENSMUST00000110698	ENSMUSG00000055737
mmu_circ_0000713	mmu-miR-297c-5	ENSMUST00000110698	ENSMUSG00000055737
mmu_circ_0000714	mmu-miR-297c-5	ENSMUST00000110698	ENSMUSG00000055737
mmu_circ_0000950	mmu-miR-297c-5	ENSMUST00000110698	ENSMUSG00000055737
mmu_circ_0000969	mmu-miR-297c-5	ENSMUST00000110698	ENSMUSG00000055737
mmu_circ_0000884	mmu-miR-297c-5	ENSMUST00000110698	ENSMUSG00000055737
mmu_circ_0000886	mmu-miR-297c-5	ENSMUST00000110698	ENSMUSG00000055737
mmu_circ_0000887	mmu-miR-297c-5	ENSMUST00000110698	ENSMUSG00000055737
mmu_circ_0000905	mmu-miR-297c-5	ENSMUST00000110698	ENSMUSG00000055737
mmu_circ_0000906	mmu-miR-297c-5	ENSMUST00000110698	ENSMUSG00000055737
mmu_circ_0000907	mmu-miR-297c-5	ENSMUST00000110698	ENSMUSG00000055737
mmu_circ_0000908	mmu-miR-297c-5	ENSMUST00000110698	ENSMUSG00000055737
novel_circ_0001680	mmu-miR-297c-5	ENSMUST00000110698	ENSMUSG00000055737
novel_circ_0001721	mmu-miR-297c-5	ENSMUST00000110698	ENSMUSG00000055737
novel_circ_0002082	mmu-miR-297c-5	ENSMUST00000110698	ENSMUSG00000055737
novel_circ_0001933	mmu-miR-297c-5	ENSMUST00000110698	ENSMUSG00000055737
novel_circ_0001934	mmu-miR-297c-5	ENSMUST00000110698	ENSMUSG00000055737
novel_circ_0002507	mmu-miR-297c-5	ENSMUST00000110698	ENSMUSG00000055737
novel_circ_0002508	mmu-miR-297c-5	ENSMUST00000110698	ENSMUSG00000055737
novel_circ_0002581	mmu-miR-297c-5	ENSMUST00000110698	ENSMUSG00000055737
novel_circ_0002374	mmu-miR-297c-5	ENSMUST00000110698	ENSMUSG00000055737
novel_circ_0002978	mmu-miR-297c-5	ENSMUST00000110698	ENSMUSG00000055737
novel_circ_0002990	mmu-miR-297c-5	ENSMUST00000110698	ENSMUSG00000055737
novel_circ_0002642	mmu-miR-297c-5	ENSMUST00000110698	ENSMUSG00000055737
novel_circ_0002695	mmu-miR-297c-5	ENSMUST00000110698	ENSMUSG00000055737
novel_circ_0002782	mmu-miR-297c-5	ENSMUST00000110698	ENSMUSG00000055737
novel_circ_0003016	mmu-miR-297c-5	ENSMUST00000110698	ENSMUSG00000055737
novel_circ_0000188	mmu-miR-297c-5	ENSMUST00000110698	ENSMUSG00000055737
novel_circ_0000268	mmu-miR-297c-5	ENSMUST00000110698	ENSMUSG00000055737
novel_circ_0000985	mmu-miR-297c-5	ENSMUST00000110698	ENSMUSG00000055737
novel_circ_0000807	mmu-miR-297c-5	ENSMUST00000110698	ENSMUSG00000055737
novel_circ_0001191	mmu-miR-297c-5	ENSMUST00000110698	ENSMUSG00000055737
novel_circ_0001191	mmu-miR-297c-5	ENSMUST00000110698	ENSMUSG00000055737
novel_circ_0001288	mmu-miR-504-5p	ENSMUST00000000128	ENSMUSG00000000126
novel_circ_0001288	mmu-miR-504-5p	ENSMUST00000108783	ENSMUSG00000000126
novel_circ_0001083	mmu-miR-504-5p	ENSMUST00000000128	ENSMUSG00000000126

novel_circ_0001083	mmu-miR-504-5p	ENSMUST00000108783	ENSMUSG00000000126
novel_circ_0001449	mmu-miR-504-5p	ENSMUST00000000128	ENSMUSG00000000126
novel_circ_0001449	mmu-miR-504-5p	ENSMUST00000108783	ENSMUSG00000000126
novel_circ_0001356	mmu-miR-504-5p	ENSMUST00000000128	ENSMUSG00000000126
novel_circ_0001356	mmu-miR-504-5p	ENSMUST00000108783	ENSMUSG00000000126
mmu_circ_0000075	mmu-miR-504-5p	ENSMUST00000000128	ENSMUSG00000000126
mmu_circ_0000075	mmu-miR-504-5p	ENSMUST00000108783	ENSMUSG00000000126
mmu_circ_0001123	mmu-miR-504-5p	ENSMUST00000000128	ENSMUSG00000000126
mmu_circ_0001123	mmu-miR-504-5p	ENSMUST00000108783	ENSMUSG00000000126
mmu_circ_0001376	mmu-miR-504-5p	ENSMUST00000000128	ENSMUSG00000000126
mmu_circ_0001376	mmu-miR-504-5p	ENSMUST00000108783	ENSMUSG00000000126
mmu_circ_0001271	mmu-miR-504-5p	ENSMUST00000000128	ENSMUSG00000000126
mmu_circ_0001271	mmu-miR-504-5p	ENSMUST00000108783	ENSMUSG00000000126
mmu_circ_0001624	mmu-miR-504-5p	ENSMUST00000000128	ENSMUSG00000000126
mmu_circ_0001624	mmu-miR-504-5p	ENSMUST00000108783	ENSMUSG00000000126
mmu_circ_0001625	mmu-miR-504-5p	ENSMUST00000000128	ENSMUSG00000000126
mmu_circ_0001625	mmu-miR-504-5p	ENSMUST00000108783	ENSMUSG00000000126
mmu_circ_0001570	mmu-miR-504-5p	ENSMUST00000000128	ENSMUSG00000000126
mmu_circ_0001570	mmu-miR-504-5p	ENSMUST00000108783	ENSMUSG00000000126
mmu_circ_0001454	mmu-miR-504-5p	ENSMUST00000000128	ENSMUSG00000000126
mmu_circ_0001454	mmu-miR-504-5p	ENSMUST00000108783	ENSMUSG00000000126
mmu_circ_0001469	mmu-miR-504-5p	ENSMUST00000000128	ENSMUSG00000000126
mmu_circ_0001469	mmu-miR-504-5p	ENSMUST00000108783	ENSMUSG00000000126
mmu_circ_0001500	mmu-miR-504-5p	ENSMUST00000000128	ENSMUSG00000000126
mmu_circ_0001500	mmu-miR-504-5p	ENSMUST00000108783	ENSMUSG00000000126
mmu_circ_0001743	mmu-miR-504-5p	ENSMUST00000000128	ENSMUSG00000000126
mmu_circ_0001743	mmu-miR-504-5p	ENSMUST00000108783	ENSMUSG00000000126
mmu_circ_0001827	mmu-miR-504-5p	ENSMUST00000000128	ENSMUSG00000000126
mmu_circ_0001827	mmu-miR-504-5p	ENSMUST00000108783	ENSMUSG00000000126
mmu_circ_0001676	mmu-miR-504-5p	ENSMUST00000000128	ENSMUSG00000000126
mmu_circ_0001676	mmu-miR-504-5p	ENSMUST00000108783	ENSMUSG00000000126
mmu_circ_0000333	mmu-miR-504-5p	ENSMUST00000000128	ENSMUSG00000000126
mmu_circ_0000333	mmu-miR-504-5p	ENSMUST00000108783	ENSMUSG00000000126
mmu_circ_0000499	mmu-miR-504-5p	ENSMUST00000000128	ENSMUSG00000000126
mmu_circ_0000499	mmu-miR-504-5p	ENSMUST00000108783	ENSMUSG00000000126
mmu_circ_0000465	mmu-miR-504-5p	ENSMUST00000000128	ENSMUSG00000000126
mmu_circ_0000465	mmu-miR-504-5p	ENSMUST00000108783	ENSMUSG00000000126
mmu_circ_0000577	mmu-miR-504-5p	ENSMUST00000000128	ENSMUSG00000000126
mmu_circ_0000577	mmu-miR-504-5p	ENSMUST00000108783	ENSMUSG00000000126
mmu_circ_0000530	mmu-miR-504-5p	ENSMUST00000000128	ENSMUSG00000000126
mmu_circ_0000530	mmu-miR-504-5p	ENSMUST00000108783	ENSMUSG00000000126
mmu_circ_0000735	mmu-miR-504-5p	ENSMUST00000000128	ENSMUSG00000000126
mmu_circ_0000735	mmu-miR-504-5p	ENSMUST00000108783	ENSMUSG00000000126
mmu_circ_0000798	mmu-miR-504-5p	ENSMUST00000000128	ENSMUSG00000000126
mmu_circ_0000798	mmu-miR-504-5p	ENSMUST00000108783	ENSMUSG00000000126
mmu_circ_0000828	mmu-miR-504-5p	ENSMUST00000000128	ENSMUSG00000000126
mmu_circ_0000828	mmu-miR-504-5p	ENSMUST00000108783	ENSMUSG00000000126
mmu_circ_0000829	mmu-miR-504-5p	ENSMUST00000000128	ENSMUSG00000000126
mmu_circ_0000829	mmu-miR-504-5p	ENSMUST00000108783	ENSMUSG00000000126
mmu_circ_0000831	mmu-miR-504-5p	ENSMUST00000000128	ENSMUSG00000000126
mmu_circ_0000831	mmu-miR-504-5p	ENSMUST00000108783	ENSMUSG00000000126
mmu_circ_0000712	mmu-miR-504-5p	ENSMUST00000000128	ENSMUSG00000000126
mmu_circ_0000712	mmu-miR-504-5p	ENSMUST00000108783	ENSMUSG00000000126
mmu_circ_0000950	mmu-miR-504-5p	ENSMUST00000000128	ENSMUSG00000000126
mmu_circ_0000950	mmu-miR-504-5p	ENSMUST00000108783	ENSMUSG00000000126
mmu_circ_0000969	mmu-miR-504-5p	ENSMUST00000000128	ENSMUSG00000000126
mmu_circ_0000969	mmu-miR-504-5p	ENSMUST00000108783	ENSMUSG00000000126
mmu_circ_0000884	mmu-miR-504-5p	ENSMUST00000000128	ENSMUSG00000000126

mmu_circ_0000884	mmu-miR-504-5p	ENSMUST00000108783	ENSMUSG00000000126
mmu_circ_0000886	mmu-miR-504-5p	ENSMUST00000000128	ENSMUSG00000000126
mmu_circ_0000886	mmu-miR-504-5p	ENSMUST00000108783	ENSMUSG00000000126
mmu_circ_0000906	mmu-miR-504-5p	ENSMUST00000000128	ENSMUSG00000000126
mmu_circ_0000906	mmu-miR-504-5p	ENSMUST00000108783	ENSMUSG00000000126
mmu_circ_0000912	mmu-miR-504-5p	ENSMUST00000000128	ENSMUSG00000000126
mmu_circ_0000912	mmu-miR-504-5p	ENSMUST00000108783	ENSMUSG00000000126
novel_circ_0001561	mmu-miR-504-5p	ENSMUST00000000128	ENSMUSG00000000126
novel_circ_0001561	mmu-miR-504-5p	ENSMUST00000108783	ENSMUSG00000000126
novel_circ_0001728	mmu-miR-504-5p	ENSMUST00000000128	ENSMUSG00000000126
novel_circ_0001728	mmu-miR-504-5p	ENSMUST00000108783	ENSMUSG00000000126
novel_circ_0001772	mmu-miR-504-5p	ENSMUST00000000128	ENSMUSG00000000126
novel_circ_0001772	mmu-miR-504-5p	ENSMUST00000108783	ENSMUSG00000000126
novel_circ_0001874	mmu-miR-504-5p	ENSMUST00000000128	ENSMUSG00000000126
novel_circ_0001874	mmu-miR-504-5p	ENSMUST00000108783	ENSMUSG00000000126
novel_circ_0002491	mmu-miR-504-5p	ENSMUST00000000128	ENSMUSG00000000126
novel_circ_0002491	mmu-miR-504-5p	ENSMUST00000108783	ENSMUSG00000000126
novel_circ_0002519	mmu-miR-504-5p	ENSMUST00000000128	ENSMUSG00000000126
novel_circ_0002519	mmu-miR-504-5p	ENSMUST00000108783	ENSMUSG00000000126
novel_circ_0002519	mmu-miR-504-5p	ENSMUST00000000128	ENSMUSG00000000126
novel_circ_0002519	mmu-miR-504-5p	ENSMUST00000108783	ENSMUSG00000000126
novel_circ_0002236	mmu-miR-504-5p	ENSMUST00000000128	ENSMUSG00000000126
novel_circ_0002236	mmu-miR-504-5p	ENSMUST00000108783	ENSMUSG00000000126
novel_circ_0002246	mmu-miR-504-5p	ENSMUST00000000128	ENSMUSG00000000126
novel_circ_0002246	mmu-miR-504-5p	ENSMUST00000108783	ENSMUSG00000000126
novel_circ_0002286	mmu-miR-504-5p	ENSMUST00000000128	ENSMUSG00000000126
novel_circ_0002286	mmu-miR-504-5p	ENSMUST00000108783	ENSMUSG00000000126
novel_circ_0002325	mmu-miR-504-5p	ENSMUST00000000128	ENSMUSG00000000126
novel_circ_0002325	mmu-miR-504-5p	ENSMUST00000108783	ENSMUSG00000000126
novel_circ_0002392	mmu-miR-504-5p	ENSMUST00000000128	ENSMUSG00000000126
novel_circ_0002392	mmu-miR-504-5p	ENSMUST00000108783	ENSMUSG00000000126
novel_circ_0002392	mmu-miR-504-5p	ENSMUST00000000128	ENSMUSG00000000126
novel_circ_0002392	mmu-miR-504-5p	ENSMUST00000108783	ENSMUSG00000000126
novel_circ_0002833	mmu-miR-504-5p	ENSMUST00000000128	ENSMUSG00000000126
novel_circ_0002833	mmu-miR-504-5p	ENSMUST00000108783	ENSMUSG00000000126
novel_circ_0002925	mmu-miR-504-5p	ENSMUST00000000128	ENSMUSG00000000126
novel_circ_0002925	mmu-miR-504-5p	ENSMUST00000108783	ENSMUSG00000000126
novel_circ_0002925	mmu-miR-504-5p	ENSMUST00000000128	ENSMUSG00000000126
novel_circ_0002925	mmu-miR-504-5p	ENSMUST00000108783	ENSMUSG00000000126
novel_circ_0002732	mmu-miR-504-5p	ENSMUST00000000128	ENSMUSG00000000126
novel_circ_0002732	mmu-miR-504-5p	ENSMUST00000108783	ENSMUSG00000000126
novel_circ_0003375	mmu-miR-504-5p	ENSMUST00000000128	ENSMUSG00000000126
novel_circ_0003375	mmu-miR-504-5p	ENSMUST00000108783	ENSMUSG00000000126
novel_circ_0003375	mmu-miR-504-5p	ENSMUST00000000128	ENSMUSG00000000126
novel_circ_0003375	mmu-miR-504-5p	ENSMUST00000108783	ENSMUSG00000000126
novel_circ_0003385	mmu-miR-504-5p	ENSMUST00000000128	ENSMUSG00000000126
novel_circ_0003385	mmu-miR-504-5p	ENSMUST00000108783	ENSMUSG00000000126
novel_circ_0003393	mmu-miR-504-5p	ENSMUST00000000128	ENSMUSG00000000126
novel_circ_0003393	mmu-miR-504-5p	ENSMUST00000108783	ENSMUSG00000000126
novel_circ_0003393	mmu-miR-504-5p	ENSMUST00000000128	ENSMUSG00000000126
novel_circ_0003393	mmu-miR-504-5p	ENSMUST00000108783	ENSMUSG00000000126
novel_circ_0003083	mmu-miR-504-5p	ENSMUST00000000128	ENSMUSG00000000126
novel_circ_0003083	mmu-miR-504-5p	ENSMUST00000108783	ENSMUSG00000000126
novel_circ_0000447	mmu-miR-504-5p	ENSMUST00000000128	ENSMUSG00000000126
novel_circ_0000447	mmu-miR-504-5p	ENSMUST00000108783	ENSMUSG00000000126
novel_circ_0000687	mmu-miR-504-5p	ENSMUST00000000128	ENSMUSG00000000126
novel_circ_0000687	mmu-miR-504-5p	ENSMUST00000108783	ENSMUSG00000000126
novel_circ_0000687	mmu-miR-504-5p	ENSMUST00000000128	ENSMUSG00000000126

novel_circ_0000687	mmu-miR-504-5p	ENSMUST00000108783	ENSMUSG00000000126
novel_circ_0000486	mmu-miR-504-5p	ENSMUST00000000128	ENSMUSG00000000126
novel_circ_0000486	mmu-miR-504-5p	ENSMUST00000108783	ENSMUSG00000000126
novel_circ_0000486	mmu-miR-504-5p	ENSMUST00000000128	ENSMUSG00000000126
novel_circ_0000486	mmu-miR-504-5p	ENSMUST00000108783	ENSMUSG00000000126
novel_circ_0000527	mmu-miR-504-5p	ENSMUST00000000128	ENSMUSG00000000126
novel_circ_0000527	mmu-miR-504-5p	ENSMUST00000108783	ENSMUSG00000000126
novel_circ_0000568	mmu-miR-504-5p	ENSMUST00000000128	ENSMUSG00000000126
novel_circ_0000568	mmu-miR-504-5p	ENSMUST00000108783	ENSMUSG00000000126
novel_circ_0000584	mmu-miR-504-5p	ENSMUST00000000128	ENSMUSG00000000126
novel_circ_0000584	mmu-miR-504-5p	ENSMUST00000108783	ENSMUSG00000000126
novel_circ_0000956	mmu-miR-504-5p	ENSMUST00000000128	ENSMUSG00000000126
novel_circ_0000956	mmu-miR-504-5p	ENSMUST00000108783	ENSMUSG00000000126
novel_circ_0001002	mmu-miR-504-5p	ENSMUST00000000128	ENSMUSG00000000126
novel_circ_0001002	mmu-miR-504-5p	ENSMUST00000108783	ENSMUSG00000000126
novel_circ_0000831	mmu-miR-504-5p	ENSMUST00000000128	ENSMUSG00000000126
novel_circ_0000831	mmu-miR-504-5p	ENSMUST00000108783	ENSMUSG00000000126
novel_circ_0000832	mmu-miR-504-5p	ENSMUST00000000128	ENSMUSG00000000126
novel_circ_0000832	mmu-miR-504-5p	ENSMUST00000108783	ENSMUSG00000000126
novel_circ_0000837	mmu-miR-504-5p	ENSMUST00000000128	ENSMUSG00000000126
novel_circ_0000837	mmu-miR-504-5p	ENSMUST00000108783	ENSMUSG00000000126
novel_circ_0001177	mmu-miR-504-5p	ENSMUST00000000128	ENSMUSG00000000126
novel_circ_0001177	mmu-miR-504-5p	ENSMUST00000108783	ENSMUSG00000000126
novel_circ_0001179	mmu-miR-504-5p	ENSMUST00000000128	ENSMUSG00000000126
novel_circ_0001179	mmu-miR-504-5p	ENSMUST00000108783	ENSMUSG00000000126
novel_circ_0001292	mmu-miR-466i-5p	ENSMUST00000073554	ENSMUSG00000062329
novel_circ_0001110	mmu-miR-466i-5p	ENSMUST00000073554	ENSMUSG00000062329
novel_circ_0001471	mmu-miR-466i-5p	ENSMUST00000073554	ENSMUSG00000062329
novel_circ_0001533	mmu-miR-466i-5p	ENSMUST00000073554	ENSMUSG00000062329
novel_circ_0001346	mmu-miR-466i-5p	ENSMUST00000073554	ENSMUSG00000062329
mmu_circ_0000129	mmu-miR-466i-5p	ENSMUST00000073554	ENSMUSG00000062329
mmu_circ_0000028	mmu-miR-466i-5p	ENSMUST00000073554	ENSMUSG00000062329
mmu_circ_0000049	mmu-miR-466i-5p	ENSMUST00000073554	ENSMUSG00000062329
mmu_circ_0000063	mmu-miR-466i-5p	ENSMUST00000073554	ENSMUSG00000062329
mmu_circ_0001123	mmu-miR-466i-5p	ENSMUST00000073554	ENSMUSG00000062329
mmu_circ_0001369	mmu-miR-466i-5p	ENSMUST00000073554	ENSMUSG00000062329
mmu_circ_0001376	mmu-miR-466i-5p	ENSMUST00000073554	ENSMUSG00000062329
mmu_circ_0001333	mmu-miR-466i-5p	ENSMUST00000073554	ENSMUSG00000062329
mmu_circ_0001646	mmu-miR-466i-5p	ENSMUST00000073554	ENSMUSG00000062329
mmu_circ_0001646	mmu-miR-466i-5p	ENSMUST00000073554	ENSMUSG00000062329
mmu_circ_0001528	mmu-miR-466i-5p	ENSMUST00000073554	ENSMUSG00000062329
mmu_circ_0001454	mmu-miR-466i-5p	ENSMUST00000073554	ENSMUSG00000062329
mmu_circ_0001482	mmu-miR-466i-5p	ENSMUST00000073554	ENSMUSG00000062329
mmu_circ_0001500	mmu-miR-466i-5p	ENSMUST00000073554	ENSMUSG00000062329
mmu_circ_0001751	mmu-miR-466i-5p	ENSMUST00000073554	ENSMUSG00000062329
mmu_circ_0001827	mmu-miR-466i-5p	ENSMUST00000073554	ENSMUSG00000062329
mmu_circ_0000301	mmu-miR-466i-5p	ENSMUST00000073554	ENSMUSG00000062329
mmu_circ_0000499	mmu-miR-466i-5p	ENSMUST00000073554	ENSMUSG00000062329
mmu_circ_0000500	mmu-miR-466i-5p	ENSMUST00000073554	ENSMUSG00000062329
mmu_circ_0000399	mmu-miR-466i-5p	ENSMUST00000073554	ENSMUSG00000062329
mmu_circ_0000530	mmu-miR-466i-5p	ENSMUST00000073554	ENSMUSG00000062329
mmu_circ_0000829	mmu-miR-466i-5p	ENSMUST00000073554	ENSMUSG00000062329
mmu_circ_0000831	mmu-miR-466i-5p	ENSMUST00000073554	ENSMUSG00000062329
mmu_circ_0000712	mmu-miR-466i-5p	ENSMUST00000073554	ENSMUSG00000062329
mmu_circ_0000713	mmu-miR-466i-5p	ENSMUST00000073554	ENSMUSG00000062329
mmu_circ_0000714	mmu-miR-466i-5p	ENSMUST00000073554	ENSMUSG00000062329
mmu_circ_0000950	mmu-miR-466i-5p	ENSMUST00000073554	ENSMUSG00000062329
mmu_circ_0000969	mmu-miR-466i-5p	ENSMUST00000073554	ENSMUSG00000062329

mmu_circ_0000884	mmu-miR-466i-5p	ENSMUST00000073554	ENSMUSG00000062329
mmu_circ_0000886	mmu-miR-466i-5p	ENSMUST00000073554	ENSMUSG00000062329
mmu_circ_0000887	mmu-miR-466i-5p	ENSMUST00000073554	ENSMUSG00000062329
mmu_circ_0000905	mmu-miR-466i-5p	ENSMUST00000073554	ENSMUSG00000062329
mmu_circ_0000906	mmu-miR-466i-5p	ENSMUST00000073554	ENSMUSG00000062329
mmu_circ_0000912	mmu-miR-466i-5p	ENSMUST00000073554	ENSMUSG00000062329
novel_circ_0001892	mmu-miR-466i-5p	ENSMUST00000073554	ENSMUSG00000062329
novel_circ_0002028	mmu-miR-466i-5p	ENSMUST00000073554	ENSMUSG00000062329
novel_circ_0002046	mmu-miR-466i-5p	ENSMUST00000073554	ENSMUSG00000062329
novel_circ_0002506	mmu-miR-466i-5p	ENSMUST00000073554	ENSMUSG00000062329
novel_circ_0002518	mmu-miR-466i-5p	ENSMUST00000073554	ENSMUSG00000062329
novel_circ_0002556	mmu-miR-466i-5p	ENSMUST00000073554	ENSMUSG00000062329
novel_circ_0002597	mmu-miR-466i-5p	ENSMUST00000073554	ENSMUSG00000062329
novel_circ_0002232	mmu-miR-466i-5p	ENSMUST00000073554	ENSMUSG00000062329
novel_circ_0002232	mmu-miR-466i-5p	ENSMUST00000073554	ENSMUSG00000062329
novel_circ_0002260	mmu-miR-466i-5p	ENSMUST00000073554	ENSMUSG00000062329
novel_circ_0002352	mmu-miR-466i-5p	ENSMUST00000073554	ENSMUSG00000062329
novel_circ_0002831	mmu-miR-466i-5p	ENSMUST00000073554	ENSMUSG00000062329
novel_circ_0002900	mmu-miR-466i-5p	ENSMUST00000073554	ENSMUSG00000062329
novel_circ_0002923	mmu-miR-466i-5p	ENSMUST00000073554	ENSMUSG00000062329
novel_circ_0002925	mmu-miR-466i-5p	ENSMUST00000073554	ENSMUSG00000062329
novel_circ_0002934	mmu-miR-466i-5p	ENSMUST00000073554	ENSMUSG00000062329
novel_circ_0002966	mmu-miR-466i-5p	ENSMUST00000073554	ENSMUSG00000062329
novel_circ_0002631	mmu-miR-466i-5p	ENSMUST00000073554	ENSMUSG00000062329
novel_circ_0002653	mmu-miR-466i-5p	ENSMUST00000073554	ENSMUSG00000062329
novel_circ_0002752	mmu-miR-466i-5p	ENSMUST00000073554	ENSMUSG00000062329
novel_circ_0002775	mmu-miR-466i-5p	ENSMUST00000073554	ENSMUSG00000062329
novel_circ_0002778	mmu-miR-466i-5p	ENSMUST00000073554	ENSMUSG00000062329
novel_circ_0002780	mmu-miR-466i-5p	ENSMUST00000073554	ENSMUSG00000062329
novel_circ_0003229	mmu-miR-466i-5p	ENSMUST00000073554	ENSMUSG00000062329
novel_circ_0003288	mmu-miR-466i-5p	ENSMUST00000073554	ENSMUSG00000062329
novel_circ_0003326	mmu-miR-466i-5p	ENSMUST00000073554	ENSMUSG00000062329
novel_circ_0003370	mmu-miR-466i-5p	ENSMUST00000073554	ENSMUSG00000062329
novel_circ_0003027	mmu-miR-466i-5p	ENSMUST00000073554	ENSMUSG00000062329
novel_circ_0003433	mmu-miR-466i-5p	ENSMUST00000073554	ENSMUSG00000062329
novel_circ_0000193	mmu-miR-466i-5p	ENSMUST00000073554	ENSMUSG00000062329
novel_circ_0000358	mmu-miR-466i-5p	ENSMUST00000073554	ENSMUSG00000062329
novel_circ_0000381	mmu-miR-466i-5p	ENSMUST00000073554	ENSMUSG00000062329
novel_circ_0000381	mmu-miR-466i-5p	ENSMUST00000073554	ENSMUSG00000062329
novel_circ_0000116	mmu-miR-466i-5p	ENSMUST00000073554	ENSMUSG00000062329
novel_circ_0000160	mmu-miR-466i-5p	ENSMUST00000073554	ENSMUSG00000062329
novel_circ_0000160	mmu-miR-466i-5p	ENSMUST00000073554	ENSMUSG00000062329
novel_circ_0000740	mmu-miR-466i-5p	ENSMUST00000073554	ENSMUSG00000062329
novel_circ_0000515	mmu-miR-466i-5p	ENSMUST00000073554	ENSMUSG00000062329
novel_circ_0000572	mmu-miR-466i-5p	ENSMUST00000073554	ENSMUSG00000062329
novel_circ_0001186	mmu-miR-466i-5p	ENSMUST00000073554	ENSMUSG00000062329
novel_circ_0001207	mmu-miR-466i-5p	ENSMUST00000073554	ENSMUSG00000062329
novel_circ_0001208	mmu-miR-466i-5p	ENSMUST00000073554	ENSMUSG00000062329
novel_circ_0001450	mmu-miR-669e-3	ENSMUST00000000128	ENSMUSG00000000126
novel_circ_0001450	mmu-miR-669e-3	ENSMUST00000108783	ENSMUSG00000000126
novel_circ_0001451	mmu-miR-669e-3	ENSMUST00000000128	ENSMUSG00000000126
novel_circ_0001451	mmu-miR-669e-3	ENSMUST00000108783	ENSMUSG00000000126
mmu_circ_0001123	mmu-miR-669e-3	ENSMUST00000000128	ENSMUSG00000000126
mmu_circ_0001123	mmu-miR-669e-3	ENSMUST00000108783	ENSMUSG00000000126
mmu_circ_0000230	mmu-miR-669e-3	ENSMUST00000000128	ENSMUSG00000000126
mmu_circ_0000230	mmu-miR-669e-3	ENSMUST00000108783	ENSMUSG00000000126
mmu_circ_0000499	mmu-miR-669e-3	ENSMUST00000000128	ENSMUSG00000000126
mmu_circ_0000499	mmu-miR-669e-3	ENSMUST00000108783	ENSMUSG00000000126

mmu_circ_0000714	mmu-miR-669e-3	ENSMUST00000000128	ENSMUSG00000000126
mmu_circ_0000714	mmu-miR-669e-3	ENSMUST00000108783	ENSMUSG00000000126
mmu_circ_0000950	mmu-miR-669e-3	ENSMUST00000000128	ENSMUSG00000000126
mmu_circ_0000950	mmu-miR-669e-3	ENSMUST00000108783	ENSMUSG00000000126
mmu_circ_0000969	mmu-miR-669e-3	ENSMUST00000000128	ENSMUSG00000000126
mmu_circ_0000969	mmu-miR-669e-3	ENSMUST00000108783	ENSMUSG00000000126
novel_circ_0002289	mmu-miR-669e-3	ENSMUST00000000128	ENSMUSG00000000126
novel_circ_0002289	mmu-miR-669e-3	ENSMUST00000108783	ENSMUSG00000000126
novel_circ_0002831	mmu-miR-669e-3	ENSMUST00000000128	ENSMUSG00000000126
novel_circ_0002831	mmu-miR-669e-3	ENSMUST00000108783	ENSMUSG00000000126
novel_circ_0000406	mmu-miR-669e-3	ENSMUST00000000128	ENSMUSG00000000126
novel_circ_0000406	mmu-miR-669e-3	ENSMUST00000108783	ENSMUSG00000000126
novel_circ_0000985	mmu-miR-669e-3	ENSMUST00000000128	ENSMUSG00000000126
novel_circ_0000985	mmu-miR-669e-3	ENSMUST00000108783	ENSMUSG00000000126
novel_circ_0001022	mmu-miR-669e-3	ENSMUST00000000128	ENSMUSG00000000126
novel_circ_0001022	mmu-miR-669e-3	ENSMUST00000108783	ENSMUSG00000000126
novel_circ_0001349	mmu-miR-1195	ENSMUST00000223576	ENSMUSG00000017491
novel_circ_0001413	mmu-miR-1195	ENSMUST00000223576	ENSMUSG00000017491
mmu_circ_0000115	mmu-miR-1195	ENSMUST00000223576	ENSMUSG00000017491
mmu_circ_0000042	mmu-miR-1195	ENSMUST00000223576	ENSMUSG00000017491
mmu_circ_0001123	mmu-miR-1195	ENSMUST00000223576	ENSMUSG00000017491
mmu_circ_0001561	mmu-miR-1195	ENSMUST00000223576	ENSMUSG00000017491
mmu_circ_0001886	mmu-miR-1195	ENSMUST00000223576	ENSMUSG00000017491
mmu_circ_0000499	mmu-miR-1195	ENSMUST00000223576	ENSMUSG00000017491
mmu_circ_0000500	mmu-miR-1195	ENSMUST00000223576	ENSMUSG00000017491
mmu_circ_0000712	mmu-miR-1195	ENSMUST00000223576	ENSMUSG00000017491
mmu_circ_0000950	mmu-miR-1195	ENSMUST00000223576	ENSMUSG00000017491
mmu_circ_0000884	mmu-miR-1195	ENSMUST00000223576	ENSMUSG00000017491
mmu_circ_0000886	mmu-miR-1195	ENSMUST00000223576	ENSMUSG00000017491
mmu_circ_0000887	mmu-miR-1195	ENSMUST00000223576	ENSMUSG00000017491
mmu_circ_0000905	mmu-miR-1195	ENSMUST00000223576	ENSMUSG00000017491
mmu_circ_0000912	mmu-miR-1195	ENSMUST00000223576	ENSMUSG00000017491
novel_circ_0001726	mmu-miR-1195	ENSMUST00000223576	ENSMUSG00000017491
novel_circ_0002262	mmu-miR-1195	ENSMUST00000223576	ENSMUSG00000017491
novel_circ_0002262	mmu-miR-1195	ENSMUST00000223576	ENSMUSG00000017491
novel_circ_0002952	mmu-miR-1195	ENSMUST00000223576	ENSMUSG00000017491
novel_circ_0002952	mmu-miR-1195	ENSMUST00000223576	ENSMUSG00000017491
novel_circ_0002978	mmu-miR-1195	ENSMUST00000223576	ENSMUSG00000017491
novel_circ_0002978	mmu-miR-1195	ENSMUST00000223576	ENSMUSG00000017491
novel_circ_0002653	mmu-miR-1195	ENSMUST00000223576	ENSMUSG00000017491
novel_circ_0000218	mmu-miR-1195	ENSMUST00000223576	ENSMUSG00000017491
novel_circ_0001464	mmu-miR-1934-5	ENSMUST00000003659	ENSMUSG00000031849
novel_circ_0001464	mmu-miR-1934-5	ENSMUST00000073554	ENSMUSG00000062329
novel_circ_0001464	mmu-miR-1934-5	ENSMUST00000212488	ENSMUSG00000031849
novel_circ_0001464	mmu-miR-1934-5	ENSMUST00000003659	ENSMUSG00000031849
novel_circ_0001464	mmu-miR-1934-5	ENSMUST00000073554	ENSMUSG00000062329
novel_circ_0001464	mmu-miR-1934-5	ENSMUST00000212488	ENSMUSG00000031849
novel_circ_0001471	mmu-miR-1934-5	ENSMUST00000003659	ENSMUSG00000031849
novel_circ_0001471	mmu-miR-1934-5	ENSMUST00000073554	ENSMUSG00000062329
novel_circ_0001471	mmu-miR-1934-5	ENSMUST00000212488	ENSMUSG00000031849
novel_circ_0001349	mmu-miR-1934-5	ENSMUST00000003659	ENSMUSG00000031849
novel_circ_0001349	mmu-miR-1934-5	ENSMUST00000073554	ENSMUSG00000062329
novel_circ_0001349	mmu-miR-1934-5	ENSMUST00000212488	ENSMUSG00000031849
novel_circ_0001358	mmu-miR-1934-5	ENSMUST00000003659	ENSMUSG00000031849
novel_circ_0001358	mmu-miR-1934-5	ENSMUST00000073554	ENSMUSG00000062329
novel_circ_0001358	mmu-miR-1934-5	ENSMUST00000212488	ENSMUSG00000031849
novel_circ_0001380	mmu-miR-1934-5	ENSMUST00000003659	ENSMUSG00000031849
novel_circ_0001380	mmu-miR-1934-5	ENSMUST00000073554	ENSMUSG00000062329

novel_circ_0001380	mmu-miR-1934-5	ENSMUST00000212488	ENSMUSG00000031849
novel_circ_0001382	mmu-miR-1934-5	ENSMUST00000003659	ENSMUSG00000031849
novel_circ_0001382	mmu-miR-1934-5	ENSMUST00000073554	ENSMUSG00000062329
novel_circ_0001382	mmu-miR-1934-5	ENSMUST00000212488	ENSMUSG00000031849
novel_circ_0001395	mmu-miR-1934-5	ENSMUST00000003659	ENSMUSG00000031849
novel_circ_0001395	mmu-miR-1934-5	ENSMUST00000073554	ENSMUSG00000062329
novel_circ_0001395	mmu-miR-1934-5	ENSMUST00000212488	ENSMUSG00000031849
novel_circ_0001416	mmu-miR-1934-5	ENSMUST00000003659	ENSMUSG00000031849
novel_circ_0001416	mmu-miR-1934-5	ENSMUST00000073554	ENSMUSG00000062329
novel_circ_0001416	mmu-miR-1934-5	ENSMUST00000212488	ENSMUSG00000031849
novel_circ_0001416	mmu-miR-1934-5	ENSMUST00000003659	ENSMUSG00000031849
novel_circ_0001416	mmu-miR-1934-5	ENSMUST00000073554	ENSMUSG00000062329
novel_circ_0001416	mmu-miR-1934-5	ENSMUST00000212488	ENSMUSG00000031849
mmu_circ_0000094	mmu-miR-1934-5	ENSMUST00000003659	ENSMUSG00000031849
mmu_circ_0000094	mmu-miR-1934-5	ENSMUST00000073554	ENSMUSG00000062329
mmu_circ_0000094	mmu-miR-1934-5	ENSMUST00000212488	ENSMUSG00000031849
mmu_circ_0000028	mmu-miR-1934-5	ENSMUST00000003659	ENSMUSG00000031849
mmu_circ_0000028	mmu-miR-1934-5	ENSMUST00000073554	ENSMUSG00000062329
mmu_circ_0000028	mmu-miR-1934-5	ENSMUST00000212488	ENSMUSG00000031849
mmu_circ_0000042	mmu-miR-1934-5	ENSMUST00000003659	ENSMUSG00000031849
mmu_circ_0000042	mmu-miR-1934-5	ENSMUST00000073554	ENSMUSG00000062329
mmu_circ_0000042	mmu-miR-1934-5	ENSMUST00000212488	ENSMUSG00000031849
mmu_circ_0000063	mmu-miR-1934-5	ENSMUST00000003659	ENSMUSG00000031849
mmu_circ_0000063	mmu-miR-1934-5	ENSMUST00000073554	ENSMUSG00000062329
mmu_circ_0000063	mmu-miR-1934-5	ENSMUST00000212488	ENSMUSG00000031849
mmu_circ_0000992	mmu-miR-1934-5	ENSMUST00000003659	ENSMUSG00000031849
mmu_circ_0000992	mmu-miR-1934-5	ENSMUST00000073554	ENSMUSG00000062329
mmu_circ_0000992	mmu-miR-1934-5	ENSMUST00000212488	ENSMUSG00000031849
mmu_circ_0001049	mmu-miR-1934-5	ENSMUST00000003659	ENSMUSG00000031849
mmu_circ_0001049	mmu-miR-1934-5	ENSMUST00000073554	ENSMUSG00000062329
mmu_circ_0001049	mmu-miR-1934-5	ENSMUST00000212488	ENSMUSG00000031849
mmu_circ_0001376	mmu-miR-1934-5	ENSMUST00000003659	ENSMUSG00000031849
mmu_circ_0001376	mmu-miR-1934-5	ENSMUST00000073554	ENSMUSG00000062329
mmu_circ_0001376	mmu-miR-1934-5	ENSMUST00000212488	ENSMUSG00000031849
mmu_circ_0001333	mmu-miR-1934-5	ENSMUST00000003659	ENSMUSG00000031849
mmu_circ_0001333	mmu-miR-1934-5	ENSMUST00000073554	ENSMUSG00000062329
mmu_circ_0001333	mmu-miR-1934-5	ENSMUST00000212488	ENSMUSG00000031849
mmu_circ_0001263	mmu-miR-1934-5	ENSMUST00000003659	ENSMUSG00000031849
mmu_circ_0001263	mmu-miR-1934-5	ENSMUST00000073554	ENSMUSG00000062329
mmu_circ_0001263	mmu-miR-1934-5	ENSMUST00000212488	ENSMUSG00000031849
mmu_circ_0001264	mmu-miR-1934-5	ENSMUST00000003659	ENSMUSG00000031849
mmu_circ_0001264	mmu-miR-1934-5	ENSMUST00000073554	ENSMUSG00000062329
mmu_circ_0001264	mmu-miR-1934-5	ENSMUST00000212488	ENSMUSG00000031849
mmu_circ_0001291	mmu-miR-1934-5	ENSMUST00000003659	ENSMUSG00000031849
mmu_circ_0001291	mmu-miR-1934-5	ENSMUST00000073554	ENSMUSG00000062329
mmu_circ_0001291	mmu-miR-1934-5	ENSMUST00000212488	ENSMUSG00000031849
mmu_circ_0001636	mmu-miR-1934-5	ENSMUST00000003659	ENSMUSG00000031849
mmu_circ_0001636	mmu-miR-1934-5	ENSMUST00000073554	ENSMUSG00000062329
mmu_circ_0001636	mmu-miR-1934-5	ENSMUST00000212488	ENSMUSG00000031849
mmu_circ_0001528	mmu-miR-1934-5	ENSMUST00000003659	ENSMUSG00000031849
mmu_circ_0001528	mmu-miR-1934-5	ENSMUST00000073554	ENSMUSG00000062329
mmu_circ_0001528	mmu-miR-1934-5	ENSMUST00000212488	ENSMUSG00000031849
mmu_circ_0001454	mmu-miR-1934-5	ENSMUST00000003659	ENSMUSG00000031849
mmu_circ_0001454	mmu-miR-1934-5	ENSMUST00000073554	ENSMUSG00000062329
mmu_circ_0001454	mmu-miR-1934-5	ENSMUST00000212488	ENSMUSG00000031849
mmu_circ_0001491	mmu-miR-1934-5	ENSMUST00000003659	ENSMUSG00000031849
mmu_circ_0001491	mmu-miR-1934-5	ENSMUST00000073554	ENSMUSG00000062329
mmu_circ_0001491	mmu-miR-1934-5	ENSMUST00000212488	ENSMUSG00000031849

mmu_circ_0001500	mmu-miR-1934-5	ENSMUST00000003659	ENSMUSG00000031849
mmu_circ_0001500	mmu-miR-1934-5	ENSMUST00000073554	ENSMUSG00000062329
mmu_circ_0001500	mmu-miR-1934-5	ENSMUST00000212488	ENSMUSG00000031849
mmu_circ_0001842	mmu-miR-1934-5	ENSMUST00000003659	ENSMUSG00000031849
mmu_circ_0001842	mmu-miR-1934-5	ENSMUST00000073554	ENSMUSG00000062329
mmu_circ_0001842	mmu-miR-1934-5	ENSMUST00000212488	ENSMUSG00000031849
mmu_circ_0001767	mmu-miR-1934-5	ENSMUST00000003659	ENSMUSG00000031849
mmu_circ_0001767	mmu-miR-1934-5	ENSMUST00000073554	ENSMUSG00000062329
mmu_circ_0001767	mmu-miR-1934-5	ENSMUST00000212488	ENSMUSG00000031849
mmu_circ_0001767	mmu-miR-1934-5	ENSMUST00000003659	ENSMUSG00000031849
mmu_circ_0001767	mmu-miR-1934-5	ENSMUST00000073554	ENSMUSG00000062329
mmu_circ_0001767	mmu-miR-1934-5	ENSMUST00000212488	ENSMUSG00000031849
mmu_circ_0001795	mmu-miR-1934-5	ENSMUST00000003659	ENSMUSG00000031849
mmu_circ_0001795	mmu-miR-1934-5	ENSMUST00000073554	ENSMUSG00000062329
mmu_circ_0001795	mmu-miR-1934-5	ENSMUST00000212488	ENSMUSG00000031849
mmu_circ_0001829	mmu-miR-1934-5	ENSMUST00000003659	ENSMUSG00000031849
mmu_circ_0001829	mmu-miR-1934-5	ENSMUST00000073554	ENSMUSG00000062329
mmu_circ_0001829	mmu-miR-1934-5	ENSMUST00000212488	ENSMUSG00000031849
mmu_circ_0001830	mmu-miR-1934-5	ENSMUST00000003659	ENSMUSG00000031849
mmu_circ_0001830	mmu-miR-1934-5	ENSMUST00000073554	ENSMUSG00000062329
mmu_circ_0001830	mmu-miR-1934-5	ENSMUST00000212488	ENSMUSG00000031849
mmu_circ_0001898	mmu-miR-1934-5	ENSMUST00000003659	ENSMUSG00000031849
mmu_circ_0001898	mmu-miR-1934-5	ENSMUST00000073554	ENSMUSG00000062329
mmu_circ_0001898	mmu-miR-1934-5	ENSMUST00000212488	ENSMUSG00000031849
mmu_circ_0000344	mmu-miR-1934-5	ENSMUST00000003659	ENSMUSG00000031849
mmu_circ_0000344	mmu-miR-1934-5	ENSMUST00000073554	ENSMUSG00000062329
mmu_circ_0000344	mmu-miR-1934-5	ENSMUST00000212488	ENSMUSG00000031849
mmu_circ_0000215	mmu-miR-1934-5	ENSMUST00000003659	ENSMUSG00000031849
mmu_circ_0000215	mmu-miR-1934-5	ENSMUST00000073554	ENSMUSG00000062329
mmu_circ_0000215	mmu-miR-1934-5	ENSMUST00000212488	ENSMUSG00000031849
mmu_circ_0000148	mmu-miR-1934-5	ENSMUST00000003659	ENSMUSG00000031849
mmu_circ_0000148	mmu-miR-1934-5	ENSMUST00000073554	ENSMUSG00000062329
mmu_circ_0000148	mmu-miR-1934-5	ENSMUST00000212488	ENSMUSG00000031849
mmu_circ_0000149	mmu-miR-1934-5	ENSMUST00000003659	ENSMUSG00000031849
mmu_circ_0000149	mmu-miR-1934-5	ENSMUST00000073554	ENSMUSG00000062329
mmu_circ_0000149	mmu-miR-1934-5	ENSMUST00000212488	ENSMUSG00000031849
mmu_circ_0000499	mmu-miR-1934-5	ENSMUST00000003659	ENSMUSG00000031849
mmu_circ_0000499	mmu-miR-1934-5	ENSMUST00000073554	ENSMUSG00000062329
mmu_circ_0000499	mmu-miR-1934-5	ENSMUST00000212488	ENSMUSG00000031849
mmu_circ_0000345	mmu-miR-1934-5	ENSMUST00000003659	ENSMUSG00000031849
mmu_circ_0000345	mmu-miR-1934-5	ENSMUST00000073554	ENSMUSG00000062329
mmu_circ_0000345	mmu-miR-1934-5	ENSMUST00000212488	ENSMUSG00000031849
mmu_circ_0000399	mmu-miR-1934-5	ENSMUST00000003659	ENSMUSG00000031849
mmu_circ_0000399	mmu-miR-1934-5	ENSMUST00000073554	ENSMUSG00000062329
mmu_circ_0000399	mmu-miR-1934-5	ENSMUST00000212488	ENSMUSG00000031849
mmu_circ_0000623	mmu-miR-1934-5	ENSMUST00000003659	ENSMUSG00000031849
mmu_circ_0000623	mmu-miR-1934-5	ENSMUST00000073554	ENSMUSG00000062329
mmu_circ_0000623	mmu-miR-1934-5	ENSMUST00000212488	ENSMUSG00000031849
mmu_circ_0000510	mmu-miR-1934-5	ENSMUST00000003659	ENSMUSG00000031849
mmu_circ_0000510	mmu-miR-1934-5	ENSMUST00000073554	ENSMUSG00000062329
mmu_circ_0000510	mmu-miR-1934-5	ENSMUST00000212488	ENSMUSG00000031849
mmu_circ_0000522	mmu-miR-1934-5	ENSMUST00000003659	ENSMUSG00000031849
mmu_circ_0000522	mmu-miR-1934-5	ENSMUST00000073554	ENSMUSG00000062329
mmu_circ_0000522	mmu-miR-1934-5	ENSMUST00000212488	ENSMUSG00000031849
mmu_circ_0000530	mmu-miR-1934-5	ENSMUST00000003659	ENSMUSG00000031849
mmu_circ_0000530	mmu-miR-1934-5	ENSMUST00000073554	ENSMUSG00000062329
mmu_circ_0000530	mmu-miR-1934-5	ENSMUST00000212488	ENSMUSG00000031849
mmu_circ_0000714	mmu-miR-1934-5	ENSMUST00000003659	ENSMUSG00000031849

mmu_circ_0000714	mmu-miR-1934-5	ENSMUST00000073554	ENSMUSG00000062329
mmu_circ_0000714	mmu-miR-1934-5	ENSMUST00000212488	ENSMUSG00000031849
mmu_circ_0000969	mmu-miR-1934-5	ENSMUST00000003659	ENSMUSG00000031849
mmu_circ_0000969	mmu-miR-1934-5	ENSMUST00000073554	ENSMUSG00000062329
mmu_circ_0000969	mmu-miR-1934-5	ENSMUST00000212488	ENSMUSG00000031849
mmu_circ_0000874	mmu-miR-1934-5	ENSMUST00000003659	ENSMUSG00000031849
mmu_circ_0000874	mmu-miR-1934-5	ENSMUST00000073554	ENSMUSG00000062329
mmu_circ_0000874	mmu-miR-1934-5	ENSMUST00000212488	ENSMUSG00000031849
mmu_circ_0000884	mmu-miR-1934-5	ENSMUST00000003659	ENSMUSG00000031849
mmu_circ_0000884	mmu-miR-1934-5	ENSMUST00000073554	ENSMUSG00000062329
mmu_circ_0000884	mmu-miR-1934-5	ENSMUST00000212488	ENSMUSG00000031849
mmu_circ_0000886	mmu-miR-1934-5	ENSMUST00000003659	ENSMUSG00000031849
mmu_circ_0000886	mmu-miR-1934-5	ENSMUST00000073554	ENSMUSG00000062329
mmu_circ_0000886	mmu-miR-1934-5	ENSMUST00000212488	ENSMUSG00000031849
mmu_circ_0000903	mmu-miR-1934-5	ENSMUST00000003659	ENSMUSG00000031849
mmu_circ_0000903	mmu-miR-1934-5	ENSMUST00000073554	ENSMUSG00000062329
mmu_circ_0000903	mmu-miR-1934-5	ENSMUST00000212488	ENSMUSG00000031849
mmu_circ_0000906	mmu-miR-1934-5	ENSMUST00000003659	ENSMUSG00000031849
mmu_circ_0000906	mmu-miR-1934-5	ENSMUST00000073554	ENSMUSG00000062329
mmu_circ_0000906	mmu-miR-1934-5	ENSMUST00000212488	ENSMUSG00000031849
mmu_circ_0000907	mmu-miR-1934-5	ENSMUST00000003659	ENSMUSG00000031849
mmu_circ_0000907	mmu-miR-1934-5	ENSMUST00000073554	ENSMUSG00000062329
mmu_circ_0000907	mmu-miR-1934-5	ENSMUST00000212488	ENSMUSG00000031849
mmu_circ_0000908	mmu-miR-1934-5	ENSMUST00000003659	ENSMUSG00000031849
mmu_circ_0000908	mmu-miR-1934-5	ENSMUST00000073554	ENSMUSG00000062329
mmu_circ_0000908	mmu-miR-1934-5	ENSMUST00000212488	ENSMUSG00000031849
novel_circ_0002114	mmu-miR-1934-5	ENSMUST00000003659	ENSMUSG00000031849
novel_circ_0002114	mmu-miR-1934-5	ENSMUST00000073554	ENSMUSG00000062329
novel_circ_0002114	mmu-miR-1934-5	ENSMUST00000212488	ENSMUSG00000031849
novel_circ_0002114	mmu-miR-1934-5	ENSMUST00000003659	ENSMUSG00000031849
novel_circ_0002114	mmu-miR-1934-5	ENSMUST00000073554	ENSMUSG00000062329
novel_circ_0002114	mmu-miR-1934-5	ENSMUST00000212488	ENSMUSG00000031849
novel_circ_0002115	mmu-miR-1934-5	ENSMUST00000003659	ENSMUSG00000031849
novel_circ_0002115	mmu-miR-1934-5	ENSMUST00000073554	ENSMUSG00000062329
novel_circ_0002115	mmu-miR-1934-5	ENSMUST00000212488	ENSMUSG00000031849
novel_circ_0002144	mmu-miR-1934-5	ENSMUST00000003659	ENSMUSG00000031849
novel_circ_0002144	mmu-miR-1934-5	ENSMUST00000073554	ENSMUSG00000062329
novel_circ_0002144	mmu-miR-1934-5	ENSMUST00000212488	ENSMUSG00000031849
novel_circ_0001758	mmu-miR-1934-5	ENSMUST00000003659	ENSMUSG00000031849
novel_circ_0001758	mmu-miR-1934-5	ENSMUST00000073554	ENSMUSG00000062329
novel_circ_0001758	mmu-miR-1934-5	ENSMUST00000212488	ENSMUSG00000031849
novel_circ_0001804	mmu-miR-1934-5	ENSMUST00000003659	ENSMUSG00000031849
novel_circ_0001804	mmu-miR-1934-5	ENSMUST00000073554	ENSMUSG00000062329
novel_circ_0001804	mmu-miR-1934-5	ENSMUST00000212488	ENSMUSG00000031849
novel_circ_0001843	mmu-miR-1934-5	ENSMUST00000003659	ENSMUSG00000031849
novel_circ_0001843	mmu-miR-1934-5	ENSMUST00000073554	ENSMUSG00000062329
novel_circ_0001843	mmu-miR-1934-5	ENSMUST00000212488	ENSMUSG00000031849
novel_circ_0001843	mmu-miR-1934-5	ENSMUST00000003659	ENSMUSG00000031849
novel_circ_0001843	mmu-miR-1934-5	ENSMUST00000073554	ENSMUSG00000062329
novel_circ_0001843	mmu-miR-1934-5	ENSMUST00000212488	ENSMUSG00000031849
novel_circ_0001844	mmu-miR-1934-5	ENSMUST00000003659	ENSMUSG00000031849
novel_circ_0001844	mmu-miR-1934-5	ENSMUST00000073554	ENSMUSG00000062329
novel_circ_0001844	mmu-miR-1934-5	ENSMUST00000212488	ENSMUSG00000031849
novel_circ_0001844	mmu-miR-1934-5	ENSMUST00000003659	ENSMUSG00000031849
novel_circ_0001844	mmu-miR-1934-5	ENSMUST00000073554	ENSMUSG00000062329
novel_circ_0001844	mmu-miR-1934-5	ENSMUST00000212488	ENSMUSG00000031849
novel_circ_0001853	mmu-miR-1934-5	ENSMUST00000003659	ENSMUSG00000031849
novel_circ_0001853	mmu-miR-1934-5	ENSMUST00000073554	ENSMUSG00000062329

novel_circ_0001853	mmu-miR-1934-5	ENSMUST00000212488	ENSMUSG00000031849
novel_circ_0001888	mmu-miR-1934-5	ENSMUST00000003659	ENSMUSG00000031849
novel_circ_0001888	mmu-miR-1934-5	ENSMUST00000073554	ENSMUSG00000062329
novel_circ_0001888	mmu-miR-1934-5	ENSMUST00000212488	ENSMUSG00000031849
novel_circ_0001907	mmu-miR-1934-5	ENSMUST00000003659	ENSMUSG00000031849
novel_circ_0001907	mmu-miR-1934-5	ENSMUST00000073554	ENSMUSG00000062329
novel_circ_0001907	mmu-miR-1934-5	ENSMUST00000212488	ENSMUSG00000031849
novel_circ_0001986	mmu-miR-1934-5	ENSMUST00000003659	ENSMUSG00000031849
novel_circ_0001986	mmu-miR-1934-5	ENSMUST00000073554	ENSMUSG00000062329
novel_circ_0001986	mmu-miR-1934-5	ENSMUST00000212488	ENSMUSG00000031849
novel_circ_0002051	mmu-miR-1934-5	ENSMUST00000003659	ENSMUSG00000031849
novel_circ_0002051	mmu-miR-1934-5	ENSMUST00000073554	ENSMUSG00000062329
novel_circ_0002051	mmu-miR-1934-5	ENSMUST00000212488	ENSMUSG00000031849
novel_circ_0002417	mmu-miR-1934-5	ENSMUST00000003659	ENSMUSG00000031849
novel_circ_0002417	mmu-miR-1934-5	ENSMUST00000073554	ENSMUSG00000062329
novel_circ_0002417	mmu-miR-1934-5	ENSMUST00000212488	ENSMUSG00000031849
novel_circ_0002436	mmu-miR-1934-5	ENSMUST00000003659	ENSMUSG00000031849
novel_circ_0002436	mmu-miR-1934-5	ENSMUST00000073554	ENSMUSG00000062329
novel_circ_0002436	mmu-miR-1934-5	ENSMUST00000212488	ENSMUSG00000031849
novel_circ_0002457	mmu-miR-1934-5	ENSMUST00000003659	ENSMUSG00000031849
novel_circ_0002457	mmu-miR-1934-5	ENSMUST00000073554	ENSMUSG00000062329
novel_circ_0002457	mmu-miR-1934-5	ENSMUST00000212488	ENSMUSG00000031849
novel_circ_0002457	mmu-miR-1934-5	ENSMUST00000003659	ENSMUSG00000031849
novel_circ_0002457	mmu-miR-1934-5	ENSMUST00000073554	ENSMUSG00000062329
novel_circ_0002457	mmu-miR-1934-5	ENSMUST00000212488	ENSMUSG00000031849
novel_circ_0002497	mmu-miR-1934-5	ENSMUST00000003659	ENSMUSG00000031849
novel_circ_0002497	mmu-miR-1934-5	ENSMUST00000073554	ENSMUSG00000062329
novel_circ_0002497	mmu-miR-1934-5	ENSMUST00000212488	ENSMUSG00000031849
novel_circ_0002530	mmu-miR-1934-5	ENSMUST00000003659	ENSMUSG00000031849
novel_circ_0002530	mmu-miR-1934-5	ENSMUST00000073554	ENSMUSG00000062329
novel_circ_0002530	mmu-miR-1934-5	ENSMUST00000212488	ENSMUSG00000031849
novel_circ_0002577	mmu-miR-1934-5	ENSMUST00000003659	ENSMUSG00000031849
novel_circ_0002577	mmu-miR-1934-5	ENSMUST00000073554	ENSMUSG00000062329
novel_circ_0002577	mmu-miR-1934-5	ENSMUST00000212488	ENSMUSG00000031849
novel_circ_0002581	mmu-miR-1934-5	ENSMUST00000003659	ENSMUSG00000031849
novel_circ_0002581	mmu-miR-1934-5	ENSMUST00000073554	ENSMUSG00000062329
novel_circ_0002581	mmu-miR-1934-5	ENSMUST00000212488	ENSMUSG00000031849
novel_circ_0002618	mmu-miR-1934-5	ENSMUST00000003659	ENSMUSG00000031849
novel_circ_0002618	mmu-miR-1934-5	ENSMUST00000073554	ENSMUSG00000062329
novel_circ_0002618	mmu-miR-1934-5	ENSMUST00000212488	ENSMUSG00000031849
novel_circ_0002618	mmu-miR-1934-5	ENSMUST00000003659	ENSMUSG00000031849
novel_circ_0002618	mmu-miR-1934-5	ENSMUST00000073554	ENSMUSG00000062329
novel_circ_0002618	mmu-miR-1934-5	ENSMUST00000212488	ENSMUSG00000031849
novel_circ_0002284	mmu-miR-1934-5	ENSMUST00000003659	ENSMUSG00000031849
novel_circ_0002284	mmu-miR-1934-5	ENSMUST00000073554	ENSMUSG00000062329
novel_circ_0002284	mmu-miR-1934-5	ENSMUST00000212488	ENSMUSG00000031849
novel_circ_0002376	mmu-miR-1934-5	ENSMUST00000003659	ENSMUSG00000031849
novel_circ_0002376	mmu-miR-1934-5	ENSMUST00000073554	ENSMUSG00000062329
novel_circ_0002376	mmu-miR-1934-5	ENSMUST00000212488	ENSMUSG00000031849
novel_circ_0002378	mmu-miR-1934-5	ENSMUST00000003659	ENSMUSG00000031849
novel_circ_0002378	mmu-miR-1934-5	ENSMUST00000073554	ENSMUSG00000062329
novel_circ_0002378	mmu-miR-1934-5	ENSMUST00000212488	ENSMUSG00000031849
novel_circ_0002826	mmu-miR-1934-5	ENSMUST00000003659	ENSMUSG00000031849
novel_circ_0002826	mmu-miR-1934-5	ENSMUST00000073554	ENSMUSG00000062329
novel_circ_0002826	mmu-miR-1934-5	ENSMUST00000212488	ENSMUSG00000031849
novel_circ_0002831	mmu-miR-1934-5	ENSMUST00000003659	ENSMUSG00000031849
novel_circ_0002831	mmu-miR-1934-5	ENSMUST00000073554	ENSMUSG00000062329
novel_circ_0002831	mmu-miR-1934-5	ENSMUST00000212488	ENSMUSG00000031849

novel_circ_0002870	mmu-miR-1934-5	ENSMUST00000003659	ENSMUSG00000031849
novel_circ_0002870	mmu-miR-1934-5	ENSMUST00000073554	ENSMUSG00000062329
novel_circ_0002870	mmu-miR-1934-5	ENSMUST00000212488	ENSMUSG00000031849
novel_circ_0002905	mmu-miR-1934-5	ENSMUST00000003659	ENSMUSG00000031849
novel_circ_0002905	mmu-miR-1934-5	ENSMUST00000073554	ENSMUSG00000062329
novel_circ_0002905	mmu-miR-1934-5	ENSMUST00000212488	ENSMUSG00000031849
novel_circ_0002909	mmu-miR-1934-5	ENSMUST00000003659	ENSMUSG00000031849
novel_circ_0002909	mmu-miR-1934-5	ENSMUST00000073554	ENSMUSG00000062329
novel_circ_0002909	mmu-miR-1934-5	ENSMUST00000212488	ENSMUSG00000031849
novel_circ_0002917	mmu-miR-1934-5	ENSMUST00000003659	ENSMUSG00000031849
novel_circ_0002917	mmu-miR-1934-5	ENSMUST00000073554	ENSMUSG00000062329
novel_circ_0002917	mmu-miR-1934-5	ENSMUST00000212488	ENSMUSG00000031849
novel_circ_0002917	mmu-miR-1934-5	ENSMUST00000003659	ENSMUSG00000031849
novel_circ_0002917	mmu-miR-1934-5	ENSMUST00000073554	ENSMUSG00000062329
novel_circ_0002917	mmu-miR-1934-5	ENSMUST00000212488	ENSMUSG00000031849
novel_circ_0002936	mmu-miR-1934-5	ENSMUST00000003659	ENSMUSG00000031849
novel_circ_0002936	mmu-miR-1934-5	ENSMUST00000073554	ENSMUSG00000062329
novel_circ_0002936	mmu-miR-1934-5	ENSMUST00000212488	ENSMUSG00000031849
novel_circ_0002631	mmu-miR-1934-5	ENSMUST00000003659	ENSMUSG00000031849
novel_circ_0002631	mmu-miR-1934-5	ENSMUST00000073554	ENSMUSG00000062329
novel_circ_0002631	mmu-miR-1934-5	ENSMUST00000212488	ENSMUSG00000031849
novel_circ_0002640	mmu-miR-1934-5	ENSMUST00000003659	ENSMUSG00000031849
novel_circ_0002640	mmu-miR-1934-5	ENSMUST00000073554	ENSMUSG00000062329
novel_circ_0002640	mmu-miR-1934-5	ENSMUST00000212488	ENSMUSG00000031849
novel_circ_0002682	mmu-miR-1934-5	ENSMUST00000003659	ENSMUSG00000031849
novel_circ_0002682	mmu-miR-1934-5	ENSMUST00000073554	ENSMUSG00000062329
novel_circ_0002682	mmu-miR-1934-5	ENSMUST00000212488	ENSMUSG00000031849
novel_circ_0002682	mmu-miR-1934-5	ENSMUST00000003659	ENSMUSG00000031849
novel_circ_0002682	mmu-miR-1934-5	ENSMUST00000073554	ENSMUSG00000062329
novel_circ_0002682	mmu-miR-1934-5	ENSMUST00000212488	ENSMUSG00000031849
novel_circ_0003185	mmu-miR-1934-5	ENSMUST00000003659	ENSMUSG00000031849
novel_circ_0003185	mmu-miR-1934-5	ENSMUST00000073554	ENSMUSG00000062329
novel_circ_0003185	mmu-miR-1934-5	ENSMUST00000212488	ENSMUSG00000031849
novel_circ_0003187	mmu-miR-1934-5	ENSMUST00000003659	ENSMUSG00000031849
novel_circ_0003187	mmu-miR-1934-5	ENSMUST00000073554	ENSMUSG00000062329
novel_circ_0003187	mmu-miR-1934-5	ENSMUST00000212488	ENSMUSG00000031849
novel_circ_0003249	mmu-miR-1934-5	ENSMUST00000003659	ENSMUSG00000031849
novel_circ_0003249	mmu-miR-1934-5	ENSMUST00000073554	ENSMUSG00000062329
novel_circ_0003249	mmu-miR-1934-5	ENSMUST00000212488	ENSMUSG00000031849
novel_circ_0003249	mmu-miR-1934-5	ENSMUST00000003659	ENSMUSG00000031849
novel_circ_0003249	mmu-miR-1934-5	ENSMUST00000073554	ENSMUSG00000062329
novel_circ_0003249	mmu-miR-1934-5	ENSMUST00000212488	ENSMUSG00000031849
novel_circ_0003259	mmu-miR-1934-5	ENSMUST00000003659	ENSMUSG00000031849
novel_circ_0003259	mmu-miR-1934-5	ENSMUST00000073554	ENSMUSG00000062329
novel_circ_0003259	mmu-miR-1934-5	ENSMUST00000212488	ENSMUSG00000031849
novel_circ_0003267	mmu-miR-1934-5	ENSMUST00000003659	ENSMUSG00000031849
novel_circ_0003267	mmu-miR-1934-5	ENSMUST00000073554	ENSMUSG00000062329
novel_circ_0003267	mmu-miR-1934-5	ENSMUST00000212488	ENSMUSG00000031849
novel_circ_0003268	mmu-miR-1934-5	ENSMUST00000003659	ENSMUSG00000031849
novel_circ_0003268	mmu-miR-1934-5	ENSMUST00000073554	ENSMUSG00000062329
novel_circ_0003268	mmu-miR-1934-5	ENSMUST00000212488	ENSMUSG00000031849
novel_circ_0003415	mmu-miR-1934-5	ENSMUST00000003659	ENSMUSG00000031849
novel_circ_0003415	mmu-miR-1934-5	ENSMUST00000073554	ENSMUSG00000062329
novel_circ_0003415	mmu-miR-1934-5	ENSMUST00000212488	ENSMUSG00000031849
novel_circ_0003041	mmu-miR-1934-5	ENSMUST00000003659	ENSMUSG00000031849
novel_circ_0003041	mmu-miR-1934-5	ENSMUST00000073554	ENSMUSG00000062329
novel_circ_0003041	mmu-miR-1934-5	ENSMUST00000212488	ENSMUSG00000031849
novel_circ_0003047	mmu-miR-1934-5	ENSMUST00000003659	ENSMUSG00000031849

novel_circ_0003047	mmu-miR-1934-5	ENSMUST00000073554	ENSMUSG00000062329
novel_circ_0003047	mmu-miR-1934-5	ENSMUST00000212488	ENSMUSG00000031849
novel_circ_0003047	mmu-miR-1934-5	ENSMUST00000003659	ENSMUSG00000031849
novel_circ_0003047	mmu-miR-1934-5	ENSMUST00000073554	ENSMUSG00000062329
novel_circ_0003047	mmu-miR-1934-5	ENSMUST00000212488	ENSMUSG00000031849
novel_circ_0003076	mmu-miR-1934-5	ENSMUST00000003659	ENSMUSG00000031849
novel_circ_0003076	mmu-miR-1934-5	ENSMUST00000073554	ENSMUSG00000062329
novel_circ_0003076	mmu-miR-1934-5	ENSMUST00000212488	ENSMUSG00000031849
novel_circ_0003077	mmu-miR-1934-5	ENSMUST00000003659	ENSMUSG00000031849
novel_circ_0003077	mmu-miR-1934-5	ENSMUST00000073554	ENSMUSG00000062329
novel_circ_0003077	mmu-miR-1934-5	ENSMUST00000212488	ENSMUSG00000031849
novel_circ_0003122	mmu-miR-1934-5	ENSMUST00000003659	ENSMUSG00000031849
novel_circ_0003122	mmu-miR-1934-5	ENSMUST00000073554	ENSMUSG00000062329
novel_circ_0003122	mmu-miR-1934-5	ENSMUST00000212488	ENSMUSG00000031849
novel_circ_0003433	mmu-miR-1934-5	ENSMUST00000003659	ENSMUSG00000031849
novel_circ_0003433	mmu-miR-1934-5	ENSMUST00000073554	ENSMUSG00000062329
novel_circ_0003433	mmu-miR-1934-5	ENSMUST00000212488	ENSMUSG00000031849
novel_circ_0003460	mmu-miR-1934-5	ENSMUST00000003659	ENSMUSG00000031849
novel_circ_0003460	mmu-miR-1934-5	ENSMUST00000073554	ENSMUSG00000062329
novel_circ_0003460	mmu-miR-1934-5	ENSMUST00000212488	ENSMUSG00000031849
novel_circ_0003478	mmu-miR-1934-5	ENSMUST00000003659	ENSMUSG00000031849
novel_circ_0003478	mmu-miR-1934-5	ENSMUST00000073554	ENSMUSG00000062329
novel_circ_0003478	mmu-miR-1934-5	ENSMUST00000212488	ENSMUSG00000031849
novel_circ_0000186	mmu-miR-1934-5	ENSMUST00000003659	ENSMUSG00000031849
novel_circ_0000186	mmu-miR-1934-5	ENSMUST00000073554	ENSMUSG00000062329
novel_circ_0000186	mmu-miR-1934-5	ENSMUST00000212488	ENSMUSG00000031849
novel_circ_0000212	mmu-miR-1934-5	ENSMUST00000003659	ENSMUSG00000031849
novel_circ_0000212	mmu-miR-1934-5	ENSMUST00000073554	ENSMUSG00000062329
novel_circ_0000212	mmu-miR-1934-5	ENSMUST00000212488	ENSMUSG00000031849
novel_circ_0000244	mmu-miR-1934-5	ENSMUST00000003659	ENSMUSG00000031849
novel_circ_0000244	mmu-miR-1934-5	ENSMUST00000073554	ENSMUSG00000062329
novel_circ_0000244	mmu-miR-1934-5	ENSMUST00000212488	ENSMUSG00000031849
novel_circ_0000268	mmu-miR-1934-5	ENSMUST00000003659	ENSMUSG00000031849
novel_circ_0000268	mmu-miR-1934-5	ENSMUST00000073554	ENSMUSG00000062329
novel_circ_0000268	mmu-miR-1934-5	ENSMUST00000212488	ENSMUSG00000031849
novel_circ_0000308	mmu-miR-1934-5	ENSMUST00000003659	ENSMUSG00000031849
novel_circ_0000308	mmu-miR-1934-5	ENSMUST00000073554	ENSMUSG00000062329
novel_circ_0000308	mmu-miR-1934-5	ENSMUST00000212488	ENSMUSG00000031849
novel_circ_0000124	mmu-miR-1934-5	ENSMUST00000003659	ENSMUSG00000031849
novel_circ_0000124	mmu-miR-1934-5	ENSMUST00000073554	ENSMUSG00000062329
novel_circ_0000124	mmu-miR-1934-5	ENSMUST00000212488	ENSMUSG00000031849
novel_circ_0000125	mmu-miR-1934-5	ENSMUST00000003659	ENSMUSG00000031849
novel_circ_0000125	mmu-miR-1934-5	ENSMUST00000073554	ENSMUSG00000062329
novel_circ_0000125	mmu-miR-1934-5	ENSMUST00000212488	ENSMUSG00000031849
novel_circ_0000625	mmu-miR-1934-5	ENSMUST00000003659	ENSMUSG00000031849
novel_circ_0000625	mmu-miR-1934-5	ENSMUST00000073554	ENSMUSG00000062329
novel_circ_0000625	mmu-miR-1934-5	ENSMUST00000212488	ENSMUSG00000031849
novel_circ_0000656	mmu-miR-1934-5	ENSMUST00000003659	ENSMUSG00000031849
novel_circ_0000656	mmu-miR-1934-5	ENSMUST00000073554	ENSMUSG00000062329
novel_circ_0000656	mmu-miR-1934-5	ENSMUST00000212488	ENSMUSG00000031849
novel_circ_0000691	mmu-miR-1934-5	ENSMUST00000003659	ENSMUSG00000031849
novel_circ_0000691	mmu-miR-1934-5	ENSMUST00000073554	ENSMUSG00000062329
novel_circ_0000691	mmu-miR-1934-5	ENSMUST00000212488	ENSMUSG00000031849
novel_circ_0000482	mmu-miR-1934-5	ENSMUST00000003659	ENSMUSG00000031849
novel_circ_0000482	mmu-miR-1934-5	ENSMUST00000073554	ENSMUSG00000062329
novel_circ_0000482	mmu-miR-1934-5	ENSMUST00000212488	ENSMUSG00000031849
novel_circ_0000589	mmu-miR-1934-5	ENSMUST00000003659	ENSMUSG00000031849
novel_circ_0000589	mmu-miR-1934-5	ENSMUST00000073554	ENSMUSG00000062329

novel_circ_0000589	mmu-miR-1934-5	ENSMUST00000212488	ENSMUSG00000031849
novel_circ_0000896	mmu-miR-1934-5	ENSMUST00000003659	ENSMUSG00000031849
novel_circ_0000896	mmu-miR-1934-5	ENSMUST00000073554	ENSMUSG00000062329
novel_circ_0000896	mmu-miR-1934-5	ENSMUST00000212488	ENSMUSG00000031849
novel_circ_0000896	mmu-miR-1934-5	ENSMUST00000003659	ENSMUSG00000031849
novel_circ_0000896	mmu-miR-1934-5	ENSMUST00000073554	ENSMUSG00000062329
novel_circ_0000896	mmu-miR-1934-5	ENSMUST00000212488	ENSMUSG00000031849
novel_circ_0001172	mmu-miR-1934-5	ENSMUST00000003659	ENSMUSG00000031849
novel_circ_0001172	mmu-miR-1934-5	ENSMUST00000073554	ENSMUSG00000062329
novel_circ_0001172	mmu-miR-1934-5	ENSMUST00000212488	ENSMUSG00000031849
novel_circ_0001248	mmu-miR-1958	ENSMUST00000225921	ENSMUSG00000017491
novel_circ_0001066	mmu-miR-1958	ENSMUST00000225921	ENSMUSG00000017491
novel_circ_0001066	mmu-miR-1958	ENSMUST00000225921	ENSMUSG00000017491
novel_circ_0001091	mmu-miR-1958	ENSMUST00000225921	ENSMUSG00000017491
novel_circ_0001101	mmu-miR-1958	ENSMUST00000225921	ENSMUSG00000017491
novel_circ_0001144	mmu-miR-1958	ENSMUST00000225921	ENSMUSG00000017491
novel_circ_0001478	mmu-miR-1958	ENSMUST00000225921	ENSMUSG00000017491
novel_circ_0001478	mmu-miR-1958	ENSMUST00000225921	ENSMUSG00000017491
novel_circ_0001485	mmu-miR-1958	ENSMUST00000225921	ENSMUSG00000017491
novel_circ_0001349	mmu-miR-1958	ENSMUST00000225921	ENSMUSG00000017491
novel_circ_0001433	mmu-miR-1958	ENSMUST00000225921	ENSMUSG00000017491
mmu_circ_0000132	mmu-miR-1958	ENSMUST00000225921	ENSMUSG00000017491
mmu_circ_0000133	mmu-miR-1958	ENSMUST00000225921	ENSMUSG00000017491
mmu_circ_0000133	mmu-miR-1958	ENSMUST00000225921	ENSMUSG00000017491
mmu_circ_0000028	mmu-miR-1958	ENSMUST00000225921	ENSMUSG00000017491
mmu_circ_0000042	mmu-miR-1958	ENSMUST00000225921	ENSMUSG00000017491
mmu_circ_0000049	mmu-miR-1958	ENSMUST00000225921	ENSMUSG00000017491
mmu_circ_0000063	mmu-miR-1958	ENSMUST00000225921	ENSMUSG00000017491
mmu_circ_0001123	mmu-miR-1958	ENSMUST00000225921	ENSMUSG00000017491
mmu_circ_0001135	mmu-miR-1958	ENSMUST00000225921	ENSMUSG00000017491
mmu_circ_0001135	mmu-miR-1958	ENSMUST00000225921	ENSMUSG00000017491
mmu_circ_0001031	mmu-miR-1958	ENSMUST00000225921	ENSMUSG00000017491
mmu_circ_0001376	mmu-miR-1958	ENSMUST00000225921	ENSMUSG00000017491
mmu_circ_0001398	mmu-miR-1958	ENSMUST00000225921	ENSMUSG00000017491
mmu_circ_0001332	mmu-miR-1958	ENSMUST00000225921	ENSMUSG00000017491
mmu_circ_0001351	mmu-miR-1958	ENSMUST00000225921	ENSMUSG00000017491
mmu_circ_0001261	mmu-miR-1958	ENSMUST00000225921	ENSMUSG00000017491
mmu_circ_0001222	mmu-miR-1958	ENSMUST00000225921	ENSMUSG00000017491
mmu_circ_0001634	mmu-miR-1958	ENSMUST00000225921	ENSMUSG00000017491
mmu_circ_0001454	mmu-miR-1958	ENSMUST00000225921	ENSMUSG00000017491
mmu_circ_0001470	mmu-miR-1958	ENSMUST00000225921	ENSMUSG00000017491
mmu_circ_0001499	mmu-miR-1958	ENSMUST00000225921	ENSMUSG00000017491
mmu_circ_0001500	mmu-miR-1958	ENSMUST00000225921	ENSMUSG00000017491
mmu_circ_0000230	mmu-miR-1958	ENSMUST00000225921	ENSMUSG00000017491
mmu_circ_0000301	mmu-miR-1958	ENSMUST00000225921	ENSMUSG00000017491
mmu_circ_0000234	mmu-miR-1958	ENSMUST00000225921	ENSMUSG00000017491
mmu_circ_0000163	mmu-miR-1958	ENSMUST00000225921	ENSMUSG00000017491
mmu_circ_0000499	mmu-miR-1958	ENSMUST00000225921	ENSMUSG00000017491
mmu_circ_0000500	mmu-miR-1958	ENSMUST00000225921	ENSMUSG00000017491
mmu_circ_0000457	mmu-miR-1958	ENSMUST00000225921	ENSMUSG00000017491
mmu_circ_0000371	mmu-miR-1958	ENSMUST00000225921	ENSMUSG00000017491
mmu_circ_0000399	mmu-miR-1958	ENSMUST00000225921	ENSMUSG00000017491
mmu_circ_0000628	mmu-miR-1958	ENSMUST00000225921	ENSMUSG00000017491
mmu_circ_0000530	mmu-miR-1958	ENSMUST00000225921	ENSMUSG00000017491
mmu_circ_0000738	mmu-miR-1958	ENSMUST00000225921	ENSMUSG00000017491
mmu_circ_0000812	mmu-miR-1958	ENSMUST00000225921	ENSMUSG00000017491
mmu_circ_0000828	mmu-miR-1958	ENSMUST00000225921	ENSMUSG00000017491
mmu_circ_0000829	mmu-miR-1958	ENSMUST00000225921	ENSMUSG00000017491

mmu_circ_0000831	mmu-miR-1958	ENSMUST00000225921	ENSMUSG00000017491
mmu_circ_0000713	mmu-miR-1958	ENSMUST00000225921	ENSMUSG00000017491
mmu_circ_0000714	mmu-miR-1958	ENSMUST00000225921	ENSMUSG00000017491
mmu_circ_0000950	mmu-miR-1958	ENSMUST00000225921	ENSMUSG00000017491
mmu_circ_0000917	mmu-miR-1958	ENSMUST00000225921	ENSMUSG00000017491
mmu_circ_0000918	mmu-miR-1958	ENSMUST00000225921	ENSMUSG00000017491
mmu_circ_0000919	mmu-miR-1958	ENSMUST00000225921	ENSMUSG00000017491
mmu_circ_0000969	mmu-miR-1958	ENSMUST00000225921	ENSMUSG00000017491
mmu_circ_0000878	mmu-miR-1958	ENSMUST00000225921	ENSMUSG00000017491
mmu_circ_0000884	mmu-miR-1958	ENSMUST00000225921	ENSMUSG00000017491
mmu_circ_0000886	mmu-miR-1958	ENSMUST00000225921	ENSMUSG00000017491
mmu_circ_0000887	mmu-miR-1958	ENSMUST00000225921	ENSMUSG00000017491
mmu_circ_0000897	mmu-miR-1958	ENSMUST00000225921	ENSMUSG00000017491
mmu_circ_0000905	mmu-miR-1958	ENSMUST00000225921	ENSMUSG00000017491
mmu_circ_0000906	mmu-miR-1958	ENSMUST00000225921	ENSMUSG00000017491
mmu_circ_0000907	mmu-miR-1958	ENSMUST00000225921	ENSMUSG00000017491
mmu_circ_0000908	mmu-miR-1958	ENSMUST00000225921	ENSMUSG00000017491
novel_circ_0001561	mmu-miR-1958	ENSMUST00000225921	ENSMUSG00000017491
novel_circ_0001702	mmu-miR-1958	ENSMUST00000225921	ENSMUSG00000017491
novel_circ_0001726	mmu-miR-1958	ENSMUST00000225921	ENSMUSG00000017491
novel_circ_0002100	mmu-miR-1958	ENSMUST00000225921	ENSMUSG00000017491
novel_circ_0001751	mmu-miR-1958	ENSMUST00000225921	ENSMUSG00000017491
novel_circ_0001813	mmu-miR-1958	ENSMUST00000225921	ENSMUSG00000017491
novel_circ_0001813	mmu-miR-1958	ENSMUST00000225921	ENSMUSG00000017491
novel_circ_0001850	mmu-miR-1958	ENSMUST00000225921	ENSMUSG00000017491
novel_circ_0001897	mmu-miR-1958	ENSMUST00000225921	ENSMUSG00000017491
novel_circ_0001899	mmu-miR-1958	ENSMUST00000225921	ENSMUSG00000017491
novel_circ_0001950	mmu-miR-1958	ENSMUST00000225921	ENSMUSG00000017491
novel_circ_0002450	mmu-miR-1958	ENSMUST00000225921	ENSMUSG00000017491
novel_circ_0002451	mmu-miR-1958	ENSMUST00000225921	ENSMUSG00000017491
novel_circ_0002339	mmu-miR-1958	ENSMUST00000225921	ENSMUSG00000017491
novel_circ_0002399	mmu-miR-1958	ENSMUST00000225921	ENSMUSG00000017491
novel_circ_0002831	mmu-miR-1958	ENSMUST00000225921	ENSMUSG00000017491
novel_circ_0002902	mmu-miR-1958	ENSMUST00000225921	ENSMUSG00000017491
novel_circ_0002902	mmu-miR-1958	ENSMUST00000225921	ENSMUSG00000017491
novel_circ_0002936	mmu-miR-1958	ENSMUST00000225921	ENSMUSG00000017491
novel_circ_0002990	mmu-miR-1958	ENSMUST00000225921	ENSMUSG00000017491
novel_circ_0002732	mmu-miR-1958	ENSMUST00000225921	ENSMUSG00000017491
novel_circ_0002782	mmu-miR-1958	ENSMUST00000225921	ENSMUSG00000017491
novel_circ_0003224	mmu-miR-1958	ENSMUST00000225921	ENSMUSG00000017491
novel_circ_0003226	mmu-miR-1958	ENSMUST00000225921	ENSMUSG00000017491
novel_circ_0003256	mmu-miR-1958	ENSMUST00000225921	ENSMUSG00000017491
novel_circ_0003370	mmu-miR-1958	ENSMUST00000225921	ENSMUSG00000017491
novel_circ_0003031	mmu-miR-1958	ENSMUST00000225921	ENSMUSG00000017491
novel_circ_0003144	mmu-miR-1958	ENSMUST00000225921	ENSMUSG00000017491
novel_circ_0003159	mmu-miR-1958	ENSMUST00000225921	ENSMUSG00000017491
novel_circ_0003458	mmu-miR-1958	ENSMUST00000225921	ENSMUSG00000017491
novel_circ_0003468	mmu-miR-1958	ENSMUST00000225921	ENSMUSG00000017491
novel_circ_0003476	mmu-miR-1958	ENSMUST00000225921	ENSMUSG00000017491
novel_circ_0000436	mmu-miR-1958	ENSMUST00000225921	ENSMUSG00000017491
novel_circ_0000441	mmu-miR-1958	ENSMUST00000225921	ENSMUSG00000017491
novel_circ_0000021	mmu-miR-1958	ENSMUST00000225921	ENSMUSG00000017491
novel_circ_0000128	mmu-miR-1958	ENSMUST00000225921	ENSMUSG00000017491
novel_circ_0000143	mmu-miR-1958	ENSMUST00000225921	ENSMUSG00000017491
novel_circ_0000631	mmu-miR-1958	ENSMUST00000225921	ENSMUSG00000017491
novel_circ_0000633	mmu-miR-1958	ENSMUST00000225921	ENSMUSG00000017491
novel_circ_0000681	mmu-miR-1958	ENSMUST00000225921	ENSMUSG00000017491
novel_circ_0000682	mmu-miR-1958	ENSMUST00000225921	ENSMUSG00000017491

novel_circ_0000684	mmu-miR-1958	ENSMUST00000225921	ENSMUSG00000017491
novel_circ_0000703	mmu-miR-1958	ENSMUST00000225921	ENSMUSG00000017491
novel_circ_0000985	mmu-miR-1958	ENSMUST00000225921	ENSMUSG00000017491
novel_circ_0000814	mmu-miR-1958	ENSMUST00000225921	ENSMUSG00000017491
novel_circ_0000826	mmu-miR-1958	ENSMUST00000225921	ENSMUSG00000017491
novel_circ_0000837	mmu-miR-1958	ENSMUST00000225921	ENSMUSG00000017491
novel_circ_0000862	mmu-miR-1958	ENSMUST00000225921	ENSMUSG00000017491
novel_circ_0000891	mmu-miR-1958	ENSMUST00000225921	ENSMUSG00000017491
novel_circ_0000891	mmu-miR-1958	ENSMUST00000225921	ENSMUSG00000017491
novel_circ_0001196	mmu-miR-1958	ENSMUST00000225921	ENSMUSG00000017491
novel_circ_0001196	mmu-miR-1958	ENSMUST00000225921	ENSMUSG00000017491
novel_circ_0001270	mmu-miR-1970	ENSMUST00000225594	ENSMUSG00000017491
novel_circ_0001112	mmu-miR-1970	ENSMUST00000225594	ENSMUSG00000017491
novel_circ_0001315	mmu-miR-1970	ENSMUST00000225594	ENSMUSG00000017491
novel_circ_0001355	mmu-miR-1970	ENSMUST00000225594	ENSMUSG00000017491
mmu_circ_0000051	mmu-miR-1970	ENSMUST00000225594	ENSMUSG00000017491
mmu_circ_0000051	mmu-miR-1970	ENSMUST00000225594	ENSMUSG00000017491
mmu_circ_0001123	mmu-miR-1970	ENSMUST00000225594	ENSMUSG00000017491
mmu_circ_0001124	mmu-miR-1970	ENSMUST00000225594	ENSMUSG00000017491
mmu_circ_0001125	mmu-miR-1970	ENSMUST00000225594	ENSMUSG00000017491
mmu_circ_0001059	mmu-miR-1970	ENSMUST00000225594	ENSMUSG00000017491
mmu_circ_0001021	mmu-miR-1970	ENSMUST00000225594	ENSMUSG00000017491
mmu_circ_0001333	mmu-miR-1970	ENSMUST00000225594	ENSMUSG00000017491
mmu_circ_0001213	mmu-miR-1970	ENSMUST00000225594	ENSMUSG00000017491
mmu_circ_0001537	mmu-miR-1970	ENSMUST00000225594	ENSMUSG00000017491
mmu_circ_0001454	mmu-miR-1970	ENSMUST00000225594	ENSMUSG00000017491
mmu_circ_0001436	mmu-miR-1970	ENSMUST00000225594	ENSMUSG00000017491
mmu_circ_0001436	mmu-miR-1970	ENSMUST00000225594	ENSMUSG00000017491
mmu_circ_0001499	mmu-miR-1970	ENSMUST00000225594	ENSMUSG00000017491
mmu_circ_0001500	mmu-miR-1970	ENSMUST00000225594	ENSMUSG00000017491
mmu_circ_0000465	mmu-miR-1970	ENSMUST00000225594	ENSMUSG00000017491
mmu_circ_0000491	mmu-miR-1970	ENSMUST00000225594	ENSMUSG00000017491
mmu_circ_0000404	mmu-miR-1970	ENSMUST00000225594	ENSMUSG00000017491
mmu_circ_0000404	mmu-miR-1970	ENSMUST00000225594	ENSMUSG00000017491
mmu_circ_0000530	mmu-miR-1970	ENSMUST00000225594	ENSMUSG00000017491
mmu_circ_0000738	mmu-miR-1970	ENSMUST00000225594	ENSMUSG00000017491
mmu_circ_0000804	mmu-miR-1970	ENSMUST00000225594	ENSMUSG00000017491
mmu_circ_0000829	mmu-miR-1970	ENSMUST00000225594	ENSMUSG00000017491
mmu_circ_0000831	mmu-miR-1970	ENSMUST00000225594	ENSMUSG00000017491
mmu_circ_0000688	mmu-miR-1970	ENSMUST00000225594	ENSMUSG00000017491
mmu_circ_0000711	mmu-miR-1970	ENSMUST00000225594	ENSMUSG00000017491
mmu_circ_0000714	mmu-miR-1970	ENSMUST00000225594	ENSMUSG00000017491
mmu_circ_0000969	mmu-miR-1970	ENSMUST00000225594	ENSMUSG00000017491
mmu_circ_0000844	mmu-miR-1970	ENSMUST00000225594	ENSMUSG00000017491
mmu_circ_0000844	mmu-miR-1970	ENSMUST00000225594	ENSMUSG00000017491
mmu_circ_0000884	mmu-miR-1970	ENSMUST00000225594	ENSMUSG00000017491
mmu_circ_0000886	mmu-miR-1970	ENSMUST00000225594	ENSMUSG00000017491
mmu_circ_0000906	mmu-miR-1970	ENSMUST00000225594	ENSMUSG00000017491
mmu_circ_0000907	mmu-miR-1970	ENSMUST00000225594	ENSMUSG00000017491
mmu_circ_0000908	mmu-miR-1970	ENSMUST00000225594	ENSMUSG00000017491
mmu_circ_0000912	mmu-miR-1970	ENSMUST00000225594	ENSMUSG00000017491
novel_circ_0001597	mmu-miR-1970	ENSMUST00000225594	ENSMUSG00000017491
novel_circ_0001597	mmu-miR-1970	ENSMUST00000225594	ENSMUSG00000017491
novel_circ_0002143	mmu-miR-1970	ENSMUST00000225594	ENSMUSG00000017491
novel_circ_0001769	mmu-miR-1970	ENSMUST00000225594	ENSMUSG00000017491
novel_circ_0001770	mmu-miR-1970	ENSMUST00000225594	ENSMUSG00000017491
novel_circ_0001782	mmu-miR-1970	ENSMUST00000225594	ENSMUSG00000017491
novel_circ_0001883	mmu-miR-1970	ENSMUST00000225594	ENSMUSG00000017491

novel_circ_0002600	mmu-miR-1970	ENSMUST00000225594	ENSMUSG00000017491
novel_circ_0002617	mmu-miR-1970	ENSMUST00000225594	ENSMUSG00000017491
novel_circ_0002274	mmu-miR-1970	ENSMUST00000225594	ENSMUSG00000017491
novel_circ_0002356	mmu-miR-1970	ENSMUST00000225594	ENSMUSG00000017491
novel_circ_0002375	mmu-miR-1970	ENSMUST00000225594	ENSMUSG00000017491
novel_circ_0002831	mmu-miR-1970	ENSMUST00000225594	ENSMUSG00000017491
novel_circ_0002913	mmu-miR-1970	ENSMUST00000225594	ENSMUSG00000017491
novel_circ_0002913	mmu-miR-1970	ENSMUST00000225594	ENSMUSG00000017491
novel_circ_0002656	mmu-miR-1970	ENSMUST00000225594	ENSMUSG00000017491
novel_circ_0002658	mmu-miR-1970	ENSMUST00000225594	ENSMUSG00000017491
novel_circ_0002680	mmu-miR-1970	ENSMUST00000225594	ENSMUSG00000017491
novel_circ_0002681	mmu-miR-1970	ENSMUST00000225594	ENSMUSG00000017491
novel_circ_0002720	mmu-miR-1970	ENSMUST00000225594	ENSMUSG00000017491
novel_circ_0002720	mmu-miR-1970	ENSMUST00000225594	ENSMUSG00000017491
novel_circ_0002724	mmu-miR-1970	ENSMUST00000225594	ENSMUSG00000017491
novel_circ_0002724	mmu-miR-1970	ENSMUST00000225594	ENSMUSG00000017491
novel_circ_0003242	mmu-miR-1970	ENSMUST00000225594	ENSMUSG00000017491
novel_circ_0003267	mmu-miR-1970	ENSMUST00000225594	ENSMUSG00000017491
novel_circ_0003268	mmu-miR-1970	ENSMUST00000225594	ENSMUSG00000017491
novel_circ_0003016	mmu-miR-1970	ENSMUST00000225594	ENSMUSG00000017491
novel_circ_0003083	mmu-miR-1970	ENSMUST00000225594	ENSMUSG00000017491
novel_circ_0003142	mmu-miR-1970	ENSMUST00000225594	ENSMUSG00000017491
novel_circ_0003156	mmu-miR-1970	ENSMUST00000225594	ENSMUSG00000017491
novel_circ_0003458	mmu-miR-1970	ENSMUST00000225594	ENSMUSG00000017491
novel_circ_0000200	mmu-miR-1970	ENSMUST00000225594	ENSMUSG00000017491
novel_circ_0000201	mmu-miR-1970	ENSMUST00000225594	ENSMUSG00000017491
novel_circ_0000208	mmu-miR-1970	ENSMUST00000225594	ENSMUSG00000017491
novel_circ_0000244	mmu-miR-1970	ENSMUST00000225594	ENSMUSG00000017491
novel_circ_0000268	mmu-miR-1970	ENSMUST00000225594	ENSMUSG00000017491
novel_circ_0000389	mmu-miR-1970	ENSMUST00000225594	ENSMUSG00000017491
novel_circ_0000443	mmu-miR-1970	ENSMUST00000225594	ENSMUSG00000017491
novel_circ_0000015	mmu-miR-1970	ENSMUST00000225594	ENSMUSG00000017491
novel_circ_0000050	mmu-miR-1970	ENSMUST00000225594	ENSMUSG00000017491
novel_circ_0000682	mmu-miR-1970	ENSMUST00000225594	ENSMUSG00000017491
novel_circ_0000753	mmu-miR-1970	ENSMUST00000225594	ENSMUSG00000017491
novel_circ_0000514	mmu-miR-1970	ENSMUST00000225594	ENSMUSG00000017491
novel_circ_0000938	mmu-miR-1970	ENSMUST00000225594	ENSMUSG00000017491
novel_circ_0001019	mmu-miR-1970	ENSMUST00000225594	ENSMUSG00000017491
novel_circ_0001019	mmu-miR-1970	ENSMUST00000225594	ENSMUSG00000017491
novel_circ_0000904	mmu-miR-1970	ENSMUST00000225594	ENSMUSG00000017491
novel_circ_0001233	mmu-miR-3061-5	ENSMUST00000000128	ENSMUSG00000000126
novel_circ_0001233	mmu-miR-3061-5	ENSMUST00000108783	ENSMUSG00000000126
novel_circ_0001106	mmu-miR-3061-5	ENSMUST00000000128	ENSMUSG00000000126
novel_circ_0001106	mmu-miR-3061-5	ENSMUST00000108783	ENSMUSG00000000126
novel_circ_0001438	mmu-miR-3061-5	ENSMUST00000000128	ENSMUSG00000000126
novel_circ_0001438	mmu-miR-3061-5	ENSMUST00000108783	ENSMUSG00000000126
novel_circ_0001349	mmu-miR-3061-5	ENSMUST00000000128	ENSMUSG00000000126
novel_circ_0001349	mmu-miR-3061-5	ENSMUST00000108783	ENSMUSG00000000126
mmu_circ_0000063	mmu-miR-3061-5	ENSMUST00000000128	ENSMUSG00000000126
mmu_circ_0000063	mmu-miR-3061-5	ENSMUST00000108783	ENSMUSG00000000126
mmu_circ_0001103	mmu-miR-3061-5	ENSMUST00000000128	ENSMUSG00000000126
mmu_circ_0001103	mmu-miR-3061-5	ENSMUST00000108783	ENSMUSG00000000126
mmu_circ_0001017	mmu-miR-3061-5	ENSMUST00000000128	ENSMUSG00000000126
mmu_circ_0001017	mmu-miR-3061-5	ENSMUST00000108783	ENSMUSG00000000126
mmu_circ_0001376	mmu-miR-3061-5	ENSMUST00000000128	ENSMUSG00000000126
mmu_circ_0001376	mmu-miR-3061-5	ENSMUST00000108783	ENSMUSG00000000126
mmu_circ_0001620	mmu-miR-3061-5	ENSMUST00000000128	ENSMUSG00000000126
mmu_circ_0001620	mmu-miR-3061-5	ENSMUST00000108783	ENSMUSG00000000126

mmu_circ_0001505	mmu-miR-3061-5	ENSMUST00000000128	ENSMUSG00000000126
mmu_circ_0001505	mmu-miR-3061-5	ENSMUST00000108783	ENSMUSG00000000126
mmu_circ_0001454	mmu-miR-3061-5	ENSMUST00000000128	ENSMUSG00000000126
mmu_circ_0001454	mmu-miR-3061-5	ENSMUST00000108783	ENSMUSG00000000126
mmu_circ_0001499	mmu-miR-3061-5	ENSMUST00000000128	ENSMUSG00000000126
mmu_circ_0001499	mmu-miR-3061-5	ENSMUST00000108783	ENSMUSG00000000126
mmu_circ_0001500	mmu-miR-3061-5	ENSMUST00000000128	ENSMUSG00000000126
mmu_circ_0001500	mmu-miR-3061-5	ENSMUST00000108783	ENSMUSG00000000126
mmu_circ_0001802	mmu-miR-3061-5	ENSMUST00000000128	ENSMUSG00000000126
mmu_circ_0001802	mmu-miR-3061-5	ENSMUST00000108783	ENSMUSG00000000126
mmu_circ_0001827	mmu-miR-3061-5	ENSMUST00000000128	ENSMUSG00000000126
mmu_circ_0001827	mmu-miR-3061-5	ENSMUST00000108783	ENSMUSG00000000126
mmu_circ_0000499	mmu-miR-3061-5	ENSMUST00000000128	ENSMUSG00000000126
mmu_circ_0000499	mmu-miR-3061-5	ENSMUST00000108783	ENSMUSG00000000126
mmu_circ_0000500	mmu-miR-3061-5	ENSMUST00000000128	ENSMUSG00000000126
mmu_circ_0000500	mmu-miR-3061-5	ENSMUST00000108783	ENSMUSG00000000126
mmu_circ_0000375	mmu-miR-3061-5	ENSMUST00000000128	ENSMUSG00000000126
mmu_circ_0000375	mmu-miR-3061-5	ENSMUST00000108783	ENSMUSG00000000126
mmu_circ_0000375	mmu-miR-3061-5	ENSMUST00000000128	ENSMUSG00000000126
mmu_circ_0000375	mmu-miR-3061-5	ENSMUST00000108783	ENSMUSG00000000126
mmu_circ_0000399	mmu-miR-3061-5	ENSMUST00000000128	ENSMUSG00000000126
mmu_circ_0000399	mmu-miR-3061-5	ENSMUST00000108783	ENSMUSG00000000126
mmu_circ_0000577	mmu-miR-3061-5	ENSMUST00000000128	ENSMUSG00000000126
mmu_circ_0000577	mmu-miR-3061-5	ENSMUST00000108783	ENSMUSG00000000126
mmu_circ_0000604	mmu-miR-3061-5	ENSMUST00000000128	ENSMUSG00000000126
mmu_circ_0000604	mmu-miR-3061-5	ENSMUST00000108783	ENSMUSG00000000126
mmu_circ_0000552	mmu-miR-3061-5	ENSMUST00000000128	ENSMUSG00000000126
mmu_circ_0000552	mmu-miR-3061-5	ENSMUST00000108783	ENSMUSG00000000126
mmu_circ_0000552	mmu-miR-3061-5	ENSMUST00000000128	ENSMUSG00000000126
mmu_circ_0000552	mmu-miR-3061-5	ENSMUST00000108783	ENSMUSG00000000126
mmu_circ_0000829	mmu-miR-3061-5	ENSMUST00000000128	ENSMUSG00000000126
mmu_circ_0000829	mmu-miR-3061-5	ENSMUST00000108783	ENSMUSG00000000126
mmu_circ_0000714	mmu-miR-3061-5	ENSMUST00000000128	ENSMUSG00000000126
mmu_circ_0000714	mmu-miR-3061-5	ENSMUST00000108783	ENSMUSG00000000126
novel_circ_0001646	mmu-miR-3061-5	ENSMUST00000000128	ENSMUSG00000000126
novel_circ_0001646	mmu-miR-3061-5	ENSMUST00000108783	ENSMUSG00000000126
novel_circ_0002111	mmu-miR-3061-5	ENSMUST00000000128	ENSMUSG00000000126
novel_circ_0002111	mmu-miR-3061-5	ENSMUST00000108783	ENSMUSG00000000126
novel_circ_0002111	mmu-miR-3061-5	ENSMUST00000000128	ENSMUSG00000000126
novel_circ_0002111	mmu-miR-3061-5	ENSMUST00000108783	ENSMUSG00000000126
novel_circ_0002129	mmu-miR-3061-5	ENSMUST00000000128	ENSMUSG00000000126
novel_circ_0002129	mmu-miR-3061-5	ENSMUST00000108783	ENSMUSG00000000126
novel_circ_0002188	mmu-miR-3061-5	ENSMUST00000000128	ENSMUSG00000000126
novel_circ_0002188	mmu-miR-3061-5	ENSMUST00000108783	ENSMUSG00000000126
novel_circ_0002188	mmu-miR-3061-5	ENSMUST00000000128	ENSMUSG00000000126
novel_circ_0002188	mmu-miR-3061-5	ENSMUST00000108783	ENSMUSG00000000126
novel_circ_0001757	mmu-miR-3061-5	ENSMUST00000000128	ENSMUSG00000000126
novel_circ_0001757	mmu-miR-3061-5	ENSMUST00000108783	ENSMUSG00000000126
novel_circ_0001905	mmu-miR-3061-5	ENSMUST00000000128	ENSMUSG00000000126
novel_circ_0001905	mmu-miR-3061-5	ENSMUST00000108783	ENSMUSG00000000126
novel_circ_0001914	mmu-miR-3061-5	ENSMUST00000000128	ENSMUSG00000000126
novel_circ_0001914	mmu-miR-3061-5	ENSMUST00000108783	ENSMUSG00000000126
novel_circ_0001935	mmu-miR-3061-5	ENSMUST00000000128	ENSMUSG00000000126
novel_circ_0001935	mmu-miR-3061-5	ENSMUST00000108783	ENSMUSG00000000126
novel_circ_0002043	mmu-miR-3061-5	ENSMUST00000000128	ENSMUSG00000000126
novel_circ_0002043	mmu-miR-3061-5	ENSMUST00000108783	ENSMUSG00000000126
novel_circ_0002452	mmu-miR-3061-5	ENSMUST00000000128	ENSMUSG00000000126
novel_circ_0002452	mmu-miR-3061-5	ENSMUST00000108783	ENSMUSG00000000126

novel_circ_0002519	mmu-miR-3061-5	ENSMUST00000000128	ENSMUSG00000000126
novel_circ_0002519	mmu-miR-3061-5	ENSMUST00000108783	ENSMUSG00000000126
novel_circ_0002597	mmu-miR-3061-5	ENSMUST00000000128	ENSMUSG00000000126
novel_circ_0002597	mmu-miR-3061-5	ENSMUST00000108783	ENSMUSG00000000126
novel_circ_0002879	mmu-miR-3061-5	ENSMUST00000000128	ENSMUSG00000000126
novel_circ_0002879	mmu-miR-3061-5	ENSMUST00000108783	ENSMUSG00000000126
novel_circ_0002884	mmu-miR-3061-5	ENSMUST00000000128	ENSMUSG00000000126
novel_circ_0002884	mmu-miR-3061-5	ENSMUST00000108783	ENSMUSG00000000126
novel_circ_0002884	mmu-miR-3061-5	ENSMUST00000000128	ENSMUSG00000000126
novel_circ_0002884	mmu-miR-3061-5	ENSMUST00000108783	ENSMUSG00000000126
novel_circ_0002925	mmu-miR-3061-5	ENSMUST00000000128	ENSMUSG00000000126
novel_circ_0002925	mmu-miR-3061-5	ENSMUST00000108783	ENSMUSG00000000126
novel_circ_0002925	mmu-miR-3061-5	ENSMUST00000000128	ENSMUSG00000000126
novel_circ_0002925	mmu-miR-3061-5	ENSMUST00000108783	ENSMUSG00000000126
novel_circ_0002677	mmu-miR-3061-5	ENSMUST00000000128	ENSMUSG00000000126
novel_circ_0002677	mmu-miR-3061-5	ENSMUST00000108783	ENSMUSG00000000126
novel_circ_0002682	mmu-miR-3061-5	ENSMUST00000000128	ENSMUSG00000000126
novel_circ_0002682	mmu-miR-3061-5	ENSMUST00000108783	ENSMUSG00000000126
novel_circ_0002719	mmu-miR-3061-5	ENSMUST00000000128	ENSMUSG00000000126
novel_circ_0002719	mmu-miR-3061-5	ENSMUST00000108783	ENSMUSG00000000126
novel_circ_0002790	mmu-miR-3061-5	ENSMUST00000000128	ENSMUSG00000000126
novel_circ_0002790	mmu-miR-3061-5	ENSMUST00000108783	ENSMUSG00000000126
novel_circ_0003353	mmu-miR-3061-5	ENSMUST00000000128	ENSMUSG00000000126
novel_circ_0003353	mmu-miR-3061-5	ENSMUST00000108783	ENSMUSG00000000126
novel_circ_0000200	mmu-miR-3061-5	ENSMUST00000000128	ENSMUSG00000000126
novel_circ_0000200	mmu-miR-3061-5	ENSMUST00000108783	ENSMUSG00000000126
novel_circ_0000668	mmu-miR-3061-5	ENSMUST00000000128	ENSMUSG00000000126
novel_circ_0000668	mmu-miR-3061-5	ENSMUST00000108783	ENSMUSG00000000126
novel_circ_0000692	mmu-miR-3061-5	ENSMUST00000000128	ENSMUSG00000000126
novel_circ_0000692	mmu-miR-3061-5	ENSMUST00000108783	ENSMUSG00000000126
novel_circ_0000703	mmu-miR-3061-5	ENSMUST00000000128	ENSMUSG00000000126
novel_circ_0000703	mmu-miR-3061-5	ENSMUST00000108783	ENSMUSG00000000126
novel_circ_0000780	mmu-miR-3061-5	ENSMUST00000000128	ENSMUSG00000000126
novel_circ_0000780	mmu-miR-3061-5	ENSMUST00000108783	ENSMUSG00000000126
novel_circ_0000483	mmu-miR-3061-5	ENSMUST00000000128	ENSMUSG00000000126
novel_circ_0000483	mmu-miR-3061-5	ENSMUST00000108783	ENSMUSG00000000126
novel_circ_0001031	mmu-miR-3061-5	ENSMUST00000000128	ENSMUSG00000000126
novel_circ_0001031	mmu-miR-3061-5	ENSMUST00000108783	ENSMUSG00000000126
novel_circ_0001227	mmu-miR-3072-5	ENSMUST00000003659	ENSMUSG00000031849
novel_circ_0001227	mmu-miR-3072-5	ENSMUST00000212488	ENSMUSG00000031849
novel_circ_0001438	mmu-miR-3072-5	ENSMUST00000003659	ENSMUSG00000031849
novel_circ_0001438	mmu-miR-3072-5	ENSMUST00000212488	ENSMUSG00000031849
novel_circ_0001454	mmu-miR-3072-5	ENSMUST00000003659	ENSMUSG00000031849
novel_circ_0001454	mmu-miR-3072-5	ENSMUST00000212488	ENSMUSG00000031849
novel_circ_0001455	mmu-miR-3072-5	ENSMUST00000003659	ENSMUSG00000031849
novel_circ_0001455	mmu-miR-3072-5	ENSMUST00000212488	ENSMUSG00000031849
mmu_circ_0001336	mmu-miR-3072-5	ENSMUST00000003659	ENSMUSG00000031849
mmu_circ_0001336	mmu-miR-3072-5	ENSMUST00000212488	ENSMUSG00000031849
mmu_circ_0001283	mmu-miR-3072-5	ENSMUST00000003659	ENSMUSG00000031849
mmu_circ_0001283	mmu-miR-3072-5	ENSMUST00000212488	ENSMUSG00000031849
mmu_circ_0001454	mmu-miR-3072-5	ENSMUST00000003659	ENSMUSG00000031849
mmu_circ_0001454	mmu-miR-3072-5	ENSMUST00000212488	ENSMUSG00000031849
mmu_circ_0001499	mmu-miR-3072-5	ENSMUST00000003659	ENSMUSG00000031849
mmu_circ_0001499	mmu-miR-3072-5	ENSMUST00000212488	ENSMUSG00000031849
mmu_circ_0001500	mmu-miR-3072-5	ENSMUST00000003659	ENSMUSG00000031849
mmu_circ_0001500	mmu-miR-3072-5	ENSMUST00000212488	ENSMUSG00000031849
mmu_circ_0000499	mmu-miR-3072-5	ENSMUST00000003659	ENSMUSG00000031849
mmu_circ_0000499	mmu-miR-3072-5	ENSMUST00000212488	ENSMUSG00000031849

mmu_circ_0000399	mmu-miR-3072-5	ENSMUST00000003659	ENSMUSG00000031849
mmu_circ_0000399	mmu-miR-3072-5	ENSMUST00000212488	ENSMUSG00000031849
mmu_circ_0000628	mmu-miR-3072-5	ENSMUST00000003659	ENSMUSG00000031849
mmu_circ_0000628	mmu-miR-3072-5	ENSMUST00000212488	ENSMUSG00000031849
mmu_circ_0000798	mmu-miR-3072-5	ENSMUST00000003659	ENSMUSG00000031849
mmu_circ_0000798	mmu-miR-3072-5	ENSMUST00000212488	ENSMUSG00000031849
mmu_circ_0000831	mmu-miR-3072-5	ENSMUST00000003659	ENSMUSG00000031849
mmu_circ_0000831	mmu-miR-3072-5	ENSMUST00000212488	ENSMUSG00000031849
mmu_circ_0000712	mmu-miR-3072-5	ENSMUST00000003659	ENSMUSG00000031849
mmu_circ_0000712	mmu-miR-3072-5	ENSMUST00000212488	ENSMUSG00000031849
mmu_circ_0000713	mmu-miR-3072-5	ENSMUST00000003659	ENSMUSG00000031849
mmu_circ_0000713	mmu-miR-3072-5	ENSMUST00000212488	ENSMUSG00000031849
mmu_circ_0000714	mmu-miR-3072-5	ENSMUST00000003659	ENSMUSG00000031849
mmu_circ_0000714	mmu-miR-3072-5	ENSMUST00000212488	ENSMUSG00000031849
mmu_circ_0000969	mmu-miR-3072-5	ENSMUST00000003659	ENSMUSG00000031849
mmu_circ_0000969	mmu-miR-3072-5	ENSMUST00000212488	ENSMUSG00000031849
mmu_circ_0000884	mmu-miR-3072-5	ENSMUST00000003659	ENSMUSG00000031849
mmu_circ_0000884	mmu-miR-3072-5	ENSMUST00000212488	ENSMUSG00000031849
mmu_circ_0000886	mmu-miR-3072-5	ENSMUST00000003659	ENSMUSG00000031849
mmu_circ_0000886	mmu-miR-3072-5	ENSMUST00000212488	ENSMUSG00000031849
novel_circ_0001672	mmu-miR-3072-5	ENSMUST00000003659	ENSMUSG00000031849
novel_circ_0001672	mmu-miR-3072-5	ENSMUST00000212488	ENSMUSG00000031849
novel_circ_0001880	mmu-miR-3072-5	ENSMUST00000003659	ENSMUSG00000031849
novel_circ_0001880	mmu-miR-3072-5	ENSMUST00000212488	ENSMUSG00000031849
novel_circ_0002481	mmu-miR-3072-5	ENSMUST00000003659	ENSMUSG00000031849
novel_circ_0002481	mmu-miR-3072-5	ENSMUST00000212488	ENSMUSG00000031849
novel_circ_0002482	mmu-miR-3072-5	ENSMUST00000003659	ENSMUSG00000031849
novel_circ_0002482	mmu-miR-3072-5	ENSMUST00000212488	ENSMUSG00000031849
novel_circ_0002530	mmu-miR-3072-5	ENSMUST00000003659	ENSMUSG00000031849
novel_circ_0002530	mmu-miR-3072-5	ENSMUST00000212488	ENSMUSG00000031849
novel_circ_0002925	mmu-miR-3072-5	ENSMUST00000003659	ENSMUSG00000031849
novel_circ_0002925	mmu-miR-3072-5	ENSMUST00000212488	ENSMUSG00000031849
novel_circ_0002925	mmu-miR-3072-5	ENSMUST00000003659	ENSMUSG00000031849
novel_circ_0002925	mmu-miR-3072-5	ENSMUST00000212488	ENSMUSG00000031849
novel_circ_0002973	mmu-miR-3072-5	ENSMUST00000003659	ENSMUSG00000031849
novel_circ_0002973	mmu-miR-3072-5	ENSMUST00000212488	ENSMUSG00000031849
novel_circ_0002656	mmu-miR-3072-5	ENSMUST00000003659	ENSMUSG00000031849
novel_circ_0002656	mmu-miR-3072-5	ENSMUST00000212488	ENSMUSG00000031849
novel_circ_0002657	mmu-miR-3072-5	ENSMUST00000003659	ENSMUSG00000031849
novel_circ_0002657	mmu-miR-3072-5	ENSMUST00000212488	ENSMUSG00000031849
novel_circ_0002658	mmu-miR-3072-5	ENSMUST00000003659	ENSMUSG00000031849
novel_circ_0002658	mmu-miR-3072-5	ENSMUST00000212488	ENSMUSG00000031849
novel_circ_0003022	mmu-miR-3072-5	ENSMUST00000003659	ENSMUSG00000031849
novel_circ_0003022	mmu-miR-3072-5	ENSMUST00000212488	ENSMUSG00000031849
novel_circ_0003083	mmu-miR-3072-5	ENSMUST00000003659	ENSMUSG00000031849
novel_circ_0003083	mmu-miR-3072-5	ENSMUST00000212488	ENSMUSG00000031849
novel_circ_0003142	mmu-miR-3072-5	ENSMUST00000003659	ENSMUSG00000031849
novel_circ_0003142	mmu-miR-3072-5	ENSMUST00000212488	ENSMUSG00000031849
novel_circ_0000200	mmu-miR-3072-5	ENSMUST00000003659	ENSMUSG00000031849
novel_circ_0000200	mmu-miR-3072-5	ENSMUST00000212488	ENSMUSG00000031849
novel_circ_0000447	mmu-miR-3072-5	ENSMUST00000003659	ENSMUSG00000031849
novel_circ_0000447	mmu-miR-3072-5	ENSMUST00000212488	ENSMUSG00000031849
novel_circ_0000054	mmu-miR-3072-5	ENSMUST00000003659	ENSMUSG00000031849
novel_circ_0000054	mmu-miR-3072-5	ENSMUST00000212488	ENSMUSG00000031849
novel_circ_0000054	mmu-miR-3072-5	ENSMUST00000003659	ENSMUSG00000031849
novel_circ_0000054	mmu-miR-3072-5	ENSMUST00000212488	ENSMUSG00000031849
novel_circ_0000486	mmu-miR-3072-5	ENSMUST00000003659	ENSMUSG00000031849
novel_circ_0000486	mmu-miR-3072-5	ENSMUST00000212488	ENSMUSG00000031849

novel_circ_0000486	mmu-miR-3072-5	ENSMUST00000003659	ENSMUSG00000031849
novel_circ_0000486	mmu-miR-3072-5	ENSMUST00000212488	ENSMUSG00000031849
novel_circ_0001106	mmu-miR-3072-3	ENSMUST00000225594	ENSMUSG00000017491
novel_circ_0001454	mmu-miR-3072-3	ENSMUST00000225594	ENSMUSG00000017491
novel_circ_0001455	mmu-miR-3072-3	ENSMUST00000225594	ENSMUSG00000017491
mmu_circ_0000059	mmu-miR-3072-3	ENSMUST00000225594	ENSMUSG00000017491
mmu_circ_0000060	mmu-miR-3072-3	ENSMUST00000225594	ENSMUSG00000017491
mmu_circ_0001076	mmu-miR-3072-3	ENSMUST00000225594	ENSMUSG00000017491
mmu_circ_0001077	mmu-miR-3072-3	ENSMUST00000225594	ENSMUSG00000017491
mmu_circ_0001369	mmu-miR-3072-3	ENSMUST00000225594	ENSMUSG00000017491
mmu_circ_0001376	mmu-miR-3072-3	ENSMUST00000225594	ENSMUSG00000017491
mmu_circ_0001634	mmu-miR-3072-3	ENSMUST00000225594	ENSMUSG00000017491
mmu_circ_0001470	mmu-miR-3072-3	ENSMUST00000225594	ENSMUSG00000017491
mmu_circ_0001775	mmu-miR-3072-3	ENSMUST00000225594	ENSMUSG00000017491
mmu_circ_0001827	mmu-miR-3072-3	ENSMUST00000225594	ENSMUSG00000017491
mmu_circ_0000499	mmu-miR-3072-3	ENSMUST00000225594	ENSMUSG00000017491
mmu_circ_0000454	mmu-miR-3072-3	ENSMUST00000225594	ENSMUSG00000017491
mmu_circ_0000402	mmu-miR-3072-3	ENSMUST00000225594	ENSMUSG00000017491
mmu_circ_0000628	mmu-miR-3072-3	ENSMUST00000225594	ENSMUSG00000017491
mmu_circ_0000530	mmu-miR-3072-3	ENSMUST00000225594	ENSMUSG00000017491
mmu_circ_0000819	mmu-miR-3072-3	ENSMUST00000225594	ENSMUSG00000017491
mmu_circ_0000833	mmu-miR-3072-3	ENSMUST00000225594	ENSMUSG00000017491
mmu_circ_0000950	mmu-miR-3072-3	ENSMUST00000225594	ENSMUSG00000017491
mmu_circ_0000962	mmu-miR-3072-3	ENSMUST00000225594	ENSMUSG00000017491
mmu_circ_0000963	mmu-miR-3072-3	ENSMUST00000225594	ENSMUSG00000017491
mmu_circ_0000969	mmu-miR-3072-3	ENSMUST00000225594	ENSMUSG00000017491
mmu_circ_0000884	mmu-miR-3072-3	ENSMUST00000225594	ENSMUSG00000017491
mmu_circ_0000886	mmu-miR-3072-3	ENSMUST00000225594	ENSMUSG00000017491
mmu_circ_0000887	mmu-miR-3072-3	ENSMUST00000225594	ENSMUSG00000017491
mmu_circ_0000912	mmu-miR-3072-3	ENSMUST00000225594	ENSMUSG00000017491
novel_circ_0001626	mmu-miR-3072-3	ENSMUST00000225594	ENSMUSG00000017491
novel_circ_0001751	mmu-miR-3072-3	ENSMUST00000225594	ENSMUSG00000017491
novel_circ_0001943	mmu-miR-3072-3	ENSMUST00000225594	ENSMUSG00000017491
novel_circ_0002295	mmu-miR-3072-3	ENSMUST00000225594	ENSMUSG00000017491
novel_circ_0002336	mmu-miR-3072-3	ENSMUST00000225594	ENSMUSG00000017491
novel_circ_0002871	mmu-miR-3072-3	ENSMUST00000225594	ENSMUSG00000017491
novel_circ_0002675	mmu-miR-3072-3	ENSMUST00000225594	ENSMUSG00000017491
novel_circ_0002743	mmu-miR-3072-3	ENSMUST00000225594	ENSMUSG00000017491
novel_circ_0003337	mmu-miR-3072-3	ENSMUST00000225594	ENSMUSG00000017491
novel_circ_0003083	mmu-miR-3072-3	ENSMUST00000225594	ENSMUSG00000017491
novel_circ_0000411	mmu-miR-3072-3	ENSMUST00000225594	ENSMUSG00000017491
novel_circ_0000773	mmu-miR-3072-3	ENSMUST00000225594	ENSMUSG00000017491
novel_circ_0000774	mmu-miR-3072-3	ENSMUST00000225594	ENSMUSG00000017491
novel_circ_0000589	mmu-miR-3072-3	ENSMUST00000225594	ENSMUSG00000017491
novel_circ_0001031	mmu-miR-3072-3	ENSMUST00000225594	ENSMUSG00000017491
novel_circ_0000885	mmu-miR-3072-3	ENSMUST00000225594	ENSMUSG00000017491
novel_circ_0001278	mmu-miR-3076-5	ENSMUST00000000128	ENSMUSG00000000126
novel_circ_0001278	mmu-miR-3076-5	ENSMUST00000108783	ENSMUSG00000000126
novel_circ_0001280	mmu-miR-3076-5	ENSMUST00000000128	ENSMUSG00000000126
novel_circ_0001280	mmu-miR-3076-5	ENSMUST00000108783	ENSMUSG00000000126
novel_circ_0001290	mmu-miR-3076-5	ENSMUST00000000128	ENSMUSG00000000126
novel_circ_0001290	mmu-miR-3076-5	ENSMUST00000108783	ENSMUSG00000000126
novel_circ_0001293	mmu-miR-3076-5	ENSMUST00000000128	ENSMUSG00000000126
novel_circ_0001293	mmu-miR-3076-5	ENSMUST00000108783	ENSMUSG00000000126
novel_circ_0001139	mmu-miR-3076-5	ENSMUST00000000128	ENSMUSG00000000126
novel_circ_0001139	mmu-miR-3076-5	ENSMUST00000108783	ENSMUSG00000000126
novel_circ_0001447	mmu-miR-3076-5	ENSMUST00000000128	ENSMUSG00000000126
novel_circ_0001447	mmu-miR-3076-5	ENSMUST00000108783	ENSMUSG00000000126

mmu_circ_0001154	mmu-miR-3076-5	ENSMUST00000000128	ENSMUSG00000000126
mmu_circ_0001154	mmu-miR-3076-5	ENSMUST00000108783	ENSMUSG00000000126
mmu_circ_0001376	mmu-miR-3076-5	ENSMUST00000000128	ENSMUSG00000000126
mmu_circ_0001376	mmu-miR-3076-5	ENSMUST00000108783	ENSMUSG00000000126
mmu_circ_0001387	mmu-miR-3076-5	ENSMUST00000000128	ENSMUSG00000000126
mmu_circ_0001387	mmu-miR-3076-5	ENSMUST00000108783	ENSMUSG00000000126
mmu_circ_0001432	mmu-miR-3076-5	ENSMUST00000000128	ENSMUSG00000000126
mmu_circ_0001432	mmu-miR-3076-5	ENSMUST00000108783	ENSMUSG00000000126
mmu_circ_0001432	mmu-miR-3076-5	ENSMUST00000000128	ENSMUSG00000000126
mmu_circ_0001432	mmu-miR-3076-5	ENSMUST00000108783	ENSMUSG00000000126
mmu_circ_0001329	mmu-miR-3076-5	ENSMUST00000000128	ENSMUSG00000000126
mmu_circ_0001329	mmu-miR-3076-5	ENSMUST00000108783	ENSMUSG00000000126
mmu_circ_0001263	mmu-miR-3076-5	ENSMUST00000000128	ENSMUSG00000000126
mmu_circ_0001263	mmu-miR-3076-5	ENSMUST00000108783	ENSMUSG00000000126
mmu_circ_0001264	mmu-miR-3076-5	ENSMUST00000000128	ENSMUSG00000000126
mmu_circ_0001264	mmu-miR-3076-5	ENSMUST00000108783	ENSMUSG00000000126
mmu_circ_0001634	mmu-miR-3076-5	ENSMUST00000000128	ENSMUSG00000000126
mmu_circ_0001634	mmu-miR-3076-5	ENSMUST00000108783	ENSMUSG00000000126
mmu_circ_0001516	mmu-miR-3076-5	ENSMUST00000000128	ENSMUSG00000000126
mmu_circ_0001516	mmu-miR-3076-5	ENSMUST00000108783	ENSMUSG00000000126
mmu_circ_0001528	mmu-miR-3076-5	ENSMUST00000000128	ENSMUSG00000000126
mmu_circ_0001528	mmu-miR-3076-5	ENSMUST00000108783	ENSMUSG00000000126
mmu_circ_0001454	mmu-miR-3076-5	ENSMUST00000000128	ENSMUSG00000000126
mmu_circ_0001454	mmu-miR-3076-5	ENSMUST00000108783	ENSMUSG00000000126
mmu_circ_0001500	mmu-miR-3076-5	ENSMUST00000000128	ENSMUSG00000000126
mmu_circ_0001500	mmu-miR-3076-5	ENSMUST00000108783	ENSMUSG00000000126
mmu_circ_0000230	mmu-miR-3076-5	ENSMUST00000000128	ENSMUSG00000000126
mmu_circ_0000230	mmu-miR-3076-5	ENSMUST00000108783	ENSMUSG00000000126
mmu_circ_0000230	mmu-miR-3076-5	ENSMUST00000000128	ENSMUSG00000000126
mmu_circ_0000230	mmu-miR-3076-5	ENSMUST00000108783	ENSMUSG00000000126
mmu_circ_0000454	mmu-miR-3076-5	ENSMUST00000000128	ENSMUSG00000000126
mmu_circ_0000454	mmu-miR-3076-5	ENSMUST00000108783	ENSMUSG00000000126
mmu_circ_0000530	mmu-miR-3076-5	ENSMUST00000000128	ENSMUSG00000000126
mmu_circ_0000530	mmu-miR-3076-5	ENSMUST00000108783	ENSMUSG00000000126
mmu_circ_0000735	mmu-miR-3076-5	ENSMUST00000000128	ENSMUSG00000000126
mmu_circ_0000735	mmu-miR-3076-5	ENSMUST00000108783	ENSMUSG00000000126
mmu_circ_0000819	mmu-miR-3076-5	ENSMUST00000000128	ENSMUSG00000000126
mmu_circ_0000819	mmu-miR-3076-5	ENSMUST00000108783	ENSMUSG00000000126
mmu_circ_0000820	mmu-miR-3076-5	ENSMUST00000000128	ENSMUSG00000000126
mmu_circ_0000820	mmu-miR-3076-5	ENSMUST00000108783	ENSMUSG00000000126
mmu_circ_0000828	mmu-miR-3076-5	ENSMUST00000000128	ENSMUSG00000000126
mmu_circ_0000828	mmu-miR-3076-5	ENSMUST00000108783	ENSMUSG00000000126
mmu_circ_0000829	mmu-miR-3076-5	ENSMUST00000000128	ENSMUSG00000000126
mmu_circ_0000829	mmu-miR-3076-5	ENSMUST00000108783	ENSMUSG00000000126
mmu_circ_0000831	mmu-miR-3076-5	ENSMUST00000000128	ENSMUSG00000000126
mmu_circ_0000831	mmu-miR-3076-5	ENSMUST00000108783	ENSMUSG00000000126
mmu_circ_0000712	mmu-miR-3076-5	ENSMUST00000000128	ENSMUSG00000000126
mmu_circ_0000712	mmu-miR-3076-5	ENSMUST00000108783	ENSMUSG00000000126
mmu_circ_0000713	mmu-miR-3076-5	ENSMUST00000000128	ENSMUSG00000000126
mmu_circ_0000713	mmu-miR-3076-5	ENSMUST00000108783	ENSMUSG00000000126
mmu_circ_0000714	mmu-miR-3076-5	ENSMUST00000000128	ENSMUSG00000000126
mmu_circ_0000714	mmu-miR-3076-5	ENSMUST00000108783	ENSMUSG00000000126
mmu_circ_0000969	mmu-miR-3076-5	ENSMUST00000000128	ENSMUSG00000000126
mmu_circ_0000969	mmu-miR-3076-5	ENSMUST00000108783	ENSMUSG00000000126
mmu_circ_0000874	mmu-miR-3076-5	ENSMUST00000000128	ENSMUSG00000000126
mmu_circ_0000874	mmu-miR-3076-5	ENSMUST00000108783	ENSMUSG00000000126
mmu_circ_0000874	mmu-miR-3076-5	ENSMUST00000000128	ENSMUSG00000000126
mmu_circ_0000874	mmu-miR-3076-5	ENSMUST00000108783	ENSMUSG00000000126

mmu_circ_0000884	mmu-miR-3076-5	ENSMUST00000000128	ENSMUSG00000000126
mmu_circ_0000884	mmu-miR-3076-5	ENSMUST00000108783	ENSMUSG00000000126
mmu_circ_0000886	mmu-miR-3076-5	ENSMUST00000000128	ENSMUSG00000000126
mmu_circ_0000886	mmu-miR-3076-5	ENSMUST00000108783	ENSMUSG00000000126
mmu_circ_0000887	mmu-miR-3076-5	ENSMUST00000000128	ENSMUSG00000000126
mmu_circ_0000887	mmu-miR-3076-5	ENSMUST00000108783	ENSMUSG00000000126
mmu_circ_0000905	mmu-miR-3076-5	ENSMUST00000000128	ENSMUSG00000000126
mmu_circ_0000905	mmu-miR-3076-5	ENSMUST00000108783	ENSMUSG00000000126
mmu_circ_0000906	mmu-miR-3076-5	ENSMUST00000000128	ENSMUSG00000000126
mmu_circ_0000906	mmu-miR-3076-5	ENSMUST00000108783	ENSMUSG00000000126
mmu_circ_0000907	mmu-miR-3076-5	ENSMUST00000000128	ENSMUSG00000000126
mmu_circ_0000907	mmu-miR-3076-5	ENSMUST00000108783	ENSMUSG00000000126
novel_circ_0001619	mmu-miR-3076-5	ENSMUST00000000128	ENSMUSG00000000126
novel_circ_0001619	mmu-miR-3076-5	ENSMUST00000108783	ENSMUSG00000000126
novel_circ_0001676	mmu-miR-3076-5	ENSMUST00000000128	ENSMUSG00000000126
novel_circ_0001676	mmu-miR-3076-5	ENSMUST00000108783	ENSMUSG00000000126
novel_circ_0002055	mmu-miR-3076-5	ENSMUST00000000128	ENSMUSG00000000126
novel_circ_0002055	mmu-miR-3076-5	ENSMUST00000108783	ENSMUSG00000000126
novel_circ_0002129	mmu-miR-3076-5	ENSMUST00000000128	ENSMUSG00000000126
novel_circ_0002129	mmu-miR-3076-5	ENSMUST00000108783	ENSMUSG00000000126
novel_circ_0002191	mmu-miR-3076-5	ENSMUST00000000128	ENSMUSG00000000126
novel_circ_0002191	mmu-miR-3076-5	ENSMUST00000108783	ENSMUSG00000000126
novel_circ_0002191	mmu-miR-3076-5	ENSMUST00000000128	ENSMUSG00000000126
novel_circ_0002191	mmu-miR-3076-5	ENSMUST00000108783	ENSMUSG00000000126
novel_circ_0002195	mmu-miR-3076-5	ENSMUST00000000128	ENSMUSG00000000126
novel_circ_0002195	mmu-miR-3076-5	ENSMUST00000108783	ENSMUSG00000000126
novel_circ_0002198	mmu-miR-3076-5	ENSMUST00000000128	ENSMUSG00000000126
novel_circ_0002198	mmu-miR-3076-5	ENSMUST00000108783	ENSMUSG00000000126
novel_circ_0002216	mmu-miR-3076-5	ENSMUST00000000128	ENSMUSG00000000126
novel_circ_0002216	mmu-miR-3076-5	ENSMUST00000108783	ENSMUSG00000000126
novel_circ_0002217	mmu-miR-3076-5	ENSMUST00000000128	ENSMUSG00000000126
novel_circ_0002217	mmu-miR-3076-5	ENSMUST00000108783	ENSMUSG00000000126
novel_circ_0001751	mmu-miR-3076-5	ENSMUST00000000128	ENSMUSG00000000126
novel_circ_0001751	mmu-miR-3076-5	ENSMUST00000108783	ENSMUSG00000000126
novel_circ_0001875	mmu-miR-3076-5	ENSMUST00000000128	ENSMUSG00000000126
novel_circ_0001875	mmu-miR-3076-5	ENSMUST00000108783	ENSMUSG00000000126
novel_circ_0001980	mmu-miR-3076-5	ENSMUST00000000128	ENSMUSG00000000126
novel_circ_0001980	mmu-miR-3076-5	ENSMUST00000108783	ENSMUSG00000000126
novel_circ_0002027	mmu-miR-3076-5	ENSMUST00000000128	ENSMUSG00000000126
novel_circ_0002027	mmu-miR-3076-5	ENSMUST00000108783	ENSMUSG00000000126
novel_circ_0002027	mmu-miR-3076-5	ENSMUST00000000128	ENSMUSG00000000126
novel_circ_0002027	mmu-miR-3076-5	ENSMUST00000108783	ENSMUSG00000000126
novel_circ_0002412	mmu-miR-3076-5	ENSMUST00000000128	ENSMUSG00000000126
novel_circ_0002412	mmu-miR-3076-5	ENSMUST00000108783	ENSMUSG00000000126
novel_circ_0002417	mmu-miR-3076-5	ENSMUST00000000128	ENSMUSG00000000126
novel_circ_0002417	mmu-miR-3076-5	ENSMUST00000108783	ENSMUSG00000000126
novel_circ_0002494	mmu-miR-3076-5	ENSMUST00000000128	ENSMUSG00000000126
novel_circ_0002494	mmu-miR-3076-5	ENSMUST00000108783	ENSMUSG00000000126
novel_circ_0002503	mmu-miR-3076-5	ENSMUST00000000128	ENSMUSG00000000126
novel_circ_0002503	mmu-miR-3076-5	ENSMUST00000108783	ENSMUSG00000000126
novel_circ_0002504	mmu-miR-3076-5	ENSMUST00000000128	ENSMUSG00000000126
novel_circ_0002504	mmu-miR-3076-5	ENSMUST00000108783	ENSMUSG00000000126
novel_circ_0002346	mmu-miR-3076-5	ENSMUST00000000128	ENSMUSG00000000126
novel_circ_0002346	mmu-miR-3076-5	ENSMUST00000108783	ENSMUSG00000000126
novel_circ_0002885	mmu-miR-3076-5	ENSMUST00000000128	ENSMUSG00000000126
novel_circ_0002885	mmu-miR-3076-5	ENSMUST00000108783	ENSMUSG00000000126
novel_circ_0002998	mmu-miR-3076-5	ENSMUST00000000128	ENSMUSG00000000126
novel_circ_0002998	mmu-miR-3076-5	ENSMUST00000108783	ENSMUSG00000000126

novel_circ_0002654	mmu-miR-3076-5	ENSMUST00000000128	ENSMUSG00000000126
novel_circ_0002654	mmu-miR-3076-5	ENSMUST00000108783	ENSMUSG00000000126
novel_circ_0002769	mmu-miR-3076-5	ENSMUST00000000128	ENSMUSG00000000126
novel_circ_0002769	mmu-miR-3076-5	ENSMUST00000108783	ENSMUSG00000000126
novel_circ_0002769	mmu-miR-3076-5	ENSMUST00000000128	ENSMUSG00000000126
novel_circ_0002769	mmu-miR-3076-5	ENSMUST00000108783	ENSMUSG00000000126
novel_circ_0003054	mmu-miR-3076-5	ENSMUST00000000128	ENSMUSG00000000126
novel_circ_0003054	mmu-miR-3076-5	ENSMUST00000108783	ENSMUSG00000000126
novel_circ_0003129	mmu-miR-3076-5	ENSMUST00000000128	ENSMUSG00000000126
novel_circ_0003129	mmu-miR-3076-5	ENSMUST00000108783	ENSMUSG00000000126
novel_circ_0000218	mmu-miR-3076-5	ENSMUST00000000128	ENSMUSG00000000126
novel_circ_0000218	mmu-miR-3076-5	ENSMUST00000108783	ENSMUSG00000000126
novel_circ_0000291	mmu-miR-3076-5	ENSMUST00000000128	ENSMUSG00000000126
novel_circ_0000291	mmu-miR-3076-5	ENSMUST00000108783	ENSMUSG00000000126
novel_circ_0000292	mmu-miR-3076-5	ENSMUST00000000128	ENSMUSG00000000126
novel_circ_0000292	mmu-miR-3076-5	ENSMUST00000108783	ENSMUSG00000000126
novel_circ_0000113	mmu-miR-3076-5	ENSMUST00000000128	ENSMUSG00000000126
novel_circ_0000113	mmu-miR-3076-5	ENSMUST00000108783	ENSMUSG00000000126
novel_circ_0000631	mmu-miR-3076-5	ENSMUST00000000128	ENSMUSG00000000126
novel_circ_0000631	mmu-miR-3076-5	ENSMUST00000108783	ENSMUSG00000000126
novel_circ_0000733	mmu-miR-3076-5	ENSMUST00000000128	ENSMUSG00000000126
novel_circ_0000733	mmu-miR-3076-5	ENSMUST00000108783	ENSMUSG00000000126
novel_circ_0000530	mmu-miR-3076-5	ENSMUST00000000128	ENSMUSG00000000126
novel_circ_0000530	mmu-miR-3076-5	ENSMUST00000108783	ENSMUSG00000000126
novel_circ_0000608	mmu-miR-3076-5	ENSMUST00000000128	ENSMUSG00000000126
novel_circ_0000608	mmu-miR-3076-5	ENSMUST00000108783	ENSMUSG00000000126
novel_circ_0000835	mmu-miR-3076-5	ENSMUST00000000128	ENSMUSG00000000126
novel_circ_0000835	mmu-miR-3076-5	ENSMUST00000108783	ENSMUSG00000000126
novel_circ_0000848	mmu-miR-3076-5	ENSMUST00000000128	ENSMUSG00000000126
novel_circ_0000848	mmu-miR-3076-5	ENSMUST00000108783	ENSMUSG00000000126
novel_circ_0001164	mmu-miR-3076-5	ENSMUST00000000128	ENSMUSG00000000126
novel_circ_0001164	mmu-miR-3076-5	ENSMUST00000108783	ENSMUSG00000000126
novel_circ_0001171	mmu-miR-3076-5	ENSMUST00000000128	ENSMUSG00000000126
novel_circ_0001171	mmu-miR-3076-5	ENSMUST00000108783	ENSMUSG00000000126
novel_circ_0001177	mmu-miR-3076-5	ENSMUST00000000128	ENSMUSG00000000126
novel_circ_0001177	mmu-miR-3076-5	ENSMUST00000108783	ENSMUSG00000000126
novel_circ_0001179	mmu-miR-3076-5	ENSMUST00000000128	ENSMUSG00000000126
novel_circ_0001179	mmu-miR-3076-5	ENSMUST00000108783	ENSMUSG00000000126
novel_circ_0001072	mmu-miR-3100-5	ENSMUST00000213072	ENSMUSG00000031849
novel_circ_0001072	mmu-miR-3100-5	ENSMUST00000213072	ENSMUSG00000031849
novel_circ_0001471	mmu-miR-3100-5	ENSMUST00000213072	ENSMUSG00000031849
novel_circ_0001318	mmu-miR-3100-5	ENSMUST00000213072	ENSMUSG00000031849
mmu_circ_0001123	mmu-miR-3100-5	ENSMUST00000213072	ENSMUSG00000031849
mmu_circ_0001124	mmu-miR-3100-5	ENSMUST00000213072	ENSMUSG00000031849
mmu_circ_0001125	mmu-miR-3100-5	ENSMUST00000213072	ENSMUSG00000031849
mmu_circ_0001148	mmu-miR-3100-5	ENSMUST00000213072	ENSMUSG00000031849
mmu_circ_0001148	mmu-miR-3100-5	ENSMUST00000213072	ENSMUSG00000031849
mmu_circ_0001021	mmu-miR-3100-5	ENSMUST00000213072	ENSMUSG00000031849
mmu_circ_0001387	mmu-miR-3100-5	ENSMUST00000213072	ENSMUSG00000031849
mmu_circ_0001333	mmu-miR-3100-5	ENSMUST00000213072	ENSMUSG00000031849
mmu_circ_0001255	mmu-miR-3100-5	ENSMUST00000213072	ENSMUSG00000031849
mmu_circ_0001256	mmu-miR-3100-5	ENSMUST00000213072	ENSMUSG00000031849
mmu_circ_0001271	mmu-miR-3100-5	ENSMUST00000213072	ENSMUSG00000031849
mmu_circ_0001213	mmu-miR-3100-5	ENSMUST00000213072	ENSMUSG00000031849
mmu_circ_0001213	mmu-miR-3100-5	ENSMUST00000213072	ENSMUSG00000031849
mmu_circ_0001528	mmu-miR-3100-5	ENSMUST00000213072	ENSMUSG00000031849
mmu_circ_0001529	mmu-miR-3100-5	ENSMUST00000213072	ENSMUSG00000031849
mmu_circ_0001454	mmu-miR-3100-5	ENSMUST00000213072	ENSMUSG00000031849

mmu_circ_0001470	mmu-miR-3100-5	ENSMUST00000213072	ENSMUSG00000031849
mmu_circ_0001501	mmu-miR-3100-5	ENSMUST00000213072	ENSMUSG00000031849
mmu_circ_0001757	mmu-miR-3100-5	ENSMUST00000213072	ENSMUSG00000031849
mmu_circ_0001898	mmu-miR-3100-5	ENSMUST00000213072	ENSMUSG00000031849
mmu_circ_0000239	mmu-miR-3100-5	ENSMUST00000213072	ENSMUSG00000031849
mmu_circ_0000230	mmu-miR-3100-5	ENSMUST00000213072	ENSMUSG00000031849
mmu_circ_0000301	mmu-miR-3100-5	ENSMUST00000213072	ENSMUSG00000031849
mmu_circ_0000495	mmu-miR-3100-5	ENSMUST00000213072	ENSMUSG00000031849
mmu_circ_0000499	mmu-miR-3100-5	ENSMUST00000213072	ENSMUSG00000031849
mmu_circ_0000500	mmu-miR-3100-5	ENSMUST00000213072	ENSMUSG00000031849
mmu_circ_0000454	mmu-miR-3100-5	ENSMUST00000213072	ENSMUSG00000031849
mmu_circ_0000465	mmu-miR-3100-5	ENSMUST00000213072	ENSMUSG00000031849
mmu_circ_0000402	mmu-miR-3100-5	ENSMUST00000213072	ENSMUSG00000031849
mmu_circ_0000628	mmu-miR-3100-5	ENSMUST00000213072	ENSMUSG00000031849
mmu_circ_0000530	mmu-miR-3100-5	ENSMUST00000213072	ENSMUSG00000031849
mmu_circ_0000829	mmu-miR-3100-5	ENSMUST00000213072	ENSMUSG00000031849
mmu_circ_0000831	mmu-miR-3100-5	ENSMUST00000213072	ENSMUSG00000031849
mmu_circ_0000705	mmu-miR-3100-5	ENSMUST00000213072	ENSMUSG00000031849
mmu_circ_0000711	mmu-miR-3100-5	ENSMUST00000213072	ENSMUSG00000031849
mmu_circ_0000712	mmu-miR-3100-5	ENSMUST00000213072	ENSMUSG00000031849
mmu_circ_0000714	mmu-miR-3100-5	ENSMUST00000213072	ENSMUSG00000031849
mmu_circ_0000969	mmu-miR-3100-5	ENSMUST00000213072	ENSMUSG00000031849
mmu_circ_0000854	mmu-miR-3100-5	ENSMUST00000213072	ENSMUSG00000031849
mmu_circ_0000884	mmu-miR-3100-5	ENSMUST00000213072	ENSMUSG00000031849
mmu_circ_0000886	mmu-miR-3100-5	ENSMUST00000213072	ENSMUSG00000031849
mmu_circ_0000887	mmu-miR-3100-5	ENSMUST00000213072	ENSMUSG00000031849
mmu_circ_0000898	mmu-miR-3100-5	ENSMUST00000213072	ENSMUSG00000031849
mmu_circ_0000905	mmu-miR-3100-5	ENSMUST00000213072	ENSMUSG00000031849
mmu_circ_0000906	mmu-miR-3100-5	ENSMUST00000213072	ENSMUSG00000031849
mmu_circ_0000907	mmu-miR-3100-5	ENSMUST00000213072	ENSMUSG00000031849
mmu_circ_0000908	mmu-miR-3100-5	ENSMUST00000213072	ENSMUSG00000031849
novel_circ_0001546	mmu-miR-3100-5	ENSMUST00000213072	ENSMUSG00000031849
novel_circ_0001547	mmu-miR-3100-5	ENSMUST00000213072	ENSMUSG00000031849
novel_circ_0002129	mmu-miR-3100-5	ENSMUST00000213072	ENSMUSG00000031849
novel_circ_0001946	mmu-miR-3100-5	ENSMUST00000213072	ENSMUSG00000031849
novel_circ_0001946	mmu-miR-3100-5	ENSMUST00000213072	ENSMUSG00000031849
novel_circ_0001994	mmu-miR-3100-5	ENSMUST00000213072	ENSMUSG00000031849
novel_circ_0002014	mmu-miR-3100-5	ENSMUST00000213072	ENSMUSG00000031849
novel_circ_0002440	mmu-miR-3100-5	ENSMUST00000213072	ENSMUSG00000031849
novel_circ_0002453	mmu-miR-3100-5	ENSMUST00000213072	ENSMUSG00000031849
novel_circ_0002488	mmu-miR-3100-5	ENSMUST00000213072	ENSMUSG00000031849
novel_circ_0002577	mmu-miR-3100-5	ENSMUST00000213072	ENSMUSG00000031849
novel_circ_0002257	mmu-miR-3100-5	ENSMUST00000213072	ENSMUSG00000031849
novel_circ_0002283	mmu-miR-3100-5	ENSMUST00000213072	ENSMUSG00000031849
novel_circ_0002283	mmu-miR-3100-5	ENSMUST00000213072	ENSMUSG00000031849
novel_circ_0002287	mmu-miR-3100-5	ENSMUST00000213072	ENSMUSG00000031849
novel_circ_0002353	mmu-miR-3100-5	ENSMUST00000213072	ENSMUSG00000031849
novel_circ_0002353	mmu-miR-3100-5	ENSMUST00000213072	ENSMUSG00000031849
novel_circ_0002934	mmu-miR-3100-5	ENSMUST00000213072	ENSMUSG00000031849
novel_circ_0002674	mmu-miR-3100-5	ENSMUST00000213072	ENSMUSG00000031849
novel_circ_0002694	mmu-miR-3100-5	ENSMUST00000213072	ENSMUSG00000031849
novel_circ_0003312	mmu-miR-3100-5	ENSMUST00000213072	ENSMUSG00000031849
novel_circ_0003313	mmu-miR-3100-5	ENSMUST00000213072	ENSMUSG00000031849
novel_circ_0003383	mmu-miR-3100-5	ENSMUST00000213072	ENSMUSG00000031849
novel_circ_0003053	mmu-miR-3100-5	ENSMUST00000213072	ENSMUSG00000031849
novel_circ_0003057	mmu-miR-3100-5	ENSMUST00000213072	ENSMUSG00000031849
novel_circ_0003164	mmu-miR-3100-5	ENSMUST00000213072	ENSMUSG00000031849
novel_circ_0003164	mmu-miR-3100-5	ENSMUST00000213072	ENSMUSG00000031849

novel_circ_0003455	mmu-miR-3100-5	ENSMUST00000213072	ENSMUSG00000031849
novel_circ_0000167	mmu-miR-3100-5	ENSMUST00000213072	ENSMUSG00000031849
novel_circ_0000257	mmu-miR-3100-5	ENSMUST00000213072	ENSMUSG00000031849
novel_circ_0000258	mmu-miR-3100-5	ENSMUST00000213072	ENSMUSG00000031849
novel_circ_0000452	mmu-miR-3100-5	ENSMUST00000213072	ENSMUSG00000031849
novel_circ_0000013	mmu-miR-3100-5	ENSMUST00000213072	ENSMUSG00000031849
novel_circ_0000021	mmu-miR-3100-5	ENSMUST00000213072	ENSMUSG00000031849
novel_circ_0000021	mmu-miR-3100-5	ENSMUST00000213072	ENSMUSG00000031849
novel_circ_0000023	mmu-miR-3100-5	ENSMUST00000213072	ENSMUSG00000031849
novel_circ_0000051	mmu-miR-3100-5	ENSMUST00000213072	ENSMUSG00000031849
novel_circ_0000068	mmu-miR-3100-5	ENSMUST00000213072	ENSMUSG00000031849
novel_circ_0000127	mmu-miR-3100-5	ENSMUST00000213072	ENSMUSG00000031849
novel_circ_0000144	mmu-miR-3100-5	ENSMUST00000213072	ENSMUSG00000031849
novel_circ_0000620	mmu-miR-3100-5	ENSMUST00000213072	ENSMUSG00000031849
novel_circ_0000514	mmu-miR-3100-5	ENSMUST00000213072	ENSMUSG00000031849
novel_circ_0000975	mmu-miR-3100-5	ENSMUST00000213072	ENSMUSG00000031849
novel_circ_0001048	mmu-miR-3100-5	ENSMUST00000213072	ENSMUSG00000031849
novel_circ_0001136	mmu-miR-486b-3	ENSMUST00000000128	ENSMUSG00000000126
novel_circ_0001136	mmu-miR-486b-3	ENSMUST00000108783	ENSMUSG00000000126
novel_circ_0001136	mmu-miR-486b-3	ENSMUST00000213072	ENSMUSG00000031849
novel_circ_0001471	mmu-miR-486b-3	ENSMUST00000000128	ENSMUSG00000000126
novel_circ_0001471	mmu-miR-486b-3	ENSMUST00000108783	ENSMUSG00000000126
novel_circ_0001471	mmu-miR-486b-3	ENSMUST00000213072	ENSMUSG00000031849
novel_circ_0001495	mmu-miR-486b-3	ENSMUST00000000128	ENSMUSG00000000126
novel_circ_0001495	mmu-miR-486b-3	ENSMUST00000108783	ENSMUSG00000000126
novel_circ_0001495	mmu-miR-486b-3	ENSMUST00000213072	ENSMUSG00000031849
novel_circ_0001388	mmu-miR-486b-3	ENSMUST00000000128	ENSMUSG00000000126
novel_circ_0001388	mmu-miR-486b-3	ENSMUST00000108783	ENSMUSG00000000126
novel_circ_0001388	mmu-miR-486b-3	ENSMUST00000213072	ENSMUSG00000031849
novel_circ_0001414	mmu-miR-486b-3	ENSMUST00000000128	ENSMUSG00000000126
novel_circ_0001414	mmu-miR-486b-3	ENSMUST00000108783	ENSMUSG00000000126
novel_circ_0001414	mmu-miR-486b-3	ENSMUST00000213072	ENSMUSG00000031849
novel_circ_0001418	mmu-miR-486b-3	ENSMUST00000000128	ENSMUSG00000000126
novel_circ_0001418	mmu-miR-486b-3	ENSMUST00000108783	ENSMUSG00000000126
novel_circ_0001418	mmu-miR-486b-3	ENSMUST00000213072	ENSMUSG00000031849
mmu_circ_0000040	mmu-miR-486b-3	ENSMUST00000000128	ENSMUSG00000000126
mmu_circ_0000040	mmu-miR-486b-3	ENSMUST00000108783	ENSMUSG00000000126
mmu_circ_0000040	mmu-miR-486b-3	ENSMUST00000213072	ENSMUSG00000031849
mmu_circ_0000041	mmu-miR-486b-3	ENSMUST00000000128	ENSMUSG00000000126
mmu_circ_0000041	mmu-miR-486b-3	ENSMUST00000108783	ENSMUSG00000000126
mmu_circ_0000041	mmu-miR-486b-3	ENSMUST00000213072	ENSMUSG00000031849
mmu_circ_0000063	mmu-miR-486b-3	ENSMUST00000000128	ENSMUSG00000000126
mmu_circ_0000063	mmu-miR-486b-3	ENSMUST00000108783	ENSMUSG00000000126
mmu_circ_0000063	mmu-miR-486b-3	ENSMUST00000213072	ENSMUSG00000031849
mmu_circ_0001376	mmu-miR-486b-3	ENSMUST00000000128	ENSMUSG00000000126
mmu_circ_0001376	mmu-miR-486b-3	ENSMUST00000108783	ENSMUSG00000000126
mmu_circ_0001376	mmu-miR-486b-3	ENSMUST00000213072	ENSMUSG00000031849
mmu_circ_0001331	mmu-miR-486b-3	ENSMUST00000000128	ENSMUSG00000000126
mmu_circ_0001331	mmu-miR-486b-3	ENSMUST00000108783	ENSMUSG00000000126
mmu_circ_0001331	mmu-miR-486b-3	ENSMUST00000213072	ENSMUSG00000031849
mmu_circ_0001333	mmu-miR-486b-3	ENSMUST00000000128	ENSMUSG00000000126
mmu_circ_0001333	mmu-miR-486b-3	ENSMUST00000108783	ENSMUSG00000000126
mmu_circ_0001333	mmu-miR-486b-3	ENSMUST00000213072	ENSMUSG00000031849
mmu_circ_0001255	mmu-miR-486b-3	ENSMUST00000000128	ENSMUSG00000000126
mmu_circ_0001255	mmu-miR-486b-3	ENSMUST00000108783	ENSMUSG00000000126
mmu_circ_0001255	mmu-miR-486b-3	ENSMUST00000213072	ENSMUSG00000031849
mmu_circ_0001256	mmu-miR-486b-3	ENSMUST00000000128	ENSMUSG00000000126
mmu_circ_0001256	mmu-miR-486b-3	ENSMUST00000108783	ENSMUSG00000000126

mmu_circ_0001256	mmu-miR-486b-3	ENSMUST00000213072	ENSMUSG00000031849
mmu_circ_0001623	mmu-miR-486b-3	ENSMUST00000000128	ENSMUSG00000000126
mmu_circ_0001623	mmu-miR-486b-3	ENSMUST00000108783	ENSMUSG00000000126
mmu_circ_0001623	mmu-miR-486b-3	ENSMUST00000213072	ENSMUSG00000031849
mmu_circ_0001505	mmu-miR-486b-3	ENSMUST00000000128	ENSMUSG00000000126
mmu_circ_0001505	mmu-miR-486b-3	ENSMUST00000108783	ENSMUSG00000000126
mmu_circ_0001505	mmu-miR-486b-3	ENSMUST00000213072	ENSMUSG00000031849
mmu_circ_0001528	mmu-miR-486b-3	ENSMUST00000000128	ENSMUSG00000000126
mmu_circ_0001528	mmu-miR-486b-3	ENSMUST00000108783	ENSMUSG00000000126
mmu_circ_0001528	mmu-miR-486b-3	ENSMUST00000213072	ENSMUSG00000031849
mmu_circ_0001454	mmu-miR-486b-3	ENSMUST00000000128	ENSMUSG00000000126
mmu_circ_0001454	mmu-miR-486b-3	ENSMUST00000108783	ENSMUSG00000000126
mmu_circ_0001454	mmu-miR-486b-3	ENSMUST00000213072	ENSMUSG00000031849
mmu_circ_0001500	mmu-miR-486b-3	ENSMUST00000000128	ENSMUSG00000000126
mmu_circ_0001500	mmu-miR-486b-3	ENSMUST00000108783	ENSMUSG00000000126
mmu_circ_0001500	mmu-miR-486b-3	ENSMUST00000213072	ENSMUSG00000031849
mmu_circ_0001501	mmu-miR-486b-3	ENSMUST00000000128	ENSMUSG00000000126
mmu_circ_0001501	mmu-miR-486b-3	ENSMUST00000108783	ENSMUSG00000000126
mmu_circ_0001501	mmu-miR-486b-3	ENSMUST00000213072	ENSMUSG00000031849
mmu_circ_0001755	mmu-miR-486b-3	ENSMUST00000000128	ENSMUSG00000000126
mmu_circ_0001755	mmu-miR-486b-3	ENSMUST00000108783	ENSMUSG00000000126
mmu_circ_0001755	mmu-miR-486b-3	ENSMUST00000213072	ENSMUSG00000031849
mmu_circ_0001886	mmu-miR-486b-3	ENSMUST00000000128	ENSMUSG00000000126
mmu_circ_0001886	mmu-miR-486b-3	ENSMUST00000108783	ENSMUSG00000000126
mmu_circ_0001886	mmu-miR-486b-3	ENSMUST00000213072	ENSMUSG00000031849
mmu_circ_0000325	mmu-miR-486b-3	ENSMUST00000000128	ENSMUSG00000000126
mmu_circ_0000325	mmu-miR-486b-3	ENSMUST00000108783	ENSMUSG00000000126
mmu_circ_0000325	mmu-miR-486b-3	ENSMUST00000213072	ENSMUSG00000031849
mmu_circ_0000498	mmu-miR-486b-3	ENSMUST00000000128	ENSMUSG00000000126
mmu_circ_0000498	mmu-miR-486b-3	ENSMUST00000108783	ENSMUSG00000000126
mmu_circ_0000498	mmu-miR-486b-3	ENSMUST00000213072	ENSMUSG00000031849
mmu_circ_0000499	mmu-miR-486b-3	ENSMUST00000000128	ENSMUSG00000000126
mmu_circ_0000499	mmu-miR-486b-3	ENSMUST00000108783	ENSMUSG00000000126
mmu_circ_0000499	mmu-miR-486b-3	ENSMUST00000213072	ENSMUSG00000031849
mmu_circ_0000500	mmu-miR-486b-3	ENSMUST00000000128	ENSMUSG00000000126
mmu_circ_0000500	mmu-miR-486b-3	ENSMUST00000108783	ENSMUSG00000000126
mmu_circ_0000500	mmu-miR-486b-3	ENSMUST00000213072	ENSMUSG00000031849
mmu_circ_0000371	mmu-miR-486b-3	ENSMUST00000000128	ENSMUSG00000000126
mmu_circ_0000371	mmu-miR-486b-3	ENSMUST00000108783	ENSMUSG00000000126
mmu_circ_0000371	mmu-miR-486b-3	ENSMUST00000213072	ENSMUSG00000031849
mmu_circ_0000399	mmu-miR-486b-3	ENSMUST00000000128	ENSMUSG00000000126
mmu_circ_0000399	mmu-miR-486b-3	ENSMUST00000108783	ENSMUSG00000000126
mmu_circ_0000399	mmu-miR-486b-3	ENSMUST00000213072	ENSMUSG00000031849
mmu_circ_0000577	mmu-miR-486b-3	ENSMUST00000000128	ENSMUSG00000000126
mmu_circ_0000577	mmu-miR-486b-3	ENSMUST00000108783	ENSMUSG00000000126
mmu_circ_0000577	mmu-miR-486b-3	ENSMUST00000213072	ENSMUSG00000031849
mmu_circ_0000628	mmu-miR-486b-3	ENSMUST00000000128	ENSMUSG00000000126
mmu_circ_0000628	mmu-miR-486b-3	ENSMUST00000108783	ENSMUSG00000000126
mmu_circ_0000628	mmu-miR-486b-3	ENSMUST00000213072	ENSMUSG00000031849
mmu_circ_0000635	mmu-miR-486b-3	ENSMUST00000000128	ENSMUSG00000000126
mmu_circ_0000635	mmu-miR-486b-3	ENSMUST00000108783	ENSMUSG00000000126
mmu_circ_0000635	mmu-miR-486b-3	ENSMUST00000213072	ENSMUSG00000031849
mmu_circ_0000644	mmu-miR-486b-3	ENSMUST00000000128	ENSMUSG00000000126
mmu_circ_0000644	mmu-miR-486b-3	ENSMUST00000108783	ENSMUSG00000000126
mmu_circ_0000644	mmu-miR-486b-3	ENSMUST00000213072	ENSMUSG00000031849
mmu_circ_0000530	mmu-miR-486b-3	ENSMUST00000000128	ENSMUSG00000000126
mmu_circ_0000530	mmu-miR-486b-3	ENSMUST00000108783	ENSMUSG00000000126
mmu_circ_0000530	mmu-miR-486b-3	ENSMUST00000213072	ENSMUSG00000031849

mmu_circ_0000554	mmu-miR-486b-3	ENSMUST00000000128	ENSMUSG00000000126
mmu_circ_0000554	mmu-miR-486b-3	ENSMUST00000108783	ENSMUSG00000000126
mmu_circ_0000554	mmu-miR-486b-3	ENSMUST00000213072	ENSMUSG00000031849
mmu_circ_0000735	mmu-miR-486b-3	ENSMUST00000000128	ENSMUSG00000000126
mmu_circ_0000735	mmu-miR-486b-3	ENSMUST00000108783	ENSMUSG00000000126
mmu_circ_0000735	mmu-miR-486b-3	ENSMUST00000213072	ENSMUSG00000031849
mmu_circ_0000806	mmu-miR-486b-3	ENSMUST00000000128	ENSMUSG00000000126
mmu_circ_0000806	mmu-miR-486b-3	ENSMUST00000108783	ENSMUSG00000000126
mmu_circ_0000806	mmu-miR-486b-3	ENSMUST00000213072	ENSMUSG00000031849
mmu_circ_0000820	mmu-miR-486b-3	ENSMUST00000000128	ENSMUSG00000000126
mmu_circ_0000820	mmu-miR-486b-3	ENSMUST00000108783	ENSMUSG00000000126
mmu_circ_0000820	mmu-miR-486b-3	ENSMUST00000213072	ENSMUSG00000031849
mmu_circ_0000828	mmu-miR-486b-3	ENSMUST00000000128	ENSMUSG00000000126
mmu_circ_0000828	mmu-miR-486b-3	ENSMUST00000108783	ENSMUSG00000000126
mmu_circ_0000828	mmu-miR-486b-3	ENSMUST00000213072	ENSMUSG00000031849
mmu_circ_0000829	mmu-miR-486b-3	ENSMUST00000000128	ENSMUSG00000000126
mmu_circ_0000829	mmu-miR-486b-3	ENSMUST00000108783	ENSMUSG00000000126
mmu_circ_0000829	mmu-miR-486b-3	ENSMUST00000213072	ENSMUSG00000031849
mmu_circ_0000688	mmu-miR-486b-3	ENSMUST00000000128	ENSMUSG00000000126
mmu_circ_0000688	mmu-miR-486b-3	ENSMUST00000108783	ENSMUSG00000000126
mmu_circ_0000688	mmu-miR-486b-3	ENSMUST00000213072	ENSMUSG00000031849
mmu_circ_0000712	mmu-miR-486b-3	ENSMUST00000000128	ENSMUSG00000000126
mmu_circ_0000712	mmu-miR-486b-3	ENSMUST00000108783	ENSMUSG00000000126
mmu_circ_0000712	mmu-miR-486b-3	ENSMUST00000213072	ENSMUSG00000031849
mmu_circ_0000713	mmu-miR-486b-3	ENSMUST00000000128	ENSMUSG00000000126
mmu_circ_0000713	mmu-miR-486b-3	ENSMUST00000108783	ENSMUSG00000000126
mmu_circ_0000713	mmu-miR-486b-3	ENSMUST00000213072	ENSMUSG00000031849
mmu_circ_0000714	mmu-miR-486b-3	ENSMUST00000000128	ENSMUSG00000000126
mmu_circ_0000714	mmu-miR-486b-3	ENSMUST00000108783	ENSMUSG00000000126
mmu_circ_0000714	mmu-miR-486b-3	ENSMUST00000213072	ENSMUSG00000031849
mmu_circ_0000919	mmu-miR-486b-3	ENSMUST00000000128	ENSMUSG00000000126
mmu_circ_0000919	mmu-miR-486b-3	ENSMUST00000108783	ENSMUSG00000000126
mmu_circ_0000919	mmu-miR-486b-3	ENSMUST00000213072	ENSMUSG00000031849
mmu_circ_0000921	mmu-miR-486b-3	ENSMUST00000000128	ENSMUSG00000000126
mmu_circ_0000921	mmu-miR-486b-3	ENSMUST00000108783	ENSMUSG00000000126
mmu_circ_0000921	mmu-miR-486b-3	ENSMUST00000213072	ENSMUSG00000031849
mmu_circ_0000969	mmu-miR-486b-3	ENSMUST00000000128	ENSMUSG00000000126
mmu_circ_0000969	mmu-miR-486b-3	ENSMUST00000108783	ENSMUSG00000000126
mmu_circ_0000969	mmu-miR-486b-3	ENSMUST00000213072	ENSMUSG00000031849
mmu_circ_0000884	mmu-miR-486b-3	ENSMUST00000000128	ENSMUSG00000000126
mmu_circ_0000884	mmu-miR-486b-3	ENSMUST00000108783	ENSMUSG00000000126
mmu_circ_0000884	mmu-miR-486b-3	ENSMUST00000213072	ENSMUSG00000031849
mmu_circ_0000886	mmu-miR-486b-3	ENSMUST00000000128	ENSMUSG00000000126
mmu_circ_0000886	mmu-miR-486b-3	ENSMUST00000108783	ENSMUSG00000000126
mmu_circ_0000886	mmu-miR-486b-3	ENSMUST00000213072	ENSMUSG00000031849
mmu_circ_0000887	mmu-miR-486b-3	ENSMUST00000000128	ENSMUSG00000000126
mmu_circ_0000887	mmu-miR-486b-3	ENSMUST00000108783	ENSMUSG00000000126
mmu_circ_0000887	mmu-miR-486b-3	ENSMUST00000213072	ENSMUSG00000031849
mmu_circ_0000905	mmu-miR-486b-3	ENSMUST00000000128	ENSMUSG00000000126
mmu_circ_0000905	mmu-miR-486b-3	ENSMUST00000108783	ENSMUSG00000000126
mmu_circ_0000905	mmu-miR-486b-3	ENSMUST00000213072	ENSMUSG00000031849
mmu_circ_0000906	mmu-miR-486b-3	ENSMUST00000000128	ENSMUSG00000000126
mmu_circ_0000906	mmu-miR-486b-3	ENSMUST00000108783	ENSMUSG00000000126
mmu_circ_0000906	mmu-miR-486b-3	ENSMUST00000213072	ENSMUSG00000031849
mmu_circ_0000907	mmu-miR-486b-3	ENSMUST00000000128	ENSMUSG00000000126
mmu_circ_0000907	mmu-miR-486b-3	ENSMUST00000108783	ENSMUSG00000000126
mmu_circ_0000907	mmu-miR-486b-3	ENSMUST00000213072	ENSMUSG00000031849
mmu_circ_0000908	mmu-miR-486b-3	ENSMUST00000000128	ENSMUSG00000000126

mmu_circ_0000908	mmu-miR-486b-3	ENSMUST00000108783	ENSMUSG00000000126
mmu_circ_0000908	mmu-miR-486b-3	ENSMUST00000213072	ENSMUSG00000031849
mmu_circ_0000912	mmu-miR-486b-3	ENSMUST00000000128	ENSMUSG00000000126
mmu_circ_0000912	mmu-miR-486b-3	ENSMUST00000108783	ENSMUSG00000000126
mmu_circ_0000912	mmu-miR-486b-3	ENSMUST00000213072	ENSMUSG00000031849
novel_circ_0001676	mmu-miR-486b-3	ENSMUST00000000128	ENSMUSG00000000126
novel_circ_0001676	mmu-miR-486b-3	ENSMUST00000108783	ENSMUSG00000000126
novel_circ_0001676	mmu-miR-486b-3	ENSMUST00000213072	ENSMUSG00000031849
novel_circ_0001726	mmu-miR-486b-3	ENSMUST00000000128	ENSMUSG00000000126
novel_circ_0001726	mmu-miR-486b-3	ENSMUST00000108783	ENSMUSG00000000126
novel_circ_0001726	mmu-miR-486b-3	ENSMUST00000213072	ENSMUSG00000031849
novel_circ_0002100	mmu-miR-486b-3	ENSMUST00000000128	ENSMUSG00000000126
novel_circ_0002100	mmu-miR-486b-3	ENSMUST00000108783	ENSMUSG00000000126
novel_circ_0002100	mmu-miR-486b-3	ENSMUST00000213072	ENSMUSG00000031849
novel_circ_0002180	mmu-miR-486b-3	ENSMUST00000000128	ENSMUSG00000000126
novel_circ_0002180	mmu-miR-486b-3	ENSMUST00000108783	ENSMUSG00000000126
novel_circ_0002180	mmu-miR-486b-3	ENSMUST00000213072	ENSMUSG00000031849
novel_circ_0002217	mmu-miR-486b-3	ENSMUST00000000128	ENSMUSG00000000126
novel_circ_0002217	mmu-miR-486b-3	ENSMUST00000108783	ENSMUSG00000000126
novel_circ_0002217	mmu-miR-486b-3	ENSMUST00000213072	ENSMUSG00000031849
novel_circ_0001751	mmu-miR-486b-3	ENSMUST00000000128	ENSMUSG00000000126
novel_circ_0001751	mmu-miR-486b-3	ENSMUST00000108783	ENSMUSG00000000126
novel_circ_0001751	mmu-miR-486b-3	ENSMUST00000213072	ENSMUSG00000031849
novel_circ_0001795	mmu-miR-486b-3	ENSMUST00000000128	ENSMUSG00000000126
novel_circ_0001795	mmu-miR-486b-3	ENSMUST00000108783	ENSMUSG00000000126
novel_circ_0001795	mmu-miR-486b-3	ENSMUST00000213072	ENSMUSG00000031849
novel_circ_0002027	mmu-miR-486b-3	ENSMUST00000000128	ENSMUSG00000000126
novel_circ_0002027	mmu-miR-486b-3	ENSMUST00000108783	ENSMUSG00000000126
novel_circ_0002027	mmu-miR-486b-3	ENSMUST00000213072	ENSMUSG00000031849
novel_circ_0002027	mmu-miR-486b-3	ENSMUST00000000128	ENSMUSG00000000126
novel_circ_0002027	mmu-miR-486b-3	ENSMUST00000108783	ENSMUSG00000000126
novel_circ_0002027	mmu-miR-486b-3	ENSMUST00000213072	ENSMUSG00000031849
novel_circ_0002447	mmu-miR-486b-3	ENSMUST00000000128	ENSMUSG00000000126
novel_circ_0002447	mmu-miR-486b-3	ENSMUST00000108783	ENSMUSG00000000126
novel_circ_0002447	mmu-miR-486b-3	ENSMUST00000213072	ENSMUSG00000031849
novel_circ_0002448	mmu-miR-486b-3	ENSMUST00000000128	ENSMUSG00000000126
novel_circ_0002448	mmu-miR-486b-3	ENSMUST00000108783	ENSMUSG00000000126
novel_circ_0002448	mmu-miR-486b-3	ENSMUST00000213072	ENSMUSG00000031849
novel_circ_0002484	mmu-miR-486b-3	ENSMUST00000000128	ENSMUSG00000000126
novel_circ_0002484	mmu-miR-486b-3	ENSMUST00000108783	ENSMUSG00000000126
novel_circ_0002484	mmu-miR-486b-3	ENSMUST00000213072	ENSMUSG00000031849
novel_circ_0002485	mmu-miR-486b-3	ENSMUST00000000128	ENSMUSG00000000126
novel_circ_0002485	mmu-miR-486b-3	ENSMUST00000108783	ENSMUSG00000000126
novel_circ_0002485	mmu-miR-486b-3	ENSMUST00000213072	ENSMUSG00000031849
novel_circ_0002494	mmu-miR-486b-3	ENSMUST00000000128	ENSMUSG00000000126
novel_circ_0002494	mmu-miR-486b-3	ENSMUST00000108783	ENSMUSG00000000126
novel_circ_0002494	mmu-miR-486b-3	ENSMUST00000213072	ENSMUSG00000031849
novel_circ_0002497	mmu-miR-486b-3	ENSMUST00000000128	ENSMUSG00000000126
novel_circ_0002497	mmu-miR-486b-3	ENSMUST00000108783	ENSMUSG00000000126
novel_circ_0002497	mmu-miR-486b-3	ENSMUST00000213072	ENSMUSG00000031849
novel_circ_0002597	mmu-miR-486b-3	ENSMUST00000000128	ENSMUSG00000000126
novel_circ_0002597	mmu-miR-486b-3	ENSMUST00000108783	ENSMUSG00000000126
novel_circ_0002597	mmu-miR-486b-3	ENSMUST00000213072	ENSMUSG00000031849
novel_circ_0002241	mmu-miR-486b-3	ENSMUST00000000128	ENSMUSG00000000126
novel_circ_0002241	mmu-miR-486b-3	ENSMUST00000108783	ENSMUSG00000000126
novel_circ_0002241	mmu-miR-486b-3	ENSMUST00000213072	ENSMUSG00000031849
novel_circ_0002257	mmu-miR-486b-3	ENSMUST00000000128	ENSMUSG00000000126
novel_circ_0002257	mmu-miR-486b-3	ENSMUST00000108783	ENSMUSG00000000126

novel_circ_0002257	mmu-miR-486b-3	ENSMUST00000213072	ENSMUSG00000031849
novel_circ_0002299	mmu-miR-486b-3	ENSMUST00000000128	ENSMUSG00000000126
novel_circ_0002299	mmu-miR-486b-3	ENSMUST00000108783	ENSMUSG00000000126
novel_circ_0002299	mmu-miR-486b-3	ENSMUST00000213072	ENSMUSG00000031849
novel_circ_0002336	mmu-miR-486b-3	ENSMUST00000000128	ENSMUSG00000000126
novel_circ_0002336	mmu-miR-486b-3	ENSMUST00000108783	ENSMUSG00000000126
novel_circ_0002336	mmu-miR-486b-3	ENSMUST00000213072	ENSMUSG00000031849
novel_circ_0002336	mmu-miR-486b-3	ENSMUST00000000128	ENSMUSG00000000126
novel_circ_0002336	mmu-miR-486b-3	ENSMUST00000108783	ENSMUSG00000000126
novel_circ_0002336	mmu-miR-486b-3	ENSMUST00000213072	ENSMUSG00000031849
novel_circ_0002343	mmu-miR-486b-3	ENSMUST00000000128	ENSMUSG00000000126
novel_circ_0002343	mmu-miR-486b-3	ENSMUST00000108783	ENSMUSG00000000126
novel_circ_0002343	mmu-miR-486b-3	ENSMUST00000213072	ENSMUSG00000031849
novel_circ_0002914	mmu-miR-486b-3	ENSMUST00000000128	ENSMUSG00000000126
novel_circ_0002914	mmu-miR-486b-3	ENSMUST00000108783	ENSMUSG00000000126
novel_circ_0002914	mmu-miR-486b-3	ENSMUST00000213072	ENSMUSG00000031849
novel_circ_0002917	mmu-miR-486b-3	ENSMUST00000000128	ENSMUSG00000000126
novel_circ_0002917	mmu-miR-486b-3	ENSMUST00000108783	ENSMUSG00000000126
novel_circ_0002917	mmu-miR-486b-3	ENSMUST00000213072	ENSMUSG00000031849
novel_circ_0002943	mmu-miR-486b-3	ENSMUST00000000128	ENSMUSG00000000126
novel_circ_0002943	mmu-miR-486b-3	ENSMUST00000108783	ENSMUSG00000000126
novel_circ_0002943	mmu-miR-486b-3	ENSMUST00000213072	ENSMUSG00000031849
novel_circ_0002943	mmu-miR-486b-3	ENSMUST00000000128	ENSMUSG00000000126
novel_circ_0002943	mmu-miR-486b-3	ENSMUST00000108783	ENSMUSG00000000126
novel_circ_0002943	mmu-miR-486b-3	ENSMUST00000213072	ENSMUSG00000031849
novel_circ_0002648	mmu-miR-486b-3	ENSMUST00000000128	ENSMUSG00000000126
novel_circ_0002648	mmu-miR-486b-3	ENSMUST00000108783	ENSMUSG00000000126
novel_circ_0002648	mmu-miR-486b-3	ENSMUST00000213072	ENSMUSG00000031849
novel_circ_0002722	mmu-miR-486b-3	ENSMUST00000000128	ENSMUSG00000000126
novel_circ_0002722	mmu-miR-486b-3	ENSMUST00000108783	ENSMUSG00000000126
novel_circ_0002722	mmu-miR-486b-3	ENSMUST00000213072	ENSMUSG00000031849
novel_circ_0002788	mmu-miR-486b-3	ENSMUST00000000128	ENSMUSG00000000126
novel_circ_0002788	mmu-miR-486b-3	ENSMUST00000108783	ENSMUSG00000000126
novel_circ_0002788	mmu-miR-486b-3	ENSMUST00000213072	ENSMUSG00000031849
novel_circ_0003213	mmu-miR-486b-3	ENSMUST00000000128	ENSMUSG00000000126
novel_circ_0003213	mmu-miR-486b-3	ENSMUST00000108783	ENSMUSG00000000126
novel_circ_0003213	mmu-miR-486b-3	ENSMUST00000213072	ENSMUSG00000031849
novel_circ_0003262	mmu-miR-486b-3	ENSMUST00000000128	ENSMUSG00000000126
novel_circ_0003262	mmu-miR-486b-3	ENSMUST00000108783	ENSMUSG00000000126
novel_circ_0003262	mmu-miR-486b-3	ENSMUST00000213072	ENSMUSG00000031849
novel_circ_0003262	mmu-miR-486b-3	ENSMUST00000000128	ENSMUSG00000000126
novel_circ_0003262	mmu-miR-486b-3	ENSMUST00000108783	ENSMUSG00000000126
novel_circ_0003262	mmu-miR-486b-3	ENSMUST00000213072	ENSMUSG00000031849
novel_circ_0003309	mmu-miR-486b-3	ENSMUST00000000128	ENSMUSG00000000126
novel_circ_0003309	mmu-miR-486b-3	ENSMUST00000108783	ENSMUSG00000000126
novel_circ_0003309	mmu-miR-486b-3	ENSMUST00000213072	ENSMUSG00000031849
novel_circ_0003053	mmu-miR-486b-3	ENSMUST00000000128	ENSMUSG00000000126
novel_circ_0003053	mmu-miR-486b-3	ENSMUST00000108783	ENSMUSG00000000126
novel_circ_0003053	mmu-miR-486b-3	ENSMUST00000213072	ENSMUSG00000031849
novel_circ_0003462	mmu-miR-486b-3	ENSMUST00000000128	ENSMUSG00000000126
novel_circ_0003462	mmu-miR-486b-3	ENSMUST00000108783	ENSMUSG00000000126
novel_circ_0003462	mmu-miR-486b-3	ENSMUST00000213072	ENSMUSG00000031849
novel_circ_0000263	mmu-miR-486b-3	ENSMUST00000000128	ENSMUSG00000000126
novel_circ_0000263	mmu-miR-486b-3	ENSMUST00000108783	ENSMUSG00000000126
novel_circ_0000263	mmu-miR-486b-3	ENSMUST00000213072	ENSMUSG00000031849
novel_circ_0000287	mmu-miR-486b-3	ENSMUST00000000128	ENSMUSG00000000126
novel_circ_0000287	mmu-miR-486b-3	ENSMUST00000108783	ENSMUSG00000000126
novel_circ_0000287	mmu-miR-486b-3	ENSMUST00000213072	ENSMUSG00000031849

novel_circ_0000291	mmu-miR-486b-3	ENSMUST00000000128	ENSMUSG00000000126
novel_circ_0000291	mmu-miR-486b-3	ENSMUST00000108783	ENSMUSG00000000126
novel_circ_0000291	mmu-miR-486b-3	ENSMUST00000213072	ENSMUSG00000031849
novel_circ_0000291	mmu-miR-486b-3	ENSMUST00000000128	ENSMUSG00000000126
novel_circ_0000291	mmu-miR-486b-3	ENSMUST00000108783	ENSMUSG00000000126
novel_circ_0000291	mmu-miR-486b-3	ENSMUST00000213072	ENSMUSG00000031849
novel_circ_0000292	mmu-miR-486b-3	ENSMUST00000000128	ENSMUSG00000000126
novel_circ_0000292	mmu-miR-486b-3	ENSMUST00000108783	ENSMUSG00000000126
novel_circ_0000292	mmu-miR-486b-3	ENSMUST00000213072	ENSMUSG00000031849
novel_circ_0000292	mmu-miR-486b-3	ENSMUST00000000128	ENSMUSG00000000126
novel_circ_0000292	mmu-miR-486b-3	ENSMUST00000108783	ENSMUSG00000000126
novel_circ_0000292	mmu-miR-486b-3	ENSMUST00000213072	ENSMUSG00000031849
novel_circ_0000400	mmu-miR-486b-3	ENSMUST00000000128	ENSMUSG00000000126
novel_circ_0000400	mmu-miR-486b-3	ENSMUST00000108783	ENSMUSG00000000126
novel_circ_0000400	mmu-miR-486b-3	ENSMUST00000213072	ENSMUSG00000031849
novel_circ_0000022	mmu-miR-486b-3	ENSMUST00000000128	ENSMUSG00000000126
novel_circ_0000022	mmu-miR-486b-3	ENSMUST00000108783	ENSMUSG00000000126
novel_circ_0000022	mmu-miR-486b-3	ENSMUST00000213072	ENSMUSG00000031849
novel_circ_0000068	mmu-miR-486b-3	ENSMUST00000000128	ENSMUSG00000000126
novel_circ_0000068	mmu-miR-486b-3	ENSMUST00000108783	ENSMUSG00000000126
novel_circ_0000068	mmu-miR-486b-3	ENSMUST00000213072	ENSMUSG00000031849
novel_circ_0000129	mmu-miR-486b-3	ENSMUST00000000128	ENSMUSG00000000126
novel_circ_0000129	mmu-miR-486b-3	ENSMUST00000108783	ENSMUSG00000000126
novel_circ_0000129	mmu-miR-486b-3	ENSMUST00000213072	ENSMUSG00000031849
novel_circ_0000131	mmu-miR-486b-3	ENSMUST00000000128	ENSMUSG00000000126
novel_circ_0000131	mmu-miR-486b-3	ENSMUST00000108783	ENSMUSG00000000126
novel_circ_0000131	mmu-miR-486b-3	ENSMUST00000213072	ENSMUSG00000031849
novel_circ_0000132	mmu-miR-486b-3	ENSMUST00000000128	ENSMUSG00000000126
novel_circ_0000132	mmu-miR-486b-3	ENSMUST00000108783	ENSMUSG00000000126
novel_circ_0000132	mmu-miR-486b-3	ENSMUST00000213072	ENSMUSG00000031849
novel_circ_0000687	mmu-miR-486b-3	ENSMUST00000000128	ENSMUSG00000000126
novel_circ_0000687	mmu-miR-486b-3	ENSMUST00000108783	ENSMUSG00000000126
novel_circ_0000687	mmu-miR-486b-3	ENSMUST00000213072	ENSMUSG00000031849
novel_circ_0000692	mmu-miR-486b-3	ENSMUST00000000128	ENSMUSG00000000126
novel_circ_0000692	mmu-miR-486b-3	ENSMUST00000108783	ENSMUSG00000000126
novel_circ_0000692	mmu-miR-486b-3	ENSMUST00000213072	ENSMUSG00000031849
novel_circ_0000696	mmu-miR-486b-3	ENSMUST00000000128	ENSMUSG00000000126
novel_circ_0000696	mmu-miR-486b-3	ENSMUST00000108783	ENSMUSG00000000126
novel_circ_0000696	mmu-miR-486b-3	ENSMUST00000213072	ENSMUSG00000031849
novel_circ_0000698	mmu-miR-486b-3	ENSMUST00000000128	ENSMUSG00000000126
novel_circ_0000698	mmu-miR-486b-3	ENSMUST00000108783	ENSMUSG00000000126
novel_circ_0000698	mmu-miR-486b-3	ENSMUST00000213072	ENSMUSG00000031849
novel_circ_0000772	mmu-miR-486b-3	ENSMUST00000000128	ENSMUSG00000000126
novel_circ_0000772	mmu-miR-486b-3	ENSMUST00000108783	ENSMUSG00000000126
novel_circ_0000772	mmu-miR-486b-3	ENSMUST00000213072	ENSMUSG00000031849
novel_circ_0000589	mmu-miR-486b-3	ENSMUST00000000128	ENSMUSG00000000126
novel_circ_0000589	mmu-miR-486b-3	ENSMUST00000108783	ENSMUSG00000000126
novel_circ_0000589	mmu-miR-486b-3	ENSMUST00000213072	ENSMUSG00000031849
novel_circ_0000594	mmu-miR-486b-3	ENSMUST00000000128	ENSMUSG00000000126
novel_circ_0000594	mmu-miR-486b-3	ENSMUST00000108783	ENSMUSG00000000126
novel_circ_0000594	mmu-miR-486b-3	ENSMUST00000213072	ENSMUSG00000031849
novel_circ_0001046	mmu-miR-486b-3	ENSMUST00000000128	ENSMUSG00000000126
novel_circ_0001046	mmu-miR-486b-3	ENSMUST00000108783	ENSMUSG00000000126
novel_circ_0001046	mmu-miR-486b-3	ENSMUST00000213072	ENSMUSG00000031849
novel_circ_0000900	mmu-miR-486b-3	ENSMUST00000000128	ENSMUSG00000000126
novel_circ_0000900	mmu-miR-486b-3	ENSMUST00000108783	ENSMUSG00000000126
novel_circ_0000900	mmu-miR-486b-3	ENSMUST00000213072	ENSMUSG00000031849
novel_circ_0000901	mmu-miR-486b-3	ENSMUST00000000128	ENSMUSG00000000126

novel_circ_0000901	mmu-miR-486b-3	ENSMUST00000108783	ENSMUSG00000000126
novel_circ_0000901	mmu-miR-486b-3	ENSMUST00000213072	ENSMUSG00000031849
novel_circ_0001177	mmu-miR-486b-3	ENSMUST00000000128	ENSMUSG00000000126
novel_circ_0001177	mmu-miR-486b-3	ENSMUST00000108783	ENSMUSG00000000126
novel_circ_0001177	mmu-miR-486b-3	ENSMUST00000213072	ENSMUSG00000031849
novel_circ_0001179	mmu-miR-486b-3	ENSMUST00000000128	ENSMUSG00000000126
novel_circ_0001179	mmu-miR-486b-3	ENSMUST00000108783	ENSMUSG00000000126
novel_circ_0001179	mmu-miR-486b-3	ENSMUST00000213072	ENSMUSG00000031849
novel_circ_0001206	mmu-miR-486b-3	ENSMUST00000000128	ENSMUSG00000000126
novel_circ_0001206	mmu-miR-486b-3	ENSMUST00000108783	ENSMUSG00000000126
novel_circ_0001206	mmu-miR-486b-3	ENSMUST00000213072	ENSMUSG00000031849
novel_circ_0001304	mmu-miR-5107-5	ENSMUST00000213072	ENSMUSG00000031849
novel_circ_0001076	mmu-miR-5107-5	ENSMUST00000213072	ENSMUSG00000031849
novel_circ_0001136	mmu-miR-5107-5	ENSMUST00000213072	ENSMUSG00000031849
novel_circ_0001508	mmu-miR-5107-5	ENSMUST00000213072	ENSMUSG00000031849
novel_circ_0001349	mmu-miR-5107-5	ENSMUST00000213072	ENSMUSG00000031849
novel_circ_0001381	mmu-miR-5107-5	ENSMUST00000213072	ENSMUSG00000031849
novel_circ_0001385	mmu-miR-5107-5	ENSMUST00000213072	ENSMUSG00000031849
novel_circ_0001388	mmu-miR-5107-5	ENSMUST00000213072	ENSMUSG00000031849
novel_circ_0001433	mmu-miR-5107-5	ENSMUST00000213072	ENSMUSG00000031849
mmu_circ_0000075	mmu-miR-5107-5	ENSMUST00000213072	ENSMUSG00000031849
mmu_circ_0000048	mmu-miR-5107-5	ENSMUST00000213072	ENSMUSG00000031849
mmu_circ_0000049	mmu-miR-5107-5	ENSMUST00000213072	ENSMUSG00000031849
mmu_circ_0000061	mmu-miR-5107-5	ENSMUST00000213072	ENSMUSG00000031849
mmu_circ_0000063	mmu-miR-5107-5	ENSMUST00000213072	ENSMUSG00000031849
mmu_circ_0001103	mmu-miR-5107-5	ENSMUST00000213072	ENSMUSG00000031849
mmu_circ_0001376	mmu-miR-5107-5	ENSMUST00000213072	ENSMUSG00000031849
mmu_circ_0001427	mmu-miR-5107-5	ENSMUST00000213072	ENSMUSG00000031849
mmu_circ_0001427	mmu-miR-5107-5	ENSMUST00000213072	ENSMUSG00000031849
mmu_circ_0001331	mmu-miR-5107-5	ENSMUST00000213072	ENSMUSG00000031849
mmu_circ_0001333	mmu-miR-5107-5	ENSMUST00000213072	ENSMUSG00000031849
mmu_circ_0001336	mmu-miR-5107-5	ENSMUST00000213072	ENSMUSG00000031849
mmu_circ_0001447	mmu-miR-5107-5	ENSMUST00000213072	ENSMUSG00000031849
mmu_circ_0001454	mmu-miR-5107-5	ENSMUST00000213072	ENSMUSG00000031849
mmu_circ_0001465	mmu-miR-5107-5	ENSMUST00000213072	ENSMUSG00000031849
mmu_circ_0001482	mmu-miR-5107-5	ENSMUST00000213072	ENSMUSG00000031849
mmu_circ_0001500	mmu-miR-5107-5	ENSMUST00000213072	ENSMUSG00000031849
mmu_circ_0001842	mmu-miR-5107-5	ENSMUST00000213072	ENSMUSG00000031849
mmu_circ_0001821	mmu-miR-5107-5	ENSMUST00000213072	ENSMUSG00000031849
mmu_circ_0001665	mmu-miR-5107-5	ENSMUST00000213072	ENSMUSG00000031849
mmu_circ_0001705	mmu-miR-5107-5	ENSMUST00000213072	ENSMUSG00000031849
mmu_circ_0001718	mmu-miR-5107-5	ENSMUST00000213072	ENSMUSG00000031849
mmu_circ_0000499	mmu-miR-5107-5	ENSMUST00000213072	ENSMUSG00000031849
mmu_circ_0000500	mmu-miR-5107-5	ENSMUST00000213072	ENSMUSG00000031849
mmu_circ_0000404	mmu-miR-5107-5	ENSMUST00000213072	ENSMUSG00000031849
mmu_circ_0000405	mmu-miR-5107-5	ENSMUST00000213072	ENSMUSG00000031849
mmu_circ_0000628	mmu-miR-5107-5	ENSMUST00000213072	ENSMUSG00000031849
mmu_circ_0000530	mmu-miR-5107-5	ENSMUST00000213072	ENSMUSG00000031849
mmu_circ_0000547	mmu-miR-5107-5	ENSMUST00000213072	ENSMUSG00000031849
mmu_circ_0000554	mmu-miR-5107-5	ENSMUST00000213072	ENSMUSG00000031849
mmu_circ_0000828	mmu-miR-5107-5	ENSMUST00000213072	ENSMUSG00000031849
mmu_circ_0000829	mmu-miR-5107-5	ENSMUST00000213072	ENSMUSG00000031849
mmu_circ_0000663	mmu-miR-5107-5	ENSMUST00000213072	ENSMUSG00000031849
mmu_circ_0000712	mmu-miR-5107-5	ENSMUST00000213072	ENSMUSG00000031849
mmu_circ_0000713	mmu-miR-5107-5	ENSMUST00000213072	ENSMUSG00000031849
mmu_circ_0000714	mmu-miR-5107-5	ENSMUST00000213072	ENSMUSG00000031849
mmu_circ_0000919	mmu-miR-5107-5	ENSMUST00000213072	ENSMUSG00000031849
mmu_circ_0000921	mmu-miR-5107-5	ENSMUST00000213072	ENSMUSG00000031849

mmu_circ_0000969	mmu-miR-5107-5	ENSMUST00000213072	ENSMUSG00000031849
mmu_circ_0000884	mmu-miR-5107-5	ENSMUST00000213072	ENSMUSG00000031849
mmu_circ_0000886	mmu-miR-5107-5	ENSMUST00000213072	ENSMUSG00000031849
mmu_circ_0000903	mmu-miR-5107-5	ENSMUST00000213072	ENSMUSG00000031849
mmu_circ_0000905	mmu-miR-5107-5	ENSMUST00000213072	ENSMUSG00000031849
mmu_circ_0000905	mmu-miR-5107-5	ENSMUST00000213072	ENSMUSG00000031849
mmu_circ_0000906	mmu-miR-5107-5	ENSMUST00000213072	ENSMUSG00000031849
mmu_circ_0000907	mmu-miR-5107-5	ENSMUST00000213072	ENSMUSG00000031849
mmu_circ_0000908	mmu-miR-5107-5	ENSMUST00000213072	ENSMUSG00000031849
novel_circ_0001551	mmu-miR-5107-5	ENSMUST00000213072	ENSMUSG00000031849
novel_circ_0002110	mmu-miR-5107-5	ENSMUST00000213072	ENSMUSG00000031849
novel_circ_0002180	mmu-miR-5107-5	ENSMUST00000213072	ENSMUSG00000031849
novel_circ_0002209	mmu-miR-5107-5	ENSMUST00000213072	ENSMUSG00000031849
novel_circ_0001795	mmu-miR-5107-5	ENSMUST00000213072	ENSMUSG00000031849
novel_circ_0001842	mmu-miR-5107-5	ENSMUST00000213072	ENSMUSG00000031849
novel_circ_0001905	mmu-miR-5107-5	ENSMUST00000213072	ENSMUSG00000031849
novel_circ_0001946	mmu-miR-5107-5	ENSMUST00000213072	ENSMUSG00000031849
novel_circ_0002499	mmu-miR-5107-5	ENSMUST00000213072	ENSMUSG00000031849
novel_circ_0002519	mmu-miR-5107-5	ENSMUST00000213072	ENSMUSG00000031849
novel_circ_0002597	mmu-miR-5107-5	ENSMUST00000213072	ENSMUSG00000031849
novel_circ_0002241	mmu-miR-5107-5	ENSMUST00000213072	ENSMUSG00000031849
novel_circ_0002243	mmu-miR-5107-5	ENSMUST00000213072	ENSMUSG00000031849
novel_circ_0002252	mmu-miR-5107-5	ENSMUST00000213072	ENSMUSG00000031849
novel_circ_0002278	mmu-miR-5107-5	ENSMUST00000213072	ENSMUSG00000031849
novel_circ_0002282	mmu-miR-5107-5	ENSMUST00000213072	ENSMUSG00000031849
novel_circ_0002282	mmu-miR-5107-5	ENSMUST00000213072	ENSMUSG00000031849
novel_circ_0002284	mmu-miR-5107-5	ENSMUST00000213072	ENSMUSG00000031849
novel_circ_0002299	mmu-miR-5107-5	ENSMUST00000213072	ENSMUSG00000031849
novel_circ_0002314	mmu-miR-5107-5	ENSMUST00000213072	ENSMUSG00000031849
novel_circ_0002336	mmu-miR-5107-5	ENSMUST00000213072	ENSMUSG00000031849
novel_circ_0002336	mmu-miR-5107-5	ENSMUST00000213072	ENSMUSG00000031849
novel_circ_0002348	mmu-miR-5107-5	ENSMUST00000213072	ENSMUSG00000031849
novel_circ_0002831	mmu-miR-5107-5	ENSMUST00000213072	ENSMUSG00000031849
novel_circ_0002925	mmu-miR-5107-5	ENSMUST00000213072	ENSMUSG00000031849
novel_circ_0002930	mmu-miR-5107-5	ENSMUST00000213072	ENSMUSG00000031849
novel_circ_0002648	mmu-miR-5107-5	ENSMUST00000213072	ENSMUSG00000031849
novel_circ_0002690	mmu-miR-5107-5	ENSMUST00000213072	ENSMUSG00000031849
novel_circ_0002775	mmu-miR-5107-5	ENSMUST00000213072	ENSMUSG00000031849
novel_circ_0002778	mmu-miR-5107-5	ENSMUST00000213072	ENSMUSG00000031849
novel_circ_0002780	mmu-miR-5107-5	ENSMUST00000213072	ENSMUSG00000031849
novel_circ_0002788	mmu-miR-5107-5	ENSMUST00000213072	ENSMUSG00000031849
novel_circ_0003174	mmu-miR-5107-5	ENSMUST00000213072	ENSMUSG00000031849
novel_circ_0003174	mmu-miR-5107-5	ENSMUST00000213072	ENSMUSG00000031849
novel_circ_0003207	mmu-miR-5107-5	ENSMUST00000213072	ENSMUSG00000031849
novel_circ_0003279	mmu-miR-5107-5	ENSMUST00000213072	ENSMUSG00000031849
novel_circ_0003279	mmu-miR-5107-5	ENSMUST00000213072	ENSMUSG00000031849
novel_circ_0003047	mmu-miR-5107-5	ENSMUST00000213072	ENSMUSG00000031849
novel_circ_0003083	mmu-miR-5107-5	ENSMUST00000213072	ENSMUSG00000031849
novel_circ_0000287	mmu-miR-5107-5	ENSMUST00000213072	ENSMUSG00000031849
novel_circ_0000444	mmu-miR-5107-5	ENSMUST00000213072	ENSMUSG00000031849
novel_circ_0000447	mmu-miR-5107-5	ENSMUST00000213072	ENSMUSG00000031849
novel_circ_0000129	mmu-miR-5107-5	ENSMUST00000213072	ENSMUSG00000031849
novel_circ_0000141	mmu-miR-5107-5	ENSMUST00000213072	ENSMUSG00000031849
novel_circ_0000143	mmu-miR-5107-5	ENSMUST00000213072	ENSMUSG00000031849
novel_circ_0000143	mmu-miR-5107-5	ENSMUST00000213072	ENSMUSG00000031849
novel_circ_0000707	mmu-miR-5107-5	ENSMUST00000213072	ENSMUSG00000031849
novel_circ_0000712	mmu-miR-5107-5	ENSMUST00000213072	ENSMUSG00000031849
novel_circ_0000484	mmu-miR-5107-5	ENSMUST00000213072	ENSMUSG00000031849

novel_circ_0000589	mmu-miR-5107-5	ENSMUST00000213072	ENSMUSG00000031849
novel_circ_0001025	mmu-miR-5107-5	ENSMUST00000213072	ENSMUSG00000031849
novel_circ_0001232	mmu-miR-5113	ENSMUST00000223576	ENSMUSG00000017491
novel_circ_0001278	mmu-miR-5113	ENSMUST00000223576	ENSMUSG00000017491
novel_circ_0001280	mmu-miR-5113	ENSMUST00000223576	ENSMUSG00000017491
novel_circ_0001300	mmu-miR-5113	ENSMUST00000223576	ENSMUSG00000017491
novel_circ_0001142	mmu-miR-5113	ENSMUST00000223576	ENSMUSG00000017491
novel_circ_0001143	mmu-miR-5113	ENSMUST00000223576	ENSMUSG00000017491
novel_circ_0001486	mmu-miR-5113	ENSMUST00000223576	ENSMUSG00000017491
novel_circ_0001543	mmu-miR-5113	ENSMUST00000223576	ENSMUSG00000017491
mmu_circ_0000061	mmu-miR-5113	ENSMUST00000223576	ENSMUSG00000017491
mmu_circ_0000063	mmu-miR-5113	ENSMUST00000223576	ENSMUSG00000017491
mmu_circ_0001154	mmu-miR-5113	ENSMUST00000223576	ENSMUSG00000017491
mmu_circ_0001162	mmu-miR-5113	ENSMUST00000223576	ENSMUSG00000017491
mmu_circ_0001170	mmu-miR-5113	ENSMUST00000223576	ENSMUSG00000017491
mmu_circ_0001123	mmu-miR-5113	ENSMUST00000223576	ENSMUSG00000017491
mmu_circ_0001143	mmu-miR-5113	ENSMUST00000223576	ENSMUSG00000017491
mmu_circ_0001103	mmu-miR-5113	ENSMUST00000223576	ENSMUSG00000017491
mmu_circ_0001021	mmu-miR-5113	ENSMUST00000223576	ENSMUSG00000017491
mmu_circ_0001031	mmu-miR-5113	ENSMUST00000223576	ENSMUSG00000017491
mmu_circ_0001376	mmu-miR-5113	ENSMUST00000223576	ENSMUSG00000017491
mmu_circ_0001387	mmu-miR-5113	ENSMUST00000223576	ENSMUSG00000017491
mmu_circ_0001410	mmu-miR-5113	ENSMUST00000223576	ENSMUSG00000017491
mmu_circ_0001279	mmu-miR-5113	ENSMUST00000223576	ENSMUSG00000017491
mmu_circ_0001598	mmu-miR-5113	ENSMUST00000223576	ENSMUSG00000017491
mmu_circ_0001528	mmu-miR-5113	ENSMUST00000223576	ENSMUSG00000017491
mmu_circ_0001447	mmu-miR-5113	ENSMUST00000223576	ENSMUSG00000017491
mmu_circ_0001454	mmu-miR-5113	ENSMUST00000223576	ENSMUSG00000017491
mmu_circ_0001470	mmu-miR-5113	ENSMUST00000223576	ENSMUSG00000017491
mmu_circ_0001473	mmu-miR-5113	ENSMUST00000223576	ENSMUSG00000017491
mmu_circ_0001499	mmu-miR-5113	ENSMUST00000223576	ENSMUSG00000017491
mmu_circ_0001500	mmu-miR-5113	ENSMUST00000223576	ENSMUSG00000017491
mmu_circ_0001755	mmu-miR-5113	ENSMUST00000223576	ENSMUSG00000017491
mmu_circ_0001767	mmu-miR-5113	ENSMUST00000223576	ENSMUSG00000017491
mmu_circ_0001775	mmu-miR-5113	ENSMUST00000223576	ENSMUSG00000017491
mmu_circ_0001775	mmu-miR-5113	ENSMUST00000223576	ENSMUSG00000017491
mmu_circ_0001825	mmu-miR-5113	ENSMUST00000223576	ENSMUSG00000017491
mmu_circ_0001827	mmu-miR-5113	ENSMUST00000223576	ENSMUSG00000017491
mmu_circ_0000333	mmu-miR-5113	ENSMUST00000223576	ENSMUSG00000017491
mmu_circ_0000148	mmu-miR-5113	ENSMUST00000223576	ENSMUSG00000017491
mmu_circ_0000149	mmu-miR-5113	ENSMUST00000223576	ENSMUSG00000017491
mmu_circ_0000499	mmu-miR-5113	ENSMUST00000223576	ENSMUSG00000017491
mmu_circ_0000500	mmu-miR-5113	ENSMUST00000223576	ENSMUSG00000017491
mmu_circ_0000491	mmu-miR-5113	ENSMUST00000223576	ENSMUSG00000017491
mmu_circ_0000402	mmu-miR-5113	ENSMUST00000223576	ENSMUSG00000017491
mmu_circ_0000603	mmu-miR-5113	ENSMUST00000223576	ENSMUSG00000017491
mmu_circ_0000644	mmu-miR-5113	ENSMUST00000223576	ENSMUSG00000017491
mmu_circ_0000526	mmu-miR-5113	ENSMUST00000223576	ENSMUSG00000017491
mmu_circ_0000526	mmu-miR-5113	ENSMUST00000223576	ENSMUSG00000017491
mmu_circ_0000530	mmu-miR-5113	ENSMUST00000223576	ENSMUSG00000017491
mmu_circ_0000555	mmu-miR-5113	ENSMUST00000223576	ENSMUSG00000017491
mmu_circ_0000735	mmu-miR-5113	ENSMUST00000223576	ENSMUSG00000017491
mmu_circ_0000828	mmu-miR-5113	ENSMUST00000223576	ENSMUSG00000017491
mmu_circ_0000829	mmu-miR-5113	ENSMUST00000223576	ENSMUSG00000017491
mmu_circ_0000833	mmu-miR-5113	ENSMUST00000223576	ENSMUSG00000017491
mmu_circ_0000712	mmu-miR-5113	ENSMUST00000223576	ENSMUSG00000017491
mmu_circ_0000714	mmu-miR-5113	ENSMUST00000223576	ENSMUSG00000017491
mmu_circ_0000950	mmu-miR-5113	ENSMUST00000223576	ENSMUSG00000017491

mmu_circ_0000969	mmu-miR-5113	ENSMUST00000223576	ENSMUSG00000017491
mmu_circ_0000884	mmu-miR-5113	ENSMUST00000223576	ENSMUSG00000017491
mmu_circ_0000886	mmu-miR-5113	ENSMUST00000223576	ENSMUSG00000017491
mmu_circ_0000887	mmu-miR-5113	ENSMUST00000223576	ENSMUSG00000017491
mmu_circ_0000898	mmu-miR-5113	ENSMUST00000223576	ENSMUSG00000017491
mmu_circ_0000905	mmu-miR-5113	ENSMUST00000223576	ENSMUSG00000017491
mmu_circ_0000905	mmu-miR-5113	ENSMUST00000223576	ENSMUSG00000017491
mmu_circ_0000906	mmu-miR-5113	ENSMUST00000223576	ENSMUSG00000017491
mmu_circ_0000907	mmu-miR-5113	ENSMUST00000223576	ENSMUSG00000017491
mmu_circ_0000912	mmu-miR-5113	ENSMUST00000223576	ENSMUSG00000017491
novel_circ_0001561	mmu-miR-5113	ENSMUST00000223576	ENSMUSG00000017491
novel_circ_0001696	mmu-miR-5113	ENSMUST00000223576	ENSMUSG00000017491
novel_circ_0002080	mmu-miR-5113	ENSMUST00000223576	ENSMUSG00000017491
novel_circ_0002082	mmu-miR-5113	ENSMUST00000223576	ENSMUSG00000017491
novel_circ_0002082	mmu-miR-5113	ENSMUST00000223576	ENSMUSG00000017491
novel_circ_0002142	mmu-miR-5113	ENSMUST00000223576	ENSMUSG00000017491
novel_circ_0001766	mmu-miR-5113	ENSMUST00000223576	ENSMUSG00000017491
novel_circ_0001815	mmu-miR-5113	ENSMUST00000223576	ENSMUSG00000017491
novel_circ_0001905	mmu-miR-5113	ENSMUST00000223576	ENSMUSG00000017491
novel_circ_0001913	mmu-miR-5113	ENSMUST00000223576	ENSMUSG00000017491
novel_circ_0001954	mmu-miR-5113	ENSMUST00000223576	ENSMUSG00000017491
novel_circ_0001954	mmu-miR-5113	ENSMUST00000223576	ENSMUSG00000017491
novel_circ_0001994	mmu-miR-5113	ENSMUST00000223576	ENSMUSG00000017491
novel_circ_0001998	mmu-miR-5113	ENSMUST00000223576	ENSMUSG00000017491
novel_circ_0002002	mmu-miR-5113	ENSMUST00000223576	ENSMUSG00000017491
novel_circ_0002031	mmu-miR-5113	ENSMUST00000223576	ENSMUSG00000017491
novel_circ_0002051	mmu-miR-5113	ENSMUST00000223576	ENSMUSG00000017491
novel_circ_0002413	mmu-miR-5113	ENSMUST00000223576	ENSMUSG00000017491
novel_circ_0002459	mmu-miR-5113	ENSMUST00000223576	ENSMUSG00000017491
novel_circ_0002472	mmu-miR-5113	ENSMUST00000223576	ENSMUSG00000017491
novel_circ_0002481	mmu-miR-5113	ENSMUST00000223576	ENSMUSG00000017491
novel_circ_0002518	mmu-miR-5113	ENSMUST00000223576	ENSMUSG00000017491
novel_circ_0002526	mmu-miR-5113	ENSMUST00000223576	ENSMUSG00000017491
novel_circ_0002228	mmu-miR-5113	ENSMUST00000223576	ENSMUSG00000017491
novel_circ_0002274	mmu-miR-5113	ENSMUST00000223576	ENSMUSG00000017491
novel_circ_0002274	mmu-miR-5113	ENSMUST00000223576	ENSMUSG00000017491
novel_circ_0002324	mmu-miR-5113	ENSMUST00000223576	ENSMUSG00000017491
novel_circ_0002374	mmu-miR-5113	ENSMUST00000223576	ENSMUSG00000017491
novel_circ_0002831	mmu-miR-5113	ENSMUST00000223576	ENSMUSG00000017491
novel_circ_0002866	mmu-miR-5113	ENSMUST00000223576	ENSMUSG00000017491
novel_circ_0002872	mmu-miR-5113	ENSMUST00000223576	ENSMUSG00000017491
novel_circ_0002946	mmu-miR-5113	ENSMUST00000223576	ENSMUSG00000017491
novel_circ_0003188	mmu-miR-5113	ENSMUST00000223576	ENSMUSG00000017491
novel_circ_0003229	mmu-miR-5113	ENSMUST00000223576	ENSMUSG00000017491
novel_circ_0003229	mmu-miR-5113	ENSMUST00000223576	ENSMUSG00000017491
novel_circ_0003270	mmu-miR-5113	ENSMUST00000223576	ENSMUSG00000017491
novel_circ_0003304	mmu-miR-5113	ENSMUST00000223576	ENSMUSG00000017491
novel_circ_0003377	mmu-miR-5113	ENSMUST00000223576	ENSMUSG00000017491
novel_circ_0003397	mmu-miR-5113	ENSMUST00000223576	ENSMUSG00000017491
novel_circ_0003503	mmu-miR-5113	ENSMUST00000223576	ENSMUSG00000017491
novel_circ_0000258	mmu-miR-5113	ENSMUST00000223576	ENSMUSG00000017491
novel_circ_0000278	mmu-miR-5113	ENSMUST00000223576	ENSMUSG00000017491
novel_circ_0000296	mmu-miR-5113	ENSMUST00000223576	ENSMUSG00000017491
novel_circ_0000296	mmu-miR-5113	ENSMUST00000223576	ENSMUSG00000017491
novel_circ_0000395	mmu-miR-5113	ENSMUST00000223576	ENSMUSG00000017491
novel_circ_0000444	mmu-miR-5113	ENSMUST00000223576	ENSMUSG00000017491
novel_circ_0000447	mmu-miR-5113	ENSMUST00000223576	ENSMUSG00000017491
novel_circ_0000051	mmu-miR-5113	ENSMUST00000223576	ENSMUSG00000017491

novel_circ_0000068	mmu-miR-5113	ENSMUST00000223576	ENSMUSG00000017491
novel_circ_0000100	mmu-miR-5113	ENSMUST00000223576	ENSMUSG00000017491
novel_circ_0000109	mmu-miR-5113	ENSMUST00000223576	ENSMUSG00000017491
novel_circ_0000620	mmu-miR-5113	ENSMUST00000223576	ENSMUSG00000017491
novel_circ_0000519	mmu-miR-5113	ENSMUST00000223576	ENSMUSG00000017491
novel_circ_0000520	mmu-miR-5113	ENSMUST00000223576	ENSMUSG00000017491
novel_circ_0000521	mmu-miR-5113	ENSMUST00000223576	ENSMUSG00000017491
novel_circ_0000589	mmu-miR-5113	ENSMUST00000223576	ENSMUSG00000017491
novel_circ_0000963	mmu-miR-5113	ENSMUST00000223576	ENSMUSG00000017491
novel_circ_0000963	mmu-miR-5113	ENSMUST00000223576	ENSMUSG00000017491
novel_circ_0000814	mmu-miR-5113	ENSMUST00000223576	ENSMUSG00000017491
novel_circ_0001177	mmu-miR-5113	ENSMUST00000223576	ENSMUSG00000017491
novel_circ_0001179	mmu-miR-5113	ENSMUST00000223576	ENSMUSG00000017491
novel_circ_0001188	mmu-miR-5113	ENSMUST00000223576	ENSMUSG00000017491
novel_circ_0001221	mmu-miR-378d	ENSMUST00000000128	ENSMUSG00000000126
novel_circ_0001221	mmu-miR-378d	ENSMUST00000108783	ENSMUSG00000000126
novel_circ_0001224	mmu-miR-378d	ENSMUST00000000128	ENSMUSG00000000126
novel_circ_0001224	mmu-miR-378d	ENSMUST00000108783	ENSMUSG00000000126
novel_circ_0001534	mmu-miR-378d	ENSMUST00000000128	ENSMUSG00000000126
novel_circ_0001534	mmu-miR-378d	ENSMUST00000108783	ENSMUSG00000000126
novel_circ_0001375	mmu-miR-378d	ENSMUST00000000128	ENSMUSG00000000126
novel_circ_0001375	mmu-miR-378d	ENSMUST00000108783	ENSMUSG00000000126
mmu_circ_0000075	mmu-miR-378d	ENSMUST00000000128	ENSMUSG00000000126
mmu_circ_0000075	mmu-miR-378d	ENSMUST00000108783	ENSMUSG00000000126
mmu_circ_0000086	mmu-miR-378d	ENSMUST00000000128	ENSMUSG00000000126
mmu_circ_0000086	mmu-miR-378d	ENSMUST00000108783	ENSMUSG00000000126
mmu_circ_0000087	mmu-miR-378d	ENSMUST00000000128	ENSMUSG00000000126
mmu_circ_0000087	mmu-miR-378d	ENSMUST00000108783	ENSMUSG00000000126
mmu_circ_0000028	mmu-miR-378d	ENSMUST00000000128	ENSMUSG00000000126
mmu_circ_0000028	mmu-miR-378d	ENSMUST00000108783	ENSMUSG00000000126
mmu_circ_0000042	mmu-miR-378d	ENSMUST00000000128	ENSMUSG00000000126
mmu_circ_0000042	mmu-miR-378d	ENSMUST00000108783	ENSMUSG00000000126
mmu_circ_0000063	mmu-miR-378d	ENSMUST00000000128	ENSMUSG00000000126
mmu_circ_0000063	mmu-miR-378d	ENSMUST00000108783	ENSMUSG00000000126
mmu_circ_0000063	mmu-miR-378d	ENSMUST00000000128	ENSMUSG00000000126
mmu_circ_0000063	mmu-miR-378d	ENSMUST00000108783	ENSMUSG00000000126
mmu_circ_0000064	mmu-miR-378d	ENSMUST00000000128	ENSMUSG00000000126
mmu_circ_0000064	mmu-miR-378d	ENSMUST00000108783	ENSMUSG00000000126
mmu_circ_0000064	mmu-miR-378d	ENSMUST00000000128	ENSMUSG00000000126
mmu_circ_0000064	mmu-miR-378d	ENSMUST00000108783	ENSMUSG00000000126
mmu_circ_0001154	mmu-miR-378d	ENSMUST00000000128	ENSMUSG00000000126
mmu_circ_0001154	mmu-miR-378d	ENSMUST00000108783	ENSMUSG00000000126
mmu_circ_0001123	mmu-miR-378d	ENSMUST00000000128	ENSMUSG00000000126
mmu_circ_0001123	mmu-miR-378d	ENSMUST00000108783	ENSMUSG00000000126
mmu_circ_0001148	mmu-miR-378d	ENSMUST00000000128	ENSMUSG00000000126
mmu_circ_0001148	mmu-miR-378d	ENSMUST00000108783	ENSMUSG00000000126
mmu_circ_0001368	mmu-miR-378d	ENSMUST00000000128	ENSMUSG00000000126
mmu_circ_0001368	mmu-miR-378d	ENSMUST00000108783	ENSMUSG00000000126
mmu_circ_0001376	mmu-miR-378d	ENSMUST00000000128	ENSMUSG00000000126
mmu_circ_0001376	mmu-miR-378d	ENSMUST00000108783	ENSMUSG00000000126
mmu_circ_0001279	mmu-miR-378d	ENSMUST00000000128	ENSMUSG00000000126
mmu_circ_0001279	mmu-miR-378d	ENSMUST00000108783	ENSMUSG00000000126
mmu_circ_0001634	mmu-miR-378d	ENSMUST00000000128	ENSMUSG00000000126
mmu_circ_0001634	mmu-miR-378d	ENSMUST00000108783	ENSMUSG00000000126
mmu_circ_0001505	mmu-miR-378d	ENSMUST00000000128	ENSMUSG00000000126
mmu_circ_0001505	mmu-miR-378d	ENSMUST00000108783	ENSMUSG00000000126
mmu_circ_0001528	mmu-miR-378d	ENSMUST00000000128	ENSMUSG00000000126
mmu_circ_0001528	mmu-miR-378d	ENSMUST00000108783	ENSMUSG00000000126

mmu_circ_0001454	mmu-miR-378d	ENSMUST00000000128	ENSMUSG00000000126
mmu_circ_0001454	mmu-miR-378d	ENSMUST00000108783	ENSMUSG00000000126
mmu_circ_0001478	mmu-miR-378d	ENSMUST00000000128	ENSMUSG00000000126
mmu_circ_0001478	mmu-miR-378d	ENSMUST00000108783	ENSMUSG00000000126
mmu_circ_0001499	mmu-miR-378d	ENSMUST00000000128	ENSMUSG00000000126
mmu_circ_0001499	mmu-miR-378d	ENSMUST00000108783	ENSMUSG00000000126
mmu_circ_0001500	mmu-miR-378d	ENSMUST00000000128	ENSMUSG00000000126
mmu_circ_0001500	mmu-miR-378d	ENSMUST00000108783	ENSMUSG00000000126
mmu_circ_0001842	mmu-miR-378d	ENSMUST00000000128	ENSMUSG00000000126
mmu_circ_0001842	mmu-miR-378d	ENSMUST00000108783	ENSMUSG00000000126
mmu_circ_0001827	mmu-miR-378d	ENSMUST00000000128	ENSMUSG00000000126
mmu_circ_0001827	mmu-miR-378d	ENSMUST00000108783	ENSMUSG00000000126
mmu_circ_0000257	mmu-miR-378d	ENSMUST00000000128	ENSMUSG00000000126
mmu_circ_0000257	mmu-miR-378d	ENSMUST00000108783	ENSMUSG00000000126
mmu_circ_0000230	mmu-miR-378d	ENSMUST00000000128	ENSMUSG00000000126
mmu_circ_0000230	mmu-miR-378d	ENSMUST00000108783	ENSMUSG00000000126
mmu_circ_0000301	mmu-miR-378d	ENSMUST00000000128	ENSMUSG00000000126
mmu_circ_0000301	mmu-miR-378d	ENSMUST00000108783	ENSMUSG00000000126
mmu_circ_0000163	mmu-miR-378d	ENSMUST00000000128	ENSMUSG00000000126
mmu_circ_0000163	mmu-miR-378d	ENSMUST00000108783	ENSMUSG00000000126
mmu_circ_0000499	mmu-miR-378d	ENSMUST00000000128	ENSMUSG00000000126
mmu_circ_0000499	mmu-miR-378d	ENSMUST00000108783	ENSMUSG00000000126
mmu_circ_0000500	mmu-miR-378d	ENSMUST00000000128	ENSMUSG00000000126
mmu_circ_0000500	mmu-miR-378d	ENSMUST00000108783	ENSMUSG00000000126
mmu_circ_0000465	mmu-miR-378d	ENSMUST00000000128	ENSMUSG00000000126
mmu_circ_0000465	mmu-miR-378d	ENSMUST00000108783	ENSMUSG00000000126
mmu_circ_0000399	mmu-miR-378d	ENSMUST00000000128	ENSMUSG00000000126
mmu_circ_0000399	mmu-miR-378d	ENSMUST00000108783	ENSMUSG00000000126
mmu_circ_0000402	mmu-miR-378d	ENSMUST00000000128	ENSMUSG00000000126
mmu_circ_0000402	mmu-miR-378d	ENSMUST00000108783	ENSMUSG00000000126
mmu_circ_0000625	mmu-miR-378d	ENSMUST00000000128	ENSMUSG00000000126
mmu_circ_0000625	mmu-miR-378d	ENSMUST00000108783	ENSMUSG00000000126
mmu_circ_0000504	mmu-miR-378d	ENSMUST00000000128	ENSMUSG00000000126
mmu_circ_0000504	mmu-miR-378d	ENSMUST00000108783	ENSMUSG00000000126
mmu_circ_0000530	mmu-miR-378d	ENSMUST00000000128	ENSMUSG00000000126
mmu_circ_0000530	mmu-miR-378d	ENSMUST00000108783	ENSMUSG00000000126
mmu_circ_0000738	mmu-miR-378d	ENSMUST00000000128	ENSMUSG00000000126
mmu_circ_0000738	mmu-miR-378d	ENSMUST00000108783	ENSMUSG00000000126
mmu_circ_0000798	mmu-miR-378d	ENSMUST00000000128	ENSMUSG00000000126
mmu_circ_0000798	mmu-miR-378d	ENSMUST00000108783	ENSMUSG00000000126
mmu_circ_0000829	mmu-miR-378d	ENSMUST00000000128	ENSMUSG00000000126
mmu_circ_0000829	mmu-miR-378d	ENSMUST00000108783	ENSMUSG00000000126
mmu_circ_0000831	mmu-miR-378d	ENSMUST00000000128	ENSMUSG00000000126
mmu_circ_0000831	mmu-miR-378d	ENSMUST00000108783	ENSMUSG00000000126
mmu_circ_0000711	mmu-miR-378d	ENSMUST00000000128	ENSMUSG00000000126
mmu_circ_0000711	mmu-miR-378d	ENSMUST00000108783	ENSMUSG00000000126
mmu_circ_0000712	mmu-miR-378d	ENSMUST00000000128	ENSMUSG00000000126
mmu_circ_0000712	mmu-miR-378d	ENSMUST00000108783	ENSMUSG00000000126
mmu_circ_0000713	mmu-miR-378d	ENSMUST00000000128	ENSMUSG00000000126
mmu_circ_0000713	mmu-miR-378d	ENSMUST00000108783	ENSMUSG00000000126
mmu_circ_0000714	mmu-miR-378d	ENSMUST00000000128	ENSMUSG00000000126
mmu_circ_0000714	mmu-miR-378d	ENSMUST00000108783	ENSMUSG00000000126
mmu_circ_0000950	mmu-miR-378d	ENSMUST00000000128	ENSMUSG00000000126
mmu_circ_0000950	mmu-miR-378d	ENSMUST00000108783	ENSMUSG00000000126
mmu_circ_0000969	mmu-miR-378d	ENSMUST00000000128	ENSMUSG00000000126
mmu_circ_0000969	mmu-miR-378d	ENSMUST00000108783	ENSMUSG00000000126
mmu_circ_0000865	mmu-miR-378d	ENSMUST00000000128	ENSMUSG00000000126
mmu_circ_0000865	mmu-miR-378d	ENSMUST00000108783	ENSMUSG00000000126

mmu_circ_0000840	mmu-miR-378d	ENSMUST00000000128	ENSMUSG00000000126
mmu_circ_0000840	mmu-miR-378d	ENSMUST00000108783	ENSMUSG00000000126
mmu_circ_0000884	mmu-miR-378d	ENSMUST00000000128	ENSMUSG00000000126
mmu_circ_0000884	mmu-miR-378d	ENSMUST00000108783	ENSMUSG00000000126
mmu_circ_0000886	mmu-miR-378d	ENSMUST00000000128	ENSMUSG00000000126
mmu_circ_0000886	mmu-miR-378d	ENSMUST00000108783	ENSMUSG00000000126
mmu_circ_0000887	mmu-miR-378d	ENSMUST00000000128	ENSMUSG00000000126
mmu_circ_0000887	mmu-miR-378d	ENSMUST00000108783	ENSMUSG00000000126
mmu_circ_0000906	mmu-miR-378d	ENSMUST00000000128	ENSMUSG00000000126
mmu_circ_0000906	mmu-miR-378d	ENSMUST00000108783	ENSMUSG00000000126
mmu_circ_0000912	mmu-miR-378d	ENSMUST00000000128	ENSMUSG00000000126
mmu_circ_0000912	mmu-miR-378d	ENSMUST00000108783	ENSMUSG00000000126
novel_circ_0001551	mmu-miR-378d	ENSMUST00000000128	ENSMUSG00000000126
novel_circ_0001551	mmu-miR-378d	ENSMUST00000108783	ENSMUSG00000000126
novel_circ_0001561	mmu-miR-378d	ENSMUST00000000128	ENSMUSG00000000126
novel_circ_0001561	mmu-miR-378d	ENSMUST00000108783	ENSMUSG00000000126
novel_circ_0001570	mmu-miR-378d	ENSMUST00000000128	ENSMUSG00000000126
novel_circ_0001570	mmu-miR-378d	ENSMUST00000108783	ENSMUSG00000000126
novel_circ_0001623	mmu-miR-378d	ENSMUST00000000128	ENSMUSG00000000126
novel_circ_0001623	mmu-miR-378d	ENSMUST00000108783	ENSMUSG00000000126
novel_circ_0001680	mmu-miR-378d	ENSMUST00000000128	ENSMUSG00000000126
novel_circ_0001680	mmu-miR-378d	ENSMUST00000108783	ENSMUSG00000000126
novel_circ_0001710	mmu-miR-378d	ENSMUST00000000128	ENSMUSG00000000126
novel_circ_0001710	mmu-miR-378d	ENSMUST00000108783	ENSMUSG00000000126
novel_circ_0001727	mmu-miR-378d	ENSMUST00000000128	ENSMUSG00000000126
novel_circ_0001727	mmu-miR-378d	ENSMUST00000108783	ENSMUSG00000000126
novel_circ_0001728	mmu-miR-378d	ENSMUST00000000128	ENSMUSG00000000126
novel_circ_0001728	mmu-miR-378d	ENSMUST00000108783	ENSMUSG00000000126
novel_circ_0002102	mmu-miR-378d	ENSMUST00000000128	ENSMUSG00000000126
novel_circ_0002102	mmu-miR-378d	ENSMUST00000108783	ENSMUSG00000000126
novel_circ_0002102	mmu-miR-378d	ENSMUST00000000128	ENSMUSG00000000126
novel_circ_0002102	mmu-miR-378d	ENSMUST00000108783	ENSMUSG00000000126
novel_circ_0002123	mmu-miR-378d	ENSMUST00000000128	ENSMUSG00000000126
novel_circ_0002123	mmu-miR-378d	ENSMUST00000108783	ENSMUSG00000000126
novel_circ_0002179	mmu-miR-378d	ENSMUST00000000128	ENSMUSG00000000126
novel_circ_0002179	mmu-miR-378d	ENSMUST00000108783	ENSMUSG00000000126
novel_circ_0002192	mmu-miR-378d	ENSMUST00000000128	ENSMUSG00000000126
novel_circ_0002192	mmu-miR-378d	ENSMUST00000108783	ENSMUSG00000000126
novel_circ_0002197	mmu-miR-378d	ENSMUST00000000128	ENSMUSG00000000126
novel_circ_0002197	mmu-miR-378d	ENSMUST00000108783	ENSMUSG00000000126
novel_circ_0002198	mmu-miR-378d	ENSMUST00000000128	ENSMUSG00000000126
novel_circ_0002198	mmu-miR-378d	ENSMUST00000108783	ENSMUSG00000000126
novel_circ_0002216	mmu-miR-378d	ENSMUST00000000128	ENSMUSG00000000126
novel_circ_0002216	mmu-miR-378d	ENSMUST00000108783	ENSMUSG00000000126
novel_circ_0001776	mmu-miR-378d	ENSMUST00000000128	ENSMUSG00000000126
novel_circ_0001776	mmu-miR-378d	ENSMUST00000108783	ENSMUSG00000000126
novel_circ_0001819	mmu-miR-378d	ENSMUST00000000128	ENSMUSG00000000126
novel_circ_0001819	mmu-miR-378d	ENSMUST00000108783	ENSMUSG00000000126
novel_circ_0001875	mmu-miR-378d	ENSMUST00000000128	ENSMUSG00000000126
novel_circ_0001875	mmu-miR-378d	ENSMUST00000108783	ENSMUSG00000000126
novel_circ_0001891	mmu-miR-378d	ENSMUST00000000128	ENSMUSG00000000126
novel_circ_0001891	mmu-miR-378d	ENSMUST00000108783	ENSMUSG00000000126
novel_circ_0001998	mmu-miR-378d	ENSMUST00000000128	ENSMUSG00000000126
novel_circ_0001998	mmu-miR-378d	ENSMUST00000108783	ENSMUSG00000000126
novel_circ_0002417	mmu-miR-378d	ENSMUST00000000128	ENSMUSG00000000126
novel_circ_0002417	mmu-miR-378d	ENSMUST00000108783	ENSMUSG00000000126
novel_circ_0002426	mmu-miR-378d	ENSMUST00000000128	ENSMUSG00000000126
novel_circ_0002426	mmu-miR-378d	ENSMUST00000108783	ENSMUSG00000000126

novel_circ_0002491	mmu-miR-378d	ENSMUST00000000128	ENSMUSG00000000126
novel_circ_0002491	mmu-miR-378d	ENSMUST00000108783	ENSMUSG00000000126
novel_circ_0002491	mmu-miR-378d	ENSMUST00000000128	ENSMUSG00000000126
novel_circ_0002491	mmu-miR-378d	ENSMUST00000108783	ENSMUSG00000000126
novel_circ_0002494	mmu-miR-378d	ENSMUST00000000128	ENSMUSG00000000126
novel_circ_0002494	mmu-miR-378d	ENSMUST00000108783	ENSMUSG00000000126
novel_circ_0002503	mmu-miR-378d	ENSMUST00000000128	ENSMUSG00000000126
novel_circ_0002503	mmu-miR-378d	ENSMUST00000108783	ENSMUSG00000000126
novel_circ_0002504	mmu-miR-378d	ENSMUST00000000128	ENSMUSG00000000126
novel_circ_0002504	mmu-miR-378d	ENSMUST00000108783	ENSMUSG00000000126
novel_circ_0002518	mmu-miR-378d	ENSMUST00000000128	ENSMUSG00000000126
novel_circ_0002518	mmu-miR-378d	ENSMUST00000108783	ENSMUSG00000000126
novel_circ_0002519	mmu-miR-378d	ENSMUST00000000128	ENSMUSG00000000126
novel_circ_0002519	mmu-miR-378d	ENSMUST00000108783	ENSMUSG00000000126
novel_circ_0002578	mmu-miR-378d	ENSMUST00000000128	ENSMUSG00000000126
novel_circ_0002578	mmu-miR-378d	ENSMUST00000108783	ENSMUSG00000000126
novel_circ_0002597	mmu-miR-378d	ENSMUST00000000128	ENSMUSG00000000126
novel_circ_0002597	mmu-miR-378d	ENSMUST00000108783	ENSMUSG00000000126
novel_circ_0002274	mmu-miR-378d	ENSMUST00000000128	ENSMUSG00000000126
novel_circ_0002274	mmu-miR-378d	ENSMUST00000108783	ENSMUSG00000000126
novel_circ_0002288	mmu-miR-378d	ENSMUST00000000128	ENSMUSG00000000126
novel_circ_0002288	mmu-miR-378d	ENSMUST00000108783	ENSMUSG00000000126
novel_circ_0002292	mmu-miR-378d	ENSMUST00000000128	ENSMUSG00000000126
novel_circ_0002292	mmu-miR-378d	ENSMUST00000108783	ENSMUSG00000000126
novel_circ_0002301	mmu-miR-378d	ENSMUST00000000128	ENSMUSG00000000126
novel_circ_0002301	mmu-miR-378d	ENSMUST00000108783	ENSMUSG00000000126
novel_circ_0002378	mmu-miR-378d	ENSMUST00000000128	ENSMUSG00000000126
novel_circ_0002378	mmu-miR-378d	ENSMUST00000108783	ENSMUSG00000000126
novel_circ_0002379	mmu-miR-378d	ENSMUST00000000128	ENSMUSG00000000126
novel_circ_0002379	mmu-miR-378d	ENSMUST00000108783	ENSMUSG00000000126
novel_circ_0002826	mmu-miR-378d	ENSMUST00000000128	ENSMUSG00000000126
novel_circ_0002826	mmu-miR-378d	ENSMUST00000108783	ENSMUSG00000000126
novel_circ_0002895	mmu-miR-378d	ENSMUST00000000128	ENSMUSG00000000126
novel_circ_0002895	mmu-miR-378d	ENSMUST00000108783	ENSMUSG00000000126
novel_circ_0002898	mmu-miR-378d	ENSMUST00000000128	ENSMUSG00000000126
novel_circ_0002898	mmu-miR-378d	ENSMUST00000108783	ENSMUSG00000000126
novel_circ_0002900	mmu-miR-378d	ENSMUST00000000128	ENSMUSG00000000126
novel_circ_0002900	mmu-miR-378d	ENSMUST00000108783	ENSMUSG00000000126
novel_circ_0002902	mmu-miR-378d	ENSMUST00000000128	ENSMUSG00000000126
novel_circ_0002902	mmu-miR-378d	ENSMUST00000108783	ENSMUSG00000000126
novel_circ_0002903	mmu-miR-378d	ENSMUST00000000128	ENSMUSG00000000126
novel_circ_0002903	mmu-miR-378d	ENSMUST00000108783	ENSMUSG00000000126
novel_circ_0002969	mmu-miR-378d	ENSMUST00000000128	ENSMUSG00000000126
novel_circ_0002969	mmu-miR-378d	ENSMUST00000108783	ENSMUSG00000000126
novel_circ_0002969	mmu-miR-378d	ENSMUST00000000128	ENSMUSG00000000126
novel_circ_0002969	mmu-miR-378d	ENSMUST00000108783	ENSMUSG00000000126
novel_circ_0002671	mmu-miR-378d	ENSMUST00000000128	ENSMUSG00000000126
novel_circ_0002671	mmu-miR-378d	ENSMUST00000108783	ENSMUSG00000000126
novel_circ_0003295	mmu-miR-378d	ENSMUST00000000128	ENSMUSG00000000126
novel_circ_0003295	mmu-miR-378d	ENSMUST00000108783	ENSMUSG00000000126
novel_circ_0003298	mmu-miR-378d	ENSMUST00000000128	ENSMUSG00000000126
novel_circ_0003298	mmu-miR-378d	ENSMUST00000108783	ENSMUSG00000000126
novel_circ_0003298	mmu-miR-378d	ENSMUST00000000128	ENSMUSG00000000126
novel_circ_0003298	mmu-miR-378d	ENSMUST00000108783	ENSMUSG00000000126
novel_circ_0003370	mmu-miR-378d	ENSMUST00000000128	ENSMUSG00000000126
novel_circ_0003370	mmu-miR-378d	ENSMUST00000108783	ENSMUSG00000000126
novel_circ_0003022	mmu-miR-378d	ENSMUST00000000128	ENSMUSG00000000126
novel_circ_0003022	mmu-miR-378d	ENSMUST00000108783	ENSMUSG00000000126

novel_circ_0003032	mmu-miR-378d	ENSMUST00000000128	ENSMUSG00000000126
novel_circ_0003032	mmu-miR-378d	ENSMUST00000108783	ENSMUSG00000000126
novel_circ_0003053	mmu-miR-378d	ENSMUST00000000128	ENSMUSG00000000126
novel_circ_0003053	mmu-miR-378d	ENSMUST00000108783	ENSMUSG00000000126
novel_circ_0000170	mmu-miR-378d	ENSMUST00000000128	ENSMUSG00000000126
novel_circ_0000170	mmu-miR-378d	ENSMUST00000108783	ENSMUSG00000000126
novel_circ_0000273	mmu-miR-378d	ENSMUST00000000128	ENSMUSG00000000126
novel_circ_0000273	mmu-miR-378d	ENSMUST00000108783	ENSMUSG00000000126
novel_circ_0000005	mmu-miR-378d	ENSMUST00000000128	ENSMUSG00000000126
novel_circ_0000005	mmu-miR-378d	ENSMUST00000108783	ENSMUSG00000000126
novel_circ_0000073	mmu-miR-378d	ENSMUST00000000128	ENSMUSG00000000126
novel_circ_0000073	mmu-miR-378d	ENSMUST00000108783	ENSMUSG00000000126
novel_circ_0000519	mmu-miR-378d	ENSMUST00000000128	ENSMUSG00000000126
novel_circ_0000519	mmu-miR-378d	ENSMUST00000108783	ENSMUSG00000000126
novel_circ_0000520	mmu-miR-378d	ENSMUST00000000128	ENSMUSG00000000126
novel_circ_0000520	mmu-miR-378d	ENSMUST00000108783	ENSMUSG00000000126
novel_circ_0000530	mmu-miR-378d	ENSMUST00000000128	ENSMUSG00000000126
novel_circ_0000530	mmu-miR-378d	ENSMUST00000108783	ENSMUSG00000000126
novel_circ_0000537	mmu-miR-378d	ENSMUST00000000128	ENSMUSG00000000126
novel_circ_0000537	mmu-miR-378d	ENSMUST00000108783	ENSMUSG00000000126
novel_circ_0000587	mmu-miR-378d	ENSMUST00000000128	ENSMUSG00000000126
novel_circ_0000587	mmu-miR-378d	ENSMUST00000108783	ENSMUSG00000000126
novel_circ_0000800	mmu-miR-378d	ENSMUST00000000128	ENSMUSG00000000126
novel_circ_0000800	mmu-miR-378d	ENSMUST00000108783	ENSMUSG00000000126
novel_circ_0000801	mmu-miR-378d	ENSMUST00000000128	ENSMUSG00000000126
novel_circ_0000801	mmu-miR-378d	ENSMUST00000108783	ENSMUSG00000000126
novel_circ_0000888	mmu-miR-378d	ENSMUST00000000128	ENSMUSG00000000126
novel_circ_0000888	mmu-miR-378d	ENSMUST00000108783	ENSMUSG00000000126
novel_circ_0000888	mmu-miR-378d	ENSMUST00000000128	ENSMUSG00000000126
novel_circ_0000888	mmu-miR-378d	ENSMUST00000108783	ENSMUSG00000000126
novel_circ_0001188	mmu-miR-378d	ENSMUST00000000128	ENSMUSG00000000126
novel_circ_0001188	mmu-miR-378d	ENSMUST00000108783	ENSMUSG00000000126
novel_circ_0001189	mmu-miR-378d	ENSMUST00000000128	ENSMUSG00000000126
novel_circ_0001189	mmu-miR-378d	ENSMUST00000108783	ENSMUSG00000000126
novel_circ_0001198	mmu-miR-378d	ENSMUST00000000128	ENSMUSG00000000126
novel_circ_0001198	mmu-miR-378d	ENSMUST00000108783	ENSMUSG00000000126
novel_circ_0001232	mmu-miR-6904-5	ENSMUST00000000128	ENSMUSG00000000126
novel_circ_0001232	mmu-miR-6904-5	ENSMUST00000108783	ENSMUSG00000000126
novel_circ_0001268	mmu-miR-6904-5	ENSMUST00000000128	ENSMUSG00000000126
novel_circ_0001268	mmu-miR-6904-5	ENSMUST00000108783	ENSMUSG00000000126
novel_circ_0001076	mmu-miR-6904-5	ENSMUST00000000128	ENSMUSG00000000126
novel_circ_0001076	mmu-miR-6904-5	ENSMUST00000108783	ENSMUSG00000000126
novel_circ_0001083	mmu-miR-6904-5	ENSMUST00000000128	ENSMUSG00000000126
novel_circ_0001083	mmu-miR-6904-5	ENSMUST00000108783	ENSMUSG00000000126
novel_circ_0001495	mmu-miR-6904-5	ENSMUST00000000128	ENSMUSG00000000126
novel_circ_0001495	mmu-miR-6904-5	ENSMUST00000108783	ENSMUSG00000000126
novel_circ_0001543	mmu-miR-6904-5	ENSMUST00000000128	ENSMUSG00000000126
novel_circ_0001543	mmu-miR-6904-5	ENSMUST00000108783	ENSMUSG00000000126
novel_circ_0001315	mmu-miR-6904-5	ENSMUST00000000128	ENSMUSG00000000126
novel_circ_0001315	mmu-miR-6904-5	ENSMUST00000108783	ENSMUSG00000000126
novel_circ_0001355	mmu-miR-6904-5	ENSMUST00000000128	ENSMUSG00000000126
novel_circ_0001355	mmu-miR-6904-5	ENSMUST00000108783	ENSMUSG00000000126
novel_circ_0001356	mmu-miR-6904-5	ENSMUST00000000128	ENSMUSG00000000126
novel_circ_0001356	mmu-miR-6904-5	ENSMUST00000108783	ENSMUSG00000000126
mmu_circ_0001154	mmu-miR-6904-5	ENSMUST00000000128	ENSMUSG00000000126
mmu_circ_0001154	mmu-miR-6904-5	ENSMUST00000108783	ENSMUSG00000000126
mmu_circ_0001154	mmu-miR-6904-5	ENSMUST00000000128	ENSMUSG00000000126
mmu_circ_0001154	mmu-miR-6904-5	ENSMUST00000108783	ENSMUSG00000000126

mmu_circ_0001021	mmu-miR-6904-5	ENSMUST00000000128	ENSMUSG00000000126
mmu_circ_0001021	mmu-miR-6904-5	ENSMUST00000108783	ENSMUSG00000000126
mmu_circ_0001368	mmu-miR-6904-5	ENSMUST00000000128	ENSMUSG00000000126
mmu_circ_0001368	mmu-miR-6904-5	ENSMUST00000108783	ENSMUSG00000000126
mmu_circ_0001376	mmu-miR-6904-5	ENSMUST00000000128	ENSMUSG00000000126
mmu_circ_0001376	mmu-miR-6904-5	ENSMUST00000108783	ENSMUSG00000000126
mmu_circ_0001333	mmu-miR-6904-5	ENSMUST00000000128	ENSMUSG00000000126
mmu_circ_0001333	mmu-miR-6904-5	ENSMUST00000108783	ENSMUSG00000000126
mmu_circ_0001311	mmu-miR-6904-5	ENSMUST00000000128	ENSMUSG00000000126
mmu_circ_0001311	mmu-miR-6904-5	ENSMUST00000108783	ENSMUSG00000000126
mmu_circ_0001213	mmu-miR-6904-5	ENSMUST00000000128	ENSMUSG00000000126
mmu_circ_0001213	mmu-miR-6904-5	ENSMUST00000108783	ENSMUSG00000000126
mmu_circ_0001598	mmu-miR-6904-5	ENSMUST00000000128	ENSMUSG00000000126
mmu_circ_0001598	mmu-miR-6904-5	ENSMUST00000108783	ENSMUSG00000000126
mmu_circ_0001528	mmu-miR-6904-5	ENSMUST00000000128	ENSMUSG00000000126
mmu_circ_0001528	mmu-miR-6904-5	ENSMUST00000108783	ENSMUSG00000000126
mmu_circ_0001500	mmu-miR-6904-5	ENSMUST00000000128	ENSMUSG00000000126
mmu_circ_0001500	mmu-miR-6904-5	ENSMUST00000108783	ENSMUSG00000000126
mmu_circ_0001842	mmu-miR-6904-5	ENSMUST00000000128	ENSMUSG00000000126
mmu_circ_0001842	mmu-miR-6904-5	ENSMUST00000108783	ENSMUSG00000000126
mmu_circ_0001767	mmu-miR-6904-5	ENSMUST00000000128	ENSMUSG00000000126
mmu_circ_0001767	mmu-miR-6904-5	ENSMUST00000108783	ENSMUSG00000000126
mmu_circ_0001768	mmu-miR-6904-5	ENSMUST00000000128	ENSMUSG00000000126
mmu_circ_0001768	mmu-miR-6904-5	ENSMUST00000108783	ENSMUSG00000000126
mmu_circ_0001795	mmu-miR-6904-5	ENSMUST00000000128	ENSMUSG00000000126
mmu_circ_0001795	mmu-miR-6904-5	ENSMUST00000108783	ENSMUSG00000000126
mmu_circ_0001795	mmu-miR-6904-5	ENSMUST00000000128	ENSMUSG00000000126
mmu_circ_0001795	mmu-miR-6904-5	ENSMUST00000108783	ENSMUSG00000000126
mmu_circ_0001802	mmu-miR-6904-5	ENSMUST00000000128	ENSMUSG00000000126
mmu_circ_0001802	mmu-miR-6904-5	ENSMUST00000108783	ENSMUSG00000000126
mmu_circ_0001695	mmu-miR-6904-5	ENSMUST00000000128	ENSMUSG00000000126
mmu_circ_0001695	mmu-miR-6904-5	ENSMUST00000108783	ENSMUSG00000000126
mmu_circ_0000325	mmu-miR-6904-5	ENSMUST00000000128	ENSMUSG00000000126
mmu_circ_0000325	mmu-miR-6904-5	ENSMUST00000108783	ENSMUSG00000000126
mmu_circ_0000229	mmu-miR-6904-5	ENSMUST00000000128	ENSMUSG00000000126
mmu_circ_0000229	mmu-miR-6904-5	ENSMUST00000108783	ENSMUSG00000000126
mmu_circ_0000229	mmu-miR-6904-5	ENSMUST00000000128	ENSMUSG00000000126
mmu_circ_0000229	mmu-miR-6904-5	ENSMUST00000108783	ENSMUSG00000000126
mmu_circ_0000230	mmu-miR-6904-5	ENSMUST00000000128	ENSMUSG00000000126
mmu_circ_0000230	mmu-miR-6904-5	ENSMUST00000108783	ENSMUSG00000000126
mmu_circ_0000277	mmu-miR-6904-5	ENSMUST00000000128	ENSMUSG00000000126
mmu_circ_0000277	mmu-miR-6904-5	ENSMUST00000108783	ENSMUSG00000000126
mmu_circ_0000287	mmu-miR-6904-5	ENSMUST00000000128	ENSMUSG00000000126
mmu_circ_0000287	mmu-miR-6904-5	ENSMUST00000108783	ENSMUSG00000000126
mmu_circ_0000181	mmu-miR-6904-5	ENSMUST00000000128	ENSMUSG00000000126
mmu_circ_0000181	mmu-miR-6904-5	ENSMUST00000108783	ENSMUSG00000000126
mmu_circ_0000500	mmu-miR-6904-5	ENSMUST00000000128	ENSMUSG00000000126
mmu_circ_0000500	mmu-miR-6904-5	ENSMUST00000108783	ENSMUSG00000000126
mmu_circ_0000454	mmu-miR-6904-5	ENSMUST00000000128	ENSMUSG00000000126
mmu_circ_0000454	mmu-miR-6904-5	ENSMUST00000108783	ENSMUSG00000000126
mmu_circ_0000375	mmu-miR-6904-5	ENSMUST00000000128	ENSMUSG00000000126
mmu_circ_0000375	mmu-miR-6904-5	ENSMUST00000108783	ENSMUSG00000000126
mmu_circ_0000623	mmu-miR-6904-5	ENSMUST00000000128	ENSMUSG00000000126
mmu_circ_0000623	mmu-miR-6904-5	ENSMUST00000108783	ENSMUSG00000000126
mmu_circ_0000628	mmu-miR-6904-5	ENSMUST00000000128	ENSMUSG00000000126
mmu_circ_0000628	mmu-miR-6904-5	ENSMUST00000108783	ENSMUSG00000000126
mmu_circ_0000806	mmu-miR-6904-5	ENSMUST00000000128	ENSMUSG00000000126
mmu_circ_0000806	mmu-miR-6904-5	ENSMUST00000108783	ENSMUSG00000000126

mmu_circ_0000831	mmu-miR-6904-5	ENSMUST00000000128	ENSMUSG00000000126
mmu_circ_0000831	mmu-miR-6904-5	ENSMUST00000108783	ENSMUSG00000000126
mmu_circ_0000833	mmu-miR-6904-5	ENSMUST00000000128	ENSMUSG00000000126
mmu_circ_0000833	mmu-miR-6904-5	ENSMUST00000108783	ENSMUSG00000000126
mmu_circ_0000714	mmu-miR-6904-5	ENSMUST00000000128	ENSMUSG00000000126
mmu_circ_0000714	mmu-miR-6904-5	ENSMUST00000108783	ENSMUSG00000000126
mmu_circ_0000917	mmu-miR-6904-5	ENSMUST00000000128	ENSMUSG00000000126
mmu_circ_0000917	mmu-miR-6904-5	ENSMUST00000108783	ENSMUSG00000000126
mmu_circ_0000918	mmu-miR-6904-5	ENSMUST00000000128	ENSMUSG00000000126
mmu_circ_0000918	mmu-miR-6904-5	ENSMUST00000108783	ENSMUSG00000000126
mmu_circ_0000919	mmu-miR-6904-5	ENSMUST00000000128	ENSMUSG00000000126
mmu_circ_0000919	mmu-miR-6904-5	ENSMUST00000108783	ENSMUSG00000000126
mmu_circ_0000964	mmu-miR-6904-5	ENSMUST00000000128	ENSMUSG00000000126
mmu_circ_0000964	mmu-miR-6904-5	ENSMUST00000108783	ENSMUSG00000000126
mmu_circ_0000969	mmu-miR-6904-5	ENSMUST00000000128	ENSMUSG00000000126
mmu_circ_0000969	mmu-miR-6904-5	ENSMUST00000108783	ENSMUSG00000000126
mmu_circ_0000884	mmu-miR-6904-5	ENSMUST00000000128	ENSMUSG00000000126
mmu_circ_0000884	mmu-miR-6904-5	ENSMUST00000108783	ENSMUSG00000000126
mmu_circ_0000886	mmu-miR-6904-5	ENSMUST00000000128	ENSMUSG00000000126
mmu_circ_0000886	mmu-miR-6904-5	ENSMUST00000108783	ENSMUSG00000000126
mmu_circ_0000906	mmu-miR-6904-5	ENSMUST00000000128	ENSMUSG00000000126
mmu_circ_0000906	mmu-miR-6904-5	ENSMUST00000108783	ENSMUSG00000000126
mmu_circ_0000907	mmu-miR-6904-5	ENSMUST00000000128	ENSMUSG00000000126
mmu_circ_0000907	mmu-miR-6904-5	ENSMUST00000108783	ENSMUSG00000000126
mmu_circ_0000908	mmu-miR-6904-5	ENSMUST00000000128	ENSMUSG00000000126
mmu_circ_0000908	mmu-miR-6904-5	ENSMUST00000108783	ENSMUSG00000000126
mmu_circ_0000912	mmu-miR-6904-5	ENSMUST00000000128	ENSMUSG00000000126
mmu_circ_0000912	mmu-miR-6904-5	ENSMUST00000108783	ENSMUSG00000000126
novel_circ_0001561	mmu-miR-6904-5	ENSMUST00000000128	ENSMUSG00000000126
novel_circ_0001561	mmu-miR-6904-5	ENSMUST00000108783	ENSMUSG00000000126
novel_circ_0001579	mmu-miR-6904-5	ENSMUST00000000128	ENSMUSG00000000126
novel_circ_0001579	mmu-miR-6904-5	ENSMUST00000108783	ENSMUSG00000000126
novel_circ_0001676	mmu-miR-6904-5	ENSMUST00000000128	ENSMUSG00000000126
novel_circ_0001676	mmu-miR-6904-5	ENSMUST00000108783	ENSMUSG00000000126
novel_circ_0001726	mmu-miR-6904-5	ENSMUST00000000128	ENSMUSG00000000126
novel_circ_0001726	mmu-miR-6904-5	ENSMUST00000108783	ENSMUSG00000000126
novel_circ_0001726	mmu-miR-6904-5	ENSMUST00000000128	ENSMUSG00000000126
novel_circ_0001726	mmu-miR-6904-5	ENSMUST00000108783	ENSMUSG00000000126
novel_circ_0002061	mmu-miR-6904-5	ENSMUST00000000128	ENSMUSG00000000126
novel_circ_0002061	mmu-miR-6904-5	ENSMUST00000108783	ENSMUSG00000000126
novel_circ_0002100	mmu-miR-6904-5	ENSMUST00000000128	ENSMUSG00000000126
novel_circ_0002100	mmu-miR-6904-5	ENSMUST00000108783	ENSMUSG00000000126
novel_circ_0002105	mmu-miR-6904-5	ENSMUST00000000128	ENSMUSG00000000126
novel_circ_0002105	mmu-miR-6904-5	ENSMUST00000108783	ENSMUSG00000000126
novel_circ_0002123	mmu-miR-6904-5	ENSMUST00000000128	ENSMUSG00000000126
novel_circ_0002123	mmu-miR-6904-5	ENSMUST00000108783	ENSMUSG00000000126
novel_circ_0002180	mmu-miR-6904-5	ENSMUST00000000128	ENSMUSG00000000126
novel_circ_0002180	mmu-miR-6904-5	ENSMUST00000108783	ENSMUSG00000000126
novel_circ_0001875	mmu-miR-6904-5	ENSMUST00000000128	ENSMUSG00000000126
novel_circ_0001875	mmu-miR-6904-5	ENSMUST00000108783	ENSMUSG00000000126
novel_circ_0001888	mmu-miR-6904-5	ENSMUST00000000128	ENSMUSG00000000126
novel_circ_0001888	mmu-miR-6904-5	ENSMUST00000108783	ENSMUSG00000000126
novel_circ_0001958	mmu-miR-6904-5	ENSMUST00000000128	ENSMUSG00000000126
novel_circ_0001958	mmu-miR-6904-5	ENSMUST00000108783	ENSMUSG00000000126
novel_circ_0001978	mmu-miR-6904-5	ENSMUST00000000128	ENSMUSG00000000126
novel_circ_0001978	mmu-miR-6904-5	ENSMUST00000108783	ENSMUSG00000000126
novel_circ_0002417	mmu-miR-6904-5	ENSMUST00000000128	ENSMUSG00000000126
novel_circ_0002417	mmu-miR-6904-5	ENSMUST00000108783	ENSMUSG00000000126

novel_circ_0002459	mmu-miR-6904-5	ENSMUST00000000128	ENSMUSG00000000126
novel_circ_0002459	mmu-miR-6904-5	ENSMUST00000108783	ENSMUSG00000000126
novel_circ_0002468	mmu-miR-6904-5	ENSMUST00000000128	ENSMUSG00000000126
novel_circ_0002468	mmu-miR-6904-5	ENSMUST00000108783	ENSMUSG00000000126
novel_circ_0002494	mmu-miR-6904-5	ENSMUST00000000128	ENSMUSG00000000126
novel_circ_0002494	mmu-miR-6904-5	ENSMUST00000108783	ENSMUSG00000000126
novel_circ_0002248	mmu-miR-6904-5	ENSMUST00000000128	ENSMUSG00000000126
novel_circ_0002248	mmu-miR-6904-5	ENSMUST00000108783	ENSMUSG00000000126
novel_circ_0002276	mmu-miR-6904-5	ENSMUST00000000128	ENSMUSG00000000126
novel_circ_0002276	mmu-miR-6904-5	ENSMUST00000108783	ENSMUSG00000000126
novel_circ_0002376	mmu-miR-6904-5	ENSMUST00000000128	ENSMUSG00000000126
novel_circ_0002376	mmu-miR-6904-5	ENSMUST00000108783	ENSMUSG00000000126
novel_circ_0002844	mmu-miR-6904-5	ENSMUST00000000128	ENSMUSG00000000126
novel_circ_0002844	mmu-miR-6904-5	ENSMUST00000108783	ENSMUSG00000000126
novel_circ_0002876	mmu-miR-6904-5	ENSMUST00000000128	ENSMUSG00000000126
novel_circ_0002876	mmu-miR-6904-5	ENSMUST00000108783	ENSMUSG00000000126
novel_circ_0002883	mmu-miR-6904-5	ENSMUST00000000128	ENSMUSG00000000126
novel_circ_0002883	mmu-miR-6904-5	ENSMUST00000108783	ENSMUSG00000000126
novel_circ_0002943	mmu-miR-6904-5	ENSMUST00000000128	ENSMUSG00000000126
novel_circ_0002943	mmu-miR-6904-5	ENSMUST00000108783	ENSMUSG00000000126
novel_circ_0002943	mmu-miR-6904-5	ENSMUST00000000128	ENSMUSG00000000126
novel_circ_0002943	mmu-miR-6904-5	ENSMUST00000108783	ENSMUSG00000000126
novel_circ_0002648	mmu-miR-6904-5	ENSMUST00000000128	ENSMUSG00000000126
novel_circ_0002648	mmu-miR-6904-5	ENSMUST00000108783	ENSMUSG00000000126
novel_circ_0002656	mmu-miR-6904-5	ENSMUST00000000128	ENSMUSG00000000126
novel_circ_0002656	mmu-miR-6904-5	ENSMUST00000108783	ENSMUSG00000000126
novel_circ_0002699	mmu-miR-6904-5	ENSMUST00000000128	ENSMUSG00000000126
novel_circ_0002699	mmu-miR-6904-5	ENSMUST00000108783	ENSMUSG00000000126
novel_circ_0002732	mmu-miR-6904-5	ENSMUST00000000128	ENSMUSG00000000126
novel_circ_0002732	mmu-miR-6904-5	ENSMUST00000108783	ENSMUSG00000000126
novel_circ_0002788	mmu-miR-6904-5	ENSMUST00000000128	ENSMUSG00000000126
novel_circ_0002788	mmu-miR-6904-5	ENSMUST00000108783	ENSMUSG00000000126
novel_circ_0003335	mmu-miR-6904-5	ENSMUST00000000128	ENSMUSG00000000126
novel_circ_0003335	mmu-miR-6904-5	ENSMUST00000108783	ENSMUSG00000000126
novel_circ_0003374	mmu-miR-6904-5	ENSMUST00000000128	ENSMUSG00000000126
novel_circ_0003374	mmu-miR-6904-5	ENSMUST00000108783	ENSMUSG00000000126
novel_circ_0000212	mmu-miR-6904-5	ENSMUST00000000128	ENSMUSG00000000126
novel_circ_0000212	mmu-miR-6904-5	ENSMUST00000108783	ENSMUSG00000000126
novel_circ_0000212	mmu-miR-6904-5	ENSMUST00000000128	ENSMUSG00000000126
novel_circ_0000212	mmu-miR-6904-5	ENSMUST00000108783	ENSMUSG00000000126
novel_circ_0000412	mmu-miR-6904-5	ENSMUST00000000128	ENSMUSG00000000126
novel_circ_0000412	mmu-miR-6904-5	ENSMUST00000108783	ENSMUSG00000000126
novel_circ_0000436	mmu-miR-6904-5	ENSMUST00000000128	ENSMUSG00000000126
novel_circ_0000436	mmu-miR-6904-5	ENSMUST00000108783	ENSMUSG00000000126
novel_circ_0000022	mmu-miR-6904-5	ENSMUST00000000128	ENSMUSG00000000126
novel_circ_0000022	mmu-miR-6904-5	ENSMUST00000108783	ENSMUSG00000000126
novel_circ_0000088	mmu-miR-6904-5	ENSMUST00000000128	ENSMUSG00000000126
novel_circ_0000088	mmu-miR-6904-5	ENSMUST00000108783	ENSMUSG00000000126
novel_circ_0000127	mmu-miR-6904-5	ENSMUST00000000128	ENSMUSG00000000126
novel_circ_0000127	mmu-miR-6904-5	ENSMUST00000108783	ENSMUSG00000000126
novel_circ_0000127	mmu-miR-6904-5	ENSMUST00000000128	ENSMUSG00000000126
novel_circ_0000127	mmu-miR-6904-5	ENSMUST00000108783	ENSMUSG00000000126
novel_circ_0000670	mmu-miR-6904-5	ENSMUST00000000128	ENSMUSG00000000126
novel_circ_0000670	mmu-miR-6904-5	ENSMUST00000108783	ENSMUSG00000000126
novel_circ_0000671	mmu-miR-6904-5	ENSMUST00000000128	ENSMUSG00000000126
novel_circ_0000671	mmu-miR-6904-5	ENSMUST00000108783	ENSMUSG00000000126
novel_circ_0000956	mmu-miR-6904-5	ENSMUST00000000128	ENSMUSG00000000126
novel_circ_0000956	mmu-miR-6904-5	ENSMUST00000108783	ENSMUSG00000000126

novel_circ_0000957	mmu-miR-6904-5	ENSMUST00000000128	ENSMUSG00000000126
novel_circ_0000957	mmu-miR-6904-5	ENSMUST00000108783	ENSMUSG00000000126
novel_circ_0001010	mmu-miR-6904-5	ENSMUST00000000128	ENSMUSG00000000126
novel_circ_0001010	mmu-miR-6904-5	ENSMUST00000108783	ENSMUSG00000000126
novel_circ_0001033	mmu-miR-6904-5	ENSMUST00000000128	ENSMUSG00000000126
novel_circ_0001033	mmu-miR-6904-5	ENSMUST00000108783	ENSMUSG00000000126
novel_circ_0001191	mmu-miR-6904-5	ENSMUST00000000128	ENSMUSG00000000126
novel_circ_0001191	mmu-miR-6904-5	ENSMUST00000108783	ENSMUSG00000000126
novel_circ_0001206	mmu-miR-6904-5	ENSMUST00000000128	ENSMUSG00000000126
novel_circ_0001206	mmu-miR-6904-5	ENSMUST00000108783	ENSMUSG00000000126
novel_circ_0001288	mmu-miR-6909-5	ENSMUST00000000128	ENSMUSG00000000126
novel_circ_0001288	mmu-miR-6909-5	ENSMUST00000108783	ENSMUSG00000000126
novel_circ_0001290	mmu-miR-6909-5	ENSMUST00000000128	ENSMUSG00000000126
novel_circ_0001290	mmu-miR-6909-5	ENSMUST00000108783	ENSMUSG00000000126
mmu_circ_0001376	mmu-miR-6909-5	ENSMUST00000000128	ENSMUSG00000000126
mmu_circ_0001376	mmu-miR-6909-5	ENSMUST00000108783	ENSMUSG00000000126
mmu_circ_0001387	mmu-miR-6909-5	ENSMUST00000000128	ENSMUSG00000000126
mmu_circ_0001387	mmu-miR-6909-5	ENSMUST00000108783	ENSMUSG00000000126
mmu_circ_0001350	mmu-miR-6909-5	ENSMUST00000000128	ENSMUSG00000000126
mmu_circ_0001350	mmu-miR-6909-5	ENSMUST00000108783	ENSMUSG00000000126
mmu_circ_0001283	mmu-miR-6909-5	ENSMUST00000000128	ENSMUSG00000000126
mmu_circ_0001283	mmu-miR-6909-5	ENSMUST00000108783	ENSMUSG00000000126
mmu_circ_0001634	mmu-miR-6909-5	ENSMUST00000000128	ENSMUSG00000000126
mmu_circ_0001634	mmu-miR-6909-5	ENSMUST00000108783	ENSMUSG00000000126
mmu_circ_0001528	mmu-miR-6909-5	ENSMUST00000000128	ENSMUSG00000000126
mmu_circ_0001528	mmu-miR-6909-5	ENSMUST00000108783	ENSMUSG00000000126
mmu_circ_0001500	mmu-miR-6909-5	ENSMUST00000000128	ENSMUSG00000000126
mmu_circ_0001500	mmu-miR-6909-5	ENSMUST00000108783	ENSMUSG00000000126
mmu_circ_0001732	mmu-miR-6909-5	ENSMUST00000000128	ENSMUSG00000000126
mmu_circ_0001732	mmu-miR-6909-5	ENSMUST00000108783	ENSMUSG00000000126
mmu_circ_0001704	mmu-miR-6909-5	ENSMUST00000000128	ENSMUSG00000000126
mmu_circ_0001704	mmu-miR-6909-5	ENSMUST00000108783	ENSMUSG00000000126
mmu_circ_0000271	mmu-miR-6909-5	ENSMUST00000000128	ENSMUSG00000000126
mmu_circ_0000271	mmu-miR-6909-5	ENSMUST00000108783	ENSMUSG00000000126
mmu_circ_0000215	mmu-miR-6909-5	ENSMUST00000000128	ENSMUSG00000000126
mmu_circ_0000215	mmu-miR-6909-5	ENSMUST00000108783	ENSMUSG00000000126
mmu_circ_0000148	mmu-miR-6909-5	ENSMUST00000000128	ENSMUSG00000000126
mmu_circ_0000148	mmu-miR-6909-5	ENSMUST00000108783	ENSMUSG00000000126
mmu_circ_0000149	mmu-miR-6909-5	ENSMUST00000000128	ENSMUSG00000000126
mmu_circ_0000149	mmu-miR-6909-5	ENSMUST00000108783	ENSMUSG00000000126
mmu_circ_0000172	mmu-miR-6909-5	ENSMUST00000000128	ENSMUSG00000000126
mmu_circ_0000172	mmu-miR-6909-5	ENSMUST00000108783	ENSMUSG00000000126
mmu_circ_0000499	mmu-miR-6909-5	ENSMUST00000000128	ENSMUSG00000000126
mmu_circ_0000499	mmu-miR-6909-5	ENSMUST00000108783	ENSMUSG00000000126
mmu_circ_0000500	mmu-miR-6909-5	ENSMUST00000000128	ENSMUSG00000000126
mmu_circ_0000500	mmu-miR-6909-5	ENSMUST00000108783	ENSMUSG00000000126
mmu_circ_0000454	mmu-miR-6909-5	ENSMUST00000000128	ENSMUSG00000000126
mmu_circ_0000454	mmu-miR-6909-5	ENSMUST00000108783	ENSMUSG00000000126
mmu_circ_0000628	mmu-miR-6909-5	ENSMUST00000000128	ENSMUSG00000000126
mmu_circ_0000628	mmu-miR-6909-5	ENSMUST00000108783	ENSMUSG00000000126
mmu_circ_0000539	mmu-miR-6909-5	ENSMUST00000000128	ENSMUSG00000000126
mmu_circ_0000539	mmu-miR-6909-5	ENSMUST00000108783	ENSMUSG00000000126
mmu_circ_0000735	mmu-miR-6909-5	ENSMUST00000000128	ENSMUSG00000000126
mmu_circ_0000735	mmu-miR-6909-5	ENSMUST00000108783	ENSMUSG00000000126
mmu_circ_0000829	mmu-miR-6909-5	ENSMUST00000000128	ENSMUSG00000000126
mmu_circ_0000829	mmu-miR-6909-5	ENSMUST00000108783	ENSMUSG00000000126
mmu_circ_0000712	mmu-miR-6909-5	ENSMUST00000000128	ENSMUSG00000000126
mmu_circ_0000712	mmu-miR-6909-5	ENSMUST00000108783	ENSMUSG00000000126

mmu_circ_0000969	mmu-miR-6909-5	ENSMUST00000000128	ENSMUSG00000000126
mmu_circ_0000969	mmu-miR-6909-5	ENSMUST00000108783	ENSMUSG00000000126
mmu_circ_0000884	mmu-miR-6909-5	ENSMUST00000000128	ENSMUSG00000000126
mmu_circ_0000884	mmu-miR-6909-5	ENSMUST00000108783	ENSMUSG00000000126
mmu_circ_0000886	mmu-miR-6909-5	ENSMUST00000000128	ENSMUSG00000000126
mmu_circ_0000886	mmu-miR-6909-5	ENSMUST00000108783	ENSMUSG00000000126
mmu_circ_0000887	mmu-miR-6909-5	ENSMUST00000000128	ENSMUSG00000000126
mmu_circ_0000887	mmu-miR-6909-5	ENSMUST00000108783	ENSMUSG00000000126
novel_circ_0001579	mmu-miR-6909-5	ENSMUST00000000128	ENSMUSG00000000126
novel_circ_0001579	mmu-miR-6909-5	ENSMUST00000108783	ENSMUSG00000000126
novel_circ_0001842	mmu-miR-6909-5	ENSMUST00000000128	ENSMUSG00000000126
novel_circ_0001842	mmu-miR-6909-5	ENSMUST00000108783	ENSMUSG00000000126
novel_circ_0001946	mmu-miR-6909-5	ENSMUST00000000128	ENSMUSG00000000126
novel_circ_0001946	mmu-miR-6909-5	ENSMUST00000108783	ENSMUSG00000000126
novel_circ_0001946	mmu-miR-6909-5	ENSMUST00000000128	ENSMUSG00000000126
novel_circ_0001946	mmu-miR-6909-5	ENSMUST00000108783	ENSMUSG00000000126
novel_circ_0001947	mmu-miR-6909-5	ENSMUST00000000128	ENSMUSG00000000126
novel_circ_0001947	mmu-miR-6909-5	ENSMUST00000108783	ENSMUSG00000000126
novel_circ_0002481	mmu-miR-6909-5	ENSMUST00000000128	ENSMUSG00000000126
novel_circ_0002481	mmu-miR-6909-5	ENSMUST00000108783	ENSMUSG00000000126
novel_circ_0002482	mmu-miR-6909-5	ENSMUST00000000128	ENSMUSG00000000126
novel_circ_0002482	mmu-miR-6909-5	ENSMUST00000108783	ENSMUSG00000000126
novel_circ_0002871	mmu-miR-6909-5	ENSMUST00000000128	ENSMUSG00000000126
novel_circ_0002871	mmu-miR-6909-5	ENSMUST00000108783	ENSMUSG00000000126
novel_circ_0002920	mmu-miR-6909-5	ENSMUST00000000128	ENSMUSG00000000126
novel_circ_0002920	mmu-miR-6909-5	ENSMUST00000108783	ENSMUSG00000000126
novel_circ_0002927	mmu-miR-6909-5	ENSMUST00000000128	ENSMUSG00000000126
novel_circ_0002927	mmu-miR-6909-5	ENSMUST00000108783	ENSMUSG00000000126
novel_circ_0003036	mmu-miR-6909-5	ENSMUST00000000128	ENSMUSG00000000126
novel_circ_0003036	mmu-miR-6909-5	ENSMUST00000108783	ENSMUSG00000000126
novel_circ_0003057	mmu-miR-6909-5	ENSMUST00000000128	ENSMUSG00000000126
novel_circ_0003057	mmu-miR-6909-5	ENSMUST00000108783	ENSMUSG00000000126
novel_circ_0003057	mmu-miR-6909-5	ENSMUST00000000128	ENSMUSG00000000126
novel_circ_0003057	mmu-miR-6909-5	ENSMUST00000108783	ENSMUSG00000000126
novel_circ_0000047	mmu-miR-6909-5	ENSMUST00000000128	ENSMUSG00000000126
novel_circ_0000047	mmu-miR-6909-5	ENSMUST00000108783	ENSMUSG00000000126
novel_circ_0001049	mmu-miR-6909-5	ENSMUST00000000128	ENSMUSG00000000126
novel_circ_0001049	mmu-miR-6909-5	ENSMUST00000108783	ENSMUSG00000000126
novel_circ_0000819	mmu-miR-6909-5	ENSMUST00000000128	ENSMUSG00000000126
novel_circ_0000819	mmu-miR-6909-5	ENSMUST00000108783	ENSMUSG00000000126
novel_circ_0001177	mmu-miR-6909-5	ENSMUST00000000128	ENSMUSG00000000126
novel_circ_0001177	mmu-miR-6909-5	ENSMUST00000108783	ENSMUSG00000000126
novel_circ_0001179	mmu-miR-6909-5	ENSMUST00000000128	ENSMUSG00000000126
novel_circ_0001179	mmu-miR-6909-5	ENSMUST00000108783	ENSMUSG00000000126
novel_circ_0001190	mmu-miR-6909-5	ENSMUST00000000128	ENSMUSG00000000126
novel_circ_0001190	mmu-miR-6909-5	ENSMUST00000108783	ENSMUSG00000000126
novel_circ_0001286	mmu-miR-6932-5	ENSMUST00000073554	ENSMUSG00000062329
novel_circ_0001287	mmu-miR-6932-5	ENSMUST00000073554	ENSMUSG00000062329
novel_circ_0001287	mmu-miR-6932-5	ENSMUST00000073554	ENSMUSG00000062329
novel_circ_0001309	mmu-miR-6932-5	ENSMUST00000073554	ENSMUSG00000062329
novel_circ_0001438	mmu-miR-6932-5	ENSMUST00000073554	ENSMUSG00000062329
novel_circ_0001543	mmu-miR-6932-5	ENSMUST00000073554	ENSMUSG00000062329
mmu_circ_0000097	mmu-miR-6932-5	ENSMUST00000073554	ENSMUSG00000062329
mmu_circ_0000052	mmu-miR-6932-5	ENSMUST00000073554	ENSMUSG00000062329
mmu_circ_0001123	mmu-miR-6932-5	ENSMUST00000073554	ENSMUSG00000062329
mmu_circ_0001145	mmu-miR-6932-5	ENSMUST00000073554	ENSMUSG00000062329
mmu_circ_0001031	mmu-miR-6932-5	ENSMUST00000073554	ENSMUSG00000062329
mmu_circ_0001033	mmu-miR-6932-5	ENSMUST00000073554	ENSMUSG00000062329

mmu_circ_0001376	mmu-miR-6932-5	ENSMUST00000073554	ENSMUSG00000062329
mmu_circ_0001385	mmu-miR-6932-5	ENSMUST00000073554	ENSMUSG00000062329
mmu_circ_0001385	mmu-miR-6932-5	ENSMUST00000073554	ENSMUSG00000062329
mmu_circ_0001387	mmu-miR-6932-5	ENSMUST00000073554	ENSMUSG00000062329
mmu_circ_0001331	mmu-miR-6932-5	ENSMUST00000073554	ENSMUSG00000062329
mmu_circ_0001454	mmu-miR-6932-5	ENSMUST00000073554	ENSMUSG00000062329
mmu_circ_0001499	mmu-miR-6932-5	ENSMUST00000073554	ENSMUSG00000062329
mmu_circ_0001500	mmu-miR-6932-5	ENSMUST00000073554	ENSMUSG00000062329
mmu_circ_0001775	mmu-miR-6932-5	ENSMUST00000073554	ENSMUSG00000062329
mmu_circ_0000257	mmu-miR-6932-5	ENSMUST00000073554	ENSMUSG00000062329
mmu_circ_0000257	mmu-miR-6932-5	ENSMUST00000073554	ENSMUSG00000062329
mmu_circ_0000220	mmu-miR-6932-5	ENSMUST00000073554	ENSMUSG00000062329
mmu_circ_0000181	mmu-miR-6932-5	ENSMUST00000073554	ENSMUSG00000062329
mmu_circ_0000499	mmu-miR-6932-5	ENSMUST00000073554	ENSMUSG00000062329
mmu_circ_0000371	mmu-miR-6932-5	ENSMUST00000073554	ENSMUSG00000062329
mmu_circ_0000625	mmu-miR-6932-5	ENSMUST00000073554	ENSMUSG00000062329
mmu_circ_0000530	mmu-miR-6932-5	ENSMUST00000073554	ENSMUSG00000062329
mmu_circ_0000831	mmu-miR-6932-5	ENSMUST00000073554	ENSMUSG00000062329
mmu_circ_0000711	mmu-miR-6932-5	ENSMUST00000073554	ENSMUSG00000062329
mmu_circ_0000950	mmu-miR-6932-5	ENSMUST00000073554	ENSMUSG00000062329
mmu_circ_0000969	mmu-miR-6932-5	ENSMUST00000073554	ENSMUSG00000062329
mmu_circ_0000884	mmu-miR-6932-5	ENSMUST00000073554	ENSMUSG00000062329
mmu_circ_0000886	mmu-miR-6932-5	ENSMUST00000073554	ENSMUSG00000062329
mmu_circ_0000887	mmu-miR-6932-5	ENSMUST00000073554	ENSMUSG00000062329
mmu_circ_0000905	mmu-miR-6932-5	ENSMUST00000073554	ENSMUSG00000062329
novel_circ_0001551	mmu-miR-6932-5	ENSMUST00000073554	ENSMUSG00000062329
novel_circ_0001625	mmu-miR-6932-5	ENSMUST00000073554	ENSMUSG00000062329
novel_circ_0002217	mmu-miR-6932-5	ENSMUST00000073554	ENSMUSG00000062329
novel_circ_0001782	mmu-miR-6932-5	ENSMUST00000073554	ENSMUSG00000062329
novel_circ_0001783	mmu-miR-6932-5	ENSMUST00000073554	ENSMUSG00000062329
novel_circ_0001809	mmu-miR-6932-5	ENSMUST00000073554	ENSMUSG00000062329
novel_circ_0001809	mmu-miR-6932-5	ENSMUST00000073554	ENSMUSG00000062329
novel_circ_0001852	mmu-miR-6932-5	ENSMUST00000073554	ENSMUSG00000062329
novel_circ_0001852	mmu-miR-6932-5	ENSMUST00000073554	ENSMUSG00000062329
novel_circ_0001947	mmu-miR-6932-5	ENSMUST00000073554	ENSMUSG00000062329
novel_circ_0001948	mmu-miR-6932-5	ENSMUST00000073554	ENSMUSG00000062329
novel_circ_0002038	mmu-miR-6932-5	ENSMUST00000073554	ENSMUSG00000062329
novel_circ_0002584	mmu-miR-6932-5	ENSMUST00000073554	ENSMUSG00000062329
novel_circ_0002327	mmu-miR-6932-5	ENSMUST00000073554	ENSMUSG00000062329
novel_circ_0002861	mmu-miR-6932-5	ENSMUST00000073554	ENSMUSG00000062329
novel_circ_0002870	mmu-miR-6932-5	ENSMUST00000073554	ENSMUSG00000062329
novel_circ_0002920	mmu-miR-6932-5	ENSMUST00000073554	ENSMUSG00000062329
novel_circ_0002790	mmu-miR-6932-5	ENSMUST00000073554	ENSMUSG00000062329
novel_circ_0003191	mmu-miR-6932-5	ENSMUST00000073554	ENSMUSG00000062329
novel_circ_0003335	mmu-miR-6932-5	ENSMUST00000073554	ENSMUSG00000062329
novel_circ_0003029	mmu-miR-6932-5	ENSMUST00000073554	ENSMUSG00000062329
novel_circ_0003060	mmu-miR-6932-5	ENSMUST00000073554	ENSMUSG00000062329
novel_circ_0003452	mmu-miR-6932-5	ENSMUST00000073554	ENSMUSG00000062329
novel_circ_0000178	mmu-miR-6932-5	ENSMUST00000073554	ENSMUSG00000062329
novel_circ_0000132	mmu-miR-6932-5	ENSMUST00000073554	ENSMUSG00000062329
novel_circ_0000647	mmu-miR-6932-5	ENSMUST00000073554	ENSMUSG00000062329
novel_circ_0000748	mmu-miR-6932-5	ENSMUST00000073554	ENSMUSG00000062329
novel_circ_0000462	mmu-miR-6932-5	ENSMUST00000073554	ENSMUSG00000062329
novel_circ_0000468	mmu-miR-6932-5	ENSMUST00000073554	ENSMUSG00000062329
novel_circ_0000605	mmu-miR-6932-5	ENSMUST00000073554	ENSMUSG00000062329
novel_circ_0000606	mmu-miR-6932-5	ENSMUST00000073554	ENSMUSG00000062329
novel_circ_0001048	mmu-miR-6932-5	ENSMUST00000073554	ENSMUSG00000062329
novel_circ_0000902	mmu-miR-6932-5	ENSMUST00000073554	ENSMUSG00000062329

novel_circ_0000903	mmu-miR-6932-5	ENSMUST00000073554	ENSMUSG00000062329
novel_circ_0001194	mmu-miR-6932-5	ENSMUST00000073554	ENSMUSG00000062329
novel_circ_0001236	mmu-miR-6966-3	ENSMUST00000000128	ENSMUSG00000000126
novel_circ_0001236	mmu-miR-6966-3	ENSMUST00000108783	ENSMUSG00000000126
novel_circ_0001070	mmu-miR-6966-3	ENSMUST00000000128	ENSMUSG00000000126
novel_circ_0001070	mmu-miR-6966-3	ENSMUST00000108783	ENSMUSG00000000126
novel_circ_0001127	mmu-miR-6966-3	ENSMUST00000000128	ENSMUSG00000000126
novel_circ_0001127	mmu-miR-6966-3	ENSMUST00000108783	ENSMUSG00000000126
novel_circ_0001433	mmu-miR-6966-3	ENSMUST00000000128	ENSMUSG00000000126
novel_circ_0001433	mmu-miR-6966-3	ENSMUST00000108783	ENSMUSG00000000126
mmu_circ_0000028	mmu-miR-6966-3	ENSMUST00000000128	ENSMUSG00000000126
mmu_circ_0000028	mmu-miR-6966-3	ENSMUST00000108783	ENSMUSG00000000126
mmu_circ_0000046	mmu-miR-6966-3	ENSMUST00000000128	ENSMUSG00000000126
mmu_circ_0000046	mmu-miR-6966-3	ENSMUST00000108783	ENSMUSG00000000126
mmu_circ_0001123	mmu-miR-6966-3	ENSMUST00000000128	ENSMUSG00000000126
mmu_circ_0001123	mmu-miR-6966-3	ENSMUST00000108783	ENSMUSG00000000126
mmu_circ_0001076	mmu-miR-6966-3	ENSMUST00000000128	ENSMUSG00000000126
mmu_circ_0001076	mmu-miR-6966-3	ENSMUST00000108783	ENSMUSG00000000126
mmu_circ_0001077	mmu-miR-6966-3	ENSMUST00000000128	ENSMUSG00000000126
mmu_circ_0001077	mmu-miR-6966-3	ENSMUST00000108783	ENSMUSG00000000126
mmu_circ_0001017	mmu-miR-6966-3	ENSMUST00000000128	ENSMUSG00000000126
mmu_circ_0001017	mmu-miR-6966-3	ENSMUST00000108783	ENSMUSG00000000126
mmu_circ_0001387	mmu-miR-6966-3	ENSMUST00000000128	ENSMUSG00000000126
mmu_circ_0001387	mmu-miR-6966-3	ENSMUST00000108783	ENSMUSG00000000126
mmu_circ_0001421	mmu-miR-6966-3	ENSMUST00000000128	ENSMUSG00000000126
mmu_circ_0001421	mmu-miR-6966-3	ENSMUST00000108783	ENSMUSG00000000126
mmu_circ_0001329	mmu-miR-6966-3	ENSMUST00000000128	ENSMUSG00000000126
mmu_circ_0001329	mmu-miR-6966-3	ENSMUST00000108783	ENSMUSG00000000126
mmu_circ_0001333	mmu-miR-6966-3	ENSMUST00000000128	ENSMUSG00000000126
mmu_circ_0001333	mmu-miR-6966-3	ENSMUST00000108783	ENSMUSG00000000126
mmu_circ_0001226	mmu-miR-6966-3	ENSMUST00000000128	ENSMUSG00000000126
mmu_circ_0001226	mmu-miR-6966-3	ENSMUST00000108783	ENSMUSG00000000126
mmu_circ_0001516	mmu-miR-6966-3	ENSMUST00000000128	ENSMUSG00000000126
mmu_circ_0001516	mmu-miR-6966-3	ENSMUST00000108783	ENSMUSG00000000126
mmu_circ_0001528	mmu-miR-6966-3	ENSMUST00000000128	ENSMUSG00000000126
mmu_circ_0001528	mmu-miR-6966-3	ENSMUST00000108783	ENSMUSG00000000126
mmu_circ_0001454	mmu-miR-6966-3	ENSMUST00000000128	ENSMUSG00000000126
mmu_circ_0001454	mmu-miR-6966-3	ENSMUST00000108783	ENSMUSG00000000126
mmu_circ_0001500	mmu-miR-6966-3	ENSMUST00000000128	ENSMUSG00000000126
mmu_circ_0001500	mmu-miR-6966-3	ENSMUST00000108783	ENSMUSG00000000126
mmu_circ_0001827	mmu-miR-6966-3	ENSMUST00000000128	ENSMUSG00000000126
mmu_circ_0001827	mmu-miR-6966-3	ENSMUST00000108783	ENSMUSG00000000126
mmu_circ_0001688	mmu-miR-6966-3	ENSMUST00000000128	ENSMUSG00000000126
mmu_circ_0001688	mmu-miR-6966-3	ENSMUST00000108783	ENSMUSG00000000126
mmu_circ_0000325	mmu-miR-6966-3	ENSMUST00000000128	ENSMUSG00000000126
mmu_circ_0000325	mmu-miR-6966-3	ENSMUST00000108783	ENSMUSG00000000126
mmu_circ_0000245	mmu-miR-6966-3	ENSMUST00000000128	ENSMUSG00000000126
mmu_circ_0000245	mmu-miR-6966-3	ENSMUST00000108783	ENSMUSG00000000126
mmu_circ_0000281	mmu-miR-6966-3	ENSMUST00000000128	ENSMUSG00000000126
mmu_circ_0000281	mmu-miR-6966-3	ENSMUST00000108783	ENSMUSG00000000126
mmu_circ_0000282	mmu-miR-6966-3	ENSMUST00000000128	ENSMUSG00000000126
mmu_circ_0000282	mmu-miR-6966-3	ENSMUST00000108783	ENSMUSG00000000126
mmu_circ_0000295	mmu-miR-6966-3	ENSMUST00000000128	ENSMUSG00000000126
mmu_circ_0000295	mmu-miR-6966-3	ENSMUST00000108783	ENSMUSG00000000126
mmu_circ_0000234	mmu-miR-6966-3	ENSMUST00000000128	ENSMUSG00000000126
mmu_circ_0000234	mmu-miR-6966-3	ENSMUST00000108783	ENSMUSG00000000126
mmu_circ_0000172	mmu-miR-6966-3	ENSMUST00000000128	ENSMUSG00000000126
mmu_circ_0000172	mmu-miR-6966-3	ENSMUST00000108783	ENSMUSG00000000126

mmu_circ_0000499	mmu-miR-6966-3	ENSMUST00000000128	ENSMUSG00000000126
mmu_circ_0000499	mmu-miR-6966-3	ENSMUST00000108783	ENSMUSG00000000126
mmu_circ_0000448	mmu-miR-6966-3	ENSMUST00000000128	ENSMUSG00000000126
mmu_circ_0000448	mmu-miR-6966-3	ENSMUST00000108783	ENSMUSG00000000126
mmu_circ_0000454	mmu-miR-6966-3	ENSMUST00000000128	ENSMUSG00000000126
mmu_circ_0000454	mmu-miR-6966-3	ENSMUST00000108783	ENSMUSG00000000126
mmu_circ_0000649	mmu-miR-6966-3	ENSMUST00000000128	ENSMUSG00000000126
mmu_circ_0000649	mmu-miR-6966-3	ENSMUST00000108783	ENSMUSG00000000126
mmu_circ_0000530	mmu-miR-6966-3	ENSMUST00000000128	ENSMUSG00000000126
mmu_circ_0000530	mmu-miR-6966-3	ENSMUST00000108783	ENSMUSG00000000126
mmu_circ_0000554	mmu-miR-6966-3	ENSMUST00000000128	ENSMUSG00000000126
mmu_circ_0000554	mmu-miR-6966-3	ENSMUST00000108783	ENSMUSG00000000126
mmu_circ_0000812	mmu-miR-6966-3	ENSMUST00000000128	ENSMUSG00000000126
mmu_circ_0000812	mmu-miR-6966-3	ENSMUST00000108783	ENSMUSG00000000126
mmu_circ_0000829	mmu-miR-6966-3	ENSMUST00000000128	ENSMUSG00000000126
mmu_circ_0000829	mmu-miR-6966-3	ENSMUST00000108783	ENSMUSG00000000126
mmu_circ_0000831	mmu-miR-6966-3	ENSMUST00000000128	ENSMUSG00000000126
mmu_circ_0000831	mmu-miR-6966-3	ENSMUST00000108783	ENSMUSG00000000126
mmu_circ_0000668	mmu-miR-6966-3	ENSMUST00000000128	ENSMUSG00000000126
mmu_circ_0000668	mmu-miR-6966-3	ENSMUST00000108783	ENSMUSG00000000126
mmu_circ_0000688	mmu-miR-6966-3	ENSMUST00000000128	ENSMUSG00000000126
mmu_circ_0000688	mmu-miR-6966-3	ENSMUST00000108783	ENSMUSG00000000126
mmu_circ_0000711	mmu-miR-6966-3	ENSMUST00000000128	ENSMUSG00000000126
mmu_circ_0000711	mmu-miR-6966-3	ENSMUST00000108783	ENSMUSG00000000126
mmu_circ_0000714	mmu-miR-6966-3	ENSMUST00000000128	ENSMUSG00000000126
mmu_circ_0000714	mmu-miR-6966-3	ENSMUST00000108783	ENSMUSG00000000126
mmu_circ_0000950	mmu-miR-6966-3	ENSMUST00000000128	ENSMUSG00000000126
mmu_circ_0000950	mmu-miR-6966-3	ENSMUST00000108783	ENSMUSG00000000126
mmu_circ_0000918	mmu-miR-6966-3	ENSMUST00000000128	ENSMUSG00000000126
mmu_circ_0000918	mmu-miR-6966-3	ENSMUST00000108783	ENSMUSG00000000126
mmu_circ_0000919	mmu-miR-6966-3	ENSMUST00000000128	ENSMUSG00000000126
mmu_circ_0000919	mmu-miR-6966-3	ENSMUST00000108783	ENSMUSG00000000126
mmu_circ_0000921	mmu-miR-6966-3	ENSMUST00000000128	ENSMUSG00000000126
mmu_circ_0000921	mmu-miR-6966-3	ENSMUST00000108783	ENSMUSG00000000126
mmu_circ_0000969	mmu-miR-6966-3	ENSMUST00000000128	ENSMUSG00000000126
mmu_circ_0000969	mmu-miR-6966-3	ENSMUST00000108783	ENSMUSG00000000126
mmu_circ_0000844	mmu-miR-6966-3	ENSMUST00000000128	ENSMUSG00000000126
mmu_circ_0000844	mmu-miR-6966-3	ENSMUST00000108783	ENSMUSG00000000126
mmu_circ_0000844	mmu-miR-6966-3	ENSMUST00000000128	ENSMUSG00000000126
mmu_circ_0000844	mmu-miR-6966-3	ENSMUST00000108783	ENSMUSG00000000126
mmu_circ_0000884	mmu-miR-6966-3	ENSMUST00000000128	ENSMUSG00000000126
mmu_circ_0000884	mmu-miR-6966-3	ENSMUST00000108783	ENSMUSG00000000126
mmu_circ_0000886	mmu-miR-6966-3	ENSMUST00000000128	ENSMUSG00000000126
mmu_circ_0000886	mmu-miR-6966-3	ENSMUST00000108783	ENSMUSG00000000126
mmu_circ_0000898	mmu-miR-6966-3	ENSMUST00000000128	ENSMUSG00000000126
mmu_circ_0000898	mmu-miR-6966-3	ENSMUST00000108783	ENSMUSG00000000126
mmu_circ_0000906	mmu-miR-6966-3	ENSMUST00000000128	ENSMUSG00000000126
mmu_circ_0000906	mmu-miR-6966-3	ENSMUST00000108783	ENSMUSG00000000126
mmu_circ_0000907	mmu-miR-6966-3	ENSMUST00000000128	ENSMUSG00000000126
mmu_circ_0000907	mmu-miR-6966-3	ENSMUST00000108783	ENSMUSG00000000126
mmu_circ_0000908	mmu-miR-6966-3	ENSMUST00000000128	ENSMUSG00000000126
mmu_circ_0000908	mmu-miR-6966-3	ENSMUST00000108783	ENSMUSG00000000126
novel_circ_0001570	mmu-miR-6966-3	ENSMUST00000000128	ENSMUSG00000000126
novel_circ_0001570	mmu-miR-6966-3	ENSMUST00000108783	ENSMUSG00000000126
novel_circ_0001672	mmu-miR-6966-3	ENSMUST00000000128	ENSMUSG00000000126
novel_circ_0001672	mmu-miR-6966-3	ENSMUST00000108783	ENSMUSG00000000126
novel_circ_0001676	mmu-miR-6966-3	ENSMUST00000000128	ENSMUSG00000000126
novel_circ_0001676	mmu-miR-6966-3	ENSMUST00000108783	ENSMUSG00000000126

novel_circ_0001676	mmu-miR-6966-3	ENSMUST00000000128	ENSMUSG00000000126
novel_circ_0001676	mmu-miR-6966-3	ENSMUST00000108783	ENSMUSG00000000126
novel_circ_0001680	mmu-miR-6966-3	ENSMUST00000000128	ENSMUSG00000000126
novel_circ_0001680	mmu-miR-6966-3	ENSMUST00000108783	ENSMUSG00000000126
novel_circ_0001708	mmu-miR-6966-3	ENSMUST00000000128	ENSMUSG00000000126
novel_circ_0001708	mmu-miR-6966-3	ENSMUST00000108783	ENSMUSG00000000126
novel_circ_0002075	mmu-miR-6966-3	ENSMUST00000000128	ENSMUSG00000000126
novel_circ_0002075	mmu-miR-6966-3	ENSMUST00000108783	ENSMUSG00000000126
novel_circ_0002096	mmu-miR-6966-3	ENSMUST00000000128	ENSMUSG00000000126
novel_circ_0002096	mmu-miR-6966-3	ENSMUST00000108783	ENSMUSG00000000126
novel_circ_0002106	mmu-miR-6966-3	ENSMUST00000000128	ENSMUSG00000000126
novel_circ_0002106	mmu-miR-6966-3	ENSMUST00000108783	ENSMUSG00000000126
novel_circ_0002213	mmu-miR-6966-3	ENSMUST00000000128	ENSMUSG00000000126
novel_circ_0002213	mmu-miR-6966-3	ENSMUST00000108783	ENSMUSG00000000126
novel_circ_0002213	mmu-miR-6966-3	ENSMUST00000000128	ENSMUSG00000000126
novel_circ_0002213	mmu-miR-6966-3	ENSMUST00000108783	ENSMUSG00000000126
novel_circ_0001751	mmu-miR-6966-3	ENSMUST00000000128	ENSMUSG00000000126
novel_circ_0001751	mmu-miR-6966-3	ENSMUST00000108783	ENSMUSG00000000126
novel_circ_0001769	mmu-miR-6966-3	ENSMUST00000000128	ENSMUSG00000000126
novel_circ_0001769	mmu-miR-6966-3	ENSMUST00000108783	ENSMUSG00000000126
novel_circ_0001770	mmu-miR-6966-3	ENSMUST00000000128	ENSMUSG00000000126
novel_circ_0001770	mmu-miR-6966-3	ENSMUST00000108783	ENSMUSG00000000126
novel_circ_0001782	mmu-miR-6966-3	ENSMUST00000000128	ENSMUSG00000000126
novel_circ_0001782	mmu-miR-6966-3	ENSMUST00000108783	ENSMUSG00000000126
novel_circ_0001805	mmu-miR-6966-3	ENSMUST00000000128	ENSMUSG00000000126
novel_circ_0001805	mmu-miR-6966-3	ENSMUST00000108783	ENSMUSG00000000126
novel_circ_0001885	mmu-miR-6966-3	ENSMUST00000000128	ENSMUSG00000000126
novel_circ_0001885	mmu-miR-6966-3	ENSMUST00000108783	ENSMUSG00000000126
novel_circ_0001912	mmu-miR-6966-3	ENSMUST00000000128	ENSMUSG00000000126
novel_circ_0001912	mmu-miR-6966-3	ENSMUST00000108783	ENSMUSG00000000126
novel_circ_0001935	mmu-miR-6966-3	ENSMUST00000000128	ENSMUSG00000000126
novel_circ_0001935	mmu-miR-6966-3	ENSMUST00000108783	ENSMUSG00000000126
novel_circ_0002050	mmu-miR-6966-3	ENSMUST00000000128	ENSMUSG00000000126
novel_circ_0002050	mmu-miR-6966-3	ENSMUST00000108783	ENSMUSG00000000126
novel_circ_0002051	mmu-miR-6966-3	ENSMUST00000000128	ENSMUSG00000000126
novel_circ_0002051	mmu-miR-6966-3	ENSMUST00000108783	ENSMUSG00000000126
novel_circ_0002403	mmu-miR-6966-3	ENSMUST00000000128	ENSMUSG00000000126
novel_circ_0002403	mmu-miR-6966-3	ENSMUST00000108783	ENSMUSG00000000126
novel_circ_0002403	mmu-miR-6966-3	ENSMUST00000000128	ENSMUSG00000000126
novel_circ_0002403	mmu-miR-6966-3	ENSMUST00000108783	ENSMUSG00000000126
novel_circ_0002447	mmu-miR-6966-3	ENSMUST00000000128	ENSMUSG00000000126
novel_circ_0002447	mmu-miR-6966-3	ENSMUST00000108783	ENSMUSG00000000126
novel_circ_0002448	mmu-miR-6966-3	ENSMUST00000000128	ENSMUSG00000000126
novel_circ_0002448	mmu-miR-6966-3	ENSMUST00000108783	ENSMUSG00000000126
novel_circ_0002481	mmu-miR-6966-3	ENSMUST00000000128	ENSMUSG00000000126
novel_circ_0002481	mmu-miR-6966-3	ENSMUST00000108783	ENSMUSG00000000126
novel_circ_0002482	mmu-miR-6966-3	ENSMUST00000000128	ENSMUSG00000000126
novel_circ_0002482	mmu-miR-6966-3	ENSMUST00000108783	ENSMUSG00000000126
novel_circ_0002484	mmu-miR-6966-3	ENSMUST00000000128	ENSMUSG00000000126
novel_circ_0002484	mmu-miR-6966-3	ENSMUST00000108783	ENSMUSG00000000126
novel_circ_0002485	mmu-miR-6966-3	ENSMUST00000000128	ENSMUSG00000000126
novel_circ_0002485	mmu-miR-6966-3	ENSMUST00000108783	ENSMUSG00000000126
novel_circ_0002584	mmu-miR-6966-3	ENSMUST00000000128	ENSMUSG00000000126
novel_circ_0002584	mmu-miR-6966-3	ENSMUST00000108783	ENSMUSG00000000126
novel_circ_0002597	mmu-miR-6966-3	ENSMUST00000000128	ENSMUSG00000000126
novel_circ_0002597	mmu-miR-6966-3	ENSMUST00000108783	ENSMUSG00000000126
novel_circ_0002250	mmu-miR-6966-3	ENSMUST00000000128	ENSMUSG00000000126
novel_circ_0002250	mmu-miR-6966-3	ENSMUST00000108783	ENSMUSG00000000126

novel_circ_0002292	mmu-miR-6966-3	ENSMUST00000000128	ENSMUSG00000000126
novel_circ_0002292	mmu-miR-6966-3	ENSMUST00000108783	ENSMUSG00000000126
novel_circ_0002298	mmu-miR-6966-3	ENSMUST00000000128	ENSMUSG00000000126
novel_circ_0002298	mmu-miR-6966-3	ENSMUST00000108783	ENSMUSG00000000126
novel_circ_0002329	mmu-miR-6966-3	ENSMUST00000000128	ENSMUSG00000000126
novel_circ_0002329	mmu-miR-6966-3	ENSMUST00000108783	ENSMUSG00000000126
novel_circ_0002340	mmu-miR-6966-3	ENSMUST00000000128	ENSMUSG00000000126
novel_circ_0002340	mmu-miR-6966-3	ENSMUST00000108783	ENSMUSG00000000126
novel_circ_0002340	mmu-miR-6966-3	ENSMUST00000000128	ENSMUSG00000000126
novel_circ_0002340	mmu-miR-6966-3	ENSMUST00000108783	ENSMUSG00000000126
novel_circ_0002879	mmu-miR-6966-3	ENSMUST00000000128	ENSMUSG00000000126
novel_circ_0002879	mmu-miR-6966-3	ENSMUST00000108783	ENSMUSG00000000126
novel_circ_0002907	mmu-miR-6966-3	ENSMUST00000000128	ENSMUSG00000000126
novel_circ_0002907	mmu-miR-6966-3	ENSMUST00000108783	ENSMUSG00000000126
novel_circ_0002909	mmu-miR-6966-3	ENSMUST00000000128	ENSMUSG00000000126
novel_circ_0002909	mmu-miR-6966-3	ENSMUST00000108783	ENSMUSG00000000126
novel_circ_0002971	mmu-miR-6966-3	ENSMUST00000000128	ENSMUSG00000000126
novel_circ_0002971	mmu-miR-6966-3	ENSMUST00000108783	ENSMUSG00000000126
novel_circ_0002631	mmu-miR-6966-3	ENSMUST00000000128	ENSMUSG00000000126
novel_circ_0002631	mmu-miR-6966-3	ENSMUST00000108783	ENSMUSG00000000126
novel_circ_0002670	mmu-miR-6966-3	ENSMUST00000000128	ENSMUSG00000000126
novel_circ_0002670	mmu-miR-6966-3	ENSMUST00000108783	ENSMUSG00000000126
novel_circ_0002683	mmu-miR-6966-3	ENSMUST00000000128	ENSMUSG00000000126
novel_circ_0002683	mmu-miR-6966-3	ENSMUST00000108783	ENSMUSG00000000126
novel_circ_0002788	mmu-miR-6966-3	ENSMUST00000000128	ENSMUSG00000000126
novel_circ_0002788	mmu-miR-6966-3	ENSMUST00000108783	ENSMUSG00000000126
novel_circ_0003206	mmu-miR-6966-3	ENSMUST00000000128	ENSMUSG00000000126
novel_circ_0003206	mmu-miR-6966-3	ENSMUST00000108783	ENSMUSG00000000126
novel_circ_0003325	mmu-miR-6966-3	ENSMUST00000000128	ENSMUSG00000000126
novel_circ_0003325	mmu-miR-6966-3	ENSMUST00000108783	ENSMUSG00000000126
novel_circ_0003350	mmu-miR-6966-3	ENSMUST00000000128	ENSMUSG00000000126
novel_circ_0003350	mmu-miR-6966-3	ENSMUST00000108783	ENSMUSG00000000126
novel_circ_0003074	mmu-miR-6966-3	ENSMUST00000000128	ENSMUSG00000000126
novel_circ_0003074	mmu-miR-6966-3	ENSMUST00000108783	ENSMUSG00000000126
novel_circ_0003077	mmu-miR-6966-3	ENSMUST00000000128	ENSMUSG00000000126
novel_circ_0003077	mmu-miR-6966-3	ENSMUST00000108783	ENSMUSG00000000126
novel_circ_0000208	mmu-miR-6966-3	ENSMUST00000000128	ENSMUSG00000000126
novel_circ_0000208	mmu-miR-6966-3	ENSMUST00000108783	ENSMUSG00000000126
novel_circ_0000208	mmu-miR-6966-3	ENSMUST00000000128	ENSMUSG00000000126
novel_circ_0000208	mmu-miR-6966-3	ENSMUST00000108783	ENSMUSG00000000126
novel_circ_0000293	mmu-miR-6966-3	ENSMUST00000000128	ENSMUSG00000000126
novel_circ_0000293	mmu-miR-6966-3	ENSMUST00000108783	ENSMUSG00000000126
novel_circ_0000315	mmu-miR-6966-3	ENSMUST00000000128	ENSMUSG00000000126
novel_circ_0000315	mmu-miR-6966-3	ENSMUST00000108783	ENSMUSG00000000126
novel_circ_0000315	mmu-miR-6966-3	ENSMUST00000000128	ENSMUSG00000000126
novel_circ_0000315	mmu-miR-6966-3	ENSMUST00000108783	ENSMUSG00000000126
novel_circ_0000365	mmu-miR-6966-3	ENSMUST00000000128	ENSMUSG00000000126
novel_circ_0000365	mmu-miR-6966-3	ENSMUST00000108783	ENSMUSG00000000126
novel_circ_0000017	mmu-miR-6966-3	ENSMUST00000000128	ENSMUSG00000000126
novel_circ_0000017	mmu-miR-6966-3	ENSMUST00000108783	ENSMUSG00000000126
novel_circ_0000065	mmu-miR-6966-3	ENSMUST00000000128	ENSMUSG00000000126
novel_circ_0000065	mmu-miR-6966-3	ENSMUST00000108783	ENSMUSG00000000126
novel_circ_0000160	mmu-miR-6966-3	ENSMUST00000000128	ENSMUSG00000000126
novel_circ_0000160	mmu-miR-6966-3	ENSMUST00000108783	ENSMUSG00000000126
novel_circ_0000625	mmu-miR-6966-3	ENSMUST00000000128	ENSMUSG00000000126
novel_circ_0000625	mmu-miR-6966-3	ENSMUST00000108783	ENSMUSG00000000126
novel_circ_0000642	mmu-miR-6966-3	ENSMUST00000000128	ENSMUSG00000000126
novel_circ_0000642	mmu-miR-6966-3	ENSMUST00000108783	ENSMUSG00000000126

novel_circ_0000680	mmu-miR-6966-3	ENSMUST00000000128	ENSMUSG00000000126
novel_circ_0000680	mmu-miR-6966-3	ENSMUST00000108783	ENSMUSG00000000126
novel_circ_0000682	mmu-miR-6966-3	ENSMUST00000000128	ENSMUSG00000000126
novel_circ_0000682	mmu-miR-6966-3	ENSMUST00000108783	ENSMUSG00000000126
novel_circ_0000682	mmu-miR-6966-3	ENSMUST00000000128	ENSMUSG00000000126
novel_circ_0000682	mmu-miR-6966-3	ENSMUST00000108783	ENSMUSG00000000126
novel_circ_0000753	mmu-miR-6966-3	ENSMUST00000000128	ENSMUSG00000000126
novel_circ_0000753	mmu-miR-6966-3	ENSMUST00000108783	ENSMUSG00000000126
novel_circ_0000756	mmu-miR-6966-3	ENSMUST00000000128	ENSMUSG00000000126
novel_circ_0000756	mmu-miR-6966-3	ENSMUST00000108783	ENSMUSG00000000126
novel_circ_0000475	mmu-miR-6966-3	ENSMUST00000000128	ENSMUSG00000000126
novel_circ_0000475	mmu-miR-6966-3	ENSMUST00000108783	ENSMUSG00000000126
novel_circ_0001048	mmu-miR-6966-3	ENSMUST00000000128	ENSMUSG00000000126
novel_circ_0001048	mmu-miR-6966-3	ENSMUST00000108783	ENSMUSG00000000126
novel_circ_0000814	mmu-miR-6966-3	ENSMUST00000000128	ENSMUSG00000000126
novel_circ_0000814	mmu-miR-6966-3	ENSMUST00000108783	ENSMUSG00000000126
novel_circ_0000858	mmu-miR-6966-3	ENSMUST00000000128	ENSMUSG00000000126
novel_circ_0000858	mmu-miR-6966-3	ENSMUST00000108783	ENSMUSG00000000126
novel_circ_0000868	mmu-miR-6966-3	ENSMUST00000000128	ENSMUSG00000000126
novel_circ_0000868	mmu-miR-6966-3	ENSMUST00000108783	ENSMUSG00000000126
novel_circ_0001213	mmu-miR-6966-3	ENSMUST00000000128	ENSMUSG00000000126
novel_circ_0001213	mmu-miR-6966-3	ENSMUST00000108783	ENSMUSG00000000126
novel_circ_0001213	mmu-miR-6966-3	ENSMUST00000000128	ENSMUSG00000000126
novel_circ_0001213	mmu-miR-6966-3	ENSMUST00000108783	ENSMUSG00000000126
novel_circ_0001228	mmu-miR-7015-5	ENSMUST00000073554	ENSMUSG00000062329
novel_circ_0001232	mmu-miR-7015-5	ENSMUST00000073554	ENSMUSG00000062329
novel_circ_0001237	mmu-miR-7015-5	ENSMUST00000073554	ENSMUSG00000062329
novel_circ_0001242	mmu-miR-7015-5	ENSMUST00000073554	ENSMUSG00000062329
novel_circ_0001254	mmu-miR-7015-5	ENSMUST00000073554	ENSMUSG00000062329
novel_circ_0001256	mmu-miR-7015-5	ENSMUST00000073554	ENSMUSG00000062329
novel_circ_0001138	mmu-miR-7015-5	ENSMUST00000073554	ENSMUSG00000062329
novel_circ_0001138	mmu-miR-7015-5	ENSMUST00000073554	ENSMUSG00000062329
novel_circ_0001464	mmu-miR-7015-5	ENSMUST00000073554	ENSMUSG00000062329
novel_circ_0001471	mmu-miR-7015-5	ENSMUST00000073554	ENSMUSG00000062329
novel_circ_0001433	mmu-miR-7015-5	ENSMUST00000073554	ENSMUSG00000062329
mmu_circ_0000075	mmu-miR-7015-5	ENSMUST00000073554	ENSMUSG00000062329
mmu_circ_0000094	mmu-miR-7015-5	ENSMUST00000073554	ENSMUSG00000062329
mmu_circ_0000132	mmu-miR-7015-5	ENSMUST00000073554	ENSMUSG00000062329
mmu_circ_0000133	mmu-miR-7015-5	ENSMUST00000073554	ENSMUSG00000062329
mmu_circ_0000028	mmu-miR-7015-5	ENSMUST00000073554	ENSMUSG00000062329
mmu_circ_0000046	mmu-miR-7015-5	ENSMUST00000073554	ENSMUSG00000062329
mmu_circ_0000048	mmu-miR-7015-5	ENSMUST00000073554	ENSMUSG00000062329
mmu_circ_0000062	mmu-miR-7015-5	ENSMUST00000073554	ENSMUSG00000062329
mmu_circ_0000063	mmu-miR-7015-5	ENSMUST00000073554	ENSMUSG00000062329
mmu_circ_0001076	mmu-miR-7015-5	ENSMUST00000073554	ENSMUSG00000062329
mmu_circ_0001076	mmu-miR-7015-5	ENSMUST00000073554	ENSMUSG00000062329
mmu_circ_0001077	mmu-miR-7015-5	ENSMUST00000073554	ENSMUSG00000062329
mmu_circ_0001077	mmu-miR-7015-5	ENSMUST00000073554	ENSMUSG00000062329
mmu_circ_0001021	mmu-miR-7015-5	ENSMUST00000073554	ENSMUSG00000062329
mmu_circ_0001028	mmu-miR-7015-5	ENSMUST00000073554	ENSMUSG00000062329
mmu_circ_0001033	mmu-miR-7015-5	ENSMUST00000073554	ENSMUSG00000062329
mmu_circ_0001033	mmu-miR-7015-5	ENSMUST00000073554	ENSMUSG00000062329
mmu_circ_0001376	mmu-miR-7015-5	ENSMUST00000073554	ENSMUSG00000062329
mmu_circ_0001387	mmu-miR-7015-5	ENSMUST00000073554	ENSMUSG00000062329
mmu_circ_0001391	mmu-miR-7015-5	ENSMUST00000073554	ENSMUSG00000062329
mmu_circ_0001421	mmu-miR-7015-5	ENSMUST00000073554	ENSMUSG00000062329
mmu_circ_0001421	mmu-miR-7015-5	ENSMUST00000073554	ENSMUSG00000062329
mmu_circ_0001333	mmu-miR-7015-5	ENSMUST00000073554	ENSMUSG00000062329

mmu_circ_0001345	mmu-miR-7015-5	ENSMUST00000073554	ENSMUSG00000062329
mmu_circ_0001283	mmu-miR-7015-5	ENSMUST00000073554	ENSMUSG00000062329
mmu_circ_0001291	mmu-miR-7015-5	ENSMUST00000073554	ENSMUSG00000062329
mmu_circ_0001528	mmu-miR-7015-5	ENSMUST00000073554	ENSMUSG00000062329
mmu_circ_0001454	mmu-miR-7015-5	ENSMUST00000073554	ENSMUSG00000062329
mmu_circ_0001496	mmu-miR-7015-5	ENSMUST00000073554	ENSMUSG00000062329
mmu_circ_0001500	mmu-miR-7015-5	ENSMUST00000073554	ENSMUSG00000062329
mmu_circ_0001794	mmu-miR-7015-5	ENSMUST00000073554	ENSMUSG00000062329
mmu_circ_0001794	mmu-miR-7015-5	ENSMUST00000073554	ENSMUSG00000062329
mmu_circ_0001798	mmu-miR-7015-5	ENSMUST00000073554	ENSMUSG00000062329
mmu_circ_0001799	mmu-miR-7015-5	ENSMUST00000073554	ENSMUSG00000062329
mmu_circ_0001827	mmu-miR-7015-5	ENSMUST00000073554	ENSMUSG00000062329
mmu_circ_0001732	mmu-miR-7015-5	ENSMUST00000073554	ENSMUSG00000062329
mmu_circ_0001688	mmu-miR-7015-5	ENSMUST00000073554	ENSMUSG00000062329
mmu_circ_0000243	mmu-miR-7015-5	ENSMUST00000073554	ENSMUSG00000062329
mmu_circ_0000230	mmu-miR-7015-5	ENSMUST00000073554	ENSMUSG00000062329
mmu_circ_0000231	mmu-miR-7015-5	ENSMUST00000073554	ENSMUSG00000062329
mmu_circ_0000281	mmu-miR-7015-5	ENSMUST00000073554	ENSMUSG00000062329
mmu_circ_0000282	mmu-miR-7015-5	ENSMUST00000073554	ENSMUSG00000062329
mmu_circ_0000432	mmu-miR-7015-5	ENSMUST00000073554	ENSMUSG00000062329
mmu_circ_0000432	mmu-miR-7015-5	ENSMUST00000073554	ENSMUSG00000062329
mmu_circ_0000454	mmu-miR-7015-5	ENSMUST00000073554	ENSMUSG00000062329
mmu_circ_0000454	mmu-miR-7015-5	ENSMUST00000073554	ENSMUSG00000062329
mmu_circ_0000349	mmu-miR-7015-5	ENSMUST00000073554	ENSMUSG00000062329
mmu_circ_0000383	mmu-miR-7015-5	ENSMUST00000073554	ENSMUSG00000062329
mmu_circ_0000399	mmu-miR-7015-5	ENSMUST00000073554	ENSMUSG00000062329
mmu_circ_0000621	mmu-miR-7015-5	ENSMUST00000073554	ENSMUSG00000062329
mmu_circ_0000628	mmu-miR-7015-5	ENSMUST00000073554	ENSMUSG00000062329
mmu_circ_0000504	mmu-miR-7015-5	ENSMUST00000073554	ENSMUSG00000062329
mmu_circ_0000530	mmu-miR-7015-5	ENSMUST00000073554	ENSMUSG00000062329
mmu_circ_0000828	mmu-miR-7015-5	ENSMUST00000073554	ENSMUSG00000062329
mmu_circ_0000829	mmu-miR-7015-5	ENSMUST00000073554	ENSMUSG00000062329
mmu_circ_0000831	mmu-miR-7015-5	ENSMUST00000073554	ENSMUSG00000062329
mmu_circ_0000833	mmu-miR-7015-5	ENSMUST00000073554	ENSMUSG00000062329
mmu_circ_0000681	mmu-miR-7015-5	ENSMUST00000073554	ENSMUSG00000062329
mmu_circ_0000712	mmu-miR-7015-5	ENSMUST00000073554	ENSMUSG00000062329
mmu_circ_0000714	mmu-miR-7015-5	ENSMUST00000073554	ENSMUSG00000062329
mmu_circ_0000950	mmu-miR-7015-5	ENSMUST00000073554	ENSMUSG00000062329
mmu_circ_0000969	mmu-miR-7015-5	ENSMUST00000073554	ENSMUSG00000062329
mmu_circ_0000878	mmu-miR-7015-5	ENSMUST00000073554	ENSMUSG00000062329
mmu_circ_0000906	mmu-miR-7015-5	ENSMUST00000073554	ENSMUSG00000062329
mmu_circ_0000907	mmu-miR-7015-5	ENSMUST00000073554	ENSMUSG00000062329
mmu_circ_0000908	mmu-miR-7015-5	ENSMUST00000073554	ENSMUSG00000062329
mmu_circ_0000912	mmu-miR-7015-5	ENSMUST00000073554	ENSMUSG00000062329
novel_circ_0001573	mmu-miR-7015-5	ENSMUST00000073554	ENSMUSG00000062329
novel_circ_0001603	mmu-miR-7015-5	ENSMUST00000073554	ENSMUSG00000062329
novel_circ_0001603	mmu-miR-7015-5	ENSMUST00000073554	ENSMUSG00000062329
novel_circ_0001619	mmu-miR-7015-5	ENSMUST00000073554	ENSMUSG00000062329
novel_circ_0001619	mmu-miR-7015-5	ENSMUST00000073554	ENSMUSG00000062329
novel_circ_0001672	mmu-miR-7015-5	ENSMUST00000073554	ENSMUSG00000062329
novel_circ_0001713	mmu-miR-7015-5	ENSMUST00000073554	ENSMUSG00000062329
novel_circ_0002145	mmu-miR-7015-5	ENSMUST00000073554	ENSMUSG00000062329
novel_circ_0002145	mmu-miR-7015-5	ENSMUST00000073554	ENSMUSG00000062329
novel_circ_0002164	mmu-miR-7015-5	ENSMUST00000073554	ENSMUSG00000062329
novel_circ_0002214	mmu-miR-7015-5	ENSMUST00000073554	ENSMUSG00000062329
novel_circ_0001752	mmu-miR-7015-5	ENSMUST00000073554	ENSMUSG00000062329
novel_circ_0001842	mmu-miR-7015-5	ENSMUST00000073554	ENSMUSG00000062329
novel_circ_0001903	mmu-miR-7015-5	ENSMUST00000073554	ENSMUSG00000062329

novel_circ_0001903	mmu-miR-7015-5	ENSMUST00000073554	ENSMUSG00000062329
novel_circ_0001964	mmu-miR-7015-5	ENSMUST00000073554	ENSMUSG00000062329
novel_circ_0001972	mmu-miR-7015-5	ENSMUST00000073554	ENSMUSG00000062329
novel_circ_0002014	mmu-miR-7015-5	ENSMUST00000073554	ENSMUSG00000062329
novel_circ_0002027	mmu-miR-7015-5	ENSMUST00000073554	ENSMUSG00000062329
novel_circ_0002027	mmu-miR-7015-5	ENSMUST00000073554	ENSMUSG00000062329
novel_circ_0002050	mmu-miR-7015-5	ENSMUST00000073554	ENSMUSG00000062329
novel_circ_0002434	mmu-miR-7015-5	ENSMUST00000073554	ENSMUSG00000062329
novel_circ_0002434	mmu-miR-7015-5	ENSMUST00000073554	ENSMUSG00000062329
novel_circ_0002447	mmu-miR-7015-5	ENSMUST00000073554	ENSMUSG00000062329
novel_circ_0002448	mmu-miR-7015-5	ENSMUST00000073554	ENSMUSG00000062329
novel_circ_0002478	mmu-miR-7015-5	ENSMUST00000073554	ENSMUSG00000062329
novel_circ_0002493	mmu-miR-7015-5	ENSMUST00000073554	ENSMUSG00000062329
novel_circ_0002507	mmu-miR-7015-5	ENSMUST00000073554	ENSMUSG00000062329
novel_circ_0002507	mmu-miR-7015-5	ENSMUST00000073554	ENSMUSG00000062329
novel_circ_0002510	mmu-miR-7015-5	ENSMUST00000073554	ENSMUSG00000062329
novel_circ_0002510	mmu-miR-7015-5	ENSMUST00000073554	ENSMUSG00000062329
novel_circ_0002511	mmu-miR-7015-5	ENSMUST00000073554	ENSMUSG00000062329
novel_circ_0002511	mmu-miR-7015-5	ENSMUST00000073554	ENSMUSG00000062329
novel_circ_0002582	mmu-miR-7015-5	ENSMUST00000073554	ENSMUSG00000062329
novel_circ_0002618	mmu-miR-7015-5	ENSMUST00000073554	ENSMUSG00000062329
novel_circ_0002618	mmu-miR-7015-5	ENSMUST00000073554	ENSMUSG00000062329
novel_circ_0002336	mmu-miR-7015-5	ENSMUST00000073554	ENSMUSG00000062329
novel_circ_0002352	mmu-miR-7015-5	ENSMUST00000073554	ENSMUSG00000062329
novel_circ_0002352	mmu-miR-7015-5	ENSMUST00000073554	ENSMUSG00000062329
novel_circ_0002360	mmu-miR-7015-5	ENSMUST00000073554	ENSMUSG00000062329
novel_circ_0002362	mmu-miR-7015-5	ENSMUST00000073554	ENSMUSG00000062329
novel_circ_0002362	mmu-miR-7015-5	ENSMUST00000073554	ENSMUSG00000062329
novel_circ_0002363	mmu-miR-7015-5	ENSMUST00000073554	ENSMUSG00000062329
novel_circ_0002364	mmu-miR-7015-5	ENSMUST00000073554	ENSMUSG00000062329
novel_circ_0002914	mmu-miR-7015-5	ENSMUST00000073554	ENSMUSG00000062329
novel_circ_0002927	mmu-miR-7015-5	ENSMUST00000073554	ENSMUSG00000062329
novel_circ_0002933	mmu-miR-7015-5	ENSMUST00000073554	ENSMUSG00000062329
novel_circ_0002939	mmu-miR-7015-5	ENSMUST00000073554	ENSMUSG00000062329
novel_circ_0002998	mmu-miR-7015-5	ENSMUST00000073554	ENSMUSG00000062329
novel_circ_0002998	mmu-miR-7015-5	ENSMUST00000073554	ENSMUSG00000062329
novel_circ_0002631	mmu-miR-7015-5	ENSMUST00000073554	ENSMUSG00000062329
novel_circ_0003029	mmu-miR-7015-5	ENSMUST00000073554	ENSMUSG00000062329
novel_circ_0003036	mmu-miR-7015-5	ENSMUST00000073554	ENSMUSG00000062329
novel_circ_0003047	mmu-miR-7015-5	ENSMUST00000073554	ENSMUSG00000062329
novel_circ_0003083	mmu-miR-7015-5	ENSMUST00000073554	ENSMUSG00000062329
novel_circ_0003119	mmu-miR-7015-5	ENSMUST00000073554	ENSMUSG00000062329
novel_circ_0003490	mmu-miR-7015-5	ENSMUST00000073554	ENSMUSG00000062329
novel_circ_0003503	mmu-miR-7015-5	ENSMUST00000073554	ENSMUSG00000062329
novel_circ_0000208	mmu-miR-7015-5	ENSMUST00000073554	ENSMUSG00000062329
novel_circ_0000211	mmu-miR-7015-5	ENSMUST00000073554	ENSMUSG00000062329
novel_circ_0000239	mmu-miR-7015-5	ENSMUST00000073554	ENSMUSG00000062329
novel_circ_0000310	mmu-miR-7015-5	ENSMUST00000073554	ENSMUSG00000062329
novel_circ_0000396	mmu-miR-7015-5	ENSMUST00000073554	ENSMUSG00000062329
novel_circ_0000441	mmu-miR-7015-5	ENSMUST00000073554	ENSMUSG00000062329
novel_circ_0000021	mmu-miR-7015-5	ENSMUST00000073554	ENSMUSG00000062329
novel_circ_0000029	mmu-miR-7015-5	ENSMUST00000073554	ENSMUSG00000062329
novel_circ_0000051	mmu-miR-7015-5	ENSMUST00000073554	ENSMUSG00000062329
novel_circ_0000051	mmu-miR-7015-5	ENSMUST00000073554	ENSMUSG00000062329
novel_circ_0000083	mmu-miR-7015-5	ENSMUST00000073554	ENSMUSG00000062329
novel_circ_0000083	mmu-miR-7015-5	ENSMUST00000073554	ENSMUSG00000062329
novel_circ_0000100	mmu-miR-7015-5	ENSMUST00000073554	ENSMUSG00000062329
novel_circ_0000116	mmu-miR-7015-5	ENSMUST00000073554	ENSMUSG00000062329

novel_circ_0000116	mmu-miR-7015-5	ENSMUST00000073554	ENSMUSG00000062329
novel_circ_0000694	mmu-miR-7015-5	ENSMUST00000073554	ENSMUSG00000062329
novel_circ_0000694	mmu-miR-7015-5	ENSMUST00000073554	ENSMUSG00000062329
novel_circ_0000695	mmu-miR-7015-5	ENSMUST00000073554	ENSMUSG00000062329
novel_circ_0000695	mmu-miR-7015-5	ENSMUST00000073554	ENSMUSG00000062329
novel_circ_0000703	mmu-miR-7015-5	ENSMUST00000073554	ENSMUSG00000062329
novel_circ_0000771	mmu-miR-7015-5	ENSMUST00000073554	ENSMUSG00000062329
novel_circ_0000525	mmu-miR-7015-5	ENSMUST00000073554	ENSMUSG00000062329
novel_circ_0000938	mmu-miR-7015-5	ENSMUST00000073554	ENSMUSG00000062329
novel_circ_0000980	mmu-miR-7015-5	ENSMUST00000073554	ENSMUSG00000062329
novel_circ_0000985	mmu-miR-7015-5	ENSMUST00000073554	ENSMUSG00000062329
novel_circ_0000795	mmu-miR-7015-5	ENSMUST00000073554	ENSMUSG00000062329
novel_circ_0000853	mmu-miR-7015-5	ENSMUST00000073554	ENSMUSG00000062329
novel_circ_0000897	mmu-miR-7015-5	ENSMUST00000073554	ENSMUSG00000062329
novel_circ_0000897	mmu-miR-7015-5	ENSMUST00000073554	ENSMUSG00000062329
novel_circ_0000904	mmu-miR-7015-5	ENSMUST00000073554	ENSMUSG00000062329
novel_circ_0001245	mmu-miR-7018-5	ENSMUST00000223576	ENSMUSG00000017491
novel_circ_0001349	mmu-miR-7018-5	ENSMUST00000223576	ENSMUSG00000017491
novel_circ_0001395	mmu-miR-7018-5	ENSMUST00000223576	ENSMUSG00000017491
novel_circ_0001400	mmu-miR-7018-5	ENSMUST00000223576	ENSMUSG00000017491
novel_circ_0001400	mmu-miR-7018-5	ENSMUST00000223576	ENSMUSG00000017491
novel_circ_0001401	mmu-miR-7018-5	ENSMUST00000223576	ENSMUSG00000017491
novel_circ_0001403	mmu-miR-7018-5	ENSMUST00000223576	ENSMUSG00000017491
novel_circ_0001403	mmu-miR-7018-5	ENSMUST00000223576	ENSMUSG00000017491
mmu_circ_0000086	mmu-miR-7018-5	ENSMUST00000223576	ENSMUSG00000017491
mmu_circ_0000086	mmu-miR-7018-5	ENSMUST00000223576	ENSMUSG00000017491
mmu_circ_0000114	mmu-miR-7018-5	ENSMUST00000223576	ENSMUSG00000017491
mmu_circ_0000125	mmu-miR-7018-5	ENSMUST00000223576	ENSMUSG00000017491
mmu_circ_0000129	mmu-miR-7018-5	ENSMUST00000223576	ENSMUSG00000017491
mmu_circ_0001113	mmu-miR-7018-5	ENSMUST00000223576	ENSMUSG00000017491
mmu_circ_0001132	mmu-miR-7018-5	ENSMUST00000223576	ENSMUSG00000017491
mmu_circ_0001103	mmu-miR-7018-5	ENSMUST00000223576	ENSMUSG00000017491
mmu_circ_0001030	mmu-miR-7018-5	ENSMUST00000223576	ENSMUSG00000017491
mmu_circ_0001049	mmu-miR-7018-5	ENSMUST00000223576	ENSMUSG00000017491
mmu_circ_0001376	mmu-miR-7018-5	ENSMUST00000223576	ENSMUSG00000017491
mmu_circ_0001332	mmu-miR-7018-5	ENSMUST00000223576	ENSMUSG00000017491
mmu_circ_0001345	mmu-miR-7018-5	ENSMUST00000223576	ENSMUSG00000017491
mmu_circ_0001356	mmu-miR-7018-5	ENSMUST00000223576	ENSMUSG00000017491
mmu_circ_0001255	mmu-miR-7018-5	ENSMUST00000223576	ENSMUSG00000017491
mmu_circ_0001256	mmu-miR-7018-5	ENSMUST00000223576	ENSMUSG00000017491
mmu_circ_0001261	mmu-miR-7018-5	ENSMUST00000223576	ENSMUSG00000017491
mmu_circ_0001213	mmu-miR-7018-5	ENSMUST00000223576	ENSMUSG00000017491
mmu_circ_0001623	mmu-miR-7018-5	ENSMUST00000223576	ENSMUSG00000017491
mmu_circ_0001454	mmu-miR-7018-5	ENSMUST00000223576	ENSMUSG00000017491
mmu_circ_0001499	mmu-miR-7018-5	ENSMUST00000223576	ENSMUSG00000017491
mmu_circ_0001500	mmu-miR-7018-5	ENSMUST00000223576	ENSMUSG00000017491
mmu_circ_0001659	mmu-miR-7018-5	ENSMUST00000223576	ENSMUSG00000017491
mmu_circ_0001675	mmu-miR-7018-5	ENSMUST00000223576	ENSMUSG00000017491
mmu_circ_0001683	mmu-miR-7018-5	ENSMUST00000223576	ENSMUSG00000017491
mmu_circ_0000287	mmu-miR-7018-5	ENSMUST00000223576	ENSMUSG00000017491
mmu_circ_0000294	mmu-miR-7018-5	ENSMUST00000223576	ENSMUSG00000017491
mmu_circ_0000295	mmu-miR-7018-5	ENSMUST00000223576	ENSMUSG00000017491
mmu_circ_0000144	mmu-miR-7018-5	ENSMUST00000223576	ENSMUSG00000017491
mmu_circ_0000145	mmu-miR-7018-5	ENSMUST00000223576	ENSMUSG00000017491
mmu_circ_0000141	mmu-miR-7018-5	ENSMUST00000223576	ENSMUSG00000017491
mmu_circ_0000499	mmu-miR-7018-5	ENSMUST00000223576	ENSMUSG00000017491
mmu_circ_0000500	mmu-miR-7018-5	ENSMUST00000223576	ENSMUSG00000017491
mmu_circ_0000465	mmu-miR-7018-5	ENSMUST00000223576	ENSMUSG00000017491

mmu_circ_0000399	mmu-miR-7018-5	ENSMUST00000223576	ENSMUSG00000017491
mmu_circ_0000628	mmu-miR-7018-5	ENSMUST00000223576	ENSMUSG00000017491
mmu_circ_0000509	mmu-miR-7018-5	ENSMUST00000223576	ENSMUSG00000017491
mmu_circ_0000530	mmu-miR-7018-5	ENSMUST00000223576	ENSMUSG00000017491
mmu_circ_0000552	mmu-miR-7018-5	ENSMUST00000223576	ENSMUSG00000017491
mmu_circ_0000729	mmu-miR-7018-5	ENSMUST00000223576	ENSMUSG00000017491
mmu_circ_0000812	mmu-miR-7018-5	ENSMUST00000223576	ENSMUSG00000017491
mmu_circ_0000828	mmu-miR-7018-5	ENSMUST00000223576	ENSMUSG00000017491
mmu_circ_0000829	mmu-miR-7018-5	ENSMUST00000223576	ENSMUSG00000017491
mmu_circ_0000831	mmu-miR-7018-5	ENSMUST00000223576	ENSMUSG00000017491
mmu_circ_0000833	mmu-miR-7018-5	ENSMUST00000223576	ENSMUSG00000017491
mmu_circ_0000705	mmu-miR-7018-5	ENSMUST00000223576	ENSMUSG00000017491
mmu_circ_0000712	mmu-miR-7018-5	ENSMUST00000223576	ENSMUSG00000017491
mmu_circ_0000713	mmu-miR-7018-5	ENSMUST00000223576	ENSMUSG00000017491
mmu_circ_0000714	mmu-miR-7018-5	ENSMUST00000223576	ENSMUSG00000017491
mmu_circ_0000950	mmu-miR-7018-5	ENSMUST00000223576	ENSMUSG00000017491
mmu_circ_0000917	mmu-miR-7018-5	ENSMUST00000223576	ENSMUSG00000017491
mmu_circ_0000918	mmu-miR-7018-5	ENSMUST00000223576	ENSMUSG00000017491
mmu_circ_0000919	mmu-miR-7018-5	ENSMUST00000223576	ENSMUSG00000017491
mmu_circ_0000969	mmu-miR-7018-5	ENSMUST00000223576	ENSMUSG00000017491
mmu_circ_0000931	mmu-miR-7018-5	ENSMUST00000223576	ENSMUSG00000017491
mmu_circ_0000894	mmu-miR-7018-5	ENSMUST00000223576	ENSMUSG00000017491
mmu_circ_0000894	mmu-miR-7018-5	ENSMUST00000223576	ENSMUSG00000017491
mmu_circ_0000905	mmu-miR-7018-5	ENSMUST00000223576	ENSMUSG00000017491
mmu_circ_0000912	mmu-miR-7018-5	ENSMUST00000223576	ENSMUSG00000017491
novel_circ_0001561	mmu-miR-7018-5	ENSMUST00000223576	ENSMUSG00000017491
novel_circ_0001700	mmu-miR-7018-5	ENSMUST00000223576	ENSMUSG00000017491
novel_circ_0001710	mmu-miR-7018-5	ENSMUST00000223576	ENSMUSG00000017491
novel_circ_0002185	mmu-miR-7018-5	ENSMUST00000223576	ENSMUSG00000017491
novel_circ_0002213	mmu-miR-7018-5	ENSMUST00000223576	ENSMUSG00000017491
novel_circ_0002215	mmu-miR-7018-5	ENSMUST00000223576	ENSMUSG00000017491
novel_circ_0001759	mmu-miR-7018-5	ENSMUST00000223576	ENSMUSG00000017491
novel_circ_0001802	mmu-miR-7018-5	ENSMUST00000223576	ENSMUSG00000017491
novel_circ_0001802	mmu-miR-7018-5	ENSMUST00000223576	ENSMUSG00000017491
novel_circ_0001875	mmu-miR-7018-5	ENSMUST00000223576	ENSMUSG00000017491
novel_circ_0001998	mmu-miR-7018-5	ENSMUST00000223576	ENSMUSG00000017491
novel_circ_0002011	mmu-miR-7018-5	ENSMUST00000223576	ENSMUSG00000017491
novel_circ_0002014	mmu-miR-7018-5	ENSMUST00000223576	ENSMUSG00000017491
novel_circ_0002042	mmu-miR-7018-5	ENSMUST00000223576	ENSMUSG00000017491
novel_circ_0002434	mmu-miR-7018-5	ENSMUST00000223576	ENSMUSG00000017491
novel_circ_0002439	mmu-miR-7018-5	ENSMUST00000223576	ENSMUSG00000017491
novel_circ_0002441	mmu-miR-7018-5	ENSMUST00000223576	ENSMUSG00000017491
novel_circ_0002455	mmu-miR-7018-5	ENSMUST00000223576	ENSMUSG00000017491
novel_circ_0002456	mmu-miR-7018-5	ENSMUST00000223576	ENSMUSG00000017491
novel_circ_0002525	mmu-miR-7018-5	ENSMUST00000223576	ENSMUSG00000017491
novel_circ_0002584	mmu-miR-7018-5	ENSMUST00000223576	ENSMUSG00000017491
novel_circ_0002228	mmu-miR-7018-5	ENSMUST00000223576	ENSMUSG00000017491
novel_circ_0002286	mmu-miR-7018-5	ENSMUST00000223576	ENSMUSG00000017491
novel_circ_0002330	mmu-miR-7018-5	ENSMUST00000223576	ENSMUSG00000017491
novel_circ_0002343	mmu-miR-7018-5	ENSMUST00000223576	ENSMUSG00000017491
novel_circ_0002344	mmu-miR-7018-5	ENSMUST00000223576	ENSMUSG00000017491
novel_circ_0002356	mmu-miR-7018-5	ENSMUST00000223576	ENSMUSG00000017491
novel_circ_0002831	mmu-miR-7018-5	ENSMUST00000223576	ENSMUSG00000017491
novel_circ_0002844	mmu-miR-7018-5	ENSMUST00000223576	ENSMUSG00000017491
novel_circ_0002844	mmu-miR-7018-5	ENSMUST00000223576	ENSMUSG00000017491
novel_circ_0002854	mmu-miR-7018-5	ENSMUST00000223576	ENSMUSG00000017491
novel_circ_0002884	mmu-miR-7018-5	ENSMUST00000223576	ENSMUSG00000017491
novel_circ_0002921	mmu-miR-7018-5	ENSMUST00000223576	ENSMUSG00000017491

novel_circ_0002944	mmu-miR-7018-5	ENSMUST00000223576	ENSMUSG00000017491
novel_circ_0002686	mmu-miR-7018-5	ENSMUST00000223576	ENSMUSG00000017491
novel_circ_0002722	mmu-miR-7018-5	ENSMUST00000223576	ENSMUSG00000017491
novel_circ_0002732	mmu-miR-7018-5	ENSMUST00000223576	ENSMUSG00000017491
novel_circ_0002740	mmu-miR-7018-5	ENSMUST00000223576	ENSMUSG00000017491
novel_circ_0002785	mmu-miR-7018-5	ENSMUST00000223576	ENSMUSG00000017491
novel_circ_0002785	mmu-miR-7018-5	ENSMUST00000223576	ENSMUSG00000017491
novel_circ_0003219	mmu-miR-7018-5	ENSMUST00000223576	ENSMUSG00000017491
novel_circ_0003219	mmu-miR-7018-5	ENSMUST00000223576	ENSMUSG00000017491
novel_circ_0003236	mmu-miR-7018-5	ENSMUST00000223576	ENSMUSG00000017491
novel_circ_0003242	mmu-miR-7018-5	ENSMUST00000223576	ENSMUSG00000017491
novel_circ_0003263	mmu-miR-7018-5	ENSMUST00000223576	ENSMUSG00000017491
novel_circ_0003264	mmu-miR-7018-5	ENSMUST00000223576	ENSMUSG00000017491
novel_circ_0003266	mmu-miR-7018-5	ENSMUST00000223576	ENSMUSG00000017491
novel_circ_0003370	mmu-miR-7018-5	ENSMUST00000223576	ENSMUSG00000017491
novel_circ_0003054	mmu-miR-7018-5	ENSMUST00000223576	ENSMUSG00000017491
novel_circ_0003064	mmu-miR-7018-5	ENSMUST00000223576	ENSMUSG00000017491
novel_circ_0003078	mmu-miR-7018-5	ENSMUST00000223576	ENSMUSG00000017491
novel_circ_0003087	mmu-miR-7018-5	ENSMUST00000223576	ENSMUSG00000017491
novel_circ_0003152	mmu-miR-7018-5	ENSMUST00000223576	ENSMUSG00000017491
novel_circ_0000170	mmu-miR-7018-5	ENSMUST00000223576	ENSMUSG00000017491
novel_circ_0000244	mmu-miR-7018-5	ENSMUST00000223576	ENSMUSG00000017491
novel_circ_0000268	mmu-miR-7018-5	ENSMUST00000223576	ENSMUSG00000017491
novel_circ_0000288	mmu-miR-7018-5	ENSMUST00000223576	ENSMUSG00000017491
novel_circ_0000298	mmu-miR-7018-5	ENSMUST00000223576	ENSMUSG00000017491
novel_circ_0000298	mmu-miR-7018-5	ENSMUST00000223576	ENSMUSG00000017491
novel_circ_0000299	mmu-miR-7018-5	ENSMUST00000223576	ENSMUSG00000017491
novel_circ_0000025	mmu-miR-7018-5	ENSMUST00000223576	ENSMUSG00000017491
novel_circ_0000054	mmu-miR-7018-5	ENSMUST00000223576	ENSMUSG00000017491
novel_circ_0000154	mmu-miR-7018-5	ENSMUST00000223576	ENSMUSG00000017491
novel_circ_0000632	mmu-miR-7018-5	ENSMUST00000223576	ENSMUSG00000017491
novel_circ_0000698	mmu-miR-7018-5	ENSMUST00000223576	ENSMUSG00000017491
novel_circ_0000756	mmu-miR-7018-5	ENSMUST00000223576	ENSMUSG00000017491
novel_circ_0000781	mmu-miR-7018-5	ENSMUST00000223576	ENSMUSG00000017491
novel_circ_0000597	mmu-miR-7018-5	ENSMUST00000223576	ENSMUSG00000017491
novel_circ_0000958	mmu-miR-7018-5	ENSMUST00000223576	ENSMUSG00000017491
novel_circ_0000985	mmu-miR-7018-5	ENSMUST00000223576	ENSMUSG00000017491
novel_circ_0001009	mmu-miR-7018-5	ENSMUST00000223576	ENSMUSG00000017491
novel_circ_0001048	mmu-miR-7018-5	ENSMUST00000223576	ENSMUSG00000017491
novel_circ_0000877	mmu-miR-7018-5	ENSMUST00000223576	ENSMUSG00000017491
novel_circ_0001172	mmu-miR-7018-5	ENSMUST00000223576	ENSMUSG00000017491
novel_circ_0001186	mmu-miR-7018-5	ENSMUST00000223576	ENSMUSG00000017491
novel_circ_0001188	mmu-miR-7018-5	ENSMUST00000223576	ENSMUSG00000017491
novel_circ_0001189	mmu-miR-7018-5	ENSMUST00000223576	ENSMUSG00000017491
novel_circ_0001207	mmu-miR-7018-5	ENSMUST00000223576	ENSMUSG00000017491
novel_circ_0001208	mmu-miR-7018-5	ENSMUST00000223576	ENSMUSG00000017491
novel_circ_0001222	mmu-miR-7033-5	ENSMUST00000213072	ENSMUSG00000031849
novel_circ_0001240	mmu-miR-7033-5	ENSMUST00000213072	ENSMUSG00000031849
novel_circ_0001241	mmu-miR-7033-5	ENSMUST00000213072	ENSMUSG00000031849
novel_circ_0001251	mmu-miR-7033-5	ENSMUST00000213072	ENSMUSG00000031849
novel_circ_0001133	mmu-miR-7033-5	ENSMUST00000213072	ENSMUSG00000031849
novel_circ_0001133	mmu-miR-7033-5	ENSMUST00000213072	ENSMUSG00000031849
novel_circ_0001458	mmu-miR-7033-5	ENSMUST00000213072	ENSMUSG00000031849
novel_circ_0001469	mmu-miR-7033-5	ENSMUST00000213072	ENSMUSG00000031849
novel_circ_0001471	mmu-miR-7033-5	ENSMUST00000213072	ENSMUSG00000031849
novel_circ_0001478	mmu-miR-7033-5	ENSMUST00000213072	ENSMUSG00000031849
novel_circ_0001480	mmu-miR-7033-5	ENSMUST00000213072	ENSMUSG00000031849
novel_circ_0001522	mmu-miR-7033-5	ENSMUST00000213072	ENSMUSG00000031849

novel_circ_0001374	mmu-miR-7033-5	ENSMUST00000213072	ENSMUSG00000031849
novel_circ_0001382	mmu-miR-7033-5	ENSMUST00000213072	ENSMUSG00000031849
mmu_circ_0000114	mmu-miR-7033-5	ENSMUST00000213072	ENSMUSG00000031849
mmu_circ_0000125	mmu-miR-7033-5	ENSMUST00000213072	ENSMUSG00000031849
mmu_circ_0000130	mmu-miR-7033-5	ENSMUST00000213072	ENSMUSG00000031849
mmu_circ_0000132	mmu-miR-7033-5	ENSMUST00000213072	ENSMUSG00000031849
mmu_circ_0000042	mmu-miR-7033-5	ENSMUST00000213072	ENSMUSG00000031849
mmu_circ_0000046	mmu-miR-7033-5	ENSMUST00000213072	ENSMUSG00000031849
mmu_circ_0000049	mmu-miR-7033-5	ENSMUST00000213072	ENSMUSG00000031849
mmu_circ_0000061	mmu-miR-7033-5	ENSMUST00000213072	ENSMUSG00000031849
mmu_circ_0000063	mmu-miR-7033-5	ENSMUST00000213072	ENSMUSG00000031849
mmu_circ_0001123	mmu-miR-7033-5	ENSMUST00000213072	ENSMUSG00000031849
mmu_circ_0001135	mmu-miR-7033-5	ENSMUST00000213072	ENSMUSG00000031849
mmu_circ_0001030	mmu-miR-7033-5	ENSMUST00000213072	ENSMUSG00000031849
mmu_circ_0001376	mmu-miR-7033-5	ENSMUST00000213072	ENSMUSG00000031849
mmu_circ_0001333	mmu-miR-7033-5	ENSMUST00000213072	ENSMUSG00000031849
mmu_circ_0001364	mmu-miR-7033-5	ENSMUST00000213072	ENSMUSG00000031849
mmu_circ_0001364	mmu-miR-7033-5	ENSMUST00000213072	ENSMUSG00000031849
mmu_circ_0001298	mmu-miR-7033-5	ENSMUST00000213072	ENSMUSG00000031849
mmu_circ_0001213	mmu-miR-7033-5	ENSMUST00000213072	ENSMUSG00000031849
mmu_circ_0001645	mmu-miR-7033-5	ENSMUST00000213072	ENSMUSG00000031849
mmu_circ_0001580	mmu-miR-7033-5	ENSMUST00000213072	ENSMUSG00000031849
mmu_circ_0001518	mmu-miR-7033-5	ENSMUST00000213072	ENSMUSG00000031849
mmu_circ_0001454	mmu-miR-7033-5	ENSMUST00000213072	ENSMUSG00000031849
mmu_circ_0001465	mmu-miR-7033-5	ENSMUST00000213072	ENSMUSG00000031849
mmu_circ_0001473	mmu-miR-7033-5	ENSMUST00000213072	ENSMUSG00000031849
mmu_circ_0001500	mmu-miR-7033-5	ENSMUST00000213072	ENSMUSG00000031849
mmu_circ_0001794	mmu-miR-7033-5	ENSMUST00000213072	ENSMUSG00000031849
mmu_circ_0001794	mmu-miR-7033-5	ENSMUST00000213072	ENSMUSG00000031849
mmu_circ_0001821	mmu-miR-7033-5	ENSMUST00000213072	ENSMUSG00000031849
mmu_circ_0001827	mmu-miR-7033-5	ENSMUST00000213072	ENSMUSG00000031849
mmu_circ_0001665	mmu-miR-7033-5	ENSMUST00000213072	ENSMUSG00000031849
mmu_circ_0001675	mmu-miR-7033-5	ENSMUST00000213072	ENSMUSG00000031849
mmu_circ_0001692	mmu-miR-7033-5	ENSMUST00000213072	ENSMUSG00000031849
mmu_circ_0001700	mmu-miR-7033-5	ENSMUST00000213072	ENSMUSG00000031849
mmu_circ_0000229	mmu-miR-7033-5	ENSMUST00000213072	ENSMUSG00000031849
mmu_circ_0000277	mmu-miR-7033-5	ENSMUST00000213072	ENSMUSG00000031849
mmu_circ_0000287	mmu-miR-7033-5	ENSMUST00000213072	ENSMUSG00000031849
mmu_circ_0000220	mmu-miR-7033-5	ENSMUST00000213072	ENSMUSG00000031849
mmu_circ_0000153	mmu-miR-7033-5	ENSMUST00000213072	ENSMUSG00000031849
mmu_circ_0000154	mmu-miR-7033-5	ENSMUST00000213072	ENSMUSG00000031849
mmu_circ_0000154	mmu-miR-7033-5	ENSMUST00000213072	ENSMUSG00000031849
mmu_circ_0000498	mmu-miR-7033-5	ENSMUST00000213072	ENSMUSG00000031849
mmu_circ_0000499	mmu-miR-7033-5	ENSMUST00000213072	ENSMUSG00000031849
mmu_circ_0000500	mmu-miR-7033-5	ENSMUST00000213072	ENSMUSG00000031849
mmu_circ_0000465	mmu-miR-7033-5	ENSMUST00000213072	ENSMUSG00000031849
mmu_circ_0000423	mmu-miR-7033-5	ENSMUST00000213072	ENSMUSG00000031849
mmu_circ_0000345	mmu-miR-7033-5	ENSMUST00000213072	ENSMUSG00000031849
mmu_circ_0000399	mmu-miR-7033-5	ENSMUST00000213072	ENSMUSG00000031849
mmu_circ_0000604	mmu-miR-7033-5	ENSMUST00000213072	ENSMUSG00000031849
mmu_circ_0000604	mmu-miR-7033-5	ENSMUST00000213072	ENSMUSG00000031849
mmu_circ_0000628	mmu-miR-7033-5	ENSMUST00000213072	ENSMUSG00000031849
mmu_circ_0000526	mmu-miR-7033-5	ENSMUST00000213072	ENSMUSG00000031849
mmu_circ_0000547	mmu-miR-7033-5	ENSMUST00000213072	ENSMUSG00000031849
mmu_circ_0000829	mmu-miR-7033-5	ENSMUST00000213072	ENSMUSG00000031849
mmu_circ_0000658	mmu-miR-7033-5	ENSMUST00000213072	ENSMUSG00000031849
mmu_circ_0000705	mmu-miR-7033-5	ENSMUST00000213072	ENSMUSG00000031849
mmu_circ_0000712	mmu-miR-7033-5	ENSMUST00000213072	ENSMUSG00000031849

mmu_circ_0000714	mmu-miR-7033-5	ENSMUST00000213072	ENSMUSG00000031849
mmu_circ_0000944	mmu-miR-7033-5	ENSMUST00000213072	ENSMUSG00000031849
mmu_circ_0000969	mmu-miR-7033-5	ENSMUST00000213072	ENSMUSG00000031849
mmu_circ_0000864	mmu-miR-7033-5	ENSMUST00000213072	ENSMUSG00000031849
mmu_circ_0000865	mmu-miR-7033-5	ENSMUST00000213072	ENSMUSG00000031849
mmu_circ_0000884	mmu-miR-7033-5	ENSMUST00000213072	ENSMUSG00000031849
mmu_circ_0000886	mmu-miR-7033-5	ENSMUST00000213072	ENSMUSG00000031849
mmu_circ_0000887	mmu-miR-7033-5	ENSMUST00000213072	ENSMUSG00000031849
mmu_circ_0000903	mmu-miR-7033-5	ENSMUST00000213072	ENSMUSG00000031849
mmu_circ_0000906	mmu-miR-7033-5	ENSMUST00000213072	ENSMUSG00000031849
mmu_circ_0000907	mmu-miR-7033-5	ENSMUST00000213072	ENSMUSG00000031849
mmu_circ_0000908	mmu-miR-7033-5	ENSMUST00000213072	ENSMUSG00000031849
mmu_circ_0000912	mmu-miR-7033-5	ENSMUST00000213072	ENSMUSG00000031849
novel_circ_0001546	mmu-miR-7033-5	ENSMUST00000213072	ENSMUSG00000031849
novel_circ_0001546	mmu-miR-7033-5	ENSMUST00000213072	ENSMUSG00000031849
novel_circ_0001547	mmu-miR-7033-5	ENSMUST00000213072	ENSMUSG00000031849
novel_circ_0001549	mmu-miR-7033-5	ENSMUST00000213072	ENSMUSG00000031849
novel_circ_0001549	mmu-miR-7033-5	ENSMUST00000213072	ENSMUSG00000031849
novel_circ_0001553	mmu-miR-7033-5	ENSMUST00000213072	ENSMUSG00000031849
novel_circ_0001553	mmu-miR-7033-5	ENSMUST00000213072	ENSMUSG00000031849
novel_circ_0001561	mmu-miR-7033-5	ENSMUST00000213072	ENSMUSG00000031849
novel_circ_0001579	mmu-miR-7033-5	ENSMUST00000213072	ENSMUSG00000031849
novel_circ_0001604	mmu-miR-7033-5	ENSMUST00000213072	ENSMUSG00000031849
novel_circ_0001605	mmu-miR-7033-5	ENSMUST00000213072	ENSMUSG00000031849
novel_circ_0001615	mmu-miR-7033-5	ENSMUST00000213072	ENSMUSG00000031849
novel_circ_0001650	mmu-miR-7033-5	ENSMUST00000213072	ENSMUSG00000031849
novel_circ_0001658	mmu-miR-7033-5	ENSMUST00000213072	ENSMUSG00000031849
novel_circ_0001678	mmu-miR-7033-5	ENSMUST00000213072	ENSMUSG00000031849
novel_circ_0001708	mmu-miR-7033-5	ENSMUST00000213072	ENSMUSG00000031849
novel_circ_0001746	mmu-miR-7033-5	ENSMUST00000213072	ENSMUSG00000031849
novel_circ_0002100	mmu-miR-7033-5	ENSMUST00000213072	ENSMUSG00000031849
novel_circ_0002114	mmu-miR-7033-5	ENSMUST00000213072	ENSMUSG00000031849
novel_circ_0002164	mmu-miR-7033-5	ENSMUST00000213072	ENSMUSG00000031849
novel_circ_0002180	mmu-miR-7033-5	ENSMUST00000213072	ENSMUSG00000031849
novel_circ_0002209	mmu-miR-7033-5	ENSMUST00000213072	ENSMUSG00000031849
novel_circ_0001777	mmu-miR-7033-5	ENSMUST00000213072	ENSMUSG00000031849
novel_circ_0001782	mmu-miR-7033-5	ENSMUST00000213072	ENSMUSG00000031849
novel_circ_0001809	mmu-miR-7033-5	ENSMUST00000213072	ENSMUSG00000031849
novel_circ_0001811	mmu-miR-7033-5	ENSMUST00000213072	ENSMUSG00000031849
novel_circ_0001842	mmu-miR-7033-5	ENSMUST00000213072	ENSMUSG00000031849
novel_circ_0001888	mmu-miR-7033-5	ENSMUST00000213072	ENSMUSG00000031849
novel_circ_0001898	mmu-miR-7033-5	ENSMUST00000213072	ENSMUSG00000031849
novel_circ_0001933	mmu-miR-7033-5	ENSMUST00000213072	ENSMUSG00000031849
novel_circ_0001933	mmu-miR-7033-5	ENSMUST00000213072	ENSMUSG00000031849
novel_circ_0001936	mmu-miR-7033-5	ENSMUST00000213072	ENSMUSG00000031849
novel_circ_0001957	mmu-miR-7033-5	ENSMUST00000213072	ENSMUSG00000031849
novel_circ_0001958	mmu-miR-7033-5	ENSMUST00000213072	ENSMUSG00000031849
novel_circ_0001965	mmu-miR-7033-5	ENSMUST00000213072	ENSMUSG00000031849
novel_circ_0001982	mmu-miR-7033-5	ENSMUST00000213072	ENSMUSG00000031849
novel_circ_0001987	mmu-miR-7033-5	ENSMUST00000213072	ENSMUSG00000031849
novel_circ_0002011	mmu-miR-7033-5	ENSMUST00000213072	ENSMUSG00000031849
novel_circ_0002027	mmu-miR-7033-5	ENSMUST00000213072	ENSMUSG00000031849
novel_circ_0002035	mmu-miR-7033-5	ENSMUST00000213072	ENSMUSG00000031849
novel_circ_0002043	mmu-miR-7033-5	ENSMUST00000213072	ENSMUSG00000031849
novel_circ_0002417	mmu-miR-7033-5	ENSMUST00000213072	ENSMUSG00000031849
novel_circ_0002427	mmu-miR-7033-5	ENSMUST00000213072	ENSMUSG00000031849
novel_circ_0002434	mmu-miR-7033-5	ENSMUST00000213072	ENSMUSG00000031849
novel_circ_0002447	mmu-miR-7033-5	ENSMUST00000213072	ENSMUSG00000031849

novel_circ_0002448	mmu-miR-7033-5	ENSMUST00000213072	ENSMUSG00000031849
novel_circ_0002449	mmu-miR-7033-5	ENSMUST00000213072	ENSMUSG00000031849
novel_circ_0002476	mmu-miR-7033-5	ENSMUST00000213072	ENSMUSG00000031849
novel_circ_0002489	mmu-miR-7033-5	ENSMUST00000213072	ENSMUSG00000031849
novel_circ_0002499	mmu-miR-7033-5	ENSMUST00000213072	ENSMUSG00000031849
novel_circ_0002503	mmu-miR-7033-5	ENSMUST00000213072	ENSMUSG00000031849
novel_circ_0002504	mmu-miR-7033-5	ENSMUST00000213072	ENSMUSG00000031849
novel_circ_0002522	mmu-miR-7033-5	ENSMUST00000213072	ENSMUSG00000031849
novel_circ_0002525	mmu-miR-7033-5	ENSMUST00000213072	ENSMUSG00000031849
novel_circ_0002556	mmu-miR-7033-5	ENSMUST00000213072	ENSMUSG00000031849
novel_circ_0002557	mmu-miR-7033-5	ENSMUST00000213072	ENSMUSG00000031849
novel_circ_0002576	mmu-miR-7033-5	ENSMUST00000213072	ENSMUSG00000031849
novel_circ_0002578	mmu-miR-7033-5	ENSMUST00000213072	ENSMUSG00000031849
novel_circ_0002584	mmu-miR-7033-5	ENSMUST00000213072	ENSMUSG00000031849
novel_circ_0002243	mmu-miR-7033-5	ENSMUST00000213072	ENSMUSG00000031849
novel_circ_0002250	mmu-miR-7033-5	ENSMUST00000213072	ENSMUSG00000031849
novel_circ_0002275	mmu-miR-7033-5	ENSMUST00000213072	ENSMUSG00000031849
novel_circ_0002278	mmu-miR-7033-5	ENSMUST00000213072	ENSMUSG00000031849
novel_circ_0002278	mmu-miR-7033-5	ENSMUST00000213072	ENSMUSG00000031849
novel_circ_0002303	mmu-miR-7033-5	ENSMUST00000213072	ENSMUSG00000031849
novel_circ_0002303	mmu-miR-7033-5	ENSMUST00000213072	ENSMUSG00000031849
novel_circ_0002323	mmu-miR-7033-5	ENSMUST00000213072	ENSMUSG00000031849
novel_circ_0002343	mmu-miR-7033-5	ENSMUST00000213072	ENSMUSG00000031849
novel_circ_0002356	mmu-miR-7033-5	ENSMUST00000213072	ENSMUSG00000031849
novel_circ_0002356	mmu-miR-7033-5	ENSMUST00000213072	ENSMUSG00000031849
novel_circ_0002362	mmu-miR-7033-5	ENSMUST00000213072	ENSMUSG00000031849
novel_circ_0002364	mmu-miR-7033-5	ENSMUST00000213072	ENSMUSG00000031849
novel_circ_0002366	mmu-miR-7033-5	ENSMUST00000213072	ENSMUSG00000031849
novel_circ_0002368	mmu-miR-7033-5	ENSMUST00000213072	ENSMUSG00000031849
novel_circ_0002374	mmu-miR-7033-5	ENSMUST00000213072	ENSMUSG00000031849
novel_circ_0002387	mmu-miR-7033-5	ENSMUST00000213072	ENSMUSG00000031849
novel_circ_0002853	mmu-miR-7033-5	ENSMUST00000213072	ENSMUSG00000031849
novel_circ_0002914	mmu-miR-7033-5	ENSMUST00000213072	ENSMUSG00000031849
novel_circ_0002926	mmu-miR-7033-5	ENSMUST00000213072	ENSMUSG00000031849
novel_circ_0002934	mmu-miR-7033-5	ENSMUST00000213072	ENSMUSG00000031849
novel_circ_0002943	mmu-miR-7033-5	ENSMUST00000213072	ENSMUSG00000031849
novel_circ_0002944	mmu-miR-7033-5	ENSMUST00000213072	ENSMUSG00000031849
novel_circ_0002962	mmu-miR-7033-5	ENSMUST00000213072	ENSMUSG00000031849
novel_circ_0002971	mmu-miR-7033-5	ENSMUST00000213072	ENSMUSG00000031849
novel_circ_0002998	mmu-miR-7033-5	ENSMUST00000213072	ENSMUSG00000031849
novel_circ_0002742	mmu-miR-7033-5	ENSMUST00000213072	ENSMUSG00000031849
novel_circ_0002788	mmu-miR-7033-5	ENSMUST00000213072	ENSMUSG00000031849
novel_circ_0003213	mmu-miR-7033-5	ENSMUST00000213072	ENSMUSG00000031849
novel_circ_0003240	mmu-miR-7033-5	ENSMUST00000213072	ENSMUSG00000031849
novel_circ_0003298	mmu-miR-7033-5	ENSMUST00000213072	ENSMUSG00000031849
novel_circ_0003014	mmu-miR-7033-5	ENSMUST00000213072	ENSMUSG00000031849
novel_circ_0003015	mmu-miR-7033-5	ENSMUST00000213072	ENSMUSG00000031849
novel_circ_0003018	mmu-miR-7033-5	ENSMUST00000213072	ENSMUSG00000031849
novel_circ_0003018	mmu-miR-7033-5	ENSMUST00000213072	ENSMUSG00000031849
novel_circ_0003028	mmu-miR-7033-5	ENSMUST00000213072	ENSMUSG00000031849
novel_circ_0003028	mmu-miR-7033-5	ENSMUST00000213072	ENSMUSG00000031849
novel_circ_0003074	mmu-miR-7033-5	ENSMUST00000213072	ENSMUSG00000031849
novel_circ_0003083	mmu-miR-7033-5	ENSMUST00000213072	ENSMUSG00000031849
novel_circ_0003113	mmu-miR-7033-5	ENSMUST00000213072	ENSMUSG00000031849
novel_circ_0000164	mmu-miR-7033-5	ENSMUST00000213072	ENSMUSG00000031849
novel_circ_0000170	mmu-miR-7033-5	ENSMUST00000213072	ENSMUSG00000031849
novel_circ_0000204	mmu-miR-7033-5	ENSMUST00000213072	ENSMUSG00000031849
novel_circ_0000204	mmu-miR-7033-5	ENSMUST00000213072	ENSMUSG00000031849

novel_circ_0000249	mmu-miR-7033-5	ENSMUST00000213072	ENSMUSG00000031849
novel_circ_0000264	mmu-miR-7033-5	ENSMUST00000213072	ENSMUSG00000031849
novel_circ_0000267	mmu-miR-7033-5	ENSMUST00000213072	ENSMUSG00000031849
novel_circ_0000287	mmu-miR-7033-5	ENSMUST00000213072	ENSMUSG00000031849
novel_circ_0000289	mmu-miR-7033-5	ENSMUST00000213072	ENSMUSG00000031849
novel_circ_0000296	mmu-miR-7033-5	ENSMUST00000213072	ENSMUSG00000031849
novel_circ_0000301	mmu-miR-7033-5	ENSMUST00000213072	ENSMUSG00000031849
novel_circ_0000316	mmu-miR-7033-5	ENSMUST00000213072	ENSMUSG00000031849
novel_circ_0000324	mmu-miR-7033-5	ENSMUST00000213072	ENSMUSG00000031849
novel_circ_0000329	mmu-miR-7033-5	ENSMUST00000213072	ENSMUSG00000031849
novel_circ_0000334	mmu-miR-7033-5	ENSMUST00000213072	ENSMUSG00000031849
novel_circ_0000359	mmu-miR-7033-5	ENSMUST00000213072	ENSMUSG00000031849
novel_circ_0000366	mmu-miR-7033-5	ENSMUST00000213072	ENSMUSG00000031849
novel_circ_0000437	mmu-miR-7033-5	ENSMUST00000213072	ENSMUSG00000031849
novel_circ_0000437	mmu-miR-7033-5	ENSMUST00000213072	ENSMUSG00000031849
novel_circ_0000447	mmu-miR-7033-5	ENSMUST00000213072	ENSMUSG00000031849
novel_circ_0000010	mmu-miR-7033-5	ENSMUST00000213072	ENSMUSG00000031849
novel_circ_0000017	mmu-miR-7033-5	ENSMUST00000213072	ENSMUSG00000031849
novel_circ_0000050	mmu-miR-7033-5	ENSMUST00000213072	ENSMUSG00000031849
novel_circ_0000072	mmu-miR-7033-5	ENSMUST00000213072	ENSMUSG00000031849
novel_circ_0000108	mmu-miR-7033-5	ENSMUST00000213072	ENSMUSG00000031849
novel_circ_0000118	mmu-miR-7033-5	ENSMUST00000213072	ENSMUSG00000031849
novel_circ_0000119	mmu-miR-7033-5	ENSMUST00000213072	ENSMUSG00000031849
novel_circ_0000119	mmu-miR-7033-5	ENSMUST00000213072	ENSMUSG00000031849
novel_circ_0000126	mmu-miR-7033-5	ENSMUST00000213072	ENSMUSG00000031849
novel_circ_0000134	mmu-miR-7033-5	ENSMUST00000213072	ENSMUSG00000031849
novel_circ_0000134	mmu-miR-7033-5	ENSMUST00000213072	ENSMUSG00000031849
novel_circ_0000138	mmu-miR-7033-5	ENSMUST00000213072	ENSMUSG00000031849
novel_circ_0000670	mmu-miR-7033-5	ENSMUST00000213072	ENSMUSG00000031849
novel_circ_0000687	mmu-miR-7033-5	ENSMUST00000213072	ENSMUSG00000031849
novel_circ_0000492	mmu-miR-7033-5	ENSMUST00000213072	ENSMUSG00000031849
novel_circ_0000492	mmu-miR-7033-5	ENSMUST00000213072	ENSMUSG00000031849
novel_circ_0000504	mmu-miR-7033-5	ENSMUST00000213072	ENSMUSG00000031849
novel_circ_0000505	mmu-miR-7033-5	ENSMUST00000213072	ENSMUSG00000031849
novel_circ_0000521	mmu-miR-7033-5	ENSMUST00000213072	ENSMUSG00000031849
novel_circ_0000589	mmu-miR-7033-5	ENSMUST00000213072	ENSMUSG00000031849
novel_circ_0000602	mmu-miR-7033-5	ENSMUST00000213072	ENSMUSG00000031849
novel_circ_0000958	mmu-miR-7033-5	ENSMUST00000213072	ENSMUSG00000031849
novel_circ_0000985	mmu-miR-7033-5	ENSMUST00000213072	ENSMUSG00000031849
novel_circ_0001001	mmu-miR-7033-5	ENSMUST00000213072	ENSMUSG00000031849
novel_circ_0001009	mmu-miR-7033-5	ENSMUST00000213072	ENSMUSG00000031849
novel_circ_0001046	mmu-miR-7033-5	ENSMUST00000213072	ENSMUSG00000031849
novel_circ_0000820	mmu-miR-7033-5	ENSMUST00000213072	ENSMUSG00000031849
novel_circ_0000823	mmu-miR-7033-5	ENSMUST00000213072	ENSMUSG00000031849
novel_circ_0000838	mmu-miR-7033-5	ENSMUST00000213072	ENSMUSG00000031849
novel_circ_0000853	mmu-miR-7033-5	ENSMUST00000213072	ENSMUSG00000031849
novel_circ_0000853	mmu-miR-7033-5	ENSMUST00000213072	ENSMUSG00000031849
novel_circ_0000861	mmu-miR-7033-5	ENSMUST00000213072	ENSMUSG00000031849
novel_circ_0001186	mmu-miR-7033-5	ENSMUST00000213072	ENSMUSG00000031849
novel_circ_0001194	mmu-miR-7033-5	ENSMUST00000213072	ENSMUSG00000031849
novel_circ_0001211	mmu-miR-7033-5	ENSMUST00000213072	ENSMUSG00000031849
novel_circ_0001103	mmu-miR-7039-5	ENSMUST00000003659	ENSMUSG00000031849
novel_circ_0001103	mmu-miR-7039-5	ENSMUST00000212488	ENSMUSG00000031849
novel_circ_0001473	mmu-miR-7039-5	ENSMUST00000003659	ENSMUSG00000031849
novel_circ_0001473	mmu-miR-7039-5	ENSMUST00000212488	ENSMUSG00000031849
novel_circ_0001473	mmu-miR-7039-5	ENSMUST00000003659	ENSMUSG00000031849
novel_circ_0001473	mmu-miR-7039-5	ENSMUST00000212488	ENSMUSG00000031849
novel_circ_0001476	mmu-miR-7039-5	ENSMUST00000003659	ENSMUSG00000031849

novel_circ_0001476	mmu-miR-7039-5	ENSMUST00000212488	ENSMUSG00000031849
novel_circ_0001382	mmu-miR-7039-5	ENSMUST00000003659	ENSMUSG00000031849
novel_circ_0001382	mmu-miR-7039-5	ENSMUST00000212488	ENSMUSG00000031849
mmu_circ_0000051	mmu-miR-7039-5	ENSMUST00000003659	ENSMUSG00000031849
mmu_circ_0000051	mmu-miR-7039-5	ENSMUST00000212488	ENSMUSG00000031849
mmu_circ_0000052	mmu-miR-7039-5	ENSMUST00000003659	ENSMUSG00000031849
mmu_circ_0000052	mmu-miR-7039-5	ENSMUST00000212488	ENSMUSG00000031849
mmu_circ_0001103	mmu-miR-7039-5	ENSMUST00000003659	ENSMUSG00000031849
mmu_circ_0001103	mmu-miR-7039-5	ENSMUST00000212488	ENSMUSG00000031849
mmu_circ_0001021	mmu-miR-7039-5	ENSMUST00000003659	ENSMUSG00000031849
mmu_circ_0001021	mmu-miR-7039-5	ENSMUST00000212488	ENSMUSG00000031849
mmu_circ_0001376	mmu-miR-7039-5	ENSMUST00000003659	ENSMUSG00000031849
mmu_circ_0001376	mmu-miR-7039-5	ENSMUST00000212488	ENSMUSG00000031849
mmu_circ_0001329	mmu-miR-7039-5	ENSMUST00000003659	ENSMUSG00000031849
mmu_circ_0001329	mmu-miR-7039-5	ENSMUST00000212488	ENSMUSG00000031849
mmu_circ_0001332	mmu-miR-7039-5	ENSMUST00000003659	ENSMUSG00000031849
mmu_circ_0001332	mmu-miR-7039-5	ENSMUST00000212488	ENSMUSG00000031849
mmu_circ_0001341	mmu-miR-7039-5	ENSMUST00000003659	ENSMUSG00000031849
mmu_circ_0001341	mmu-miR-7039-5	ENSMUST00000212488	ENSMUSG00000031849
mmu_circ_0001283	mmu-miR-7039-5	ENSMUST00000003659	ENSMUSG00000031849
mmu_circ_0001283	mmu-miR-7039-5	ENSMUST00000212488	ENSMUSG00000031849
mmu_circ_0001623	mmu-miR-7039-5	ENSMUST00000003659	ENSMUSG00000031849
mmu_circ_0001623	mmu-miR-7039-5	ENSMUST00000212488	ENSMUSG00000031849
mmu_circ_0001635	mmu-miR-7039-5	ENSMUST00000003659	ENSMUSG00000031849
mmu_circ_0001635	mmu-miR-7039-5	ENSMUST00000212488	ENSMUSG00000031849
mmu_circ_0001499	mmu-miR-7039-5	ENSMUST00000003659	ENSMUSG00000031849
mmu_circ_0001499	mmu-miR-7039-5	ENSMUST00000212488	ENSMUSG00000031849
mmu_circ_0001501	mmu-miR-7039-5	ENSMUST00000003659	ENSMUSG00000031849
mmu_circ_0001501	mmu-miR-7039-5	ENSMUST00000212488	ENSMUSG00000031849
mmu_circ_0001659	mmu-miR-7039-5	ENSMUST00000003659	ENSMUSG00000031849
mmu_circ_0001659	mmu-miR-7039-5	ENSMUST00000212488	ENSMUSG00000031849
mmu_circ_0000328	mmu-miR-7039-5	ENSMUST00000003659	ENSMUSG00000031849
mmu_circ_0000328	mmu-miR-7039-5	ENSMUST00000212488	ENSMUSG00000031849
mmu_circ_0000284	mmu-miR-7039-5	ENSMUST00000003659	ENSMUSG00000031849
mmu_circ_0000284	mmu-miR-7039-5	ENSMUST00000212488	ENSMUSG00000031849
mmu_circ_0000233	mmu-miR-7039-5	ENSMUST00000003659	ENSMUSG00000031849
mmu_circ_0000233	mmu-miR-7039-5	ENSMUST00000212488	ENSMUSG00000031849
mmu_circ_0000500	mmu-miR-7039-5	ENSMUST00000003659	ENSMUSG00000031849
mmu_circ_0000500	mmu-miR-7039-5	ENSMUST00000212488	ENSMUSG00000031849
mmu_circ_0000465	mmu-miR-7039-5	ENSMUST00000003659	ENSMUSG00000031849
mmu_circ_0000465	mmu-miR-7039-5	ENSMUST00000212488	ENSMUSG00000031849
mmu_circ_0000384	mmu-miR-7039-5	ENSMUST00000003659	ENSMUSG00000031849
mmu_circ_0000384	mmu-miR-7039-5	ENSMUST00000212488	ENSMUSG00000031849
mmu_circ_0000402	mmu-miR-7039-5	ENSMUST00000003659	ENSMUSG00000031849
mmu_circ_0000402	mmu-miR-7039-5	ENSMUST00000212488	ENSMUSG00000031849
mmu_circ_0000530	mmu-miR-7039-5	ENSMUST00000003659	ENSMUSG00000031849
mmu_circ_0000530	mmu-miR-7039-5	ENSMUST00000212488	ENSMUSG00000031849
mmu_circ_0000738	mmu-miR-7039-5	ENSMUST00000003659	ENSMUSG00000031849
mmu_circ_0000738	mmu-miR-7039-5	ENSMUST00000212488	ENSMUSG00000031849
mmu_circ_0000831	mmu-miR-7039-5	ENSMUST00000003659	ENSMUSG00000031849
mmu_circ_0000831	mmu-miR-7039-5	ENSMUST00000212488	ENSMUSG00000031849
mmu_circ_0000711	mmu-miR-7039-5	ENSMUST00000003659	ENSMUSG00000031849
mmu_circ_0000711	mmu-miR-7039-5	ENSMUST00000212488	ENSMUSG00000031849
mmu_circ_0000969	mmu-miR-7039-5	ENSMUST00000003659	ENSMUSG00000031849
mmu_circ_0000969	mmu-miR-7039-5	ENSMUST00000212488	ENSMUSG00000031849
mmu_circ_0000884	mmu-miR-7039-5	ENSMUST00000003659	ENSMUSG00000031849
mmu_circ_0000884	mmu-miR-7039-5	ENSMUST00000212488	ENSMUSG00000031849
novel_circ_0001551	mmu-miR-7039-5	ENSMUST00000003659	ENSMUSG00000031849

novel_circ_0001551	mmu-miR-7039-5	ENSMUST00000212488	ENSMUSG00000031849
novel_circ_0001580	mmu-miR-7039-5	ENSMUST00000003659	ENSMUSG00000031849
novel_circ_0001580	mmu-miR-7039-5	ENSMUST00000212488	ENSMUSG00000031849
novel_circ_0001609	mmu-miR-7039-5	ENSMUST00000003659	ENSMUSG00000031849
novel_circ_0001609	mmu-miR-7039-5	ENSMUST00000212488	ENSMUSG00000031849
novel_circ_0001809	mmu-miR-7039-5	ENSMUST00000003659	ENSMUSG00000031849
novel_circ_0001809	mmu-miR-7039-5	ENSMUST00000212488	ENSMUSG00000031849
novel_circ_0001809	mmu-miR-7039-5	ENSMUST00000003659	ENSMUSG00000031849
novel_circ_0001809	mmu-miR-7039-5	ENSMUST00000212488	ENSMUSG00000031849
novel_circ_0001842	mmu-miR-7039-5	ENSMUST00000003659	ENSMUSG00000031849
novel_circ_0001842	mmu-miR-7039-5	ENSMUST00000212488	ENSMUSG00000031849
novel_circ_0001873	mmu-miR-7039-5	ENSMUST00000003659	ENSMUSG00000031849
novel_circ_0001873	mmu-miR-7039-5	ENSMUST00000212488	ENSMUSG00000031849
novel_circ_0001905	mmu-miR-7039-5	ENSMUST00000003659	ENSMUSG00000031849
novel_circ_0001905	mmu-miR-7039-5	ENSMUST00000212488	ENSMUSG00000031849
novel_circ_0001911	mmu-miR-7039-5	ENSMUST00000003659	ENSMUSG00000031849
novel_circ_0001911	mmu-miR-7039-5	ENSMUST00000212488	ENSMUSG00000031849
novel_circ_0001913	mmu-miR-7039-5	ENSMUST00000003659	ENSMUSG00000031849
novel_circ_0001913	mmu-miR-7039-5	ENSMUST00000212488	ENSMUSG00000031849
novel_circ_0001914	mmu-miR-7039-5	ENSMUST00000003659	ENSMUSG00000031849
novel_circ_0001914	mmu-miR-7039-5	ENSMUST00000212488	ENSMUSG00000031849
novel_circ_0001957	mmu-miR-7039-5	ENSMUST00000003659	ENSMUSG00000031849
novel_circ_0001957	mmu-miR-7039-5	ENSMUST00000212488	ENSMUSG00000031849
novel_circ_0001958	mmu-miR-7039-5	ENSMUST00000003659	ENSMUSG00000031849
novel_circ_0001958	mmu-miR-7039-5	ENSMUST00000212488	ENSMUSG00000031849
novel_circ_0001990	mmu-miR-7039-5	ENSMUST00000003659	ENSMUSG00000031849
novel_circ_0001990	mmu-miR-7039-5	ENSMUST00000212488	ENSMUSG00000031849
novel_circ_0002403	mmu-miR-7039-5	ENSMUST00000003659	ENSMUSG00000031849
novel_circ_0002403	mmu-miR-7039-5	ENSMUST00000212488	ENSMUSG00000031849
novel_circ_0002488	mmu-miR-7039-5	ENSMUST00000003659	ENSMUSG00000031849
novel_circ_0002488	mmu-miR-7039-5	ENSMUST00000212488	ENSMUSG00000031849
novel_circ_0002488	mmu-miR-7039-5	ENSMUST00000003659	ENSMUSG00000031849
novel_circ_0002488	mmu-miR-7039-5	ENSMUST00000212488	ENSMUSG00000031849
novel_circ_0002301	mmu-miR-7039-5	ENSMUST00000003659	ENSMUSG00000031849
novel_circ_0002301	mmu-miR-7039-5	ENSMUST00000212488	ENSMUSG00000031849
novel_circ_0002318	mmu-miR-7039-5	ENSMUST00000003659	ENSMUSG00000031849
novel_circ_0002318	mmu-miR-7039-5	ENSMUST00000212488	ENSMUSG00000031849
novel_circ_0002319	mmu-miR-7039-5	ENSMUST00000003659	ENSMUSG00000031849
novel_circ_0002319	mmu-miR-7039-5	ENSMUST00000212488	ENSMUSG00000031849
novel_circ_0002363	mmu-miR-7039-5	ENSMUST00000003659	ENSMUSG00000031849
novel_circ_0002363	mmu-miR-7039-5	ENSMUST00000212488	ENSMUSG00000031849
novel_circ_0002364	mmu-miR-7039-5	ENSMUST00000003659	ENSMUSG00000031849
novel_circ_0002364	mmu-miR-7039-5	ENSMUST00000212488	ENSMUSG00000031849
novel_circ_0002866	mmu-miR-7039-5	ENSMUST00000003659	ENSMUSG00000031849
novel_circ_0002866	mmu-miR-7039-5	ENSMUST00000212488	ENSMUSG00000031849
novel_circ_0002978	mmu-miR-7039-5	ENSMUST00000003659	ENSMUSG00000031849
novel_circ_0002978	mmu-miR-7039-5	ENSMUST00000212488	ENSMUSG00000031849
novel_circ_0002978	mmu-miR-7039-5	ENSMUST00000003659	ENSMUSG00000031849
novel_circ_0002978	mmu-miR-7039-5	ENSMUST00000212488	ENSMUSG00000031849
novel_circ_0003208	mmu-miR-7039-5	ENSMUST00000003659	ENSMUSG00000031849
novel_circ_0003208	mmu-miR-7039-5	ENSMUST00000212488	ENSMUSG00000031849
novel_circ_0003313	mmu-miR-7039-5	ENSMUST00000003659	ENSMUSG00000031849
novel_circ_0003313	mmu-miR-7039-5	ENSMUST00000212488	ENSMUSG00000031849
novel_circ_0003368	mmu-miR-7039-5	ENSMUST00000003659	ENSMUSG00000031849
novel_circ_0003368	mmu-miR-7039-5	ENSMUST00000212488	ENSMUSG00000031849
novel_circ_0003023	mmu-miR-7039-5	ENSMUST00000003659	ENSMUSG00000031849
novel_circ_0003023	mmu-miR-7039-5	ENSMUST00000212488	ENSMUSG00000031849
novel_circ_0000164	mmu-miR-7039-5	ENSMUST00000003659	ENSMUSG00000031849

novel_circ_0000164	mmu-miR-7039-5	ENSMUST00000212488	ENSMUSG00000031849
novel_circ_0000252	mmu-miR-7039-5	ENSMUST00000003659	ENSMUSG00000031849
novel_circ_0000252	mmu-miR-7039-5	ENSMUST00000212488	ENSMUSG00000031849
novel_circ_0000148	mmu-miR-7039-5	ENSMUST00000003659	ENSMUSG00000031849
novel_circ_0000148	mmu-miR-7039-5	ENSMUST00000212488	ENSMUSG00000031849
novel_circ_0000753	mmu-miR-7039-5	ENSMUST00000003659	ENSMUSG00000031849
novel_circ_0000753	mmu-miR-7039-5	ENSMUST00000212488	ENSMUSG00000031849
novel_circ_0000591	mmu-miR-7039-5	ENSMUST00000003659	ENSMUSG00000031849
novel_circ_0000591	mmu-miR-7039-5	ENSMUST00000212488	ENSMUSG00000031849
novel_circ_0000968	mmu-miR-7039-5	ENSMUST00000003659	ENSMUSG00000031849
novel_circ_0000968	mmu-miR-7039-5	ENSMUST00000212488	ENSMUSG00000031849
novel_circ_0000831	mmu-miR-7039-5	ENSMUST00000003659	ENSMUSG00000031849
novel_circ_0000831	mmu-miR-7039-5	ENSMUST00000212488	ENSMUSG00000031849
novel_circ_0001232	mmu-miR-7080-5	ENSMUST00000073554	ENSMUSG00000062329
mmu_circ_0000132	mmu-miR-7080-5	ENSMUST00000073554	ENSMUSG00000062329
mmu_circ_0000132	mmu-miR-7080-5	ENSMUST00000073554	ENSMUSG00000062329
mmu_circ_0000057	mmu-miR-7080-5	ENSMUST00000073554	ENSMUSG00000062329
mmu_circ_0000063	mmu-miR-7080-5	ENSMUST00000073554	ENSMUSG00000062329
mmu_circ_0001153	mmu-miR-7080-5	ENSMUST00000073554	ENSMUSG00000062329
mmu_circ_0001068	mmu-miR-7080-5	ENSMUST00000073554	ENSMUSG00000062329
mmu_circ_0001103	mmu-miR-7080-5	ENSMUST00000073554	ENSMUSG00000062329
mmu_circ_0001376	mmu-miR-7080-5	ENSMUST00000073554	ENSMUSG00000062329
mmu_circ_0001333	mmu-miR-7080-5	ENSMUST00000073554	ENSMUSG00000062329
mmu_circ_0001500	mmu-miR-7080-5	ENSMUST00000073554	ENSMUSG00000062329
mmu_circ_0001766	mmu-miR-7080-5	ENSMUST00000073554	ENSMUSG00000062329
mmu_circ_0001767	mmu-miR-7080-5	ENSMUST00000073554	ENSMUSG00000062329
mmu_circ_0000349	mmu-miR-7080-5	ENSMUST00000073554	ENSMUSG00000062329
mmu_circ_0000399	mmu-miR-7080-5	ENSMUST00000073554	ENSMUSG00000062329
mmu_circ_0000530	mmu-miR-7080-5	ENSMUST00000073554	ENSMUSG00000062329
mmu_circ_0000798	mmu-miR-7080-5	ENSMUST00000073554	ENSMUSG00000062329
mmu_circ_0000828	mmu-miR-7080-5	ENSMUST00000073554	ENSMUSG00000062329
mmu_circ_0000829	mmu-miR-7080-5	ENSMUST00000073554	ENSMUSG00000062329
mmu_circ_0000831	mmu-miR-7080-5	ENSMUST00000073554	ENSMUSG00000062329
mmu_circ_0000917	mmu-miR-7080-5	ENSMUST00000073554	ENSMUSG00000062329
mmu_circ_0000918	mmu-miR-7080-5	ENSMUST00000073554	ENSMUSG00000062329
mmu_circ_0000919	mmu-miR-7080-5	ENSMUST00000073554	ENSMUSG00000062329
mmu_circ_0000969	mmu-miR-7080-5	ENSMUST00000073554	ENSMUSG00000062329
mmu_circ_0000906	mmu-miR-7080-5	ENSMUST00000073554	ENSMUSG00000062329
mmu_circ_0000907	mmu-miR-7080-5	ENSMUST00000073554	ENSMUSG00000062329
novel_circ_0001650	mmu-miR-7080-5	ENSMUST00000073554	ENSMUSG00000062329
novel_circ_0001650	mmu-miR-7080-5	ENSMUST00000073554	ENSMUSG00000062329
novel_circ_0002215	mmu-miR-7080-5	ENSMUST00000073554	ENSMUSG00000062329
novel_circ_0001905	mmu-miR-7080-5	ENSMUST00000073554	ENSMUSG00000062329
novel_circ_0002478	mmu-miR-7080-5	ENSMUST00000073554	ENSMUSG00000062329
novel_circ_0002518	mmu-miR-7080-5	ENSMUST00000073554	ENSMUSG00000062329
novel_circ_0002252	mmu-miR-7080-5	ENSMUST00000073554	ENSMUSG00000062329
novel_circ_0002260	mmu-miR-7080-5	ENSMUST00000073554	ENSMUSG00000062329
novel_circ_0002260	mmu-miR-7080-5	ENSMUST00000073554	ENSMUSG00000062329
novel_circ_0002287	mmu-miR-7080-5	ENSMUST00000073554	ENSMUSG00000062329
novel_circ_0002287	mmu-miR-7080-5	ENSMUST00000073554	ENSMUSG00000062329
novel_circ_0002301	mmu-miR-7080-5	ENSMUST00000073554	ENSMUSG00000062329
novel_circ_0002359	mmu-miR-7080-5	ENSMUST00000073554	ENSMUSG00000062329
novel_circ_0002964	mmu-miR-7080-5	ENSMUST00000073554	ENSMUSG00000062329
novel_circ_0000264	mmu-miR-7080-5	ENSMUST00000073554	ENSMUSG00000062329
novel_circ_0000327	mmu-miR-7080-5	ENSMUST00000073554	ENSMUSG00000062329
novel_circ_0000368	mmu-miR-7080-5	ENSMUST00000073554	ENSMUSG00000062329
novel_circ_0000009	mmu-miR-7080-5	ENSMUST00000073554	ENSMUSG00000062329
novel_circ_0000021	mmu-miR-7080-5	ENSMUST00000073554	ENSMUSG00000062329

novel_circ_0000627	mmu-miR-7080-5	ENSMUST00000073554	ENSMUSG00000062329
novel_circ_0000627	mmu-miR-7080-5	ENSMUST00000073554	ENSMUSG00000062329
novel_circ_0000766	mmu-miR-7080-5	ENSMUST00000073554	ENSMUSG00000062329
novel_circ_0000525	mmu-miR-7080-5	ENSMUST00000073554	ENSMUSG00000062329
novel_circ_0000852	mmu-miR-7080-5	ENSMUST00000073554	ENSMUSG00000062329
novel_circ_0000858	mmu-miR-7080-5	ENSMUST00000073554	ENSMUSG00000062329
novel_circ_0001201	mmu-miR-7080-5	ENSMUST00000073554	ENSMUSG00000062329
novel_circ_0001220	mmu-miR-7648-3	ENSMUST00000213072	ENSMUSG00000031849
novel_circ_0001230	mmu-miR-7648-3	ENSMUST00000213072	ENSMUSG00000031849
novel_circ_0001112	mmu-miR-7648-3	ENSMUST00000213072	ENSMUSG00000031849
novel_circ_0001141	mmu-miR-7648-3	ENSMUST00000213072	ENSMUSG00000031849
novel_circ_0001478	mmu-miR-7648-3	ENSMUST00000213072	ENSMUSG00000031849
novel_circ_0001495	mmu-miR-7648-3	ENSMUST00000213072	ENSMUSG00000031849
novel_circ_0001339	mmu-miR-7648-3	ENSMUST00000213072	ENSMUSG00000031849
novel_circ_0001349	mmu-miR-7648-3	ENSMUST00000213072	ENSMUSG00000031849
novel_circ_0001375	mmu-miR-7648-3	ENSMUST00000213072	ENSMUSG00000031849
mmu_circ_0000010	mmu-miR-7648-3	ENSMUST00000213072	ENSMUSG00000031849
mmu_circ_0000011	mmu-miR-7648-3	ENSMUST00000213072	ENSMUSG00000031849
mmu_circ_0000028	mmu-miR-7648-3	ENSMUST00000213072	ENSMUSG00000031849
mmu_circ_0000063	mmu-miR-7648-3	ENSMUST00000213072	ENSMUSG00000031849
mmu_circ_0001170	mmu-miR-7648-3	ENSMUST00000213072	ENSMUSG00000031849
mmu_circ_0001123	mmu-miR-7648-3	ENSMUST00000213072	ENSMUSG00000031849
mmu_circ_0001103	mmu-miR-7648-3	ENSMUST00000213072	ENSMUSG00000031849
mmu_circ_0001049	mmu-miR-7648-3	ENSMUST00000213072	ENSMUSG00000031849
mmu_circ_0001376	mmu-miR-7648-3	ENSMUST00000213072	ENSMUSG00000031849
mmu_circ_0001387	mmu-miR-7648-3	ENSMUST00000213072	ENSMUSG00000031849
mmu_circ_0001396	mmu-miR-7648-3	ENSMUST00000213072	ENSMUSG00000031849
mmu_circ_0001427	mmu-miR-7648-3	ENSMUST00000213072	ENSMUSG00000031849
mmu_circ_0001351	mmu-miR-7648-3	ENSMUST00000213072	ENSMUSG00000031849
mmu_circ_0001271	mmu-miR-7648-3	ENSMUST00000213072	ENSMUSG00000031849
mmu_circ_0001283	mmu-miR-7648-3	ENSMUST00000213072	ENSMUSG00000031849
mmu_circ_0001636	mmu-miR-7648-3	ENSMUST00000213072	ENSMUSG00000031849
mmu_circ_0001505	mmu-miR-7648-3	ENSMUST00000213072	ENSMUSG00000031849
mmu_circ_0001516	mmu-miR-7648-3	ENSMUST00000213072	ENSMUSG00000031849
mmu_circ_0001516	mmu-miR-7648-3	ENSMUST00000213072	ENSMUSG00000031849
mmu_circ_0001528	mmu-miR-7648-3	ENSMUST00000213072	ENSMUSG00000031849
mmu_circ_0001454	mmu-miR-7648-3	ENSMUST00000213072	ENSMUSG00000031849
mmu_circ_0001473	mmu-miR-7648-3	ENSMUST00000213072	ENSMUSG00000031849
mmu_circ_0001499	mmu-miR-7648-3	ENSMUST00000213072	ENSMUSG00000031849
mmu_circ_0001500	mmu-miR-7648-3	ENSMUST00000213072	ENSMUSG00000031849
mmu_circ_0001842	mmu-miR-7648-3	ENSMUST00000213072	ENSMUSG00000031849
mmu_circ_0001795	mmu-miR-7648-3	ENSMUST00000213072	ENSMUSG00000031849
mmu_circ_0001825	mmu-miR-7648-3	ENSMUST00000213072	ENSMUSG00000031849
mmu_circ_0001827	mmu-miR-7648-3	ENSMUST00000213072	ENSMUSG00000031849
mmu_circ_0001698	mmu-miR-7648-3	ENSMUST00000213072	ENSMUSG00000031849
mmu_circ_0001718	mmu-miR-7648-3	ENSMUST00000213072	ENSMUSG00000031849
mmu_circ_0000230	mmu-miR-7648-3	ENSMUST00000213072	ENSMUSG00000031849
mmu_circ_0000301	mmu-miR-7648-3	ENSMUST00000213072	ENSMUSG00000031849
mmu_circ_0000233	mmu-miR-7648-3	ENSMUST00000213072	ENSMUSG00000031849
mmu_circ_0000153	mmu-miR-7648-3	ENSMUST00000213072	ENSMUSG00000031849
mmu_circ_0000154	mmu-miR-7648-3	ENSMUST00000213072	ENSMUSG00000031849
mmu_circ_0000499	mmu-miR-7648-3	ENSMUST00000213072	ENSMUSG00000031849
mmu_circ_0000371	mmu-miR-7648-3	ENSMUST00000213072	ENSMUSG00000031849
mmu_circ_0000530	mmu-miR-7648-3	ENSMUST00000213072	ENSMUSG00000031849
mmu_circ_0000735	mmu-miR-7648-3	ENSMUST00000213072	ENSMUSG00000031849
mmu_circ_0000738	mmu-miR-7648-3	ENSMUST00000213072	ENSMUSG00000031849
mmu_circ_0000738	mmu-miR-7648-3	ENSMUST00000213072	ENSMUSG00000031849
mmu_circ_0000798	mmu-miR-7648-3	ENSMUST00000213072	ENSMUSG00000031849

mmu_circ_0000829	mmu-miR-7648-3	ENSMUST00000213072	ENSMUSG00000031849
mmu_circ_0000831	mmu-miR-7648-3	ENSMUST00000213072	ENSMUSG00000031849
mmu_circ_0000688	mmu-miR-7648-3	ENSMUST00000213072	ENSMUSG00000031849
mmu_circ_0000711	mmu-miR-7648-3	ENSMUST00000213072	ENSMUSG00000031849
mmu_circ_0000713	mmu-miR-7648-3	ENSMUST00000213072	ENSMUSG00000031849
mmu_circ_0000714	mmu-miR-7648-3	ENSMUST00000213072	ENSMUSG00000031849
mmu_circ_0000950	mmu-miR-7648-3	ENSMUST00000213072	ENSMUSG00000031849
mmu_circ_0000969	mmu-miR-7648-3	ENSMUST00000213072	ENSMUSG00000031849
mmu_circ_0000884	mmu-miR-7648-3	ENSMUST00000213072	ENSMUSG00000031849
mmu_circ_0000886	mmu-miR-7648-3	ENSMUST00000213072	ENSMUSG00000031849
mmu_circ_0000905	mmu-miR-7648-3	ENSMUST00000213072	ENSMUSG00000031849
mmu_circ_0000906	mmu-miR-7648-3	ENSMUST00000213072	ENSMUSG00000031849
mmu_circ_0000907	mmu-miR-7648-3	ENSMUST00000213072	ENSMUSG00000031849
mmu_circ_0000908	mmu-miR-7648-3	ENSMUST00000213072	ENSMUSG00000031849
novel_circ_0001564	mmu-miR-7648-3	ENSMUST00000213072	ENSMUSG00000031849
novel_circ_0001566	mmu-miR-7648-3	ENSMUST00000213072	ENSMUSG00000031849
novel_circ_0001579	mmu-miR-7648-3	ENSMUST00000213072	ENSMUSG00000031849
novel_circ_0001619	mmu-miR-7648-3	ENSMUST00000213072	ENSMUSG00000031849
novel_circ_0001728	mmu-miR-7648-3	ENSMUST00000213072	ENSMUSG00000031849
novel_circ_0002061	mmu-miR-7648-3	ENSMUST00000213072	ENSMUSG00000031849
novel_circ_0001759	mmu-miR-7648-3	ENSMUST00000213072	ENSMUSG00000031849
novel_circ_0001806	mmu-miR-7648-3	ENSMUST00000213072	ENSMUSG00000031849
novel_circ_0001859	mmu-miR-7648-3	ENSMUST00000213072	ENSMUSG00000031849
novel_circ_0001875	mmu-miR-7648-3	ENSMUST00000213072	ENSMUSG00000031849
novel_circ_0001875	mmu-miR-7648-3	ENSMUST00000213072	ENSMUSG00000031849
novel_circ_0001946	mmu-miR-7648-3	ENSMUST00000213072	ENSMUSG00000031849
novel_circ_0001992	mmu-miR-7648-3	ENSMUST00000213072	ENSMUSG00000031849
novel_circ_0002030	mmu-miR-7648-3	ENSMUST00000213072	ENSMUSG00000031849
novel_circ_0002441	mmu-miR-7648-3	ENSMUST00000213072	ENSMUSG00000031849
novel_circ_0002450	mmu-miR-7648-3	ENSMUST00000213072	ENSMUSG00000031849
novel_circ_0002451	mmu-miR-7648-3	ENSMUST00000213072	ENSMUSG00000031849
novel_circ_0002499	mmu-miR-7648-3	ENSMUST00000213072	ENSMUSG00000031849
novel_circ_0002503	mmu-miR-7648-3	ENSMUST00000213072	ENSMUSG00000031849
novel_circ_0002504	mmu-miR-7648-3	ENSMUST00000213072	ENSMUSG00000031849
novel_circ_0002288	mmu-miR-7648-3	ENSMUST00000213072	ENSMUSG00000031849
novel_circ_0002327	mmu-miR-7648-3	ENSMUST00000213072	ENSMUSG00000031849
novel_circ_0002343	mmu-miR-7648-3	ENSMUST00000213072	ENSMUSG00000031849
novel_circ_0002826	mmu-miR-7648-3	ENSMUST00000213072	ENSMUSG00000031849
novel_circ_0002835	mmu-miR-7648-3	ENSMUST00000213072	ENSMUSG00000031849
novel_circ_0002866	mmu-miR-7648-3	ENSMUST00000213072	ENSMUSG00000031849
novel_circ_0002871	mmu-miR-7648-3	ENSMUST00000213072	ENSMUSG00000031849
novel_circ_0002895	mmu-miR-7648-3	ENSMUST00000213072	ENSMUSG00000031849
novel_circ_0002913	mmu-miR-7648-3	ENSMUST00000213072	ENSMUSG00000031849
novel_circ_0002917	mmu-miR-7648-3	ENSMUST00000213072	ENSMUSG00000031849
novel_circ_0002917	mmu-miR-7648-3	ENSMUST00000213072	ENSMUSG00000031849
novel_circ_0002923	mmu-miR-7648-3	ENSMUST00000213072	ENSMUSG00000031849
novel_circ_0002977	mmu-miR-7648-3	ENSMUST00000213072	ENSMUSG00000031849
novel_circ_0002640	mmu-miR-7648-3	ENSMUST00000213072	ENSMUSG00000031849
novel_circ_0002706	mmu-miR-7648-3	ENSMUST00000213072	ENSMUSG00000031849
novel_circ_0002706	mmu-miR-7648-3	ENSMUST00000213072	ENSMUSG00000031849
novel_circ_0002732	mmu-miR-7648-3	ENSMUST00000213072	ENSMUSG00000031849
novel_circ_0002743	mmu-miR-7648-3	ENSMUST00000213072	ENSMUSG00000031849
novel_circ_0002743	mmu-miR-7648-3	ENSMUST00000213072	ENSMUSG00000031849
novel_circ_0002782	mmu-miR-7648-3	ENSMUST00000213072	ENSMUSG00000031849
novel_circ_0002788	mmu-miR-7648-3	ENSMUST00000213072	ENSMUSG00000031849
novel_circ_0002812	mmu-miR-7648-3	ENSMUST00000213072	ENSMUSG00000031849
novel_circ_0003177	mmu-miR-7648-3	ENSMUST00000213072	ENSMUSG00000031849
novel_circ_0003191	mmu-miR-7648-3	ENSMUST00000213072	ENSMUSG00000031849

novel_circ_0003219	mmu-miR-7648-3	ENSMUST00000213072	ENSMUSG00000031849
novel_circ_0003220	mmu-miR-7648-3	ENSMUST00000213072	ENSMUSG00000031849
novel_circ_0003220	mmu-miR-7648-3	ENSMUST00000213072	ENSMUSG00000031849
novel_circ_0003230	mmu-miR-7648-3	ENSMUST00000213072	ENSMUSG00000031849
novel_circ_0003240	mmu-miR-7648-3	ENSMUST00000213072	ENSMUSG00000031849
novel_circ_0003249	mmu-miR-7648-3	ENSMUST00000213072	ENSMUSG00000031849
novel_circ_0003397	mmu-miR-7648-3	ENSMUST00000213072	ENSMUSG00000031849
novel_circ_0003399	mmu-miR-7648-3	ENSMUST00000213072	ENSMUSG00000031849
novel_circ_0003017	mmu-miR-7648-3	ENSMUST00000213072	ENSMUSG00000031849
novel_circ_0003083	mmu-miR-7648-3	ENSMUST00000213072	ENSMUSG00000031849
novel_circ_0003142	mmu-miR-7648-3	ENSMUST00000213072	ENSMUSG00000031849
novel_circ_0003491	mmu-miR-7648-3	ENSMUST00000213072	ENSMUSG00000031849
novel_circ_0000344	mmu-miR-7648-3	ENSMUST00000213072	ENSMUSG00000031849
novel_circ_0000344	mmu-miR-7648-3	ENSMUST00000213072	ENSMUSG00000031849
novel_circ_0000441	mmu-miR-7648-3	ENSMUST00000213072	ENSMUSG00000031849
novel_circ_0000109	mmu-miR-7648-3	ENSMUST00000213072	ENSMUSG00000031849
novel_circ_0000129	mmu-miR-7648-3	ENSMUST00000213072	ENSMUSG00000031849
novel_circ_0000148	mmu-miR-7648-3	ENSMUST00000213072	ENSMUSG00000031849
novel_circ_0000663	mmu-miR-7648-3	ENSMUST00000213072	ENSMUSG00000031849
novel_circ_0000668	mmu-miR-7648-3	ENSMUST00000213072	ENSMUSG00000031849
novel_circ_0000589	mmu-miR-7648-3	ENSMUST00000213072	ENSMUSG00000031849
novel_circ_0000594	mmu-miR-7648-3	ENSMUST00000213072	ENSMUSG00000031849
novel_circ_0000958	mmu-miR-7648-3	ENSMUST00000213072	ENSMUSG00000031849
novel_circ_0001048	mmu-miR-7648-3	ENSMUST00000213072	ENSMUSG00000031849
novel_circ_0000843	mmu-miR-7648-3	ENSMUST00000213072	ENSMUSG00000031849
novel_circ_0001223	mmu-miR-7667-5	ENSMUST00000213072	ENSMUSG00000031849
novel_circ_0001248	mmu-miR-7667-5	ENSMUST00000213072	ENSMUSG00000031849
novel_circ_0001251	mmu-miR-7667-5	ENSMUST00000213072	ENSMUSG00000031849
novel_circ_0001290	mmu-miR-7667-5	ENSMUST00000213072	ENSMUSG00000031849
novel_circ_0001478	mmu-miR-7667-5	ENSMUST00000213072	ENSMUSG00000031849
novel_circ_0001513	mmu-miR-7667-5	ENSMUST00000213072	ENSMUSG00000031849
novel_circ_0001357	mmu-miR-7667-5	ENSMUST00000213072	ENSMUSG00000031849
novel_circ_0001357	mmu-miR-7667-5	ENSMUST00000213072	ENSMUSG00000031849
novel_circ_0001398	mmu-miR-7667-5	ENSMUST00000213072	ENSMUSG00000031849
mmu_circ_0000087	mmu-miR-7667-5	ENSMUST00000213072	ENSMUSG00000031849
mmu_circ_0000132	mmu-miR-7667-5	ENSMUST00000213072	ENSMUSG00000031849
mmu_circ_0000133	mmu-miR-7667-5	ENSMUST00000213072	ENSMUSG00000031849
mmu_circ_0000029	mmu-miR-7667-5	ENSMUST00000213072	ENSMUSG00000031849
mmu_circ_0000049	mmu-miR-7667-5	ENSMUST00000213072	ENSMUSG00000031849
mmu_circ_0001123	mmu-miR-7667-5	ENSMUST00000213072	ENSMUSG00000031849
mmu_circ_0001028	mmu-miR-7667-5	ENSMUST00000213072	ENSMUSG00000031849
mmu_circ_0001028	mmu-miR-7667-5	ENSMUST00000213072	ENSMUSG00000031849
mmu_circ_0001376	mmu-miR-7667-5	ENSMUST00000213072	ENSMUSG00000031849
mmu_circ_0001634	mmu-miR-7667-5	ENSMUST00000213072	ENSMUSG00000031849
mmu_circ_0001561	mmu-miR-7667-5	ENSMUST00000213072	ENSMUSG00000031849
mmu_circ_0001598	mmu-miR-7667-5	ENSMUST00000213072	ENSMUSG00000031849
mmu_circ_0001507	mmu-miR-7667-5	ENSMUST00000213072	ENSMUSG00000031849
mmu_circ_0001528	mmu-miR-7667-5	ENSMUST00000213072	ENSMUSG00000031849
mmu_circ_0001454	mmu-miR-7667-5	ENSMUST00000213072	ENSMUSG00000031849
mmu_circ_0001500	mmu-miR-7667-5	ENSMUST00000213072	ENSMUSG00000031849
mmu_circ_0001827	mmu-miR-7667-5	ENSMUST00000213072	ENSMUSG00000031849
mmu_circ_0001697	mmu-miR-7667-5	ENSMUST00000213072	ENSMUSG00000031849
mmu_circ_0001698	mmu-miR-7667-5	ENSMUST00000213072	ENSMUSG00000031849
mmu_circ_0000301	mmu-miR-7667-5	ENSMUST00000213072	ENSMUSG00000031849
mmu_circ_0000500	mmu-miR-7667-5	ENSMUST00000213072	ENSMUSG00000031849
mmu_circ_0000465	mmu-miR-7667-5	ENSMUST00000213072	ENSMUSG00000031849
mmu_circ_0000482	mmu-miR-7667-5	ENSMUST00000213072	ENSMUSG00000031849
mmu_circ_0000491	mmu-miR-7667-5	ENSMUST00000213072	ENSMUSG00000031849

mmu_circ_0000349	mmu-miR-7667-5	ENSMUST00000213072	ENSMUSG00000031849
mmu_circ_0000374	mmu-miR-7667-5	ENSMUST00000213072	ENSMUSG00000031849
mmu_circ_0000402	mmu-miR-7667-5	ENSMUST00000213072	ENSMUSG00000031849
mmu_circ_0000628	mmu-miR-7667-5	ENSMUST00000213072	ENSMUSG00000031849
mmu_circ_0000526	mmu-miR-7667-5	ENSMUST00000213072	ENSMUSG00000031849
mmu_circ_0000530	mmu-miR-7667-5	ENSMUST00000213072	ENSMUSG00000031849
mmu_circ_0000819	mmu-miR-7667-5	ENSMUST00000213072	ENSMUSG00000031849
mmu_circ_0000819	mmu-miR-7667-5	ENSMUST00000213072	ENSMUSG00000031849
mmu_circ_0000829	mmu-miR-7667-5	ENSMUST00000213072	ENSMUSG00000031849
mmu_circ_0000831	mmu-miR-7667-5	ENSMUST00000213072	ENSMUSG00000031849
mmu_circ_0000668	mmu-miR-7667-5	ENSMUST00000213072	ENSMUSG00000031849
mmu_circ_0000714	mmu-miR-7667-5	ENSMUST00000213072	ENSMUSG00000031849
mmu_circ_0000969	mmu-miR-7667-5	ENSMUST00000213072	ENSMUSG00000031849
mmu_circ_0000844	mmu-miR-7667-5	ENSMUST00000213072	ENSMUSG00000031849
mmu_circ_0000861	mmu-miR-7667-5	ENSMUST00000213072	ENSMUSG00000031849
mmu_circ_0000884	mmu-miR-7667-5	ENSMUST00000213072	ENSMUSG00000031849
mmu_circ_0000886	mmu-miR-7667-5	ENSMUST00000213072	ENSMUSG00000031849
mmu_circ_0000887	mmu-miR-7667-5	ENSMUST00000213072	ENSMUSG00000031849
mmu_circ_0000898	mmu-miR-7667-5	ENSMUST00000213072	ENSMUSG00000031849
mmu_circ_0000903	mmu-miR-7667-5	ENSMUST00000213072	ENSMUSG00000031849
mmu_circ_0000905	mmu-miR-7667-5	ENSMUST00000213072	ENSMUSG00000031849
mmu_circ_0000906	mmu-miR-7667-5	ENSMUST00000213072	ENSMUSG00000031849
mmu_circ_0000907	mmu-miR-7667-5	ENSMUST00000213072	ENSMUSG00000031849
mmu_circ_0000908	mmu-miR-7667-5	ENSMUST00000213072	ENSMUSG00000031849
novel_circ_0001578	mmu-miR-7667-5	ENSMUST00000213072	ENSMUSG00000031849
novel_circ_0001578	mmu-miR-7667-5	ENSMUST00000213072	ENSMUSG00000031849
novel_circ_0001661	mmu-miR-7667-5	ENSMUST00000213072	ENSMUSG00000031849
novel_circ_0001761	mmu-miR-7667-5	ENSMUST00000213072	ENSMUSG00000031849
novel_circ_0001782	mmu-miR-7667-5	ENSMUST00000213072	ENSMUSG00000031849
novel_circ_0001850	mmu-miR-7667-5	ENSMUST00000213072	ENSMUSG00000031849
novel_circ_0001883	mmu-miR-7667-5	ENSMUST00000213072	ENSMUSG00000031849
novel_circ_0001892	mmu-miR-7667-5	ENSMUST00000213072	ENSMUSG00000031849
novel_circ_0002412	mmu-miR-7667-5	ENSMUST00000213072	ENSMUSG00000031849
novel_circ_0002417	mmu-miR-7667-5	ENSMUST00000213072	ENSMUSG00000031849
novel_circ_0002427	mmu-miR-7667-5	ENSMUST00000213072	ENSMUSG00000031849
novel_circ_0002478	mmu-miR-7667-5	ENSMUST00000213072	ENSMUSG00000031849
novel_circ_0002500	mmu-miR-7667-5	ENSMUST00000213072	ENSMUSG00000031849
novel_circ_0002501	mmu-miR-7667-5	ENSMUST00000213072	ENSMUSG00000031849
novel_circ_0002226	mmu-miR-7667-5	ENSMUST00000213072	ENSMUSG00000031849
novel_circ_0002226	mmu-miR-7667-5	ENSMUST00000213072	ENSMUSG00000031849
novel_circ_0002323	mmu-miR-7667-5	ENSMUST00000213072	ENSMUSG00000031849
novel_circ_0002378	mmu-miR-7667-5	ENSMUST00000213072	ENSMUSG00000031849
novel_circ_0002863	mmu-miR-7667-5	ENSMUST00000213072	ENSMUSG00000031849
novel_circ_0002863	mmu-miR-7667-5	ENSMUST00000213072	ENSMUSG00000031849
novel_circ_0002884	mmu-miR-7667-5	ENSMUST00000213072	ENSMUSG00000031849
novel_circ_0002900	mmu-miR-7667-5	ENSMUST00000213072	ENSMUSG00000031849
novel_circ_0003009	mmu-miR-7667-5	ENSMUST00000213072	ENSMUSG00000031849
novel_circ_0002642	mmu-miR-7667-5	ENSMUST00000213072	ENSMUSG00000031849
novel_circ_0002653	mmu-miR-7667-5	ENSMUST00000213072	ENSMUSG00000031849
novel_circ_0002670	mmu-miR-7667-5	ENSMUST00000213072	ENSMUSG00000031849
novel_circ_0002675	mmu-miR-7667-5	ENSMUST00000213072	ENSMUSG00000031849
novel_circ_0002695	mmu-miR-7667-5	ENSMUST00000213072	ENSMUSG00000031849
novel_circ_0002706	mmu-miR-7667-5	ENSMUST00000213072	ENSMUSG00000031849
novel_circ_0002781	mmu-miR-7667-5	ENSMUST00000213072	ENSMUSG00000031849
novel_circ_0003207	mmu-miR-7667-5	ENSMUST00000213072	ENSMUSG00000031849
novel_circ_0003262	mmu-miR-7667-5	ENSMUST00000213072	ENSMUSG00000031849
novel_circ_0003368	mmu-miR-7667-5	ENSMUST00000213072	ENSMUSG00000031849
novel_circ_0003456	mmu-miR-7667-5	ENSMUST00000213072	ENSMUSG00000031849

novel_circ_0003462	mmu-miR-7667-5	ENSMUST00000213072	ENSMUSG00000031849
novel_circ_0000218	mmu-miR-7667-5	ENSMUST00000213072	ENSMUSG00000031849
novel_circ_0000295	mmu-miR-7667-5	ENSMUST00000213072	ENSMUSG00000031849
novel_circ_0000295	mmu-miR-7667-5	ENSMUST00000213072	ENSMUSG00000031849
novel_circ_0000298	mmu-miR-7667-5	ENSMUST00000213072	ENSMUSG00000031849
novel_circ_0000298	mmu-miR-7667-5	ENSMUST00000213072	ENSMUSG00000031849
novel_circ_0000368	mmu-miR-7667-5	ENSMUST00000213072	ENSMUSG00000031849
novel_circ_0000108	mmu-miR-7667-5	ENSMUST00000213072	ENSMUSG00000031849
novel_circ_0000108	mmu-miR-7667-5	ENSMUST00000213072	ENSMUSG00000031849
novel_circ_0000145	mmu-miR-7667-5	ENSMUST00000213072	ENSMUSG00000031849
novel_circ_0000633	mmu-miR-7667-5	ENSMUST00000213072	ENSMUSG00000031849
novel_circ_0000668	mmu-miR-7667-5	ENSMUST00000213072	ENSMUSG00000031849
novel_circ_0000723	mmu-miR-7667-5	ENSMUST00000213072	ENSMUSG00000031849
novel_circ_0000757	mmu-miR-7667-5	ENSMUST00000213072	ENSMUSG00000031849
novel_circ_0000773	mmu-miR-7667-5	ENSMUST00000213072	ENSMUSG00000031849
novel_circ_0000774	mmu-miR-7667-5	ENSMUST00000213072	ENSMUSG00000031849
novel_circ_0000774	mmu-miR-7667-5	ENSMUST00000213072	ENSMUSG00000031849
novel_circ_0000462	mmu-miR-7667-5	ENSMUST00000213072	ENSMUSG00000031849
novel_circ_0000525	mmu-miR-7667-5	ENSMUST00000213072	ENSMUSG00000031849
novel_circ_0000533	mmu-miR-7667-5	ENSMUST00000213072	ENSMUSG00000031849
novel_circ_0000533	mmu-miR-7667-5	ENSMUST00000213072	ENSMUSG00000031849
novel_circ_0000537	mmu-miR-7667-5	ENSMUST00000213072	ENSMUSG00000031849
novel_circ_0000538	mmu-miR-7667-5	ENSMUST00000213072	ENSMUSG00000031849
novel_circ_0000589	mmu-miR-7667-5	ENSMUST00000213072	ENSMUSG00000031849
novel_circ_0000938	mmu-miR-7667-5	ENSMUST00000213072	ENSMUSG00000031849
novel_circ_0000951	mmu-miR-7667-5	ENSMUST00000213072	ENSMUSG00000031849
novel_circ_0001172	mmu-miR-7667-5	ENSMUST00000213072	ENSMUSG00000031849
novel_circ_0001172	mmu-miR-7667-5	ENSMUST00000213072	ENSMUSG00000031849
novel_circ_0001220	mmu-miR-7679-5	ENSMUST00000000128	ENSMUSG00000000126
novel_circ_0001220	mmu-miR-7679-5	ENSMUST00000108783	ENSMUSG00000000126
novel_circ_0001220	mmu-miR-7679-5	ENSMUST00000213072	ENSMUSG00000031849
novel_circ_0001224	mmu-miR-7679-5	ENSMUST00000000128	ENSMUSG00000000126
novel_circ_0001224	mmu-miR-7679-5	ENSMUST00000108783	ENSMUSG00000000126
novel_circ_0001224	mmu-miR-7679-5	ENSMUST00000213072	ENSMUSG00000031849
novel_circ_0001242	mmu-miR-7679-5	ENSMUST00000000128	ENSMUSG00000000126
novel_circ_0001242	mmu-miR-7679-5	ENSMUST00000108783	ENSMUSG00000000126
novel_circ_0001242	mmu-miR-7679-5	ENSMUST00000213072	ENSMUSG00000031849
novel_circ_0001259	mmu-miR-7679-5	ENSMUST00000000128	ENSMUSG00000000126
novel_circ_0001259	mmu-miR-7679-5	ENSMUST00000108783	ENSMUSG00000000126
novel_circ_0001259	mmu-miR-7679-5	ENSMUST00000213072	ENSMUSG00000031849
novel_circ_0001288	mmu-miR-7679-5	ENSMUST00000000128	ENSMUSG00000000126
novel_circ_0001288	mmu-miR-7679-5	ENSMUST00000108783	ENSMUSG00000000126
novel_circ_0001288	mmu-miR-7679-5	ENSMUST00000213072	ENSMUSG00000031849
novel_circ_0001078	mmu-miR-7679-5	ENSMUST00000000128	ENSMUSG00000000126
novel_circ_0001078	mmu-miR-7679-5	ENSMUST00000108783	ENSMUSG00000000126
novel_circ_0001078	mmu-miR-7679-5	ENSMUST00000213072	ENSMUSG00000031849
novel_circ_0001112	mmu-miR-7679-5	ENSMUST00000000128	ENSMUSG00000000126
novel_circ_0001112	mmu-miR-7679-5	ENSMUST00000108783	ENSMUSG00000000126
novel_circ_0001112	mmu-miR-7679-5	ENSMUST00000213072	ENSMUSG00000031849
novel_circ_0001112	mmu-miR-7679-5	ENSMUST00000000128	ENSMUSG00000000126
novel_circ_0001112	mmu-miR-7679-5	ENSMUST00000108783	ENSMUSG00000000126
novel_circ_0001112	mmu-miR-7679-5	ENSMUST00000213072	ENSMUSG00000031849
novel_circ_0001495	mmu-miR-7679-5	ENSMUST00000000128	ENSMUSG00000000126
novel_circ_0001495	mmu-miR-7679-5	ENSMUST00000108783	ENSMUSG00000000126
novel_circ_0001495	mmu-miR-7679-5	ENSMUST00000213072	ENSMUSG00000031849
novel_circ_0001533	mmu-miR-7679-5	ENSMUST00000000128	ENSMUSG00000000126
novel_circ_0001533	mmu-miR-7679-5	ENSMUST00000108783	ENSMUSG00000000126
novel_circ_0001533	mmu-miR-7679-5	ENSMUST00000213072	ENSMUSG00000031849

novel_circ_0001534	mmu-miR-7679-5	ENSMUST00000000128	ENSMUSG00000000126
novel_circ_0001534	mmu-miR-7679-5	ENSMUST00000108783	ENSMUSG00000000126
novel_circ_0001534	mmu-miR-7679-5	ENSMUST00000213072	ENSMUSG00000031849
novel_circ_0001339	mmu-miR-7679-5	ENSMUST00000000128	ENSMUSG00000000126
novel_circ_0001339	mmu-miR-7679-5	ENSMUST00000108783	ENSMUSG00000000126
novel_circ_0001339	mmu-miR-7679-5	ENSMUST00000213072	ENSMUSG00000031849
novel_circ_0001375	mmu-miR-7679-5	ENSMUST00000000128	ENSMUSG00000000126
novel_circ_0001375	mmu-miR-7679-5	ENSMUST00000108783	ENSMUSG00000000126
novel_circ_0001375	mmu-miR-7679-5	ENSMUST00000213072	ENSMUSG00000031849
novel_circ_0001380	mmu-miR-7679-5	ENSMUST00000000128	ENSMUSG00000000126
novel_circ_0001380	mmu-miR-7679-5	ENSMUST00000108783	ENSMUSG00000000126
novel_circ_0001380	mmu-miR-7679-5	ENSMUST00000213072	ENSMUSG00000031849
novel_circ_0001380	mmu-miR-7679-5	ENSMUST00000000128	ENSMUSG00000000126
novel_circ_0001380	mmu-miR-7679-5	ENSMUST00000108783	ENSMUSG00000000126
novel_circ_0001380	mmu-miR-7679-5	ENSMUST00000213072	ENSMUSG00000031849
mmu_circ_0000075	mmu-miR-7679-5	ENSMUST00000000128	ENSMUSG00000000126
mmu_circ_0000075	mmu-miR-7679-5	ENSMUST00000108783	ENSMUSG00000000126
mmu_circ_0000075	mmu-miR-7679-5	ENSMUST00000213072	ENSMUSG00000031849
mmu_circ_0000042	mmu-miR-7679-5	ENSMUST00000000128	ENSMUSG00000000126
mmu_circ_0000042	mmu-miR-7679-5	ENSMUST00000108783	ENSMUSG00000000126
mmu_circ_0000042	mmu-miR-7679-5	ENSMUST00000213072	ENSMUSG00000031849
mmu_circ_0000063	mmu-miR-7679-5	ENSMUST00000000128	ENSMUSG00000000126
mmu_circ_0000063	mmu-miR-7679-5	ENSMUST00000108783	ENSMUSG00000000126
mmu_circ_0000063	mmu-miR-7679-5	ENSMUST00000213072	ENSMUSG00000031849
mmu_circ_0001123	mmu-miR-7679-5	ENSMUST00000000128	ENSMUSG00000000126
mmu_circ_0001123	mmu-miR-7679-5	ENSMUST00000108783	ENSMUSG00000000126
mmu_circ_0001123	mmu-miR-7679-5	ENSMUST00000213072	ENSMUSG00000031849
mmu_circ_0001103	mmu-miR-7679-5	ENSMUST00000000128	ENSMUSG00000000126
mmu_circ_0001103	mmu-miR-7679-5	ENSMUST00000108783	ENSMUSG00000000126
mmu_circ_0001103	mmu-miR-7679-5	ENSMUST00000213072	ENSMUSG00000031849
mmu_circ_0001049	mmu-miR-7679-5	ENSMUST00000000128	ENSMUSG00000000126
mmu_circ_0001049	mmu-miR-7679-5	ENSMUST00000108783	ENSMUSG00000000126
mmu_circ_0001049	mmu-miR-7679-5	ENSMUST00000213072	ENSMUSG00000031849
mmu_circ_0001376	mmu-miR-7679-5	ENSMUST00000000128	ENSMUSG00000000126
mmu_circ_0001376	mmu-miR-7679-5	ENSMUST00000108783	ENSMUSG00000000126
mmu_circ_0001376	mmu-miR-7679-5	ENSMUST00000213072	ENSMUSG00000031849
mmu_circ_0001387	mmu-miR-7679-5	ENSMUST00000000128	ENSMUSG00000000126
mmu_circ_0001387	mmu-miR-7679-5	ENSMUST00000108783	ENSMUSG00000000126
mmu_circ_0001387	mmu-miR-7679-5	ENSMUST00000213072	ENSMUSG00000031849
mmu_circ_0001395	mmu-miR-7679-5	ENSMUST00000000128	ENSMUSG00000000126
mmu_circ_0001395	mmu-miR-7679-5	ENSMUST00000108783	ENSMUSG00000000126
mmu_circ_0001395	mmu-miR-7679-5	ENSMUST00000213072	ENSMUSG00000031849
mmu_circ_0001329	mmu-miR-7679-5	ENSMUST00000000128	ENSMUSG00000000126
mmu_circ_0001329	mmu-miR-7679-5	ENSMUST00000108783	ENSMUSG00000000126
mmu_circ_0001329	mmu-miR-7679-5	ENSMUST00000213072	ENSMUSG00000031849
mmu_circ_0001331	mmu-miR-7679-5	ENSMUST00000000128	ENSMUSG00000000126
mmu_circ_0001331	mmu-miR-7679-5	ENSMUST00000108783	ENSMUSG00000000126
mmu_circ_0001331	mmu-miR-7679-5	ENSMUST00000213072	ENSMUSG00000031849
mmu_circ_0001333	mmu-miR-7679-5	ENSMUST00000000128	ENSMUSG00000000126
mmu_circ_0001333	mmu-miR-7679-5	ENSMUST00000108783	ENSMUSG00000000126
mmu_circ_0001333	mmu-miR-7679-5	ENSMUST00000213072	ENSMUSG00000031849
mmu_circ_0001248	mmu-miR-7679-5	ENSMUST00000000128	ENSMUSG00000000126
mmu_circ_0001248	mmu-miR-7679-5	ENSMUST00000108783	ENSMUSG00000000126
mmu_circ_0001248	mmu-miR-7679-5	ENSMUST00000213072	ENSMUSG00000031849
mmu_circ_0001249	mmu-miR-7679-5	ENSMUST00000000128	ENSMUSG00000000126
mmu_circ_0001249	mmu-miR-7679-5	ENSMUST00000108783	ENSMUSG00000000126
mmu_circ_0001249	mmu-miR-7679-5	ENSMUST00000213072	ENSMUSG00000031849
mmu_circ_0001279	mmu-miR-7679-5	ENSMUST00000000128	ENSMUSG00000000126

mmu_circ_0001279	mmu-miR-7679-5	ENSMUST00000108783	ENSMUSG00000000126
mmu_circ_0001279	mmu-miR-7679-5	ENSMUST00000213072	ENSMUSG00000031849
mmu_circ_0001279	mmu-miR-7679-5	ENSMUST00000000128	ENSMUSG00000000126
mmu_circ_0001279	mmu-miR-7679-5	ENSMUST00000108783	ENSMUSG00000000126
mmu_circ_0001279	mmu-miR-7679-5	ENSMUST00000213072	ENSMUSG00000031849
mmu_circ_0001283	mmu-miR-7679-5	ENSMUST00000000128	ENSMUSG00000000126
mmu_circ_0001283	mmu-miR-7679-5	ENSMUST00000108783	ENSMUSG00000000126
mmu_circ_0001283	mmu-miR-7679-5	ENSMUST00000213072	ENSMUSG00000031849
mmu_circ_0001505	mmu-miR-7679-5	ENSMUST00000000128	ENSMUSG00000000126
mmu_circ_0001505	mmu-miR-7679-5	ENSMUST00000108783	ENSMUSG00000000126
mmu_circ_0001505	mmu-miR-7679-5	ENSMUST00000213072	ENSMUSG00000031849
mmu_circ_0001454	mmu-miR-7679-5	ENSMUST00000000128	ENSMUSG00000000126
mmu_circ_0001454	mmu-miR-7679-5	ENSMUST00000108783	ENSMUSG00000000126
mmu_circ_0001454	mmu-miR-7679-5	ENSMUST00000213072	ENSMUSG00000031849
mmu_circ_0001496	mmu-miR-7679-5	ENSMUST00000000128	ENSMUSG00000000126
mmu_circ_0001496	mmu-miR-7679-5	ENSMUST00000108783	ENSMUSG00000000126
mmu_circ_0001496	mmu-miR-7679-5	ENSMUST00000213072	ENSMUSG00000031849
mmu_circ_0001500	mmu-miR-7679-5	ENSMUST00000000128	ENSMUSG00000000126
mmu_circ_0001500	mmu-miR-7679-5	ENSMUST00000108783	ENSMUSG00000000126
mmu_circ_0001500	mmu-miR-7679-5	ENSMUST00000213072	ENSMUSG00000031849
mmu_circ_0001775	mmu-miR-7679-5	ENSMUST00000000128	ENSMUSG00000000126
mmu_circ_0001775	mmu-miR-7679-5	ENSMUST00000108783	ENSMUSG00000000126
mmu_circ_0001775	mmu-miR-7679-5	ENSMUST00000213072	ENSMUSG00000031849
mmu_circ_0001795	mmu-miR-7679-5	ENSMUST00000000128	ENSMUSG00000000126
mmu_circ_0001795	mmu-miR-7679-5	ENSMUST00000108783	ENSMUSG00000000126
mmu_circ_0001795	mmu-miR-7679-5	ENSMUST00000213072	ENSMUSG00000031849
mmu_circ_0001827	mmu-miR-7679-5	ENSMUST00000000128	ENSMUSG00000000126
mmu_circ_0001827	mmu-miR-7679-5	ENSMUST00000108783	ENSMUSG00000000126
mmu_circ_0001827	mmu-miR-7679-5	ENSMUST00000213072	ENSMUSG00000031849
mmu_circ_0000271	mmu-miR-7679-5	ENSMUST00000000128	ENSMUSG00000000126
mmu_circ_0000271	mmu-miR-7679-5	ENSMUST00000108783	ENSMUSG00000000126
mmu_circ_0000271	mmu-miR-7679-5	ENSMUST00000213072	ENSMUSG00000031849
mmu_circ_0000230	mmu-miR-7679-5	ENSMUST00000000128	ENSMUSG00000000126
mmu_circ_0000230	mmu-miR-7679-5	ENSMUST00000108783	ENSMUSG00000000126
mmu_circ_0000230	mmu-miR-7679-5	ENSMUST00000213072	ENSMUSG00000031849
mmu_circ_0000499	mmu-miR-7679-5	ENSMUST00000000128	ENSMUSG00000000126
mmu_circ_0000499	mmu-miR-7679-5	ENSMUST00000108783	ENSMUSG00000000126
mmu_circ_0000499	mmu-miR-7679-5	ENSMUST00000213072	ENSMUSG00000031849
mmu_circ_0000399	mmu-miR-7679-5	ENSMUST00000000128	ENSMUSG00000000126
mmu_circ_0000399	mmu-miR-7679-5	ENSMUST00000108783	ENSMUSG00000000126
mmu_circ_0000399	mmu-miR-7679-5	ENSMUST00000213072	ENSMUSG00000031849
mmu_circ_0000628	mmu-miR-7679-5	ENSMUST00000000128	ENSMUSG00000000126
mmu_circ_0000628	mmu-miR-7679-5	ENSMUST00000108783	ENSMUSG00000000126
mmu_circ_0000628	mmu-miR-7679-5	ENSMUST00000213072	ENSMUSG00000031849
mmu_circ_0000530	mmu-miR-7679-5	ENSMUST00000000128	ENSMUSG00000000126
mmu_circ_0000530	mmu-miR-7679-5	ENSMUST00000108783	ENSMUSG00000000126
mmu_circ_0000530	mmu-miR-7679-5	ENSMUST00000213072	ENSMUSG00000031849
mmu_circ_0000738	mmu-miR-7679-5	ENSMUST00000000128	ENSMUSG00000000126
mmu_circ_0000738	mmu-miR-7679-5	ENSMUST00000108783	ENSMUSG00000000126
mmu_circ_0000738	mmu-miR-7679-5	ENSMUST00000213072	ENSMUSG00000031849
mmu_circ_0000803	mmu-miR-7679-5	ENSMUST00000000128	ENSMUSG00000000126
mmu_circ_0000803	mmu-miR-7679-5	ENSMUST00000108783	ENSMUSG00000000126
mmu_circ_0000803	mmu-miR-7679-5	ENSMUST00000213072	ENSMUSG00000031849
mmu_circ_0000804	mmu-miR-7679-5	ENSMUST00000000128	ENSMUSG00000000126
mmu_circ_0000804	mmu-miR-7679-5	ENSMUST00000108783	ENSMUSG00000000126
mmu_circ_0000804	mmu-miR-7679-5	ENSMUST00000213072	ENSMUSG00000031849
mmu_circ_0000829	mmu-miR-7679-5	ENSMUST00000000128	ENSMUSG00000000126
mmu_circ_0000829	mmu-miR-7679-5	ENSMUST00000108783	ENSMUSG00000000126

mmu_circ_0000829	mmu-miR-7679-5	ENSMUST00000213072	ENSMUSG00000031849
mmu_circ_0000658	mmu-miR-7679-5	ENSMUST00000000128	ENSMUSG00000000126
mmu_circ_0000658	mmu-miR-7679-5	ENSMUST00000108783	ENSMUSG00000000126
mmu_circ_0000658	mmu-miR-7679-5	ENSMUST00000213072	ENSMUSG00000031849
mmu_circ_0000659	mmu-miR-7679-5	ENSMUST00000000128	ENSMUSG00000000126
mmu_circ_0000659	mmu-miR-7679-5	ENSMUST00000108783	ENSMUSG00000000126
mmu_circ_0000659	mmu-miR-7679-5	ENSMUST00000213072	ENSMUSG00000031849
mmu_circ_0000711	mmu-miR-7679-5	ENSMUST00000000128	ENSMUSG00000000126
mmu_circ_0000711	mmu-miR-7679-5	ENSMUST00000108783	ENSMUSG00000000126
mmu_circ_0000711	mmu-miR-7679-5	ENSMUST00000213072	ENSMUSG00000031849
mmu_circ_0000950	mmu-miR-7679-5	ENSMUST00000000128	ENSMUSG00000000126
mmu_circ_0000950	mmu-miR-7679-5	ENSMUST00000108783	ENSMUSG00000000126
mmu_circ_0000950	mmu-miR-7679-5	ENSMUST00000213072	ENSMUSG00000031849
mmu_circ_0000919	mmu-miR-7679-5	ENSMUST00000000128	ENSMUSG00000000126
mmu_circ_0000919	mmu-miR-7679-5	ENSMUST00000108783	ENSMUSG00000000126
mmu_circ_0000919	mmu-miR-7679-5	ENSMUST00000213072	ENSMUSG00000031849
mmu_circ_0000921	mmu-miR-7679-5	ENSMUST00000000128	ENSMUSG00000000126
mmu_circ_0000921	mmu-miR-7679-5	ENSMUST00000108783	ENSMUSG00000000126
mmu_circ_0000921	mmu-miR-7679-5	ENSMUST00000213072	ENSMUSG00000031849
mmu_circ_0000962	mmu-miR-7679-5	ENSMUST00000000128	ENSMUSG00000000126
mmu_circ_0000962	mmu-miR-7679-5	ENSMUST00000108783	ENSMUSG00000000126
mmu_circ_0000962	mmu-miR-7679-5	ENSMUST00000213072	ENSMUSG00000031849
mmu_circ_0000969	mmu-miR-7679-5	ENSMUST00000000128	ENSMUSG00000000126
mmu_circ_0000969	mmu-miR-7679-5	ENSMUST00000108783	ENSMUSG00000000126
mmu_circ_0000969	mmu-miR-7679-5	ENSMUST00000213072	ENSMUSG00000031849
mmu_circ_0000884	mmu-miR-7679-5	ENSMUST00000000128	ENSMUSG00000000126
mmu_circ_0000884	mmu-miR-7679-5	ENSMUST00000108783	ENSMUSG00000000126
mmu_circ_0000884	mmu-miR-7679-5	ENSMUST00000213072	ENSMUSG00000031849
mmu_circ_0000886	mmu-miR-7679-5	ENSMUST00000000128	ENSMUSG00000000126
mmu_circ_0000886	mmu-miR-7679-5	ENSMUST00000108783	ENSMUSG00000000126
mmu_circ_0000886	mmu-miR-7679-5	ENSMUST00000213072	ENSMUSG00000031849
mmu_circ_0000887	mmu-miR-7679-5	ENSMUST00000000128	ENSMUSG00000000126
mmu_circ_0000887	mmu-miR-7679-5	ENSMUST00000108783	ENSMUSG00000000126
mmu_circ_0000887	mmu-miR-7679-5	ENSMUST00000213072	ENSMUSG00000031849
mmu_circ_0000906	mmu-miR-7679-5	ENSMUST00000000128	ENSMUSG00000000126
mmu_circ_0000906	mmu-miR-7679-5	ENSMUST00000108783	ENSMUSG00000000126
mmu_circ_0000906	mmu-miR-7679-5	ENSMUST00000213072	ENSMUSG00000031849
mmu_circ_0000907	mmu-miR-7679-5	ENSMUST00000000128	ENSMUSG00000000126
mmu_circ_0000907	mmu-miR-7679-5	ENSMUST00000108783	ENSMUSG00000000126
mmu_circ_0000907	mmu-miR-7679-5	ENSMUST00000213072	ENSMUSG00000031849
novel_circ_0001561	mmu-miR-7679-5	ENSMUST00000000128	ENSMUSG00000000126
novel_circ_0001561	mmu-miR-7679-5	ENSMUST00000108783	ENSMUSG00000000126
novel_circ_0001561	mmu-miR-7679-5	ENSMUST00000213072	ENSMUSG00000031849
novel_circ_0001564	mmu-miR-7679-5	ENSMUST00000000128	ENSMUSG00000000126
novel_circ_0001564	mmu-miR-7679-5	ENSMUST00000108783	ENSMUSG00000000126
novel_circ_0001564	mmu-miR-7679-5	ENSMUST00000213072	ENSMUSG00000031849
novel_circ_0001579	mmu-miR-7679-5	ENSMUST00000000128	ENSMUSG00000000126
novel_circ_0001579	mmu-miR-7679-5	ENSMUST00000108783	ENSMUSG00000000126
novel_circ_0001579	mmu-miR-7679-5	ENSMUST00000213072	ENSMUSG00000031849
novel_circ_0001619	mmu-miR-7679-5	ENSMUST00000000128	ENSMUSG00000000126
novel_circ_0001619	mmu-miR-7679-5	ENSMUST00000108783	ENSMUSG00000000126
novel_circ_0001619	mmu-miR-7679-5	ENSMUST00000213072	ENSMUSG00000031849
novel_circ_0001736	mmu-miR-7679-5	ENSMUST00000000128	ENSMUSG00000000126
novel_circ_0001736	mmu-miR-7679-5	ENSMUST00000108783	ENSMUSG00000000126
novel_circ_0001736	mmu-miR-7679-5	ENSMUST00000213072	ENSMUSG00000031849
novel_circ_0002124	mmu-miR-7679-5	ENSMUST00000000128	ENSMUSG00000000126
novel_circ_0002124	mmu-miR-7679-5	ENSMUST00000108783	ENSMUSG00000000126
novel_circ_0002124	mmu-miR-7679-5	ENSMUST00000213072	ENSMUSG00000031849

novel_circ_0002167	mmu-miR-7679-5	ENSMUST00000000128	ENSMUSG00000000126
novel_circ_0002167	mmu-miR-7679-5	ENSMUST00000108783	ENSMUSG00000000126
novel_circ_0002167	mmu-miR-7679-5	ENSMUST00000213072	ENSMUSG00000031849
novel_circ_0002216	mmu-miR-7679-5	ENSMUST00000000128	ENSMUSG00000000126
novel_circ_0002216	mmu-miR-7679-5	ENSMUST00000108783	ENSMUSG00000000126
novel_circ_0002216	mmu-miR-7679-5	ENSMUST00000213072	ENSMUSG00000031849
novel_circ_0001875	mmu-miR-7679-5	ENSMUST00000000128	ENSMUSG00000000126
novel_circ_0001875	mmu-miR-7679-5	ENSMUST00000108783	ENSMUSG00000000126
novel_circ_0001875	mmu-miR-7679-5	ENSMUST00000213072	ENSMUSG00000031849
novel_circ_0001888	mmu-miR-7679-5	ENSMUST00000000128	ENSMUSG00000000126
novel_circ_0001888	mmu-miR-7679-5	ENSMUST00000108783	ENSMUSG00000000126
novel_circ_0001888	mmu-miR-7679-5	ENSMUST00000213072	ENSMUSG00000031849
novel_circ_0001893	mmu-miR-7679-5	ENSMUST00000000128	ENSMUSG00000000126
novel_circ_0001893	mmu-miR-7679-5	ENSMUST00000108783	ENSMUSG00000000126
novel_circ_0001893	mmu-miR-7679-5	ENSMUST00000213072	ENSMUSG00000031849
novel_circ_0001893	mmu-miR-7679-5	ENSMUST00000000128	ENSMUSG00000000126
novel_circ_0001893	mmu-miR-7679-5	ENSMUST00000108783	ENSMUSG00000000126
novel_circ_0001893	mmu-miR-7679-5	ENSMUST00000213072	ENSMUSG00000031849
novel_circ_0001913	mmu-miR-7679-5	ENSMUST00000000128	ENSMUSG00000000126
novel_circ_0001913	mmu-miR-7679-5	ENSMUST00000108783	ENSMUSG00000000126
novel_circ_0001913	mmu-miR-7679-5	ENSMUST00000213072	ENSMUSG00000031849
novel_circ_0001935	mmu-miR-7679-5	ENSMUST00000000128	ENSMUSG00000000126
novel_circ_0001935	mmu-miR-7679-5	ENSMUST00000108783	ENSMUSG00000000126
novel_circ_0001935	mmu-miR-7679-5	ENSMUST00000213072	ENSMUSG00000031849
novel_circ_0001945	mmu-miR-7679-5	ENSMUST00000000128	ENSMUSG00000000126
novel_circ_0001945	mmu-miR-7679-5	ENSMUST00000108783	ENSMUSG00000000126
novel_circ_0001945	mmu-miR-7679-5	ENSMUST00000213072	ENSMUSG00000031849
novel_circ_0001946	mmu-miR-7679-5	ENSMUST00000000128	ENSMUSG00000000126
novel_circ_0001946	mmu-miR-7679-5	ENSMUST00000108783	ENSMUSG00000000126
novel_circ_0001946	mmu-miR-7679-5	ENSMUST00000213072	ENSMUSG00000031849
novel_circ_0001954	mmu-miR-7679-5	ENSMUST00000000128	ENSMUSG00000000126
novel_circ_0001954	mmu-miR-7679-5	ENSMUST00000108783	ENSMUSG00000000126
novel_circ_0001954	mmu-miR-7679-5	ENSMUST00000213072	ENSMUSG00000031849
novel_circ_0001992	mmu-miR-7679-5	ENSMUST00000000128	ENSMUSG00000000126
novel_circ_0001992	mmu-miR-7679-5	ENSMUST00000108783	ENSMUSG00000000126
novel_circ_0001992	mmu-miR-7679-5	ENSMUST00000213072	ENSMUSG00000031849
novel_circ_0002030	mmu-miR-7679-5	ENSMUST00000000128	ENSMUSG00000000126
novel_circ_0002030	mmu-miR-7679-5	ENSMUST00000108783	ENSMUSG00000000126
novel_circ_0002030	mmu-miR-7679-5	ENSMUST00000213072	ENSMUSG00000031849
novel_circ_0002450	mmu-miR-7679-5	ENSMUST00000000128	ENSMUSG00000000126
novel_circ_0002450	mmu-miR-7679-5	ENSMUST00000108783	ENSMUSG00000000126
novel_circ_0002450	mmu-miR-7679-5	ENSMUST00000213072	ENSMUSG00000031849
novel_circ_0002451	mmu-miR-7679-5	ENSMUST00000000128	ENSMUSG00000000126
novel_circ_0002451	mmu-miR-7679-5	ENSMUST00000108783	ENSMUSG00000000126
novel_circ_0002451	mmu-miR-7679-5	ENSMUST00000213072	ENSMUSG00000031849
novel_circ_0002494	mmu-miR-7679-5	ENSMUST00000000128	ENSMUSG00000000126
novel_circ_0002494	mmu-miR-7679-5	ENSMUST00000108783	ENSMUSG00000000126
novel_circ_0002494	mmu-miR-7679-5	ENSMUST00000213072	ENSMUSG00000031849
novel_circ_0002499	mmu-miR-7679-5	ENSMUST00000000128	ENSMUSG00000000126
novel_circ_0002499	mmu-miR-7679-5	ENSMUST00000108783	ENSMUSG00000000126
novel_circ_0002499	mmu-miR-7679-5	ENSMUST00000213072	ENSMUSG00000031849
novel_circ_0002500	mmu-miR-7679-5	ENSMUST00000000128	ENSMUSG00000000126
novel_circ_0002500	mmu-miR-7679-5	ENSMUST00000108783	ENSMUSG00000000126
novel_circ_0002500	mmu-miR-7679-5	ENSMUST00000213072	ENSMUSG00000031849
novel_circ_0002501	mmu-miR-7679-5	ENSMUST00000000128	ENSMUSG00000000126
novel_circ_0002501	mmu-miR-7679-5	ENSMUST00000108783	ENSMUSG00000000126
novel_circ_0002501	mmu-miR-7679-5	ENSMUST00000213072	ENSMUSG00000031849
novel_circ_0002501	mmu-miR-7679-5	ENSMUST00000000128	ENSMUSG00000000126

novel_circ_0002501	mmu-miR-7679-5	ENSMUST00000108783	ENSMUSG00000000126
novel_circ_0002501	mmu-miR-7679-5	ENSMUST00000213072	ENSMUSG00000031849
novel_circ_0002503	mmu-miR-7679-5	ENSMUST00000000128	ENSMUSG00000000126
novel_circ_0002503	mmu-miR-7679-5	ENSMUST00000108783	ENSMUSG00000000126
novel_circ_0002503	mmu-miR-7679-5	ENSMUST00000213072	ENSMUSG00000031849
novel_circ_0002504	mmu-miR-7679-5	ENSMUST00000000128	ENSMUSG00000000126
novel_circ_0002504	mmu-miR-7679-5	ENSMUST00000108783	ENSMUSG00000000126
novel_circ_0002504	mmu-miR-7679-5	ENSMUST00000213072	ENSMUSG00000031849
novel_circ_0002522	mmu-miR-7679-5	ENSMUST00000000128	ENSMUSG00000000126
novel_circ_0002522	mmu-miR-7679-5	ENSMUST00000108783	ENSMUSG00000000126
novel_circ_0002522	mmu-miR-7679-5	ENSMUST00000213072	ENSMUSG00000031849
novel_circ_0002565	mmu-miR-7679-5	ENSMUST00000000128	ENSMUSG00000000126
novel_circ_0002565	mmu-miR-7679-5	ENSMUST00000108783	ENSMUSG00000000126
novel_circ_0002565	mmu-miR-7679-5	ENSMUST00000213072	ENSMUSG00000031849
novel_circ_0002578	mmu-miR-7679-5	ENSMUST00000000128	ENSMUSG00000000126
novel_circ_0002578	mmu-miR-7679-5	ENSMUST00000108783	ENSMUSG00000000126
novel_circ_0002578	mmu-miR-7679-5	ENSMUST00000213072	ENSMUSG00000031849
novel_circ_0002288	mmu-miR-7679-5	ENSMUST00000000128	ENSMUSG00000000126
novel_circ_0002288	mmu-miR-7679-5	ENSMUST00000108783	ENSMUSG00000000126
novel_circ_0002288	mmu-miR-7679-5	ENSMUST00000213072	ENSMUSG00000031849
novel_circ_0002367	mmu-miR-7679-5	ENSMUST00000000128	ENSMUSG00000000126
novel_circ_0002367	mmu-miR-7679-5	ENSMUST00000108783	ENSMUSG00000000126
novel_circ_0002367	mmu-miR-7679-5	ENSMUST00000213072	ENSMUSG00000031849
novel_circ_0002368	mmu-miR-7679-5	ENSMUST00000000128	ENSMUSG00000000126
novel_circ_0002368	mmu-miR-7679-5	ENSMUST00000108783	ENSMUSG00000000126
novel_circ_0002368	mmu-miR-7679-5	ENSMUST00000213072	ENSMUSG00000031849
novel_circ_0002826	mmu-miR-7679-5	ENSMUST00000000128	ENSMUSG00000000126
novel_circ_0002826	mmu-miR-7679-5	ENSMUST00000108783	ENSMUSG00000000126
novel_circ_0002826	mmu-miR-7679-5	ENSMUST00000213072	ENSMUSG00000031849
novel_circ_0002831	mmu-miR-7679-5	ENSMUST00000000128	ENSMUSG00000000126
novel_circ_0002831	mmu-miR-7679-5	ENSMUST00000108783	ENSMUSG00000000126
novel_circ_0002831	mmu-miR-7679-5	ENSMUST00000213072	ENSMUSG00000031849
novel_circ_0002835	mmu-miR-7679-5	ENSMUST00000000128	ENSMUSG00000000126
novel_circ_0002835	mmu-miR-7679-5	ENSMUST00000108783	ENSMUSG00000000126
novel_circ_0002835	mmu-miR-7679-5	ENSMUST00000213072	ENSMUSG00000031849
novel_circ_0002836	mmu-miR-7679-5	ENSMUST00000000128	ENSMUSG00000000126
novel_circ_0002836	mmu-miR-7679-5	ENSMUST00000108783	ENSMUSG00000000126
novel_circ_0002836	mmu-miR-7679-5	ENSMUST00000213072	ENSMUSG00000031849
novel_circ_0002853	mmu-miR-7679-5	ENSMUST00000000128	ENSMUSG00000000126
novel_circ_0002853	mmu-miR-7679-5	ENSMUST00000108783	ENSMUSG00000000126
novel_circ_0002853	mmu-miR-7679-5	ENSMUST00000213072	ENSMUSG00000031849
novel_circ_0002883	mmu-miR-7679-5	ENSMUST00000000128	ENSMUSG00000000126
novel_circ_0002883	mmu-miR-7679-5	ENSMUST00000108783	ENSMUSG00000000126
novel_circ_0002883	mmu-miR-7679-5	ENSMUST00000213072	ENSMUSG00000031849
novel_circ_0002898	mmu-miR-7679-5	ENSMUST00000000128	ENSMUSG00000000126
novel_circ_0002898	mmu-miR-7679-5	ENSMUST00000108783	ENSMUSG00000000126
novel_circ_0002898	mmu-miR-7679-5	ENSMUST00000213072	ENSMUSG00000031849
novel_circ_0002998	mmu-miR-7679-5	ENSMUST00000000128	ENSMUSG00000000126
novel_circ_0002998	mmu-miR-7679-5	ENSMUST00000108783	ENSMUSG00000000126
novel_circ_0002998	mmu-miR-7679-5	ENSMUST00000213072	ENSMUSG00000031849
novel_circ_0002732	mmu-miR-7679-5	ENSMUST00000000128	ENSMUSG00000000126
novel_circ_0002732	mmu-miR-7679-5	ENSMUST00000108783	ENSMUSG00000000126
novel_circ_0002732	mmu-miR-7679-5	ENSMUST00000213072	ENSMUSG00000031849
novel_circ_0003177	mmu-miR-7679-5	ENSMUST00000000128	ENSMUSG00000000126
novel_circ_0003177	mmu-miR-7679-5	ENSMUST00000108783	ENSMUSG00000000126
novel_circ_0003177	mmu-miR-7679-5	ENSMUST00000213072	ENSMUSG00000031849
novel_circ_0003191	mmu-miR-7679-5	ENSMUST00000000128	ENSMUSG00000000126
novel_circ_0003191	mmu-miR-7679-5	ENSMUST00000108783	ENSMUSG00000000126

novel_circ_0003191	mmu-miR-7679-5	ENSMUST00000213072	ENSMUSG00000031849
novel_circ_0003195	mmu-miR-7679-5	ENSMUST00000000128	ENSMUSG00000000126
novel_circ_0003195	mmu-miR-7679-5	ENSMUST00000108783	ENSMUSG00000000126
novel_circ_0003195	mmu-miR-7679-5	ENSMUST00000213072	ENSMUSG00000031849
novel_circ_0003195	mmu-miR-7679-5	ENSMUST00000000128	ENSMUSG00000000126
novel_circ_0003195	mmu-miR-7679-5	ENSMUST00000108783	ENSMUSG00000000126
novel_circ_0003195	mmu-miR-7679-5	ENSMUST00000213072	ENSMUSG00000031849
novel_circ_0003029	mmu-miR-7679-5	ENSMUST00000000128	ENSMUSG00000000126
novel_circ_0003029	mmu-miR-7679-5	ENSMUST00000108783	ENSMUSG00000000126
novel_circ_0003029	mmu-miR-7679-5	ENSMUST00000213072	ENSMUSG00000031849
novel_circ_0003032	mmu-miR-7679-5	ENSMUST00000000128	ENSMUSG00000000126
novel_circ_0003032	mmu-miR-7679-5	ENSMUST00000108783	ENSMUSG00000000126
novel_circ_0003032	mmu-miR-7679-5	ENSMUST00000213072	ENSMUSG00000031849
novel_circ_0003045	mmu-miR-7679-5	ENSMUST00000000128	ENSMUSG00000000126
novel_circ_0003045	mmu-miR-7679-5	ENSMUST00000108783	ENSMUSG00000000126
novel_circ_0003045	mmu-miR-7679-5	ENSMUST00000213072	ENSMUSG00000031849
novel_circ_0003045	mmu-miR-7679-5	ENSMUST00000000128	ENSMUSG00000000126
novel_circ_0003045	mmu-miR-7679-5	ENSMUST00000108783	ENSMUSG00000000126
novel_circ_0003045	mmu-miR-7679-5	ENSMUST00000213072	ENSMUSG00000031849
novel_circ_0003122	mmu-miR-7679-5	ENSMUST00000000128	ENSMUSG00000000126
novel_circ_0003122	mmu-miR-7679-5	ENSMUST00000108783	ENSMUSG00000000126
novel_circ_0003122	mmu-miR-7679-5	ENSMUST00000213072	ENSMUSG00000031849
novel_circ_0003457	mmu-miR-7679-5	ENSMUST00000000128	ENSMUSG00000000126
novel_circ_0003457	mmu-miR-7679-5	ENSMUST00000108783	ENSMUSG00000000126
novel_circ_0003457	mmu-miR-7679-5	ENSMUST00000213072	ENSMUSG00000031849
novel_circ_0000200	mmu-miR-7679-5	ENSMUST00000000128	ENSMUSG00000000126
novel_circ_0000200	mmu-miR-7679-5	ENSMUST00000108783	ENSMUSG00000000126
novel_circ_0000200	mmu-miR-7679-5	ENSMUST00000213072	ENSMUSG00000031849
novel_circ_0000206	mmu-miR-7679-5	ENSMUST00000000128	ENSMUSG00000000126
novel_circ_0000206	mmu-miR-7679-5	ENSMUST00000108783	ENSMUSG00000000126
novel_circ_0000206	mmu-miR-7679-5	ENSMUST00000213072	ENSMUSG00000031849
novel_circ_0000344	mmu-miR-7679-5	ENSMUST00000000128	ENSMUSG00000000126
novel_circ_0000344	mmu-miR-7679-5	ENSMUST00000108783	ENSMUSG00000000126
novel_circ_0000344	mmu-miR-7679-5	ENSMUST00000213072	ENSMUSG00000031849
novel_circ_0000344	mmu-miR-7679-5	ENSMUST00000000128	ENSMUSG00000000126
novel_circ_0000344	mmu-miR-7679-5	ENSMUST00000108783	ENSMUSG00000000126
novel_circ_0000344	mmu-miR-7679-5	ENSMUST00000213072	ENSMUSG00000031849
novel_circ_0000441	mmu-miR-7679-5	ENSMUST00000000128	ENSMUSG00000000126
novel_circ_0000441	mmu-miR-7679-5	ENSMUST00000108783	ENSMUSG00000000126
novel_circ_0000441	mmu-miR-7679-5	ENSMUST00000213072	ENSMUSG00000031849
novel_circ_0000129	mmu-miR-7679-5	ENSMUST00000000128	ENSMUSG00000000126
novel_circ_0000129	mmu-miR-7679-5	ENSMUST00000108783	ENSMUSG00000000126
novel_circ_0000129	mmu-miR-7679-5	ENSMUST00000213072	ENSMUSG00000031849
novel_circ_0000141	mmu-miR-7679-5	ENSMUST00000000128	ENSMUSG00000000126
novel_circ_0000141	mmu-miR-7679-5	ENSMUST00000108783	ENSMUSG00000000126
novel_circ_0000141	mmu-miR-7679-5	ENSMUST00000213072	ENSMUSG00000031849
novel_circ_0000148	mmu-miR-7679-5	ENSMUST00000000128	ENSMUSG00000000126
novel_circ_0000148	mmu-miR-7679-5	ENSMUST00000108783	ENSMUSG00000000126
novel_circ_0000148	mmu-miR-7679-5	ENSMUST00000213072	ENSMUSG00000031849
novel_circ_0000663	mmu-miR-7679-5	ENSMUST00000000128	ENSMUSG00000000126
novel_circ_0000663	mmu-miR-7679-5	ENSMUST00000108783	ENSMUSG00000000126
novel_circ_0000663	mmu-miR-7679-5	ENSMUST00000213072	ENSMUSG00000031849
novel_circ_0000668	mmu-miR-7679-5	ENSMUST00000000128	ENSMUSG00000000126
novel_circ_0000668	mmu-miR-7679-5	ENSMUST00000108783	ENSMUSG00000000126
novel_circ_0000668	mmu-miR-7679-5	ENSMUST00000213072	ENSMUSG00000031849
novel_circ_0000698	mmu-miR-7679-5	ENSMUST00000000128	ENSMUSG00000000126
novel_circ_0000698	mmu-miR-7679-5	ENSMUST00000108783	ENSMUSG00000000126
novel_circ_0000698	mmu-miR-7679-5	ENSMUST00000213072	ENSMUSG00000031849

novel_circ_0000475	mmu-miR-7679-5	ENSMUST00000000128	ENSMUSG00000000126
novel_circ_0000475	mmu-miR-7679-5	ENSMUST00000108783	ENSMUSG00000000126
novel_circ_0000475	mmu-miR-7679-5	ENSMUST00000213072	ENSMUSG00000031849
novel_circ_0000559	mmu-miR-7679-5	ENSMUST00000000128	ENSMUSG00000000126
novel_circ_0000559	mmu-miR-7679-5	ENSMUST00000108783	ENSMUSG00000000126
novel_circ_0000559	mmu-miR-7679-5	ENSMUST00000213072	ENSMUSG00000031849
novel_circ_0000559	mmu-miR-7679-5	ENSMUST00000000128	ENSMUSG00000000126
novel_circ_0000559	mmu-miR-7679-5	ENSMUST00000108783	ENSMUSG00000000126
novel_circ_0000559	mmu-miR-7679-5	ENSMUST00000213072	ENSMUSG00000031849
novel_circ_0000589	mmu-miR-7679-5	ENSMUST00000000128	ENSMUSG00000000126
novel_circ_0000589	mmu-miR-7679-5	ENSMUST00000108783	ENSMUSG00000000126
novel_circ_0000589	mmu-miR-7679-5	ENSMUST00000213072	ENSMUSG00000031849
novel_circ_0001010	mmu-miR-7679-5	ENSMUST00000000128	ENSMUSG00000000126
novel_circ_0001010	mmu-miR-7679-5	ENSMUST00000108783	ENSMUSG00000000126
novel_circ_0001010	mmu-miR-7679-5	ENSMUST00000213072	ENSMUSG00000031849
novel_circ_0001018	mmu-miR-7679-5	ENSMUST00000000128	ENSMUSG00000000126
novel_circ_0001018	mmu-miR-7679-5	ENSMUST00000108783	ENSMUSG00000000126
novel_circ_0001018	mmu-miR-7679-5	ENSMUST00000213072	ENSMUSG00000031849
novel_circ_0001037	mmu-miR-7679-5	ENSMUST00000000128	ENSMUSG00000000126
novel_circ_0001037	mmu-miR-7679-5	ENSMUST00000108783	ENSMUSG00000000126
novel_circ_0001037	mmu-miR-7679-5	ENSMUST00000213072	ENSMUSG00000031849
novel_circ_0001039	mmu-miR-7679-5	ENSMUST00000000128	ENSMUSG00000000126
novel_circ_0001039	mmu-miR-7679-5	ENSMUST00000108783	ENSMUSG00000000126
novel_circ_0001039	mmu-miR-7679-5	ENSMUST00000213072	ENSMUSG00000031849
novel_circ_0001245	mmu-miR-497b	ENSMUST00000003659	ENSMUSG00000031849
novel_circ_0001245	mmu-miR-497b	ENSMUST00000212488	ENSMUSG00000031849
novel_circ_0001251	mmu-miR-497b	ENSMUST00000003659	ENSMUSG00000031849
novel_circ_0001251	mmu-miR-497b	ENSMUST00000212488	ENSMUSG00000031849
novel_circ_0001277	mmu-miR-497b	ENSMUST00000003659	ENSMUSG00000031849
novel_circ_0001277	mmu-miR-497b	ENSMUST00000212488	ENSMUSG00000031849
novel_circ_0001292	mmu-miR-497b	ENSMUST00000003659	ENSMUSG00000031849
novel_circ_0001292	mmu-miR-497b	ENSMUST00000212488	ENSMUSG00000031849
novel_circ_0001084	mmu-miR-497b	ENSMUST00000003659	ENSMUSG00000031849
novel_circ_0001084	mmu-miR-497b	ENSMUST00000212488	ENSMUSG00000031849
novel_circ_0001114	mmu-miR-497b	ENSMUST00000003659	ENSMUSG00000031849
novel_circ_0001114	mmu-miR-497b	ENSMUST00000212488	ENSMUSG00000031849
novel_circ_0001115	mmu-miR-497b	ENSMUST00000003659	ENSMUSG00000031849
novel_circ_0001115	mmu-miR-497b	ENSMUST00000212488	ENSMUSG00000031849
novel_circ_0001137	mmu-miR-497b	ENSMUST00000003659	ENSMUSG00000031849
novel_circ_0001137	mmu-miR-497b	ENSMUST00000212488	ENSMUSG00000031849
novel_circ_0001458	mmu-miR-497b	ENSMUST00000003659	ENSMUSG00000031849
novel_circ_0001458	mmu-miR-497b	ENSMUST00000212488	ENSMUSG00000031849
novel_circ_0001371	mmu-miR-497b	ENSMUST00000003659	ENSMUSG00000031849
novel_circ_0001371	mmu-miR-497b	ENSMUST00000212488	ENSMUSG00000031849
novel_circ_0001374	mmu-miR-497b	ENSMUST00000003659	ENSMUSG00000031849
novel_circ_0001374	mmu-miR-497b	ENSMUST00000212488	ENSMUSG00000031849
novel_circ_0001414	mmu-miR-497b	ENSMUST00000003659	ENSMUSG00000031849
novel_circ_0001414	mmu-miR-497b	ENSMUST00000212488	ENSMUSG00000031849
novel_circ_0001430	mmu-miR-497b	ENSMUST00000003659	ENSMUSG00000031849
novel_circ_0001430	mmu-miR-497b	ENSMUST00000212488	ENSMUSG00000031849
mmu_circ_0000097	mmu-miR-497b	ENSMUST00000003659	ENSMUSG00000031849
mmu_circ_0000097	mmu-miR-497b	ENSMUST00000212488	ENSMUSG00000031849
mmu_circ_0000114	mmu-miR-497b	ENSMUST00000003659	ENSMUSG00000031849
mmu_circ_0000114	mmu-miR-497b	ENSMUST00000212488	ENSMUSG00000031849
mmu_circ_0000063	mmu-miR-497b	ENSMUST00000003659	ENSMUSG00000031849
mmu_circ_0000063	mmu-miR-497b	ENSMUST00000212488	ENSMUSG00000031849
mmu_circ_0001162	mmu-miR-497b	ENSMUST00000003659	ENSMUSG00000031849
mmu_circ_0001162	mmu-miR-497b	ENSMUST00000212488	ENSMUSG00000031849

mmu_circ_0001167	mmu-miR-497b	ENSMUST00000003659	ENSMUSG00000031849
mmu_circ_0001167	mmu-miR-497b	ENSMUST00000212488	ENSMUSG00000031849
mmu_circ_0001123	mmu-miR-497b	ENSMUST00000003659	ENSMUSG00000031849
mmu_circ_0001123	mmu-miR-497b	ENSMUST00000212488	ENSMUSG00000031849
mmu_circ_0001135	mmu-miR-497b	ENSMUST00000003659	ENSMUSG00000031849
mmu_circ_0001135	mmu-miR-497b	ENSMUST00000212488	ENSMUSG00000031849
mmu_circ_0001135	mmu-miR-497b	ENSMUST00000003659	ENSMUSG00000031849
mmu_circ_0001135	mmu-miR-497b	ENSMUST00000212488	ENSMUSG00000031849
mmu_circ_0001148	mmu-miR-497b	ENSMUST00000003659	ENSMUSG00000031849
mmu_circ_0001148	mmu-miR-497b	ENSMUST00000212488	ENSMUSG00000031849
mmu_circ_0001103	mmu-miR-497b	ENSMUST00000003659	ENSMUSG00000031849
mmu_circ_0001103	mmu-miR-497b	ENSMUST00000212488	ENSMUSG00000031849
mmu_circ_0001103	mmu-miR-497b	ENSMUST00000003659	ENSMUSG00000031849
mmu_circ_0001103	mmu-miR-497b	ENSMUST00000212488	ENSMUSG00000031849
mmu_circ_0001369	mmu-miR-497b	ENSMUST00000003659	ENSMUSG00000031849
mmu_circ_0001369	mmu-miR-497b	ENSMUST00000212488	ENSMUSG00000031849
mmu_circ_0001369	mmu-miR-497b	ENSMUST00000003659	ENSMUSG00000031849
mmu_circ_0001369	mmu-miR-497b	ENSMUST00000212488	ENSMUSG00000031849
mmu_circ_0001376	mmu-miR-497b	ENSMUST00000003659	ENSMUSG00000031849
mmu_circ_0001376	mmu-miR-497b	ENSMUST00000212488	ENSMUSG00000031849
mmu_circ_0001421	mmu-miR-497b	ENSMUST00000003659	ENSMUSG00000031849
mmu_circ_0001421	mmu-miR-497b	ENSMUST00000212488	ENSMUSG00000031849
mmu_circ_0001331	mmu-miR-497b	ENSMUST00000003659	ENSMUSG00000031849
mmu_circ_0001331	mmu-miR-497b	ENSMUST00000212488	ENSMUSG00000031849
mmu_circ_0001333	mmu-miR-497b	ENSMUST00000003659	ENSMUSG00000031849
mmu_circ_0001333	mmu-miR-497b	ENSMUST00000212488	ENSMUSG00000031849
mmu_circ_0001291	mmu-miR-497b	ENSMUST00000003659	ENSMUSG00000031849
mmu_circ_0001291	mmu-miR-497b	ENSMUST00000212488	ENSMUSG00000031849
mmu_circ_0001507	mmu-miR-497b	ENSMUST00000003659	ENSMUSG00000031849
mmu_circ_0001507	mmu-miR-497b	ENSMUST00000212488	ENSMUSG00000031849
mmu_circ_0001528	mmu-miR-497b	ENSMUST00000003659	ENSMUSG00000031849
mmu_circ_0001528	mmu-miR-497b	ENSMUST00000212488	ENSMUSG00000031849
mmu_circ_0001454	mmu-miR-497b	ENSMUST00000003659	ENSMUSG00000031849
mmu_circ_0001454	mmu-miR-497b	ENSMUST00000212488	ENSMUSG00000031849
mmu_circ_0001499	mmu-miR-497b	ENSMUST00000003659	ENSMUSG00000031849
mmu_circ_0001499	mmu-miR-497b	ENSMUST00000212488	ENSMUSG00000031849
mmu_circ_0001500	mmu-miR-497b	ENSMUST00000003659	ENSMUSG00000031849
mmu_circ_0001500	mmu-miR-497b	ENSMUST00000212488	ENSMUSG00000031849
mmu_circ_0001774	mmu-miR-497b	ENSMUST00000003659	ENSMUSG00000031849
mmu_circ_0001774	mmu-miR-497b	ENSMUST00000212488	ENSMUSG00000031849
mmu_circ_0001795	mmu-miR-497b	ENSMUST00000003659	ENSMUSG00000031849
mmu_circ_0001795	mmu-miR-497b	ENSMUST00000212488	ENSMUSG00000031849
mmu_circ_0001818	mmu-miR-497b	ENSMUST00000003659	ENSMUSG00000031849
mmu_circ_0001818	mmu-miR-497b	ENSMUST00000212488	ENSMUSG00000031849
mmu_circ_0001743	mmu-miR-497b	ENSMUST00000003659	ENSMUSG00000031849
mmu_circ_0001743	mmu-miR-497b	ENSMUST00000212488	ENSMUSG00000031849
mmu_circ_0001665	mmu-miR-497b	ENSMUST00000003659	ENSMUSG00000031849
mmu_circ_0001665	mmu-miR-497b	ENSMUST00000212488	ENSMUSG00000031849
mmu_circ_0000239	mmu-miR-497b	ENSMUST00000003659	ENSMUSG00000031849
mmu_circ_0000239	mmu-miR-497b	ENSMUST00000212488	ENSMUSG00000031849
mmu_circ_0000499	mmu-miR-497b	ENSMUST00000003659	ENSMUSG00000031849
mmu_circ_0000499	mmu-miR-497b	ENSMUST00000212488	ENSMUSG00000031849
mmu_circ_0000500	mmu-miR-497b	ENSMUST00000003659	ENSMUSG00000031849
mmu_circ_0000500	mmu-miR-497b	ENSMUST00000212488	ENSMUSG00000031849
mmu_circ_0000477	mmu-miR-497b	ENSMUST00000003659	ENSMUSG00000031849
mmu_circ_0000477	mmu-miR-497b	ENSMUST00000212488	ENSMUSG00000031849
mmu_circ_0000399	mmu-miR-497b	ENSMUST00000003659	ENSMUSG00000031849
mmu_circ_0000399	mmu-miR-497b	ENSMUST00000212488	ENSMUSG00000031849

mmu_circ_0000588	mmu-miR-497b	ENSMUST00000003659	ENSMUSG00000031849
mmu_circ_0000588	mmu-miR-497b	ENSMUST00000212488	ENSMUSG00000031849
mmu_circ_0000588	mmu-miR-497b	ENSMUST00000003659	ENSMUSG00000031849
mmu_circ_0000588	mmu-miR-497b	ENSMUST00000212488	ENSMUSG00000031849
mmu_circ_0000628	mmu-miR-497b	ENSMUST00000003659	ENSMUSG00000031849
mmu_circ_0000628	mmu-miR-497b	ENSMUST00000212488	ENSMUSG00000031849
mmu_circ_0000635	mmu-miR-497b	ENSMUST00000003659	ENSMUSG00000031849
mmu_circ_0000635	mmu-miR-497b	ENSMUST00000212488	ENSMUSG00000031849
mmu_circ_0000644	mmu-miR-497b	ENSMUST00000003659	ENSMUSG00000031849
mmu_circ_0000644	mmu-miR-497b	ENSMUST00000212488	ENSMUSG00000031849
mmu_circ_0000507	mmu-miR-497b	ENSMUST00000003659	ENSMUSG00000031849
mmu_circ_0000507	mmu-miR-497b	ENSMUST00000212488	ENSMUSG00000031849
mmu_circ_0000530	mmu-miR-497b	ENSMUST00000003659	ENSMUSG00000031849
mmu_circ_0000530	mmu-miR-497b	ENSMUST00000212488	ENSMUSG00000031849
mmu_circ_0000795	mmu-miR-497b	ENSMUST00000003659	ENSMUSG00000031849
mmu_circ_0000795	mmu-miR-497b	ENSMUST00000212488	ENSMUSG00000031849
mmu_circ_0000804	mmu-miR-497b	ENSMUST00000003659	ENSMUSG00000031849
mmu_circ_0000804	mmu-miR-497b	ENSMUST00000212488	ENSMUSG00000031849
mmu_circ_0000812	mmu-miR-497b	ENSMUST00000003659	ENSMUSG00000031849
mmu_circ_0000812	mmu-miR-497b	ENSMUST00000212488	ENSMUSG00000031849
mmu_circ_0000828	mmu-miR-497b	ENSMUST00000003659	ENSMUSG00000031849
mmu_circ_0000828	mmu-miR-497b	ENSMUST00000212488	ENSMUSG00000031849
mmu_circ_0000829	mmu-miR-497b	ENSMUST00000003659	ENSMUSG00000031849
mmu_circ_0000829	mmu-miR-497b	ENSMUST00000212488	ENSMUSG00000031849
mmu_circ_0000693	mmu-miR-497b	ENSMUST00000003659	ENSMUSG00000031849
mmu_circ_0000693	mmu-miR-497b	ENSMUST00000212488	ENSMUSG00000031849
mmu_circ_0000711	mmu-miR-497b	ENSMUST00000003659	ENSMUSG00000031849
mmu_circ_0000711	mmu-miR-497b	ENSMUST00000212488	ENSMUSG00000031849
mmu_circ_0000713	mmu-miR-497b	ENSMUST00000003659	ENSMUSG00000031849
mmu_circ_0000713	mmu-miR-497b	ENSMUST00000212488	ENSMUSG00000031849
mmu_circ_0000714	mmu-miR-497b	ENSMUST00000003659	ENSMUSG00000031849
mmu_circ_0000714	mmu-miR-497b	ENSMUST00000212488	ENSMUSG00000031849
mmu_circ_0000944	mmu-miR-497b	ENSMUST00000003659	ENSMUSG00000031849
mmu_circ_0000944	mmu-miR-497b	ENSMUST00000212488	ENSMUSG00000031849
mmu_circ_0000950	mmu-miR-497b	ENSMUST00000003659	ENSMUSG00000031849
mmu_circ_0000950	mmu-miR-497b	ENSMUST00000212488	ENSMUSG00000031849
mmu_circ_0000962	mmu-miR-497b	ENSMUST00000003659	ENSMUSG00000031849
mmu_circ_0000962	mmu-miR-497b	ENSMUST00000212488	ENSMUSG00000031849
mmu_circ_0000969	mmu-miR-497b	ENSMUST00000003659	ENSMUSG00000031849
mmu_circ_0000969	mmu-miR-497b	ENSMUST00000212488	ENSMUSG00000031849
mmu_circ_0000857	mmu-miR-497b	ENSMUST00000003659	ENSMUSG00000031849
mmu_circ_0000857	mmu-miR-497b	ENSMUST00000212488	ENSMUSG00000031849
mmu_circ_0000862	mmu-miR-497b	ENSMUST00000003659	ENSMUSG00000031849
mmu_circ_0000862	mmu-miR-497b	ENSMUST00000212488	ENSMUSG00000031849
mmu_circ_0000876	mmu-miR-497b	ENSMUST00000003659	ENSMUSG00000031849
mmu_circ_0000876	mmu-miR-497b	ENSMUST00000212488	ENSMUSG00000031849
mmu_circ_0000884	mmu-miR-497b	ENSMUST00000003659	ENSMUSG00000031849
mmu_circ_0000884	mmu-miR-497b	ENSMUST00000212488	ENSMUSG00000031849
mmu_circ_0000886	mmu-miR-497b	ENSMUST00000003659	ENSMUSG00000031849
mmu_circ_0000886	mmu-miR-497b	ENSMUST00000212488	ENSMUSG00000031849
mmu_circ_0000906	mmu-miR-497b	ENSMUST00000003659	ENSMUSG00000031849
mmu_circ_0000906	mmu-miR-497b	ENSMUST00000212488	ENSMUSG00000031849
mmu_circ_0000907	mmu-miR-497b	ENSMUST00000003659	ENSMUSG00000031849
mmu_circ_0000907	mmu-miR-497b	ENSMUST00000212488	ENSMUSG00000031849
mmu_circ_0000908	mmu-miR-497b	ENSMUST00000003659	ENSMUSG00000031849
mmu_circ_0000908	mmu-miR-497b	ENSMUST00000212488	ENSMUSG00000031849
mmu_circ_0000912	mmu-miR-497b	ENSMUST00000003659	ENSMUSG00000031849
mmu_circ_0000912	mmu-miR-497b	ENSMUST00000212488	ENSMUSG00000031849

novel_circ_0001552	mmu-miR-497b	ENSMUST00000003659	ENSMUSG00000031849
novel_circ_0001552	mmu-miR-497b	ENSMUST00000212488	ENSMUSG00000031849
novel_circ_0001552	mmu-miR-497b	ENSMUST00000003659	ENSMUSG00000031849
novel_circ_0001552	mmu-miR-497b	ENSMUST00000212488	ENSMUSG00000031849
novel_circ_0001553	mmu-miR-497b	ENSMUST00000003659	ENSMUSG00000031849
novel_circ_0001553	mmu-miR-497b	ENSMUST00000212488	ENSMUSG00000031849
novel_circ_0001553	mmu-miR-497b	ENSMUST00000003659	ENSMUSG00000031849
novel_circ_0001553	mmu-miR-497b	ENSMUST00000212488	ENSMUSG00000031849
novel_circ_0001570	mmu-miR-497b	ENSMUST00000003659	ENSMUSG00000031849
novel_circ_0001570	mmu-miR-497b	ENSMUST00000212488	ENSMUSG00000031849
novel_circ_0001608	mmu-miR-497b	ENSMUST00000003659	ENSMUSG00000031849
novel_circ_0001608	mmu-miR-497b	ENSMUST00000212488	ENSMUSG00000031849
novel_circ_0001676	mmu-miR-497b	ENSMUST00000003659	ENSMUSG00000031849
novel_circ_0001676	mmu-miR-497b	ENSMUST00000212488	ENSMUSG00000031849
novel_circ_0002115	mmu-miR-497b	ENSMUST00000003659	ENSMUSG00000031849
novel_circ_0002115	mmu-miR-497b	ENSMUST00000212488	ENSMUSG00000031849
novel_circ_0002123	mmu-miR-497b	ENSMUST00000003659	ENSMUSG00000031849
novel_circ_0002123	mmu-miR-497b	ENSMUST00000212488	ENSMUSG00000031849
novel_circ_0002142	mmu-miR-497b	ENSMUST00000003659	ENSMUSG00000031849
novel_circ_0002142	mmu-miR-497b	ENSMUST00000212488	ENSMUSG00000031849
novel_circ_0002192	mmu-miR-497b	ENSMUST00000003659	ENSMUSG00000031849
novel_circ_0002192	mmu-miR-497b	ENSMUST00000212488	ENSMUSG00000031849
novel_circ_0002198	mmu-miR-497b	ENSMUST00000003659	ENSMUSG00000031849
novel_circ_0002198	mmu-miR-497b	ENSMUST00000212488	ENSMUSG00000031849
novel_circ_0001761	mmu-miR-497b	ENSMUST00000003659	ENSMUSG00000031849
novel_circ_0001761	mmu-miR-497b	ENSMUST00000212488	ENSMUSG00000031849
novel_circ_0001800	mmu-miR-497b	ENSMUST00000003659	ENSMUSG00000031849
novel_circ_0001800	mmu-miR-497b	ENSMUST00000212488	ENSMUSG00000031849
novel_circ_0001859	mmu-miR-497b	ENSMUST00000003659	ENSMUSG00000031849
novel_circ_0001859	mmu-miR-497b	ENSMUST00000212488	ENSMUSG00000031849
novel_circ_0001905	mmu-miR-497b	ENSMUST00000003659	ENSMUSG00000031849
novel_circ_0001905	mmu-miR-497b	ENSMUST00000212488	ENSMUSG00000031849
novel_circ_0001905	mmu-miR-497b	ENSMUST00000003659	ENSMUSG00000031849
novel_circ_0001905	mmu-miR-497b	ENSMUST00000212488	ENSMUSG00000031849
novel_circ_0001912	mmu-miR-497b	ENSMUST00000003659	ENSMUSG00000031849
novel_circ_0001912	mmu-miR-497b	ENSMUST00000212488	ENSMUSG00000031849
novel_circ_0001912	mmu-miR-497b	ENSMUST00000003659	ENSMUSG00000031849
novel_circ_0001912	mmu-miR-497b	ENSMUST00000212488	ENSMUSG00000031849
novel_circ_0002468	mmu-miR-497b	ENSMUST00000003659	ENSMUSG00000031849
novel_circ_0002468	mmu-miR-497b	ENSMUST00000212488	ENSMUSG00000031849
novel_circ_0002479	mmu-miR-497b	ENSMUST00000003659	ENSMUSG00000031849
novel_circ_0002479	mmu-miR-497b	ENSMUST00000212488	ENSMUSG00000031849
novel_circ_0002489	mmu-miR-497b	ENSMUST00000003659	ENSMUSG00000031849
novel_circ_0002489	mmu-miR-497b	ENSMUST00000212488	ENSMUSG00000031849
novel_circ_0002499	mmu-miR-497b	ENSMUST00000003659	ENSMUSG00000031849
novel_circ_0002499	mmu-miR-497b	ENSMUST00000212488	ENSMUSG00000031849
novel_circ_0002500	mmu-miR-497b	ENSMUST00000003659	ENSMUSG00000031849
novel_circ_0002500	mmu-miR-497b	ENSMUST00000212488	ENSMUSG00000031849
novel_circ_0002501	mmu-miR-497b	ENSMUST00000003659	ENSMUSG00000031849
novel_circ_0002501	mmu-miR-497b	ENSMUST00000212488	ENSMUSG00000031849
novel_circ_0002555	mmu-miR-497b	ENSMUST00000003659	ENSMUSG00000031849
novel_circ_0002555	mmu-miR-497b	ENSMUST00000212488	ENSMUSG00000031849
novel_circ_0002617	mmu-miR-497b	ENSMUST00000003659	ENSMUSG00000031849
novel_circ_0002617	mmu-miR-497b	ENSMUST00000212488	ENSMUSG00000031849
novel_circ_0002318	mmu-miR-497b	ENSMUST00000003659	ENSMUSG00000031849
novel_circ_0002318	mmu-miR-497b	ENSMUST00000212488	ENSMUSG00000031849
novel_circ_0002319	mmu-miR-497b	ENSMUST00000003659	ENSMUSG00000031849
novel_circ_0002319	mmu-miR-497b	ENSMUST00000212488	ENSMUSG00000031849

novel_circ_0002348	mmu-miR-497b	ENSMUST00000003659	ENSMUSG00000031849
novel_circ_0002348	mmu-miR-497b	ENSMUST00000212488	ENSMUSG00000031849
novel_circ_0002398	mmu-miR-497b	ENSMUST00000003659	ENSMUSG00000031849
novel_circ_0002398	mmu-miR-497b	ENSMUST00000212488	ENSMUSG00000031849
novel_circ_0002917	mmu-miR-497b	ENSMUST00000003659	ENSMUSG00000031849
novel_circ_0002917	mmu-miR-497b	ENSMUST00000212488	ENSMUSG00000031849
novel_circ_0002928	mmu-miR-497b	ENSMUST00000003659	ENSMUSG00000031849
novel_circ_0002928	mmu-miR-497b	ENSMUST00000212488	ENSMUSG00000031849
novel_circ_0002944	mmu-miR-497b	ENSMUST00000003659	ENSMUSG00000031849
novel_circ_0002944	mmu-miR-497b	ENSMUST00000212488	ENSMUSG00000031849
novel_circ_0002946	mmu-miR-497b	ENSMUST00000003659	ENSMUSG00000031849
novel_circ_0002946	mmu-miR-497b	ENSMUST00000212488	ENSMUSG00000031849
novel_circ_0002946	mmu-miR-497b	ENSMUST00000003659	ENSMUSG00000031849
novel_circ_0002946	mmu-miR-497b	ENSMUST00000212488	ENSMUSG00000031849
novel_circ_0002970	mmu-miR-497b	ENSMUST00000003659	ENSMUSG00000031849
novel_circ_0002970	mmu-miR-497b	ENSMUST00000212488	ENSMUSG00000031849
novel_circ_0002982	mmu-miR-497b	ENSMUST00000003659	ENSMUSG00000031849
novel_circ_0002982	mmu-miR-497b	ENSMUST00000212488	ENSMUSG00000031849
novel_circ_0002991	mmu-miR-497b	ENSMUST00000003659	ENSMUSG00000031849
novel_circ_0002991	mmu-miR-497b	ENSMUST00000212488	ENSMUSG00000031849
novel_circ_0002680	mmu-miR-497b	ENSMUST00000003659	ENSMUSG00000031849
novel_circ_0002680	mmu-miR-497b	ENSMUST00000212488	ENSMUSG00000031849
novel_circ_0002788	mmu-miR-497b	ENSMUST00000003659	ENSMUSG00000031849
novel_circ_0002788	mmu-miR-497b	ENSMUST00000212488	ENSMUSG00000031849
novel_circ_0003337	mmu-miR-497b	ENSMUST00000003659	ENSMUSG00000031849
novel_circ_0003337	mmu-miR-497b	ENSMUST00000212488	ENSMUSG00000031849
novel_circ_0003350	mmu-miR-497b	ENSMUST00000003659	ENSMUSG00000031849
novel_circ_0003350	mmu-miR-497b	ENSMUST00000212488	ENSMUSG00000031849
novel_circ_0003377	mmu-miR-497b	ENSMUST00000003659	ENSMUSG00000031849
novel_circ_0003377	mmu-miR-497b	ENSMUST00000212488	ENSMUSG00000031849
novel_circ_0003382	mmu-miR-497b	ENSMUST00000003659	ENSMUSG00000031849
novel_circ_0003382	mmu-miR-497b	ENSMUST00000212488	ENSMUSG00000031849
novel_circ_0003065	mmu-miR-497b	ENSMUST00000003659	ENSMUSG00000031849
novel_circ_0003065	mmu-miR-497b	ENSMUST00000212488	ENSMUSG00000031849
novel_circ_0003067	mmu-miR-497b	ENSMUST00000003659	ENSMUSG00000031849
novel_circ_0003067	mmu-miR-497b	ENSMUST00000212488	ENSMUSG00000031849
novel_circ_0003076	mmu-miR-497b	ENSMUST00000003659	ENSMUSG00000031849
novel_circ_0003076	mmu-miR-497b	ENSMUST00000212488	ENSMUSG00000031849
novel_circ_0003491	mmu-miR-497b	ENSMUST00000003659	ENSMUSG00000031849
novel_circ_0003491	mmu-miR-497b	ENSMUST00000212488	ENSMUSG00000031849
novel_circ_0003491	mmu-miR-497b	ENSMUST00000003659	ENSMUSG00000031849
novel_circ_0003491	mmu-miR-497b	ENSMUST00000212488	ENSMUSG00000031849
novel_circ_0000164	mmu-miR-497b	ENSMUST00000003659	ENSMUSG00000031849
novel_circ_0000164	mmu-miR-497b	ENSMUST00000212488	ENSMUSG00000031849
novel_circ_0000192	mmu-miR-497b	ENSMUST00000003659	ENSMUSG00000031849
novel_circ_0000192	mmu-miR-497b	ENSMUST00000212488	ENSMUSG00000031849
novel_circ_0000240	mmu-miR-497b	ENSMUST00000003659	ENSMUSG00000031849
novel_circ_0000240	mmu-miR-497b	ENSMUST00000212488	ENSMUSG00000031849
novel_circ_0000264	mmu-miR-497b	ENSMUST00000003659	ENSMUSG00000031849
novel_circ_0000264	mmu-miR-497b	ENSMUST00000212488	ENSMUSG00000031849
novel_circ_0000280	mmu-miR-497b	ENSMUST00000003659	ENSMUSG00000031849
novel_circ_0000280	mmu-miR-497b	ENSMUST00000212488	ENSMUSG00000031849
novel_circ_0000120	mmu-miR-497b	ENSMUST00000003659	ENSMUSG00000031849
novel_circ_0000120	mmu-miR-497b	ENSMUST00000212488	ENSMUSG00000031849
novel_circ_0000691	mmu-miR-497b	ENSMUST00000003659	ENSMUSG00000031849
novel_circ_0000691	mmu-miR-497b	ENSMUST00000212488	ENSMUSG00000031849
novel_circ_0000698	mmu-miR-497b	ENSMUST00000003659	ENSMUSG00000031849
novel_circ_0000698	mmu-miR-497b	ENSMUST00000212488	ENSMUSG00000031849

novel_circ_0000731	mmu-miR-497b	ENSMUST00000003659	ENSMUSG00000031849
novel_circ_0000731	mmu-miR-497b	ENSMUST00000212488	ENSMUSG00000031849
novel_circ_0000733	mmu-miR-497b	ENSMUST00000003659	ENSMUSG00000031849
novel_circ_0000733	mmu-miR-497b	ENSMUST00000212488	ENSMUSG00000031849
novel_circ_0000775	mmu-miR-497b	ENSMUST00000003659	ENSMUSG00000031849
novel_circ_0000775	mmu-miR-497b	ENSMUST00000212488	ENSMUSG00000031849
novel_circ_0000501	mmu-miR-497b	ENSMUST00000003659	ENSMUSG00000031849
novel_circ_0000501	mmu-miR-497b	ENSMUST00000212488	ENSMUSG00000031849
novel_circ_0000505	mmu-miR-497b	ENSMUST00000003659	ENSMUSG00000031849
novel_circ_0000505	mmu-miR-497b	ENSMUST00000212488	ENSMUSG00000031849
novel_circ_0000595	mmu-miR-497b	ENSMUST00000003659	ENSMUSG00000031849
novel_circ_0000595	mmu-miR-497b	ENSMUST00000212488	ENSMUSG00000031849
novel_circ_0000942	mmu-miR-497b	ENSMUST00000003659	ENSMUSG00000031849
novel_circ_0000942	mmu-miR-497b	ENSMUST00000212488	ENSMUSG00000031849
novel_circ_0000942	mmu-miR-497b	ENSMUST00000003659	ENSMUSG00000031849
novel_circ_0000942	mmu-miR-497b	ENSMUST00000212488	ENSMUSG00000031849
novel_circ_0000954	mmu-miR-497b	ENSMUST00000003659	ENSMUSG00000031849
novel_circ_0000954	mmu-miR-497b	ENSMUST00000212488	ENSMUSG00000031849
novel_circ_0000954	mmu-miR-497b	ENSMUST00000003659	ENSMUSG00000031849
novel_circ_0000954	mmu-miR-497b	ENSMUST00000212488	ENSMUSG00000031849
novel_circ_0001001	mmu-miR-497b	ENSMUST00000003659	ENSMUSG00000031849
novel_circ_0001001	mmu-miR-497b	ENSMUST00000212488	ENSMUSG00000031849
novel_circ_0001015	mmu-miR-497b	ENSMUST00000003659	ENSMUSG00000031849
novel_circ_0001015	mmu-miR-497b	ENSMUST00000212488	ENSMUSG00000031849
novel_circ_0000835	mmu-miR-497b	ENSMUST00000003659	ENSMUSG00000031849
novel_circ_0000835	mmu-miR-497b	ENSMUST00000212488	ENSMUSG00000031849
novel_circ_0001194	mmu-miR-497b	ENSMUST00000003659	ENSMUSG00000031849
novel_circ_0001194	mmu-miR-497b	ENSMUST00000212488	ENSMUSG00000031849
novel_circ_0001207	mmu-miR-497b	ENSMUST00000003659	ENSMUSG00000031849
novel_circ_0001207	mmu-miR-497b	ENSMUST00000212488	ENSMUSG00000031849
novel_circ_0001208	mmu-miR-497b	ENSMUST00000003659	ENSMUSG00000031849
novel_circ_0001208	mmu-miR-497b	ENSMUST00000212488	ENSMUSG00000031849
novel_circ_0001270	mmu-miR-1970c-	ENSMUST00000225594	ENSMUSG00000017491
novel_circ_0001112	mmu-miR-1970c-	ENSMUST00000225594	ENSMUSG00000017491
novel_circ_0001315	mmu-miR-1970c-	ENSMUST00000225594	ENSMUSG00000017491
novel_circ_0001355	mmu-miR-1970c-	ENSMUST00000225594	ENSMUSG00000017491
mmu_circ_0000051	mmu-miR-1970c-	ENSMUST00000225594	ENSMUSG00000017491
mmu_circ_0000051	mmu-miR-1970c-	ENSMUST00000225594	ENSMUSG00000017491
mmu_circ_0001123	mmu-miR-1970c-	ENSMUST00000225594	ENSMUSG00000017491
mmu_circ_0001124	mmu-miR-1970c-	ENSMUST00000225594	ENSMUSG00000017491
mmu_circ_0001125	mmu-miR-1970c-	ENSMUST00000225594	ENSMUSG00000017491
mmu_circ_0001059	mmu-miR-1970c-	ENSMUST00000225594	ENSMUSG00000017491
mmu_circ_0001021	mmu-miR-1970c-	ENSMUST00000225594	ENSMUSG00000017491
mmu_circ_0001333	mmu-miR-1970c-	ENSMUST00000225594	ENSMUSG00000017491
mmu_circ_0001213	mmu-miR-1970c-	ENSMUST00000225594	ENSMUSG00000017491
mmu_circ_0001537	mmu-miR-1970c-	ENSMUST00000225594	ENSMUSG00000017491
mmu_circ_0001454	mmu-miR-1970c-	ENSMUST00000225594	ENSMUSG00000017491
mmu_circ_0001436	mmu-miR-1970c-	ENSMUST00000225594	ENSMUSG00000017491
mmu_circ_0001436	mmu-miR-1970c-	ENSMUST00000225594	ENSMUSG00000017491
mmu_circ_0001499	mmu-miR-1970c-	ENSMUST00000225594	ENSMUSG00000017491
mmu_circ_0001500	mmu-miR-1970c-	ENSMUST00000225594	ENSMUSG00000017491
mmu_circ_0000465	mmu-miR-1970c-	ENSMUST00000225594	ENSMUSG00000017491
mmu_circ_0000491	mmu-miR-1970c-	ENSMUST00000225594	ENSMUSG00000017491
mmu_circ_0000404	mmu-miR-1970c-	ENSMUST00000225594	ENSMUSG00000017491
mmu_circ_0000404	mmu-miR-1970c-	ENSMUST00000225594	ENSMUSG00000017491
mmu_circ_0000530	mmu-miR-1970c-	ENSMUST00000225594	ENSMUSG00000017491
mmu_circ_0000738	mmu-miR-1970c-	ENSMUST00000225594	ENSMUSG00000017491
mmu_circ_0000804	mmu-miR-1970c-	ENSMUST00000225594	ENSMUSG00000017491

mmu_circ_0000829	mmu-miR-1970c-	ENSMUST00000225594	ENSMUSG00000017491
mmu_circ_0000831	mmu-miR-1970c-	ENSMUST00000225594	ENSMUSG00000017491
mmu_circ_0000688	mmu-miR-1970c-	ENSMUST00000225594	ENSMUSG00000017491
mmu_circ_0000711	mmu-miR-1970c-	ENSMUST00000225594	ENSMUSG00000017491
mmu_circ_0000714	mmu-miR-1970c-	ENSMUST00000225594	ENSMUSG00000017491
mmu_circ_0000969	mmu-miR-1970c-	ENSMUST00000225594	ENSMUSG00000017491
mmu_circ_0000844	mmu-miR-1970c-	ENSMUST00000225594	ENSMUSG00000017491
mmu_circ_0000844	mmu-miR-1970c-	ENSMUST00000225594	ENSMUSG00000017491
mmu_circ_0000884	mmu-miR-1970c-	ENSMUST00000225594	ENSMUSG00000017491
mmu_circ_0000886	mmu-miR-1970c-	ENSMUST00000225594	ENSMUSG00000017491
mmu_circ_0000906	mmu-miR-1970c-	ENSMUST00000225594	ENSMUSG00000017491
mmu_circ_0000907	mmu-miR-1970c-	ENSMUST00000225594	ENSMUSG00000017491
mmu_circ_0000908	mmu-miR-1970c-	ENSMUST00000225594	ENSMUSG00000017491
mmu_circ_0000912	mmu-miR-1970c-	ENSMUST00000225594	ENSMUSG00000017491
novel_circ_0001597	mmu-miR-1970c-	ENSMUST00000225594	ENSMUSG00000017491
novel_circ_0001597	mmu-miR-1970c-	ENSMUST00000225594	ENSMUSG00000017491
novel_circ_0002143	mmu-miR-1970c-	ENSMUST00000225594	ENSMUSG00000017491
novel_circ_0001769	mmu-miR-1970c-	ENSMUST00000225594	ENSMUSG00000017491
novel_circ_0001770	mmu-miR-1970c-	ENSMUST00000225594	ENSMUSG00000017491
novel_circ_0001782	mmu-miR-1970c-	ENSMUST00000225594	ENSMUSG00000017491
novel_circ_0001883	mmu-miR-1970c-	ENSMUST00000225594	ENSMUSG00000017491
novel_circ_0002600	mmu-miR-1970c-	ENSMUST00000225594	ENSMUSG00000017491
novel_circ_0002617	mmu-miR-1970c-	ENSMUST00000225594	ENSMUSG00000017491
novel_circ_0002274	mmu-miR-1970c-	ENSMUST00000225594	ENSMUSG00000017491
novel_circ_0002356	mmu-miR-1970c-	ENSMUST00000225594	ENSMUSG00000017491
novel_circ_0002375	mmu-miR-1970c-	ENSMUST00000225594	ENSMUSG00000017491
novel_circ_0002831	mmu-miR-1970c-	ENSMUST00000225594	ENSMUSG00000017491
novel_circ_0002913	mmu-miR-1970c-	ENSMUST00000225594	ENSMUSG00000017491
novel_circ_0002913	mmu-miR-1970c-	ENSMUST00000225594	ENSMUSG00000017491
novel_circ_0002656	mmu-miR-1970c-	ENSMUST00000225594	ENSMUSG00000017491
novel_circ_0002658	mmu-miR-1970c-	ENSMUST00000225594	ENSMUSG00000017491
novel_circ_0002680	mmu-miR-1970c-	ENSMUST00000225594	ENSMUSG00000017491
novel_circ_0002681	mmu-miR-1970c-	ENSMUST00000225594	ENSMUSG00000017491
novel_circ_0002720	mmu-miR-1970c-	ENSMUST00000225594	ENSMUSG00000017491
novel_circ_0002720	mmu-miR-1970c-	ENSMUST00000225594	ENSMUSG00000017491
novel_circ_0002724	mmu-miR-1970c-	ENSMUST00000225594	ENSMUSG00000017491
novel_circ_0002724	mmu-miR-1970c-	ENSMUST00000225594	ENSMUSG00000017491
novel_circ_0003242	mmu-miR-1970c-	ENSMUST00000225594	ENSMUSG00000017491
novel_circ_0003267	mmu-miR-1970c-	ENSMUST00000225594	ENSMUSG00000017491
novel_circ_0003268	mmu-miR-1970c-	ENSMUST00000225594	ENSMUSG00000017491
novel_circ_0003016	mmu-miR-1970c-	ENSMUST00000225594	ENSMUSG00000017491
novel_circ_0003083	mmu-miR-1970c-	ENSMUST00000225594	ENSMUSG00000017491
novel_circ_0003142	mmu-miR-1970c-	ENSMUST00000225594	ENSMUSG00000017491
novel_circ_0003156	mmu-miR-1970c-	ENSMUST00000225594	ENSMUSG00000017491
novel_circ_0003458	mmu-miR-1970c-	ENSMUST00000225594	ENSMUSG00000017491
novel_circ_0000200	mmu-miR-1970c-	ENSMUST00000225594	ENSMUSG00000017491
novel_circ_0000201	mmu-miR-1970c-	ENSMUST00000225594	ENSMUSG00000017491
novel_circ_0000208	mmu-miR-1970c-	ENSMUST00000225594	ENSMUSG00000017491
novel_circ_0000244	mmu-miR-1970c-	ENSMUST00000225594	ENSMUSG00000017491
novel_circ_0000268	mmu-miR-1970c-	ENSMUST00000225594	ENSMUSG00000017491
novel_circ_0000389	mmu-miR-1970c-	ENSMUST00000225594	ENSMUSG00000017491
novel_circ_0000443	mmu-miR-1970c-	ENSMUST00000225594	ENSMUSG00000017491
novel_circ_0000015	mmu-miR-1970c-	ENSMUST00000225594	ENSMUSG00000017491
novel_circ_0000050	mmu-miR-1970c-	ENSMUST00000225594	ENSMUSG00000017491
novel_circ_0000682	mmu-miR-1970c-	ENSMUST00000225594	ENSMUSG00000017491
novel_circ_0000753	mmu-miR-1970c-	ENSMUST00000225594	ENSMUSG00000017491
novel_circ_0000514	mmu-miR-1970c-	ENSMUST00000225594	ENSMUSG00000017491
novel_circ_0000938	mmu-miR-1970c-	ENSMUST00000225594	ENSMUSG00000017491

novel_circ_0001019	mmu-miR-1970c-5	ENSMUST00000225594	ENSMUSG00000017491
novel_circ_0001019	mmu-miR-1970c-5	ENSMUST00000225594	ENSMUSG00000017491
novel_circ_0000904	mmu-miR-1970c-5	ENSMUST00000225594	ENSMUSG00000017491
mmu_circ_0001376	mmu-miR-99b-5p	ENSMUST00000003659	ENSMUSG00000031849
mmu_circ_0001376	mmu-miR-99b-5p	ENSMUST00000212488	ENSMUSG00000031849
mmu_circ_0001632	mmu-miR-99b-5p	ENSMUST00000003659	ENSMUSG00000031849
mmu_circ_0001632	mmu-miR-99b-5p	ENSMUST00000212488	ENSMUSG00000031849
mmu_circ_0000713	mmu-miR-99b-5p	ENSMUST00000003659	ENSMUSG00000031849
mmu_circ_0000713	mmu-miR-99b-5p	ENSMUST00000212488	ENSMUSG00000031849
mmu_circ_0000714	mmu-miR-99b-5p	ENSMUST00000003659	ENSMUSG00000031849
mmu_circ_0000714	mmu-miR-99b-5p	ENSMUST00000212488	ENSMUSG00000031849
novel_circ_0002622	mmu-miR-99b-5p	ENSMUST00000003659	ENSMUSG00000031849
novel_circ_0002622	mmu-miR-99b-5p	ENSMUST00000212488	ENSMUSG00000031849
novel_circ_0002301	mmu-miR-99b-5p	ENSMUST00000003659	ENSMUSG00000031849
novel_circ_0002301	mmu-miR-99b-5p	ENSMUST00000212488	ENSMUSG00000031849
novel_circ_0002887	mmu-miR-99b-5p	ENSMUST00000003659	ENSMUSG00000031849
novel_circ_0002887	mmu-miR-99b-5p	ENSMUST00000212488	ENSMUSG00000031849
novel_circ_0002720	mmu-miR-99b-5p	ENSMUST00000003659	ENSMUSG00000031849
novel_circ_0002720	mmu-miR-99b-5p	ENSMUST00000212488	ENSMUSG00000031849
novel_circ_0000181	mmu-miR-99b-5p	ENSMUST00000003659	ENSMUSG00000031849
novel_circ_0000181	mmu-miR-99b-5p	ENSMUST00000212488	ENSMUSG00000031849
novel_circ_0000182	mmu-miR-99b-5p	ENSMUST00000003659	ENSMUSG00000031849
novel_circ_0000182	mmu-miR-99b-5p	ENSMUST00000212488	ENSMUSG00000031849
novel_circ_0000199	mmu-miR-99b-5p	ENSMUST00000003659	ENSMUSG00000031849
novel_circ_0000199	mmu-miR-99b-5p	ENSMUST00000212488	ENSMUSG00000031849
novel_circ_0000280	mmu-miR-99b-5p	ENSMUST00000003659	ENSMUSG00000031849
novel_circ_0000280	mmu-miR-99b-5p	ENSMUST00000212488	ENSMUSG00000031849
novel_circ_0000108	mmu-miR-99b-5p	ENSMUST00000003659	ENSMUSG00000031849
novel_circ_0000108	mmu-miR-99b-5p	ENSMUST00000212488	ENSMUSG00000031849
novel_circ_0000140	mmu-miR-99b-5p	ENSMUST00000003659	ENSMUSG00000031849
novel_circ_0000140	mmu-miR-99b-5p	ENSMUST00000212488	ENSMUSG00000031849
novel_circ_0000140	mmu-miR-99b-5p	ENSMUST00000003659	ENSMUSG00000031849
novel_circ_0000140	mmu-miR-99b-5p	ENSMUST00000212488	ENSMUSG00000031849

Table S22 circRNA-miRNA-mRNA co-expression networks

miRNA id	circRNA source gene id	cor(miRNAvscir	pval	mRNA id	cor(miRNAvsmRNA)	pval
mmu-let-7g-3p	n/a	-0.887800682	0.018176809	ENSMUSG00000058248	-0.875483728	0.022291184
mmu-let-7g-3p	n/a	-0.887800682	0.018176809	ENSMUSG00000054150	-0.858204501	0.028733478
mmu-let-7g-3p	n/a	-0.887800682	0.018176809	ENSMUSG00000048012	-0.870542927	0.024053906
mmu-let-7g-3p	n/a	-0.887800682	0.018176809	ENSMUSG00000034724	-0.917093889	0.01002521
mmu-let-7g-3p	n/a	-0.887800682	0.018176809	ENSMUSG00000037007	-0.948460186	0.003916075
mmu-let-7g-3p	ENSMUSG00000006673;	-0.927918296	0.007606398	ENSMUSG00000058248	-0.875483728	0.022291184
mmu-let-7g-3p	ENSMUSG00000006673;	-0.927918296	0.007606398	ENSMUSG00000054150	-0.858204501	0.028733478
mmu-let-7g-3p	ENSMUSG00000006673;	-0.927918296	0.007606398	ENSMUSG00000048012	-0.870542927	0.024053906
mmu-let-7g-3p	ENSMUSG00000006673;	-0.927918296	0.007606398	ENSMUSG00000034724	-0.917093889	0.01002521
mmu-let-7g-3p	ENSMUSG00000006673;	-0.927918296	0.007606398	ENSMUSG00000037007	-0.948460186	0.003916075
mmu-let-7g-3p	ENSMUSG00000062949;	-0.90164495	0.014034844	ENSMUSG00000058248	-0.875483728	0.022291184
mmu-let-7g-3p	ENSMUSG00000062949;	-0.90164495	0.014034844	ENSMUSG00000054150	-0.858204501	0.028733478
mmu-let-7g-3p	ENSMUSG00000062949;	-0.90164495	0.014034844	ENSMUSG00000048012	-0.870542927	0.024053906
mmu-let-7g-3p	ENSMUSG00000062949;	-0.90164495	0.014034844	ENSMUSG00000034724	-0.917093889	0.01002521
mmu-let-7g-3p	ENSMUSG00000062949;	-0.90164495	0.014034844	ENSMUSG00000037007	-0.948460186	0.003916075
mmu-let-7g-3p	ENSMUSG00000079509;	-0.90164495	0.014034844	ENSMUSG00000058248	-0.875483728	0.022291184
mmu-let-7g-3p	ENSMUSG00000079509;	-0.90164495	0.014034844	ENSMUSG00000054150	-0.858204501	0.028733478
mmu-let-7g-3p	ENSMUSG00000079509;	-0.90164495	0.014034844	ENSMUSG00000048012	-0.870542927	0.024053906
mmu-let-7g-3p	ENSMUSG00000079509;	-0.90164495	0.014034844	ENSMUSG00000034724	-0.917093889	0.01002521
mmu-let-7g-3p	ENSMUSG00000079509;	-0.90164495	0.014034844	ENSMUSG00000037007	-0.948460186	0.003916075
mmu-miR-101a-3p	ENSMUSG00000028580;	-0.865077895	0.026077903	ENSMUSG00000079494	-0.896570625	0.015493229
mmu-miR-101a-3p	ENSMUSG00000028580;	-0.865077895	0.026077903	ENSMUSG00000033847	-0.872552102	0.023329384
mmu-miR-101b-3p	ENSMUSG00000024241;	-0.921398864	0.009024404	ENSMUSG00000079494	-0.852055214	0.031212407
mmu-miR-101b-3p	ENSMUSG00000024135;	-0.941951351	0.004956667	ENSMUSG00000079494	-0.852055214	0.031212407
mmu-miR-101b-3p	ENSMUSG00000040850;	-0.89833949	0.014976965	ENSMUSG00000079494	-0.852055214	0.031212407
mmu-miR-103-3p	ENSMUSG00000040785;	-0.884026554	0.019394848	ENSMUSG00000023452	-0.868770694	0.024701738
mmu-miR-106a-5p	ENSMUSG00000001150;	-0.888450762	0.017970832	ENSMUSG00000037904	-0.928570329	0.007471073
mmu-miR-106a-5p	ENSMUSG00000001150;	-0.888450762	0.017970832	ENSMUSG00000008348	-0.931779028	0.006822398
mmu-miR-106a-5p	ENSMUSG00000001150;	-0.888450762	0.017970832	ENSMUSG00000037904	-0.888450762	0.017970832
mmu-miR-106a-5p	ENSMUSG00000021557;	-0.888450762	0.017970832	ENSMUSG00000037904	-0.928570329	0.007471073
mmu-miR-106a-5p	ENSMUSG00000021557;	-0.888450762	0.017970832	ENSMUSG00000008348	-0.931779028	0.006822398
mmu-miR-106a-5p	ENSMUSG00000021557;	-0.888450762	0.017970832	ENSMUSG00000037904	-0.888450762	0.017970832
mmu-miR-106a-5p	ENSMUSG00000052656;	-0.888450762	0.017970832	ENSMUSG00000037904	-0.928570329	0.007471073
mmu-miR-106a-5p	ENSMUSG00000052656;	-0.888450762	0.017970832	ENSMUSG00000008348	-0.931779028	0.006822398
mmu-miR-106a-5p	ENSMUSG00000052656;	-0.888450762	0.017970832	ENSMUSG00000037904	-0.888450762	0.017970832
mmu-miR-106a-5p	ENSMUSG00000038563;	-0.888450762	0.017970832	ENSMUSG00000037904	-0.928570329	0.007471073
mmu-miR-106a-5p	ENSMUSG00000038563;	-0.888450762	0.017970832	ENSMUSG00000008348	-0.931779028	0.006822398

mmu-miR-106a-5p	ENSMUSG00000038563;	-0.888450762	0.017970832	ENSMUSG00000037904	-0.888450762	0.017970832
mmu-miR-106a-5p	ENSMUSG00000036550;	-0.888450762	0.017970832	ENSMUSG00000037904	-0.928570329	0.007471073
mmu-miR-106a-5p	ENSMUSG00000036550;	-0.888450762	0.017970832	ENSMUSG00000008348	-0.931779028	0.006822398
mmu-miR-106a-5p	ENSMUSG00000036550;	-0.888450762	0.017970832	ENSMUSG00000037904	-0.888450762	0.017970832
mmu-miR-107-5p	ENSMUSG00000034007;	-0.920375072	0.009257777		-0.851127962	0.031594609
mmu-miR-10a-3p	ENSMUSG00000025453;E	-0.947023692	0.004135395	ENSMUSG00000027581	-0.925954253	0.008021171
mmu-miR-1198-5p	ENSMUSG00000032355;	-0.856260118	0.029506815	ENSMUSG00000028464	-0.892994598	0.01656262
mmu-miR-1198-5p	ENSMUSG00000032355;	-0.856260118	0.029506815	ENSMUSG00000039914	-0.906025668	0.012831811
mmu-miR-1198-5p	ENSMUSG00000032355;	-0.856260118	0.029506815	ENSMUSG00000034055	-0.990180222	0.000144169
mmu-miR-1198-5p	ENSMUSG00000032355;	-0.856260118	0.029506815	ENSMUSG00000028584	-0.866273303	0.025628538
mmu-miR-1198-5p	ENSMUSG00000032355;	-0.856260118	0.029506815	ENSMUSG00000019777	-0.9172804	0.009980792
mmu-miR-1198-5p	ENSMUSG00000032355;	-0.856260118	0.029506815		-0.898452551	0.014944254
mmu-miR-1224-5p	ENSMUSG00000022797;	-0.89583894	0.01570924	ENSMUSG00000031949	-0.931189574	0.006939408
mmu-miR-1231-5p	ENSMUSG00000037174;	-0.91303845	0.011014652	ENSMUSG00000024043	-0.923294438	0.008599957
mmu-miR-1231-5p	ENSMUSG00000037174;	-0.91303845	0.011014652	ENSMUSG00000009995	-0.887014449	0.01842743
mmu-miR-1231-5p	ENSMUSG00000037174;	-0.91303845	0.011014652	ENSMUSG00000034684	-0.85827271	0.028706526
mmu-miR-1231-5p	ENSMUSG00000037174;	-0.91303845	0.011014652	ENSMUSG00000041961	-0.963529341	0.001970909
mmu-miR-1231-5p	ENSMUSG00000037174;	-0.91303845	0.011014652	ENSMUSG00000035545	-0.860777217	0.027725199
mmu-miR-1231-5p	ENSMUSG00000037174;	-0.91303845	0.011014652	ENSMUSG00000042873	-0.915865718	0.010320091
mmu-miR-1231-5p	ENSMUSG00000037174;	-0.91303845	0.011014652	ENSMUSG00000037824	-0.889132449	0.01775605
mmu-miR-125a-3p	ENSMUSG00000040785;	-0.950011642	0.003685798	ENSMUSG00000019846	-0.905207235	0.013052614
mmu-miR-125a-3p	ENSMUSG00000040785;	-0.950011642	0.003685798	ENSMUSG00000020679	-0.883205793	0.019664742
mmu-miR-125a-3p	ENSMUSG00000040785;	-0.950011642	0.003685798	ENSMUSG00000026574	-0.875916976	0.022139667
mmu-miR-125a-3p	ENSMUSG00000040785;	-0.950011642	0.003685798	ENSMUSG00000073802	-0.901245558	0.014147111
mmu-miR-125a-3p	ENSMUSG00000040785;	-0.950011642	0.003685798	ENSMUSG00000033256	-0.880496444	0.020568329
mmu-miR-125a-3p	ENSMUSG00000040785;	-0.950011642	0.003685798	ENSMUSG00000020190	-0.865040856	0.026091885
mmu-miR-125a-3p	ENSMUSG00000040785;	-0.950011642	0.003685798	ENSMUSG00000107283	-0.916817934	0.010091105
mmu-miR-125a-3p	ENSMUSG00000040785;	-0.950011642	0.003685798	ENSMUSG00000071078	-0.86740062	0.025208173
mmu-miR-125a-3p	ENSMUSG00000038538;	-0.907994497	0.012308105	ENSMUSG00000019846	-0.905207235	0.013052614
mmu-miR-125a-3p	ENSMUSG00000038538;	-0.907994497	0.012308105	ENSMUSG00000020679	-0.883205793	0.019664742
mmu-miR-125a-3p	ENSMUSG00000038538;	-0.907994497	0.012308105	ENSMUSG00000026574	-0.875916976	0.022139667
mmu-miR-125a-3p	ENSMUSG00000038538;	-0.907994497	0.012308105	ENSMUSG00000073802	-0.901245558	0.014147111
mmu-miR-125a-3p	ENSMUSG00000038538;	-0.907994497	0.012308105	ENSMUSG00000033256	-0.880496444	0.020568329
mmu-miR-125a-3p	ENSMUSG00000038538;	-0.907994497	0.012308105	ENSMUSG00000020190	-0.865040856	0.026091885
mmu-miR-125a-3p	ENSMUSG00000038538;	-0.907994497	0.012308105	ENSMUSG00000107283	-0.916817934	0.010091105
mmu-miR-125a-3p	ENSMUSG00000038538;	-0.907994497	0.012308105	ENSMUSG00000071078	-0.86740062	0.025208173
mmu-miR-125a-5p	ENSMUSG00000022186;	-0.865639991	0.025866144	ENSMUSG00000037579	-0.891125294	0.017135268
mmu-miR-125a-5p	ENSMUSG00000022186;	-0.865639991	0.025866144	ENSMUSG00000042116	-0.861706389	0.027365243

mmu-miR-125a-5p	ENSMUSG00000022186;	-0.865639991	0.025866144	ENSMUSG00000040964	-0.872411128	0.023379876
mmu-miR-125a-5p	ENSMUSG00000022186;	-0.865639991	0.025866144	ENSMUSG00000020523	-0.926601623	0.007883272
mmu-miR-125a-5p	ENSMUSG00000022186;	-0.865639991	0.025866144	ENSMUSG00000024347	-0.852277876	0.031120956
mmu-miR-125a-5p	ENSMUSG00000022186;	-0.865639991	0.025866144		-0.853375922	0.030671823
mmu-miR-125b-5p	ENSMUSG00000022186;	-0.954619678	0.003042333	ENSMUSG00000027257	-0.8956971	0.015751282
mmu-miR-125b-5p	ENSMUSG00000022186;	-0.954619678	0.003042333	ENSMUSG00000031781	-0.905631669	0.01293788
mmu-miR-125b-5p	ENSMUSG00000022186;	-0.954619678	0.003042333	ENSMUSG00000040532	-0.921684068	0.008959907
mmu-miR-125b-5p	ENSMUSG00000022186;	-0.954619678	0.003042333		-0.896467574	0.015523565
mmu-miR-129b-5p	ENSMUSG00000034349;	-0.853713486	0.030534369	ENSMUSG00000026014	-0.853211811	0.030738753
mmu-miR-129b-5p	ENSMUSG00000034349;	-0.853713486	0.030534369		-0.957830546	0.0026299
mmu-miR-133b-3p	ENSMUSG00000032324;	-0.890923654	0.017197598	ENSMUSG00000026765	-0.926765208	0.007848611
mmu-miR-133b-3p	ENSMUSG00000032324;	-0.890923654	0.017197598	ENSMUSG00000038000	-0.880001979	0.02073533
mmu-miR-133b-3p	ENSMUSG00000032324;	-0.890923654	0.017197598	ENSMUSG00000032492	-0.908082254	0.012285008
mmu-miR-133b-3p	ENSMUSG00000032324;	-0.890923654	0.017197598	ENSMUSG00000024219	-0.892900702	0.016591161
mmu-miR-140-3p	ENSMUSG00000035139;	-0.922887075	0.008690332	ENSMUSG00000006457	-0.85917098	0.028352701
mmu-miR-140-3p	ENSMUSG00000035139;	-0.922887075	0.008690332	ENSMUSG00000074676	-0.933537764	0.006479054
mmu-miR-140-3p	ENSMUSG00000035139;	-0.922887075	0.008690332	ENSMUSG00000023495	-0.908607797	0.012147124
mmu-miR-142b	ENSMUSG00000045038;	-0.876369192	0.021982043	ENSMUSG00000059479	-0.928907296	0.007401601
mmu-miR-143-3p	ENSMUSG00000035139;	-0.857343536	0.029074709		-0.857178861	0.029140193
mmu-miR-143-5p	ENSMUSG00000089872;	-0.930369946	0.007103721	ENSMUSG00000043727	-0.884577297	0.019214747
mmu-miR-143-5p	ENSMUSG00000089872;	-0.930369946	0.007103721	ENSMUSG00000005958	-0.957773211	0.002637005
mmu-miR-145a-5p	ENSMUSG00000021846;	-0.885821385	0.018810875	ENSMUSG00000034930	-0.852112468	0.031188879
mmu-miR-145a-5p	ENSMUSG00000021846;	-0.885821385	0.018810875	ENSMUSG00000079534	-0.877718427	0.02151495
mmu-miR-145b	ENSMUSG00000027651;	-0.929853538	0.00720821	ENSMUSG00000020614	-0.94530659	0.004405249
mmu-miR-145b	ENSMUSG00000027651;	-0.929853538	0.00720821	ENSMUSG00000037916	-0.867387398	0.025213084
mmu-miR-148a-5p	ENSMUSG00000033102;	-0.851244477	0.031546462		-0.86054554	0.027815296
mmu-miR-148b-5p	ENSMUSG00000033102;	-0.855267498	0.029905354	ENSMUSG00000027901	-0.893390789	0.016442451
mmu-miR-148b-5p	ENSMUSG00000033102;	-0.855267498	0.029905354		-0.869681479	0.024367781
mmu-miR-148b-5p	ENSMUSG00000033102;	-0.855267498	0.029905354		-0.957383527	0.002685546
mmu-miR-148b-5p	ENSMUSG00000044471;	-0.897979173	0.015081445	ENSMUSG00000027901	-0.893390789	0.016442451
mmu-miR-148b-5p	ENSMUSG00000044471;	-0.897979173	0.015081445		-0.869681479	0.024367781
mmu-miR-148b-5p	ENSMUSG00000044471;	-0.897979173	0.015081445		-0.957383527	0.002685546
mmu-miR-148b-5p	ENSMUSG00000004356;	-0.91458924	0.010630961	ENSMUSG00000027901	-0.893390789	0.016442451
mmu-miR-148b-5p	ENSMUSG00000004356;	-0.91458924	0.010630961		-0.869681479	0.024367781
mmu-miR-148b-5p	ENSMUSG00000004356;	-0.91458924	0.010630961		-0.957383527	0.002685546
mmu-miR-149-5p	ENSMUSG00000048249;	-0.854551122	0.030194551	ENSMUSG00000038534	-0.907001697	0.01257087
mmu-miR-149-5p	ENSMUSG00000048249;	-0.854551122	0.030194551	ENSMUSG00000031826	-0.86846439	0.024814536
mmu-miR-149-5p	ENSMUSG00000048249;	-0.854551122	0.030194551	ENSMUSG00000052698	-0.863916792	0.026517921

mmu-miR-149-5p	ENSMUSG00000052085;	-0.855919034	0.029643475	ENSMUSG00000038534	-0.907001697	0.01257087
mmu-miR-149-5p	ENSMUSG00000052085;	-0.855919034	0.029643475	ENSMUSG00000031826	-0.86846439	0.024814536
mmu-miR-149-5p	ENSMUSG00000052085;	-0.855919034	0.029643475	ENSMUSG00000052698	-0.863916792	0.026517921
mmu-miR-150-3p	ENSMUSG00000035901;	-0.949279902	0.003793553	ENSMUSG00000031889	-0.961805507	0.002160369
mmu-miR-150-3p	ENSMUSG00000035901;	-0.949279902	0.003793553	ENSMUSG00000052712	-0.86806411	0.024962309
mmu-miR-150-3p	ENSMUSG00000035901;	-0.949279902	0.003793553	ENSMUSG00000002100	-0.888376742	0.017994229
mmu-miR-150-3p	ENSMUSG00000035901;	-0.949279902	0.003793553	ENSMUSG00000026269	-0.911982407	0.011279705
mmu-miR-150-3p	ENSMUSG00000035901;	-0.949279902	0.003793553	ENSMUSG00000033287	-0.857710088	0.028929198
mmu-miR-150-3p	ENSMUSG00000035901;	-0.949279902	0.003793553	ENSMUSG00000071064	-0.890977381	0.01718098
mmu-miR-150-5p	ENSMUSG00000024095;	-0.874313422	0.022702931	ENSMUSG00000074896	-0.866162671	0.02566997
mmu-miR-150-5p	ENSMUSG00000024095;	-0.874313422	0.022702931	ENSMUSG00000035953	-0.910852372	0.011566708
mmu-miR-151-3p	ENSMUSG00000001472;	-0.894042333	0.016245746		-0.891121056	0.017136577
mmu-miR-151-3p	ENSMUSG00000035139;	-0.935212488	0.006160162		-0.891121056	0.017136577
mmu-miR-151-5p	ENSMUSG00000000538;	-0.858557824	0.028593996	ENSMUSG00000091650	-0.896728085	0.015446931
mmu-miR-151-5p	ENSMUSG00000037257;	-0.891926	0.016888833	ENSMUSG00000091650	-0.896728085	0.015446931
mmu-miR-152-5p	ENSMUSG00000042446;	-0.908992028	0.012046792		-0.936836489	0.005858444
mmu-miR-152-5p	ENSMUSG00000002944;	-0.912801508	0.011073855		-0.936836489	0.005858444
mmu-miR-15a-5p	ENSMUSG00000041297;	-0.900051747	0.014485256	ENSMUSG00000051811	-0.90597841	0.012844511
mmu-miR-15a-5p	ENSMUSG00000041297;	-0.900051747	0.014485256	ENSMUSG00000007812	-0.876454348	0.021952421
mmu-miR-15a-5p	ENSMUSG00000024241;	-0.909306773	0.011964904	ENSMUSG00000051811	-0.90597841	0.012844511
mmu-miR-15a-5p	ENSMUSG00000024241;	-0.909306773	0.011964904	ENSMUSG00000007812	-0.876454348	0.021952421
mmu-miR-15a-5p	ENSMUSG00000096370;	-0.948537846	0.003904385	ENSMUSG00000051811	-0.90597841	0.012844511
mmu-miR-15a-5p	ENSMUSG00000096370;	-0.948537846	0.003904385	ENSMUSG00000007812	-0.876454348	0.021952421
mmu-miR-15a-5p	ENSMUSG00000052812;	-0.939953801	0.005300069	ENSMUSG00000051811	-0.90597841	0.012844511
mmu-miR-15a-5p	ENSMUSG00000052812;	-0.939953801	0.005300069	ENSMUSG00000007812	-0.876454348	0.021952421
mmu-miR-15a-5p	ENSMUSG00000038578;	-0.926260886	0.007955709	ENSMUSG00000051811	-0.90597841	0.012844511
mmu-miR-15a-5p	ENSMUSG00000038578;	-0.926260886	0.007955709	ENSMUSG00000007812	-0.876454348	0.021952421
mmu-miR-15a-5p	ENSMUSG00000031543;	-0.939953801	0.005300069	ENSMUSG00000051811	-0.90597841	0.012844511
mmu-miR-15a-5p	ENSMUSG00000031543;	-0.939953801	0.005300069	ENSMUSG00000007812	-0.876454348	0.021952421
mmu-miR-17-3p	ENSMUSG00000020074;	-0.932315859	0.006716679	ENSMUSG00000024969	-0.865515897	0.025912823
mmu-miR-17-3p	ENSMUSG00000020074;	-0.932315859	0.006716679	ENSMUSG00000025402	-0.896046116	0.015647931
mmu-miR-17-3p	ENSMUSG00000020074;	-0.932315859	0.006716679	ENSMUSG00000027376	-0.937504376	0.00573651
mmu-miR-17-3p	ENSMUSG00000020074;	-0.932315859	0.006716679	ENSMUSG00000047562	-0.932315859	0.006716679
mmu-miR-17-3p	ENSMUSG00000020074;	-0.932315859	0.006716679	ENSMUSG00000005107	-0.858294705	0.028697837
mmu-miR-17-3p	ENSMUSG00000020074;	-0.932315859	0.006716679	ENSMUSG00000070563	-0.932315859	0.006716679
mmu-miR-17-3p	ENSMUSG00000020074;	-0.932315859	0.006716679	ENSMUSG00000055994	-0.933223174	0.006539833
mmu-miR-17-3p	ENSMUSG00000020074;	-0.932315859	0.006716679	ENSMUSG00000034429	-0.932315859	0.006716679
mmu-miR-17-3p	ENSMUSG00000020074;	-0.932315859	0.006716679	ENSMUSG00000046020	-0.865308509	0.025990924

mmu-miR-17-3p	ENSMUSG00000020074;	-0.932315859	0.006716679	ENSMUSG00000033295	-0.979338961	0.000635908
mmu-miR-17-3p	ENSMUSG00000020074;	-0.932315859	0.006716679	ENSMUSG00000028057	-0.932315859	0.006716679
mmu-miR-17-3p	ENSMUSG00000020074;	-0.932315859	0.006716679	ENSMUSG00000037455	-0.932315859	0.006716679
mmu-miR-17-3p	ENSMUSG00000020074;	-0.932315859	0.006716679	ENSMUSG00000061374	-0.932315859	0.006716679
mmu-miR-17-3p	ENSMUSG00000005371;	-0.932315859	0.006716679	ENSMUSG00000024969	-0.865515897	0.025912823
mmu-miR-17-3p	ENSMUSG00000005371;	-0.932315859	0.006716679	ENSMUSG00000025402	-0.896046116	0.015647931
mmu-miR-17-3p	ENSMUSG00000005371;	-0.932315859	0.006716679	ENSMUSG00000027376	-0.937504376	0.00573651
mmu-miR-17-3p	ENSMUSG00000005371;	-0.932315859	0.006716679	ENSMUSG00000047562	-0.932315859	0.006716679
mmu-miR-17-3p	ENSMUSG00000005371;	-0.932315859	0.006716679	ENSMUSG00000005107	-0.858294705	0.028697837
mmu-miR-17-3p	ENSMUSG00000005371;	-0.932315859	0.006716679	ENSMUSG00000070563	-0.932315859	0.006716679
mmu-miR-17-3p	ENSMUSG00000005371;	-0.932315859	0.006716679	ENSMUSG00000055994	-0.933223174	0.006539833
mmu-miR-17-3p	ENSMUSG00000005371;	-0.932315859	0.006716679	ENSMUSG00000034429	-0.932315859	0.006716679
mmu-miR-17-3p	ENSMUSG00000005371;	-0.932315859	0.006716679	ENSMUSG00000046020	-0.865308509	0.025990924
mmu-miR-17-3p	ENSMUSG00000005371;	-0.932315859	0.006716679	ENSMUSG00000033295	-0.979338961	0.000635908
mmu-miR-17-3p	ENSMUSG00000005371;	-0.932315859	0.006716679	ENSMUSG00000028057	-0.932315859	0.006716679
mmu-miR-17-3p	ENSMUSG00000005371;	-0.932315859	0.006716679	ENSMUSG00000037455	-0.932315859	0.006716679
mmu-miR-17-3p	ENSMUSG00000005371;	-0.932315859	0.006716679	ENSMUSG00000061374	-0.932315859	0.006716679
mmu-miR-17-3p	ENSMUSG00000041258;	-0.932315859	0.006716679	ENSMUSG00000024969	-0.865515897	0.025912823
mmu-miR-17-3p	ENSMUSG00000041258;	-0.932315859	0.006716679	ENSMUSG00000025402	-0.896046116	0.015647931
mmu-miR-17-3p	ENSMUSG00000041258;	-0.932315859	0.006716679	ENSMUSG00000027376	-0.937504376	0.00573651
mmu-miR-17-3p	ENSMUSG00000041258;	-0.932315859	0.006716679	ENSMUSG00000047562	-0.932315859	0.006716679
mmu-miR-17-3p	ENSMUSG00000041258;	-0.932315859	0.006716679	ENSMUSG00000005107	-0.858294705	0.028697837
mmu-miR-17-3p	ENSMUSG00000041258;	-0.932315859	0.006716679	ENSMUSG00000070563	-0.932315859	0.006716679
mmu-miR-17-3p	ENSMUSG00000041258;	-0.932315859	0.006716679	ENSMUSG00000055994	-0.933223174	0.006539833
mmu-miR-17-3p	ENSMUSG00000041258;	-0.932315859	0.006716679	ENSMUSG00000034429	-0.932315859	0.006716679
mmu-miR-17-3p	ENSMUSG00000041258;	-0.932315859	0.006716679	ENSMUSG00000046020	-0.865308509	0.025990924
mmu-miR-17-3p	ENSMUSG00000041258;	-0.932315859	0.006716679	ENSMUSG00000033295	-0.979338961	0.000635908
mmu-miR-17-3p	ENSMUSG00000041258;	-0.932315859	0.006716679	ENSMUSG00000028057	-0.932315859	0.006716679
mmu-miR-17-3p	ENSMUSG00000041258;	-0.932315859	0.006716679	ENSMUSG00000037455	-0.932315859	0.006716679
mmu-miR-17-3p	ENSMUSG00000041258;	-0.932315859	0.006716679	ENSMUSG00000061374	-0.932315859	0.006716679
mmu-miR-17-3p	ENSMUSG00000009112;	-0.932315859	0.006716679	ENSMUSG00000024969	-0.865515897	0.025912823
mmu-miR-17-3p	ENSMUSG00000009112;	-0.932315859	0.006716679	ENSMUSG00000025402	-0.896046116	0.015647931
mmu-miR-17-3p	ENSMUSG00000009112;	-0.932315859	0.006716679	ENSMUSG00000027376	-0.937504376	0.00573651
mmu-miR-17-3p	ENSMUSG00000009112;	-0.932315859	0.006716679	ENSMUSG00000047562	-0.932315859	0.006716679
mmu-miR-17-3p	ENSMUSG00000009112;	-0.932315859	0.006716679	ENSMUSG00000005107	-0.858294705	0.028697837
mmu-miR-17-3p	ENSMUSG00000009112;	-0.932315859	0.006716679	ENSMUSG00000070563	-0.932315859	0.006716679
mmu-miR-17-3p	ENSMUSG00000009112;	-0.932315859	0.006716679	ENSMUSG00000055994	-0.933223174	0.006539833
mmu-miR-17-3p	ENSMUSG00000009112;	-0.932315859	0.006716679	ENSMUSG00000034429	-0.932315859	0.006716679

mmu-miR-17-3p	ENSMUSG00000009112;	-0.932315859	0.006716679	ENSMUSG00000046020	-0.865308509	0.025990924
mmu-miR-17-3p	ENSMUSG00000009112;	-0.932315859	0.006716679	ENSMUSG00000033295	-0.979338961	0.000635908
mmu-miR-17-3p	ENSMUSG00000009112;	-0.932315859	0.006716679	ENSMUSG00000028057	-0.932315859	0.006716679
mmu-miR-17-3p	ENSMUSG00000009112;	-0.932315859	0.006716679	ENSMUSG00000037455	-0.932315859	0.006716679
mmu-miR-17-3p	ENSMUSG00000009112;	-0.932315859	0.006716679	ENSMUSG00000061374	-0.932315859	0.006716679
mmu-miR-17-3p	ENSMUSG00000030849;	-0.932315859	0.006716679	ENSMUSG00000024969	-0.865515897	0.025912823
mmu-miR-17-3p	ENSMUSG00000030849;	-0.932315859	0.006716679	ENSMUSG00000025402	-0.896046116	0.015647931
mmu-miR-17-3p	ENSMUSG00000030849;	-0.932315859	0.006716679	ENSMUSG00000027376	-0.937504376	0.00573651
mmu-miR-17-3p	ENSMUSG00000030849;	-0.932315859	0.006716679	ENSMUSG00000047562	-0.932315859	0.006716679
mmu-miR-17-3p	ENSMUSG00000030849;	-0.932315859	0.006716679	ENSMUSG00000005107	-0.858294705	0.028697837
mmu-miR-17-3p	ENSMUSG00000030849;	-0.932315859	0.006716679	ENSMUSG00000070563	-0.932315859	0.006716679
mmu-miR-17-3p	ENSMUSG00000030849;	-0.932315859	0.006716679	ENSMUSG00000055994	-0.933223174	0.006539833
mmu-miR-17-3p	ENSMUSG00000030849;	-0.932315859	0.006716679	ENSMUSG00000034429	-0.932315859	0.006716679
mmu-miR-17-3p	ENSMUSG00000030849;	-0.932315859	0.006716679	ENSMUSG00000046020	-0.865308509	0.025990924
mmu-miR-17-3p	ENSMUSG00000030849;	-0.932315859	0.006716679	ENSMUSG00000033295	-0.979338961	0.000635908
mmu-miR-17-3p	ENSMUSG00000030849;	-0.932315859	0.006716679	ENSMUSG00000028057	-0.932315859	0.006716679
mmu-miR-17-3p	ENSMUSG00000030849;	-0.932315859	0.006716679	ENSMUSG00000037455	-0.932315859	0.006716679
mmu-miR-17-3p	ENSMUSG00000030849;	-0.932315859	0.006716679	ENSMUSG00000061374	-0.932315859	0.006716679
mmu-miR-17-3p	ENSMUSG00000020227;	-0.868447674	0.024820698	ENSMUSG00000024969	-0.865515897	0.025912823
mmu-miR-17-3p	ENSMUSG00000020227;	-0.868447674	0.024820698	ENSMUSG00000025402	-0.896046116	0.015647931
mmu-miR-17-3p	ENSMUSG00000020227;	-0.868447674	0.024820698	ENSMUSG00000027376	-0.937504376	0.00573651
mmu-miR-17-3p	ENSMUSG00000020227;	-0.868447674	0.024820698	ENSMUSG00000047562	-0.932315859	0.006716679
mmu-miR-17-3p	ENSMUSG00000020227;	-0.868447674	0.024820698	ENSMUSG00000005107	-0.858294705	0.028697837
mmu-miR-17-3p	ENSMUSG00000020227;	-0.868447674	0.024820698	ENSMUSG00000070563	-0.932315859	0.006716679
mmu-miR-17-3p	ENSMUSG00000020227;	-0.868447674	0.024820698	ENSMUSG00000055994	-0.933223174	0.006539833
mmu-miR-17-3p	ENSMUSG00000020227;	-0.868447674	0.024820698	ENSMUSG00000034429	-0.932315859	0.006716679
mmu-miR-17-3p	ENSMUSG00000020227;	-0.868447674	0.024820698	ENSMUSG00000046020	-0.865308509	0.025990924
mmu-miR-17-3p	ENSMUSG00000020227;	-0.868447674	0.024820698	ENSMUSG00000033295	-0.979338961	0.000635908
mmu-miR-17-3p	ENSMUSG00000020227;	-0.868447674	0.024820698	ENSMUSG00000028057	-0.932315859	0.006716679
mmu-miR-17-3p	ENSMUSG00000020227;	-0.868447674	0.024820698	ENSMUSG00000037455	-0.932315859	0.006716679
mmu-miR-17-3p	ENSMUSG00000020227;	-0.868447674	0.024820698	ENSMUSG00000061374	-0.932315859	0.006716679
mmu-miR-17-3p	ENSMUSG00000020361;	-0.932315859	0.006716679	ENSMUSG00000024969	-0.865515897	0.025912823
mmu-miR-17-3p	ENSMUSG00000020361;	-0.932315859	0.006716679	ENSMUSG00000025402	-0.896046116	0.015647931
mmu-miR-17-3p	ENSMUSG00000020361;	-0.932315859	0.006716679	ENSMUSG00000027376	-0.937504376	0.00573651
mmu-miR-17-3p	ENSMUSG00000020361;	-0.932315859	0.006716679	ENSMUSG00000047562	-0.932315859	0.006716679
mmu-miR-17-3p	ENSMUSG00000020361;	-0.932315859	0.006716679	ENSMUSG00000005107	-0.858294705	0.028697837
mmu-miR-17-3p	ENSMUSG00000020361;	-0.932315859	0.006716679	ENSMUSG00000070563	-0.932315859	0.006716679
mmu-miR-17-3p	ENSMUSG00000020361;	-0.932315859	0.006716679	ENSMUSG00000055994	-0.933223174	0.006539833

mmu-miR-17-3p	ENSMUSG00000020361;	-0.932315859	0.006716679	ENSMUSG00000034429	-0.932315859	0.006716679
mmu-miR-17-3p	ENSMUSG00000020361;	-0.932315859	0.006716679	ENSMUSG00000046020	-0.865308509	0.025990924
mmu-miR-17-3p	ENSMUSG00000020361;	-0.932315859	0.006716679	ENSMUSG00000033295	-0.979338961	0.000635908
mmu-miR-17-3p	ENSMUSG00000020361;	-0.932315859	0.006716679	ENSMUSG00000028057	-0.932315859	0.006716679
mmu-miR-17-3p	ENSMUSG00000020361;	-0.932315859	0.006716679	ENSMUSG00000037455	-0.932315859	0.006716679
mmu-miR-17-3p	ENSMUSG00000020361;	-0.932315859	0.006716679	ENSMUSG00000061374	-0.932315859	0.006716679
mmu-miR-17-3p	ENSMUSG00000020898;	-0.932315859	0.006716679	ENSMUSG00000024969	-0.865515897	0.025912823
mmu-miR-17-3p	ENSMUSG00000020898;	-0.932315859	0.006716679	ENSMUSG00000025402	-0.896046116	0.015647931
mmu-miR-17-3p	ENSMUSG00000020898;	-0.932315859	0.006716679	ENSMUSG00000027376	-0.937504376	0.00573651
mmu-miR-17-3p	ENSMUSG00000020898;	-0.932315859	0.006716679	ENSMUSG00000047562	-0.932315859	0.006716679
mmu-miR-17-3p	ENSMUSG00000020898;	-0.932315859	0.006716679	ENSMUSG00000005107	-0.858294705	0.028697837
mmu-miR-17-3p	ENSMUSG00000020898;	-0.932315859	0.006716679	ENSMUSG00000070563	-0.932315859	0.006716679
mmu-miR-17-3p	ENSMUSG00000020898;	-0.932315859	0.006716679	ENSMUSG00000055994	-0.933223174	0.006539833
mmu-miR-17-3p	ENSMUSG00000020898;	-0.932315859	0.006716679	ENSMUSG00000034429	-0.932315859	0.006716679
mmu-miR-17-3p	ENSMUSG00000020898;	-0.932315859	0.006716679	ENSMUSG00000046020	-0.865308509	0.025990924
mmu-miR-17-3p	ENSMUSG00000020898;	-0.932315859	0.006716679	ENSMUSG00000033295	-0.979338961	0.000635908
mmu-miR-17-3p	ENSMUSG00000020898;	-0.932315859	0.006716679	ENSMUSG00000028057	-0.932315859	0.006716679
mmu-miR-17-3p	ENSMUSG00000020898;	-0.932315859	0.006716679	ENSMUSG00000037455	-0.932315859	0.006716679
mmu-miR-17-3p	ENSMUSG00000020898;	-0.932315859	0.006716679	ENSMUSG00000061374	-0.932315859	0.006716679
mmu-miR-17-3p	ENSMUSG00000042029;	-0.932315859	0.006716679	ENSMUSG00000024969	-0.865515897	0.025912823
mmu-miR-17-3p	ENSMUSG00000042029;	-0.932315859	0.006716679	ENSMUSG00000025402	-0.896046116	0.015647931
mmu-miR-17-3p	ENSMUSG00000042029;	-0.932315859	0.006716679	ENSMUSG00000027376	-0.937504376	0.00573651
mmu-miR-17-3p	ENSMUSG00000042029;	-0.932315859	0.006716679	ENSMUSG00000047562	-0.932315859	0.006716679
mmu-miR-17-3p	ENSMUSG00000042029;	-0.932315859	0.006716679	ENSMUSG00000005107	-0.858294705	0.028697837
mmu-miR-17-3p	ENSMUSG00000042029;	-0.932315859	0.006716679	ENSMUSG00000070563	-0.932315859	0.006716679
mmu-miR-17-3p	ENSMUSG00000042029;	-0.932315859	0.006716679	ENSMUSG00000055994	-0.933223174	0.006539833
mmu-miR-17-3p	ENSMUSG00000042029;	-0.932315859	0.006716679	ENSMUSG00000034429	-0.932315859	0.006716679
mmu-miR-17-3p	ENSMUSG00000042029;	-0.932315859	0.006716679	ENSMUSG00000046020	-0.865308509	0.025990924
mmu-miR-17-3p	ENSMUSG00000042029;	-0.932315859	0.006716679	ENSMUSG00000033295	-0.979338961	0.000635908
mmu-miR-17-3p	ENSMUSG00000042029;	-0.932315859	0.006716679	ENSMUSG00000028057	-0.932315859	0.006716679
mmu-miR-17-3p	ENSMUSG00000042029;	-0.932315859	0.006716679	ENSMUSG00000037455	-0.932315859	0.006716679
mmu-miR-17-3p	ENSMUSG00000042029;	-0.932315859	0.006716679	ENSMUSG00000061374	-0.932315859	0.006716679
mmu-miR-17-3p	ENSMUSG00000021830;	-0.932315859	0.006716679	ENSMUSG00000024969	-0.865515897	0.025912823
mmu-miR-17-3p	ENSMUSG00000021830;	-0.932315859	0.006716679	ENSMUSG00000025402	-0.896046116	0.015647931
mmu-miR-17-3p	ENSMUSG00000021830;	-0.932315859	0.006716679	ENSMUSG00000027376	-0.937504376	0.00573651
mmu-miR-17-3p	ENSMUSG00000021830;	-0.932315859	0.006716679	ENSMUSG00000047562	-0.932315859	0.006716679
mmu-miR-17-3p	ENSMUSG00000021830;	-0.932315859	0.006716679	ENSMUSG00000005107	-0.858294705	0.028697837
mmu-miR-17-3p	ENSMUSG00000021830;	-0.932315859	0.006716679	ENSMUSG00000070563	-0.932315859	0.006716679

mmu-miR-17-3p	ENSMUSG00000021830;	-0.932315859	0.006716679	ENSMUSG00000055994	-0.933223174	0.006539833
mmu-miR-17-3p	ENSMUSG00000021830;	-0.932315859	0.006716679	ENSMUSG00000034429	-0.932315859	0.006716679
mmu-miR-17-3p	ENSMUSG00000021830;	-0.932315859	0.006716679	ENSMUSG00000046020	-0.865308509	0.025990924
mmu-miR-17-3p	ENSMUSG00000021830;	-0.932315859	0.006716679	ENSMUSG00000033295	-0.979338961	0.000635908
mmu-miR-17-3p	ENSMUSG00000021830;	-0.932315859	0.006716679	ENSMUSG00000028057	-0.932315859	0.006716679
mmu-miR-17-3p	ENSMUSG00000021830;	-0.932315859	0.006716679	ENSMUSG00000037455	-0.932315859	0.006716679
mmu-miR-17-3p	ENSMUSG00000021830;	-0.932315859	0.006716679	ENSMUSG00000061374	-0.932315859	0.006716679
mmu-miR-17-3p	ENSMUSG00000022108;	-0.932315859	0.006716679	ENSMUSG00000024969	-0.865515897	0.025912823
mmu-miR-17-3p	ENSMUSG00000022108;	-0.932315859	0.006716679	ENSMUSG00000025402	-0.896046116	0.015647931
mmu-miR-17-3p	ENSMUSG00000022108;	-0.932315859	0.006716679	ENSMUSG00000027376	-0.937504376	0.00573651
mmu-miR-17-3p	ENSMUSG00000022108;	-0.932315859	0.006716679	ENSMUSG00000047562	-0.932315859	0.006716679
mmu-miR-17-3p	ENSMUSG00000022108;	-0.932315859	0.006716679	ENSMUSG00000005107	-0.858294705	0.028697837
mmu-miR-17-3p	ENSMUSG00000022108;	-0.932315859	0.006716679	ENSMUSG00000070563	-0.932315859	0.006716679
mmu-miR-17-3p	ENSMUSG00000022108;	-0.932315859	0.006716679	ENSMUSG00000055994	-0.933223174	0.006539833
mmu-miR-17-3p	ENSMUSG00000022108;	-0.932315859	0.006716679	ENSMUSG00000034429	-0.932315859	0.006716679
mmu-miR-17-3p	ENSMUSG00000022108;	-0.932315859	0.006716679	ENSMUSG00000046020	-0.865308509	0.025990924
mmu-miR-17-3p	ENSMUSG00000022108;	-0.932315859	0.006716679	ENSMUSG00000033295	-0.979338961	0.000635908
mmu-miR-17-3p	ENSMUSG00000022108;	-0.932315859	0.006716679	ENSMUSG00000028057	-0.932315859	0.006716679
mmu-miR-17-3p	ENSMUSG00000022108;	-0.932315859	0.006716679	ENSMUSG00000037455	-0.932315859	0.006716679
mmu-miR-17-3p	ENSMUSG00000022108;	-0.932315859	0.006716679	ENSMUSG00000061374	-0.932315859	0.006716679
mmu-miR-17-3p	ENSMUSG00000037210;	-0.932315859	0.006716679	ENSMUSG00000024969	-0.865515897	0.025912823
mmu-miR-17-3p	ENSMUSG00000037210;	-0.932315859	0.006716679	ENSMUSG00000025402	-0.896046116	0.015647931
mmu-miR-17-3p	ENSMUSG00000037210;	-0.932315859	0.006716679	ENSMUSG00000027376	-0.937504376	0.00573651
mmu-miR-17-3p	ENSMUSG00000037210;	-0.932315859	0.006716679	ENSMUSG00000047562	-0.932315859	0.006716679
mmu-miR-17-3p	ENSMUSG00000037210;	-0.932315859	0.006716679	ENSMUSG00000005107	-0.858294705	0.028697837
mmu-miR-17-3p	ENSMUSG00000037210;	-0.932315859	0.006716679	ENSMUSG00000070563	-0.932315859	0.006716679
mmu-miR-17-3p	ENSMUSG00000037210;	-0.932315859	0.006716679	ENSMUSG00000055994	-0.933223174	0.006539833
mmu-miR-17-3p	ENSMUSG00000037210;	-0.932315859	0.006716679	ENSMUSG00000034429	-0.932315859	0.006716679
mmu-miR-17-3p	ENSMUSG00000037210;	-0.932315859	0.006716679	ENSMUSG00000046020	-0.865308509	0.025990924
mmu-miR-17-3p	ENSMUSG00000037210;	-0.932315859	0.006716679	ENSMUSG00000033295	-0.979338961	0.000635908
mmu-miR-17-3p	ENSMUSG00000037210;	-0.932315859	0.006716679	ENSMUSG00000028057	-0.932315859	0.006716679
mmu-miR-17-3p	ENSMUSG00000037210;	-0.932315859	0.006716679	ENSMUSG00000037455	-0.932315859	0.006716679
mmu-miR-17-3p	ENSMUSG00000037210;	-0.932315859	0.006716679	ENSMUSG00000061374	-0.932315859	0.006716679
mmu-miR-17-3p	ENSMUSG00000029173;	-0.932315859	0.006716679	ENSMUSG00000024969	-0.865515897	0.025912823
mmu-miR-17-3p	ENSMUSG00000029173;	-0.932315859	0.006716679	ENSMUSG00000025402	-0.896046116	0.015647931
mmu-miR-17-3p	ENSMUSG00000029173;	-0.932315859	0.006716679	ENSMUSG00000027376	-0.937504376	0.00573651
mmu-miR-17-3p	ENSMUSG00000029173;	-0.932315859	0.006716679	ENSMUSG00000047562	-0.932315859	0.006716679
mmu-miR-17-3p	ENSMUSG00000029173;	-0.932315859	0.006716679	ENSMUSG00000005107	-0.858294705	0.028697837

mmu-miR-17-3p	ENSMUSG00000029173;	-0.932315859	0.006716679	ENSMUSG00000070563	-0.932315859	0.006716679
mmu-miR-17-3p	ENSMUSG00000029173;	-0.932315859	0.006716679	ENSMUSG00000055994	-0.933223174	0.006539833
mmu-miR-17-3p	ENSMUSG00000029173;	-0.932315859	0.006716679	ENSMUSG00000034429	-0.932315859	0.006716679
mmu-miR-17-3p	ENSMUSG00000029173;	-0.932315859	0.006716679	ENSMUSG00000046020	-0.865308509	0.025990924
mmu-miR-17-3p	ENSMUSG00000029173;	-0.932315859	0.006716679	ENSMUSG00000033295	-0.979338961	0.000635908
mmu-miR-17-3p	ENSMUSG00000029173;	-0.932315859	0.006716679	ENSMUSG00000028057	-0.932315859	0.006716679
mmu-miR-17-3p	ENSMUSG00000029173;	-0.932315859	0.006716679	ENSMUSG00000037455	-0.932315859	0.006716679
mmu-miR-17-3p	ENSMUSG00000029173;	-0.932315859	0.006716679	ENSMUSG00000061374	-0.932315859	0.006716679
mmu-miR-17-3p	ENSMUSG00000049313;	-0.932315859	0.006716679	ENSMUSG00000024969	-0.865515897	0.025912823
mmu-miR-17-3p	ENSMUSG00000049313;	-0.932315859	0.006716679	ENSMUSG00000025402	-0.896046116	0.015647931
mmu-miR-17-3p	ENSMUSG00000049313;	-0.932315859	0.006716679	ENSMUSG00000027376	-0.937504376	0.00573651
mmu-miR-17-3p	ENSMUSG00000049313;	-0.932315859	0.006716679	ENSMUSG00000047562	-0.932315859	0.006716679
mmu-miR-17-3p	ENSMUSG00000049313;	-0.932315859	0.006716679	ENSMUSG00000005107	-0.858294705	0.028697837
mmu-miR-17-3p	ENSMUSG00000049313;	-0.932315859	0.006716679	ENSMUSG00000070563	-0.932315859	0.006716679
mmu-miR-17-3p	ENSMUSG00000049313;	-0.932315859	0.006716679	ENSMUSG00000055994	-0.933223174	0.006539833
mmu-miR-17-3p	ENSMUSG00000049313;	-0.932315859	0.006716679	ENSMUSG00000034429	-0.932315859	0.006716679
mmu-miR-17-3p	ENSMUSG00000049313;	-0.932315859	0.006716679	ENSMUSG00000046020	-0.865308509	0.025990924
mmu-miR-17-3p	ENSMUSG00000049313;	-0.932315859	0.006716679	ENSMUSG00000033295	-0.979338961	0.000635908
mmu-miR-17-3p	ENSMUSG00000049313;	-0.932315859	0.006716679	ENSMUSG00000028057	-0.932315859	0.006716679
mmu-miR-17-3p	ENSMUSG00000049313;	-0.932315859	0.006716679	ENSMUSG00000037455	-0.932315859	0.006716679
mmu-miR-17-3p	ENSMUSG00000049313;	-0.932315859	0.006716679	ENSMUSG00000061374	-0.932315859	0.006716679
mmu-miR-181b-5p	ENSMUSG00000048000;	-0.88429145	0.019308123	ENSMUSG00000004056	-0.91679313	0.010097038
mmu-miR-181b-5p	ENSMUSG00000048000;	-0.88429145	0.019308123	ENSMUSG00000050530	-0.899464592	0.014652978
mmu-miR-181b-5p	ENSMUSG00000039033;	-0.858580064	0.028585226	ENSMUSG00000004056	-0.91679313	0.010097038
mmu-miR-181b-5p	ENSMUSG00000039033;	-0.858580064	0.028585226	ENSMUSG00000050530	-0.899464592	0.014652978
mmu-miR-181d-5p	ENSMUSG00000039033;	-0.882257312	0.019978857	ENSMUSG00000040390	-0.879252496	0.020989693
mmu-miR-1843a-5p	ENSMUSG00000044471;	-0.912004491	0.011274131	ENSMUSG00000002104	-0.920576631	0.009211603
mmu-miR-1843a-5p	ENSMUSG00000044471;	-0.912004491	0.011274131	ENSMUSG00000028357	-0.860739258	0.027739951
mmu-miR-1843a-5p	ENSMUSG00000044471;	-0.912004491	0.011274131	ENSMUSG00000024844	-0.935549226	0.006096992
mmu-miR-1843a-5p	ENSMUSG00000044471;	-0.912004491	0.011274131	ENSMUSG00000015575	-0.860739258	0.027739951
mmu-miR-1843a-5p	ENSMUSG00000044471;	-0.912004491	0.011274131		-0.860739258	0.027739951
mmu-miR-1843a-5p	ENSMUSG00000044471;	-0.912004491	0.011274131		-0.873556737	0.022971067
mmu-miR-1843a-5p	ENSMUSG00000044471;	-0.912004491	0.011274131		-0.863104743	0.026827737
mmu-miR-1843a-5p	ENSMUSG00000013076;	-0.900763041	0.01428332	ENSMUSG00000002104	-0.920576631	0.009211603
mmu-miR-1843a-5p	ENSMUSG00000013076;	-0.900763041	0.01428332	ENSMUSG00000028357	-0.860739258	0.027739951
mmu-miR-1843a-5p	ENSMUSG00000013076;	-0.900763041	0.01428332	ENSMUSG00000024844	-0.935549226	0.006096992
mmu-miR-1843a-5p	ENSMUSG00000013076;	-0.900763041	0.01428332	ENSMUSG00000015575	-0.860739258	0.027739951
mmu-miR-1843a-5p	ENSMUSG00000013076;	-0.900763041	0.01428332		-0.860739258	0.027739951

mmu-miR-1843a-5p	ENSMUSG00000013076;	-0.900763041	0.01428332		-0.873556737	0.022971067
mmu-miR-1843a-5p	ENSMUSG00000013076;	-0.900763041	0.01428332		-0.863104743	0.026827737
mmu-miR-1843a-5p	ENSMUSG00000021870;	-0.860739258	0.027739951	ENSMUSG00000002104	-0.920576631	0.009211603
mmu-miR-1843a-5p	ENSMUSG00000021870;	-0.860739258	0.027739951	ENSMUSG00000028357	-0.860739258	0.027739951
mmu-miR-1843a-5p	ENSMUSG00000021870;	-0.860739258	0.027739951	ENSMUSG00000024844	-0.935549226	0.006096992
mmu-miR-1843a-5p	ENSMUSG00000021870;	-0.860739258	0.027739951	ENSMUSG00000015575	-0.860739258	0.027739951
mmu-miR-1843a-5p	ENSMUSG00000021870;	-0.860739258	0.027739951		-0.860739258	0.027739951
mmu-miR-1843a-5p	ENSMUSG00000021870;	-0.860739258	0.027739951		-0.873556737	0.022971067
mmu-miR-1843a-5p	ENSMUSG00000021870;	-0.860739258	0.027739951		-0.863104743	0.026827737
mmu-miR-1843a-5p	ENSMUSG00000030516;	-0.860739258	0.027739951	ENSMUSG00000002104	-0.920576631	0.009211603
mmu-miR-1843a-5p	ENSMUSG00000030516;	-0.860739258	0.027739951	ENSMUSG00000028357	-0.860739258	0.027739951
mmu-miR-1843a-5p	ENSMUSG00000030516;	-0.860739258	0.027739951	ENSMUSG00000024844	-0.935549226	0.006096992
mmu-miR-1843a-5p	ENSMUSG00000030516;	-0.860739258	0.027739951	ENSMUSG00000015575	-0.860739258	0.027739951
mmu-miR-1843a-5p	ENSMUSG00000030516;	-0.860739258	0.027739951		-0.860739258	0.027739951
mmu-miR-1843a-5p	ENSMUSG00000030516;	-0.860739258	0.027739951		-0.873556737	0.022971067
mmu-miR-1843a-5p	ENSMUSG00000030516;	-0.860739258	0.027739951		-0.863104743	0.026827737
mmu-miR-1843a-5p	ENSMUSG00000045103;	-0.860739258	0.027739951	ENSMUSG00000002104	-0.920576631	0.009211603
mmu-miR-1843a-5p	ENSMUSG00000045103;	-0.860739258	0.027739951	ENSMUSG00000028357	-0.860739258	0.027739951
mmu-miR-1843a-5p	ENSMUSG00000045103;	-0.860739258	0.027739951	ENSMUSG00000024844	-0.935549226	0.006096992
mmu-miR-1843a-5p	ENSMUSG00000045103;	-0.860739258	0.027739951	ENSMUSG00000015575	-0.860739258	0.027739951
mmu-miR-1843a-5p	ENSMUSG00000045103;	-0.860739258	0.027739951		-0.860739258	0.027739951
mmu-miR-1843a-5p	ENSMUSG00000045103;	-0.860739258	0.027739951		-0.873556737	0.022971067
mmu-miR-1843a-5p	ENSMUSG00000045103;	-0.860739258	0.027739951		-0.863104743	0.026827737
mmu-miR-1843a-5p	ENSMUSG00000068457;	-0.860739258	0.027739951	ENSMUSG00000002104	-0.920576631	0.009211603
mmu-miR-1843a-5p	ENSMUSG00000068457;	-0.860739258	0.027739951	ENSMUSG00000028357	-0.860739258	0.027739951
mmu-miR-1843a-5p	ENSMUSG00000068457;	-0.860739258	0.027739951	ENSMUSG00000024844	-0.935549226	0.006096992
mmu-miR-1843a-5p	ENSMUSG00000068457;	-0.860739258	0.027739951	ENSMUSG00000015575	-0.860739258	0.027739951
mmu-miR-1843a-5p	ENSMUSG00000068457;	-0.860739258	0.027739951		-0.860739258	0.027739951
mmu-miR-1843a-5p	ENSMUSG00000068457;	-0.860739258	0.027739951		-0.873556737	0.022971067
mmu-miR-1843a-5p	ENSMUSG00000068457;	-0.860739258	0.027739951		-0.863104743	0.026827737
mmu-miR-1843b-5p	ENSMUSG00000042807;	-0.926662497	0.007870365	ENSMUSG00000029071	-0.900257663	0.014426656
mmu-miR-1843b-5p	ENSMUSG00000042807;	-0.926662497	0.007870365	ENSMUSG00000049791	-0.88252632	0.019889526
mmu-miR-1843b-5p	ENSMUSG00000042807;	-0.926662497	0.007870365	ENSMUSG00000074793	-0.97847005	0.000690318
mmu-miR-1843b-5p	ENSMUSG00000042807;	-0.926662497	0.007870365	ENSMUSG00000067786	-0.892619095	0.016676902
mmu-miR-1843b-5p	ENSMUSG00000042807;	-0.926662497	0.007870365	ENSMUSG00000079497	-0.926662497	0.007870365
mmu-miR-1843b-5p	ENSMUSG00000042807;	-0.926662497	0.007870365	ENSMUSG00000095463	-0.93614467	0.00598607
mmu-miR-1843b-5p	ENSMUSG00000042807;	-0.926662497	0.007870365	ENSMUSG00000022066	-0.903797249	0.013437277
mmu-miR-1843b-5p	ENSMUSG00000042807;	-0.926662497	0.007870365	ENSMUSG00000030246	-0.93896329	0.005474524

mmu-miR-1843b-5p	ENSMUSG00000038349;	-0.926662497	0.007870365	ENSMUSG00000029071	-0.900257663	0.014426656
mmu-miR-1843b-5p	ENSMUSG00000038349;	-0.926662497	0.007870365	ENSMUSG00000049791	-0.88252632	0.019889526
mmu-miR-1843b-5p	ENSMUSG00000038349;	-0.926662497	0.007870365	ENSMUSG00000074793	-0.97847005	0.000690318
mmu-miR-1843b-5p	ENSMUSG00000038349;	-0.926662497	0.007870365	ENSMUSG00000067786	-0.892619095	0.016676902
mmu-miR-1843b-5p	ENSMUSG00000038349;	-0.926662497	0.007870365	ENSMUSG00000079497	-0.926662497	0.007870365
mmu-miR-1843b-5p	ENSMUSG00000038349;	-0.926662497	0.007870365	ENSMUSG00000095463	-0.93614467	0.00598607
mmu-miR-1843b-5p	ENSMUSG00000038349;	-0.926662497	0.007870365	ENSMUSG00000022066	-0.903797249	0.013437277
mmu-miR-1843b-5p	ENSMUSG00000038349;	-0.926662497	0.007870365	ENSMUSG00000030246	-0.93896329	0.005474524
mmu-miR-1843b-5p	ENSMUSG00000046079;	-0.926662497	0.007870365	ENSMUSG00000029071	-0.900257663	0.014426656
mmu-miR-1843b-5p	ENSMUSG00000046079;	-0.926662497	0.007870365	ENSMUSG00000049791	-0.88252632	0.019889526
mmu-miR-1843b-5p	ENSMUSG00000046079;	-0.926662497	0.007870365	ENSMUSG00000074793	-0.97847005	0.000690318
mmu-miR-1843b-5p	ENSMUSG00000046079;	-0.926662497	0.007870365	ENSMUSG00000067786	-0.892619095	0.016676902
mmu-miR-1843b-5p	ENSMUSG00000046079;	-0.926662497	0.007870365	ENSMUSG00000079497	-0.926662497	0.007870365
mmu-miR-1843b-5p	ENSMUSG00000046079;	-0.926662497	0.007870365	ENSMUSG00000095463	-0.93614467	0.00598607
mmu-miR-1843b-5p	ENSMUSG00000046079;	-0.926662497	0.007870365	ENSMUSG00000022066	-0.903797249	0.013437277
mmu-miR-1843b-5p	ENSMUSG00000046079;	-0.926662497	0.007870365	ENSMUSG00000030246	-0.93896329	0.005474524
mmu-miR-1843b-5p	ENSMUSG00000032621;	-0.926662497	0.007870365	ENSMUSG00000029071	-0.900257663	0.014426656
mmu-miR-1843b-5p	ENSMUSG00000032621;	-0.926662497	0.007870365	ENSMUSG00000049791	-0.88252632	0.019889526
mmu-miR-1843b-5p	ENSMUSG00000032621;	-0.926662497	0.007870365	ENSMUSG00000074793	-0.97847005	0.000690318
mmu-miR-1843b-5p	ENSMUSG00000032621;	-0.926662497	0.007870365	ENSMUSG00000067786	-0.892619095	0.016676902
mmu-miR-1843b-5p	ENSMUSG00000032621;	-0.926662497	0.007870365	ENSMUSG00000079497	-0.926662497	0.007870365
mmu-miR-1843b-5p	ENSMUSG00000032621;	-0.926662497	0.007870365	ENSMUSG00000095463	-0.93614467	0.00598607
mmu-miR-1843b-5p	ENSMUSG00000032621;	-0.926662497	0.007870365	ENSMUSG00000022066	-0.903797249	0.013437277
mmu-miR-1843b-5p	ENSMUSG00000032621;	-0.926662497	0.007870365	ENSMUSG00000030246	-0.93896329	0.005474524
mmu-miR-1843b-5p	ENSMUSG00000037487;	-0.926662497	0.007870365	ENSMUSG00000029071	-0.900257663	0.014426656
mmu-miR-1843b-5p	ENSMUSG00000037487;	-0.926662497	0.007870365	ENSMUSG00000049791	-0.88252632	0.019889526
mmu-miR-1843b-5p	ENSMUSG00000037487;	-0.926662497	0.007870365	ENSMUSG00000074793	-0.97847005	0.000690318
mmu-miR-1843b-5p	ENSMUSG00000037487;	-0.926662497	0.007870365	ENSMUSG00000067786	-0.892619095	0.016676902
mmu-miR-1843b-5p	ENSMUSG00000037487;	-0.926662497	0.007870365	ENSMUSG00000079497	-0.926662497	0.007870365
mmu-miR-1843b-5p	ENSMUSG00000037487;	-0.926662497	0.007870365	ENSMUSG00000095463	-0.93614467	0.00598607
mmu-miR-1843b-5p	ENSMUSG00000037487;	-0.926662497	0.007870365	ENSMUSG00000022066	-0.903797249	0.013437277
mmu-miR-1843b-5p	ENSMUSG00000037487;	-0.926662497	0.007870365	ENSMUSG00000030246	-0.93896329	0.005474524
mmu-miR-1843b-5p	ENSMUSG00000013662;	-0.926662497	0.007870365	ENSMUSG00000029071	-0.900257663	0.014426656
mmu-miR-1843b-5p	ENSMUSG00000013662;	-0.926662497	0.007870365	ENSMUSG00000049791	-0.88252632	0.019889526
mmu-miR-1843b-5p	ENSMUSG00000013662;	-0.926662497	0.007870365	ENSMUSG00000074793	-0.97847005	0.000690318
mmu-miR-1843b-5p	ENSMUSG00000013662;	-0.926662497	0.007870365	ENSMUSG00000067786	-0.892619095	0.016676902
mmu-miR-1843b-5p	ENSMUSG00000013662;	-0.926662497	0.007870365	ENSMUSG00000079497	-0.926662497	0.007870365
mmu-miR-1843b-5p	ENSMUSG00000013662;	-0.926662497	0.007870365	ENSMUSG00000095463	-0.93614467	0.00598607

mmu-miR-1843b-5p	ENSMUSG00000013662;	-0.926662497	0.007870365	ENSMUSG00000022066	-0.903797249	0.013437277
mmu-miR-1843b-5p	ENSMUSG00000013662;	-0.926662497	0.007870365	ENSMUSG00000030246	-0.93896329	0.005474524
mmu-miR-1843b-5p	ENSMUSG00000037514;	-0.926662497	0.007870365	ENSMUSG00000029071	-0.900257663	0.014426656
mmu-miR-1843b-5p	ENSMUSG00000037514;	-0.926662497	0.007870365	ENSMUSG00000049791	-0.88252632	0.019889526
mmu-miR-1843b-5p	ENSMUSG00000037514;	-0.926662497	0.007870365	ENSMUSG00000074793	-0.97847005	0.000690318
mmu-miR-1843b-5p	ENSMUSG00000037514;	-0.926662497	0.007870365	ENSMUSG00000067786	-0.892619095	0.016676902
mmu-miR-1843b-5p	ENSMUSG00000037514;	-0.926662497	0.007870365	ENSMUSG00000079497	-0.926662497	0.007870365
mmu-miR-1843b-5p	ENSMUSG00000037514;	-0.926662497	0.007870365	ENSMUSG00000095463	-0.93614467	0.00598607
mmu-miR-1843b-5p	ENSMUSG00000037514;	-0.926662497	0.007870365	ENSMUSG00000022066	-0.903797249	0.013437277
mmu-miR-1843b-5p	ENSMUSG00000037514;	-0.926662497	0.007870365	ENSMUSG00000030246	-0.93896329	0.005474524
mmu-miR-1843b-5p	ENSMUSG00000066894;	-0.926662497	0.007870365	ENSMUSG00000029071	-0.900257663	0.014426656
mmu-miR-1843b-5p	ENSMUSG00000066894;	-0.926662497	0.007870365	ENSMUSG00000049791	-0.88252632	0.019889526
mmu-miR-1843b-5p	ENSMUSG00000066894;	-0.926662497	0.007870365	ENSMUSG00000074793	-0.97847005	0.000690318
mmu-miR-1843b-5p	ENSMUSG00000066894;	-0.926662497	0.007870365	ENSMUSG00000067786	-0.892619095	0.016676902
mmu-miR-1843b-5p	ENSMUSG00000066894;	-0.926662497	0.007870365	ENSMUSG00000079497	-0.926662497	0.007870365
mmu-miR-1843b-5p	ENSMUSG00000066894;	-0.926662497	0.007870365	ENSMUSG00000095463	-0.93614467	0.00598607
mmu-miR-1843b-5p	ENSMUSG00000066894;	-0.926662497	0.007870365	ENSMUSG00000022066	-0.903797249	0.013437277
mmu-miR-1843b-5p	ENSMUSG00000066894;	-0.926662497	0.007870365	ENSMUSG00000030246	-0.93896329	0.005474524
mmu-miR-1843b-5p	ENSMUSG00000030516;	-0.926662497	0.007870365	ENSMUSG00000029071	-0.900257663	0.014426656
mmu-miR-1843b-5p	ENSMUSG00000030516;	-0.926662497	0.007870365	ENSMUSG00000049791	-0.88252632	0.019889526
mmu-miR-1843b-5p	ENSMUSG00000030516;	-0.926662497	0.007870365	ENSMUSG00000074793	-0.97847005	0.000690318
mmu-miR-1843b-5p	ENSMUSG00000030516;	-0.926662497	0.007870365	ENSMUSG00000067786	-0.892619095	0.016676902
mmu-miR-1843b-5p	ENSMUSG00000030516;	-0.926662497	0.007870365	ENSMUSG00000079497	-0.926662497	0.007870365
mmu-miR-1843b-5p	ENSMUSG00000030516;	-0.926662497	0.007870365	ENSMUSG00000095463	-0.93614467	0.00598607
mmu-miR-1843b-5p	ENSMUSG00000030516;	-0.926662497	0.007870365	ENSMUSG00000022066	-0.903797249	0.013437277
mmu-miR-1843b-5p	ENSMUSG00000030516;	-0.926662497	0.007870365	ENSMUSG00000030246	-0.93896329	0.005474524
mmu-miR-1843b-5p	ENSMUSG00000045103;	-0.926662497	0.007870365	ENSMUSG00000029071	-0.900257663	0.014426656
mmu-miR-1843b-5p	ENSMUSG00000045103;	-0.926662497	0.007870365	ENSMUSG00000049791	-0.88252632	0.019889526
mmu-miR-1843b-5p	ENSMUSG00000045103;	-0.926662497	0.007870365	ENSMUSG00000074793	-0.97847005	0.000690318
mmu-miR-1843b-5p	ENSMUSG00000045103;	-0.926662497	0.007870365	ENSMUSG00000067786	-0.892619095	0.016676902
mmu-miR-1843b-5p	ENSMUSG00000045103;	-0.926662497	0.007870365	ENSMUSG00000079497	-0.926662497	0.007870365
mmu-miR-1843b-5p	ENSMUSG00000045103;	-0.926662497	0.007870365	ENSMUSG00000095463	-0.93614467	0.00598607
mmu-miR-1843b-5p	ENSMUSG00000045103;	-0.926662497	0.007870365	ENSMUSG00000022066	-0.903797249	0.013437277
mmu-miR-1843b-5p	ENSMUSG00000045103;	-0.926662497	0.007870365	ENSMUSG00000030246	-0.93896329	0.005474524
mmu-miR-1843b-5p	ENSMUSG00000068457;	-0.926662497	0.007870365	ENSMUSG00000029071	-0.900257663	0.014426656
mmu-miR-1843b-5p	ENSMUSG00000068457;	-0.926662497	0.007870365	ENSMUSG00000049791	-0.88252632	0.019889526
mmu-miR-1843b-5p	ENSMUSG00000068457;	-0.926662497	0.007870365	ENSMUSG00000074793	-0.97847005	0.000690318
mmu-miR-1843b-5p	ENSMUSG00000068457;	-0.926662497	0.007870365	ENSMUSG00000067786	-0.892619095	0.016676902

mmu-miR-1843b-5p	ENSMUSG00000068457;	-0.926662497	0.007870365	ENSMUSG00000079497	-0.926662497	0.007870365
mmu-miR-1843b-5p	ENSMUSG00000068457;	-0.926662497	0.007870365	ENSMUSG00000095463	-0.93614467	0.00598607
mmu-miR-1843b-5p	ENSMUSG00000068457;	-0.926662497	0.007870365	ENSMUSG00000022066	-0.903797249	0.013437277
mmu-miR-1843b-5p	ENSMUSG00000068457;	-0.926662497	0.007870365	ENSMUSG00000030246	-0.93896329	0.005474524
mmu-miR-185-3p	ENSMUSG00000048410;	-0.893635985	0.016368291	ENSMUSG00000002233	-0.914590853	0.010630566
mmu-miR-185-3p	ENSMUSG00000048410;	-0.893635985	0.016368291	ENSMUSG00000014599	-0.893906874	0.016286548
mmu-miR-185-3p	ENSMUSG00000048410;	-0.893635985	0.016368291	ENSMUSG00000029674	-0.868655277	0.024744212
mmu-miR-185-3p	ENSMUSG00000048410;	-0.893635985	0.016368291	ENSMUSG00000021448	-0.913130524	0.010991687
mmu-miR-185-3p	ENSMUSG00000048410;	-0.893635985	0.016368291	ENSMUSG00000027996	-0.898888729	0.014818379
mmu-miR-185-3p	ENSMUSG00000048410;	-0.893635985	0.016368291	ENSMUSG00000028047	-0.956474718	0.002800447
mmu-miR-185-3p	ENSMUSG00000048410;	-0.893635985	0.016368291	ENSMUSG00000033595	-0.889216696	0.017729592
mmu-miR-185-3p	ENSMUSG00000048410;	-0.893635985	0.016368291	ENSMUSG00000045107	-0.865307434	0.025991329
mmu-miR-185-3p	ENSMUSG00000048410;	-0.893635985	0.016368291	ENSMUSG00000025372	-0.85753787	0.028997521
mmu-miR-185-3p	ENSMUSG00000048410;	-0.893635985	0.016368291	ENSMUSG00000061702	-0.947317716	0.004090027
mmu-miR-185-3p	ENSMUSG00000048410;	-0.893635985	0.016368291	ENSMUSG00000022971	-0.971976066	0.001167007
mmu-miR-185-3p	ENSMUSG00000048410;	-0.893635985	0.016368291	ENSMUSG00000008575	-0.876049876	0.022093288
mmu-miR-185-3p	ENSMUSG00000048410;	-0.893635985	0.016368291	ENSMUSG00000008305	-0.876757017	0.021847291
mmu-miR-185-3p	ENSMUSG00000048410;	-0.893635985	0.016368291	ENSMUSG00000027655	-0.872494326	0.023350071
mmu-miR-185-3p	ENSMUSG00000048410;	-0.893635985	0.016368291	ENSMUSG00000101174	-0.853412994	0.030656713
mmu-miR-185-3p	ENSMUSG00000048410;	-0.893635985	0.016368291	ENSMUSG00000025269	-0.979307815	0.00063782
mmu-miR-185-3p	ENSMUSG00000048410;	-0.893635985	0.016368291	ENSMUSG00000022812	-0.989724396	0.00015784
mmu-miR-185-3p	ENSMUSG00000048410;	-0.893635985	0.016368291	ENSMUSG00000036667	-0.858248831	0.02871596
mmu-miR-185-3p	ENSMUSG00000048410;	-0.893635985	0.016368291	ENSMUSG00000055407	-0.863568282	0.026650677
mmu-miR-185-3p	ENSMUSG00000048410;	-0.893635985	0.016368291	ENSMUSG00000053175	-0.926992959	0.007800477
mmu-miR-185-3p	ENSMUSG00000048410;	-0.893635985	0.016368291	ENSMUSG00000026458	-0.885630885	0.018872451
mmu-miR-185-3p	ENSMUSG00000048410;	-0.893635985	0.016368291	ENSMUSG00000026827	-0.871720365	0.023628034
mmu-miR-185-3p	ENSMUSG00000048410;	-0.893635985	0.016368291	ENSMUSG00000040812	-0.879460022	0.020919113
mmu-miR-185-3p	ENSMUSG00000048410;	-0.893635985	0.016368291	ENSMUSG00000032289	-0.898624145	0.014894673
mmu-miR-185-3p	ENSMUSG00000048410;	-0.893635985	0.016368291	ENSMUSG00000002985	-0.903509361	0.01351648
mmu-miR-185-3p	ENSMUSG00000048410;	-0.893635985	0.016368291	ENSMUSG00000021972	-0.974373052	0.000976696
mmu-miR-185-3p	ENSMUSG00000048410;	-0.893635985	0.016368291	ENSMUSG00000023826	-0.869897513	0.024288886
mmu-miR-185-3p	ENSMUSG00000048410;	-0.893635985	0.016368291	ENSMUSG00000032599	-0.898704173	0.014871577
mmu-miR-185-3p	ENSMUSG00000048410;	-0.893635985	0.016368291	ENSMUSG00000028080	-0.906593219	0.012679761
mmu-miR-185-3p	ENSMUSG00000048410;	-0.893635985	0.016368291	ENSMUSG00000033278	-0.875474594	0.022294384
mmu-miR-185-3p	ENSMUSG00000048410;	-0.893635985	0.016368291	ENSMUSG00000038982	-0.855121242	0.02996429
mmu-miR-185-3p	ENSMUSG00000048410;	-0.893635985	0.016368291	ENSMUSG00000060450	-0.875048141	0.022444016
mmu-miR-188-5p	ENSMUSG00000024135;	-0.920953158	0.009125647	ENSMUSG00000020098	-0.876764647	0.021844644
mmu-miR-188-5p	ENSMUSG00000024135;	-0.920953158	0.009125647	ENSMUSG00000029556	-0.901289638	0.014134699

mmu-miR-188-5p	ENSMUSG00000024135;	-0.920953158	0.009125647	ENSMUSG00000001964	-0.880053476	0.020717908
mmu-miR-188-5p	ENSMUSG00000024135;	-0.920953158	0.009125647		-0.899303465	0.014699167
mmu-miR-1927	ENSMUSG00000020717;	-0.911240875	0.011467643	ENSMUSG00000037373	-0.941222678	0.005080629
mmu-miR-1927	ENSMUSG00000020717;	-0.911240875	0.011467643	ENSMUSG00000020400	-0.880867138	0.020443553
mmu-miR-1927	ENSMUSG00000020717;	-0.911240875	0.011467643	ENSMUSG00000068246	-0.883249365	0.019650369
mmu-miR-1934-5p	ENSMUSG00000042439;	-0.884571013	0.019216797	ENSMUSG00000030208	-0.947735582	0.004025972
mmu-miR-1934-5p	ENSMUSG00000042439;	-0.884571013	0.019216797	ENSMUSG00000036620	-0.858448129	0.028637266
mmu-miR-1934-5p	ENSMUSG00000042439;	-0.884571013	0.019216797	ENSMUSG00000025375	-0.927201746	0.007756478
mmu-miR-1934-5p	ENSMUSG00000042439;	-0.884571013	0.019216797	ENSMUSG00000040652	-0.856083364	0.029577597
mmu-miR-1934-5p	ENSMUSG00000042439;	-0.884571013	0.019216797	ENSMUSG00000022421	-0.960024544	0.002365114
mmu-miR-1934-5p	ENSMUSG00000039987;	-0.921689681	0.00895864	ENSMUSG00000030208	-0.947735582	0.004025972
mmu-miR-1934-5p	ENSMUSG00000039987;	-0.921689681	0.00895864	ENSMUSG00000036620	-0.858448129	0.028637266
mmu-miR-1934-5p	ENSMUSG00000039987;	-0.921689681	0.00895864	ENSMUSG00000025375	-0.927201746	0.007756478
mmu-miR-1934-5p	ENSMUSG00000039987;	-0.921689681	0.00895864	ENSMUSG00000040652	-0.856083364	0.029577597
mmu-miR-1934-5p	ENSMUSG00000039987;	-0.921689681	0.00895864	ENSMUSG00000022421	-0.960024544	0.002365114
mmu-miR-193a-3p	ENSMUSG00000045594;	-0.958853205	0.002504756	ENSMUSG00000025371	-0.891388114	0.01705419
mmu-miR-193a-3p	ENSMUSG00000045594;	-0.958853205	0.002504756	ENSMUSG00000056116	-0.949321358	0.003787407
mmu-miR-193a-3p	ENSMUSG00000045594;	-0.958853205	0.002504756	ENSMUSG00000056952	-0.895943987	0.01567814
mmu-miR-193a-3p	ENSMUSG00000045594;	-0.958853205	0.002504756	ENSMUSG00000056076	-0.912283996	0.011203698
mmu-miR-193a-3p	ENSMUSG00000045594;	-0.958853205	0.002504756	ENSMUSG00000027395	-0.914879541	0.010559869
mmu-miR-193a-5p	ENSMUSG00000033444;	-0.878290462	0.021318363	ENSMUSG00000026028	-0.925048378	0.008216089
mmu-miR-193a-5p	ENSMUSG00000035139;	-0.866323704	0.025609674	ENSMUSG00000026028	-0.925048378	0.008216089
mmu-miR-1943-3p	ENSMUSG00000048410;	-0.870433222	0.024093771	ENSMUSG00000000093	-0.974377618	0.000976349
mmu-miR-1943-3p	ENSMUSG00000048410;	-0.870433222	0.024093771	ENSMUSG00000002043	-0.851469063	0.031453756
mmu-miR-1943-3p	ENSMUSG00000048410;	-0.870433222	0.024093771	ENSMUSG00000025575	-0.946214216	0.004261567
mmu-miR-1943-3p	ENSMUSG00000048410;	-0.870433222	0.024093771	ENSMUSG00000040479	-0.93614547	0.005985921
mmu-miR-1943-3p	ENSMUSG00000048410;	-0.870433222	0.024093771	ENSMUSG00000022971	-0.916644499	0.010132627
mmu-miR-1943-3p	ENSMUSG00000048410;	-0.870433222	0.024093771	ENSMUSG00000038697	-0.890442759	0.017346687
mmu-miR-1943-3p	ENSMUSG00000048410;	-0.870433222	0.024093771	ENSMUSG00000070003	-0.876038563	0.022097234
mmu-miR-1954	ENSMUSG00000020700;	-0.882644143	0.01985046	ENSMUSG00000030474	-0.880424443	0.020592606
mmu-miR-1954	ENSMUSG00000020700;	-0.882644143	0.01985046	ENSMUSG00000039244	-0.854147251	0.030358172
mmu-miR-1954	ENSMUSG00000020700;	-0.882644143	0.01985046	ENSMUSG00000050966	-0.851609175	0.031395985
mmu-miR-1954	ENSMUSG00000020700;	-0.882644143	0.01985046	ENSMUSG00000054150	-0.864010653	0.026482221
mmu-miR-1954	ENSMUSG00000020700;	-0.882644143	0.01985046	ENSMUSG00000059883	-0.904721409	0.013184545
mmu-miR-1954	ENSMUSG00000020700;	-0.882644143	0.01985046	ENSMUSG00000030649	-0.850271741	0.031949482
mmu-miR-1954	ENSMUSG00000020700;	-0.882644143	0.01985046	ENSMUSG00000016541	-0.851824434	0.031307326
mmu-miR-1954	ENSMUSG00000020340;	-0.942991431	0.004782327	ENSMUSG00000030474	-0.880424443	0.020592606
mmu-miR-1954	ENSMUSG00000020340;	-0.942991431	0.004782327	ENSMUSG00000039244	-0.854147251	0.030358172

mmu-miR-1954	ENSMUSG00000020340;	-0.942991431	0.004782327	ENSMUSG00000050966	-0.851609175	0.031395985
mmu-miR-1954	ENSMUSG00000020340;	-0.942991431	0.004782327	ENSMUSG00000054150	-0.864010653	0.026482221
mmu-miR-1954	ENSMUSG00000020340;	-0.942991431	0.004782327	ENSMUSG00000059883	-0.904721409	0.013184545
mmu-miR-1954	ENSMUSG00000020340;	-0.942991431	0.004782327	ENSMUSG00000030649	-0.850271741	0.031949482
mmu-miR-1954	ENSMUSG00000020340;	-0.942991431	0.004782327	ENSMUSG00000016541	-0.851824434	0.031307326
mmu-miR-1954	ENSMUSG00000026436;	-0.935207411	0.006161117	ENSMUSG00000030474	-0.880424443	0.020592606
mmu-miR-1954	ENSMUSG00000026436;	-0.935207411	0.006161117	ENSMUSG00000039244	-0.854147251	0.030358172
mmu-miR-1954	ENSMUSG00000026436;	-0.935207411	0.006161117	ENSMUSG00000050966	-0.851609175	0.031395985
mmu-miR-1954	ENSMUSG00000026436;	-0.935207411	0.006161117	ENSMUSG00000054150	-0.864010653	0.026482221
mmu-miR-1954	ENSMUSG00000026436;	-0.935207411	0.006161117	ENSMUSG00000059883	-0.904721409	0.013184545
mmu-miR-1954	ENSMUSG00000026436;	-0.935207411	0.006161117	ENSMUSG00000030649	-0.850271741	0.031949482
mmu-miR-1954	ENSMUSG00000026436;	-0.935207411	0.006161117	ENSMUSG00000016541	-0.851824434	0.031307326
mmu-miR-1963	ENSMUSG00000038538;	-0.897978515	0.015081636	ENSMUSG00000051403	-0.850163435	0.031994504
mmu-miR-1963	ENSMUSG00000038538;	-0.897978515	0.015081636	ENSMUSG00000025077	-0.852776757	0.030916516
mmu-miR-1966-3p	ENSMUSG00000056608;	-0.866660392	0.025483824		-0.884205077	0.01933638
mmu-miR-199a-5p	ENSMUSG00000030201;	-0.870495468	0.024071148	ENSMUSG00000001249	-0.906943078	0.012586469
mmu-miR-199a-5p	ENSMUSG00000048249;	-0.860188868	0.027954274	ENSMUSG00000001249	-0.906943078	0.012586469
mmu-miR-204-3p	ENSMUSG00000040785;	-0.866205972	0.02565375	ENSMUSG00000004207	-0.903354493	0.013559179
mmu-miR-204-3p	ENSMUSG00000040785;	-0.866205972	0.02565375	ENSMUSG00000023829	-0.891377649	0.017057415
mmu-miR-204-3p	ENSMUSG00000040785;	-0.866205972	0.02565375	ENSMUSG00000040121	-0.874709466	0.022563189
mmu-miR-204-3p	ENSMUSG00000040785;	-0.866205972	0.02565375	ENSMUSG00000041653	-0.922313249	0.008818418
mmu-miR-204-3p	ENSMUSG00000040785;	-0.866205972	0.02565375	ENSMUSG00000040913	-0.885704184	0.018848747
mmu-miR-204-3p	ENSMUSG00000040785;	-0.866205972	0.02565375	ENSMUSG00000045268	-0.862897689	0.026907006
mmu-miR-204-3p	ENSMUSG00000040785;	-0.866205972	0.02565375	ENSMUSG00000026854	-0.873017228	0.023163162
mmu-miR-204-3p	ENSMUSG00000040785;	-0.866205972	0.02565375	ENSMUSG00000071757	-0.869580395	0.024404739
mmu-miR-204-3p	ENSMUSG00000040785;	-0.866205972	0.02565375	ENSMUSG00000060601	-0.916252446	0.010226791
mmu-miR-204-3p	ENSMUSG00000040785;	-0.866205972	0.02565375	ENSMUSG00000001918	-0.892782883	0.016627007
mmu-miR-204-3p	ENSMUSG00000040785;	-0.866205972	0.02565375	ENSMUSG00000054263	-0.928646207	0.007455402
mmu-miR-204-3p	ENSMUSG00000040785;	-0.866205972	0.02565375	ENSMUSG00000004207	-0.859914318	0.028061477
mmu-miR-204-3p	ENSMUSG00000040785;	-0.866205972	0.02565375	ENSMUSG00000022091	-0.853620629	0.030572151
mmu-miR-204-3p	ENSMUSG00000040785;	-0.866205972	0.02565375	ENSMUSG00000006435	-0.891045361	0.017159964
mmu-miR-204-3p	ENSMUSG00000040785;	-0.866205972	0.02565375		-0.931181455	0.006941026
mmu-miR-204-5p	n/a	-0.862601988	0.027020406	ENSMUSG00000041731	-0.914747669	0.010592135
mmu-miR-204-5p	n/a	-0.862601988	0.027020406	ENSMUSG00000028610	-0.857284472	0.029098188
mmu-miR-204-5p	n/a	-0.862601988	0.027020406	ENSMUSG00000026478	-0.888429492	0.017977554
mmu-miR-204-5p	n/a	-0.862601988	0.027020406	ENSMUSG00000004567	-0.859512029	0.028218909
mmu-miR-204-5p	n/a	-0.862601988	0.027020406		-0.938320864	0.00558915
mmu-miR-204-5p	ENSMUSG00000034349;	-0.890436897	0.017348509	ENSMUSG00000041731	-0.914747669	0.010592135

mmu-miR-204-5p	ENSMUSG00000034349;	-0.890436897	0.017348509	ENSMUSG00000028610	-0.857284472	0.029098188
mmu-miR-204-5p	ENSMUSG00000034349;	-0.890436897	0.017348509	ENSMUSG00000026478	-0.888429492	0.017977554
mmu-miR-204-5p	ENSMUSG00000034349;	-0.890436897	0.017348509	ENSMUSG00000004567	-0.859512029	0.028218909
mmu-miR-204-5p	ENSMUSG00000034349;	-0.890436897	0.017348509		-0.938320864	0.00558915
mmu-miR-20b-5p	ENSMUSG00000025220;	-0.892732816	0.016642252	ENSMUSG00000020777	-0.859466299	0.028236831
mmu-miR-212-5p	ENSMUSG00000024566;	-0.928124087	0.00756356	ENSMUSG00000031169	-0.857347708	0.029073051
mmu-miR-212-5p	ENSMUSG00000039033;	-0.901427526	0.014095908	ENSMUSG00000031169	-0.857347708	0.029073051
mmu-miR-214-3p	ENSMUSG00000048410;	-0.868265032	0.024888082	ENSMUSG00000022225	-0.863873378	0.026534442
mmu-miR-214-3p	ENSMUSG00000048410;	-0.868265032	0.024888082	ENSMUSG00000022761	-0.923528652	0.008548203
mmu-miR-214-3p	ENSMUSG00000048410;	-0.868265032	0.024888082	ENSMUSG00000028621	-0.897509554	0.015218143
mmu-miR-214-3p	ENSMUSG00000048410;	-0.868265032	0.024888082	ENSMUSG00000051703	-0.895123926	0.015921721
mmu-miR-214-3p	ENSMUSG00000048410;	-0.868265032	0.024888082	ENSMUSG00000023452	-0.92864354	0.007455952
mmu-miR-214-3p	ENSMUSG00000048410;	-0.868265032	0.024888082	ENSMUSG00000030929	-0.882489651	0.019901692
mmu-miR-214-3p	ENSMUSG00000048410;	-0.868265032	0.024888082	ENSMUSG00000034544	-0.877137764	0.021715383
mmu-miR-214-3p	ENSMUSG00000048410;	-0.868265032	0.024888082	ENSMUSG00000056692	-0.866721842	0.025460887
mmu-miR-214-3p	ENSMUSG00000048410;	-0.868265032	0.024888082	ENSMUSG00000037224	-0.868341942	0.024859696
mmu-miR-214-3p	ENSMUSG00000048410;	-0.868265032	0.024888082	ENSMUSG00000101174	-0.891349998	0.017065937
mmu-miR-214-3p	ENSMUSG00000048410;	-0.868265032	0.024888082	ENSMUSG00000041115	-0.928733045	0.007437487
mmu-miR-214-3p	ENSMUSG00000048410;	-0.868265032	0.024888082	ENSMUSG00000039515	-0.857712974	0.028928054
mmu-miR-214-3p	ENSMUSG00000048410;	-0.868265032	0.024888082	ENSMUSG00000049680	-0.899071348	0.01476583
mmu-miR-214-3p	ENSMUSG00000048410;	-0.868265032	0.024888082	ENSMUSG00000024213	-0.913755311	0.010836469
mmu-miR-214-3p	ENSMUSG00000048410;	-0.868265032	0.024888082	ENSMUSG00000002871	-0.88079869	0.020466566
mmu-miR-214-3p	ENSMUSG00000048410;	-0.868265032	0.024888082	ENSMUSG00000025147	-0.919553313	0.009447192
mmu-miR-214-3p	ENSMUSG00000048410;	-0.868265032	0.024888082	ENSMUSG00000031765	-0.933301329	0.006524707
mmu-miR-214-3p	ENSMUSG00000048410;	-0.868265032	0.024888082	ENSMUSG00000034485	-0.875585182	0.022255659
mmu-miR-214-3p	ENSMUSG00000038538;	-0.855801158	0.029690774	ENSMUSG00000022225	-0.863873378	0.026534442
mmu-miR-214-3p	ENSMUSG00000038538;	-0.855801158	0.029690774	ENSMUSG00000022761	-0.923528652	0.008548203
mmu-miR-214-3p	ENSMUSG00000038538;	-0.855801158	0.029690774	ENSMUSG00000028621	-0.897509554	0.015218143
mmu-miR-214-3p	ENSMUSG00000038538;	-0.855801158	0.029690774	ENSMUSG00000051703	-0.895123926	0.015921721
mmu-miR-214-3p	ENSMUSG00000038538;	-0.855801158	0.029690774	ENSMUSG00000023452	-0.92864354	0.007455952
mmu-miR-214-3p	ENSMUSG00000038538;	-0.855801158	0.029690774	ENSMUSG00000030929	-0.882489651	0.019901692
mmu-miR-214-3p	ENSMUSG00000038538;	-0.855801158	0.029690774	ENSMUSG00000034544	-0.877137764	0.021715383
mmu-miR-214-3p	ENSMUSG00000038538;	-0.855801158	0.029690774	ENSMUSG00000056692	-0.866721842	0.025460887
mmu-miR-214-3p	ENSMUSG00000038538;	-0.855801158	0.029690774	ENSMUSG00000037224	-0.868341942	0.024859696
mmu-miR-214-3p	ENSMUSG00000038538;	-0.855801158	0.029690774	ENSMUSG00000101174	-0.891349998	0.017065937
mmu-miR-214-3p	ENSMUSG00000038538;	-0.855801158	0.029690774	ENSMUSG00000041115	-0.928733045	0.007437487
mmu-miR-214-3p	ENSMUSG00000038538;	-0.855801158	0.029690774	ENSMUSG00000039515	-0.857712974	0.028928054
mmu-miR-214-3p	ENSMUSG00000038538;	-0.855801158	0.029690774	ENSMUSG00000049680	-0.899071348	0.01476583

mmu-miR-214-3p	ENSMUSG00000038538;	-0.855801158	0.029690774	ENSMUSG00000024213	-0.913755311	0.010836469
mmu-miR-214-3p	ENSMUSG00000038538;	-0.855801158	0.029690774	ENSMUSG00000002871	-0.88079869	0.020466566
mmu-miR-214-3p	ENSMUSG00000038538;	-0.855801158	0.029690774	ENSMUSG00000025147	-0.919553313	0.009447192
mmu-miR-214-3p	ENSMUSG00000038538;	-0.855801158	0.029690774	ENSMUSG00000031765	-0.933301329	0.006524707
mmu-miR-214-3p	ENSMUSG00000038538;	-0.855801158	0.029690774	ENSMUSG00000034485	-0.875585182	0.022255659
mmu-miR-214-3p	n/a	-0.888835974	0.01784931	ENSMUSG00000022225	-0.863873378	0.026534442
mmu-miR-214-3p	n/a	-0.888835974	0.01784931	ENSMUSG00000022761	-0.923528652	0.008548203
mmu-miR-214-3p	n/a	-0.888835974	0.01784931	ENSMUSG00000028621	-0.897509554	0.015218143
mmu-miR-214-3p	n/a	-0.888835974	0.01784931	ENSMUSG00000051703	-0.895123926	0.015921721
mmu-miR-214-3p	n/a	-0.888835974	0.01784931	ENSMUSG00000023452	-0.92864354	0.007455952
mmu-miR-214-3p	n/a	-0.888835974	0.01784931	ENSMUSG00000030929	-0.882489651	0.019901692
mmu-miR-214-3p	n/a	-0.888835974	0.01784931	ENSMUSG00000034544	-0.877137764	0.021715383
mmu-miR-214-3p	n/a	-0.888835974	0.01784931	ENSMUSG00000056692	-0.866721842	0.025460887
mmu-miR-214-3p	n/a	-0.888835974	0.01784931	ENSMUSG00000037224	-0.868341942	0.024859696
mmu-miR-214-3p	n/a	-0.888835974	0.01784931	ENSMUSG00000101174	-0.891349998	0.017065937
mmu-miR-214-3p	n/a	-0.888835974	0.01784931	ENSMUSG00000041115	-0.928733045	0.007437487
mmu-miR-214-3p	n/a	-0.888835974	0.01784931	ENSMUSG00000039515	-0.857712974	0.028928054
mmu-miR-214-3p	n/a	-0.888835974	0.01784931	ENSMUSG00000049680	-0.899071348	0.01476583
mmu-miR-214-3p	n/a	-0.888835974	0.01784931	ENSMUSG00000024213	-0.913755311	0.010836469
mmu-miR-214-3p	n/a	-0.888835974	0.01784931	ENSMUSG00000002871	-0.88079869	0.020466566
mmu-miR-214-3p	n/a	-0.888835974	0.01784931	ENSMUSG00000025147	-0.919553313	0.009447192
mmu-miR-214-3p	n/a	-0.888835974	0.01784931	ENSMUSG00000031765	-0.933301329	0.006524707
mmu-miR-214-3p	n/a	-0.888835974	0.01784931	ENSMUSG00000034485	-0.875585182	0.022255659
mmu-miR-214-5p	ENSMUSG00000024135;	-0.882494063	0.019900228	ENSMUSG00000044881	-0.875523266	0.022277336
mmu-miR-214-5p	ENSMUSG00000024135;	-0.882494063	0.019900228	ENSMUSG00000032340	-0.897502464	0.015220211
mmu-miR-214-5p	ENSMUSG00000024135;	-0.882494063	0.019900228	ENSMUSG00000078735	-0.964792353	0.001837546
mmu-miR-21a-3p	ENSMUSG00000024135;	-0.90149604	0.014076652	ENSMUSG00000029151	-0.892490049	0.016716263
mmu-miR-21a-3p	ENSMUSG00000024135;	-0.90149604	0.014076652	ENSMUSG00000047654	-0.937419421	0.00575195
mmu-miR-21a-3p	ENSMUSG00000024135;	-0.90149604	0.014076652	ENSMUSG00000064247	-0.886907712	0.018461581
mmu-miR-21a-3p	ENSMUSG00000024135;	-0.90149604	0.014076652		-0.850412307	0.031891094
mmu-miR-21a-3p	ENSMUSG00000026103;	-0.878379696	0.021287774	ENSMUSG00000029151	-0.892490049	0.016716263
mmu-miR-21a-3p	ENSMUSG00000026103;	-0.878379696	0.021287774	ENSMUSG00000047654	-0.937419421	0.00575195
mmu-miR-21a-3p	ENSMUSG00000026103;	-0.878379696	0.021287774	ENSMUSG00000064247	-0.886907712	0.018461581
mmu-miR-21a-3p	ENSMUSG00000026103;	-0.878379696	0.021287774		-0.850412307	0.031891094
mmu-miR-21a-3p	ENSMUSG00000062949;	-0.897884224	0.015109035	ENSMUSG00000029151	-0.892490049	0.016716263
mmu-miR-21a-3p	ENSMUSG00000062949;	-0.897884224	0.015109035	ENSMUSG00000047654	-0.937419421	0.00575195
mmu-miR-21a-3p	ENSMUSG00000062949;	-0.897884224	0.015109035	ENSMUSG00000064247	-0.886907712	0.018461581
mmu-miR-21a-3p	ENSMUSG00000062949;	-0.897884224	0.015109035		-0.850412307	0.031891094

mmu-miR-221-3p	ENSMUSG00000040785;	-0.852298906	0.031112325	ENSMUSG00000057897	-0.854394148	0.030258097
mmu-miR-221-3p	ENSMUSG00000040785;	-0.852298906	0.031112325	ENSMUSG00000029507	-0.890100446	0.01745319
mmu-miR-221-3p	ENSMUSG00000040785;	-0.852298906	0.031112325	ENSMUSG00000019873	-0.879368383	0.020950266
mmu-miR-222-5p	ENSMUSG00000040785;	-0.883803372	0.019468061	ENSMUSG00000019846	-0.968214939	0.001499379
mmu-miR-222-5p	ENSMUSG00000040785;	-0.883803372	0.019468061	ENSMUSG00000041189	-0.865598921	0.025881588
mmu-miR-222-5p	ENSMUSG00000040785;	-0.883803372	0.019468061	ENSMUSG00000031543	-0.913238977	0.010964667
mmu-miR-223-3p	ENSMUSG00000058997;	-0.911628923	0.011369106		-0.862229014	0.027163762
mmu-miR-23a-3p	ENSMUSG00000040785;	-0.86853071	0.024790092		-0.877482531	0.021596269
mmu-miR-28a-5p	ENSMUSG00000039852;	-0.913907044	0.010798935		-0.908870637	0.012078446
mmu-miR-28a-5p	ENSMUSG00000046138;	-0.914106509	0.01074969		-0.908870637	0.012078446
mmu-miR-28a-5p	ENSMUSG00000028920;	-0.897999784	0.015075459		-0.908870637	0.012078446
mmu-miR-291b-5p	ENSMUSG00000024566;	-0.859188083	0.028345985		-0.851127962	0.031594609
mmu-miR-299a-3p	ENSMUSG00000037174;	-0.851062975	0.031621478		-0.853417555	0.030654854
mmu-miR-299b-5p	ENSMUSG00000048410;	-0.91010222	0.011759157		-0.886018279	0.018747333
mmu-miR-299b-5p	n/a	-0.930217917	0.007134405		-0.886018279	0.018747333
mmu-miR-29c-3p	ENSMUSG00000024122;	-0.94726959	0.004097436	ENSMUSG00000021368	-0.879466122	0.02091704
mmu-miR-29c-3p	ENSMUSG00000024135;	-0.964483699	0.001869711	ENSMUSG00000021368	-0.879466122	0.02091704
mmu-miR-29c-3p	ENSMUSG00000052812;	-0.964483699	0.001869711	ENSMUSG00000021368	-0.879466122	0.02091704
mmu-miR-29c-3p	ENSMUSG00000016534;	-0.971767352	0.001184372	ENSMUSG00000021368	-0.879466122	0.02091704
mmu-miR-29c-3p	ENSMUSG00000079509;	-0.975524762	0.000891225	ENSMUSG00000021368	-0.879466122	0.02091704
mmu-miR-301b-5p	ENSMUSG00000035139;	-0.873432781	0.023015135	ENSMUSG00000019846	-0.970257068	0.001313807
mmu-miR-301b-5p	ENSMUSG00000035139;	-0.873432781	0.023015135	ENSMUSG00000041653	-0.921872827	0.008917344
mmu-miR-3060-3p	ENSMUSG00000013076;	-0.884100606	0.019370585	ENSMUSG00000058665	-0.996426784	1.91E-05
mmu-miR-3064-3p	ENSMUSG00000048410;	-0.905909925	0.012862926	ENSMUSG00000049807	-0.936184224	0.005978737
mmu-miR-3064-3p	ENSMUSG00000048410;	-0.905909925	0.012862926		-0.875001731	0.022460329
mmu-miR-3072-3p	ENSMUSG00000046079;	-0.86806414	0.024962298	ENSMUSG00000028940	-0.86806414	0.024962298
mmu-miR-3072-3p	ENSMUSG00000046079;	-0.86806414	0.024962298	ENSMUSG00000039329	-0.86806414	0.024962298
mmu-miR-3072-3p	ENSMUSG00000046079;	-0.86806414	0.024962298	ENSMUSG00000041654	-0.886895633	0.018465448
mmu-miR-3072-3p	ENSMUSG00000046079;	-0.86806414	0.024962298	ENSMUSG00000026442	-0.973382986	0.00105327
mmu-miR-3072-3p	ENSMUSG00000046079;	-0.86806414	0.024962298	ENSMUSG00000045591	-0.86806414	0.024962298
mmu-miR-3072-3p	ENSMUSG00000046079;	-0.86806414	0.024962298	ENSMUSG00000049551	-0.892057318	0.01684858
mmu-miR-3072-3p	ENSMUSG00000046079;	-0.86806414	0.024962298	ENSMUSG00000054793	-0.884232002	0.019327569
mmu-miR-3072-3p	ENSMUSG00000046079;	-0.86806414	0.024962298	ENSMUSG00000055239	-0.898950181	0.014800686
mmu-miR-3072-3p	ENSMUSG00000046079;	-0.86806414	0.024962298	ENSMUSG00000053929	-0.86806414	0.024962298
mmu-miR-3072-3p	ENSMUSG00000046079;	-0.86806414	0.024962298	ENSMUSG00000042429	-0.877224031	0.021685549
mmu-miR-3072-3p	ENSMUSG00000046079;	-0.86806414	0.024962298	ENSMUSG00000026883	-0.869614932	0.024392109
mmu-miR-3072-3p	ENSMUSG00000046079;	-0.86806414	0.024962298	ENSMUSG00000019194	-0.907485054	0.012442604
mmu-miR-3072-3p	ENSMUSG00000046079;	-0.86806414	0.024962298	ENSMUSG00000020836	-0.87628593	0.022011024

mmu-miR-3072-3p	ENSMUSG00000046079;	-0.86806414	0.024962298	ENSMUSG00000078786	-0.873360016	0.023041022
mmu-miR-3072-3p	ENSMUSG00000046079;	-0.86806414	0.024962298	ENSMUSG00000027646	-0.891482527	0.01702511
mmu-miR-3072-3p	ENSMUSG00000046079;	-0.86806414	0.024962298	ENSMUSG00000026883	-0.870348609	0.024124538
mmu-miR-3072-3p	ENSMUSG00000046079;	-0.86806414	0.024962298	ENSMUSG00000026883	-0.86806414	0.024962298
mmu-miR-3072-3p	ENSMUSG00000046079;	-0.86806414	0.024962298	ENSMUSG00000024163	-0.85677548	0.029300894
mmu-miR-3072-3p	ENSMUSG00000046079;	-0.86806414	0.024962298	ENSMUSG00000062044	-0.86806414	0.024962298
mmu-miR-3072-3p	ENSMUSG00000046079;	-0.86806414	0.024962298	ENSMUSG00000022753	-0.891826991	0.016919212
mmu-miR-3072-3p	ENSMUSG00000046079;	-0.86806414	0.024962298	ENSMUSG00000024163	-0.86806414	0.024962298
mmu-miR-3072-3p	ENSMUSG00000046079;	-0.86806414	0.024962298	ENSMUSG00000034472	-0.926275	0.007952702
mmu-miR-3072-3p	ENSMUSG00000046079;	-0.86806414	0.024962298	ENSMUSG00000072946	-0.874027674	0.022804011
mmu-miR-3072-3p	ENSMUSG00000046079;	-0.86806414	0.024962298	ENSMUSG00000053297	-0.94822843	0.003951062
mmu-miR-3072-3p	ENSMUSG00000046079;	-0.86806414	0.024962298	ENSMUSG00000021219	-0.86806414	0.024962298
mmu-miR-3072-3p	ENSMUSG00000046079;	-0.86806414	0.024962298	ENSMUSG00000091491	-0.86806414	0.024962298
mmu-miR-3072-3p	ENSMUSG00000046079;	-0.86806414	0.024962298	ENSMUSG00000024163	-0.869879371	0.024295506
mmu-miR-3072-3p	ENSMUSG00000046079;	-0.86806414	0.024962298	ENSMUSG00000023949	-0.86806414	0.024962298
mmu-miR-3072-3p	ENSMUSG00000046079;	-0.86806414	0.024962298	ENSMUSG00000025178	-0.889659391	0.017590874
mmu-miR-3072-3p	ENSMUSG00000046079;	-0.86806414	0.024962298		-0.86806414	0.024962298
mmu-miR-3074-1-3	ENSMUSG00000034724;	-0.894400951	0.016137963	ENSMUSG00000037773	-0.864619415	0.026251233
mmu-miR-3074-2-3	ENSMUSG00000041258;	-0.853945834	0.030439929	ENSMUSG00000029464	-0.911921728	0.011295027
mmu-miR-3074-2-3	ENSMUSG00000002489;	-0.919744667	0.009402919	ENSMUSG00000029464	-0.911921728	0.011295027
mmu-miR-3074-5p	ENSMUSG00000030592;	-0.872477366	0.023356145	ENSMUSG00000053329	-0.903518181	0.01351405
mmu-miR-3074-5p	ENSMUSG00000030592;	-0.872477366	0.023356145	ENSMUSG00000060913	-0.899065568	0.014767492
mmu-miR-3074-5p	ENSMUSG00000030592;	-0.872477366	0.023356145	ENSMUSG00000001604	-0.868109902	0.024945383
mmu-miR-3074-5p	ENSMUSG00000030592;	-0.872477366	0.023356145	ENSMUSG00000031841	-0.866424247	0.025572061
mmu-miR-3074-5p	ENSMUSG00000030592;	-0.872477366	0.023356145	ENSMUSG00000025579	-0.958164455	0.002588709
mmu-miR-3074-5p	ENSMUSG00000030592;	-0.872477366	0.023356145		-0.884790343	0.019145294
mmu-miR-3079-5p	ENSMUSG00000024566;	-0.859188083	0.028345985		-0.851127962	0.031594609
mmu-miR-3084-3p	ENSMUSG00000057406;	-0.856441539	0.029434247	ENSMUSG00000030834	-0.893747239	0.016334696
mmu-miR-3084-3p	ENSMUSG00000057406;	-0.856441539	0.029434247	ENSMUSG00000040860	-0.900956836	0.014228538
mmu-miR-3085-3p	ENSMUSG00000024566;	-0.859188083	0.028345985	ENSMUSG00000103088	-0.929350002	0.007310811
mmu-miR-3085-3p	ENSMUSG00000024566;	-0.859188083	0.028345985	ENSMUSG00000027950	-0.95863831	0.002530803
mmu-miR-3085-3p	ENSMUSG00000024566;	-0.859188083	0.028345985	ENSMUSG00000022263	-0.866995127	0.025358997
mmu-miR-3085-3p	ENSMUSG00000024566;	-0.859188083	0.028345985	ENSMUSG00000070643	-0.870914291	0.023919195
mmu-miR-3085-3p	ENSMUSG00000024566;	-0.859188083	0.028345985	ENSMUSG00000055707	-0.953315287	0.00321832
mmu-miR-3085-3p	ENSMUSG00000024566;	-0.859188083	0.028345985		-0.851127962	0.031594609
mmu-miR-3086-5p	ENSMUSG00000036309;	-0.882264896	0.019976336	ENSMUSG00000033327	-0.853354692	0.030680477
mmu-miR-3089-3p	ENSMUSG00000040913;	-0.871769748	0.023610251	ENSMUSG00000020325	-0.942454549	0.004871938
mmu-miR-3091-3p	ENSMUSG00000027651;	-0.929853538	0.00720821	ENSMUSG00000022565	-0.956036595	0.002856686

mmu-miR-3091-3p	ENSMUSG00000027651;	-0.929853538	0.00720821	ENSMUSG00000012114	-0.953287334	0.003222144
mmu-miR-3098-3p	ENSMUSG00000005312;	-0.893759864	0.016330885	ENSMUSG00000003731	-0.89134542	0.017067349
mmu-miR-3098-3p	ENSMUSG00000005312;	-0.893759864	0.016330885	ENSMUSG00000051969	-0.956664837	0.002776214
mmu-miR-3098-3p	ENSMUSG00000005312;	-0.893759864	0.016330885	ENSMUSG00000053094	-0.863671252	0.026611421
mmu-miR-3098-3p	ENSMUSG00000005312;	-0.893759864	0.016330885	ENSMUSG00000054034	-0.852840394	0.030890484
mmu-miR-3098-3p	ENSMUSG00000005312;	-0.893759864	0.016330885	ENSMUSG00000026858	-0.873488857	0.022995194
mmu-miR-3098-3p	ENSMUSG00000005312;	-0.893759864	0.016330885	ENSMUSG00000000223	-0.924030751	0.008437769
mmu-miR-3098-3p	ENSMUSG00000005312;	-0.893759864	0.016330885	ENSMUSG00000030546	-0.873209544	0.023094599
mmu-miR-30a-5p	ENSMUSG00000024085;	-0.954138466	0.003106691	ENSMUSG00000036529	-0.866268063	0.0256305
mmu-miR-30b-3p	ENSMUSG00000040785;	-0.867665397	0.02510992	ENSMUSG00000025035	-0.927750212	0.007641475
mmu-miR-30b-3p	ENSMUSG00000040785;	-0.867665397	0.02510992		-0.859491714	0.02822687
mmu-miR-30b-3p	ENSMUSG00000024566;	-0.87035218	0.02412324	ENSMUSG00000025035	-0.927750212	0.007641475
mmu-miR-30b-3p	ENSMUSG00000024566;	-0.87035218	0.02412324		-0.859491714	0.02822687
mmu-miR-30c-1-3p	ENSMUSG00000035139;	-0.859346149	0.028283945	ENSMUSG00000024325	-0.91996958	0.00935101
mmu-miR-30c-1-3p	ENSMUSG00000035139;	-0.859346149	0.028283945	ENSMUSG00000028063	-0.8693217	0.024499442
mmu-miR-30c-1-3p	ENSMUSG00000035139;	-0.859346149	0.028283945	ENSMUSG00000024187	-0.953194733	0.003234831
mmu-miR-30c-1-3p	ENSMUSG00000035139;	-0.859346149	0.028283945		-0.860458467	0.027849194
mmu-miR-30d-3p	ENSMUSG00000034801;	-0.898621982	0.014895297		-0.898621982	0.014895297
mmu-miR-30d-3p	ENSMUSG00000025812;	-0.898621982	0.014895297		-0.898621982	0.014895297
mmu-miR-30d-3p	ENSMUSG00000034163;	-0.898621982	0.014895297		-0.898621982	0.014895297
mmu-miR-30d-3p	ENSMUSG00000018800;	-0.898621982	0.014895297		-0.898621982	0.014895297
mmu-miR-30d-3p	ENSMUSG00000034329;	-0.898621982	0.014895297		-0.898621982	0.014895297
mmu-miR-30d-3p	ENSMUSG00000006005;	-0.898621982	0.014895297		-0.898621982	0.014895297
mmu-miR-30d-3p	ENSMUSG00000008763;	-0.898621982	0.014895297		-0.898621982	0.014895297
mmu-miR-30d-3p	ENSMUSG00000030201;	-0.865841142	0.025790563		-0.898621982	0.014895297
mmu-miR-30d-3p	ENSMUSG00000042770;	-0.898621982	0.014895297		-0.898621982	0.014895297
mmu-miR-30d-3p	ENSMUSG00000004567;	-0.898621982	0.014895297		-0.898621982	0.014895297
mmu-miR-30d-3p	ENSMUSG00000032228;	-0.898621982	0.014895297		-0.898621982	0.014895297
mmu-miR-30d-3p	ENSMUSG00000032462;	-0.898621982	0.014895297		-0.898621982	0.014895297
mmu-miR-30e-3p	ENSMUSG00000040785;	-0.887715397	0.018203915		-0.860756469	0.027733262
mmu-miR-30f	ENSMUSG00000040785;	-0.851081877	0.031613661	ENSMUSG00000078896	-0.896284648	0.015577485
mmu-miR-3102-3p	ENSMUSG00000041258;	-0.866122292	0.0256851	ENSMUSG00000000317	-0.906699371	0.01265142
mmu-miR-3102-3p	ENSMUSG00000041258;	-0.866122292	0.0256851	ENSMUSG00000003545	-0.92333036	0.00859201
mmu-miR-3102-3p	ENSMUSG00000041258;	-0.866122292	0.0256851	ENSMUSG00000024319	-0.863849849	0.026543397
mmu-miR-3102-3p	ENSMUSG00000041258;	-0.866122292	0.0256851	ENSMUSG00000031508	-0.949834518	0.003711741
mmu-miR-3102-3p	ENSMUSG00000041258;	-0.866122292	0.0256851	ENSMUSG00000035278	-0.933784822	0.006431516
mmu-miR-3102-3p	ENSMUSG00000041258;	-0.866122292	0.0256851	ENSMUSG00000025422	-0.856960031	0.029227319
mmu-miR-3102-3p	ENSMUSG00000041258;	-0.866122292	0.0256851	ENSMUSG00000042821	-0.983947605	0.000384451

mmu-miR-3102-3p	ENSMUSG00000041258;	-0.866122292	0.0256851	ENSMUSG00000039059	-0.933890704	0.006411195
mmu-miR-3102-3p	ENSMUSG00000041258;	-0.866122292	0.0256851	ENSMUSG00000023033	-0.909517436	0.011910247
mmu-miR-3102-3p	ENSMUSG00000041258;	-0.866122292	0.0256851	ENSMUSG00000026867	-0.925132742	0.00819784
mmu-miR-3102-3p	ENSMUSG00000041258;	-0.866122292	0.0256851	ENSMUSG00000057068	-0.933890704	0.006411195
mmu-miR-3102-3p	ENSMUSG00000041258;	-0.866122292	0.0256851	ENSMUSG00000061046	-0.877813687	0.021482153
mmu-miR-3102-3p	ENSMUSG00000041258;	-0.866122292	0.0256851	ENSMUSG00000044475	-0.951152344	0.003520863
mmu-miR-3102-3p	ENSMUSG00000041258;	-0.866122292	0.0256851	ENSMUSG00000020323	-0.938616994	0.005536169
mmu-miR-3102-3p	ENSMUSG00000041258;	-0.866122292	0.0256851	ENSMUSG00000040563	-0.921264109	0.009054955
mmu-miR-3102-3p	ENSMUSG00000041258;	-0.866122292	0.0256851	ENSMUSG00000032609	-0.920977524	0.009120098
mmu-miR-3102-3p	ENSMUSG00000041258;	-0.866122292	0.0256851	ENSMUSG00000058740	-0.933890704	0.006411195
mmu-miR-3102-3p	ENSMUSG00000041258;	-0.866122292	0.0256851	ENSMUSG00000037905	-0.92655105	0.007894003
mmu-miR-3102-3p	ENSMUSG00000041258;	-0.866122292	0.0256851	ENSMUSG00000030739	-0.932512191	0.006678216
mmu-miR-3102-3p	ENSMUSG00000041258;	-0.866122292	0.0256851	ENSMUSG00000030591	-0.9193085	0.009503981
mmu-miR-3102-3p	ENSMUSG00000041258;	-0.866122292	0.0256851	ENSMUSG00000064030	-0.933045381	0.006574305
mmu-miR-3102-3p	ENSMUSG00000041258;	-0.866122292	0.0256851	ENSMUSG00000054648	-0.934972792	0.006205322
mmu-miR-3102-3p	ENSMUSG00000041258;	-0.866122292	0.0256851	ENSMUSG00000054648	-0.933893258	0.006410705
mmu-miR-3102-3p	ENSMUSG00000026655;	-0.933890704	0.006411195	ENSMUSG00000000317	-0.906699371	0.01265142
mmu-miR-3102-3p	ENSMUSG00000026655;	-0.933890704	0.006411195	ENSMUSG00000003545	-0.92333036	0.00859201
mmu-miR-3102-3p	ENSMUSG00000026655;	-0.933890704	0.006411195	ENSMUSG00000024319	-0.863849849	0.026543397
mmu-miR-3102-3p	ENSMUSG00000026655;	-0.933890704	0.006411195	ENSMUSG00000031508	-0.949834518	0.003711741
mmu-miR-3102-3p	ENSMUSG00000026655;	-0.933890704	0.006411195	ENSMUSG00000035278	-0.933784822	0.006431516
mmu-miR-3102-3p	ENSMUSG00000026655;	-0.933890704	0.006411195	ENSMUSG00000025422	-0.856960031	0.029227319
mmu-miR-3102-3p	ENSMUSG00000026655;	-0.933890704	0.006411195	ENSMUSG00000042821	-0.983947605	0.000384451
mmu-miR-3102-3p	ENSMUSG00000026655;	-0.933890704	0.006411195	ENSMUSG00000039059	-0.933890704	0.006411195
mmu-miR-3102-3p	ENSMUSG00000026655;	-0.933890704	0.006411195	ENSMUSG00000023033	-0.909517436	0.011910247
mmu-miR-3102-3p	ENSMUSG00000026655;	-0.933890704	0.006411195	ENSMUSG00000026867	-0.925132742	0.00819784
mmu-miR-3102-3p	ENSMUSG00000026655;	-0.933890704	0.006411195	ENSMUSG00000057068	-0.933890704	0.006411195
mmu-miR-3102-3p	ENSMUSG00000026655;	-0.933890704	0.006411195	ENSMUSG00000061046	-0.877813687	0.021482153
mmu-miR-3102-3p	ENSMUSG00000026655;	-0.933890704	0.006411195	ENSMUSG00000044475	-0.951152344	0.003520863
mmu-miR-3102-3p	ENSMUSG00000026655;	-0.933890704	0.006411195	ENSMUSG00000020323	-0.938616994	0.005536169
mmu-miR-3102-3p	ENSMUSG00000026655;	-0.933890704	0.006411195	ENSMUSG00000040563	-0.921264109	0.009054955
mmu-miR-3102-3p	ENSMUSG00000026655;	-0.933890704	0.006411195	ENSMUSG00000032609	-0.920977524	0.009120098
mmu-miR-3102-3p	ENSMUSG00000026655;	-0.933890704	0.006411195	ENSMUSG00000058740	-0.933890704	0.006411195
mmu-miR-3102-3p	ENSMUSG00000026655;	-0.933890704	0.006411195	ENSMUSG00000037905	-0.92655105	0.007894003
mmu-miR-3102-3p	ENSMUSG00000026655;	-0.933890704	0.006411195	ENSMUSG00000030739	-0.932512191	0.006678216
mmu-miR-3102-3p	ENSMUSG00000026655;	-0.933890704	0.006411195	ENSMUSG00000030591	-0.9193085	0.009503981
mmu-miR-3102-3p	ENSMUSG00000026655;	-0.933890704	0.006411195	ENSMUSG00000064030	-0.933045381	0.006574305
mmu-miR-3102-3p	ENSMUSG00000026655;	-0.933890704	0.006411195	ENSMUSG00000054648	-0.934972792	0.006205322

mmu-miR-3102-3p	ENSMUSG00000026655;	-0.933890704	0.006411195	ENSMUSG00000054648	-0.933893258	0.006410705
mmu-miR-3102-3p	ENSMUSG00000039477;	-0.933890704	0.006411195	ENSMUSG00000000317	-0.906699371	0.01265142
mmu-miR-3102-3p	ENSMUSG00000039477;	-0.933890704	0.006411195	ENSMUSG00000003545	-0.92333036	0.00859201
mmu-miR-3102-3p	ENSMUSG00000039477;	-0.933890704	0.006411195	ENSMUSG00000024319	-0.863849849	0.026543397
mmu-miR-3102-3p	ENSMUSG00000039477;	-0.933890704	0.006411195	ENSMUSG00000031508	-0.949834518	0.003711741
mmu-miR-3102-3p	ENSMUSG00000039477;	-0.933890704	0.006411195	ENSMUSG00000035278	-0.933784822	0.006431516
mmu-miR-3102-3p	ENSMUSG00000039477;	-0.933890704	0.006411195	ENSMUSG00000025422	-0.856960031	0.029227319
mmu-miR-3102-3p	ENSMUSG00000039477;	-0.933890704	0.006411195	ENSMUSG00000042821	-0.983947605	0.000384451
mmu-miR-3102-3p	ENSMUSG00000039477;	-0.933890704	0.006411195	ENSMUSG00000039059	-0.933890704	0.006411195
mmu-miR-3102-3p	ENSMUSG00000039477;	-0.933890704	0.006411195	ENSMUSG00000023033	-0.909517436	0.011910247
mmu-miR-3102-3p	ENSMUSG00000039477;	-0.933890704	0.006411195	ENSMUSG00000026867	-0.925132742	0.00819784
mmu-miR-3102-3p	ENSMUSG00000039477;	-0.933890704	0.006411195	ENSMUSG00000057068	-0.933890704	0.006411195
mmu-miR-3102-3p	ENSMUSG00000039477;	-0.933890704	0.006411195	ENSMUSG00000061046	-0.877813687	0.021482153
mmu-miR-3102-3p	ENSMUSG00000039477;	-0.933890704	0.006411195	ENSMUSG00000044475	-0.951152344	0.003520863
mmu-miR-3102-3p	ENSMUSG00000039477;	-0.933890704	0.006411195	ENSMUSG00000020323	-0.938616994	0.005536169
mmu-miR-3102-3p	ENSMUSG00000039477;	-0.933890704	0.006411195	ENSMUSG00000040563	-0.921264109	0.009054955
mmu-miR-3102-3p	ENSMUSG00000039477;	-0.933890704	0.006411195	ENSMUSG00000032609	-0.920977524	0.009120098
mmu-miR-3102-3p	ENSMUSG00000039477;	-0.933890704	0.006411195	ENSMUSG00000058740	-0.933890704	0.006411195
mmu-miR-3102-3p	ENSMUSG00000039477;	-0.933890704	0.006411195	ENSMUSG00000037905	-0.92655105	0.007894003
mmu-miR-3102-3p	ENSMUSG00000039477;	-0.933890704	0.006411195	ENSMUSG00000030739	-0.932512191	0.006678216
mmu-miR-3102-3p	ENSMUSG00000039477;	-0.933890704	0.006411195	ENSMUSG00000030591	-0.9193085	0.009503981
mmu-miR-3102-3p	ENSMUSG00000039477;	-0.933890704	0.006411195	ENSMUSG00000064030	-0.933045381	0.006574305
mmu-miR-3102-3p	ENSMUSG00000039477;	-0.933890704	0.006411195	ENSMUSG00000054648	-0.934972792	0.006205322
mmu-miR-3102-3p	ENSMUSG00000039477;	-0.933890704	0.006411195	ENSMUSG00000054648	-0.933893258	0.006410705
mmu-miR-3109-3p	ENSMUSG00000041258;	-0.972621199	0.001114137	ENSMUSG00000002458	-0.856072042	0.029582133
mmu-miR-3109-3p	ENSMUSG00000041258;	-0.972621199	0.001114137	ENSMUSG00000025139	-0.87317993	0.023105151
mmu-miR-3154	ENSMUSG00000024566;	-0.859188083	0.028345985	ENSMUSG00000041096	-0.900732368	0.014291999
mmu-miR-3154	ENSMUSG00000024566;	-0.859188083	0.028345985	ENSMUSG00000018340	-0.857251428	0.029111327
mmu-miR-3154	ENSMUSG00000024566;	-0.859188083	0.028345985		-0.851127962	0.031594609
mmu-miR-320-3p	ENSMUSG00000055447;	-0.869592833	0.02440019	ENSMUSG00000032717	-0.856985634	0.029217119
mmu-miR-320-3p	ENSMUSG00000055447;	-0.869592833	0.02440019	ENSMUSG00000040428	-0.880680483	0.020506336
mmu-miR-320-3p	ENSMUSG00000055447;	-0.869592833	0.02440019	ENSMUSG00000021135	-0.876264608	0.022018449
mmu-miR-322-5p	ENSMUSG00000096370;	-0.905062785	0.013091774	ENSMUSG00000061028	-0.870757006	0.023976206
mmu-miR-324-3p	ENSMUSG00000037174;	-0.859708855	0.028141831	ENSMUSG00000005362	-0.89266019	0.016664376
mmu-miR-324-3p	ENSMUSG00000037174;	-0.859708855	0.028141831	ENSMUSG00000061689	-0.863859167	0.02653985
mmu-miR-324-3p	ENSMUSG00000037174;	-0.859708855	0.028141831	ENSMUSG00000033955	-0.852429985	0.031058555
mmu-miR-324-3p	ENSMUSG00000037174;	-0.859708855	0.028141831	ENSMUSG00000045639	-0.876425418	0.021962482
mmu-miR-324-3p	ENSMUSG00000037174;	-0.859708855	0.028141831	ENSMUSG00000048442	-0.875278741	0.022363045

mmu-miR-324-3p	ENSMUSG00000037174;	-0.859708855	0.028141831	ENSMUSG00000090291	-0.866347747	0.025600677
mmu-miR-324-3p	ENSMUSG00000037174;	-0.859708855	0.028141831	ENSMUSG00000042425	-0.959646698	0.002409728
mmu-miR-324-3p	ENSMUSG00000037174;	-0.859708855	0.028141831	ENSMUSG00000037126	-0.850005328	0.032060282
mmu-miR-324-3p	ENSMUSG00000037174;	-0.859708855	0.028141831	ENSMUSG00000045629	-0.86614656	0.025676006
mmu-miR-324-5p	ENSMUSG00000045038;	-0.903792553	0.013438567	ENSMUSG00000002550	-0.903850154	0.013422747
mmu-miR-324-5p	ENSMUSG00000045038;	-0.903792553	0.013438567	ENSMUSG00000078974	-0.911499291	0.011401978
mmu-miR-324-5p	ENSMUSG00000027651;	-0.852493114	0.031032674	ENSMUSG00000002550	-0.903850154	0.013422747
mmu-miR-324-5p	ENSMUSG00000027651;	-0.852493114	0.031032674	ENSMUSG00000078974	-0.911499291	0.011401978
mmu-miR-326-3p	ENSMUSG00000026082;	-0.896827869	0.015417626	ENSMUSG00000014813	-0.986861806	0.000257784
mmu-miR-326-3p	ENSMUSG00000026082;	-0.896827869	0.015417626	ENSMUSG00000020422	-0.876885164	0.021802852
mmu-miR-326-3p	ENSMUSG00000026082;	-0.896827869	0.015417626	ENSMUSG00000047153	-0.875498357	0.02228606
mmu-miR-326-3p	ENSMUSG00000026082;	-0.896827869	0.015417626	ENSMUSG00000022463	-0.902334624	0.013841997
mmu-miR-326-3p	ENSMUSG00000026082;	-0.896827869	0.015417626	ENSMUSG00000025876	-0.915018924	0.010525817
mmu-miR-326-3p	ENSMUSG00000026082;	-0.896827869	0.015417626	ENSMUSG00000029119	-0.860176743	0.027959005
mmu-miR-326-3p	ENSMUSG00000026082;	-0.896827869	0.015417626	ENSMUSG00000031529	-0.876759937	0.021846278
mmu-miR-326-3p	ENSMUSG00000026082;	-0.896827869	0.015417626	ENSMUSG00000032598	-0.880153925	0.020683943
mmu-miR-326-3p	ENSMUSG00000026082;	-0.896827869	0.015417626	ENSMUSG00000037681	-0.888462992	0.017966968
mmu-miR-326-3p	ENSMUSG00000026082;	-0.896827869	0.015417626	ENSMUSG00000040940	-0.933459895	0.006494072
mmu-miR-326-3p	ENSMUSG00000026082;	-0.896827869	0.015417626	ENSMUSG00000036948	-0.946306173	0.00424714
mmu-miR-326-3p	ENSMUSG00000026082;	-0.896827869	0.015417626	ENSMUSG00000042804	-0.874598995	0.022602127
mmu-miR-326-3p	ENSMUSG00000026082;	-0.896827869	0.015417626	ENSMUSG00000047115	-0.857516091	0.029006166
mmu-miR-326-3p	ENSMUSG00000026082;	-0.896827869	0.015417626	ENSMUSG00000017897	-0.93991707	0.005306489
mmu-miR-326-3p	ENSMUSG00000026082;	-0.896827869	0.015417626	ENSMUSG00000022544	-0.908335455	0.012218483
mmu-miR-326-3p	ENSMUSG00000026082;	-0.896827869	0.015417626	ENSMUSG00000057789	-0.939284008	0.005417735
mmu-miR-326-3p	ENSMUSG00000026082;	-0.896827869	0.015417626	ENSMUSG00000030583	-0.85778399	0.028899903
mmu-miR-326-3p	ENSMUSG00000026082;	-0.896827869	0.015417626	ENSMUSG00000068328	-0.861769836	0.027340745
mmu-miR-326-3p	ENSMUSG00000026082;	-0.896827869	0.015417626	ENSMUSG00000031831	-0.952666038	0.00330773
mmu-miR-326-3p	ENSMUSG00000026082;	-0.896827869	0.015417626	ENSMUSG00000048852	-0.862490261	0.027063311
mmu-miR-326-3p	ENSMUSG00000026082;	-0.896827869	0.015417626	ENSMUSG00000059273	-0.909030249	0.012036834
mmu-miR-326-3p	ENSMUSG00000026082;	-0.896827869	0.015417626	ENSMUSG00000028792	-0.869336788	0.024493914
mmu-miR-326-3p	ENSMUSG00000026082;	-0.896827869	0.015417626	ENSMUSG00000020806	-0.889647534	0.017594582
mmu-miR-326-3p	ENSMUSG00000026082;	-0.896827869	0.015417626	ENSMUSG00000024456	-0.942474049	0.004868669
mmu-miR-326-3p	ENSMUSG00000026082;	-0.896827869	0.015417626	ENSMUSG00000033416	-0.887392039	0.018306864
mmu-miR-326-3p	ENSMUSG00000026082;	-0.896827869	0.015417626	ENSMUSG00000000902	-0.85668027	0.029338885
mmu-miR-326-3p	ENSMUSG00000026082;	-0.896827869	0.015417626	ENSMUSG00000024150	-0.859165341	0.028354916
mmu-miR-326-3p	ENSMUSG00000026082;	-0.896827869	0.015417626	ENSMUSG00000041890	-0.872489212	0.023351902
mmu-miR-326-3p	ENSMUSG00000026082;	-0.896827869	0.015417626	ENSMUSG00000036966	-0.862150516	0.027193979
mmu-miR-326-3p	ENSMUSG00000026082;	-0.896827869	0.015417626	ENSMUSG00000071711	-0.940116891	0.00527161

mmu-miR-326-3p	ENSMUSG00000026082;	-0.896827869	0.015417626	ENSMUSG00000032041	-0.891577915	0.016995753
mmu-miR-326-3p	ENSMUSG00000026082;	-0.896827869	0.015417626	ENSMUSG00000012296	-0.887979791	0.018119947
mmu-miR-326-3p	ENSMUSG00000026082;	-0.896827869	0.015417626	ENSMUSG00000009092	-0.893717758	0.016343595
mmu-miR-326-3p	ENSMUSG00000026082;	-0.896827869	0.015417626	ENSMUSG00000043460	-0.869105031	0.024578896
mmu-miR-326-3p	ENSMUSG00000026082;	-0.896827869	0.015417626		-0.909444308	0.011929207
mmu-miR-326-3p	ENSMUSG00000026082;	-0.896827869	0.015417626		-0.89081295	0.017231864
mmu-miR-326-3p	ENSMUSG00000041852;	-0.953216795	0.003231806	ENSMUSG00000014813	-0.986861806	0.000257784
mmu-miR-326-3p	ENSMUSG00000041852;	-0.953216795	0.003231806	ENSMUSG00000020422	-0.876885164	0.021802852
mmu-miR-326-3p	ENSMUSG00000041852;	-0.953216795	0.003231806	ENSMUSG00000047153	-0.875498357	0.02228606
mmu-miR-326-3p	ENSMUSG00000041852;	-0.953216795	0.003231806	ENSMUSG00000022463	-0.902334624	0.013841997
mmu-miR-326-3p	ENSMUSG00000041852;	-0.953216795	0.003231806	ENSMUSG00000025876	-0.915018924	0.010525817
mmu-miR-326-3p	ENSMUSG00000041852;	-0.953216795	0.003231806	ENSMUSG00000029119	-0.860176743	0.027959005
mmu-miR-326-3p	ENSMUSG00000041852;	-0.953216795	0.003231806	ENSMUSG00000031529	-0.876759937	0.021846278
mmu-miR-326-3p	ENSMUSG00000041852;	-0.953216795	0.003231806	ENSMUSG00000032598	-0.880153925	0.020683943
mmu-miR-326-3p	ENSMUSG00000041852;	-0.953216795	0.003231806	ENSMUSG00000037681	-0.888462992	0.017966968
mmu-miR-326-3p	ENSMUSG00000041852;	-0.953216795	0.003231806	ENSMUSG00000040940	-0.933459895	0.006494072
mmu-miR-326-3p	ENSMUSG00000041852;	-0.953216795	0.003231806	ENSMUSG00000036948	-0.946306173	0.00424714
mmu-miR-326-3p	ENSMUSG00000041852;	-0.953216795	0.003231806	ENSMUSG00000042804	-0.874598995	0.022602127
mmu-miR-326-3p	ENSMUSG00000041852;	-0.953216795	0.003231806	ENSMUSG00000047115	-0.857516091	0.029006166
mmu-miR-326-3p	ENSMUSG00000041852;	-0.953216795	0.003231806	ENSMUSG00000017897	-0.93991707	0.005306489
mmu-miR-326-3p	ENSMUSG00000041852;	-0.953216795	0.003231806	ENSMUSG00000022544	-0.908335455	0.012218483
mmu-miR-326-3p	ENSMUSG00000041852;	-0.953216795	0.003231806	ENSMUSG00000057789	-0.939284008	0.005417735
mmu-miR-326-3p	ENSMUSG00000041852;	-0.953216795	0.003231806	ENSMUSG00000030583	-0.85778399	0.028899903
mmu-miR-326-3p	ENSMUSG00000041852;	-0.953216795	0.003231806	ENSMUSG00000068328	-0.861769836	0.027340745
mmu-miR-326-3p	ENSMUSG00000041852;	-0.953216795	0.003231806	ENSMUSG00000031831	-0.952666038	0.00330773
mmu-miR-326-3p	ENSMUSG00000041852;	-0.953216795	0.003231806	ENSMUSG00000048852	-0.862490261	0.027063311
mmu-miR-326-3p	ENSMUSG00000041852;	-0.953216795	0.003231806	ENSMUSG00000059273	-0.909030249	0.012036834
mmu-miR-326-3p	ENSMUSG00000041852;	-0.953216795	0.003231806	ENSMUSG00000028792	-0.869336788	0.024493914
mmu-miR-326-3p	ENSMUSG00000041852;	-0.953216795	0.003231806	ENSMUSG00000020806	-0.889647534	0.017594582
mmu-miR-326-3p	ENSMUSG00000041852;	-0.953216795	0.003231806	ENSMUSG00000024456	-0.942474049	0.004868669
mmu-miR-326-3p	ENSMUSG00000041852;	-0.953216795	0.003231806	ENSMUSG00000033416	-0.887392039	0.018306864
mmu-miR-326-3p	ENSMUSG00000041852;	-0.953216795	0.003231806	ENSMUSG00000000902	-0.85668027	0.029338885
mmu-miR-326-3p	ENSMUSG00000041852;	-0.953216795	0.003231806	ENSMUSG00000024150	-0.859165341	0.028354916
mmu-miR-326-3p	ENSMUSG00000041852;	-0.953216795	0.003231806	ENSMUSG00000041890	-0.872489212	0.023351902
mmu-miR-326-3p	ENSMUSG00000041852;	-0.953216795	0.003231806	ENSMUSG00000036966	-0.862150516	0.027193979
mmu-miR-326-3p	ENSMUSG00000041852;	-0.953216795	0.003231806	ENSMUSG00000071711	-0.940116891	0.00527161
mmu-miR-326-3p	ENSMUSG00000041852;	-0.953216795	0.003231806	ENSMUSG00000032041	-0.891577915	0.016995753
mmu-miR-326-3p	ENSMUSG00000041852;	-0.953216795	0.003231806	ENSMUSG00000012296	-0.887979791	0.018119947

mmu-miR-326-3p	ENSMUSG00000041852;	-0.953216795	0.003231806	ENSMUSG00000009092	-0.893717758	0.016343595
mmu-miR-326-3p	ENSMUSG00000041852;	-0.953216795	0.003231806	ENSMUSG00000043460	-0.869105031	0.024578896
mmu-miR-326-3p	ENSMUSG00000041852;	-0.953216795	0.003231806		-0.909444308	0.011929207
mmu-miR-326-3p	ENSMUSG00000041852;	-0.953216795	0.003231806		-0.89081295	0.017231864
mmu-miR-328-5p	ENSMUSG00000041297;	-0.852171712	0.031164543	ENSMUSG00000005899	-0.955512442	0.002924691
mmu-miR-328-5p	ENSMUSG00000041297;	-0.852171712	0.031164543	ENSMUSG00000017446	-0.921030608	0.009108014
mmu-miR-328-5p	ENSMUSG00000041297;	-0.852171712	0.031164543	ENSMUSG00000022020	-0.924434042	0.008349572
mmu-miR-328-5p	ENSMUSG00000041297;	-0.852171712	0.031164543	ENSMUSG00000026668	-0.882314857	0.019959732
mmu-miR-328-5p	ENSMUSG00000041297;	-0.852171712	0.031164543	ENSMUSG00000026466	-0.971884132	0.00117464
mmu-miR-328-5p	ENSMUSG00000041297;	-0.852171712	0.031164543	ENSMUSG00000016179	-0.853584856	0.030586712
mmu-miR-328-5p	ENSMUSG00000041297;	-0.852171712	0.031164543	ENSMUSG00000036123	-0.92009531	0.009322053
mmu-miR-329-5p	ENSMUSG00000035234;	-0.875899268	0.02214585	ENSMUSG00000023908	-0.873649468	0.022938127
mmu-miR-329-5p	ENSMUSG00000035234;	-0.875899268	0.02214585	ENSMUSG00000046694	-0.903688701	0.013467114
mmu-miR-329-5p	ENSMUSG00000035234;	-0.875899268	0.02214585	ENSMUSG00000028700	-0.868759261	0.024705943
mmu-miR-329-5p	ENSMUSG00000035234;	-0.875899268	0.02214585	ENSMUSG00000110234	-0.886344764	0.018642198
mmu-miR-329-5p	ENSMUSG00000035234;	-0.875899268	0.02214585		-0.868759261	0.024705943
mmu-miR-329-5p	ENSMUSG00000044471;	-0.865353868	0.025973832	ENSMUSG00000023908	-0.873649468	0.022938127
mmu-miR-329-5p	ENSMUSG00000044471;	-0.865353868	0.025973832	ENSMUSG00000046694	-0.903688701	0.013467114
mmu-miR-329-5p	ENSMUSG00000044471;	-0.865353868	0.025973832	ENSMUSG00000028700	-0.868759261	0.024705943
mmu-miR-329-5p	ENSMUSG00000044471;	-0.865353868	0.025973832	ENSMUSG00000110234	-0.886344764	0.018642198
mmu-miR-329-5p	ENSMUSG00000044471;	-0.865353868	0.025973832		-0.868759261	0.024705943
mmu-miR-329-5p	ENSMUSG00000022329;	-0.868759261	0.024705943	ENSMUSG00000023908	-0.873649468	0.022938127
mmu-miR-329-5p	ENSMUSG00000022329;	-0.868759261	0.024705943	ENSMUSG00000046694	-0.903688701	0.013467114
mmu-miR-329-5p	ENSMUSG00000022329;	-0.868759261	0.024705943	ENSMUSG00000028700	-0.868759261	0.024705943
mmu-miR-329-5p	ENSMUSG00000022329;	-0.868759261	0.024705943	ENSMUSG00000110234	-0.886344764	0.018642198
mmu-miR-329-5p	ENSMUSG00000022329;	-0.868759261	0.024705943		-0.868759261	0.024705943
mmu-miR-329-5p	ENSMUSG00000024376;	-0.868759261	0.024705943	ENSMUSG00000023908	-0.873649468	0.022938127
mmu-miR-329-5p	ENSMUSG00000024376;	-0.868759261	0.024705943	ENSMUSG00000046694	-0.903688701	0.013467114
mmu-miR-329-5p	ENSMUSG00000024376;	-0.868759261	0.024705943	ENSMUSG00000028700	-0.868759261	0.024705943
mmu-miR-329-5p	ENSMUSG00000024376;	-0.868759261	0.024705943	ENSMUSG00000110234	-0.886344764	0.018642198
mmu-miR-329-5p	ENSMUSG00000024376;	-0.868759261	0.024705943		-0.868759261	0.024705943
mmu-miR-329-5p	ENSMUSG00000066894;	-0.868759261	0.024705943	ENSMUSG00000023908	-0.873649468	0.022938127
mmu-miR-329-5p	ENSMUSG00000066894;	-0.868759261	0.024705943	ENSMUSG00000046694	-0.903688701	0.013467114
mmu-miR-329-5p	ENSMUSG00000066894;	-0.868759261	0.024705943	ENSMUSG00000028700	-0.868759261	0.024705943
mmu-miR-329-5p	ENSMUSG00000066894;	-0.868759261	0.024705943	ENSMUSG00000110234	-0.886344764	0.018642198
mmu-miR-329-5p	ENSMUSG00000066894;	-0.868759261	0.024705943		-0.868759261	0.024705943
mmu-miR-329-5p	ENSMUSG00000030516;	-0.868759261	0.024705943	ENSMUSG00000023908	-0.873649468	0.022938127
mmu-miR-329-5p	ENSMUSG00000030516;	-0.868759261	0.024705943	ENSMUSG00000046694	-0.903688701	0.013467114

mmu-miR-329-5p	ENSMUSG00000030516;	-0.868759261	0.024705943	ENSMUSG00000028700	-0.868759261	0.024705943
mmu-miR-329-5p	ENSMUSG00000030516;	-0.868759261	0.024705943	ENSMUSG00000110234	-0.886344764	0.018642198
mmu-miR-329-5p	ENSMUSG00000030516;	-0.868759261	0.024705943		-0.868759261	0.024705943
mmu-miR-330-3p	ENSMUSG00000040785;	-0.851466229	0.031454925	ENSMUSG00000017309	-0.960605819	0.002297284
mmu-miR-330-3p	ENSMUSG00000040785;	-0.851466229	0.031454925	ENSMUSG00000039095	-0.879693214	0.020839941
mmu-miR-330-3p	ENSMUSG00000040785;	-0.851466229	0.031454925	ENSMUSG00000033717	-0.926255829	0.007956786
mmu-miR-330-3p	ENSMUSG00000040785;	-0.851466229	0.031454925	ENSMUSG00000043122	-0.870488551	0.024073661
mmu-miR-330-3p	ENSMUSG00000040785;	-0.851466229	0.031454925	ENSMUSG00000039376	-0.956953841	0.002739576
mmu-miR-330-3p	ENSMUSG00000040785;	-0.851466229	0.031454925	ENSMUSG00000078570	-0.921755934	0.00894369
mmu-miR-330-3p	ENSMUSG00000040785;	-0.851466229	0.031454925	ENSMUSG00000070643	-0.909996284	0.011786458
mmu-miR-330-3p	ENSMUSG00000040785;	-0.851466229	0.031454925	ENSMUSG00000067653	-0.880057516	0.020716541
mmu-miR-330-3p	ENSMUSG00000040785;	-0.851466229	0.031454925	ENSMUSG00000066235	-0.892284802	0.01677896
mmu-miR-330-5p	ENSMUSG00000022801;	-0.946698271	0.004185894	ENSMUSG00000048583	-0.871694419	0.023637379
mmu-miR-330-5p	ENSMUSG00000022801;	-0.946698271	0.004185894	ENSMUSG00000027895	-0.853383993	0.030668533
mmu-miR-330-5p	ENSMUSG00000022801;	-0.946698271	0.004185894	ENSMUSG00000041020	-0.872908654	0.023201912
mmu-miR-330-5p	ENSMUSG00000022801;	-0.946698271	0.004185894	ENSMUSG00000041653	-0.919324852	0.009500183
mmu-miR-330-5p	ENSMUSG00000022801;	-0.946698271	0.004185894	ENSMUSG00000049699	-0.910850624	0.011567154
mmu-miR-330-5p	ENSMUSG00000022801;	-0.946698271	0.004185894	ENSMUSG00000045733	-0.949215377	0.003803128
mmu-miR-330-5p	ENSMUSG00000022801;	-0.946698271	0.004185894	ENSMUSG00000061048	-0.906232714	0.012776241
mmu-miR-330-5p	ENSMUSG00000022801;	-0.946698271	0.004185894	ENSMUSG00000035407	-0.865850637	0.025786998
mmu-miR-330-5p	ENSMUSG00000022801;	-0.946698271	0.004185894	ENSMUSG00000053477	-0.857507726	0.029009487
mmu-miR-330-5p	ENSMUSG00000022801;	-0.946698271	0.004185894	ENSMUSG00000025020	-0.984799533	0.000344825
mmu-miR-330-5p	ENSMUSG00000022801;	-0.946698271	0.004185894	ENSMUSG00000090486	-0.887713139	0.018204633
mmu-miR-330-5p	ENSMUSG00000022801;	-0.946698271	0.004185894	ENSMUSG00000053477	-0.870123871	0.02420635
mmu-miR-330-5p	ENSMUSG00000022801;	-0.946698271	0.004185894	ENSMUSG00000009281	-0.8974886	0.015224256
mmu-miR-330-5p	ENSMUSG00000022801;	-0.946698271	0.004185894	ENSMUSG00000062044	-0.859020158	0.028411964
mmu-miR-330-5p	ENSMUSG00000022801;	-0.946698271	0.004185894	ENSMUSG00000004791	-0.875577943	0.022258193
mmu-miR-330-5p	ENSMUSG00000022801;	-0.946698271	0.004185894	ENSMUSG00000023993	-0.874198082	0.022743706
mmu-miR-330-5p	ENSMUSG00000038538;	-0.963926992	0.001928423	ENSMUSG00000048583	-0.871694419	0.023637379
mmu-miR-330-5p	ENSMUSG00000038538;	-0.963926992	0.001928423	ENSMUSG00000027895	-0.853383993	0.030668533
mmu-miR-330-5p	ENSMUSG00000038538;	-0.963926992	0.001928423	ENSMUSG00000041020	-0.872908654	0.023201912
mmu-miR-330-5p	ENSMUSG00000038538;	-0.963926992	0.001928423	ENSMUSG00000041653	-0.919324852	0.009500183
mmu-miR-330-5p	ENSMUSG00000038538;	-0.963926992	0.001928423	ENSMUSG00000049699	-0.910850624	0.011567154
mmu-miR-330-5p	ENSMUSG00000038538;	-0.963926992	0.001928423	ENSMUSG00000045733	-0.949215377	0.003803128
mmu-miR-330-5p	ENSMUSG00000038538;	-0.963926992	0.001928423	ENSMUSG00000061048	-0.906232714	0.012776241
mmu-miR-330-5p	ENSMUSG00000038538;	-0.963926992	0.001928423	ENSMUSG00000035407	-0.865850637	0.025786998
mmu-miR-330-5p	ENSMUSG00000038538;	-0.963926992	0.001928423	ENSMUSG00000053477	-0.857507726	0.029009487
mmu-miR-330-5p	ENSMUSG00000038538;	-0.963926992	0.001928423	ENSMUSG00000025020	-0.984799533	0.000344825

mmu-miR-330-5p	ENSMUSG00000038538;	-0.963926992	0.001928423	ENSMUSG00000090486	-0.887713139	0.018204633
mmu-miR-330-5p	ENSMUSG00000038538;	-0.963926992	0.001928423	ENSMUSG00000053477	-0.870123871	0.02420635
mmu-miR-330-5p	ENSMUSG00000038538;	-0.963926992	0.001928423	ENSMUSG00000009281	-0.8974886	0.015224256
mmu-miR-330-5p	ENSMUSG00000038538;	-0.963926992	0.001928423	ENSMUSG00000062044	-0.859020158	0.028411964
mmu-miR-330-5p	ENSMUSG00000038538;	-0.963926992	0.001928423	ENSMUSG00000004791	-0.875577943	0.022258193
mmu-miR-330-5p	ENSMUSG00000038538;	-0.963926992	0.001928423	ENSMUSG00000023993	-0.874198082	0.022743706
mmu-miR-337-3p	ENSMUSG00000035845;	-0.872459983	0.023362372	ENSMUSG00000024181	-0.890647214	0.017283226
mmu-miR-337-3p	ENSMUSG00000035845;	-0.872459983	0.023362372	ENSMUSG00000049792	-0.985267923	0.000323952
mmu-miR-338-5p	ENSMUSG00000033102;	-0.860712573	0.027750324		-0.89103904	0.017161917
mmu-miR-339-3p	ENSMUSG00000025453;E	-0.871132085	0.023840359	ENSMUSG00000030116	-0.871132085	0.023840359
mmu-miR-339-3p	ENSMUSG00000025453;E	-0.871132085	0.023840359	ENSMUSG00000030877	-0.871132085	0.023840359
mmu-miR-339-3p	ENSMUSG00000030067;E	-0.974448438	0.000970982	ENSMUSG00000030116	-0.871132085	0.023840359
mmu-miR-339-3p	ENSMUSG00000030067;E	-0.974448438	0.000970982	ENSMUSG00000030877	-0.871132085	0.023840359
mmu-miR-339-3p	ENSMUSG00000017499;	-0.872647025	0.023295415	ENSMUSG00000030116	-0.871132085	0.023840359
mmu-miR-339-3p	ENSMUSG00000017499;	-0.872647025	0.023295415	ENSMUSG00000030877	-0.871132085	0.023840359
mmu-miR-3470b	ENSMUSG00000028053;	-0.862765237	0.026957773	ENSMUSG00000010755	-0.984830582	0.000343422
mmu-miR-3470b	ENSMUSG00000028053;	-0.862765237	0.026957773	ENSMUSG00000009079	-0.873667999	0.022931547
mmu-miR-3470b	ENSMUSG00000028053;	-0.862765237	0.026957773	ENSMUSG00000037355	-0.862462849	0.027073843
mmu-miR-3470b	ENSMUSG00000028053;	-0.862765237	0.026957773	ENSMUSG00000036473	-0.932192019	0.006740996
mmu-miR-3470b	ENSMUSG00000028053;	-0.862765237	0.026957773	ENSMUSG00000040860	-0.867855792	0.02503938
mmu-miR-3470b	ENSMUSG00000028053;	-0.862765237	0.026957773	ENSMUSG00000037754	-0.911092497	0.011505429
mmu-miR-3470b	ENSMUSG00000028053;	-0.862765237	0.026957773	ENSMUSG00000079477	-0.895010042	0.01595569
mmu-miR-3470b	ENSMUSG00000028053;	-0.862765237	0.026957773	ENSMUSG00000020109	-0.907958991	0.012317456
mmu-miR-3470b	ENSMUSG00000028053;	-0.862765237	0.026957773	ENSMUSG00000066037	-0.947402347	0.004077014
mmu-miR-3470b	ENSMUSG00000028053;	-0.862765237	0.026957773	ENSMUSG00000040913	-0.961805445	0.002160376
mmu-miR-3470b	ENSMUSG00000028053;	-0.862765237	0.026957773	ENSMUSG00000037280	-0.861662478	0.027382204
mmu-miR-3470b	ENSMUSG00000028053;	-0.862765237	0.026957773	ENSMUSG00000043467	-0.865937751	0.0257543
mmu-miR-3470b	ENSMUSG00000028053;	-0.862765237	0.026957773	ENSMUSG00000026594	-0.851270664	0.031535646
mmu-miR-3470b	ENSMUSG00000028053;	-0.862765237	0.026957773	ENSMUSG00000005813	-0.928863195	0.007410676
mmu-miR-3470b	ENSMUSG00000028053;	-0.862765237	0.026957773	ENSMUSG00000107283	-0.867335465	0.025232378
mmu-miR-3470b	ENSMUSG00000028053;	-0.862765237	0.026957773	ENSMUSG00000024082	-0.875546882	0.022269067
mmu-miR-3470b	ENSMUSG00000028053;	-0.862765237	0.026957773	ENSMUSG00000024442	-0.851369983	0.031494639
mmu-miR-3474	ENSMUSG00000040785;	-0.87096828	0.023899641	ENSMUSG00000002233	-0.864080931	0.026455506
mmu-miR-3474	ENSMUSG00000040785;	-0.87096828	0.023899641	ENSMUSG00000020894	-0.881105831	0.020363402
mmu-miR-3474	ENSMUSG00000040785;	-0.87096828	0.023899641	ENSMUSG00000021765	-0.876682802	0.021873046
mmu-miR-3474	ENSMUSG00000040785;	-0.87096828	0.023899641	ENSMUSG00000036636	-0.927854954	0.007619607
mmu-miR-3474	ENSMUSG00000040785;	-0.87096828	0.023899641	ENSMUSG00000029847	-0.952889716	0.00327679
mmu-miR-3474	ENSMUSG00000040785;	-0.87096828	0.023899641	ENSMUSG00000031340	-0.873363344	0.023039838

mmu-miR-3474	ENSMUSG00000040785;	-0.87096828	0.023899641	ENSMUSG00000032182	-0.933725278	0.006442958
mmu-miR-3474	ENSMUSG00000040785;	-0.87096828	0.023899641	ENSMUSG00000033768	-0.865487792	0.025923401
mmu-miR-3474	ENSMUSG00000040785;	-0.87096828	0.023899641	ENSMUSG00000056596	-0.851184635	0.031571186
mmu-miR-3474	ENSMUSG00000040785;	-0.87096828	0.023899641	ENSMUSG00000091712	-0.851270306	0.031535794
mmu-miR-3474	ENSMUSG00000040785;	-0.87096828	0.023899641	ENSMUSG00000037649	-0.967439574	0.001573012
mmu-miR-3475-3p	ENSMUSG00000024566;	-0.906790588	0.01262709	ENSMUSG00000061577	-0.900148102	0.014457821
mmu-miR-3475-3p	ENSMUSG00000024566;	-0.906790588	0.01262709		-0.855634324	0.029757778
mmu-miR-34b-3p	ENSMUSG00000064351;	-0.886984891	0.018436884		-0.85601344	0.02960562
mmu-miR-350-5p	ENSMUSG00000024566;	-0.990022608	0.000148826	ENSMUSG00000057110	-0.864831035	0.026171161
mmu-miR-350-5p	ENSMUSG00000024566;	-0.990022608	0.000148826	ENSMUSG00000035401	-0.867970431	0.024996954
mmu-miR-350-5p	ENSMUSG00000024566;	-0.990022608	0.000148826		-0.932314561	0.006716934
mmu-miR-3572-3p	ENSMUSG00000020640;	-0.941871392	0.004970196	ENSMUSG00000052151	-0.902358965	0.013835214
mmu-miR-3572-3p	ENSMUSG00000020640;	-0.941871392	0.004970196	ENSMUSG00000022313	-0.886103257	0.018719941
mmu-miR-3572-5p	ENSMUSG00000040785;	-0.862178118	0.027183352	ENSMUSG00000022074	-0.868469611	0.024812611
mmu-miR-3572-5p	ENSMUSG00000040785;	-0.862178118	0.027183352	ENSMUSG00000022621	-0.965622354	0.00175242
mmu-miR-3572-5p	ENSMUSG00000040785;	-0.862178118	0.027183352	ENSMUSG00000039262	-0.861785489	0.027334703
mmu-miR-3572-5p	ENSMUSG00000040785;	-0.862178118	0.027183352	ENSMUSG00000041782	-0.964643755	0.001852997
mmu-miR-3572-5p	ENSMUSG00000040785;	-0.862178118	0.027183352	ENSMUSG00000034353	-0.93513653	0.006174456
mmu-miR-3572-5p	ENSMUSG00000040785;	-0.862178118	0.027183352	ENSMUSG00000033272	-0.855436603	0.029837279
mmu-miR-370-3p	ENSMUSG00000032355;	-0.875144363	0.022410212	ENSMUSG00000000531	-0.87754526	0.021574631
mmu-miR-370-3p	ENSMUSG00000032355;	-0.875144363	0.022410212	ENSMUSG00000000690	-0.871118278	0.023845353
mmu-miR-370-3p	ENSMUSG00000032355;	-0.875144363	0.022410212	ENSMUSG00000053329	-0.93594941	0.006022334
mmu-miR-370-3p	ENSMUSG00000032355;	-0.875144363	0.022410212	ENSMUSG00000005683	-0.93731554	0.005770858
mmu-miR-370-3p	ENSMUSG00000032355;	-0.875144363	0.022410212	ENSMUSG00000006463	-0.936458638	0.005927983
mmu-miR-370-3p	ENSMUSG00000032355;	-0.875144363	0.022410212	ENSMUSG00000009281	-0.960742175	0.002281514
mmu-miR-370-3p	ENSMUSG00000032355;	-0.875144363	0.022410212	ENSMUSG00000027895	-0.878546581	0.021230624
mmu-miR-370-3p	ENSMUSG00000032355;	-0.875144363	0.022410212	ENSMUSG00000018428	-0.888887648	0.017833038
mmu-miR-370-3p	ENSMUSG00000032355;	-0.875144363	0.022410212	ENSMUSG00000018566	-0.960102255	0.00235599
mmu-miR-370-3p	ENSMUSG00000032355;	-0.875144363	0.022410212	ENSMUSG00000020393	-0.955689668	0.002901609
mmu-miR-370-3p	ENSMUSG00000032355;	-0.875144363	0.022410212	ENSMUSG00000020722	-0.857348502	0.029072735
mmu-miR-370-3p	ENSMUSG00000032355;	-0.875144363	0.022410212	ENSMUSG00000020810	-0.945385153	0.00439272
mmu-miR-370-3p	ENSMUSG00000032355;	-0.875144363	0.022410212	ENSMUSG00000021062	-0.867298935	0.025245954
mmu-miR-370-3p	ENSMUSG00000032355;	-0.875144363	0.022410212	ENSMUSG00000021376	-0.877087128	0.021732904
mmu-miR-370-3p	ENSMUSG00000032355;	-0.875144363	0.022410212	ENSMUSG00000058975	-0.9295164	0.007276828
mmu-miR-370-3p	ENSMUSG00000032355;	-0.875144363	0.022410212	ENSMUSG00000025781	-0.889194836	0.017736456
mmu-miR-370-3p	ENSMUSG00000032355;	-0.875144363	0.022410212	ENSMUSG00000025813	-0.874246157	0.022726706
mmu-miR-370-3p	ENSMUSG00000032355;	-0.875144363	0.022410212	ENSMUSG00000025880	-0.959318774	0.00244878
mmu-miR-370-3p	ENSMUSG00000032355;	-0.875144363	0.022410212	ENSMUSG00000026478	-0.945819755	0.004323725

mmu-miR-370-3p	ENSMUSG00000032355;	-0.875144363	0.022410212	ENSMUSG00000026489	-0.910317543	0.011703759
mmu-miR-370-3p	ENSMUSG00000032355;	-0.875144363	0.022410212	ENSMUSG00000026853	-0.853111377	0.030779747
mmu-miR-370-3p	ENSMUSG00000032355;	-0.875144363	0.022410212	ENSMUSG00000028150	-0.917410261	0.009949923
mmu-miR-370-3p	ENSMUSG00000032355;	-0.875144363	0.022410212	ENSMUSG00000029312	-0.966154221	0.001698919
mmu-miR-370-3p	ENSMUSG00000032355;	-0.875144363	0.022410212	ENSMUSG00000029499	-0.852296016	0.031113511
mmu-miR-370-3p	ENSMUSG00000032355;	-0.875144363	0.022410212	ENSMUSG00000030189	-0.903150027	0.013615653
mmu-miR-370-3p	ENSMUSG00000032355;	-0.875144363	0.022410212	ENSMUSG00000030516	-0.897361635	0.015261322
mmu-miR-370-3p	ENSMUSG00000032355;	-0.875144363	0.022410212	ENSMUSG00000031312	-0.898617002	0.014896735
mmu-miR-370-3p	ENSMUSG00000032355;	-0.875144363	0.022410212	ENSMUSG00000031782	-0.895139692	0.015917021
mmu-miR-370-3p	ENSMUSG00000032355;	-0.875144363	0.022410212	ENSMUSG00000031790	-0.874572058	0.022611626
mmu-miR-370-3p	ENSMUSG00000032355;	-0.875144363	0.022410212	ENSMUSG00000031840	-0.945975121	0.00429919
mmu-miR-370-3p	ENSMUSG00000032355;	-0.875144363	0.022410212	ENSMUSG00000032047	-0.936936623	0.005840083
mmu-miR-370-3p	ENSMUSG00000032355;	-0.875144363	0.022410212	ENSMUSG00000032194	-0.926196019	0.007969535
mmu-miR-370-3p	ENSMUSG00000032355;	-0.875144363	0.022410212	ENSMUSG00000032549	-0.861976012	0.027261211
mmu-miR-370-3p	ENSMUSG00000032355;	-0.875144363	0.022410212	ENSMUSG00000039496	-0.941431321	0.005044981
mmu-miR-370-3p	ENSMUSG00000032355;	-0.875144363	0.022410212	ENSMUSG00000036975	-0.86752023	0.025163765
mmu-miR-370-3p	ENSMUSG00000032355;	-0.875144363	0.022410212	ENSMUSG00000064080	-0.981400387	0.000515701
mmu-miR-370-3p	ENSMUSG00000032355;	-0.875144363	0.022410212	ENSMUSG00000035923	-0.885018999	0.019070885
mmu-miR-370-3p	ENSMUSG00000032355;	-0.875144363	0.022410212	ENSMUSG00000041731	-0.92629233	0.007949011
mmu-miR-370-3p	ENSMUSG00000032355;	-0.875144363	0.022410212	ENSMUSG00000030554	-0.940854495	0.005143835
mmu-miR-370-3p	ENSMUSG00000032355;	-0.875144363	0.022410212	ENSMUSG00000021411	-0.870260733	0.024156512
mmu-miR-370-3p	ENSMUSG00000032355;	-0.875144363	0.022410212	ENSMUSG00000050234	-0.890342741	0.017377773
mmu-miR-370-3p	ENSMUSG00000032355;	-0.875144363	0.022410212	ENSMUSG00000067653	-0.850532489	0.031841213
mmu-miR-370-3p	ENSMUSG00000032355;	-0.875144363	0.022410212	ENSMUSG00000043795	-0.88827232	0.018027259
mmu-miR-370-3p	ENSMUSG00000032355;	-0.875144363	0.022410212	ENSMUSG00000051652	-0.877848527	0.021470163
mmu-miR-370-3p	ENSMUSG00000032355;	-0.875144363	0.022410212	ENSMUSG00000044938	-0.926505861	0.007903597
mmu-miR-370-3p	ENSMUSG00000032355;	-0.875144363	0.022410212	ENSMUSG00000048096	-0.875688043	0.022219669
mmu-miR-370-3p	ENSMUSG00000032355;	-0.875144363	0.022410212	ENSMUSG00000028458	-0.891300635	0.017081156
mmu-miR-370-3p	ENSMUSG00000032355;	-0.875144363	0.022410212	ENSMUSG00000035437	-0.899203775	0.01472778
mmu-miR-370-3p	ENSMUSG00000032355;	-0.875144363	0.022410212	ENSMUSG00000051984	-0.853094515	0.030786633
mmu-miR-370-3p	ENSMUSG00000032355;	-0.875144363	0.022410212	ENSMUSG00000049265	-0.939213962	0.005430113
mmu-miR-370-3p	ENSMUSG00000032355;	-0.875144363	0.022410212	ENSMUSG00000002781	-0.864264441	0.026385807
mmu-miR-370-3p	ENSMUSG00000032355;	-0.875144363	0.022410212	ENSMUSG00000038528	-0.917815812	0.009853815
mmu-miR-370-3p	ENSMUSG00000032355;	-0.875144363	0.022410212	ENSMUSG00000063821	-0.88557915	0.01888919
mmu-miR-370-3p	ENSMUSG00000032355;	-0.875144363	0.022410212	ENSMUSG00000030554	-0.881825137	0.020122774
mmu-miR-370-3p	ENSMUSG00000032355;	-0.875144363	0.022410212	ENSMUSG00000022197	-0.95521296	0.0029639
mmu-miR-370-3p	ENSMUSG00000032355;	-0.875144363	0.022410212	ENSMUSG00000071856	-0.945175807	0.004426146
mmu-miR-370-3p	ENSMUSG00000032355;	-0.875144363	0.022410212	ENSMUSG00000004892	-0.933583949	0.006470154

mmu-miR-370-3p	ENSMUSG00000032355;	-0.875144363	0.022410212	ENSMUSG00000043822	-0.97343196	0.001049414
mmu-miR-370-3p	ENSMUSG00000032355;	-0.875144363	0.022410212	ENSMUSG00000026858	-0.935116115	0.0061783
mmu-miR-370-3p	ENSMUSG00000032355;	-0.875144363	0.022410212	ENSMUSG00000072812	-0.960579228	0.002300366
mmu-miR-370-3p	ENSMUSG00000032355;	-0.875144363	0.022410212	ENSMUSG00000001604	-0.96840312	0.001481772
mmu-miR-370-3p	ENSMUSG00000032355;	-0.875144363	0.022410212	ENSMUSG00000042826	-0.877048193	0.02174638
mmu-miR-370-3p	ENSMUSG00000032355;	-0.875144363	0.022410212	ENSMUSG00000020937	-0.877673937	0.021530275
mmu-miR-370-3p	ENSMUSG00000032355;	-0.875144363	0.022410212	ENSMUSG00000017167	-0.86863291	0.024752447
mmu-miR-370-3p	ENSMUSG00000032355;	-0.875144363	0.022410212	ENSMUSG00000041351	-0.86406303	0.02646231
mmu-miR-370-3p	ENSMUSG00000032355;	-0.875144363	0.022410212	ENSMUSG00000037139	-0.884942603	0.01909573
mmu-miR-370-3p	ENSMUSG00000032355;	-0.875144363	0.022410212	ENSMUSG00000042828	-0.899060294	0.014769008
mmu-miR-370-3p	ENSMUSG00000032355;	-0.875144363	0.022410212	ENSMUSG00000038763	-0.882308915	0.019961706
mmu-miR-370-3p	ENSMUSG00000032355;	-0.875144363	0.022410212	ENSMUSG00000028433	-0.902897321	0.013685608
mmu-miR-370-3p	ENSMUSG00000032355;	-0.875144363	0.022410212	ENSMUSG00000078937	-0.89617144	0.0156109
mmu-miR-370-3p	ENSMUSG00000032355;	-0.875144363	0.022410212	ENSMUSG00000021451	-0.966717318	0.001643171
mmu-miR-370-3p	ENSMUSG00000032355;	-0.875144363	0.022410212	ENSMUSG00000029452	-0.907919824	0.012327775
mmu-miR-370-3p	ENSMUSG00000032355;	-0.875144363	0.022410212	ENSMUSG00000064080	-0.958985937	0.002488734
mmu-miR-370-3p	ENSMUSG00000032355;	-0.875144363	0.022410212	ENSMUSG00000032366	-0.940114054	0.005272105
mmu-miR-370-3p	ENSMUSG00000032355;	-0.875144363	0.022410212	ENSMUSG00000024862	-0.893424908	0.016432122
mmu-miR-370-3p	ENSMUSG00000032355;	-0.875144363	0.022410212	ENSMUSG00000031841	-0.905616249	0.01294204
mmu-miR-370-3p	ENSMUSG00000032355;	-0.875144363	0.022410212	ENSMUSG00000039376	-0.961235689	0.002224883
mmu-miR-370-3p	ENSMUSG00000032355;	-0.875144363	0.022410212	ENSMUSG00000052563	-0.927210427	0.007754652
mmu-miR-370-3p	ENSMUSG00000032355;	-0.875144363	0.022410212	ENSMUSG00000075254	-0.918442441	0.009706208
mmu-miR-370-3p	ENSMUSG00000032355;	-0.875144363	0.022410212	ENSMUSG00000020937	-0.874579845	0.02260888
mmu-miR-370-3p	ENSMUSG00000032355;	-0.875144363	0.022410212	ENSMUSG00000020728	-0.927091886	0.007779615
mmu-miR-370-3p	ENSMUSG00000032355;	-0.875144363	0.022410212	ENSMUSG00000021838	-0.903377208	0.013552912
mmu-miR-370-3p	ENSMUSG00000032355;	-0.875144363	0.022410212	ENSMUSG00000079020	-0.924839542	0.008261347
mmu-miR-370-3p	ENSMUSG00000032355;	-0.875144363	0.022410212	ENSMUSG00000031936	-0.955386029	0.00294121
mmu-miR-370-3p	ENSMUSG00000032355;	-0.875144363	0.022410212	ENSMUSG00000026478	-0.919391972	0.0094846
mmu-miR-370-3p	ENSMUSG00000032355;	-0.875144363	0.022410212	ENSMUSG00000026259	-0.868355256	0.024854784
mmu-miR-370-3p	ENSMUSG00000032355;	-0.875144363	0.022410212	ENSMUSG00000048027	-0.916065739	0.010271784
mmu-miR-370-3p	ENSMUSG00000032355;	-0.875144363	0.022410212	ENSMUSG00000039004	-0.852118962	0.031186212
mmu-miR-370-3p	ENSMUSG00000032355;	-0.875144363	0.022410212	ENSMUSG00000037032	-0.922792341	0.008711416
mmu-miR-370-3p	ENSMUSG00000032355;	-0.875144363	0.022410212	ENSMUSG00000067653	-0.895460607	0.015821499
mmu-miR-370-3p	ENSMUSG00000032355;	-0.875144363	0.022410212	ENSMUSG00000103144	-0.885341782	0.018966083
mmu-miR-370-3p	ENSMUSG00000032355;	-0.875144363	0.022410212	ENSMUSG00000053553	-0.876620883	0.021894546
mmu-miR-370-3p	ENSMUSG00000032355;	-0.875144363	0.022410212	ENSMUSG00000009281	-0.95141735	0.003483077
mmu-miR-370-3p	ENSMUSG00000032355;	-0.875144363	0.022410212	ENSMUSG00000009281	-0.91483994	0.010569553
mmu-miR-370-3p	ENSMUSG00000032355;	-0.875144363	0.022410212	ENSMUSG00000009281	-0.924024214	0.008439202

mmu-miR-370-3p	ENSMUSG00000032355;	-0.875144363	0.022410212	ENSMUSG00000025813	-0.885669502	0.018859961
mmu-miR-370-3p	ENSMUSG00000032355;	-0.875144363	0.022410212	ENSMUSG00000053329	-0.905670216	0.012927484
mmu-miR-370-3p	ENSMUSG00000032355;	-0.875144363	0.022410212	ENSMUSG00000040097	-0.899170706	0.014737277
mmu-miR-370-5p	ENSMUSG00000025019;	-0.908062711	0.012290149	ENSMUSG00000004558	-0.887347699	0.018321002
mmu-miR-370-5p	ENSMUSG00000025019;	-0.908062711	0.012290149	ENSMUSG00000054003	-0.870466313	0.024081743
mmu-miR-370-5p	ENSMUSG00000025019;	-0.908062711	0.012290149	ENSMUSG00000018340	-0.896971004	0.015375636
mmu-miR-370-5p	ENSMUSG00000025019;	-0.908062711	0.012290149	ENSMUSG00000051373	-0.875022092	0.022453171
mmu-miR-370-5p	ENSMUSG00000025019;	-0.908062711	0.012290149	ENSMUSG00000021200	-0.917891347	0.009835965
mmu-miR-376b-5p	ENSMUSG00000053063;	-0.881443361	0.020250318		-0.909431452	0.011932541
mmu-miR-378a-3p	ENSMUSG00000048410;	-0.850018403	0.03205484	ENSMUSG00000057058	-0.886112154	0.018717075
mmu-miR-378a-3p	ENSMUSG00000028053;	-0.885989083	0.018756749	ENSMUSG00000057058	-0.886112154	0.018717075
mmu-miR-382-5p	ENSMUSG00000034349;	-0.94086023	0.005142848		-0.891772925	0.016935812
mmu-miR-421-3p	ENSMUSG00000032355;	-0.881472242	0.020240656	ENSMUSG00000033439	-0.930866364	0.007003979
mmu-miR-423-5p	ENSMUSG00000106106;E	-0.85663189	0.029358198	ENSMUSG00000027895	-0.977140592	0.000777856
mmu-miR-423-5p	ENSMUSG00000106106;E	-0.85663189	0.029358198	ENSMUSG00000020882	-0.908070142	0.012288194
mmu-miR-423-5p	ENSMUSG00000106106;E	-0.85663189	0.029358198	ENSMUSG00000049097	-0.909446438	0.011928654
mmu-miR-423-5p	ENSMUSG00000106106;E	-0.85663189	0.029358198	ENSMUSG00000033871	-0.864200429	0.02641011
mmu-miR-423-5p	ENSMUSG00000106106;E	-0.85663189	0.029358198	ENSMUSG00000029478	-0.851856674	0.031294058
mmu-miR-423-5p	ENSMUSG00000106106;E	-0.85663189	0.029358198	ENSMUSG00000036026	-0.896403786	0.015542357
mmu-miR-423-5p	ENSMUSG00000106106;E	-0.85663189	0.029358198	ENSMUSG00000032816	-0.882909551	0.019762595
mmu-miR-429-3p	ENSMUSG00000020817;	-0.903171686	0.013609666	ENSMUSG00000048038	-0.853900541	0.030458327
mmu-miR-429-3p	ENSMUSG00000020817;	-0.903171686	0.013609666	ENSMUSG00000064302	-0.86088032	0.027685147
mmu-miR-429-3p	ENSMUSG00000022387;	-0.853900541	0.030458327	ENSMUSG00000048038	-0.853900541	0.030458327
mmu-miR-429-3p	ENSMUSG00000022387;	-0.853900541	0.030458327	ENSMUSG00000064302	-0.86088032	0.027685147
mmu-miR-429-3p	ENSMUSG00000026670;	-0.853900541	0.030458327	ENSMUSG00000048038	-0.853900541	0.030458327
mmu-miR-429-3p	ENSMUSG00000026670;	-0.853900541	0.030458327	ENSMUSG00000064302	-0.86088032	0.027685147
mmu-miR-429-3p	ENSMUSG00000028826;	-0.853900541	0.030458327	ENSMUSG00000048038	-0.853900541	0.030458327
mmu-miR-429-3p	ENSMUSG00000028826;	-0.853900541	0.030458327	ENSMUSG00000064302	-0.86088032	0.027685147
mmu-miR-429-3p	ENSMUSG00000029192;	-0.853900541	0.030458327	ENSMUSG00000048038	-0.853900541	0.030458327
mmu-miR-429-3p	ENSMUSG00000029192;	-0.853900541	0.030458327	ENSMUSG00000064302	-0.86088032	0.027685147
mmu-miR-429-3p	ENSMUSG00000034832;	-0.853900541	0.030458327	ENSMUSG00000048038	-0.853900541	0.030458327
mmu-miR-429-3p	ENSMUSG00000034832;	-0.853900541	0.030458327	ENSMUSG00000064302	-0.86088032	0.027685147
mmu-miR-433-3p	ENSMUSG00000038538;	-0.8807282	0.020490277	ENSMUSG00000024137	-0.864949222	0.026126493
mmu-miR-466d-3p	ENSMUSG00000021846;	-0.899980396	0.014505588	ENSMUSG00000020220	-0.879832144	0.02079284
mmu-miR-466d-3p	ENSMUSG00000021846;	-0.899980396	0.014505588	ENSMUSG00000020315	-0.891945145	0.016882961
mmu-miR-466f-3p	ENSMUSG00000030849;E	-0.860917987	0.027670522	ENSMUSG00000026456	-0.956005348	0.002860718
mmu-miR-466f-3p	ENSMUSG00000030849;E	-0.860917987	0.027670522	ENSMUSG00000029089	-0.889033417	0.017787176
mmu-miR-466f-3p	ENSMUSG00000030849;E	-0.860917987	0.027670522	ENSMUSG00000031028	-0.890176227	0.017429585

mmu-miR-466f-3p	ENSMUSG00000030849;E	-0.860917987	0.027670522	ENSMUSG00000074923	-0.878173253	0.021358573
mmu-miR-466f-3p	ENSMUSG00000030849;E	-0.860917987	0.027670522	ENSMUSG00000039068	-0.859833938	0.028092899
mmu-miR-466f-3p	ENSMUSG00000030849;E	-0.860917987	0.027670522	ENSMUSG00000047731	-0.950544921	0.003608229
mmu-miR-466f-3p	ENSMUSG00000030849;E	-0.860917987	0.027670522	ENSMUSG00000021905	-0.872797658	0.023241559
mmu-miR-466i-5p	ENSMUSG00000040785;	-0.859572284	0.028195302	ENSMUSG00000025475	-0.867027476	0.025346949
mmu-miR-466i-5p	ENSMUSG00000040785;	-0.859572284	0.028195302	ENSMUSG00000025532	-0.870929851	0.023913558
mmu-miR-466i-5p	ENSMUSG00000040785;	-0.859572284	0.028195302	ENSMUSG00000040325	-0.910915885	0.011550484
mmu-miR-466i-5p	ENSMUSG00000040785;	-0.859572284	0.028195302	ENSMUSG00000048387	-0.945650767	0.004350489
mmu-miR-466i-5p	ENSMUSG00000040785;	-0.859572284	0.028195302	ENSMUSG00000045757	-0.962950251	0.002033597
mmu-miR-466i-5p	ENSMUSG00000040785;	-0.859572284	0.028195302	ENSMUSG00000027523	-0.964375478	0.001881054
mmu-miR-466i-5p	ENSMUSG00000040785;	-0.859572284	0.028195302	ENSMUSG00000090053	-0.887609107	0.018237725
mmu-miR-466i-5p	ENSMUSG00000040785;	-0.859572284	0.028195302	ENSMUSG00000035623	-0.869616956	0.024391368
mmu-miR-466i-5p	ENSMUSG00000040785;	-0.859572284	0.028195302	ENSMUSG00000003283	-0.968829026	0.001442301
mmu-miR-466i-5p	ENSMUSG00000040785;	-0.859572284	0.028195302	ENSMUSG00000040272	-0.879113225	0.021037122
mmu-miR-466i-5p	ENSMUSG00000040785;	-0.859572284	0.028195302	ENSMUSG00000079477	-0.879508021	0.020902805
mmu-miR-466i-5p	ENSMUSG00000040785;	-0.859572284	0.028195302	ENSMUSG00000039977	-0.902988276	0.01366041
mmu-miR-466i-5p	ENSMUSG00000040785;	-0.859572284	0.028195302	ENSMUSG00000028849	-0.925506614	0.008117205
mmu-miR-466i-5p	ENSMUSG00000040785;	-0.859572284	0.028195302	ENSMUSG00000039943	-0.912118699	0.011245325
mmu-miR-466i-5p	ENSMUSG00000040785;	-0.859572284	0.028195302	ENSMUSG00000051111	-0.867243496	0.025266565
mmu-miR-466i-5p	ENSMUSG00000040785;	-0.859572284	0.028195302	ENSMUSG00000036913	-0.905102504	0.013081001
mmu-miR-466i-5p	ENSMUSG00000040785;	-0.859572284	0.028195302	ENSMUSG00000039763	-0.912503039	0.011148651
mmu-miR-466i-5p	ENSMUSG00000040785;	-0.859572284	0.028195302	ENSMUSG00000021972	-0.883509276	0.019564739
mmu-miR-466i-5p	ENSMUSG00000040785;	-0.859572284	0.028195302	ENSMUSG00000025656	-0.872715013	0.0232711
mmu-miR-466i-5p	ENSMUSG00000040785;	-0.859572284	0.028195302	ENSMUSG00000094800	-0.921739183	0.008947469
mmu-miR-466i-5p	ENSMUSG00000040785;	-0.859572284	0.028195302	ENSMUSG00000067242	-0.892308185	0.016771811
mmu-miR-466i-5p	ENSMUSG00000040785;	-0.859572284	0.028195302	ENSMUSG00000036864	-0.883214441	0.019661889
mmu-miR-466i-5p	ENSMUSG00000040785;	-0.859572284	0.028195302	ENSMUSG00000053825	-0.871357673	0.023758831
mmu-miR-466i-5p	ENSMUSG00000040785;	-0.859572284	0.028195302	ENSMUSG00000043487	-0.910243283	0.01172285
mmu-miR-466i-5p	ENSMUSG00000040785;	-0.859572284	0.028195302	ENSMUSG00000035765	-0.866543343	0.025527542
mmu-miR-466q	ENSMUSG00000023015;	-0.853956008	0.030435796	ENSMUSG00000030110	-0.927247211	0.007746913
mmu-miR-466q	ENSMUSG00000023015;	-0.853956008	0.030435796	ENSMUSG00000038500	-0.907844614	0.012347603
mmu-miR-466q	ENSMUSG00000023015;	-0.853956008	0.030435796	ENSMUSG00000006262	-0.869292284	0.024510222
mmu-miR-484	ENSMUSG00000057191;	-0.854649354	0.030154818		-0.890288195	0.017394737
mmu-miR-491-5p	ENSMUSG00000041852;	-0.863681978	0.026607333	ENSMUSG00000020232	-0.934820794	0.006234042
mmu-miR-497a-5p	ENSMUSG00000055013;	-0.937569041	0.005724771	ENSMUSG00000022841	-0.876496992	0.021937594
mmu-miR-497a-5p	ENSMUSG00000055013;	-0.937569041	0.005724771	ENSMUSG00000032281	-0.851801937	0.031316586
mmu-miR-497a-5p	ENSMUSG00000055013;	-0.937569041	0.005724771	ENSMUSG00000041264	-0.945746324	0.004335345
mmu-miR-497a-5p	ENSMUSG00000035107;	-0.896662091	0.015466327	ENSMUSG00000022841	-0.876496992	0.021937594

mmu-miR-497a-5p	ENSMUSG00000035107;	-0.896662091	0.015466327	ENSMUSG00000032281	-0.851801937	0.031316586
mmu-miR-497a-5p	ENSMUSG00000035107;	-0.896662091	0.015466327	ENSMUSG00000041264	-0.945746324	0.004335345
mmu-miR-497a-5p	ENSMUSG00000024135;	-0.97076926	0.001269166	ENSMUSG00000022841	-0.876496992	0.021937594
mmu-miR-497a-5p	ENSMUSG00000024135;	-0.97076926	0.001269166	ENSMUSG00000032281	-0.851801937	0.031316586
mmu-miR-497a-5p	ENSMUSG00000024135;	-0.97076926	0.001269166	ENSMUSG00000041264	-0.945746324	0.004335345
mmu-miR-497a-5p	ENSMUSG00000041258;	-0.913525165	0.010893521	ENSMUSG00000022841	-0.876496992	0.021937594
mmu-miR-497a-5p	ENSMUSG00000041258;	-0.913525165	0.010893521	ENSMUSG00000032281	-0.851801937	0.031316586
mmu-miR-497a-5p	ENSMUSG00000041258;	-0.913525165	0.010893521	ENSMUSG00000041264	-0.945746324	0.004335345
mmu-miR-497a-5p	ENSMUSG00000021932;	-0.94462888	0.004514059	ENSMUSG00000022841	-0.876496992	0.021937594
mmu-miR-497a-5p	ENSMUSG00000021932;	-0.94462888	0.004514059	ENSMUSG00000032281	-0.851801937	0.031316586
mmu-miR-497a-5p	ENSMUSG00000021932;	-0.94462888	0.004514059	ENSMUSG00000041264	-0.945746324	0.004335345
mmu-miR-500-3p	ENSMUSG00000030067;E	-0.913605199	0.010873664	ENSMUSG00000042590	-0.91873157	0.009638466
mmu-miR-503-5p	ENSMUSG00000042807;	-0.926739836	0.007853982	ENSMUSG00000021910	-0.885403662	0.018946023
mmu-miR-503-5p	ENSMUSG00000042807;	-0.926739836	0.007853982	ENSMUSG00000037907	-0.896575434	0.015491814
mmu-miR-503-5p	ENSMUSG00000042807;	-0.926739836	0.007853982	ENSMUSG00000022199	-0.923765663	0.008495987
mmu-miR-503-5p	ENSMUSG00000006005;	-0.850694165	0.031774168	ENSMUSG00000021910	-0.885403662	0.018946023
mmu-miR-503-5p	ENSMUSG00000006005;	-0.850694165	0.031774168	ENSMUSG00000037907	-0.896575434	0.015491814
mmu-miR-503-5p	ENSMUSG00000006005;	-0.850694165	0.031774168	ENSMUSG00000022199	-0.923765663	0.008495987
mmu-miR-503-5p	ENSMUSG00000046079;	-0.926739836	0.007853982	ENSMUSG00000021910	-0.885403662	0.018946023
mmu-miR-503-5p	ENSMUSG00000046079;	-0.926739836	0.007853982	ENSMUSG00000037907	-0.896575434	0.015491814
mmu-miR-503-5p	ENSMUSG00000046079;	-0.926739836	0.007853982	ENSMUSG00000022199	-0.923765663	0.008495987
mmu-miR-503-5p	ENSMUSG00000044471;	-0.879031658	0.021064924	ENSMUSG00000021910	-0.885403662	0.018946023
mmu-miR-503-5p	ENSMUSG00000044471;	-0.879031658	0.021064924	ENSMUSG00000037907	-0.896575434	0.015491814
mmu-miR-503-5p	ENSMUSG00000044471;	-0.879031658	0.021064924	ENSMUSG00000022199	-0.923765663	0.008495987
mmu-miR-503-5p	ENSMUSG00000022558;	-0.926739836	0.007853982	ENSMUSG00000021910	-0.885403662	0.018946023
mmu-miR-503-5p	ENSMUSG00000022558;	-0.926739836	0.007853982	ENSMUSG00000037907	-0.896575434	0.015491814
mmu-miR-503-5p	ENSMUSG00000022558;	-0.926739836	0.007853982	ENSMUSG00000022199	-0.923765663	0.008495987
mmu-miR-503-5p	ENSMUSG00000022994;	-0.926739836	0.007853982	ENSMUSG00000021910	-0.885403662	0.018946023
mmu-miR-503-5p	ENSMUSG00000022994;	-0.926739836	0.007853982	ENSMUSG00000037907	-0.896575434	0.015491814
mmu-miR-503-5p	ENSMUSG00000022994;	-0.926739836	0.007853982	ENSMUSG00000022199	-0.923765663	0.008495987
mmu-miR-503-5p	ENSMUSG00000028369;	-0.926739836	0.007853982	ENSMUSG00000021910	-0.885403662	0.018946023
mmu-miR-503-5p	ENSMUSG00000028369;	-0.926739836	0.007853982	ENSMUSG00000037907	-0.896575434	0.015491814
mmu-miR-503-5p	ENSMUSG00000028369;	-0.926739836	0.007853982	ENSMUSG00000022199	-0.923765663	0.008495987
mmu-miR-5107-5p	ENSMUSG00000040785;	-0.862178118	0.027183352	ENSMUSG00000004929	-0.868605136	0.024762675
mmu-miR-5107-5p	ENSMUSG00000040785;	-0.862178118	0.027183352	ENSMUSG00000025184	-0.868266411	0.024887573
mmu-miR-5107-5p	ENSMUSG00000040785;	-0.862178118	0.027183352	ENSMUSG00000025369	-0.958657378	0.002528487
mmu-miR-5107-5p	ENSMUSG00000040785;	-0.862178118	0.027183352	ENSMUSG00000041303	-0.89042697	0.017351593
mmu-miR-5107-5p	ENSMUSG00000040785;	-0.862178118	0.027183352	ENSMUSG00000041632	-0.870758234	0.02397576

mmu-miR-5107-5p	ENSMUSG00000040785;	-0.862178118	0.027183352	ENSMUSG00000051146	-0.951518221	0.003468747
mmu-miR-5107-5p	ENSMUSG00000040785;	-0.862178118	0.027183352	ENSMUSG00000050043	-0.868674197	0.024737247
mmu-miR-5107-5p	ENSMUSG00000040785;	-0.862178118	0.027183352	ENSMUSG00000053128	-0.901122825	0.014181698
mmu-miR-5107-5p	ENSMUSG00000040785;	-0.862178118	0.027183352	ENSMUSG00000061577	-0.879986078	0.020740711
mmu-miR-5107-5p	ENSMUSG00000040785;	-0.862178118	0.027183352	ENSMUSG00000025949	-0.985024578	0.000334716
mmu-miR-5107-5p	ENSMUSG00000040785;	-0.862178118	0.027183352	ENSMUSG00000032305	-0.885077954	0.019051723
mmu-miR-5107-5p	ENSMUSG00000040785;	-0.862178118	0.027183352	ENSMUSG00000075600	-0.938405465	0.005573989
mmu-miR-5107-5p	ENSMUSG00000040785;	-0.862178118	0.027183352	ENSMUSG00000020770	-0.885061151	0.019057183
mmu-miR-5107-5p	ENSMUSG00000040785;	-0.862178118	0.027183352	ENSMUSG00000020886	-0.867849549	0.025041692
mmu-miR-5107-5p	ENSMUSG00000040785;	-0.862178118	0.027183352	ENSMUSG00000001173	-0.890452054	0.0173438
mmu-miR-5107-5p	ENSMUSG00000040785;	-0.862178118	0.027183352	ENSMUSG00000046711	-0.948788849	0.00386672
mmu-miR-5107-5p	ENSMUSG00000040785;	-0.862178118	0.027183352	ENSMUSG00000032279	-0.912716725	0.011095077
mmu-miR-5107-5p	ENSMUSG00000040785;	-0.862178118	0.027183352	ENSMUSG00000029098	-0.864652503	0.026238706
mmu-miR-5107-5p	ENSMUSG00000040785;	-0.862178118	0.027183352	ENSMUSG00000022516	-0.906941757	0.01258682
mmu-miR-5107-5p	ENSMUSG00000040785;	-0.862178118	0.027183352	ENSMUSG00000054008	-0.863169767	0.026802866
mmu-miR-511-5p	ENSMUSG00000039166;	-0.886134758	0.018709792	ENSMUSG00000053134	-0.992615555	8.16E-05
mmu-miR-511-5p	ENSMUSG00000022019;	-0.886903399	0.018462961	ENSMUSG00000053134	-0.992615555	8.16E-05
mmu-miR-5132-3p	ENSMUSG00000034154;	-0.885747702	0.01883468	ENSMUSG00000026424	-0.910008222	0.01178338
mmu-miR-540-5p	ENSMUSG00000041852;	-0.911441589	0.011416624	ENSMUSG00000040280	-0.876571642	0.021911651
mmu-miR-540-5p	ENSMUSG00000041852;	-0.911441589	0.011416624	ENSMUSG00000038861	-0.925002302	0.008226064
mmu-miR-540-5p	ENSMUSG00000041852;	-0.911441589	0.011416624		-0.894868342	0.015998006
mmu-miR-541-5p	ENSMUSG00000022801;	-0.871713004	0.023630685	ENSMUSG00000033278	-0.903452142	0.013532248
mmu-miR-541-5p	ENSMUSG00000022801;	-0.871713004	0.023630685		-0.88706767	0.018410413
mmu-miR-541-5p	ENSMUSG00000022801;	-0.871713004	0.023630685		-0.894232744	0.016188475
mmu-miR-574-5p	ENSMUSG00000027620;	-0.888106583	0.018079745	ENSMUSG00000049327	-0.903905947	0.013407431
mmu-miR-574-5p	ENSMUSG00000027620;	-0.888106583	0.018079745	ENSMUSG00000007617	-0.904095434	0.013355479
mmu-miR-574-5p	ENSMUSG00000027620;	-0.888106583	0.018079745	ENSMUSG00000028868	-0.851087915	0.031611165
mmu-miR-574-5p	ENSMUSG00000027620;	-0.888106583	0.018079745	ENSMUSG00000025825	-0.894879911	0.015994549
mmu-miR-574-5p	ENSMUSG00000027620;	-0.888106583	0.018079745	ENSMUSG00000048264	-0.908609812	0.012146597
mmu-miR-574-5p	ENSMUSG00000027620;	-0.888106583	0.018079745	ENSMUSG00000102091	-0.939293916	0.005415985
mmu-miR-574-5p	ENSMUSG00000027620;	-0.888106583	0.018079745	ENSMUSG00000032329	-0.894885502	0.015992878
mmu-miR-574-5p	ENSMUSG00000027620;	-0.888106583	0.018079745	ENSMUSG00000015599	-0.974402471	0.000974464
mmu-miR-615-5p	ENSMUSG00000042439;	-0.884571013	0.019216797	ENSMUSG00000031903	-0.874475687	0.022645627
mmu-miR-615-5p	ENSMUSG00000042439;	-0.884571013	0.019216797	ENSMUSG00000045691	-0.928431858	0.007499712
mmu-miR-615-5p	ENSMUSG00000042439;	-0.884571013	0.019216797	ENSMUSG00000050373	-0.875282614	0.022361686
mmu-miR-615-5p	ENSMUSG00000042439;	-0.884571013	0.019216797	ENSMUSG00000028104	-0.910508175	0.01165482
mmu-miR-615-5p	ENSMUSG00000042439;	-0.884571013	0.019216797	ENSMUSG00000071658	-0.856274123	0.02950121
mmu-miR-615-5p	ENSMUSG00000042439;	-0.884571013	0.019216797	ENSMUSG00000018474	-0.882075031	0.020039498

mmu-miR-615-5p	ENSMUSG00000042439;	-0.884571013	0.019216797	ENSMUSG00000032312	-0.856772611	0.029302038
mmu-miR-653-5p	ENSMUSG00000020305;	-0.853608411	0.030577124		-0.871685307	0.023640662
mmu-miR-6539	ENSMUSG00000073557;	-0.85019706	0.031980523	ENSMUSG00000027895	-0.873338764	0.023048586
mmu-miR-6539	ENSMUSG00000073557;	-0.85019706	0.031980523	ENSMUSG00000027255	-0.862589071	0.027025365
mmu-miR-6539	ENSMUSG00000073557;	-0.85019706	0.031980523	ENSMUSG00000038112	-0.85651969	0.029403013
mmu-miR-6539	ENSMUSG00000073557;	-0.85019706	0.031980523	ENSMUSG00000038387	-0.861063469	0.027614069
mmu-miR-6539	ENSMUSG00000073557;	-0.85019706	0.031980523	ENSMUSG00000050945	-0.855238903	0.029916872
mmu-miR-6539	ENSMUSG00000073557;	-0.85019706	0.031980523	ENSMUSG00000004892	-0.971937173	0.001170233
mmu-miR-6539	ENSMUSG00000073557;	-0.85019706	0.031980523	ENSMUSG00000074457	-0.903140354	0.013618328
mmu-miR-6539	ENSMUSG00000073557;	-0.85019706	0.031980523	ENSMUSG00000030965	-0.886738451	0.018515798
mmu-miR-6539	ENSMUSG00000073557;	-0.85019706	0.031980523	ENSMUSG00000023277	-0.88928679	0.017707593
mmu-miR-6539	ENSMUSG00000073557;	-0.85019706	0.031980523	ENSMUSG00000107283	-0.954439132	0.003066402
mmu-miR-6539	ENSMUSG00000073557;	-0.85019706	0.031980523	ENSMUSG00000031691	-0.932724278	0.006636788
mmu-miR-654-3p	ENSMUSG00000036591;	-0.859183244	0.028347885		-0.971126841	0.001238454
mmu-miR-664-5p	ENSMUSG00000038538;	-0.94009828	0.005274854	ENSMUSG00000029120	-0.858882397	0.028466146
mmu-miR-664-5p	ENSMUSG00000038538;	-0.94009828	0.005274854	ENSMUSG00000030337	-0.907274823	0.012498314
mmu-miR-664-5p	ENSMUSG00000038538;	-0.94009828	0.005274854	ENSMUSG00000040140	-0.877003266	0.021761935
mmu-miR-664-5p	ENSMUSG00000038538;	-0.94009828	0.005274854	ENSMUSG00000022620	-0.907195439	0.012519382
mmu-miR-664-5p	ENSMUSG00000038538;	-0.94009828	0.005274854	ENSMUSG00000022637	-0.853540641	0.030604714
mmu-miR-665-3p	ENSMUSG00000037174;	-0.928720359	0.007440102	ENSMUSG00000040158	-0.866044618	0.025714216
mmu-miR-665-3p	ENSMUSG00000037174;	-0.928720359	0.007440102	ENSMUSG00000038502	-0.857176124	0.029141281
mmu-miR-665-3p	ENSMUSG00000037174;	-0.928720359	0.007440102	ENSMUSG00000039157	-0.877869586	0.021462918
mmu-miR-665-3p	ENSMUSG00000037174;	-0.928720359	0.007440102	ENSMUSG00000031442	-0.899525863	0.014635432
mmu-miR-665-3p	ENSMUSG00000037174;	-0.928720359	0.007440102	ENSMUSG00000036120	-0.905093008	0.013083576
mmu-miR-666-3p	ENSMUSG00000037174;	-0.872315042	0.02341432	ENSMUSG00000070802	-0.904760309	0.013173958
mmu-miR-666-3p	ENSMUSG00000037174;	-0.872315042	0.02341432	ENSMUSG00000047215	-0.975729217	0.000876458
mmu-miR-666-3p	ENSMUSG00000037174;	-0.872315042	0.02341432	ENSMUSG00000030330	-0.890709496	0.017263916
mmu-miR-666-3p	ENSMUSG00000037174;	-0.872315042	0.02341432	ENSMUSG00000028184	-0.90988481	0.01181522
mmu-miR-667-3p	ENSMUSG00000038910;	-0.883149623	0.019683278	ENSMUSG00000049295	-0.90600456	0.012837483
mmu-miR-667-3p	ENSMUSG00000038910;	-0.883149623	0.019683278	ENSMUSG00000036986	-0.852255079	0.031130313
mmu-miR-667-3p	ENSMUSG00000038910;	-0.883149623	0.019683278	ENSMUSG00000060098	-0.867873428	0.025032851
mmu-miR-667-3p	ENSMUSG00000038910;	-0.883149623	0.019683278	ENSMUSG00000041515	-0.864703325	0.02621947
mmu-miR-667-3p	ENSMUSG00000038910;	-0.883149623	0.019683278		-0.885905673	0.018783661
mmu-miR-667-3p	ENSMUSG00000045038;	-0.926186168	0.007971636	ENSMUSG00000049295	-0.90600456	0.012837483
mmu-miR-667-3p	ENSMUSG00000045038;	-0.926186168	0.007971636	ENSMUSG00000036986	-0.852255079	0.031130313
mmu-miR-667-3p	ENSMUSG00000045038;	-0.926186168	0.007971636	ENSMUSG00000060098	-0.867873428	0.025032851
mmu-miR-667-3p	ENSMUSG00000045038;	-0.926186168	0.007971636	ENSMUSG00000041515	-0.864703325	0.02621947
mmu-miR-667-3p	ENSMUSG00000045038;	-0.926186168	0.007971636		-0.885905673	0.018783661

mmu-miR-669f-5p	ENSMUSG00000005103;	-0.866814466	0.025426331	ENSMUSG00000006611	-0.88036643	0.020612178
mmu-miR-669f-5p	ENSMUSG00000108596;E	-0.945107155	0.004437134	ENSMUSG00000006611	-0.88036643	0.020612178
mmu-miR-669l-5p	ENSMUSG00000039652;	-0.855332687	0.029879103		-0.907184937	0.01252217
mmu-miR-669m-5p	ENSMUSG00000021458;	-0.859951526	0.028046937	ENSMUSG00000028011	-0.953430262	0.003202612
mmu-miR-669m-5p	ENSMUSG00000021458;	-0.859951526	0.028046937	ENSMUSG00000043727	-0.916524968	0.010161291
mmu-miR-669m-5p	ENSMUSG00000021458;	-0.859951526	0.028046937	ENSMUSG00000017561	-0.855123261	0.029963476
mmu-miR-669m-5p	ENSMUSG00000021458;	-0.859951526	0.028046937	ENSMUSG00000040749	-0.851349368	0.031503148
mmu-miR-669m-5p	ENSMUSG00000021458;	-0.859951526	0.028046937	ENSMUSG00000074342	-0.954165627	0.00310304
mmu-miR-669m-5p	ENSMUSG00000021458;	-0.859951526	0.028046937	ENSMUSG00000014592	-0.914836469	0.010570402
mmu-miR-669m-5p	ENSMUSG00000021458;	-0.859951526	0.028046937	ENSMUSG00000028555	-0.859951526	0.028046937
mmu-miR-669m-5p	ENSMUSG00000021458;	-0.859951526	0.028046937	ENSMUSG00000079477	-0.851201893	0.031564055
mmu-miR-669m-5p	ENSMUSG00000021458;	-0.859951526	0.028046937	ENSMUSG00000020738	-0.860682444	0.027762038
mmu-miR-669m-5p	ENSMUSG00000021458;	-0.859951526	0.028046937	ENSMUSG00000027601	-0.910393274	0.011684305
mmu-miR-669m-5p	ENSMUSG00000021458;	-0.859951526	0.028046937	ENSMUSG00000020738	-0.859951526	0.028046937
mmu-miR-669m-5p	ENSMUSG00000021458;	-0.859951526	0.028046937	ENSMUSG00000057103	-0.896130514	0.015622988
mmu-miR-669m-5p	ENSMUSG00000021458;	-0.859951526	0.028046937	ENSMUSG00000030823	-0.859951526	0.028046937
mmu-miR-669m-5p	ENSMUSG00000021458;	-0.859951526	0.028046937	ENSMUSG00000005763	-0.881106702	0.02036311
mmu-miR-669m-5p	ENSMUSG00000021458;	-0.859951526	0.028046937	ENSMUSG00000030228	-0.859951526	0.028046937
mmu-miR-669m-5p	ENSMUSG00000047888;	-0.859951526	0.028046937	ENSMUSG00000028011	-0.953430262	0.003202612
mmu-miR-669m-5p	ENSMUSG00000047888;	-0.859951526	0.028046937	ENSMUSG00000043727	-0.916524968	0.010161291
mmu-miR-669m-5p	ENSMUSG00000047888;	-0.859951526	0.028046937	ENSMUSG00000017561	-0.855123261	0.029963476
mmu-miR-669m-5p	ENSMUSG00000047888;	-0.859951526	0.028046937	ENSMUSG00000040749	-0.851349368	0.031503148
mmu-miR-669m-5p	ENSMUSG00000047888;	-0.859951526	0.028046937	ENSMUSG00000074342	-0.954165627	0.00310304
mmu-miR-669m-5p	ENSMUSG00000047888;	-0.859951526	0.028046937	ENSMUSG00000014592	-0.914836469	0.010570402
mmu-miR-669m-5p	ENSMUSG00000047888;	-0.859951526	0.028046937	ENSMUSG00000028555	-0.859951526	0.028046937
mmu-miR-669m-5p	ENSMUSG00000047888;	-0.859951526	0.028046937	ENSMUSG00000079477	-0.851201893	0.031564055
mmu-miR-669m-5p	ENSMUSG00000047888;	-0.859951526	0.028046937	ENSMUSG00000020738	-0.860682444	0.027762038
mmu-miR-669m-5p	ENSMUSG00000047888;	-0.859951526	0.028046937	ENSMUSG00000027601	-0.910393274	0.011684305
mmu-miR-669m-5p	ENSMUSG00000047888;	-0.859951526	0.028046937	ENSMUSG00000020738	-0.859951526	0.028046937
mmu-miR-669m-5p	ENSMUSG00000047888;	-0.859951526	0.028046937	ENSMUSG00000057103	-0.896130514	0.015622988
mmu-miR-669m-5p	ENSMUSG00000047888;	-0.859951526	0.028046937	ENSMUSG00000030823	-0.859951526	0.028046937
mmu-miR-669m-5p	ENSMUSG00000047888;	-0.859951526	0.028046937	ENSMUSG00000005763	-0.881106702	0.02036311
mmu-miR-669m-5p	ENSMUSG00000047888;	-0.859951526	0.028046937	ENSMUSG00000030228	-0.859951526	0.028046937
mmu-miR-669m-5p	ENSMUSG00000042439;	-0.859951526	0.028046937	ENSMUSG00000028011	-0.953430262	0.003202612
mmu-miR-669m-5p	ENSMUSG00000042439;	-0.859951526	0.028046937	ENSMUSG00000043727	-0.916524968	0.010161291
mmu-miR-669m-5p	ENSMUSG00000042439;	-0.859951526	0.028046937	ENSMUSG00000017561	-0.855123261	0.029963476
mmu-miR-669m-5p	ENSMUSG00000042439;	-0.859951526	0.028046937	ENSMUSG00000040749	-0.851349368	0.031503148
mmu-miR-669m-5p	ENSMUSG00000042439;	-0.859951526	0.028046937	ENSMUSG00000074342	-0.954165627	0.00310304

mmu-miR-669m-5p	ENSMUSG00000042439;	-0.859951526	0.028046937	ENSMUSG00000014592	-0.914836469	0.010570402
mmu-miR-669m-5p	ENSMUSG00000042439;	-0.859951526	0.028046937	ENSMUSG00000028555	-0.859951526	0.028046937
mmu-miR-669m-5p	ENSMUSG00000042439;	-0.859951526	0.028046937	ENSMUSG00000079477	-0.851201893	0.031564055
mmu-miR-669m-5p	ENSMUSG00000042439;	-0.859951526	0.028046937	ENSMUSG00000020738	-0.860682444	0.027762038
mmu-miR-669m-5p	ENSMUSG00000042439;	-0.859951526	0.028046937	ENSMUSG00000027601	-0.910393274	0.011684305
mmu-miR-669m-5p	ENSMUSG00000042439;	-0.859951526	0.028046937	ENSMUSG00000020738	-0.859951526	0.028046937
mmu-miR-669m-5p	ENSMUSG00000042439;	-0.859951526	0.028046937	ENSMUSG00000057103	-0.896130514	0.015622988
mmu-miR-669m-5p	ENSMUSG00000042439;	-0.859951526	0.028046937	ENSMUSG00000030823	-0.859951526	0.028046937
mmu-miR-669m-5p	ENSMUSG00000042439;	-0.859951526	0.028046937	ENSMUSG00000005763	-0.881106702	0.02036311
mmu-miR-669m-5p	ENSMUSG00000042439;	-0.859951526	0.028046937	ENSMUSG00000030228	-0.859951526	0.028046937
mmu-miR-669m-5p	ENSMUSG00000029376;	-0.859951526	0.028046937	ENSMUSG00000028011	-0.953430262	0.003202612
mmu-miR-669m-5p	ENSMUSG00000029376;	-0.859951526	0.028046937	ENSMUSG00000043727	-0.916524968	0.010161291
mmu-miR-669m-5p	ENSMUSG00000029376;	-0.859951526	0.028046937	ENSMUSG00000017561	-0.855123261	0.029963476
mmu-miR-669m-5p	ENSMUSG00000029376;	-0.859951526	0.028046937	ENSMUSG00000040749	-0.851349368	0.031503148
mmu-miR-669m-5p	ENSMUSG00000029376;	-0.859951526	0.028046937	ENSMUSG00000074342	-0.954165627	0.00310304
mmu-miR-669m-5p	ENSMUSG00000029376;	-0.859951526	0.028046937	ENSMUSG00000014592	-0.914836469	0.010570402
mmu-miR-669m-5p	ENSMUSG00000029376;	-0.859951526	0.028046937	ENSMUSG00000028555	-0.859951526	0.028046937
mmu-miR-669m-5p	ENSMUSG00000029376;	-0.859951526	0.028046937	ENSMUSG00000079477	-0.851201893	0.031564055
mmu-miR-669m-5p	ENSMUSG00000029376;	-0.859951526	0.028046937	ENSMUSG00000020738	-0.860682444	0.027762038
mmu-miR-669m-5p	ENSMUSG00000029376;	-0.859951526	0.028046937	ENSMUSG00000027601	-0.910393274	0.011684305
mmu-miR-669m-5p	ENSMUSG00000029376;	-0.859951526	0.028046937	ENSMUSG00000020738	-0.859951526	0.028046937
mmu-miR-669m-5p	ENSMUSG00000029376;	-0.859951526	0.028046937	ENSMUSG00000057103	-0.896130514	0.015622988
mmu-miR-669m-5p	ENSMUSG00000029376;	-0.859951526	0.028046937	ENSMUSG00000030823	-0.859951526	0.028046937
mmu-miR-669m-5p	ENSMUSG00000029376;	-0.859951526	0.028046937	ENSMUSG00000005763	-0.881106702	0.02036311
mmu-miR-669m-5p	ENSMUSG00000029376;	-0.859951526	0.028046937	ENSMUSG00000030228	-0.859951526	0.028046937
mmu-miR-669m-5p	ENSMUSG00000046311;	-0.859951526	0.028046937	ENSMUSG00000028011	-0.953430262	0.003202612
mmu-miR-669m-5p	ENSMUSG00000046311;	-0.859951526	0.028046937	ENSMUSG00000043727	-0.916524968	0.010161291
mmu-miR-669m-5p	ENSMUSG00000046311;	-0.859951526	0.028046937	ENSMUSG00000017561	-0.855123261	0.029963476
mmu-miR-669m-5p	ENSMUSG00000046311;	-0.859951526	0.028046937	ENSMUSG00000040749	-0.851349368	0.031503148
mmu-miR-669m-5p	ENSMUSG00000046311;	-0.859951526	0.028046937	ENSMUSG00000074342	-0.954165627	0.00310304
mmu-miR-669m-5p	ENSMUSG00000046311;	-0.859951526	0.028046937	ENSMUSG00000014592	-0.914836469	0.010570402
mmu-miR-669m-5p	ENSMUSG00000046311;	-0.859951526	0.028046937	ENSMUSG00000028555	-0.859951526	0.028046937
mmu-miR-669m-5p	ENSMUSG00000046311;	-0.859951526	0.028046937	ENSMUSG00000079477	-0.851201893	0.031564055
mmu-miR-669m-5p	ENSMUSG00000046311;	-0.859951526	0.028046937	ENSMUSG00000020738	-0.860682444	0.027762038
mmu-miR-669m-5p	ENSMUSG00000046311;	-0.859951526	0.028046937	ENSMUSG00000027601	-0.910393274	0.011684305
mmu-miR-669m-5p	ENSMUSG00000046311;	-0.859951526	0.028046937	ENSMUSG00000020738	-0.859951526	0.028046937
mmu-miR-669m-5p	ENSMUSG00000046311;	-0.859951526	0.028046937	ENSMUSG00000057103	-0.896130514	0.015622988
mmu-miR-669m-5p	ENSMUSG00000046311;	-0.859951526	0.028046937	ENSMUSG00000030823	-0.859951526	0.028046937

mmu-miR-669m-5p	ENSMUSG00000046311;	-0.859951526	0.028046937	ENSMUSG00000005763	-0.881106702	0.02036311
mmu-miR-669m-5p	ENSMUSG00000046311;	-0.859951526	0.028046937	ENSMUSG00000030228	-0.859951526	0.028046937
mmu-miR-669m-5p	ENSMUSG00000018171;	-0.859951526	0.028046937	ENSMUSG00000028011	-0.953430262	0.003202612
mmu-miR-669m-5p	ENSMUSG00000018171;	-0.859951526	0.028046937	ENSMUSG00000043727	-0.916524968	0.010161291
mmu-miR-669m-5p	ENSMUSG00000018171;	-0.859951526	0.028046937	ENSMUSG00000017561	-0.855123261	0.029963476
mmu-miR-669m-5p	ENSMUSG00000018171;	-0.859951526	0.028046937	ENSMUSG00000040749	-0.851349368	0.031503148
mmu-miR-669m-5p	ENSMUSG00000018171;	-0.859951526	0.028046937	ENSMUSG00000074342	-0.954165627	0.00310304
mmu-miR-669m-5p	ENSMUSG00000018171;	-0.859951526	0.028046937	ENSMUSG00000014592	-0.914836469	0.010570402
mmu-miR-669m-5p	ENSMUSG00000018171;	-0.859951526	0.028046937	ENSMUSG00000028555	-0.859951526	0.028046937
mmu-miR-669m-5p	ENSMUSG00000018171;	-0.859951526	0.028046937	ENSMUSG00000079477	-0.851201893	0.031564055
mmu-miR-669m-5p	ENSMUSG00000018171;	-0.859951526	0.028046937	ENSMUSG00000020738	-0.860682444	0.027762038
mmu-miR-669m-5p	ENSMUSG00000018171;	-0.859951526	0.028046937	ENSMUSG00000027601	-0.910393274	0.011684305
mmu-miR-669m-5p	ENSMUSG00000018171;	-0.859951526	0.028046937	ENSMUSG00000020738	-0.859951526	0.028046937
mmu-miR-669m-5p	ENSMUSG00000018171;	-0.859951526	0.028046937	ENSMUSG00000057103	-0.896130514	0.015622988
mmu-miR-669m-5p	ENSMUSG00000018171;	-0.859951526	0.028046937	ENSMUSG00000030823	-0.859951526	0.028046937
mmu-miR-669m-5p	ENSMUSG00000018171;	-0.859951526	0.028046937	ENSMUSG00000005763	-0.881106702	0.02036311
mmu-miR-669m-5p	ENSMUSG00000018171;	-0.859951526	0.028046937	ENSMUSG00000030228	-0.859951526	0.028046937
mmu-miR-669m-5p	ENSMUSG00000021189;	-0.859951526	0.028046937	ENSMUSG00000028011	-0.953430262	0.003202612
mmu-miR-669m-5p	ENSMUSG00000021189;	-0.859951526	0.028046937	ENSMUSG00000043727	-0.916524968	0.010161291
mmu-miR-669m-5p	ENSMUSG00000021189;	-0.859951526	0.028046937	ENSMUSG00000017561	-0.855123261	0.029963476
mmu-miR-669m-5p	ENSMUSG00000021189;	-0.859951526	0.028046937	ENSMUSG00000040749	-0.851349368	0.031503148
mmu-miR-669m-5p	ENSMUSG00000021189;	-0.859951526	0.028046937	ENSMUSG00000074342	-0.954165627	0.00310304
mmu-miR-669m-5p	ENSMUSG00000021189;	-0.859951526	0.028046937	ENSMUSG00000014592	-0.914836469	0.010570402
mmu-miR-669m-5p	ENSMUSG00000021189;	-0.859951526	0.028046937	ENSMUSG00000028555	-0.859951526	0.028046937
mmu-miR-669m-5p	ENSMUSG00000021189;	-0.859951526	0.028046937	ENSMUSG00000079477	-0.851201893	0.031564055
mmu-miR-669m-5p	ENSMUSG00000021189;	-0.859951526	0.028046937	ENSMUSG00000020738	-0.860682444	0.027762038
mmu-miR-669m-5p	ENSMUSG00000021189;	-0.859951526	0.028046937	ENSMUSG00000027601	-0.910393274	0.011684305
mmu-miR-669m-5p	ENSMUSG00000021189;	-0.859951526	0.028046937	ENSMUSG00000020738	-0.859951526	0.028046937
mmu-miR-669m-5p	ENSMUSG00000021189;	-0.859951526	0.028046937	ENSMUSG00000057103	-0.896130514	0.015622988
mmu-miR-669m-5p	ENSMUSG00000021189;	-0.859951526	0.028046937	ENSMUSG00000030823	-0.859951526	0.028046937
mmu-miR-669m-5p	ENSMUSG00000021189;	-0.859951526	0.028046937	ENSMUSG00000005763	-0.881106702	0.02036311
mmu-miR-669m-5p	ENSMUSG00000021189;	-0.859951526	0.028046937	ENSMUSG00000030228	-0.859951526	0.028046937
mmu-miR-669m-5p	ENSMUSG00000021189;	-0.859951526	0.028046937	ENSMUSG00000028011	-0.953430262	0.003202612
mmu-miR-669m-5p	ENSMUSG00000021189;	-0.859951526	0.028046937	ENSMUSG00000043727	-0.916524968	0.010161291
mmu-miR-669m-5p	ENSMUSG00000021189;	-0.859951526	0.028046937	ENSMUSG00000017561	-0.855123261	0.029963476
mmu-miR-669m-5p	ENSMUSG00000021189;	-0.859951526	0.028046937	ENSMUSG00000040749	-0.851349368	0.031503148
mmu-miR-669m-5p	ENSMUSG00000021189;	-0.859951526	0.028046937	ENSMUSG00000074342	-0.954165627	0.00310304
mmu-miR-669m-5p	ENSMUSG00000021189;	-0.859951526	0.028046937	ENSMUSG00000014592	-0.914836469	0.010570402

mmu-miR-669m-5p	ENSMUSG00000021189;	-0.859951526	0.028046937	ENSMUSG00000028555	-0.859951526	0.028046937
mmu-miR-669m-5p	ENSMUSG00000021189;	-0.859951526	0.028046937	ENSMUSG00000079477	-0.851201893	0.031564055
mmu-miR-669m-5p	ENSMUSG00000021189;	-0.859951526	0.028046937	ENSMUSG00000020738	-0.860682444	0.027762038
mmu-miR-669m-5p	ENSMUSG00000021189;	-0.859951526	0.028046937	ENSMUSG00000027601	-0.910393274	0.011684305
mmu-miR-669m-5p	ENSMUSG00000021189;	-0.859951526	0.028046937	ENSMUSG00000020738	-0.859951526	0.028046937
mmu-miR-669m-5p	ENSMUSG00000021189;	-0.859951526	0.028046937	ENSMUSG00000057103	-0.896130514	0.015622988
mmu-miR-669m-5p	ENSMUSG00000021189;	-0.859951526	0.028046937	ENSMUSG00000030823	-0.859951526	0.028046937
mmu-miR-669m-5p	ENSMUSG00000021189;	-0.859951526	0.028046937	ENSMUSG00000005763	-0.881106702	0.02036311
mmu-miR-669m-5p	ENSMUSG00000021189;	-0.859951526	0.028046937	ENSMUSG00000030228	-0.859951526	0.028046937
mmu-miR-669m-5p	ENSMUSG00000056214;	-0.859951526	0.028046937	ENSMUSG00000028011	-0.953430262	0.003202612
mmu-miR-669m-5p	ENSMUSG00000056214;	-0.859951526	0.028046937	ENSMUSG00000043727	-0.916524968	0.010161291
mmu-miR-669m-5p	ENSMUSG00000056214;	-0.859951526	0.028046937	ENSMUSG00000017561	-0.855123261	0.029963476
mmu-miR-669m-5p	ENSMUSG00000056214;	-0.859951526	0.028046937	ENSMUSG00000040749	-0.851349368	0.031503148
mmu-miR-669m-5p	ENSMUSG00000056214;	-0.859951526	0.028046937	ENSMUSG00000074342	-0.954165627	0.00310304
mmu-miR-669m-5p	ENSMUSG00000056214;	-0.859951526	0.028046937	ENSMUSG00000014592	-0.914836469	0.010570402
mmu-miR-669m-5p	ENSMUSG00000056214;	-0.859951526	0.028046937	ENSMUSG00000028555	-0.859951526	0.028046937
mmu-miR-669m-5p	ENSMUSG00000056214;	-0.859951526	0.028046937	ENSMUSG00000079477	-0.851201893	0.031564055
mmu-miR-669m-5p	ENSMUSG00000056214;	-0.859951526	0.028046937	ENSMUSG00000020738	-0.860682444	0.027762038
mmu-miR-669m-5p	ENSMUSG00000056214;	-0.859951526	0.028046937	ENSMUSG00000027601	-0.910393274	0.011684305
mmu-miR-669m-5p	ENSMUSG00000056214;	-0.859951526	0.028046937	ENSMUSG00000020738	-0.859951526	0.028046937
mmu-miR-669m-5p	ENSMUSG00000056214;	-0.859951526	0.028046937	ENSMUSG00000057103	-0.896130514	0.015622988
mmu-miR-669m-5p	ENSMUSG00000056214;	-0.859951526	0.028046937	ENSMUSG00000030823	-0.859951526	0.028046937
mmu-miR-669m-5p	ENSMUSG00000056214;	-0.859951526	0.028046937	ENSMUSG00000005763	-0.881106702	0.02036311
mmu-miR-669m-5p	ENSMUSG00000056214;	-0.859951526	0.028046937	ENSMUSG00000030228	-0.859951526	0.028046937
mmu-miR-671-5p	ENSMUSG00000030276;	-0.976599669	0.000814957	ENSMUSG00000017446	-0.859257636	0.028318678
mmu-miR-671-5p	ENSMUSG00000030276;	-0.976599669	0.000814957	ENSMUSG00000038793	-0.946846995	0.004162778
mmu-miR-671-5p	ENSMUSG00000030276;	-0.976599669	0.000814957	ENSMUSG00000060224	-0.92190265	0.008910629
mmu-miR-671-5p	ENSMUSG00000030276;	-0.976599669	0.000814957	ENSMUSG00000020823	-0.869890823	0.024291327
mmu-miR-671-5p	ENSMUSG00000030276;	-0.976599669	0.000814957	ENSMUSG00000031601	-0.905920165	0.012860172
mmu-miR-673-5p	ENSMUSG00000040785;	-0.852022377	0.031225904	ENSMUSG00000112129	-0.856929722	0.029239397
mmu-miR-673-5p	ENSMUSG00000040785;	-0.852022377	0.031225904	ENSMUSG00000025754	-0.897778566	0.015139765
mmu-miR-673-5p	ENSMUSG00000040785;	-0.852022377	0.031225904	ENSMUSG00000028849	-0.900921085	0.014238636
mmu-miR-673-5p	ENSMUSG00000040785;	-0.852022377	0.031225904	ENSMUSG00000022194	-0.969438077	0.001386774
mmu-miR-679-5p	ENSMUSG00000001472;	-0.873572452	0.022965483	ENSMUSG00000052026	-0.857170958	0.029143337
mmu-miR-679-5p	ENSMUSG00000001472;	-0.873572452	0.022965483	ENSMUSG00000041241	-0.960827762	0.002271642
mmu-miR-679-5p	ENSMUSG00000001472;	-0.873572452	0.022965483		-0.968167948	0.001503792
mmu-miR-6903-5p	ENSMUSG00000024566;	-0.859188083	0.028345985		-0.851127962	0.031594609
mmu-miR-6916-5p	ENSMUSG00000024566;	-0.859188083	0.028345985	ENSMUSG00000023439	-0.944670458	0.004507346

mmu-miR-6916-5p	ENSMUSG00000024566;	-0.859188083	0.028345985		-0.851127962	0.031594609
mmu-miR-6929-3p	ENSMUSG00000040084;	-0.953649408	0.003172777	ENSMUSG00000022711	-0.914799796	0.010579375
mmu-miR-6929-3p	ENSMUSG00000040084;	-0.953649408	0.003172777	ENSMUSG00000029111	-0.943168242	0.004752994
mmu-miR-6929-3p	ENSMUSG00000040084;	-0.953649408	0.003172777	ENSMUSG00000050628	-0.936322681	0.005953102
mmu-miR-6929-3p	ENSMUSG00000040084;	-0.953649408	0.003172777	ENSMUSG00000063406	-0.912127716	0.011243053
mmu-miR-6929-3p	ENSMUSG00000040084;	-0.953649408	0.003172777	ENSMUSG00000078517	-0.878272412	0.021324553
mmu-miR-6929-3p	ENSMUSG00000040084;	-0.953649408	0.003172777	ENSMUSG00000003208	-0.94087086	0.005141018
mmu-miR-6929-3p	ENSMUSG00000040084;	-0.953649408	0.003172777	ENSMUSG00000028894	-0.96976574	0.001357347
mmu-miR-6929-3p	ENSMUSG00000040084;	-0.953649408	0.003172777	ENSMUSG00000011877	-0.870584186	0.024038922
mmu-miR-6929-3p	ENSMUSG00000040084;	-0.953649408	0.003172777	ENSMUSG00000039230	-0.890426583	0.017351713
mmu-miR-6929-3p	ENSMUSG00000040084;	-0.953649408	0.003172777	ENSMUSG00000030304	-0.894530932	0.016098982
mmu-miR-6929-3p	ENSMUSG00000040084;	-0.953649408	0.003172777	ENSMUSG00000017718	-0.951463091	0.003476575
mmu-miR-6929-3p	ENSMUSG00000040084;	-0.953649408	0.003172777	ENSMUSG00000026439	-0.874526974	0.022627529
mmu-miR-6937-5p	ENSMUSG00000034007;	-0.946833769	0.004164831	ENSMUSG00000103088	-0.912984709	0.011028066
mmu-miR-6937-5p	ENSMUSG00000034007;	-0.946833769	0.004164831	ENSMUSG00000009828	-0.917086365	0.010027004
mmu-miR-6958-3p	ENSMUSG00000024566;	-0.859188083	0.028345985	ENSMUSG00000034006	-0.933479708	0.006490249
mmu-miR-6958-3p	ENSMUSG00000024566;	-0.859188083	0.028345985		-0.851127962	0.031594609
mmu-miR-6986-5p	ENSMUSG00000005312;	-0.903053975	0.013642222	ENSMUSG00000027439	-0.872716121	0.023270704
mmu-miR-6986-5p	ENSMUSG00000005312;	-0.903053975	0.013642222	ENSMUSG00000017670	-0.937553986	0.005727503
mmu-miR-6986-5p	ENSMUSG00000005312;	-0.903053975	0.013642222	ENSMUSG00000039021	-0.950160585	0.003664051
mmu-miR-6986-5p	ENSMUSG00000005312;	-0.903053975	0.013642222	ENSMUSG00000003526	-0.86018627	0.027955288
mmu-miR-6986-5p	ENSMUSG00000005312;	-0.903053975	0.013642222	ENSMUSG00000025505	-0.859838989	0.028090924
mmu-miR-6992-5p	ENSMUSG00000026004;	-0.861529028	0.027433779	ENSMUSG00000030353	-0.851017967	0.031640093
mmu-miR-6992-5p	ENSMUSG00000026004;	-0.861529028	0.027433779	ENSMUSG00000028949	-0.902446816	0.013810747
mmu-miR-6992-5p	ENSMUSG00000026004;	-0.861529028	0.027433779	ENSMUSG00000037621	-0.901507467	0.014073441
mmu-miR-6992-5p	ENSMUSG00000026004;	-0.861529028	0.027433779	ENSMUSG00000042041	-0.961841811	0.002156291
mmu-miR-6992-5p	ENSMUSG00000026004;	-0.861529028	0.027433779	ENSMUSG00000002781	-0.865617708	0.025874522
mmu-miR-6992-5p	ENSMUSG00000026004;	-0.861529028	0.027433779	ENSMUSG00000058979	-0.964888808	0.001827551
mmu-miR-6992-5p	ENSMUSG00000026004;	-0.861529028	0.027433779	ENSMUSG00000039137	-0.854323783	0.030286602
mmu-miR-6992-5p	ENSMUSG00000026004;	-0.861529028	0.027433779	ENSMUSG00000060601	-0.864749515	0.026201993
mmu-miR-6992-5p	ENSMUSG00000026004;	-0.861529028	0.027433779	ENSMUSG00000079415	-0.971289633	0.001224595
mmu-miR-6992-5p	ENSMUSG00000026004;	-0.861529028	0.027433779	ENSMUSG00000022636	-0.970359767	0.001304795
mmu-miR-6992-5p	ENSMUSG00000026004;	-0.861529028	0.027433779	ENSMUSG00000060600	-0.872869512	0.02321589
mmu-miR-6992-5p	ENSMUSG00000026004;	-0.861529028	0.027433779	ENSMUSG00000022091	-0.944526887	0.004530547
mmu-miR-6992-5p	ENSMUSG00000026004;	-0.861529028	0.027433779	ENSMUSG00000022091	-0.916418202	0.010186928
mmu-miR-6992-5p	ENSMUSG00000026004;	-0.861529028	0.027433779	ENSMUSG00000021891	-0.946882735	0.004157232
mmu-miR-7012-5p	ENSMUSG00000020422;	-0.934826421	0.006232978	ENSMUSG00000005947	-0.880108602	0.020699265
mmu-miR-7012-5p	ENSMUSG00000020422;	-0.934826421	0.006232978	ENSMUSG00000015016	-0.885076585	0.019052168

mmu-miR-7012-5p	ENSMUSG00000020422;	-0.934826421	0.006232978	ENSMUSG00000028078	-0.85707257	0.029182497
mmu-miR-7012-5p	ENSMUSG00000020422;	-0.934826421	0.006232978	ENSMUSG00000030546	-0.85538128	0.029859542
mmu-miR-7012-5p	ENSMUSG00000020422;	-0.934826421	0.006232978	ENSMUSG00000038526	-0.857946837	0.028835399
mmu-miR-7012-5p	ENSMUSG00000020422;	-0.934826421	0.006232978	ENSMUSG00000041329	-0.859073979	0.02839081
mmu-miR-7012-5p	ENSMUSG00000020422;	-0.934826421	0.006232978	ENSMUSG00000054013	-0.914690468	0.010606145
mmu-miR-7012-5p	ENSMUSG00000020422;	-0.934826421	0.006232978	ENSMUSG00000017631	-0.951777695	0.003432018
mmu-miR-7012-5p	ENSMUSG00000020422;	-0.934826421	0.006232978	ENSMUSG00000037224	-0.864909196	0.026141616
mmu-miR-7012-5p	ENSMUSG00000020422;	-0.934826421	0.006232978	ENSMUSG00000030683	-0.870219606	0.024171484
mmu-miR-7012-5p	ENSMUSG00000020422;	-0.934826421	0.006232978	ENSMUSG00000013367	-0.8710727	0.023861842
mmu-miR-7012-5p	ENSMUSG00000020422;	-0.934826421	0.006232978	ENSMUSG00000059436	-0.880525331	0.020558593
mmu-miR-7012-5p	ENSMUSG00000020422;	-0.934826421	0.006232978	ENSMUSG00000027194	-0.850740036	0.031755158
mmu-miR-7012-5p	ENSMUSG00000020422;	-0.934826421	0.006232978	ENSMUSG00000037341	-0.862502099	0.027058764
mmu-miR-7012-5p	ENSMUSG00000020422;	-0.934826421	0.006232978	ENSMUSG00000036948	-0.897966253	0.015085197
mmu-miR-7012-5p	ENSMUSG00000020422;	-0.934826421	0.006232978	ENSMUSG00000037787	-0.921684789	0.008959744
mmu-miR-7012-5p	ENSMUSG00000020422;	-0.934826421	0.006232978	ENSMUSG00000066900	-0.899752265	0.014570687
mmu-miR-7012-5p	ENSMUSG00000020422;	-0.934826421	0.006232978	ENSMUSG00000040648	-0.877567715	0.021566887
mmu-miR-7012-5p	ENSMUSG00000020422;	-0.934826421	0.006232978	ENSMUSG00000024442	-0.862818703	0.026937275
mmu-miR-7012-5p	ENSMUSG00000020422;	-0.934826421	0.006232978		-0.878995673	0.021077195
mmu-miR-7024-5p	ENSMUSG00000024566;	-0.859188083	0.028345985	ENSMUSG00000103088	-0.929350002	0.007310811
mmu-miR-7024-5p	ENSMUSG00000024566;	-0.859188083	0.028345985	ENSMUSG00000061046	-0.904226284	0.01331966
mmu-miR-7024-5p	ENSMUSG00000024566;	-0.859188083	0.028345985	ENSMUSG00000061689	-0.931800383	0.006818177
mmu-miR-7024-5p	ENSMUSG00000024566;	-0.859188083	0.028345985	ENSMUSG00000085795	-0.913257552	0.010960042
mmu-miR-7024-5p	ENSMUSG00000024566;	-0.859188083	0.028345985		-0.851127962	0.031594609
mmu-miR-7031-5p	ENSMUSG00000021596;	-0.953509358	0.003191828	ENSMUSG00000000876	-0.856905128	0.029249198
mmu-miR-7031-5p	ENSMUSG00000021596;	-0.953509358	0.003191828	ENSMUSG00000005262	-0.902043501	0.013923244
mmu-miR-7031-5p	ENSMUSG00000021596;	-0.953509358	0.003191828	ENSMUSG00000020956	-0.900190165	0.014445852
mmu-miR-7031-5p	ENSMUSG00000021596;	-0.953509358	0.003191828	ENSMUSG00000029594	-0.879610772	0.020867915
mmu-miR-7031-5p	ENSMUSG00000021596;	-0.953509358	0.003191828	ENSMUSG00000005142	-0.874492133	0.022639823
mmu-miR-7031-5p	ENSMUSG00000021596;	-0.953509358	0.003191828	ENSMUSG00000046591	-0.867261416	0.025259902
mmu-miR-7031-5p	ENSMUSG00000021596;	-0.953509358	0.003191828	ENSMUSG00000026339	-0.932996751	0.00658375
mmu-miR-7031-5p	ENSMUSG00000021596;	-0.953509358	0.003191828	ENSMUSG00000025228	-0.850790717	0.031734161
mmu-miR-7031-5p	ENSMUSG00000021596;	-0.953509358	0.003191828	ENSMUSG00000036398	-0.891029705	0.017164802
mmu-miR-7031-5p	ENSMUSG00000021596;	-0.953509358	0.003191828	ENSMUSG00000040552	-0.918744434	0.009635457
mmu-miR-7031-5p	ENSMUSG00000021596;	-0.953509358	0.003191828	ENSMUSG00000036565	-0.851719988	0.031350329
mmu-miR-7031-5p	ENSMUSG00000021596;	-0.953509358	0.003191828	ENSMUSG00000024454	-0.942647031	0.004839717
mmu-miR-7031-5p	ENSMUSG00000021596;	-0.953509358	0.003191828	ENSMUSG00000050106	-0.927273272	0.007741433
mmu-miR-7031-5p	ENSMUSG00000021596;	-0.953509358	0.003191828	ENSMUSG00000054000	-0.910264774	0.011717324
mmu-miR-7031-5p	ENSMUSG00000021596;	-0.953509358	0.003191828	ENSMUSG00000013646	-0.944189251	0.004585339

mmu-miR-7031-5p	ENSMUSG00000021596;	-0.953509358	0.003191828	ENSMUSG00000003208	-0.876771849	0.021842145
mmu-miR-7031-5p	ENSMUSG00000021596;	-0.953509358	0.003191828	ENSMUSG00000029723	-0.956152173	0.002841796
mmu-miR-7031-5p	ENSMUSG00000021596;	-0.953509358	0.003191828	ENSMUSG00000018168	-0.888499833	0.01795533
mmu-miR-7031-5p	ENSMUSG00000021596;	-0.953509358	0.003191828	ENSMUSG00000020770	-0.927269741	0.007742176
mmu-miR-7031-5p	ENSMUSG00000021596;	-0.953509358	0.003191828	ENSMUSG00000059851	-0.989934426	0.000151464
mmu-miR-7031-5p	ENSMUSG00000021596;	-0.953509358	0.003191828	ENSMUSG00000018899	-0.868757462	0.024706605
mmu-miR-7031-5p	ENSMUSG00000021596;	-0.953509358	0.003191828	ENSMUSG00000031902	-0.883815871	0.019463958
mmu-miR-7031-5p	ENSMUSG00000021596;	-0.953509358	0.003191828	ENSMUSG00000022090	-0.872533295	0.023336117
mmu-miR-7031-5p	ENSMUSG00000021596;	-0.953509358	0.003191828	ENSMUSG00000090394	-0.912146872	0.011238225
mmu-miR-7031-5p	ENSMUSG00000021596;	-0.953509358	0.003191828	ENSMUSG00000026288	-0.89251711	0.016708006
mmu-miR-7031-5p	ENSMUSG00000021596;	-0.953509358	0.003191828	ENSMUSG00000029705	-0.907724603	0.012379273
mmu-miR-7031-5p	ENSMUSG00000021596;	-0.953509358	0.003191828	ENSMUSG00000029705	-0.912475403	0.011155589
mmu-miR-7031-5p	ENSMUSG00000021596;	-0.953509358	0.003191828	ENSMUSG00000046876	-0.914564125	0.010637123
mmu-miR-7031-5p	ENSMUSG00000021596;	-0.953509358	0.003191828	ENSMUSG00000079658	-0.910395709	0.01168368
mmu-miR-7031-5p	ENSMUSG00000021596;	-0.953509358	0.003191828	ENSMUSG00000029175	-0.865990777	0.025734407
mmu-miR-7031-5p	ENSMUSG00000021596;	-0.953509358	0.003191828	ENSMUSG00000032436	-0.863525498	0.026666996
mmu-miR-7031-5p	ENSMUSG00000021596;	-0.953509358	0.003191828	ENSMUSG00000062054	-0.859627738	0.028173584
mmu-miR-7031-5p	ENSMUSG00000026436;	-0.882420356	0.019924691	ENSMUSG00000000876	-0.856905128	0.029249198
mmu-miR-7031-5p	ENSMUSG00000026436;	-0.882420356	0.019924691	ENSMUSG00000005262	-0.902043501	0.013923244
mmu-miR-7031-5p	ENSMUSG00000026436;	-0.882420356	0.019924691	ENSMUSG00000020956	-0.900190165	0.014445852
mmu-miR-7031-5p	ENSMUSG00000026436;	-0.882420356	0.019924691	ENSMUSG00000029594	-0.879610772	0.020867915
mmu-miR-7031-5p	ENSMUSG00000026436;	-0.882420356	0.019924691	ENSMUSG00000005142	-0.874492133	0.022639823
mmu-miR-7031-5p	ENSMUSG00000026436;	-0.882420356	0.019924691	ENSMUSG00000046591	-0.867261416	0.025259902
mmu-miR-7031-5p	ENSMUSG00000026436;	-0.882420356	0.019924691	ENSMUSG00000026339	-0.932996751	0.00658375
mmu-miR-7031-5p	ENSMUSG00000026436;	-0.882420356	0.019924691	ENSMUSG00000025228	-0.850790717	0.031734161
mmu-miR-7031-5p	ENSMUSG00000026436;	-0.882420356	0.019924691	ENSMUSG00000036398	-0.891029705	0.017164802
mmu-miR-7031-5p	ENSMUSG00000026436;	-0.882420356	0.019924691	ENSMUSG00000040552	-0.918744434	0.009635457
mmu-miR-7031-5p	ENSMUSG00000026436;	-0.882420356	0.019924691	ENSMUSG00000036565	-0.851719988	0.031350329
mmu-miR-7031-5p	ENSMUSG00000026436;	-0.882420356	0.019924691	ENSMUSG00000024454	-0.942647031	0.004839717
mmu-miR-7031-5p	ENSMUSG00000026436;	-0.882420356	0.019924691	ENSMUSG00000050106	-0.927273272	0.007741433
mmu-miR-7031-5p	ENSMUSG00000026436;	-0.882420356	0.019924691	ENSMUSG00000054000	-0.910264774	0.011717324
mmu-miR-7031-5p	ENSMUSG00000026436;	-0.882420356	0.019924691	ENSMUSG00000013646	-0.944189251	0.004585339
mmu-miR-7031-5p	ENSMUSG00000026436;	-0.882420356	0.019924691	ENSMUSG00000003208	-0.876771849	0.021842145
mmu-miR-7031-5p	ENSMUSG00000026436;	-0.882420356	0.019924691	ENSMUSG00000029723	-0.956152173	0.002841796
mmu-miR-7031-5p	ENSMUSG00000026436;	-0.882420356	0.019924691	ENSMUSG00000018168	-0.888499833	0.01795533
mmu-miR-7031-5p	ENSMUSG00000026436;	-0.882420356	0.019924691	ENSMUSG00000020770	-0.927269741	0.007742176
mmu-miR-7031-5p	ENSMUSG00000026436;	-0.882420356	0.019924691	ENSMUSG00000059851	-0.989934426	0.000151464
mmu-miR-7031-5p	ENSMUSG00000026436;	-0.882420356	0.019924691	ENSMUSG00000018899	-0.868757462	0.024706605

mmu-miR-7031-5p	ENSMUSG00000026436;	-0.882420356	0.019924691	ENSMUSG00000031902	-0.883815871	0.019463958
mmu-miR-7031-5p	ENSMUSG00000026436;	-0.882420356	0.019924691	ENSMUSG00000022090	-0.872533295	0.023336117
mmu-miR-7031-5p	ENSMUSG00000026436;	-0.882420356	0.019924691	ENSMUSG00000090394	-0.912146872	0.011238225
mmu-miR-7031-5p	ENSMUSG00000026436;	-0.882420356	0.019924691	ENSMUSG00000026288	-0.89251711	0.016708006
mmu-miR-7031-5p	ENSMUSG00000026436;	-0.882420356	0.019924691	ENSMUSG00000029705	-0.907724603	0.012379273
mmu-miR-7031-5p	ENSMUSG00000026436;	-0.882420356	0.019924691	ENSMUSG00000029705	-0.912475403	0.011155589
mmu-miR-7031-5p	ENSMUSG00000026436;	-0.882420356	0.019924691	ENSMUSG00000046876	-0.914564125	0.010637123
mmu-miR-7031-5p	ENSMUSG00000026436;	-0.882420356	0.019924691	ENSMUSG00000079658	-0.910395709	0.01168368
mmu-miR-7031-5p	ENSMUSG00000026436;	-0.882420356	0.019924691	ENSMUSG00000029175	-0.865990777	0.025734407
mmu-miR-7031-5p	ENSMUSG00000026436;	-0.882420356	0.019924691	ENSMUSG00000032436	-0.863525498	0.026666996
mmu-miR-7031-5p	ENSMUSG00000026436;	-0.882420356	0.019924691	ENSMUSG00000062054	-0.859627738	0.028173584
mmu-miR-7031-5p	ENSMUSG00000038784;	-0.89885011	0.014829503	ENSMUSG00000000876	-0.856905128	0.029249198
mmu-miR-7031-5p	ENSMUSG00000038784;	-0.89885011	0.014829503	ENSMUSG00000005262	-0.902043501	0.013923244
mmu-miR-7031-5p	ENSMUSG00000038784;	-0.89885011	0.014829503	ENSMUSG00000020956	-0.900190165	0.014445852
mmu-miR-7031-5p	ENSMUSG00000038784;	-0.89885011	0.014829503	ENSMUSG00000029594	-0.879610772	0.020867915
mmu-miR-7031-5p	ENSMUSG00000038784;	-0.89885011	0.014829503	ENSMUSG00000005142	-0.874492133	0.022639823
mmu-miR-7031-5p	ENSMUSG00000038784;	-0.89885011	0.014829503	ENSMUSG00000046591	-0.867261416	0.025259902
mmu-miR-7031-5p	ENSMUSG00000038784;	-0.89885011	0.014829503	ENSMUSG00000026339	-0.932996751	0.00658375
mmu-miR-7031-5p	ENSMUSG00000038784;	-0.89885011	0.014829503	ENSMUSG00000025228	-0.850790717	0.031734161
mmu-miR-7031-5p	ENSMUSG00000038784;	-0.89885011	0.014829503	ENSMUSG00000036398	-0.891029705	0.017164802
mmu-miR-7031-5p	ENSMUSG00000038784;	-0.89885011	0.014829503	ENSMUSG00000040552	-0.918744434	0.009635457
mmu-miR-7031-5p	ENSMUSG00000038784;	-0.89885011	0.014829503	ENSMUSG00000036565	-0.851719988	0.031350329
mmu-miR-7031-5p	ENSMUSG00000038784;	-0.89885011	0.014829503	ENSMUSG00000024454	-0.942647031	0.004839717
mmu-miR-7031-5p	ENSMUSG00000038784;	-0.89885011	0.014829503	ENSMUSG00000050106	-0.927273272	0.007741433
mmu-miR-7031-5p	ENSMUSG00000038784;	-0.89885011	0.014829503	ENSMUSG00000054000	-0.910264774	0.011717324
mmu-miR-7031-5p	ENSMUSG00000038784;	-0.89885011	0.014829503	ENSMUSG00000013646	-0.944189251	0.004585339
mmu-miR-7031-5p	ENSMUSG00000038784;	-0.89885011	0.014829503	ENSMUSG00000003208	-0.876771849	0.021842145
mmu-miR-7031-5p	ENSMUSG00000038784;	-0.89885011	0.014829503	ENSMUSG00000029723	-0.956152173	0.002841796
mmu-miR-7031-5p	ENSMUSG00000038784;	-0.89885011	0.014829503	ENSMUSG00000018168	-0.888499833	0.01795533
mmu-miR-7031-5p	ENSMUSG00000038784;	-0.89885011	0.014829503	ENSMUSG00000020770	-0.927269741	0.007742176
mmu-miR-7031-5p	ENSMUSG00000038784;	-0.89885011	0.014829503	ENSMUSG00000059851	-0.989934426	0.000151464
mmu-miR-7031-5p	ENSMUSG00000038784;	-0.89885011	0.014829503	ENSMUSG00000018899	-0.868757462	0.024706605
mmu-miR-7031-5p	ENSMUSG00000038784;	-0.89885011	0.014829503	ENSMUSG00000031902	-0.883815871	0.019463958
mmu-miR-7031-5p	ENSMUSG00000038784;	-0.89885011	0.014829503	ENSMUSG00000022090	-0.872533295	0.023336117
mmu-miR-7031-5p	ENSMUSG00000038784;	-0.89885011	0.014829503	ENSMUSG00000090394	-0.912146872	0.011238225
mmu-miR-7031-5p	ENSMUSG00000038784;	-0.89885011	0.014829503	ENSMUSG00000026288	-0.89251711	0.016708006
mmu-miR-7031-5p	ENSMUSG00000038784;	-0.89885011	0.014829503	ENSMUSG00000029705	-0.907724603	0.012379273
mmu-miR-7031-5p	ENSMUSG00000038784;	-0.89885011	0.014829503	ENSMUSG00000029705	-0.912475403	0.011155589

mmu-miR-7031-5p	ENSMUSG00000038784;	-0.89885011	0.014829503	ENSMUSG00000046876	-0.914564125	0.010637123
mmu-miR-7031-5p	ENSMUSG00000038784;	-0.89885011	0.014829503	ENSMUSG00000079658	-0.910395709	0.01168368
mmu-miR-7031-5p	ENSMUSG00000038784;	-0.89885011	0.014829503	ENSMUSG00000029175	-0.865990777	0.025734407
mmu-miR-7031-5p	ENSMUSG00000038784;	-0.89885011	0.014829503	ENSMUSG00000032436	-0.863525498	0.026666996
mmu-miR-7031-5p	ENSMUSG00000038784;	-0.89885011	0.014829503	ENSMUSG00000062054	-0.859627738	0.028173584
mmu-miR-7038-5p	ENSMUSG00000034154;	-0.885747702	0.01883468	ENSMUSG00000045503	-0.866954792	0.025374023
mmu-miR-7038-5p	ENSMUSG00000033721;	-0.970632464	0.001281014	ENSMUSG00000045503	-0.866954792	0.025374023
mmu-miR-7043-3p	ENSMUSG00000040785;	-0.876353246	0.021987592	ENSMUSG00000023452	-0.913694554	0.010851516
mmu-miR-7043-3p	ENSMUSG00000040785;	-0.876353246	0.021987592	ENSMUSG00000035642	-0.94122281	0.005080607
mmu-miR-7043-3p	ENSMUSG00000040785;	-0.876353246	0.021987592	ENSMUSG00000079278	-0.954537438	0.003053285
mmu-miR-7043-3p	ENSMUSG00000040785;	-0.876353246	0.021987592	ENSMUSG00000046792	-0.850505663	0.031852344
mmu-miR-7043-3p	ENSMUSG00000040785;	-0.876353246	0.021987592	ENSMUSG00000055254	-0.882289356	0.019968206
mmu-miR-7051-5p	ENSMUSG00000040084;	-0.87987633	0.02077787	ENSMUSG00000020346	-0.990261184	0.000141805
mmu-miR-7051-5p	ENSMUSG00000040084;	-0.87987633	0.02077787	ENSMUSG00000033819	-0.877044749	0.021747572
mmu-miR-7051-5p	ENSMUSG00000040084;	-0.87987633	0.02077787	ENSMUSG00000003233	-0.959335215	0.002446815
mmu-miR-7051-5p	ENSMUSG00000040084;	-0.87987633	0.02077787		-0.936930366	0.00584123
mmu-miR-7054-5p	ENSMUSG00000049878;	-0.878096088	0.021385065	ENSMUSG00000029048	-0.870947063	0.023907324
mmu-miR-7054-5p	ENSMUSG00000049878;	-0.878096088	0.021385065	ENSMUSG00000041193	-0.851961312	0.031251012
mmu-miR-7054-5p	ENSMUSG00000049878;	-0.878096088	0.021385065	ENSMUSG00000002320	-0.856609854	0.029366998
mmu-miR-7054-5p	ENSMUSG00000049878;	-0.878096088	0.021385065	ENSMUSG00000000131	-0.877118882	0.021721916
mmu-miR-7054-5p	ENSMUSG00000049878;	-0.878096088	0.021385065	ENSMUSG00000024960	-0.898913175	0.014811339
mmu-miR-7059-5p	ENSMUSG00000028580;	-0.908779405	0.012102263	ENSMUSG00000039515	-0.871432998	0.023731639
mmu-miR-7061-5p	ENSMUSG00000024566;	-0.859188083	0.028345985		-0.851127962	0.031594609
mmu-miR-7069-3p	ENSMUSG00000042439;	-0.875387536	0.022324891	ENSMUSG00000005958	-0.897066724	0.015347587
mmu-miR-7069-3p	ENSMUSG00000042439;	-0.875387536	0.022324891	ENSMUSG00000018930	-0.875638721	0.022236923
mmu-miR-7069-3p	ENSMUSG00000042439;	-0.875387536	0.022324891	ENSMUSG00000025875	-0.864175741	0.026419485
mmu-miR-7069-3p	ENSMUSG00000042439;	-0.875387536	0.022324891	ENSMUSG00000027380	-0.888238765	0.018037879
mmu-miR-7069-3p	ENSMUSG00000042439;	-0.875387536	0.022324891	ENSMUSG00000028069	-0.961938444	0.002145454
mmu-miR-7069-3p	ENSMUSG00000042439;	-0.875387536	0.022324891	ENSMUSG00000074577	-0.92618016	0.007972917
mmu-miR-7069-3p	ENSMUSG00000042439;	-0.875387536	0.022324891	ENSMUSG00000025375	-0.917575684	0.009910666
mmu-miR-7069-3p	ENSMUSG00000042439;	-0.875387536	0.022324891	ENSMUSG00000013523	-0.873323258	0.023054105
mmu-miR-7069-3p	ENSMUSG00000042439;	-0.875387536	0.022324891	ENSMUSG00000041025	-0.870522758	0.024061233
mmu-miR-7069-3p	ENSMUSG00000042439;	-0.875387536	0.022324891	ENSMUSG00000096188	-0.933398087	0.006506005
mmu-miR-7075-3p	ENSMUSG00000004730;	-0.874361212	0.022686047	ENSMUSG00000021892	-0.869931841	0.02427636
mmu-miR-7075-3p	ENSMUSG00000004730;	-0.874361212	0.022686047	ENSMUSG00000002778	-0.892192107	0.016807312
mmu-miR-7081-3p	ENSMUSG00000022797;	-0.89583894	0.01570924	ENSMUSG00000017802	-0.867102606	0.025318978
mmu-miR-7081-3p	ENSMUSG00000022797;	-0.89583894	0.01570924	ENSMUSG00000038150	-0.901584604	0.01405178
mmu-miR-7081-3p	ENSMUSG00000022797;	-0.89583894	0.01570924	ENSMUSG00000090877	-0.861692365	0.027370659

mmu-miR-708-5p	ENSMUSG00000025019;	-0.866226172	0.025646185	ENSMUSG00000027782	-0.873556001	0.022971329
mmu-miR-708-5p	ENSMUSG00000014932;	-0.913748626	0.010838124	ENSMUSG00000027782	-0.873556001	0.022971329
mmu-miR-708-5p	n/a	-0.879713508	0.020833057	ENSMUSG00000027782	-0.873556001	0.022971329
mmu-miR-708-5p	ENSMUSG00000062604;	-0.911871115	0.011307815	ENSMUSG00000027782	-0.873556001	0.022971329
mmu-miR-758-3p	ENSMUSG00000001911;	-0.858901241	0.028458732		-0.892093677	0.016837443
mmu-miR-7648-3p	ENSMUSG00000024566;	-0.859188083	0.028345985	ENSMUSG00000028063	-0.90438431	0.013276464
mmu-miR-7648-3p	ENSMUSG00000024566;	-0.859188083	0.028345985	ENSMUSG00000038248	-0.866402538	0.02558018
mmu-miR-7648-3p	ENSMUSG00000024566;	-0.859188083	0.028345985	ENSMUSG00000039263	-0.921058182	0.009101741
mmu-miR-7648-3p	ENSMUSG00000024566;	-0.859188083	0.028345985	ENSMUSG00000036955	-0.986793768	0.000260455
mmu-miR-7648-3p	ENSMUSG00000024566;	-0.859188083	0.028345985	ENSMUSG00000026031	-0.870159829	0.024193252
mmu-miR-7648-3p	ENSMUSG00000024566;	-0.859188083	0.028345985	ENSMUSG00000069727	-0.857851839	0.02887302
mmu-miR-7648-3p	ENSMUSG00000024566;	-0.859188083	0.028345985	ENSMUSG00000027575	-0.889738167	0.017566244
mmu-miR-7648-3p	ENSMUSG00000024566;	-0.859188083	0.028345985	ENSMUSG00000054690	-0.858940791	0.028443174
mmu-miR-7648-3p	ENSMUSG00000024566;	-0.859188083	0.028345985	ENSMUSG00000070643	-0.870914291	0.023919195
mmu-miR-7648-3p	ENSMUSG00000024566;	-0.859188083	0.028345985	ENSMUSG00000085795	-0.913257552	0.010960042
mmu-miR-7648-3p	ENSMUSG00000024566;	-0.859188083	0.028345985	ENSMUSG00000055707	-0.953315287	0.00321832
mmu-miR-7648-3p	ENSMUSG00000024566;	-0.859188083	0.028345985	ENSMUSG00000040260	-0.862024441	0.027242544
mmu-miR-7648-3p	ENSMUSG00000024566;	-0.859188083	0.028345985		-0.851127962	0.031594609
mmu-miR-7649-3p	ENSMUSG00000041852;	-0.948728278	0.003875793	ENSMUSG00000020399	-0.883063109	0.019711843
mmu-miR-7649-3p	ENSMUSG00000041852;	-0.948728278	0.003875793	ENSMUSG00000027936	-0.851764091	0.031332168
mmu-miR-7649-3p	ENSMUSG00000041852;	-0.948728278	0.003875793	ENSMUSG00000038172	-0.9282404	0.007539399
mmu-miR-7649-3p	ENSMUSG00000041852;	-0.948728278	0.003875793	ENSMUSG00000037214	-0.866356128	0.025597541
mmu-miR-7649-3p	ENSMUSG00000041852;	-0.948728278	0.003875793	ENSMUSG00000037343	-0.868406287	0.02483596
mmu-miR-7649-3p	ENSMUSG00000041852;	-0.948728278	0.003875793	ENSMUSG00000033352	-0.882908019	0.019763101
mmu-miR-7649-3p	ENSMUSG00000041852;	-0.948728278	0.003875793	ENSMUSG00000074476	-0.899473676	0.014650376
mmu-miR-7649-3p	ENSMUSG00000041852;	-0.948728278	0.003875793	ENSMUSG00000029826	-0.887686024	0.018213255
mmu-miR-7649-3p	ENSMUSG00000041852;	-0.948728278	0.003875793	ENSMUSG00000032400	-0.864076024	0.026457371
mmu-miR-7649-3p	ENSMUSG00000041852;	-0.948728278	0.003875793	ENSMUSG00000026939	-0.860037108	0.028013507
mmu-miR-7649-3p	ENSMUSG00000041852;	-0.948728278	0.003875793	ENSMUSG00000040557	-0.861765799	0.027342304
mmu-miR-7649-3p	ENSMUSG00000041852;	-0.948728278	0.003875793	ENSMUSG00000048442	-0.873077372	0.023141709
mmu-miR-7649-3p	ENSMUSG00000041852;	-0.948728278	0.003875793	ENSMUSG00000078676	-0.861686945	0.027372753
mmu-miR-7649-3p	ENSMUSG00000041852;	-0.948728278	0.003875793	ENSMUSG00000033697	-0.892961467	0.016572688
mmu-miR-7649-3p	ENSMUSG00000041852;	-0.948728278	0.003875793		-0.904911521	0.013132842
mmu-miR-7649-5p	ENSMUSG00000041852;	-0.886858495	0.018477338	ENSMUSG00000020439	-0.85131933	0.031515549
mmu-miR-7649-5p	ENSMUSG00000040084;	-0.933336724	0.006517863	ENSMUSG00000020439	-0.85131933	0.031515549
mmu-miR-7667-3p	ENSMUSG00000094936;	-0.915542003	0.010398504	ENSMUSG00000009406	-0.939276294	0.005419097
mmu-miR-7667-3p	ENSMUSG00000094936;	-0.915542003	0.010398504	ENSMUSG00000018672	-0.884146767	0.019355468
mmu-miR-7667-3p	ENSMUSG00000094936;	-0.915542003	0.010398504	ENSMUSG00000042590	-0.889133722	0.01775565

mmu-miR-7667-3p	ENSMUSG00000094936;	-0.915542003	0.010398504	ENSMUSG00000000776	-0.896660605	0.015466764
mmu-miR-7667-5p	ENSMUSG00000041852;	-0.85761761	0.028965877	ENSMUSG00000040479	-0.982315945	0.000466324
mmu-miR-7667-5p	ENSMUSG00000041852;	-0.85761761	0.028965877	ENSMUSG00000028124	-0.916678424	0.010124498
mmu-miR-7667-5p	ENSMUSG00000041852;	-0.85761761	0.028965877	ENSMUSG00000029919	-0.854633883	0.030161074
mmu-miR-7667-5p	ENSMUSG00000041852;	-0.85761761	0.028965877	ENSMUSG00000031953	-0.898197069	0.015018221
mmu-miR-7667-5p	ENSMUSG00000041852;	-0.85761761	0.028965877	ENSMUSG00000039257	-0.867045768	0.025340137
mmu-miR-7667-5p	ENSMUSG00000041852;	-0.85761761	0.028965877	ENSMUSG00000045248	-0.9058876	0.012868932
mmu-miR-7667-5p	ENSMUSG00000041852;	-0.85761761	0.028965877	ENSMUSG00000052551	-0.895955096	0.015674852
mmu-miR-7667-5p	ENSMUSG00000041852;	-0.85761761	0.028965877	ENSMUSG00000055235	-0.856866184	0.029264722
mmu-miR-7667-5p	ENSMUSG00000041852;	-0.85761761	0.028965877	ENSMUSG00000029468	-0.932424018	0.006695477
mmu-miR-7667-5p	ENSMUSG00000041852;	-0.85761761	0.028965877	ENSMUSG00000028960	-0.917213589	0.009996693
mmu-miR-7667-5p	ENSMUSG00000041852;	-0.85761761	0.028965877	ENSMUSG00000009073	-0.972412745	0.001131087
mmu-miR-7667-5p	ENSMUSG00000041852;	-0.85761761	0.028965877	ENSMUSG00000024908	-0.898566454	0.014911333
mmu-miR-7667-5p	ENSMUSG00000041852;	-0.85761761	0.028965877	ENSMUSG00000093485	-0.893975588	0.016265844
mmu-miR-7667-5p	ENSMUSG00000041852;	-0.85761761	0.028965877	ENSMUSG00000033697	-0.851472869	0.031452186
mmu-miR-7667-5p	ENSMUSG00000026134;	-0.858078636	0.028783243	ENSMUSG00000040479	-0.982315945	0.000466324
mmu-miR-7667-5p	ENSMUSG00000026134;	-0.858078636	0.028783243	ENSMUSG00000028124	-0.916678424	0.010124498
mmu-miR-7667-5p	ENSMUSG00000026134;	-0.858078636	0.028783243	ENSMUSG00000029919	-0.854633883	0.030161074
mmu-miR-7667-5p	ENSMUSG00000026134;	-0.858078636	0.028783243	ENSMUSG00000031953	-0.898197069	0.015018221
mmu-miR-7667-5p	ENSMUSG00000026134;	-0.858078636	0.028783243	ENSMUSG00000039257	-0.867045768	0.025340137
mmu-miR-7667-5p	ENSMUSG00000026134;	-0.858078636	0.028783243	ENSMUSG00000045248	-0.9058876	0.012868932
mmu-miR-7667-5p	ENSMUSG00000026134;	-0.858078636	0.028783243	ENSMUSG00000052551	-0.895955096	0.015674852
mmu-miR-7667-5p	ENSMUSG00000026134;	-0.858078636	0.028783243	ENSMUSG00000055235	-0.856866184	0.029264722
mmu-miR-7667-5p	ENSMUSG00000026134;	-0.858078636	0.028783243	ENSMUSG00000029468	-0.932424018	0.006695477
mmu-miR-7667-5p	ENSMUSG00000026134;	-0.858078636	0.028783243	ENSMUSG00000028960	-0.917213589	0.009996693
mmu-miR-7667-5p	ENSMUSG00000026134;	-0.858078636	0.028783243	ENSMUSG00000009073	-0.972412745	0.001131087
mmu-miR-7667-5p	ENSMUSG00000026134;	-0.858078636	0.028783243	ENSMUSG00000024908	-0.898566454	0.014911333
mmu-miR-7667-5p	ENSMUSG00000026134;	-0.858078636	0.028783243	ENSMUSG00000093485	-0.893975588	0.016265844
mmu-miR-7667-5p	ENSMUSG00000026134;	-0.858078636	0.028783243	ENSMUSG00000033697	-0.851472869	0.031452186
mmu-miR-7668-5p	ENSMUSG00000038784;	-0.89885011	0.014829503	ENSMUSG00000029538	-0.884214843	0.019333184
mmu-miR-7686-5p	ENSMUSG00000034007;	-0.920375072	0.009257777	ENSMUSG00000027950	-0.95863831	0.002530803
mmu-miR-7686-5p	ENSMUSG00000034007;	-0.920375072	0.009257777	ENSMUSG00000078139	-0.855685797	0.029737097
mmu-miR-7686-5p	ENSMUSG00000034007;	-0.920375072	0.009257777	ENSMUSG00000038437	-0.893869497	0.016297815
mmu-miR-7686-5p	ENSMUSG00000034007;	-0.920375072	0.009257777	ENSMUSG00000005338	-0.859307809	0.028298988
mmu-miR-7686-5p	ENSMUSG00000034007;	-0.920375072	0.009257777	ENSMUSG00000048351	-0.952977658	0.003264665
mmu-miR-7686-5p	ENSMUSG00000034007;	-0.920375072	0.009257777	ENSMUSG00000003308	-0.942434798	0.00487525
mmu-miR-7686-5p	ENSMUSG00000034007;	-0.920375072	0.009257777		-0.851127962	0.031594609
mmu-miR-7688-5p	ENSMUSG00000020422;	-0.904530707	0.013236507	ENSMUSG00000025139	-0.92861511	0.007461822

mmu-miR-7688-5p	ENSMUSG00000020422;	-0.904530707	0.013236507	ENSMUSG00000078139	-0.860039405	0.02801261
mmu-miR-7688-5p	ENSMUSG00000020422;	-0.904530707	0.013236507	ENSMUSG00000011096	-0.907534253	0.012429584
mmu-miR-7688-5p	ENSMUSG00000020422;	-0.904530707	0.013236507		-0.952303352	0.003358201
mmu-miR-8096	ENSMUSG00000040785;	-0.945507172	0.004373295	ENSMUSG00000021218	-0.934382092	0.006317299
mmu-miR-8097	ENSMUSG00000033102;	-0.9446359	0.004512925	ENSMUSG00000063882	-0.924467002	0.008342384
mmu-miR-8097	ENSMUSG00000033102;	-0.9446359	0.004512925	ENSMUSG00000021843	-0.949590638	0.003747608
mmu-miR-8097	ENSMUSG00000033102;	-0.9446359	0.004512925	ENSMUSG00000021843	-0.909000688	0.012044535
mmu-miR-8097	ENSMUSG00000033102;	-0.9446359	0.004512925		-0.865181762	0.026038711
mmu-miR-8103	ENSMUSG00000041852;	-0.881051625	0.02038159	ENSMUSG00000002748	-0.877326492	0.021650141
mmu-miR-8103	ENSMUSG00000041852;	-0.881051625	0.02038159	ENSMUSG00000025417	-0.956753792	0.002764911
mmu-miR-8103	ENSMUSG00000041852;	-0.881051625	0.02038159	ENSMUSG00000020592	-0.851010171	0.031643318
mmu-miR-8103	ENSMUSG00000041852;	-0.881051625	0.02038159	ENSMUSG00000022098	-0.932774937	0.006626912
mmu-miR-8103	ENSMUSG00000041852;	-0.881051625	0.02038159	ENSMUSG00000029148	-0.988529048	0.000196619
mmu-miR-8103	ENSMUSG00000041852;	-0.881051625	0.02038159	ENSMUSG00000032312	-0.872876254	0.023213482
mmu-miR-8103	ENSMUSG00000041852;	-0.881051625	0.02038159	ENSMUSG00000039367	-0.865970669	0.02574195
mmu-miR-8103	ENSMUSG00000041852;	-0.881051625	0.02038159	ENSMUSG00000050107	-0.866126386	0.025683566
mmu-miR-8103	ENSMUSG00000041852;	-0.881051625	0.02038159	ENSMUSG00000027593	-0.956030411	0.002857483
mmu-miR-8103	ENSMUSG00000041852;	-0.881051625	0.02038159	ENSMUSG00000063406	-0.876366433	0.021983003
mmu-miR-8103	ENSMUSG00000041852;	-0.881051625	0.02038159	ENSMUSG00000052533	-0.916207162	0.010237695
mmu-miR-8103	ENSMUSG00000041852;	-0.881051625	0.02038159	ENSMUSG00000026842	-0.985689994	0.000305699
mmu-miR-8103	ENSMUSG00000041852;	-0.881051625	0.02038159	ENSMUSG00000035929	-0.927968315	0.007595975
mmu-miR-8103	ENSMUSG00000041852;	-0.881051625	0.02038159	ENSMUSG00000032249	-0.890739907	0.017254491
mmu-miR-8103	ENSMUSG00000041852;	-0.881051625	0.02038159	ENSMUSG00000059901	-0.910193713	0.011735602
mmu-miR-8103	ENSMUSG00000041852;	-0.881051625	0.02038159	ENSMUSG00000024621	-0.875962774	0.022123679
mmu-miR-8103	ENSMUSG00000041852;	-0.881051625	0.02038159	ENSMUSG00000020340	-0.878136714	0.021371115
mmu-miR-8103	ENSMUSG00000041852;	-0.881051625	0.02038159	ENSMUSG00000030680	-0.876258285	0.022020651
mmu-miR-8103	ENSMUSG00000041852;	-0.881051625	0.02038159	ENSMUSG00000021917	-0.908698562	0.012123387
mmu-miR-874-3p	ENSMUSG00000074497;	-0.90438587	0.013276038	ENSMUSG00000026792	-0.85499427	0.0300155
mmu-miR-874-3p	ENSMUSG00000074497;	-0.90438587	0.013276038	ENSMUSG00000052406	-0.974211705	0.000988979
mmu-miR-874-3p	ENSMUSG00000074497;	-0.90438587	0.013276038	ENSMUSG00000037916	-0.859719941	0.028137492
mmu-miR-874-5p	ENSMUSG00000037174;	-0.921552671	0.008989593	ENSMUSG00000018672	-0.856585994	0.029376527
mmu-miR-874-5p	ENSMUSG00000037174;	-0.921552671	0.008989593	ENSMUSG00000036989	-0.884839927	0.019129146
mmu-miR-877-3p	ENSMUSG00000038648;	-0.856266747	0.029504161	ENSMUSG00000017446	-0.898838105	0.014832962
mmu-miR-877-3p	ENSMUSG00000038648;	-0.856266747	0.029504161	ENSMUSG00000040687	-0.904446894	0.013259375
mmu-miR-877-3p	ENSMUSG00000038648;	-0.856266747	0.029504161	ENSMUSG00000027950	-0.960918897	0.002261154
mmu-miR-877-3p	ENSMUSG00000030201;	-0.859102037	0.028379784	ENSMUSG00000017446	-0.898838105	0.014832962
mmu-miR-877-3p	ENSMUSG00000030201;	-0.859102037	0.028379784	ENSMUSG00000040687	-0.904446894	0.013259375
mmu-miR-877-3p	ENSMUSG00000030201;	-0.859102037	0.028379784	ENSMUSG00000027950	-0.960918897	0.002261154

mmu-miR-93-5p	ENSMUSG00000062604;	-0.927491851	0.007695544		-0.890849254	0.017220623
mmu-miR-9769-3p	ENSMUSG00000001376;	-0.912863487	0.011058354	ENSMUSG00000078762	-0.949423662	0.003772263
mmu-miR-9769-3p	ENSMUSG00000001376;	-0.912863487	0.011058354	ENSMUSG00000018750	-0.895906139	0.015689342
mmu-miR-9769-3p	ENSMUSG00000001376;	-0.912863487	0.011058354	ENSMUSG00000038712	-0.910051115	0.011772323
mmu-miR-98-5p	ENSMUSG00000048410;	-0.962072847	0.002130425	ENSMUSG00000044250	-0.902216805	0.01387485
mmu-miR-98-5p	ENSMUSG00000048410;	-0.962072847	0.002130425		-0.887918739	0.01813932
novel_200	ENSMUSG00000029313;	-0.910179452	0.011739272	ENSMUSG00000073421	-0.876714668	0.021861986
novel_298	ENSMUSG00000094936;	-0.864746232	0.026203235	ENSMUSG00000020232	-0.943868125	0.004637751
novel_298	ENSMUSG00000094936;	-0.864746232	0.026203235	ENSMUSG00000059939	-0.924316084	0.008375322
novel_298	ENSMUSG00000094936;	-0.864746232	0.026203235	ENSMUSG00000045374	-0.914739381	0.010594164
novel_298	ENSMUSG00000094936;	-0.864746232	0.026203235	ENSMUSG00000045374	-0.854245853	0.030318187
novel_331	ENSMUSG00000035247;	-0.883904891	0.019434742	ENSMUSG00000022711	-0.929678541	0.007243788
novel_331	ENSMUSG00000035247;	-0.883904891	0.019434742	ENSMUSG00000028378	-0.85629572	0.029492567
novel_331	ENSMUSG00000035247;	-0.883904891	0.019434742	ENSMUSG00000030867	-0.993493603	6.34E-05
novel_331	ENSMUSG00000035247;	-0.883904891	0.019434742	ENSMUSG00000031986	-0.896920389	0.015390478
novel_331	ENSMUSG00000035247;	-0.883904891	0.019434742	ENSMUSG00000035885	-0.883577263	0.019542369
novel_331	ENSMUSG00000035247;	-0.883904891	0.019434742	ENSMUSG00000029462	-0.868184133	0.024917956
novel_331	ENSMUSG00000035247;	-0.883904891	0.019434742	ENSMUSG00000040725	-0.914715153	0.010600098
novel_335	ENSMUSG00000024566;	-0.859188083	0.028345985		-0.851127962	0.031594609
novel_341	ENSMUSG00000041852;	-0.91979719	0.009390784	ENSMUSG00000009406	-0.930509934	0.007075525
novel_341	ENSMUSG00000041852;	-0.91979719	0.009390784	ENSMUSG00000021506	-0.929923662	0.007193978
novel_341	ENSMUSG00000041852;	-0.91979719	0.009390784	ENSMUSG00000028540	-0.85557174	0.029782931
novel_341	ENSMUSG00000041852;	-0.91979719	0.009390784	ENSMUSG00000036985	-0.960529746	0.002306106
novel_341	ENSMUSG00000041852;	-0.91979719	0.009390784	ENSMUSG00000042626	-0.910707309	0.011603804
novel_341	ENSMUSG00000041852;	-0.91979719	0.009390784	ENSMUSG00000040680	-0.9339399	0.006401764
novel_341	ENSMUSG00000041852;	-0.91979719	0.009390784	ENSMUSG00000043333	-0.910055762	0.011771126
novel_341	ENSMUSG00000041852;	-0.91979719	0.009390784	ENSMUSG00000003198	-0.88882594	0.01785247
novel_341	ENSMUSG00000041852;	-0.91979719	0.009390784	ENSMUSG00000032593	-0.89717853	0.015314855
novel_341	ENSMUSG00000041852;	-0.91979719	0.009390784	ENSMUSG00000020387	-0.951941867	0.003408879
novel_341	ENSMUSG00000041852;	-0.91979719	0.009390784	ENSMUSG00000015149	-0.853903643	0.030457067
novel_347	ENSMUSG00000022498;	-0.871651012	0.023653018	ENSMUSG00000015484	-0.917941763	0.00982406
novel_347	ENSMUSG00000022498;	-0.871651012	0.023653018	ENSMUSG00000023286	-0.878894976	0.021111551
novel_347	ENSMUSG00000022498;	-0.871651012	0.023653018	ENSMUSG00000024524	-0.928520118	0.007481452
novel_347	ENSMUSG00000022498;	-0.871651012	0.023653018	ENSMUSG00000048965	-0.85297842	0.030834056
novel_347	ENSMUSG00000022498;	-0.871651012	0.023653018	ENSMUSG00000001472	-0.950576349	0.003603682
novel_347	ENSMUSG00000022498;	-0.871651012	0.023653018	ENSMUSG00000034157	-0.886661208	0.018540566
novel_347	ENSMUSG00000022498;	-0.871651012	0.023653018	ENSMUSG00000059895	-0.919006334	0.009574303
